# Trends in future health financing and coverage: future health spending and universal health coverage in 188 countries, 2016–40

**DOI:** 10.1016/S0140-6736(18)30697-4

**Published:** 2018-05-05

**Authors:** Global Burden of Disease Health Financing Collaborator Network, Joseph L Dieleman, Nafis Sadat, Angela Y Chang, Nancy Fullman, Cristiana Abbafati, Pawan Acharya, Arsène Kouablan Adou, Aliasghar Ahmad Kiadaliri, Khurshid Alam, Reza Alizadeh-Navaei, Ala'a Alkerwi, Walid Ammar, Carl Abelardo T Antonio, Olatunde Aremu, Solomon Weldegebreal Asgedom, Tesfay Mehari Atey, Leticia Avila-Burgos, Rakesh Ayer, Hamid Badali, Maciej Banach, Amrit Banstola, Aleksandra Barac, Abate Bekele Belachew, Charles Birungi, Nicola L Bragazzi, Nicholas J K Breitborde, Lucero Cahuana-Hurtado, Josip Car, Ferrán Catalá-López, Abigail Chapin, Catherine S Chen, Lalit Dandona, Rakhi Dandona, Ahmad Daryani, Samath D Dharmaratne, Manisha Dubey, Dumessa Edessa, Erika Eldrenkamp, Babak Eshrati, André Faro, Andrea B Feigl, Ama P Fenny, Florian Fischer, Nataliya Foigt, Kyle J Foreman, Mamata Ghimire, Srinivas Goli, Alemayehu Desalegne Hailu, Samer Hamidi, Hilda L Harb, Simon I Hay, Delia Hendrie, Gloria Ikilezi, Mehdi Javanbakht, Denny John, Jost B Jonas, Alexander Kaldjian, Amir Kasaeian, Yawukal Chane Kasahun, Ibrahim A Khalil, Young-Ho Khang, Jagdish Khubchandani, Yun Jin Kim, Jonas M Kinge, Soewarta Kosen, Kristopher J Krohn, G Anil Kumar, Alessandra Lafranconi, Hilton Lam, Stefan Listl, Hassan Magdy Abd El Razek, Mohammed Magdy Abd El Razek, Azeem Majeed, Reza Malekzadeh, Deborah Carvalho Malta, Gabriel Martinez, George A Mensah, Atte Meretoja, Angela Micah, Ted R Miller, Erkin M Mirrakhimov, Fitsum Weldegebreal Mlashu, Ebrahim Mohammed, Shafiu Mohammed, Mark Moses, Seyyed Meysam Mousavi, Mohsen Naghavi, Vinay Nangia, Frida Namnyak Ngalesoni, Cuong Tat Nguyen, Trang Huyen Nguyen, Yirga Niriayo, Mehdi Noroozi, Mayowa O Owolabi, Tejas Patel, David M Pereira, Suzanne Polinder, Mostafa Qorbani, Anwar Rafay, Alireza Rafiei, Vafa Rahimi-Movaghar, Rajesh Kumar Rai, Usha Ram, Chhabi Lal Ranabhat, Sarah E Ray, Robert C Reiner, Haniye Sadat Sajadi, Rocco Santoro, João Vasco Santos, Abdur Razzaque Sarker, Benn Sartorius, Maheswar Satpathy, Sadaf G Sepanlou, Masood Ali Shaikh, Mehdi Sharif, Jun She, Aziz Sheikh, Mark G Shrime, Mekonnen Sisay, Samir Soneji, Moslem Soofi, Reed J D Sorensen, Henok Tadesse, Tianchan Tao, Tara Templin, Azeb Gebresilassie Tesema, Subash Thapa, Ruoyan Tobe-Gai, Roman Topor-Madry, Bach Xuan Tran, Khanh Bao Tran, Tung Thanh Tran, Eduardo A Undurraga, Tommi Vasankari, Francesco S Violante, Andrea Werdecker, Tissa Wijeratne, Gelin Xu, Naohiro Yonemoto, Mustafa Z Younis, Chuanhua Yu, Maysaa El Sayed Zaki, Bianca Zlavog, Christopher J L Murray

## Abstract

**Background:**

Achieving universal health coverage (UHC) requires health financing systems that provide prepaid pooled resources for key health services without placing undue financial stress on households. Understanding current and future trajectories of health financing is vital for progress towards UHC. We used historical health financing data for 188 countries from 1995 to 2015 to estimate future scenarios of health spending and pooled health spending through to 2040.

**Methods:**

We extracted historical data on gross domestic product (GDP) and health spending for 188 countries from 1995 to 2015, and projected annual GDP, development assistance for health, and government, out-of-pocket, and prepaid private health spending from 2015 through to 2040 as a reference scenario. These estimates were generated using an ensemble of models that varied key demographic and socioeconomic determinants. We generated better and worse alternative future scenarios based on the global distribution of historic health spending growth rates. Last, we used stochastic frontier analysis to investigate the association between pooled health resources and UHC index, a measure of a country's UHC service coverage. Finally, we estimated future UHC performance and the number of people covered under the three future scenarios.

**Findings:**

In the reference scenario, global health spending was projected to increase from US$10 trillion (95% uncertainty interval 10 trillion to 10 trillion) in 2015 to $20 trillion (18 trillion to 22 trillion) in 2040. Per capita health spending was projected to increase fastest in upper-middle-income countries, at 4·2% (3·4–5·1) per year, followed by lower-middle-income countries (4·0%, 3·6–4·5) and low-income countries (2·2%, 1·7–2·8). Despite global growth, per capita health spending was projected to range from only $40 (24–65) to $413 (263–668) in 2040 in low-income countries, and from $140 (90–200) to $1699 (711–3423) in lower-middle-income countries. Globally, the share of health spending covered by pooled resources would range widely, from 19·8% (10·3–38·6) in Nigeria to 97·9% (96·4–98·5) in Seychelles. Historical performance on the UHC index was significantly associated with pooled resources per capita. Across the alternative scenarios, we estimate UHC reaching between 5·1 billion (4·9 billion to 5·3 billion) and 5·6 billion (5·3 billion to 5·8 billion) lives in 2030.

**Interpretation:**

We chart future scenarios for health spending and its relationship with UHC. Ensuring that all countries have sustainable pooled health resources is crucial to the achievement of UHC.

**Funding:**

The Bill & Melinda Gates Foundation.

## Introduction

Estimates of future global and national health spending are valuable inputs for health system planning and can guide progress towards achieving universal health coverage (UHC). UHC has emerged as both a global and national health priority, and progressive realisation of UHC is viewed as a critical path for improving health outcomes and achieving greater equity in health across all populations. Globally, the importance of UHC is highlighted by its codification in the Sustainable Development Goals (SDGs) in 2015, although its thematic origins come from the Alma Ata Declaration of 1978.^1^, ^2^ Nationally, the health benefits and protections against catastrophic health spending that result from UHC are highlighted by UHC exemplars such as Japan, Chile, and Thailand, and UHC initiatives or proposals are increasingly topping policy agendas.^3^, ^4^, ^5^, ^6^, ^7^ Numerous case studies have sought to identify key factors in achieving UHC and have posited several drivers, including sustained political will, clearly defined health service packages, and phased implementation to ensure that all populations are covered.^8^, ^9^ However, across development and health-care settings, it is increasingly recognised that creating and maintaining robust health financing systems is equally important to achieving UHC.

Achieving UHC for all populations requires the harmonisation of political, social, economic, and health leadership, as well as mature health systems capable of ensuring efficiency and equity. Furthermore, health financing systems must be able to deliver a sufficient set of pooled resources for health,^10^, ^11^ which requires sustaining sufficient supplies of resources to finance key health services at the country level. Pooled resources consist of prepaid revenues through government financing, social health insurance, private insurance, or development assistance for health (DAH), which help to mitigate individual-level financial risks across populations and thus fund care for more people. The cornerstones of UHC—providing access to essential health services for all populations and protection against catastrophic health spending—are best supported through the establishment of sufficient and stable supplies of pooled resources for health. Conversely, persistent challenges in the stability or sufficiency of pooled health resources, as well as reliance on out-of-pocket spending, can significantly hinder whether and how UHC can be successfully implemented, scaled up, and maintained. Tracking country-level pooled resources for health and understanding how trends in the availability of resources can affect UHC performance are vital inputs into policy dialogues and budgeting processes related to UHC.

**Research in context**

**Evidence before the study**

Achieving universal health coverage (UHC)—ie, access to essential health services and financial risk protection—is increasingly viewed as crucial to improving health outcomes. Despite the ascent of UHC in global and national policy agendas, little is known about how health financing might or might not constrain progressive realisation of this goal. Previous studies, including work by the Global Burden of Disease Financing Global Health Collaborator Network, have provided past estimates and predictions of total health spending and spending disaggregated by source (ie, government, out-of-pocket, and prepaid private spending and development assistance for health); however, these analyses have not directly examined how these financing trends might relate to UHC. At the country level, a key component of successful UHC programme design and sustainability is the existence of a sufficient, stable supply of pooled resources for health. The Global Burden of Diseases, Injuries, and Risk Factors Study (GBD) 2016 produced projections of health service coverage through to 2030 on the basis of past trends for 188 countries. Although an important first step, these estimates did not account for potential financing constraints. To better inform long-term investments for UHC initiatives and financing, it is crucial to understand how country-level pooled resources are related to gains in UHC performance.

**Added value of this study**

Drawing from past trends and relationships between key economic, demographic, and health financing indicators for 188 countries from 1995 to 2015, we estimated three future scenarios for health spending through to 2040. These scenarios consisted of a reference scenario, as well as better and worse scenarios, which constituted the 85th and 15th percentiles of long-term global health spending growth rates, respectively. We then assessed the relationship between pooled health resources per capita and performance on the UHC index, a summary measure of UHC service coverage developed as part of the GBD 2016 study. We used the relationship between financing and UHC index to evaluate the frontier for UHC achievement on the basis of past levels of health spending. The frontier represents the modeled optimal UHC index for each amount of pooled health spending per capita. We then applied the relationships identified to the three future health spending scenarios to quantify the possible trajectories and the extent to which they catalysed (or constrained) future gains in UHC by country, income group, and GBD super-region.

**Implications of all the available evidence**

Although per capita health spending was projected to significantly increase worldwide, such gains were varied, and most increases in health spending and pooled health spending were concentrated among upper-middle-income and high-income countries rather than lower-middle-income and low-income countries. By 2040, country-specific pooled spending per capita was projected to range from $30 to $14 876, a magnitude of disparity that could hinder progress on UHC for many places most in need. We found that greater pooled health resources per capita were positively related to the UHC index performance and could be a part of substantial gains in UHC. With 2015 as the baseline, our better and worse health spending scenarios projected that 0·8–1·3 billion additional lives could be covered with UHC service coverage by 2030 and 1·1–2·0 billion by 2040. These results not only emphasise the overall importance of sustained pooled resources for health, but also the potential to substantially accelerate global gains if the better financing scenarios can be actualised. Across the development spectrum, deliberate action focused on the expansion of pooled resources for health will be crucial to bringing the aspirations of UHC closer to reality for all populations.

Little is known about how financial resources for health might catalyse or constrain potential future progress on UHC. Previous studies have explored how to translate health resources into achieving UHC by offering cost estimates for UHC attainment. Although such studies can be useful for initial planning purposes, they often fail to account for system inefficiencies and implementation challenges associated with programmatic scale-up. Moreover, measures of health-service costs are fundamentally different from quantification of the amount of total health spending needed to implement and sustain national health systems. Other studies have tracked historical changes in total health spending and the associations between these changes and income, as well as retrospective relationships between public spending on health and UHC progress.^11^, ^12^, ^13^, ^14^, ^15^ However, such analyses do not shed light on what resources might be available in the future and how they can be leveraged to accelerate advances in UHC.

We used historical health financing data for 188 countries from 1995 to 2015 to estimate future scenarios of health spending and pooled health spending through to 2040. Additionally, we assessed past relationships between pooled health spending and performance on a measure of UHC service coverage. Last, we quantified the magnitude by which changes in health financing, as projected into the future, could lead to changes in UHC by 2030 and 2040—key information for the development of long-term policy and strategy to achieve UHC.

## Methods

### Overview

We estimated national GDP, government spending, health spending, and performance on the UHC index for each year through to 2040 for 188 countries. Our estimates are based on past trends and relationships from economic, demographic, and health financing data over time. The methods used build and improve on the methods from our previous research.^16^, ^17^ More detail on these methods and the data used in this analysis are provided later in this paper and in the appendix (pp 10–20). In brief, these methodological advances include the estimation of alternative (better and worse) future scenarios in addition to reference scenarios for each country; development of a structural framework to identify key covariates upon which to build our econometric models; and incorporation of several improvements to identify, rank, and pool the models that ultimately compose our final ensemble model and estimates of uncertainty.^18^ We then used these financing projections to estimate UHC index performance for each country-year through to 2040 using stochastic frontier analysis (SFA). All analyses were done with R Statistical Software and R-INLA, and visualisations were produced with ggplot2.^19^, ^20^, ^21^, ^22^, ^23^

### Data

We extracted health spending data for 188 countries spanning 1995–2015 from the Institute for Health Metrics and Evaluation's (IHME) Financing Global Health 2017 database.^24^ These data build on data published in the WHO Global Health Expenditure Database, as well as additional global health financing data such as National Health Accounts and project-level DAH data from 23 major channels of development assistance; additional details on how the Financing Global Health 2017 database was constructed are available elsewhere.^24^, ^25^ These data track current health spending (ie, net of investment spending) and are composed of four mutually exclusive categories: government health spending from domestic sources, which includes general government and social health insurance; prepaid private health spending, which includes private insurance and non-governmental organisation spending; out-of-pocket health spending, which includes all spending at point of service and copayments; and DAH, defined as the financial and in-kind resources transferred from development agencies to low-income and middle-income countries with the primary purpose of maintaining or improving health.^25^

We calculated GDP and government spending data for 188 countries from 1970 to 2017 using methods that have been described elsewhere.^26^ GDP and all spending estimates were modelled and reported in 2017 purchasing power parity US$ adjusted for inflation.

We used the UHC index, which was developed as part of the Global Burden of Diseases, Injuries, and Risk Factors Study (GBD) 2016.^27^ This index is a summary measure of health service coverage that is based on the coverage of nine interventions and risk-standardised rates of death from 32 causes amenable to health care. The nine interventions are primarily focused on infectious diseases and reproductive, maternal, and child health priority areas (ie, coverage of DPT3 vaccine, measles vaccine, polio vaccine, at least one antenatal visits, at least four antenatal visits, presence of a skilled-birth attendant, in-facility birth, antiretroviral therapy, and met need for family planning with modern contraception methods). The 32 causes of death were components of the Healthcare Access and Quality (HAQ) index, which represents a wider range of key health conditions, including cancers, stroke, and diabetes;^28^ although the UHC index draws from components of the HAQ Index, it is not exclusively composed of risk-standardised rates of death from causes amenable to health care. In 2016, UHC index values ranged from 85·7 (95% UI 82·0–89·3) in Switzerland to 26·9 (24·2–30·1) in Somalia. A full description of the UHC index components and its construction is available in the appendix (pp 21–24).^27^

### Reference case for future health spending

We estimated the annual growth rate of GDP from 2018 to 2040 using an ensemble modelling approach. Covariates for potential model inclusion were fraction of total population younger than 20 years and older than 65 years (separately), average years of education, total fertility rate, and a convergence term, which is the 1-year lag of the non-differenced dependent variable. Inclusion of the convergence term allows for models in which countries spending more on health have slower spending growth rates. This models converging amounts of spending per capita. We also included four distinct weight schemes to weight recent years more heavily, as well as up to three degrees of autoregressive terms and up to three degrees of a moving average residual. The combination of covariates and alternative models specifications leads to 11 520 potential models. We selected 825 of 11 520 possible model specifications, after excluding models with non-significant independent variables (p value greater than 0·10), an estimated coefficient on the convergence terms greater than zero, and predictions that fell outside the bounds of historical GDP growth from 1970 to 2017. More detail is provided in the appendix (p 15).

Out-of-pocket and prepaid private health spending, as well as total government spending were modelled as a share of GDP, whereas government health spending was modelled as a share of total government spending. We used the previously described ensemble modelling approach for each measure, and conducted this modelling in sequence, such that projected estimates were used as covariates in subsequent models, as shown in the appendix (pp 10, 11).^20^, ^21^

We used a three-step process to estimate the future DAH disbursed to low-income and middle-income countries. For sources of DAH that are countries or national treasuries, we modelled DAH as a share of the source's government spending to make estimates of total DAH provided from 2018 to 2040. For sources without an associated GDP time series, such as corporate donations and private foundations, we estimated future DAH using autoregressive integrated moving average (ARIMA) models with no covariates. Second, we modelled DAH received for each recipient country, measured as a share of the total amount of DAH provided through 2040. Finally, we estimated the transition of countries from middle-income to high-income status on the basis of GDP per capita. This transition occurs when GDP per capita surpasses $13 741 per capita, the point of high-income transition defined by the World Bank.^29^ To estimate total health spending for the reference scenario, we added DAH received by countries to country-level reference estimates for government, prepaid private, and out-of-pocket health spending.

### Alternative future health spending scenarios

In addition to generating a reference scenario for each country from 2016 to 2040, we estimated two sets of alternative health spending scenarios for total, government, prepaid private, and out-of-pocket spending and DAH. These alternative scenarios, termed the better and worse scenarios are associated with greater and fewer resources being available for health, respectively, compared with the reference scenario. Although many high-income countries might prefer to limit rather than expand health spending, from the vantage point of the health sector exclusively, having more resources for health is generally better. To inform these alternative scenarios, we first regressed 20 year growth rate of total health spending per capita from 1995 to 2015 on the convergence term. We then computed better and worse growth rates for each country by adding the country-specific fitted value and 85th or 15th percentiles of the estimated residual of the long-term growth rates, which resulted in better and worse future scenarios for total health spending per capita, by country, from 2016 to 2040. In cases where better or worse scenarios were higher or lower than the reference projections, we adjusted the respective scenarios to overlap with the reference case. We completed this process for government health, prepaid private, and out-of-pocket spending and DAH and scaled these estimates proportionally to the better and worse total health spending scenarios.

### Relationship between health spending and UHC index performance

To measure the relationship between health spending and UHC index performance, we used SFA to regress annual country-specific UHC index estimates on per capita pooled resources for health (ie, the sum of government health spending, prepaid private spending, and DAH). We selected SFA over other methods such as data envelopment analysis because SFA allowed us to incorporate measurement error and to draw from a wide range of peer countries to set the frontier—ie, the modelled optimal UHC index for each amount of pooled health spending per capita. The gap between the frontier and observed spending represents the sum of measurement error and unobserved factors related to the translation of health spending into gains in UHC.^15^ These factors, which we refer to collectively as inefficiency, reflect a range of influences, within and outside the health sector, that might ultimately prevent a country from reaching the UHC index frontier; such factors include social, political, demographic, and economic trends, as well as those within the health sector that can affect efficiency, such as governance and corruption. To model these unobserved factors, we assumed a one-sided half-normal distribution of residuals.

Using SFA, we estimated potential future performance on the UHC index based on the three pooled health spending scenarios (reference, better, and worse). We measured the gap between the frontier and observed UHC index at the country level by use of time series regression. Further details are provided in the appendix (pp 22–24).

The UHC index provides a good approximation of the average coverage of essential health services across a wide range of priority health areas. To estimate the number of people covered by UHC health services, we assumed the UHC index to be a coverage measure and multiplied the UHC index by each location-year-specific population. This assumption allows the aggregation across individuals estimated to be covered with a subset of the high-quality services.

We categorised potential drivers of change in UHC index performance into two distinct components, using the decomposition method described by Das Gupta: the change in UHC index performance associated with changes in pooled total health spending per capita and the change in performance associated with changing efficiency.^30^

### Aggregation across income groups and regions

We report health spending and UHC index performance for each country, World Bank 2017 income group, and GBD super-region.^29^, ^31^ We aggregated spending per capita or per GDP by calculating spending for the group relative to the total population or GDP; subsequently, these measures reflect the group or region as a whole instead of the average of countries comprising the given group. We used population-weighted means to measure the UHC index by income group or GBD super-region. We report all estimates up to 2040, and highlight some of the projected figures for 2030, given its significance as the target year for achieving SDGs. The full results for all time periods are presented in the appendix (pp 65–75).

### Uncertainty

We propagated uncertainty throughout our analysis. For reference scenarios, we used a four-part process to capture data, model, and parameter uncertainties. First, to propagate data uncertainty, we randomly selected different draws for historical data and covariates. Second, to propagate model uncertainty, we used the ensemble modelling framework. Third, to propagate parameter uncertainty, we took a random sample from the posterior distributions of each model specification to create at least 1000 draws. Fourth, we added empirical noise to our linear projections using a first-order random walk. The variance of the random walk was determined by estimated residuals from the out-of-sample validation. Countries where sub-models did not fit the observed data well have the largest uncertainty. For alternative scenarios, the uncertainty from the mean of reference scenarios was added to the better and worse scenarios. We completed the frontier analyses and future UHC estimation using each of the estimated draws. Each estimate reported in this text is a mean of these 1000 draws, and the uncertainty interval (UI) was constructed by extracting the range between the 2·5th and 97·5th percentile of the 1000 draws.

### Role of the funding source

The funder of this study had no role in the study design, data collection, data analysis, data interpretation, or writing of the report. All authors had full access to all the data in the study and the corresponding author had final responsibility for the decision to submit for publication.

## Results

In 2015, $10 trillion (95% UI 10 trillion to 10 trillion) was spent on health globally, and total health spending was projected to reach $15 trillion (14 trillion to 16 trillion) in 2030 and $20 trillion (18 trillion to 22 trillion) in 2040. In 2040, health spending per capita is expected to be 45·9 (37·1–54·6) times larger in high-income countries than in countries that are considered low-income currently. Across the four income groups, we estimated that health spending per capita in 2040 would be $8666 (7430–9657) for high-income countries, $2670 (2217–3302) for upper-middle-income countries, $714 (638–801) for lower-middle-income countries, and $190 (166–219) for low-income countries (table 1). Within the income groups, expected spending on health also varied dramatically, with per capita health spending projected to range from only $40 (24–65) to $413 (263–668) in 2040 in low-income countries, and from $140 (90–200) to $1699 (711–3423) in lower-middle-income countries. Per-capita spending was projected to increase in 174 of 188 countries, with the largest increases in spending in upper-middle-income-countries (4·2%, 3·4–5·1).

**Table 1:** Health spending, health spending by source, and growth, 2015–40

		**Total health spending per capita ($)**		**Health spending as a proportion of total, 2040**				**Per capita annualised rate of change, 2015–40**				
		2015	2040	Government spending	Pre-paid private spending	Out-of-pocket spending	Development assistance for health	Total (%)	Government spending (%)	Pre-paid private spending (%)	Out-of-pocket spending (%)	Development assistance for health (%)
Global		1332 (1325 to 1343)	2318 (2099 to 2540)	61·3% (57·2 to 66·3)	13·5% (8·3 to 16·9)	24·7% (21·9 to 27·6)	0·5% (0·5 to 0·6)	2·2% (1·8 to 2·6)	2·3% (1·9 to 2·9)	1·1% (−0·9 to 2·1)	2·6% (2·3 to 3·0)	2·3% (1·9 to 2·9)
World Bank income group												
	High income	5551 (5503 to 5605)	8666 (7430 to 9657)	67·3% (61·7 to 76·1)	19·2% (9·9 to 24·8)	13·4% (11·5 to 16·0)	0·0% (0·0 to 0·0)	1·8% (1·2 to 2·2)	2·0% (1·5 to 2·5)	1·2% (−1·7 to 2·5)	1·6% (1·1 to 2·1)	..
	Upper-middle income	949 (942 to 959)	2670 (2217 to 3302)	64·2% (56·7 to 71·3)	6·9% (4·7 to 10·1)	28·8% (22·4 to 35·5)	0·1% (0·1 to 0·2)	4·2% (3·4 to 5·1)	4·6% (3·5 to 5·9)	2·6% (1·2 to 4·3)	3·7% (3·0 to 4·5)	1·6% (−0·1 to 3·4)
	Lower-middle income	266 (263 to 268)	714 (638 to 801)	31·9% (27·5 to 37·1)	8·4% (6·3 to 10·8)	57·9% (52·7 to 63·0)	1·8% (1·4 to 2·2)	4·0% (3·6 to 4·5)	4·0% (3·3 to 4·7)	4·5% (3·4 to 5·6)	4·0% (3·3 to 4·8)	1·8% (1·1 to 2·5)
	Low income	110 (108 to 111)	190 (166 to 219)	29·8% (23·2 to 37·7)	11·8% (6·9 to 20·2)	35·7% (29·7 to 41·7)	22·7% (18·6 to 26·7)	2·2% (1·7 to 2·8)	3·5% (2·3 to 4·9)	4·1% (1·9 to 7·0)	1·8% (1·2 to 2·6)	1·0% (0·3 to 1·8)
GBD super-regions												
	Central Europe, eastern Europe, and central Asia	1288 (1273 to 1300)	2120 (1847 to 2427)	56·3% (49·5 to 62·7)	3·3% (2·4 to 4·3)	39·9% (33·4 to 46·9)	0·5% (0·3 to 0·6)	2·0% (1·4 to 2·6)	1·6% (0·9 to 2·4)	2·4% (1·3 to 3·5)	2·5% (1·7 to 3·5)	4·1% (3·1 to 5·4)
	GBD high income	5839 (5785 to 5897)	9054 (7715 to 10 101)	67·5% (61·8 to 76·9)	19·6% (9·9 to 25·5)	12·8% (10·9 to 15·5)	0·0% (0·0 to 0·0)	1·8% (1·1 to 2·2)	2·0% (1·5 to 2·5)	1·1% (−1·9 to 2·4)	1·5% (1·0 to 2·0)	−40·0% (−77·4 to 1·5)
	Latin America and Caribbean	1065 (1051 to 1077)	1550 (1356 to 1751)	51·2% (44·9 to 57·6)	18·6% (12·4 to 23·7)	29·9% (25·1 to 35·7)	0·3% (0·2 to 0·6)	1·5% (1·0 to 2·0)	1·6% (0·8 to 2·4)	1·7% (0·1 to 2·8)	1·2% (0·6 to 2·0)	−1·7% (−3·7 to 0·9)
	North Africa and Middle East	888 (872 to 905)	1496 (1254 to 1806)	56·9% (48·5 to 65·4)	7·8% (4·8 to 12·3)	34·9% (27·5 to 42·8)	0·4% (0·3 to 0·6)	2·1% (1·4 to 2·9)	1·9% (0·8 to 3·1)	2·3% (0·6 to 4·3)	2·3% (1·4 to 3·4)	1·9% (0·7 to 3·3)
	South Asia	210 (207 to 212)	692 (587 to 828)	28·9% (22·3 to 36·6)	9·9% (6·2 to 14·4)	60·6% (52·8 to 67·9)	0·6% (0·4 to 0·9)	4·9% (4·2 to 5·6)	5·4% (4·1 to 6·6)	5·8% (3·8 to 7·6)	4·6% (3·7 to 5·6)	0·0% (−1·3 to 1·6)
	Southeast Asia, east Asia, and Oceania	672 (663 to 682)	2632 (2015 to 3454)	63·6% (53·8 to 72·9)	5·3% (3·0 to 9·0)	31·0% (22·6 to 40·4)	0·1% (0·1 to 0·2)	5·6% (4·5 to 6·8)	6·1% (4·4 to 7·7)	3·5% (1·4 to 6·0)	5·0% (4·1 to 6·0)	1·7% (0·6 to 3·2)
	Sub-Saharan Africa	202 (199 to 206)	289 (260 to 327)	34·5% (28·9 to 41·1)	11·0% (8·1 to 15·7)	39·4% (33·4 to 45·0)	15·1% (12·7 to 17·5)	1·4% (1·0 to 1·9)	1·4% (0·6 to 2·4)	0·0% (−1·2 to 1·6)	2·1% (1·3 to 2·9)	1·3% (0·7 to 1·9)

Spending is measured in 2017 purchasing-power parity-adjusted dollars. Income groups are the 2017 World Bank income groups, held constant across time. Estimates in parentheses are uncertainty intervals. Projections are based on the reference scenario. 2030 values and country-specific results are available in the appendix. GBD=Global Burden of Disease.

**Figure 1:**
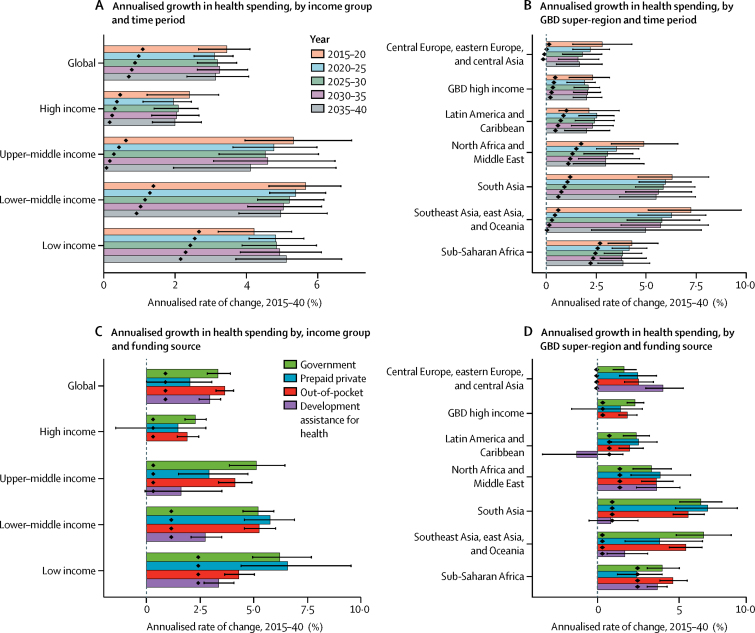
Annualised growth in total health spending per capita Per capita spending is measured in 2017 purchasing-power parity-adjusted dollars adjusted for inflation. Income groups are based on 2017 World Bank income groups held constant across time. Black lines represent uncertainty intervals. Projections are based on the reference scenario. Black diamonds show expected population growth rates. GBD=Global Burden of Disease.

Figure 1 shows the estimated growth rates across time and spending category globally, for each income group and GBD super-region. Globally, growth rates for total spending were expected to be relatively constant across the next 25 years, with an average of 3·0% (95% UI 2·6–3·4) over time. The largest growth rates were for out-of-pocket spending, followed by government health spending and DAH. Growth rates for per-capita spending, however, were expected to decrease over time in all GBD super-regions and income groups except low-income. Across income groups, the highest average annual growth rates for total spending over time were estimated to occur in low-income countries (5·0%, 4·5–5·7) and lower-middle-income countries (4·9%, 4·5–5·4); the largest annualised growth rates were for prepaid private spending and government health spending. Despite having the largest health spending growth rate, health spending per-capita growth in low-income countries was projected to remain low (table 1). The region of southeast Asia, east Asia, and Oceania was projected to have the highest average annual growth over time (5·6%, 4·5–6·8), followed by south Asia (5·3%, 4·7–6·1), although growth declined across time. The highest growth rates were for prepaid private spending in south Asia and government health spending in southeast Asia, east Asia, and Oceania. The GBD high-income region was estimated to have the lowest total spending growth (1·9%, 1·3–2·4).

Focusing on the growth of per-capita pooled spending (ie, government health spending, prepaid private spending, and DAH) we estimated a projected global growth rate of 2·0% (95% UI 0·5–3·4) per year for 2015–40. In contrast, out-of-pocket spending was projected to increase at an annualised rate of 2·6% (2·3–3·0) from 2015 to 2040 (table 1). Upper-middle-income countries had the largest rate of growth in pooled spending per-capita, at 4·3% per year (2·4–6·1), whereas lower-middle-income countries were also projected to have substantial growth in pooled spending, at 3·9% per year (2·2–5·6). By 2040, it was estimated that pooled resources in these countries would reach only 42·1% (37·0–47·4) of total health spending and remain at $300 per capita (261–345; table 2). Under the reference scenario, high-income countries had the largest projected pooled health spending per capita ($7508, 5192–9814) in 2040. Conversely, the lowest projections for pooled health spending per capita in 2040 were in sub-Saharan Africa ($175, 152–207), and south Asia ($273, 211–342).

**Table 2:** Three scenarios of pooled health spending, UHC index, and covered lives in 2030

		**Pooled health spending per capita ($)**				**Universal health coverage index**				**Lives covered (millions)**			
		2015 observed	2030 worse scenario	2030 reference scenario	2030 better scenario	2015 observed	2030 worse scenario	2030 reference scenario	2030 better scenario	2015 observed	2030 worse scenario	2030 reference scenario	2030 better scenario
Global		1036 (999 to 1076)	989 (747 to 1256)	1401 (1015 to 1818)	1917 (1414 to 2468)	59·2 (58·2 to 60·1)	61·4 (58·7 to 63·5)	64·8 (61·8 to 67·0)	67·1 (64·1 to 69·5)	4325 (4250 to 4390)	5109 (4887 to 5283)	5390 (5147 to 5579)	5586 (5335 to 5782)
World Bank Income Groups													
	High income	4768 (4605 to 4941)	4775 (3755 to 5762)	6213 (4653 to 7613)	8950 (6874 to 10912)	76·8 (75·7 to 77·6)	77·8 (75·6 to 79·4)	79·9 (77·3 to 81·8)	84·5 (81·9 to 86·5)	893 (880 to 902)	942 (915 to 962)	967 (936 to 990)	1023 (992 to 1047)
	Upper-middle income	646 (622 to 672)	715 (488 to 1011)	1251 (850 to 1787)	1537 (1038 to 2193)	65·6 (64·5 to 66·6)	67·1 (64·0 to 69·8)	72·4 (68·9 to 75·4)	74·3 (70·8 to 77·4)	1677 (1649 to 1702)	1788 (1705 to 1860)	1929 (1838 to 2009)	1982 (1888 to 2064)
	Lower-middle income	113 (106 to 120)	136 (94 to 191)	205 (143 to 287)	254 (174 to 362)	50·3 (49·1 to 51·5)	55·2 (52·6 to 57·1)	58·2 (55·3 to 60·2)	59·9 (56·9 to 62·0)	1482 (1445 to 1516)	1912 (1822 to 1976)	2014 (1917 to 2085)	2074 (1971 to 2146)
	Low income	67 (63 to 72)	74 (44 to 122)	94 (54 to 157)	141 (80 to 238)	42·7 (41·6 to 43·9)	47·5 (44·7 to 50·5)	48·7 (45·7 to 51·8)	51·5 (48·2 to 55·0)	273 (266 to 281)	467 (439 to 497)	479 (449 to 510)	507 (474 to 540)
GBD super-regions													
	Central Europe, eastern Europe, and central Asia	839 (801 to 885)	918 (652 to 1261)	1096 (756 to 1534)	1677 (1175 to 2336)	63·8 (61·9 to 65·6)	67·3 (63·8 to 70·3)	68·6 (64·8 to 71·9)	72·9 (68·9 to 76·2)	263 (256 to 271)	282 (268 to 295)	288 (272 to 302)	306 (289 to 320)
	GBD high income	5036 (4873 to 5208)	5015 (3974 to 5988)	6538 (4929 to 7925)	9403 (7278 to 11 338)	77·0 (75·8 to 77·8)	77·5 (75·5 to 79·1)	79·6 (77·1 to 81·4)	84·2 (81·7 to 86·1)	812 (800 to 821)	853 (831 to 871)	876 (849 to 896)	927 (900 to 947)
	Latin America and Caribbean	723 (693 to 755)	721 (493 to 960)	913 (611 to 1231)	1442 (960 to 1948)	60·7 (59·5 to 61·7)	62·5 (59·6 to 64·5)	64·3 (61·2 to 66·5)	68·3 (65·0 to 70·6)	344 (337 to 349)	403 (385 to 416)	415 (395 to 429)	441 (420 to 456)
	North Africa and Middle East	597 (560 to 638)	639 (362 to 1019)	823 (449 to 1344)	1182 (648 to 1925)	59·5 (58·5 to 60·6)	63·5 (59·8 to 67·1)	65·3 (61·2 to 69·3)	68·8 (64·5 to 72·9)	336 (330 to 342)	447 (421 to 473)	460 (432 to 489)	485 (455 to 514)
	South Asia	74 (71 to 77)	94 (70 to 123)	167 (124 to 219)	175 (129 to 231)	48·8 (47·1 to 50·2)	54·6 (52·3 to 56·5)	58·5 (56·0 to 60·6)	59·1 (56·5 to 61·2)	820 (792 to 844)	1021 (978 to 1057)	1094 (1047 to 1133)	1105 (1056 to 1145)
	Southeast Asia, east Asia, and Oceania	439 (423 to 457)	491 (350 to 691)	1080 (764 to 1532)	1143 (807 to 1621)	63·8 (62·7 to 64·7)	65·1 (62·4 to 67·4)	71·7 (68·7 to 74·3)	72·5 (69·5 to 75·1)	1320 (1298 to 1340)	1382 (1326 to 1432)	1522 (1459 to 1578)	1539 (1476 to 1596)
	Sub-Saharan Africa	134 (127 to 142)	131 (84 to 204)	155 (96 to 245)	258 (160 to 407)	45·1 (43·9 to 46·3)	49·4 (45·9 to 52·8)	50·3 (46·7 to 53·9)	53·7 (49·7 to 57·5)	430 (419 to 442)	720 (670 to 770)	734 (681 to 787)	783 (725 to 839)

Spending is measured in 2017 purchasing-power parity-adjusted dollars adjusted for inflation. Income groups are the 2017 World Bank income groups, held constant across time. Estimates in parentheses are uncertainty intervals. The reference scenario is based on past trends and relationships with key drivers of health spending, whereas the better and worse alternative scenarios show potential trajectories based on those observed historically. 2040 values and country-specific results are available in the supplementary appendix. GBD=Global Burden of Disease.

Globally, the estimated growth rate for total health spending under the better future scenario was 4·2% per year (3·8–4·5) through to 2040; however, even within this group of better scenarios, growth rates across countries ranged from 2·1% (0·4–4·5) in Liberia to 6·3% (5·0–7·8) in China. Growth rates for individual countries are available online. By 2040, the difference between the reference and better future scenarios for total health spending per capita was $1386 (1279–1477), with the better scenario being 59·9% (55·2–63·7) higher than the reference in 2040. Relative to the reference scenario, sub-Saharan Africa had the largest potential percentage gains under the better scenario by 2040. Furthermore, the better future scenario showed growth potential for pooled financing, with an annualised global growth rate of 4·0% (2·7–5·4). The better scenario would increase pooled health spending by 62·8% (60·3–69·0) compared with the reference by 2040. Across countries, the better future scenario for pooled resource growth ranged from 1·5% (−0·9 to 4·6) in Liberia to 6·7% (4·8 to 8·5) in China, reflecting important potential for gains.

For the worse health spending future scenario, the global growth rate was −0·3% (−0·7 to 0·0) through to 2040. The gap in total health spending per capita was $1085 (959 to 1234) between the reference and worse future scenarios, and spending was 46·8% (44·9 to 49·0) lower in the worse scenario than in the reference scenario in 2040. Under the worse scenario, pooled resources for health were projected to decrease at an annualised rate of 0·3% (−1·5 to 0·8).

**Figure 2:**
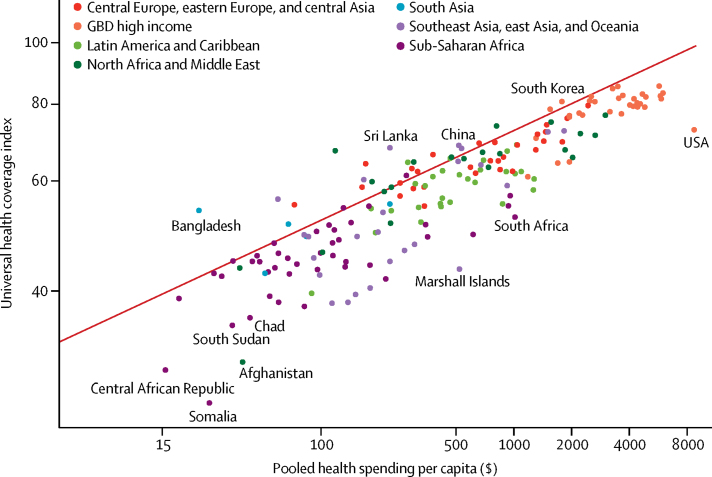
Universal health coverage financing frontier Pooled health spending per capita for 2015 is measured in 2017 purchasing-power parity-adjusted dollars adjusted for inflation. The red line represents the fitted frontier value of the universal health coverage index fitted using data from 1995 through 2015. Each dot represents a country colour-coded by Global Burden of Disease super-region. GBD=Global Burden of Disease.

Drawing from the empirical relationship between pooled resources per capita and the UHC index, we generated a UHC frontier, which highlights how financing can constrain progress in achieving UHC, as well as the gaps between potential and observed UHC (figure 2). The distance between observed performance on a country's UHC index and the frontier values estimated on the basis of the pooled resources per capita can reflect current challenges in translating national health resource into the maximum expected UHC index. The figure shows the large variations in the level of system inefficiency that hinders countries from achieving the optimal level of UHC. Despite this variation, the upward and significant (p<0·0001) slope of the UHC frontier shows a positive relationship between pooled resources for health and the UHC index: a 10% increase of pooled resources per capita was associated with a 1·4% (1·4–1·5) increase in the UHC index. Some countries, such as China and India, increased pooled spending per capita by more than 265% between 1995 and 2015 and had increases in UHC index of approximately 40%, suggesting that increases in spending can lead to substantial progress towards UHC.

**Figure 3:**
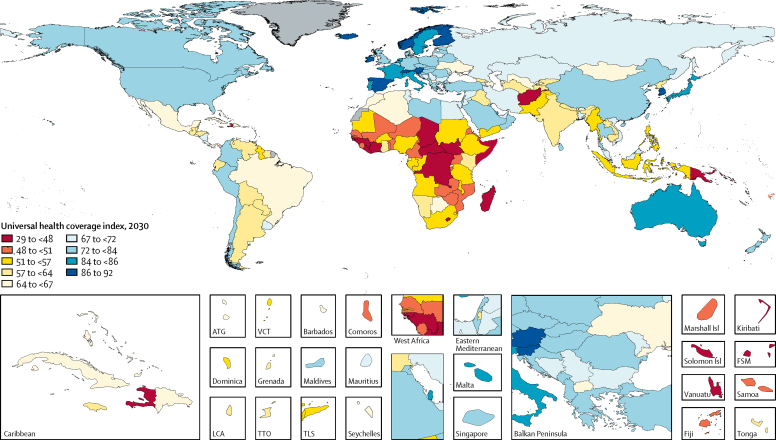
Universal health coverage in 2030 Projections are based on the reference scenario. Grey signifies countries without estimates. ATG=Antigua and Barbuda. VCT=Saint Vincent and the Grenadines. LCA=Saint Lucia. TTO=Trinidad and Tobago. Isl=Islands. FSM=Federated States of Micronesia. TLS=Timor-Leste.

Drawing from the UHC index frontier and the future scenarios for pooled health resource spending, we projected that, globally, the UHC index would increase to 64·8 (95% UI 61·8–67·0) in 2030, a 9·4% (4·6–14·2) rise from 2015 (figure 3), and to 67 (63–71) in 2040, a 13·7% (6·5–21·0) rise from 2015. Between 2015 and 2030 (the timespan for the SDGs), lower-middle-income countries saw the largest gains on the UHC index relative to their starting point, rising 15·6% (10·6–20·8) between 2015 and 2030. By contrast, high-income countries had the smallest increase, rising by 4·0% (0·9–6·7) between 2015 and 2030. During this same time, UHC index increased by 14·0% (7·0–21·7) in low-income countries and 10·4% (5·2–15·7) in upper-middle-income countries. In all four income groups, these gains are expected to continue through 2040. Sensitivity analyses reported in the appendix highlight that these results are robust to alternative modelling assumptions.

**Figure 4:**
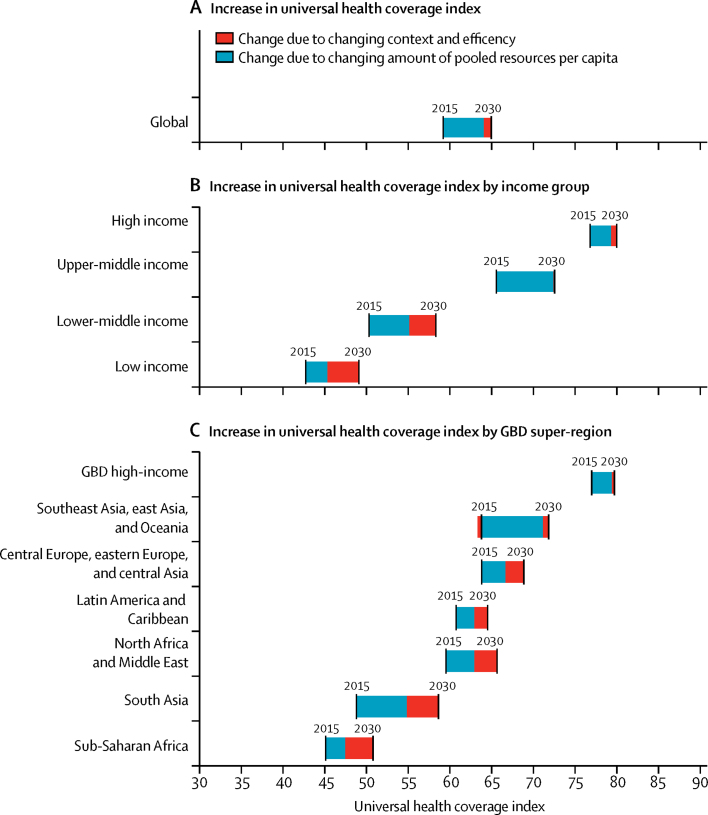
Increase in universal health coverage index from 2015 to 2030, by driver Income groups are based on 2017 World Bank income groups held constant across time. Universal health coverage index is the population weighted mean for each income group and Global Burden of Disease super-region. Black lines show the year-specific measure of the universal health coverage index. The bars connecting the black lines show the drivers of the universal health coverage index increases. Projections are based on the reference scenario. GBD=Global Burden of Disease.

Figure 4 shows the drivers of increases in UHC, decomposing the increase from 2015 to 2030 and from 2030 to 2040 into two distinct drivers—changes in the UHC index associated with changes in pooled health resources per capita and changes in the UHC index associated with changes in health system inefficiency and contextual factors. In most lower-middle-income, upper-middle-income, and high-income countries, increases in pooled spending per capita were predicted to be the primary drivers of the largest increases in UHC performance. Between 2015 and 2030, the proportion of the improvement in UHC index attributable to changes in pooled spending was 58·1% (40·9–68·7) in lower-middle-income countries, 98·0% (96·8–98·6) in upper-middle-income countries, and 74·2% (23·3–87·3) in high-income countries. Conversely, the proportion of improvement attributable to changes in system efficiency and contextual factors was 41·9% (31·3–59·1) in lower-middle-income countries, 2·0% (1·4–3·2) in upper-middle-income countries, and 25·8% (12·7–76·7) in high-income countries. For low-income countries and sub-Saharan Africa, we estimated that gains in efficiency and improvements in the contextual factors would lead to larger increases in UHC index (67·8% [43·5–97·0] in low-income countries and 66·7% [39·3–97·4] in sub-Saharan Africa, respectively). In the longer term (2030–40), however, projected increases in the amount of pooled resources per capita would have a larger impact in the progress towards UHC (55·7% [16·0–73·0] in low-income countries and 93·5% (90·9–95·4) in high-income countries).

**Figure 5:**
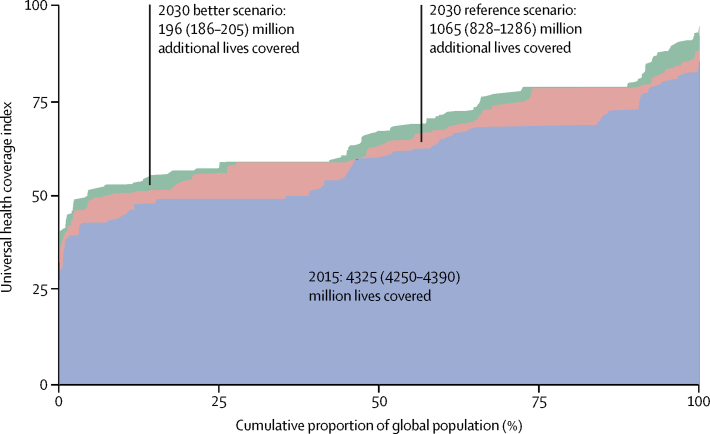
Lives covered, 2015, and under the 2030 reference and 2030 better scenarios On the horizontal axis, the global population is ordered by each country's UHC index rank. Similar to a concentration curve, each shift in the graph represents a country, with longer lines representing more populous countries. The area under the curve represents the share of the global population covered by UHC services. UHC=universal health coverage.

Figure 5 shows the change in the number of lives covered globally between 2015 and under the reference and better scenarios in 2030, as well as the distribution of the UHC index across countries. In 2015, we estimated that 4·3 billion (95% UI 4·2 billion to 4·4 billion) lives were covered under UHC, with the UHC index ranging from 26·5 (23·8–29·6) in Somalia to 85·3 (81·8–88·5) in Switzerland (table 2) across countries and half of the global population living under health systems with a UHC index less than 60. On the basis of the projected progress on the UHC index between 2015 and 2030, we estimated that an additional 1·1 billion (0·8 billion to 1·3 billion) people would be covered in 2030 under the reference scenario, and a further 196 million (186 million to 205 million) lives would be covered under the better scenario. The most pronounced projected gains in lives covered between 2015 and 2030 (shifts upward on figure 5) were for low-income countries (205·6 million [176·2 million to 236·6 million]; increase of 75·2% [64·4–87·0]), sub-Saharan Africa (303·9 million [252·7 million to 357·2 million], increase of 70·6% [58·9–83·6]), and South Asia (274·2 million [237·0 million to 304·5 million], increase of 33·5% [29·0–38·2]; table 2). Across income groups, there was a strong relationship between national income and the proportion of the population covered. By 2030, UHC was projected to cover 1·0 billion (0·9 billion to 1·0 billion) people in high-income countries (79·9% [77·3–81·8] of the population), whereas coverage was projected to be 72·4% (68·9–75·4) in upper-middle-income countries, 58·2% (55·3–60·2) in lower-middle-income countries, and 48·7% (45·6–51·9) in low-income countries. In addition, our analysis shows a projected difference of 870·5 million (790·4 million to 940·8 million) people being covered between the better and worse scenarios, stressing the need to ensure sufficient health financing for UHC in the SDG era.

## Discussion

Our projections highlight the large differences in expected future health spending and pooled health spending per capita across the globe, with high-income countries projected to spend 45·9 times (95% UI 37·1–54·6) more on total health expenditure per capita than low-income countries in 2040. Moreover, our reference scenario suggests relatively poor growth in pooled health spending for some countries that are already spending very little on health, including Angola, Benin, Chad, Guinea-Bissau, Nigeria, and Congo (Brazzaville). Our analysis, which quantifies the relationship between pooled resources for health per capita and potential performance in terms of UHC service coverage, showed a strong relationship between increased pooled resources and improved UHC performance. The gap between observed and potential UHC index performance given a country's pooled resources per capita varied considerably across countries, showing opportunity to better leverage these resources for UHC gains. Better and worse scenarios for health spending shed light on how trajectories in total health spending and pooled resources could enable accelerated gains in UHC or constrain progress if advances in health resources for UHC are not realised.

There is increasing global consensus on UHC and its ability to improve population health outcomes in an equitable, sustainable manner. Yet far less agreement exists in terms of how to translate UHC ambitions into reality, both in terms of universal service coverage and financial risk protection for all populations. Most recommended strategies focus on particular aspects of reaching UHC, such as strengthening human resources for health, improving the quality or efficiency of care provided, and updating the package of health services covered by insurance schemes.^32^, ^33^ However, fundamental to the success of these strategies, as well as overall UHC programme implementation and long-term sustainability, are sufficient and stable health financing systems. Understanding the levels, trends, and future scenarios for country-level health financing—and how these trajectories relate to potential advances in UHC—is crucial to informing policy and investment decisions regarding UHC.

Globally, we projected that the largest growth in total health spending would be in government spending from 2015 to 2040, although growth rates were highest for out-of-pocket health spending. Notably, the magnitude of this growth varied by and within income groups and GBD super-regions. We found that countries with substantial projected growth in pooled spending also had large projected gains in performance on the UHC index, which aligns with previous research on how pooled resources for health are positively related to UHC. By contrast, reliance on out-of-pocket spending, which was also estimated to grow, has been shown to deter care and lead to catastrophic health spending or impoverishment.^17^, ^34^

While not causal, the strong, positive relationship between pooled spending per capita and UHC index aligns with previous work that has described the relationships between higher public spending on improved service coverage and health outcomes.^15^, ^35^, ^36^ Economic theory further posits that the pooling of financial resources spreads financial risk across the population, and because individuals are protected from carrying the full burden of paying for their own health services, they are more likely to access and receive care.^37^ Globally, we found that along the frontier, a 10% increase in pooled health spending per capita from 2015 to 2030 was associated with a 1·4% (1·4–1·5) increase in performance on the UHC index. While this observation is encouraging, many countries remain some distance from the frontier, suggesting that gains in UHC could also be made by improving health system efficiency. Some countries, including Sri Lanka and South Korea, had projections exceeding this pace for both pooled heath spending and UHC performance, findings supported by past work documenting these countries' exceptional progress on UHC relative to their development status. It is important to recognise that increasing health spending is neither a necessary nor sufficient condition for improving UHC; the USA provides a counterexample, wherein very high health spending has not translated into high access to quality care for all populations. Rather, ensuring a supply of additional pooled resources for UHC, alongside other important sociopolitical factors and policy levers, is likely to provide a strong foundation for equity-focused, sustainable UHC programmes.

The intersection of pooled health spending and financial risk protection—the other key component of achieving UHC in the SDG area—has important programmatic and policy ramifications, particularly in terms of sustainably funding UHC initiatives. Protection against catastrophic health spending is generally offered through nationally funded insurance schemes or programmes that involve a mixture of privately and publicly funded insurance.^38^ Subsequently, the present study's quantification of the relationships between health spending and UHC service coverage performance might represent an overall underestimate of the pooled funds needed to achieve UHC more broadly. This might be particularly relevant in countries where the composition of total health spending has been historically more heavily skewed toward out-of-pocket or prepaid private health spending at the population level. Although valuable improvements in the measurement of catastrophic health spending have been made, no full time series currently exists for household catastrophic health spending across countries.^39^, ^40^ This data scarcity poses substantial challenges for the evaluation of potential shifts in response to policy implementation or the effects of pooled resources for health, although valuable improvements in the measurement of catastrophic health spending have been made. A critical area of future work entails not only generating estimates of financial risk protection for all countries and over time, but also evaluating the relationships between pooled health spending and the distribution of catastrophic health spending.

Generating future scenarios for pooled resources and potential UHC performance through 2040 has many applications, particularly in terms of budgetary planning and consideration of the impact of different investments. Our selection of three scenarios—reference, better, and worse—offers empirically derived guidance on what the world could achieve within realistic, albeit ambitious, parameters. Our better and worse scenarios were set to reflect the realistic extremes achieved historically by some countries, rather than hypothetical rates of growth or other arbitrary thresholds. We ground these scenario-based projections in what has been possible in the past, therefore offering a platform on which bold, and pragmatic, policy options can be evaluated.

This research also helps highlight the role health system efficiency can play in helping countries to progress towards UHC. We found that, in many countries, improvements in system efficiency could lead to similar or even larger UHC gains than solely increasing the amount of pooled resources. Although we found large variations in efficiency levels across countries, we cannot establish the causes of inefficiency, which might include corruption, low health worker productivity, excess administrative costs, spending on unnecessary care, or high prices, among others. Future work should consider how to quantify the drivers of system inefficiencies, as well as options for improving efficiency without jeopardising the quality of UHC.

More broadly, pathways to achieving UHC are complex and can come in various forms. Whether a country is successful in mobilising additional resources for UHC or improving system efficiency depends on not only technical knowledge, but also long-term, pragmatic political strategies that account for local political and historical legacies.^41^ How the benefits of UHC are distributed across different subgroups in a country needs to be actively discussed to ensure equity. Vulnerable populations, such as the poor, informal sector workers, and those living in rural areas, might be left without access to health coverage unless deliberate efforts are made to reach them. The allocation of the national health budgets towards UHC also needs to be balanced with spending on other crucial health areas, including health emergency preparedness, health promotion, and capital investments such as hospitals.^42^ Furthermore, in the absence of good governance and leadership at the national and local levels, any efforts towards and gains in UHC could be in vain. Such factors, as well as forces that primarily exist outside of the health sector (ie, physical infrastructure, education systems), have the potential to substantially accelerate or constrain a country's progress toward UHC.

Estimating levels of future spending and how spending might affect UHC is intrinsically uncertain, given that spending patterns and health financing systems evolve over time and are shaped by many factors. These include national and international policymaking, shifts in the supply and demand of health services, economic development, changes in political regimes and policy directions, and economic, social, political, and abrupt events such as civil strife, natural disasters, and emergent epidemics. In the absence of systematic and credible projections of all of these factors, we rely on past global and country-specific spending trends and the relationships between these variables and underlying economic development, government spending, and demographic measures. To capture the uncertainty of these relationships in our future scenarios, we have sought to propagate uncertainty from a range of sources, including data, model, and parameter uncertainty. As advances are made to quantify projections of a wider range of factors related to UHC, we aim to incorporate them into our models and increasingly narrow our estimates of uncertainty.

Our study has some limitations. First, precise data on different types of health financing were not readily available across countries and over time. This is particularly challenging for out-of-pocket and prepaid private spending, given that many countries do not have robust information systems on such data and or because informal payments constitute a large share of health spending. For this study, we relied on data from the Institute for Health Metrics and Evaluation's Financing Global Health database, estimates a complete dataset across time, and quantifies uncertainty.^25^ Second, projected levels and types of health spending in 2040 were based on relationships observed from 1995 to 2015. These projections reflect trends in country development and demographic changes, but cannot capture the full range of unobserved historical policy or environmental changes. Furthermore, these projections do not account for unknown future impacts of changes in international and national policy, macroeconomic development, new technologies, emergent epidemics, and natural disasters. Third, alternative frontier methods, such as data envelopment analysis, could have been used instead of SFA. However, our sensitivity analyses (appendix) showed that our overall results were robust to the choice of method. Fourth, the positive association between the share of pooled resources and UHC performance does not represent causality. More research is needed to explore the other possible mechanisms driving this relationship. Fifth, our measure of UHC focused on performance in terms of service coverage and health outcomes rather than both coverage and financial risk protection. Although we expect that protection from catastrophic health expenditure is well correlated with both pooled resources per capita and our measure of UHC, our current results do not capture the effects of financial risk protection on UHC performance and how pooled resources might support this aspect of UHC. Similarly, the current measure of UHC approximates personal health-care access and quality, but our study cannot estimate the direct effect of pooled resources on quality of care. Future work to establish a cohesive metric of overall UHC, a measure of service coverage, quality of care, and financial risk protection, will support improved assessments of the funding required to reach and maintain high-quality UHC for each country over time.^43^ Sixth, the DAH dataset currently only includes financial flows from high-income countries to lower income countries. It does not capture the growing contributions and development assistance made by low-income and middle-income countries. Finally, we posit that, although more health spending per capita is better for the health system, there are many examples showing that greater spending does not always lead to better health outcomes. Equitable spending in allied sectors such as education and social protection has also been shown to affect health but was not considered in this study.^44^

Achieving UHC has great potential for improving population health outcomes and narrowing health inequalities. Ensuring that all countries across the development spectrum have a stable and sufficient supply of pooled resources for health is likely to help bridge current gaps in UHC performance. Our future health spending scenarios show the potential for spending to catalyse—or constrain—progress in UHC performance, particularly given the relationship between pooled resources per capita and levels achieved on the UHC index. These results are crucial for long-term resource planning and to address financing gaps: vital steps along the path to bringing UHC within reach of all populations.

Correspondence to: Dr Joseph L Dieleman, Institute for Health Metrics and Evaluation, Seattle, WA 98121, USA **dieleman@uw.edu**

**This online publication has been corrected. The corrected version first appeared at thelancet.com on May 3, 2018**
