# Department of Error

**DOI:** 10.1016/S0140-6736(18)30985-1

**Published:** 2018-05-05

*Global Burden of Disease Health Financing Collaborator Network. Trends in future health financing and coverage: future health spending and universal health coverage in 188 countries, 2016–40.* Lancet *2018; **391:** 1783–98—*In table 2 of this Article, a digit was mistakenly omitted from after the decimal point in the value for universal health coverage index in high-income countries in 2030 under the worse scenario. This correction has been made to the online version as of May 3, 2018, and the printed version is correct.

