# Supplementary material for: Trends in future health financing and coverage: future health spending and universal health coverage in 188 countries, 2016–40
Source: Lancet. 2018 May 5;391(10132):1783–98. doi: 10.1016/S0140-6736(18)30697-4 (PMC5946843; doi:10.1016/S0140-6736(18)30697-4)
Supplement: Supplementary appendix [file mmc1.pdf]

THE LANCET

Supplementary appendix

This appendix formed part of the original submission and has been peer reviewed. We post it as supplied by the authors.

Supplement to: Global Burden of Disease Health Financing Collaborator Network. Trends in future health financing and coverage: future health spending and universal health coverage in 188 countries, 2016–40. *Lancet* 2018; published online April 17. http://dx.doi.org/10.1016/S0140-6736(18)30697-4.

Estimating future health spending and universal health coverage, 2016 - 2040

Supplementary Appendix

Global Burden of Disease Financing Global Health Collaborator Network

Updated: Friday 13th April, 2018

Contents

1 Data **4**

1.1 Summary of Data Sources 4

1.2 Institute for Health Metrics and Evaluation's Development Assistance for Health Database 4

1.3 World Health Organization's Global Health Observatory 5

1.4 United Nations World Population Prospects 2017 6

1.5 WB, IMF, PWT, and Maddison 6

2 Covariates **8**

2.1 List of Forecasted Variables 8

2.2 Covariates Used for Forecasting 8

3 Ensemble Modeling **10**

3.1 Sub-model Setup 10

3.2 Covariates 10

3.3 Specifications 11

4 Package and Architecture **12**

4.1 Architecture 12

4.2 R-INLA 12

5 Inclusion and Exclusion Criteria **13**

6 Creating the Forecasts **14**

6.1 Ranking Sub-Models 14

6.2 Uncertainty Estimation 14

6.3 Sensitivity Analysis 15

7 Ad-hoc Draws Correlation **17**

7.1 Motivation 17

7.2 Bivariate Correlated Distributions 17

8 Future Health Scenarios **19**

8.1 Long-term Growth Regressions 19

8.2 Forecasting Better and Worse Scenarios 19

8.3 Uncertainty Estimation 20

9 Universal Health Coverage Index **21**
9.1 Definition 21
9.2 Forecasting UHC 22
9.2.1 Stochastic Frontier Analysis 22
9.2.2 Forecasting Steps 22
9.2.3 Decomposition of Forecasting Components 23
9.3 Comparison of Forecasts 24

A Tables and Figures **25**
A.1 State Space Diagram of Forecasting Components 26
A.2 Prediction and Covariates Map with Transformations 27
A.3 Comparison of Forecasts 28
A.4 Sensitivity Analyses on UN WPP Variants 34

B Additional Tables and Figures **41**
B.1 Table: Future Health Spending in 2015 and 2030 41
B.2 Table: Future Health Spending in 2015 and 2040 53
B.3 Table: Future Health Scenarios in 2030 65
B.4 Table: Future Health Scenarios in 2040 76
B.5 Figure: Comparison of Future Health Scenarios 88

Introduction

The objective of this study is to provide data on future health spending patterns that can guide decision-makers. These analyses produced comprehensive and comparable set of gross domestic product and all-sector government spending estimates, followed by all the components of a country's total health expenditure. Additionally, using observed past trends, we predicted the possible trajectories of the covariates of interest conditional on following an optimistic and pessimistic pattern based on global rates of change.

The purpose of this appendix is to describe in detail the methodology used in our analyses. Subsequent sections contain information on all data sources, ensemble forecasting strategies, inclusion criteria, and uncertainty estimation used to generate our estimates, as well as our guidelines on how our future health scenarios analyses were conducted.

Authors' Contributions

Managing the estimation process

Abigail Chapin and Joseph Dieleman

Writing the first draft of the manuscript

Angela Chang, Joseph Dieleman, and Nancy Fullman.

Providing data or critical feedback on data sources

Cristiana Abbafati, Tara Ballav Adhikari, Kouablan Arsene Adou, Aliasghar Ahmad Kiadaliri, Khurshid Alam, Lafranconi Alessandra, Ala'a Alkerwi, Walid Ammar, Olatunde Aremu, Solomon W. Asgedom, Ashish Awasthi, Rakesh Ayer, Maciej Banach, Amrit Banstola, Aleksandra Barac, Charles Birungi, Josip Car, FerrÃąn CatalÃą-LÃşpez, Lalit Dandona, Rakhi Dandona, Ahmad Daryani, Samath Dharmaratne, Manisha Dubey, Dumessa Edessa, Babak Eshrati, Andre Faro, Ruoyan Gai, Mamata Ghimire, Alemayehu Hailu, Hilda Harb, Delia Hendrie, Mehdi Javanbakht, Denny John, Jost B. Jonas, Amir Kasaeian, Yawukaal Kasahun, Ibrahim Khalil, Young-Ho Khang, Jagdish Khubchandani, Yun Jin Kim, Soewarta Kosen, Kristopher Krohn, G Anil Kumar, Muhammed Magdy Abd El Razek, Azeem Majeed, Reza Malekzadeh, Atte Meretoja, Ted Miller, Erkin Mirrakhimov, Shafiu Mohammed, Mohsen Naghavi, Frida Ngalesoni, Trang Nguyen Huyen, Cuong Nguyen Tat, Mehdi Noroozi, Mayowa Owolabi, David Pereira, Suzanne Polinder, Mostafa Qorvani, Anwar Rafay, Alireza Rafiei, Vafa Rahimi-Movaghar, Rajesh Kumar Rai, Chhabi Lal Ranabhat, Maheswar Satpathy, Sadaf Sepanlou, Masood Ali Shaikh, Mehdi Sharif, Aziz Sheikh, Mark Shrime, Mekon-

nen Sisay, Bach Tran, Khanh Bao Tran, Tung Tran Thanh, Eduardo Undurraga, Tommi Vasankari, Francesco S Violante, Andrea Werdecker, Tissa Wijeratne, Gelin Xu, Ebrahim M. Yimer, Naohiro Yonemoto, Mustafa Younis, and Chuanhua Yu.

Developing methods or computational machinery

Joseph Dieleman, Kyle Foreman, Robert Reiner, Nafis Sadat, and Tara Templin.

Applying analytical methods to produce estimates

Khurshid Alam, Solomon W. Asgedom, Charles Birungi, Catherine Chen, Ahmad Daryani, Maysaa El Sayed Zaki, Erika Eldrenkamp, Yawukaal Kasahun, Ibrahim Khalil, Yun Jin Kim, Hassan Magdy Abd El Razek, Muhammed Magdy Abd El Razek, Shafiu Mohammed, Frida Ngalesoni, Alireza Rafiei, Nafis Sadat, Maheswar Satpathy, Mehdi Sharif, Tianchan Tao, Tissa Wijeratne, and Ebrahim M. Yimer.

Providing critical feedback on methods or results

Cristiana Abbafati, Pawan Acharya, Tara Ballav Adhikari, Kouablan Arsene Adou, Aliasghar Ahmad Kiadaliri, Khurshid Alam, Reza Alizadeh-Navaei, Ala'a Alkerwi, Walid Ammar, Carl Abelardo Antonio, Olatunde Aremu, Solomon W. Asgedom, Tesfay Mehari Atey, Leticia Avila-Burgos, Ashish Awasthi, Hamid Badali, Amrit Banstola, Aleksandra Barac, Abate Bekele, Charles Birungi, Nicola Luigi Bragazzi, Nicholas Breitborde, Lucero Cahuana-Hurtado, Josip Car, Deborah Carvalho Malta, FerrÃąn CatalÃą-LÃşpez, Lalit Dandona, Rakhi Dandona, Ahmad Daryani, Samath Dharmaratne, Manisha Dubey, Dumessa Edessa, Maysaa El Sayed Zaki, Babak Eshrati, Andre Faro, Andrea Feigl, Ama Fenny, Florian Fischer, Nataliya Foigt, Kyle Foreman, Nancy Fullman, Ruoyan Gai, Srinivas Goli, Alemayehu Hailu, Samer Hamidi, Hilda Harb, Simon Hay, Delia Hendrie, Mehdi Javanbakht, Denny John, Jost B. Jonas, Amir Kasaeian, Yawukaal Kasahun, Ibrahim Khalil, Young-Ho Khang, Jagdish Khubchandani, Yun Jin Kim, Jonas Kinge, G Anil Kumar, Hilton Lam, Yirga Legesse, Stefan Listl, Hassan Magdy Abd El Razek, Muhammed Magdy Abd El Razek, Azeem Majeed, Reza Malekzadeh, Gabriel Martinez, George Mensah, Atte Meretoja, Ted Miller, Erkin Mirrakhimov, Shafiu Mohammed, Mohsen Naghavi, Vinay Nangia, Frida Ngalesoni, Trang Nguyen Huyen, Cuong Nguyen Tat, Mehdi Noroozi, Mayowa Owolabi, Tejas Patel, David Pereira, Suzanne Polinder, Mostafa Qorvani, Anwar Rafay, Alireza Rafiei, Vafa Rahimi-Movaghar, Rajesh Kumar Rai, Usha Ram, Chhabi Lal Ranabhat, Sarah Ray, Haniye S Sajadi, JoÃčo Vasco Santos, Abdur Razzaque Sarker, Benn Sartorius, Maheswar Satpathy, Sadaf Sepanlou, Masood Ali Shaikh, Mehdi Sharif, Aziz Sheikh, Mark Shrime, Mekonnen Sisay, Samir Soneji, Moslem Soofi, Reed Sorensen, Tara Templin, Azeb Tesema, Subash

Thapa, Roman Topor-Madry, Bach Tran, Khanh Bao Tran, Tung Tran Thanh, Eduardo Undurraga, Tommi Vasankari, Francesco S Violante, Fitsum Weldegebreal, Andrea Werdecker, Tissa Wijeratne, Gelin Xu, Ebrahim M. Yimer, Naohiro Yonemoto, Mustafa Younis, and Chuanhua Yu.

Drafting the work or revising is critically for important intellectual content
Khurshid Alam, Solomon W. Asgedom, Ashish Awasthi, Hamid Badali, Amrit Banstola, Angela Chang, Ahmad Daryani, Joseph Dieleman, Manisha Dubey, Maysaa El Sayed Zaki, Andre Faro, Florian Fischer, Nataliya Foigt, Nancy Fullman, Yawukaal Kasahun, Jagdish Khubchandani, Yun Jin Kim, Kristopher Krohn, Hilton Lam, Hassan Magdy Abd El Razek, Muhammed Magdy Abd El Razek, Azeem Majeed, Seyyed Meysam Mousavi, Christopher Murray, Frida Ngalesoni, Mayowa Owolabi, Anwar Rafay, Alireza Rafiei, Vafa Rahimi-Movaghar, Rocco Santoro, Abdur Razzaque Sarker, Maheswar Satpathy, Mehdi Sharif, Bach Tran, and Ebrahim M. Yimer.

Extracting, cleaning, or cataloging data; designing or coding figures and tables
Catherine Chen, Erika Eldrenkamp, Nafis Sadat, and Tianchan Tao.

Managing the overall research enterprise
Abigail Chapin, Joseph Dieleman, and Christopher Murray.

1 Data

1.1 Summary of Data Sources

We used data from seven sources for the analyses:

- World Health Organization's (WHO) Global Health Observatory
- Institute for Health Metrics and EvaluationâĂŹs Development Assistance for Health Database (IHME)
- International Monetary Fund's (IMF) World Economic Outlook (WEO) database
- Penn World Tables 9.0 (PWT)
- World Bank (WB) World Development Indicators database (WDI)
- Angus Maddison Project
- United Nations (UN) World Population Prospects (WPP)

Specifically, we collected health expenditure information on all available sources that is comparable across countries and complete for most countries from WHO and IHME, and demographic data from the WPP, while the underlying data for producing gross domestic product (GDP) and general government expenditure (GGE) were extracted from the IMF, WB, and PWT. Table 1 presents the definitions for the various health expenditure sources.

1.2 Institute for Health Metrics and Evaluation's Development Assistance for Health Database

Development assistance for health estimates were obtained from the Institute for Health Metrics and Evaluation's Development Assistance for Health Database. To generate these estimates, IHME collected audited budgets, annual reports, and project records from the primary development agencies providing assistance for the health sector. These records are augmented by information acquired via correspondence, and are standardized and compiled to provide a comprehensive perspective on international financial flows for health. These estimates are tracked backward to the source of the funds and forward to the country recipient, and are available from 1990 through 2017 and 2016, respectively.

1.3 World Health Organization's Global Health Observatory

We used Global Health Expenditure Database (GHED) data from the WHO to generate our estimates, which spans for 188 countries between 2000 through 2015. From the GHED, we pulled the following variables:

(i) Transfers from government domestic revenue (allocated to health purposes)

(ii) Social insurance contributions

(iii) Compulsory prepayment

(iv) Voluntary prepayment

(v) Other revenues from corporations

(vi) Other revenues from non-profit institutions serving households (NPISH)

(vii) Gross Domestic Product

(viii) Household out-of-pocket payment

(ix) Government schemes and compulsory contributory health care financing schemes

To ensure we were using the best possible data, we downloaded the metadata for each data point for all of the indicators from the GHED website. We used the metadata to decide how each given data point should be weighted, from 1 to 5, being applied as inverse variance weights. We established guidelines for the metadata that informed how the underlying data points should be weighted, giving priority to factors such as complete, documented source information and penalizing factors such as having been derived or estimated. We adjusted these data by converting them from current local currency to 2017 purchasing-power-parity (PPP$). Details of the weighting guidelines and classification are explained in the supplementary appendix our retrospective analysis paper [1].

Once we have an incomplete set of data points for the health expenditure variables we are interested in forecasting, we used Spatiotemporal Gaussian process regression (ST-GPR) to model the full time-series for each variable across 188 countries. ST-GPR is a stochastic modeling technique that is designed to detect signals amidst noisy data. Unlike classical linear models that assume that the trend underlying data follows a definitive functional form, GPR assumes that the specific trend of interest follows a Gaussian Process, existing with some pointwise mean and covariance function. [2]. The covariates that were used in order to determine the initial fit of our health expenditure variables are: lag-distributed

income, all-sector government expenditure per capita, healthcare access and quality index [3], and proportion of total population over the age of 64; using a Matérn covariance matrix for the distribution of the Gaussian process. Once we determined an initial prediction of our dependent variables, using the variability of data across regions, Gaussian process regressions (GPR) were run in order to estimate 1,000 draws of each country-year estimate per metric.

1.4 United Nations World Population Prospects 2017

The United Nations (UN) World Population Prospects (WPP) provides population estimates and forecasts by age, sex, country, and year from 1950 until 2100. Using a cohort-component approach, WPP utilizes life tables to generate forecasts of age-specific mortality rates. Their modeling strategy involves a hierarchical Bayesian model (with an AR(1) process) of female life expectancy (with male life expectancy being highly correlated with female's) that prioritizes country data if available, but otherwise draws on regional data [4, 5]. A separate step models the male-female difference in life expectancy. From their final data source, we generate multiple indicators of demographic context, such as the total fertility rate (TFR), the proportion of the population under the age of 20, the proportion aged 65 years and over, and the total population for each country and year. We used the medium variant of the dataset produced by the WPP, while the high and low variants were used for sensitivity analyses in chapter 6.3.

For a certain set of countries where either age-sex specific population or the TFR data were missing, we used a combination of a secondary data source (the US Census Bureau [6], which spanned from 2000 through 2050) and the global burden of disease (GBD) region specific rate of change to fill in and extrapolate, therefore giving us complete country-year time series for TFR and age-sex specific population spanning from 1950 through 2099 (2099 being the final year of TFR data available for other countries with existing data points).

1.5 WB, IMF, PWT, and Maddison

The WDI Database provides data on a wide range of development related variables, including data on GDP and GDP per capita. Data series in this database begin in 1960. The IMF's WEO Database provides data on various macroeconomic indicators. Macroeconomic series data are available from 1980 to present. The PWT is a database that provides real national accounts data for 167 countries and territories. The data series starts in 1950. The Maddison Project database provides historical GDP, GDP per capita, and population data dating as far back as Roman times. We utilized GDP per capita as a primary covariate to produce forecasts. GDP per capita from 1950 through 2016 was constructed using the

method described in *James et al* [7] . The method utilized extracted data from a number of sources (IMF, WB, PWT and Maddison), and used multiple random effects models to estimate a mean GDP per capita series to be used in our analysis. Similarly, we used the same methodology to produce a mean general government expenditure (GGE) per GDP series, from 1980 through 2016.

2 Covariates

2.1 List of Forecasted Variables

The following are the list of variables which are forecasted in the manuscript:

- GDP: Gross Domestic Product (national income of a country)
- GGE: General Government Expenditure (all sector government expenditure in a country)
- DAH_d: Development Assistance for Health donated
- DAH_r: Development Assistance for Health received
- GHE: Government Health Expenditure
- OOP: Out-of-pocket Private Expenditure
- PPP: Prepaid Private Expenditure
- THE: Total Health Expenditure
- UHC: Universal Health Coverage Index

2.2 Covariates Used for Forecasting

The following covariates are used as predictors, or independent variables, in our models:

- Total population of a country (1950 - 2100)
- Proportion of total population below the age of 15 (1950 - 2100)
- Proportion of total population above the age of 64 (1950 - 2100)
- Total Fertility Rate (1950 - 2099)
- Education attainment per capita [8] (1980 - 2040)
- An indicator variable used to denote the anomalous disbursement of DAH in our DAH_d forecasts (1 for years in 2000 - 2010, 0 otherwise)
- An indicator variable used to denote the Ebola crisis in Guinea, Sierra Leone and Liberia in our DAH_r forecasts.

Additionally, we also use the forecasted GDP and GGE per capita as covariates to predict the health expenditure variables. Table 2 contains the summary statistics of all the covariates predicted and used in our modeling, between 1995 through 2015. Section A.1 in the Tables and Figures section shows our full pathway for forecasting all our endogenous variables and how each of those variable feed into a succeeding model.

3 Ensemble Modeling

The purpose of ensemble modeling is to make sure that we capture the most out of what we have in our arsenal in terms of covariates and model specifications. We are agnostic about one model being the sole predictor of the future, and allow an ensemble of beliefs about predicting off of the past trends. 'Ensembling', in simple terms, is a way of pooling a number of sub-models, where the space of sub-models span different inclusions and combinations of predictors, and/or different econometric specifications.

3.1 Sub-model Setup

Our basic sub-model is a linear mixed effect model of the following form, for country i and time t:

$$Y_{i,t} = (\alpha + \alpha_i) + \left(\sum_{p=1}^{3} \rho_p Y_{i,t-p} \right) + X'\beta + \varepsilon_{i,t} \tag{1}$$

where

$$\left(\alpha, \vec{\beta}\right) \sim N\left(0, \frac{1}{0.01^2}\right) \quad \text{(Fixed Effects with 0.01 precision prior)} \tag{2}$$

$$\alpha_i \sim N(0, \sigma_a^2) \quad \text{(Country specific random intercept)} \tag{3}$$

$$\varepsilon_{i,t} \sim N\left(\sum_{m=1}^{3} \phi_m \varepsilon_{i,t-m}, \sigma_i^2 \right) \quad \text{(Autocorrelated residuals as time random effects)} \tag{4}$$

'Fixed effect' in a Bayesian setting is essentially allowing the prior to move around freely so that it estimates a value closest to the frequentist approximation. It is allowed to do so by having a very small precision value (0.01) and therefore a large variance for the prior distribution, which effectively allows the posterior estimates to be based off of the data entirely. The likelihood function for the data were all set to be Gaussian distributions for all of our metrics and models.

3.2 Covariates

Using our linear baseline model as defined in section 3.1, we created our set of ensemble sub-models by using all combinations of each of the covariates in Table A.2. For example, if we were predicting GDP per capita with TFR and Population as the predictors, then we would get a possible combination of four specifications to use (including a no-covariate one). Covariates were included as fixed effects.

3.3 Specifications

1) **ARIMA (Autoregressive Integrated Moving Average) terms**: We allowed up to three degrees of lags in the model (traditional auto-regressive terms), where each degree of AR term will include itself and all other lower degrees of lags. For example, a GDP per capita model with AR(3) specification (and using log of GDP per capita) will include once, twice and thrice lagged log GDP per capita term. These were included as fixed effects.

   Additionally, in order to predict the best set of fixed effect coefficients, we test and include auto-correlated residuals in our models (traditional moving-average terms in an ARIMA setup). This basically means that we allow our models to estimate the residuals with an autoregressive process of their own. These were included as random effects, and we allowed this to exist at the country-year level.

2) **Upweighting of Recent Years**: One of the other specifications we included in our sub-models was the option of weighting the recent years higher. This is particularly helpful for countries like Ethiopia and Nigeria, where they had rapid economic growth in recent years, and we believe that is a better predictor of the data than the further past. We generated a column of weights as such:

$$Weight = \frac{100}{(T + 1 - t)^{\tau}} \tag{5}$$

   where T is the final year of in-sample data we have, and t is the year at that data point. This is an exponential decaying weighting function, where the degree of decaying is determined by τ, and we test and include a set of values of $\tau : \{0, 0.25, 0.5, 0.6, 1\}$, where $\tau = 0$ refers to equal weights (all time periods are weighed equally at 100). The weighting functions enter our model through the likelihood function by simply multiplying each of the data points with the associated weight values.

3) **Convergence Term**: We also allowed for the inclusion of a 'convergence term' in the list of sub-models. A convergence term is the one-year lag of the non-differenced dependent variable, and gets updated as each year is forecasted in the future. If a convergence term was considered in a sub-model, then we only included that sub-model if the coefficient on the convergence term was estimated to be negative (and statistically significant at 10% level).

4 Package and Architecture

4.1 Architecture

All analysis and forecasting were done on a parallel computing cluster with 20,000 nodes with a CentOS interface. We compiled R[9] version 3.4.3 from source code on a Docker based on Debian OS, which was deployed as a Singularity container with all the necessary compilers and binaries (GCC, G++ and Fortran 7.2.0).

4.2 R-INLA

We used the library **R-INLA**[10, 11, 12] to run our baseline mixed effects model. **INLA** stands for Integrated Nested Laplace Approximation, and it's a powerful method of approximating the integral of the Gaussian probability distribution function (which doesn't exist in closed form) by using a Laplace Approximation[13, 14], which gives very precise results, and is relatively faster than other approximation packages which exist like Stan or Bugs. Using the Newton-Raphson optimization algorithm instead of slower methods like Markov Chain Monte Carlo, **INLA** is powerful and flexible and allows us to specify our own priors, and automatically simulates posterior draws, which is very helpful and therefore lets us create parameter draws much faster without needing the help of any other external libraries.

5 Inclusion and Exclusion Criteria

After we ran all possible combination of our sub-models and created a mean set of forecasts, we only want to keep the best possible set of sub-models. Hence, we implied the following set of inclusion and exclusion criteria in order to filter out the 'unrealistic' sub-models:

(1) All of the estimated coefficients must be **statistically significant at 10% level of significance**. For the fixed effects, we took the mean and the standard deviation of the posterior estimates, and filtered out the sub-model if the absolute z-score is below 1.645 (the absolute value of the one-sided 95th quantile of a standard normal distribution).

For the random effects, we look at the whether the measure of variance is statistically significant or not. The model outputs the mean and standard deviation of the *precision of the random effects*, and therefore allowed us to exclude the specification if the precisions are not statistically significant at 10% level.

(2) If there were any estimated coefficient that **defied a prior belief we have on the direction of the value**, then we dropped that sub-model from consideration. For example, we strongly believe that as a donor country's (high income countries) income (GDP per capita) grows, they will be able to donate more DAH to lower income countries, and so, if we ran a sub-model predicting DAH_d and get a negative coefficient on GDP per capita, we dropped that sub-model from consideration.

Our prior beliefs on the covariates for each dependent variable are listed in Table A.2.

(3) The forecasted trajectory growth **must not exceed observed growth**. We believe that a country will not grow faster than how much it has grown in the past trend. In order to come up with the bounds, we run a stochastic frontier analysis (SFA) of the change in the predicted variable against the level value of the predicted variable. SFA is just like an ordinary least squares specification, except with the addition of an additional 'inefficiency' term with a half-normal distribution. This allows us to estimate (for example, for GDP per capita): conditional of a country's income, how much growth rate did we see in the country's GDP. We ran this analysis across all of the observed data points, and derived a relationship binding the growth rates of GDP against the absolute values of GDP. More details on the implementation of SFA are explained in section 9.2.1.

6 Creating the Forecasts

6.1 Ranking Sub-Models

In order to find out with sub-models would be able to predict a country's future the best, we ran out-of-sample predictive validity (OOS-PV) tests[15]. Simply put: we took each sub-model that passed the criteria in section 5, and instead of running it on all of the past data, we left out some number of recent most years. Then we ran the sub-model on the truncated past, and forecasted those years left out. For example, our GDP data extends from 1970 through 2017; we left out 10 years of data, and reran a sub-model from 1970 - 2007, and use the results of that sub-model to forecast GDP for the out-of-sample years (2007 - 2016).

This gave us essentially two trajectories between 2007 through 2016: the truth and the out-of-sample predictions. For each year, we computed the squared error (the difference) between these two lines, and averaged these errors for each of the neighboring years. So for example, we had squared errors for 10 data points between 2007 and 2017, and so the first mean squared error sum was just the squared error at 2007 and 2008, the second was the mean of the sum of squared errors for 2007, 2008 and 2009, and so on. We then took the square root of this new series to get the running root mean squared errors (RMSE) for a given country.

We looked at a country's 2007 RMSE values for each sub-models ran, and listed out the best 10% of the sub-models (that is, the lowest 10% RMSE values), and we did so for every year out-of-sample. For a single country, we may potentially have completely different set of sub-models for each of the OOS years. Then, for the 10% of the sub-models selected in the *first* year OOS, we only used those models to predict the *first* year of forecast for each country; the set of 10% of the sub-models selected in the *second* year OOS were used to predict the *second* year of forecast for each country, and so on, until the last year OOS model selections are used to compute the forecast the remaining years. This allowed us to narrow down every country's trajectory with the best performing OOS-PV sub-models for each year.

6.2 Uncertainty Estimation

To estimate the uncertainty intervals (UI), we reran the selected, ranked sub-models from section 6.1 and simulated draws instead of just getting a mean estimate of the future. There are four types of uncertainty we implemented in forecasting:

(i) **Model Uncertainty** : This type of uncertainty comes from having more than one type of specification to create forecasts, and therefore we included a set of sub-models

in the ensemble to incorporate for this uncertainty (which are ranked within each country-year).

(ii) **Data Uncertainty** : If our covariates themselves had draws of the future data (for example, when we forecasted GDP as a covariate to forecast PPP), then we picked randomly from the draws of the independent variable when predicting a sub-model's trajectory, if that covariate was included.

(iii) **Parameter Uncertainty** : This type of uncertainty is due to the variance for the posterior distributions. Once we have run a sub-model, we simulated from each of the estimated posterior distribution to create a set of simulated coefficients. **INLA** does so by using a Gaussian copula distribution across the parameters being estimated, and therefore produces a correlated set of coefficients.

(iv) **Fundamental Uncertainty** : The in-sample data and the fitted line in the past will never line up perfectly: there will always be errors from the model fit. We needed to reflect this level of uncertainty in our forecasts as well. By extracting these empirical residuals produced by a sub-model, we forecasted future country-specific residuals by using a random walk process, where the variance of the process is the variance of the residuals from the model fit σ^2_ε:

$$\hat{\varepsilon}_{i,t} \sim N(\varepsilon_{i,\hat{t}-1}, \sigma^2_{\varepsilon,t}) \tag{6}$$

A random walk is an AR(1) process with the coefficient equal to 1: in other words, the current value is independent of last year's value (except for the starting position), and will propagate forward with a random Gaussian noise of $\sigma^2_{\varepsilon,t}$ variance.

All of the above were used to simulate 1,000 forecasts (or draws), and so in order to construct our UIs, we took the mean and the values of the 2.5th and 97.5th quantiles to estimate 95% UIs. The complete set of health expenditure forecasts (per capita) for all 188 countries in our analyses have been included as Figure B.5 at the end of this appendix.

6.3 Sensitivity Analysis

Given that each of the forecasted variable drew from a large pool of sub-models and uncertainty methods, sensitivity analyses related to the ensemble architecture would not be as informative. Therefore, our main method of sensitivity analysis was conducted using changes in covariate data.

The main source of demographic variables (population fractions and fertility) came from the UN WPP database. While all of the forecasts were generated using the medium variant of the UN data, we used the high and low variants of the TFR and population data. Those two variants had the most diverging growth rates out of the nine variants produced by UN WPP (the mean of the annualized growth rates of population across all of our countries for the high and low variants were 28.1% and 14.9% respectively).

We conducted the sensitivity analyses on GDP per capita forecasts, since GDP per capita uses all of the demographic variables we considered in our ensemble, and it fed into all the succeeding predicted variables in our pipeline. Figures 8 and 10 show the scatterplot of 2040 GDP per capita values for each country using the High and Low variants (respectively) against our baseline reference forecasts (which used the medium variant). Our forecasts based on the medium variant are very robust and barely changed when we reran the ensemble using the different variants, and this was true for almost all years of the forecasts; figures 7 and 9 show the scatterplot of 2040 GDP per capita values for each country using the High and Low variants (respectively) against our baseline reference forecasts for all years between 2018 and 2040 inclusive.

7 Ad-hoc Draws Correlation

7.1 Motivation

Given our current setup of compiling draws for each single year, there is no way of enforcing a temporal correlation across the draws right from the ensemble architecture. For example, the sub-models used in the first 10 years of GDP per capita forecasts were independently constructed and only depended on OOS-PV fits, while the 10th year (2027) onwards all draw from the same set of sub-models. This section details on the method used to generate the same correlation in the first 10 years of that example, drawing from the existing correlation from the 10th year onwards.

7.2 Bivariate Correlated Distributions

Using the GDP per capita example: following from uncertainty estimation, once we generated approximately 1,000 forecast draws for a country and year for any of the covariates, we used the following strategy to achieve consistent temporal correlation across all time periods in the future:

(a) For each country, we recorded country-specific Spearman's correlation coefficient across all draws between 2027 and 2040, which gave us a country-specific correlation vector.

(b) For each value of correlation in step (a), we simulated a bivariate uniform distribution for each country and year (2018 through 2027). This simulated distribution was ranked in such a way that the marginal distributions in the joint distribution were correlated with that value of correlation coefficient we supplied (this joint distribution is known as a *copula*).

(c) We recorded the ranks of the copula, and sorted our draws (within each country) using those temporal ranks, and therefore we ended up with a complete time-series data for all draws, such that each country and year will follow the same rank correlation structure that exists between 2027 and 2040.

(d) Finally, we calculated our final set of uncertainty intervals by taking the 2.5th and 97.5th percentiles of these correlated draws.

We used this method at the end of forecasting every metric, since one metric fed into the other sequentially. Figure 1 compares the growth rates of the two metrics at each end of our forecasting pipeline: THE per capita and GDP per capita. The annualized growth rates

are for the years 2015 through 2040, and we can see that after we have forecasted all of our metrics, we find a positive correlation between GDP and THE per capita growth rates.

8 Future Health Scenarios

We established the trajectories that our health expenditures are expected to take in the next 23 years using our ensemble models (from hereby referred to as the 'reference' case). The reference forecasts were built upon the basis of each country's past trends and expected future trends from covariates. We additionally also predicted what the possible trajectories for each country would look like if they were to follow the possible optimistic and pessimistic growth rates observed globally (referred to as 'better' and 'worse' cases, respectively).

8.1 Long-term Growth Regressions

In order to determine what the possible better and worse growth rates for each country would be, we ran long-term growth regressions with the following specification:

$$Y_{i,T} - Y_{i,t} = \alpha + \beta Y_{i.t} + \epsilon_{i,t} \tag{7}$$

where the dependent variable represented the long term growth rate of Y for country i, which was computed either as logarithmic or logistic growth rates (for fractions).

The only independent variable we used ($Y_{i,t}$) was the value of Y_i at time t, and it served as a convergence term in this regression. This allowed us to predict the long-term growth rates of Y, conditional on a country's level of Y at time t.

8.2 Forecasting Better and Worse Scenarios

In order to estimate what the future better and worse trajectories would be for each country, we followed these steps (assuming that we are forecasting from 2016 through 2040, with observed data between 1995 and 2015):

(i) We computed the 85th and 15th percentiles of the empirical residuals ϵ, as $Q_{0.85}(\hat{\epsilon_{i,t}})$ and $Q_{0.15}(\hat{\epsilon_{i,t}})$ respectively, where $Q_p(\cdot)$ is a quantile function for a percentile p.

(ii) We computed the starting annualized growth rate from the fitted scenario regression, such that, for country i:

$$\text{Better growth rate} = \exp(\hat{\alpha}) \times \exp(Q_{0.85}(\hat{\epsilon_{i,t}})) \times (Y_{i,2015}^{\hat{\beta}})^{(1/(2015-1995))} \tag{8}$$

$$\text{Worse growth rate} = \exp(\hat{\alpha}) \times \exp(Q_{0.15}(\hat{\epsilon_{i,t}})) \times (Y_{i,2015}^{\hat{\beta}})^{(1/(2015-1995))} \tag{9}$$

(iii) Finally, once we have established the growth rates as a function of the convergence term, we recursively created better and worse trajectories, conditional on the updated

growth rates every year, such that:

$$Y_{i,t+1} = Y_{i,t} \times \exp(\hat{\alpha}) \times \exp(Q_{0.85}(\hat{\epsilon_{i,t}})) \times (Y_{i,t}^{\hat{\beta}}) \tag{10}$$

where $\exp(\hat{\alpha}) \times \exp(Q_{0.85}(\hat{\epsilon_{i,t}})) \times (Y_{i,t}^{\hat{\beta}})$ was the conditional growth rate for a single year.

One condition that we imposed for the computed scenarios is: the better projection cannot be lower than the reference projection, and the worse projection cannot be higher than the reference projection. For countries with wide forecasts where this case did happen, we moved the better and worse forecasts down and up to overlap on top of the reference line respectively.

8.3 Uncertainty Estimation

The uncertainty intervals around a scenario were expected to take the same shape as the uncertainty around our reference forecasts. Therefore, once we have propagated a mean set of better and worse forecasts in section 8.2, we created the draws around our scenarios in the following way:

(i) We took our reference forecast's mean line and the 1,000 draws around that line.

(ii) We computed the deviation of the mean from each of the draws (in logarithmic or logistic transformation, depending on the space of the covariate).

(iii) We took each of the scenario mean lines and added the deviations from the previous step to the mean lines, giving us 1,000 draws of the scenario projections.

Figure 2 shows the distribution of the growth rates between the reference and scenarios of THE per capita. The green and red densities, respectively, are the conditional better and worse growth rates of THE per capita; the blue histogram shows the reference growth rate; the scatterplot shows each of better and worse growth rates scattered against reference.

9 Universal Health Coverage Index

Universal health coverage (UHC) has emerged as both a global and national health priority, with achieving UHC viewed as a critical path to improved health outcomes and greater equity in health across all populations. This series was produced as part of the Global Burden of Disease Sustainable Development Goals analysis [16], for 188 countries between 1990 through 2016. This section focuses the method used for forecasting the UHC index from 2016 through 2040 using our health financing variables, particularly the sum of GHE, DAH and PPP per capita, hereby referred as 'pooled health resources per capita' or 'pooled spending per capita'.

9.1 Definition

The UHC index included nine measures of coverage for a subset of interventions for communicable diseases and maternal and child health and the 32 causes that comprise the HAQ Index, a summary measure of personal healthcare access and quality based on riskâĂŘstandardised death rates from causes amenable to healthcare. The measurement approach used for GBD 2016 represents a substantial improvement since GBD 2015, considerably expanding the representation of essential health services pertaining to reproductive, maternal, newborn, and child health (RMNCH); infectious diseases; nonâĂŘcommunicable diseases; and service capacity and access. Each component of the UHC index was scaled on a scale of 0 to 100, with 0 being the worst observed from 1990 to 2016 and 100 being the best observed during this time, and then the arithmetic mean was taken of each component. We then projected the UHC index, based on past trends, as a composite indicator from 2017 to 2030.

The measures of intervention coverage were as follows: three doses of diphtheriaâĂŘpertussisâĂŘtetanus (DPT3), measles vaccine, three doses of the oral polio vaccine or inactivated polio vaccine; met need for family planning with modern methods; antenatal care (ANC) coverage (one ANC visit [ANC1] and four ANC visits [ANC4]); skilled birth attendance (SBA); inâĂŘfacility delivery rates; and coverage of antiretroviral therapy (ART) among people living with HIV.ÂăÂă

The causes that comprised the HAQ Index are as follows: tuberculosis, diarrheal diseases, lower respiratory infections, upper respiratory infections, diphtheria, whooping cough, tetanus, measles, maternal disorders, neonatal disorders, colon and rectum cancer, nonâĂŘmelanoma cancer, breast cancer, cervical cancer, uterine cancer, testicular cancer, Hodgkin's lymphoma, leukemia, rheumatic heart disease, ischemic heart disease, cerebrovascular disease, hypertensive heart disease, peptic ulcer disease, appendicitis, hernia, gallbladder and biliary diseases, epilepsy, diabetes, chronic kidney disease, congenital heart anomalies, and

adverse effects of medical treatment.

To construct the composite UHC Index, cause-specific death rates were risk-standardized and draw-level estimates for both intervention coverage and risk-standardized cause-specific death rates were computed as part of GBD 2016. For each input, 1,000 draws were used in order to estimate uncertainty. Then each of the UHC Index component were scaled on a scale of 0 to 100 from 1990 to 2016, followed by taking the arithmetic mean across components. More details on the modeling and creation of the HAQ and UHC index can be found in the supplementary appendix of the SDG analysis paper [16], SDG Indicator 3.8.

In this paper, we treated the UHC index as a fraction of the population with UHC; therefore, we computed the number of lives covered by UHC by multiplying the UHC index (in ratio space) with the population of the country for each time period.

9.2 Forecasting UHC

9.2.1 Stochastic Frontier Analysis

We used a stochastic frontier model to forecast the level of UHC index achievable by all countries between 2016 through 2040. Implementing the work of Battese and Coelli [17, 18], our SFA model, with a production function specification, was such:

$$\ln(UHC_{i,t}) = \alpha + \beta \ln(X_{i,t}) - \nu_{i,t} + \epsilon_{i,t} \tag{11}$$

$$\nu_{i,t} \sim N^{+}(0, \sigma_{\nu}^{2}) \tag{12}$$

$$\epsilon_{i,t} \sim N(0, \sigma_{\epsilon}^{2}) \tag{13}$$

where our observed outcome was the logged UHC index, with our single covariate X being the country-year specific pooled spending per capita, $\epsilon_{i,t}$ is the noise component and $\nu_{i,t}$ is the estimated technical efficiency that a country would need to achieve the optimal, frontier goal. The prior distribution of technical efficiency is a half-normal distribution, describing an unbounded distribution between zero and very high efficiency.

9.2.2 Forecasting Steps

We forecasted the UHC index from 2016 through 2040 in the following steps:

(i) Forecasts of the pooled spending were developed by adding the forecasts of GHE, DAH and PPP per capita, modeled previously using ensembles.

(ii) For each of the error components (efficiency ν and noise ϵ) for a country, we added them together to create a unified residual time series for each country. That series

was separately forecasted for each country using a weighted ordinary linear regression (using a linear time trend as a covariate), where recent time periods were weighed higher than the further past.

(iii) Using the draws of reference, better and worse scenarios of $X_{i,t}$ along with forecasts of the summed residuals from (ii), we created reference, better and worse projections of the UHC index from 2016 through 2040.

Figure **??** contains the full time-series of the UHC index for each individual country, for the reference, better and worse scenarios.

9.2.3 Decomposition of Forecasting Components

Once we have created forecasts of the UHC index in equation 11, we split out the partial effects of each of the components that contributed to the prediction of the UHC index, namely pooled resources, and efficiency and noise. The model used in equation 11 predicted a log-linear relationship between UHC and the predictors; we measured the additive effect of our predictors using the Das Gupta decomposition [19], the steps of which are as follows:

From equation 11, exponentiating both sides give us the following identity:

$$UHC = X_{i,t}^{\beta} * \exp(\gamma_{i,t}) \tag{14}$$

where $\gamma_{i,t}$ is the additive efficiency and error term.
The Das Gupta decomposition will measure $R_1 = AB$ and $R_2 = ab$, such that:

$$R_1 = AB \tag{15}$$
$$R_2 = ab \tag{16}$$
$$A = X_{i,2015}^{\beta} \tag{17}$$
$$a = X_{i,2040}^{\beta} \tag{18}$$
$$B = \exp(\gamma_{i,2015}) \tag{19}$$
$$b = \exp(\gamma_{i,2040}) \tag{20}$$

such that:

$$UHC_{i,2040} - UHC_{i,2015} = \delta_1 + \delta_2 \tag{21}$$

$$\delta_1 = \frac{(b+B)(a-A)}{2} \tag{22}$$

$$\delta_2 = \frac{(a+A)(b-B)}{2} \tag{23}$$

Figure 4 of the manuscript shows the δ_1 and δ_2 effects aggregated to the global level, World Bank income group, and the GBD super-regions.

9.3 Comparison of Forecasts

We forecasted the UHC index relying solely on the level and projection of health resources. To compare with the original analysis and modeling of the UHC index, figure 3 shows the difference between our projections of UHC against the projections created originally by Lim et al [16], grouped by the seven GBD super regions. Figure 5 additionally shows the change in the value of UHC between 2015 and 2030 between the two aforementioned series. The dots on both figures represent a country. The figures show that, while the level of UHC projected in 2030 ends up being very similar across all regions between the two series, the rate of growth needed to achieve those values vary non-uniformly between the two estimation processes, even within a single super region.

In order to test the robustness of our SFA model, we ran an extra set of forecasts for the UHC index using a truncated normal distribution instead of the half-normal distribution for the inefficiency term. The coefficient on the pooled health resources per capita were very similar, with the different not being statistically significant (0.1434 versus 0.1413); figure 6 shows the values of the 2030 forecasts of these two models against each other, and the difference are only in the decimal places, hence lining up almost perfectly on the unity line.

A Tables and Figures

Table 1: Definitions of health expenditure sources

Health Expenditure Type	Definition
Development assistance for health	Financial and in-kind contributions from global health channels that aim to improve or maintain health in low- or middle-income countries.
Government health expenditure as source	Government health expenditure as source only includes domestically financed government expenditure on health, including public health spending.
Out-of-pocket expenditure	Paid by individuals for health services; considered catastrophic if exceeding 40% of a household's annual income.
Prepaid private health expenditure	Private risk pooling against catastrophic health expenditure; includes private insurance and non-governmental organizations.

Table 2: Summary statistics of the covariates used

Statistic	N	Mean	St. Dev.	Min	Max
Log GDP per capita	3,948	8.996	1.253	5.616	11.827
Logit GGE per GDP	3,948	−1.151	0.503	−3.178	2.664
Log of total population	3,948	15.638	1.964	10.840	21.058
Logit of proportion of population aged 14 years and under	3,948	−0.886	0.459	−1.963	−0.160
Logit of proportion of population aged 65 years and over	3,948	−2.780	0.724	−4.885	−1.045
Log total fertility rate	3,948	1.020	0.513	0.049	2.045
Log of total education per capita	3,948	2.065	0.453	0.151	2.703
Logit of DAH per GDP	3,948	−6.361	1.464	−7.600	0.110
Logit of GHE per GDP	3,948	−3.645	0.745	−6.468	−1.949
Logit of PPP per GDP	3,948	−6.313	1.749	−16.658	−2.678
Logit of OOP per GDP	3,948	−4.051	0.705	−6.989	−1.974

A.1 State Space Diagram of Forecasting Components

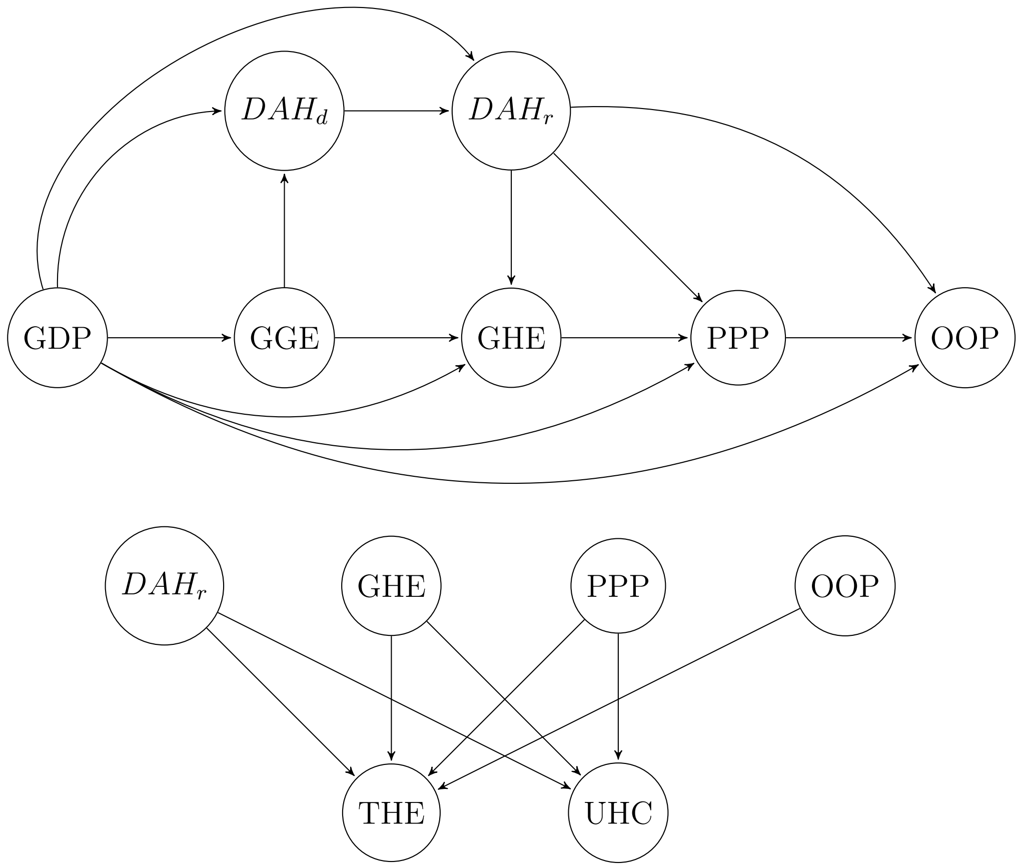

A.2 Prediction and Covariates Map with Transformations

	Predicted Variables	Covariates	Extra Specifications
1	GDP and GDPpc (both in log)	Pop, Pop <15$^-$, Pop >64$^+$, TFR$^-$, Education$^+$	ARIMA(1-3, 1, 1-3) and and 4 up-weighting schemes
2	GGE per GDP (logit)	GDPpc$^+$, Pop >64$^+$, Education$^+$	ARIMA(1-2, 1, 1-3) and and 4 up-weighting schemes
3	DAH donated per 2nd GDP lag (logit)	GDPpc$^+$, GGE/GDP$^+$, Bush Era Dummy$^+$	ARIMA(1-2, 1, 1-3)
4	DAH received per total DAH donated (logit)	GDPpc$^-$, Pop$^-$, Pop <15$^+$, TFR$^+$, Ebola dummy$^+$, total DAH envelope	ARIMA(1, 1, 1)
5	GHE per GGE (logit)	GDPpc$^+$, Pop >64$^+$, GGEpc$^+$, DAH/GDP$^-$	ARIMA(0, 1, 0)
6	PPP per GDP (logit)	GDPpc$^+$, Pop >64$^+$, DAH/GDP, GHE/GDP,	ARIMA(1-2, 1, 1-3)
7	OOP per GDP (logit)	GDPpc$^-$, Pop >64$^+$, DAH/GDP, GHE/GDP, PPP/GDP	ARIMA(1-2, 1, 1-3)
8	UHC (log)	Sum of GHEpc, DAHpc and PPPpc	Stochastic Frontier Analysis

A.3 Comparison of Forecasts

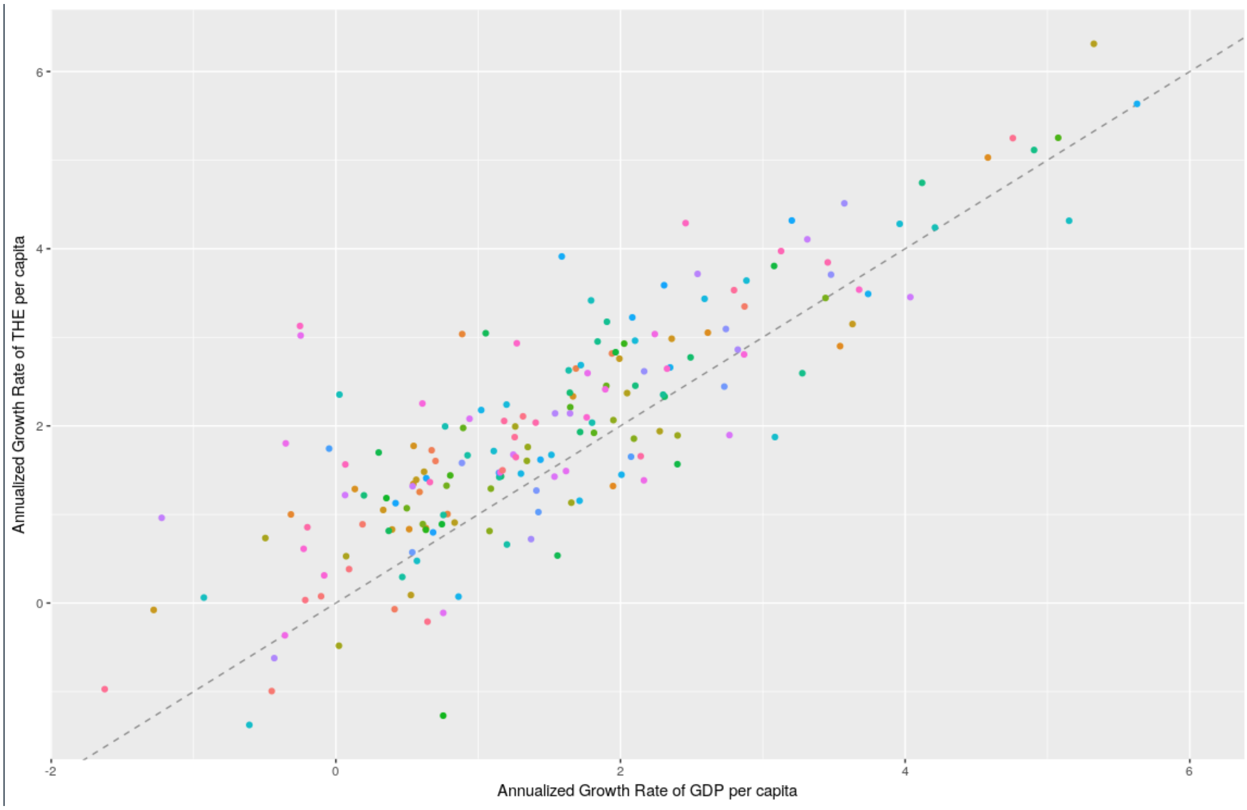

Figure 1: Growth rates of THE per capita against GDP per capita between 2015 and 2040 inclusive

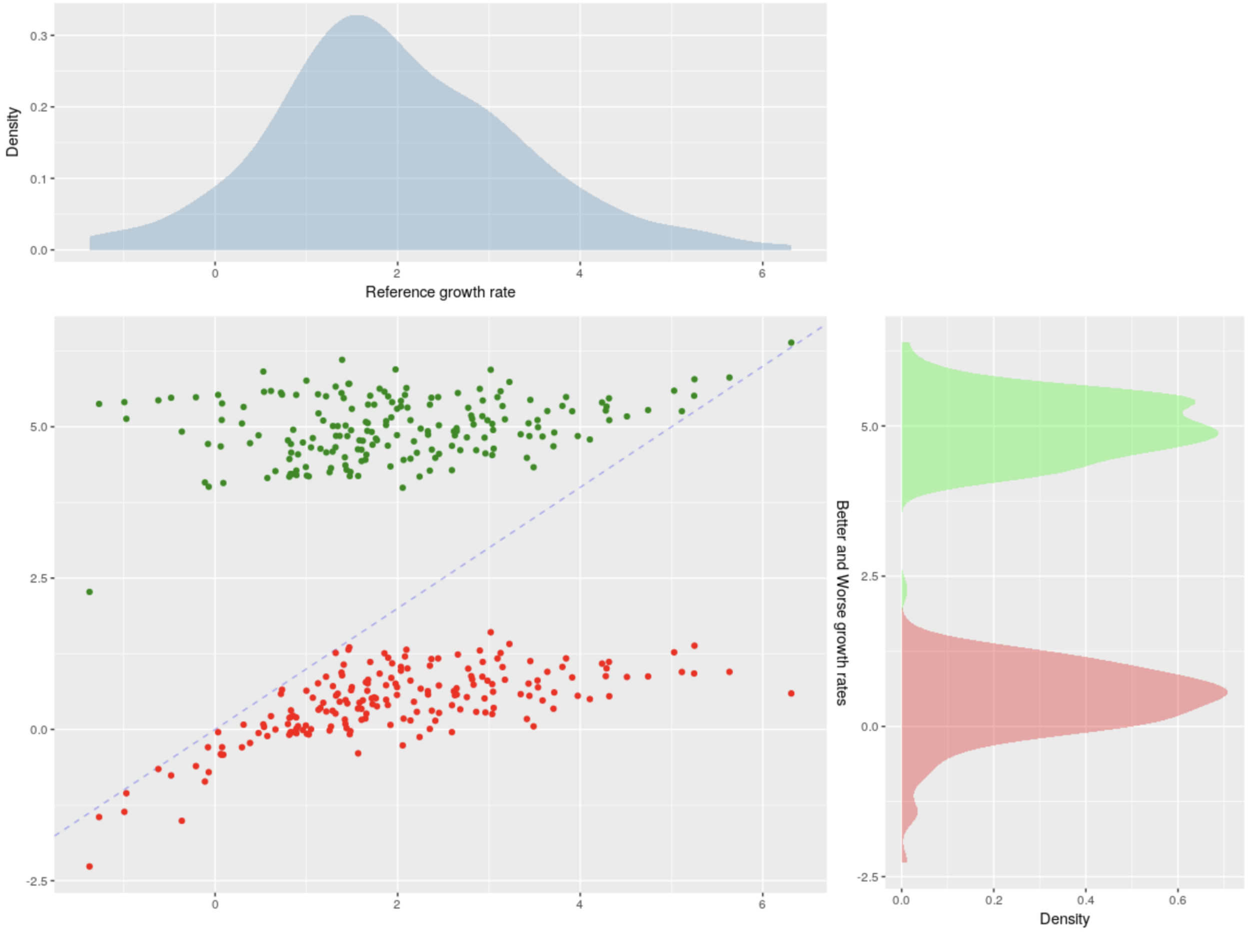

Figure 2: Growth rates of THE per capita scenarios between 2015 and 2040 inclusive

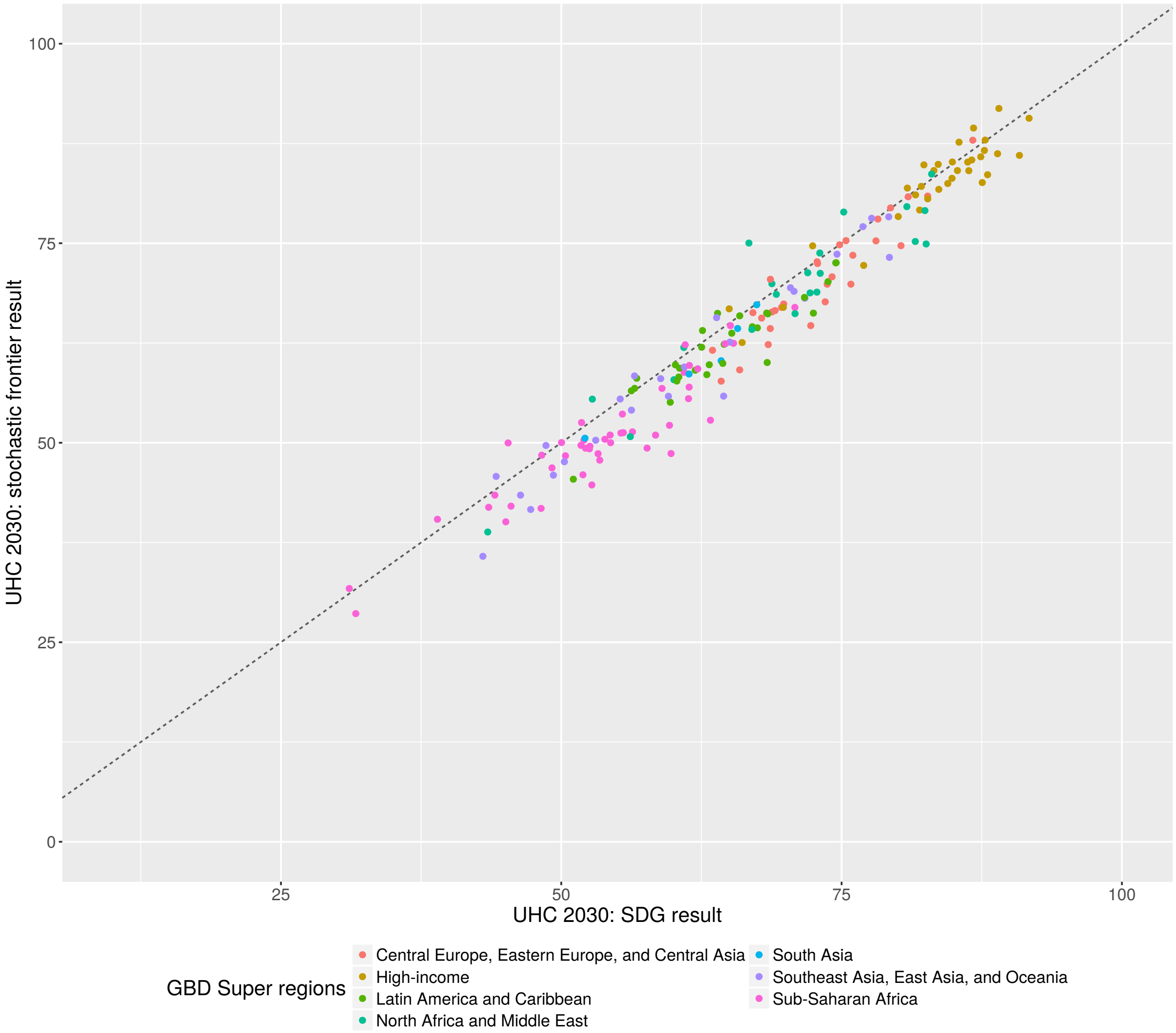

Figure 3: Comparing UHC index forecasts for the year 2030

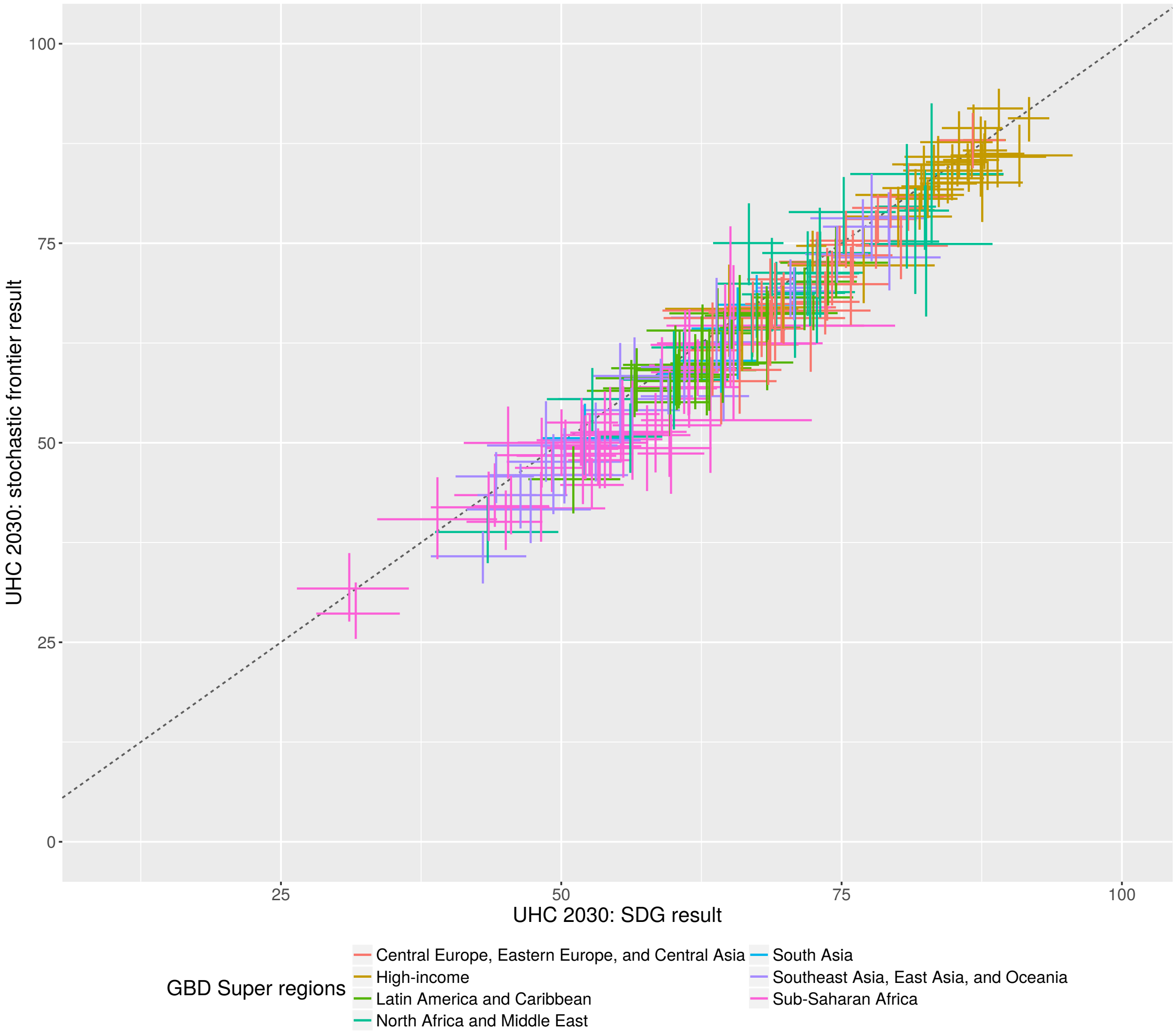

Figure 4: Comparing UHC index forecasts for the year 2030 with uncertainty intervals

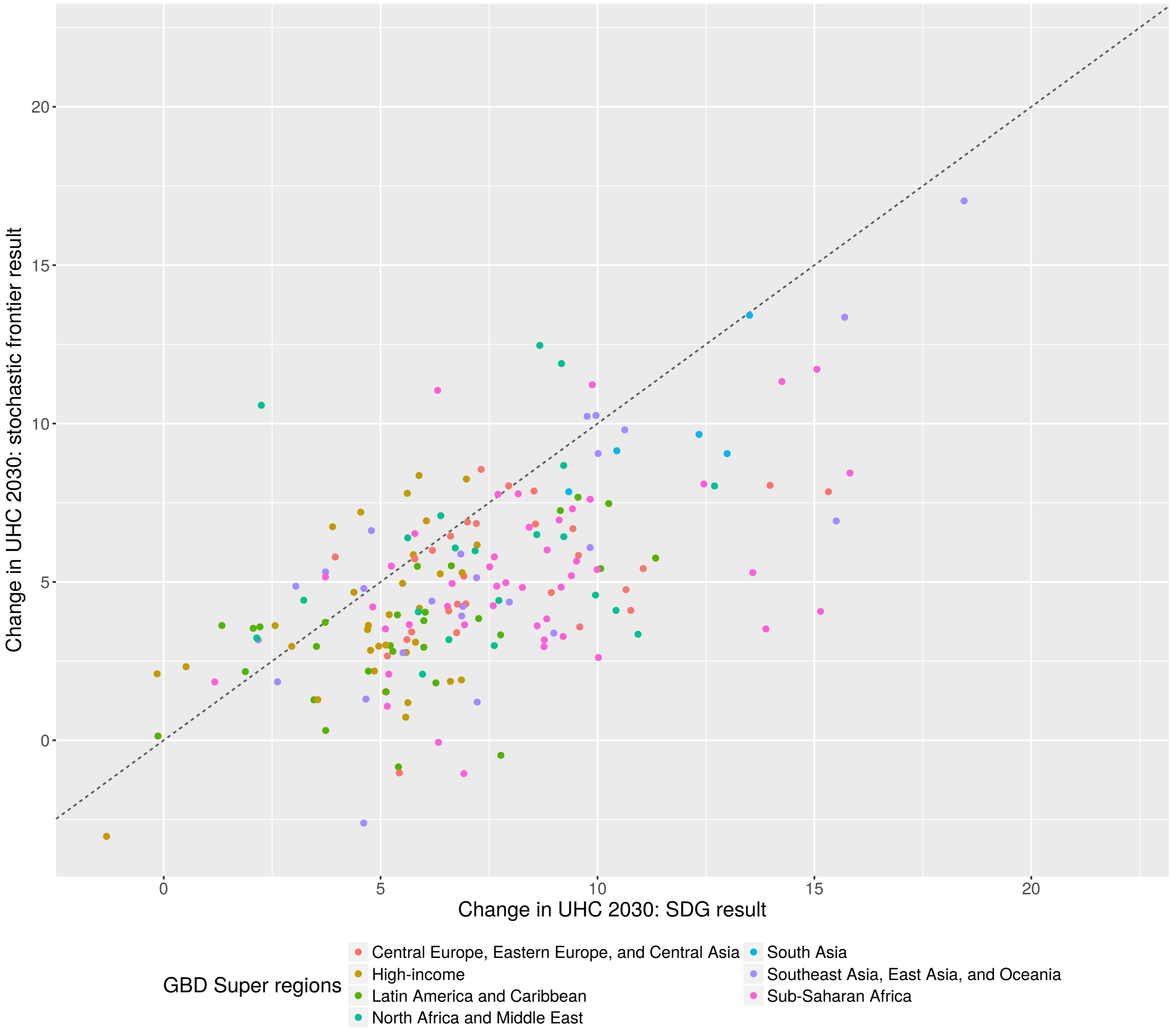

Figure 5: Comparing change in UHC index forecasts between years 2015 and 2030

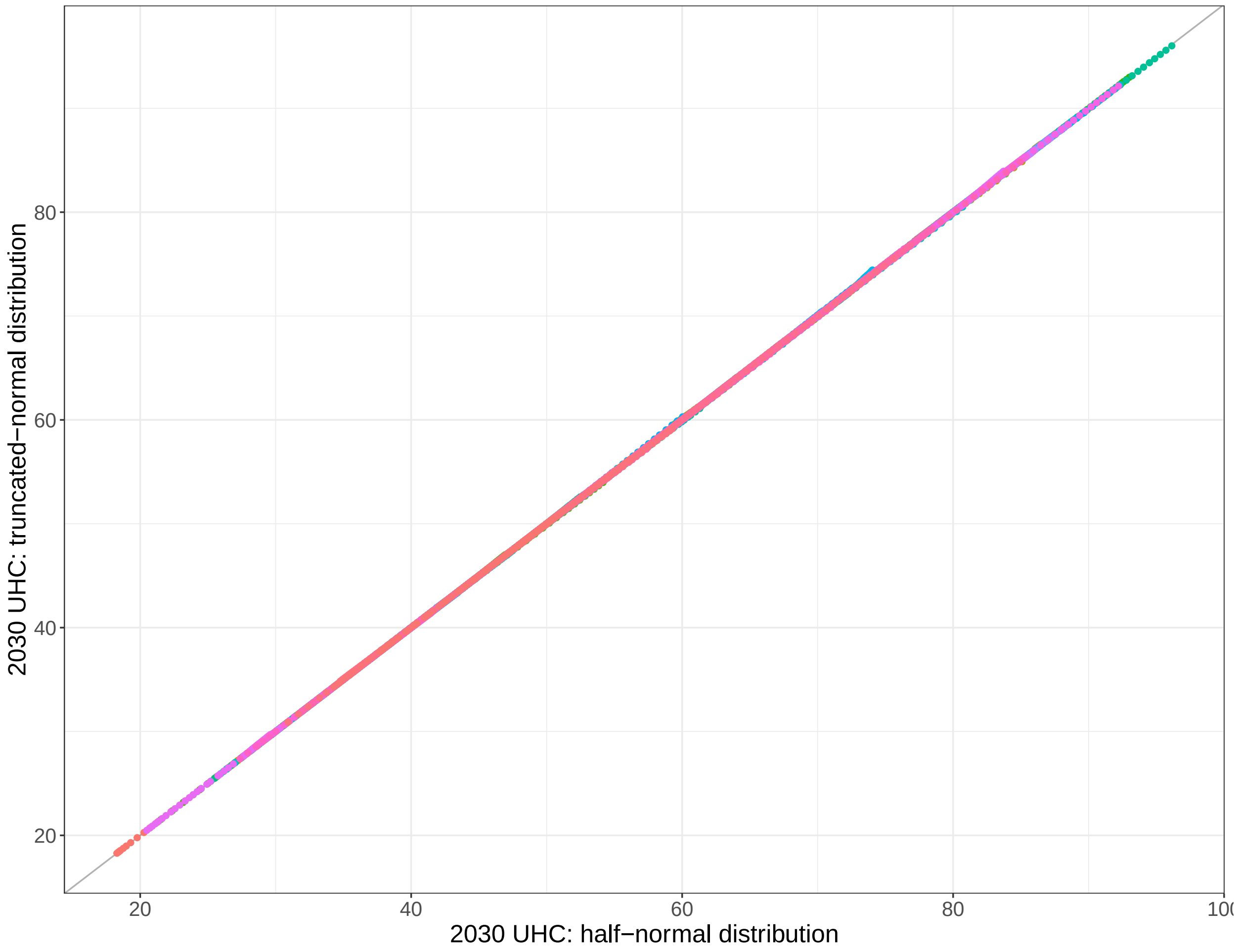

Figure 6: Comparing forecasts using half-normal and truncated normal distributions for the inefficiency term

A.4 Sensitivity Analyses on UN WPP Variants

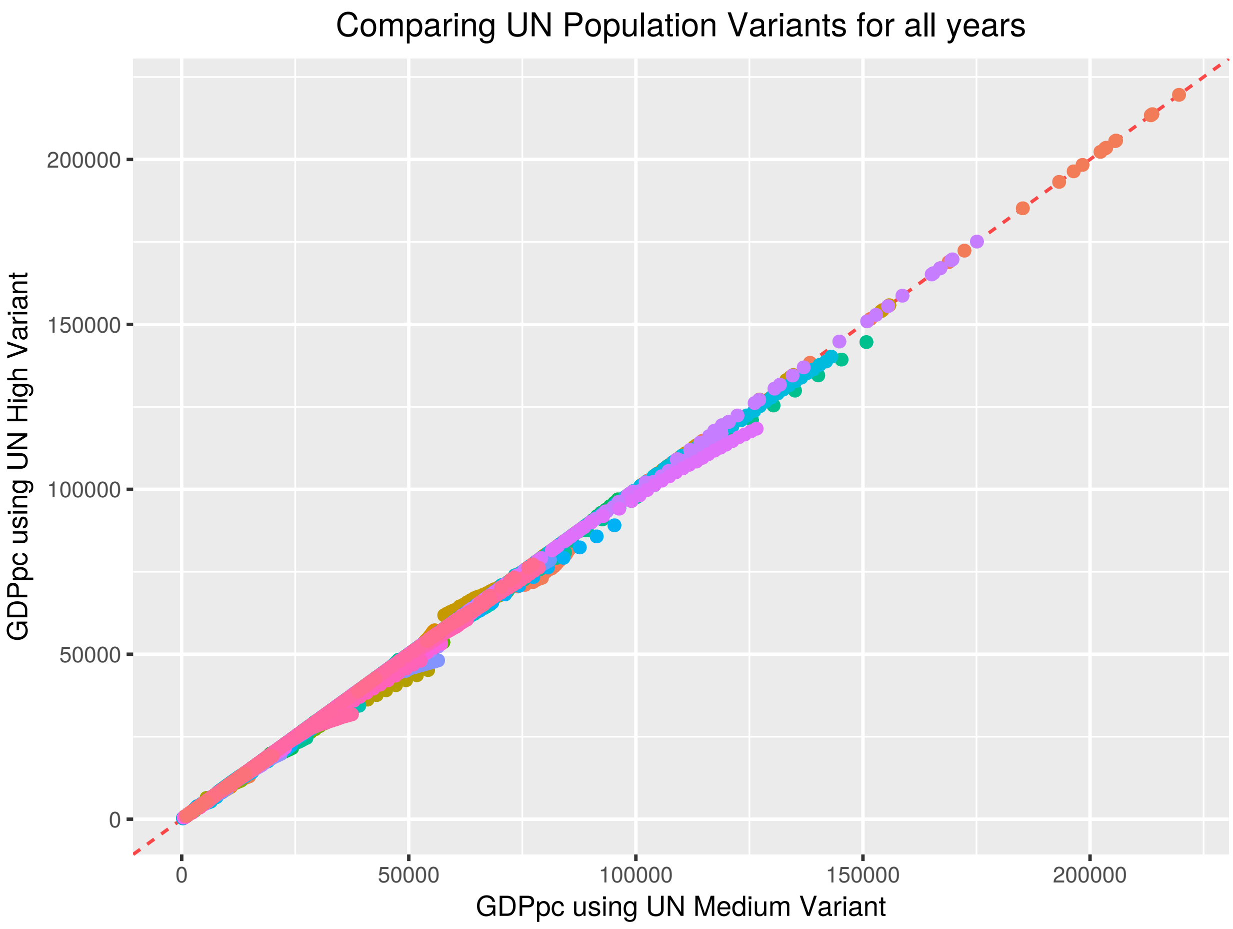

Figure 7: Comparing GDP per capita forecasts between the High and Medium UN Variants for all years

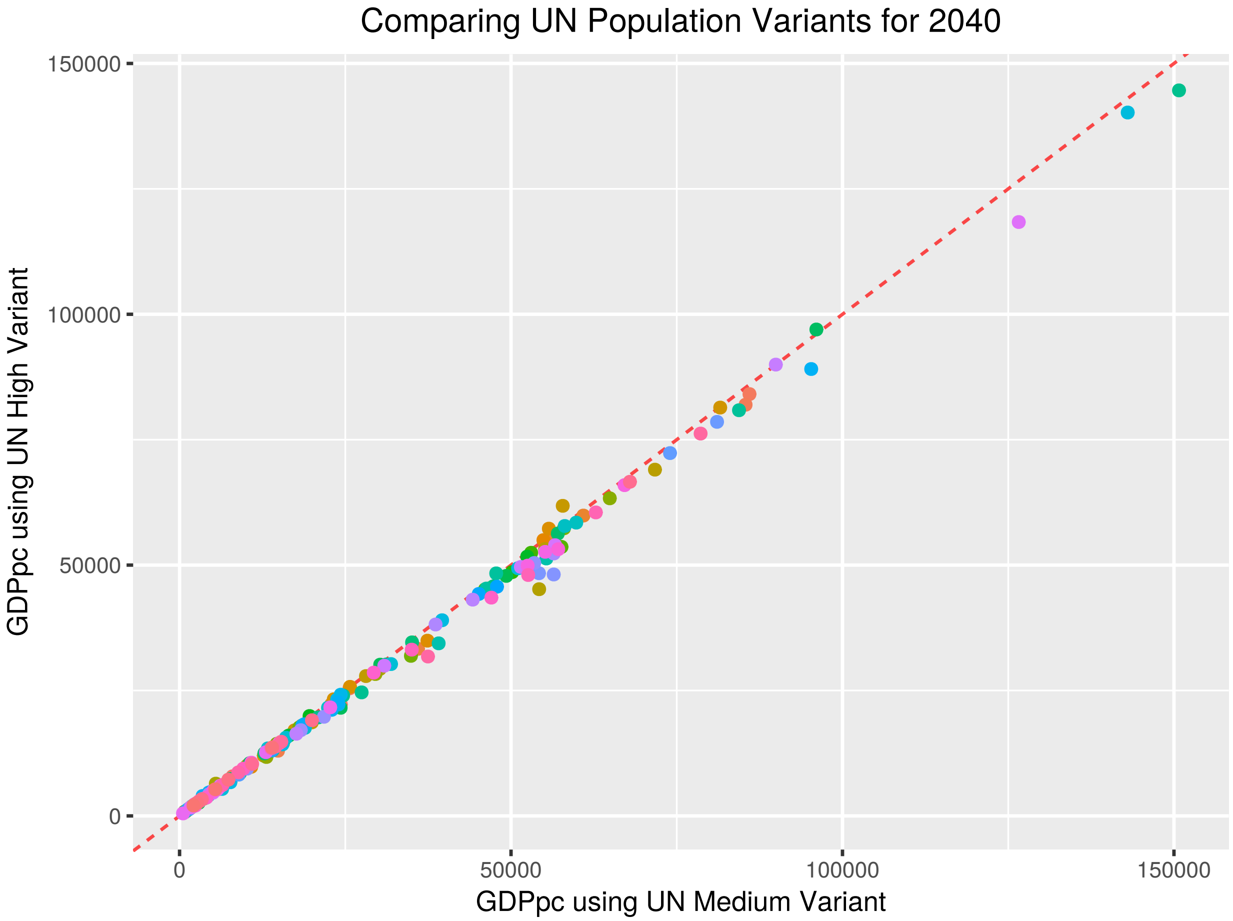

Figure 8: Comparing GDP per capita forecasts between the High and Medium UN Variants for 2040

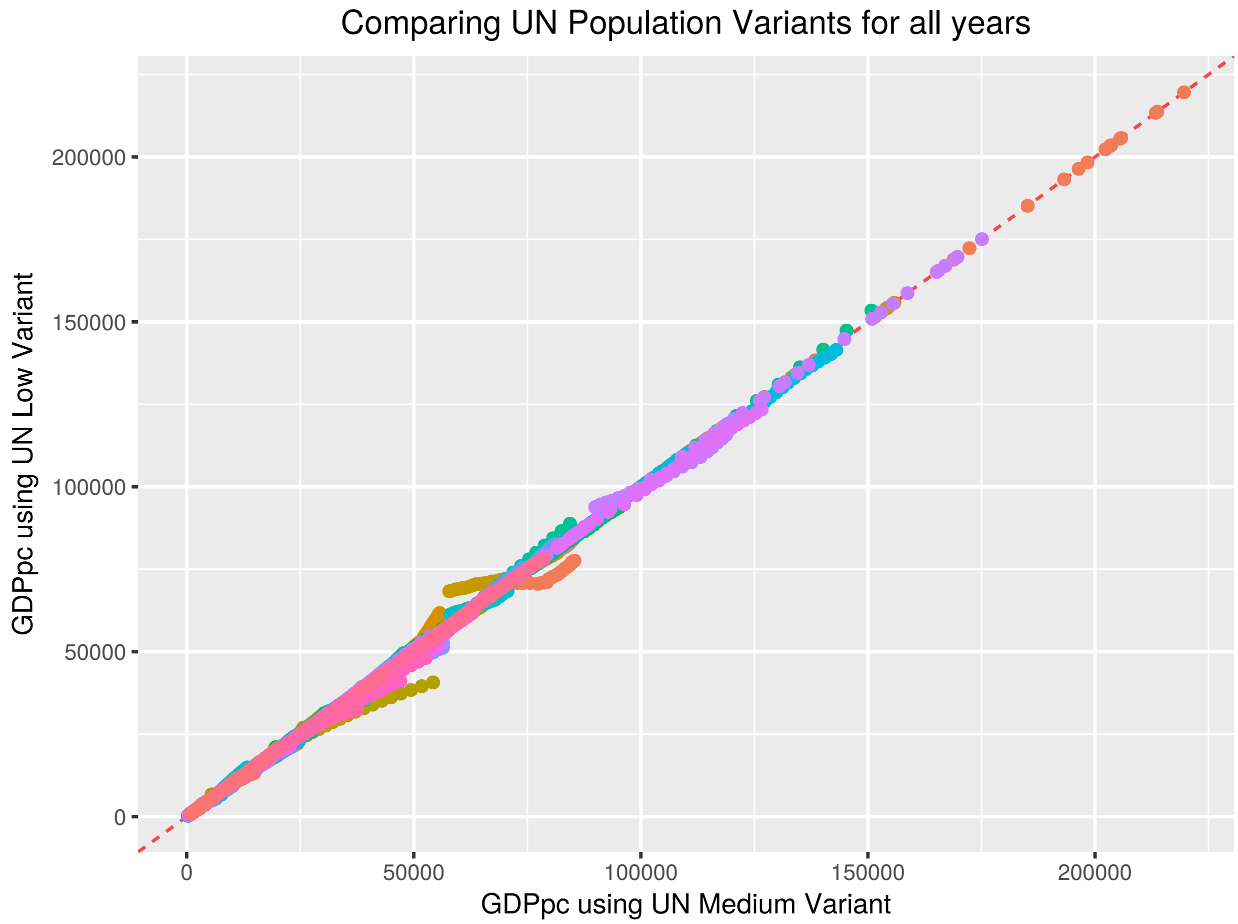

Figure 9: Comparing GDP per capita forecasts between the Low and Medium UN Variants for all years

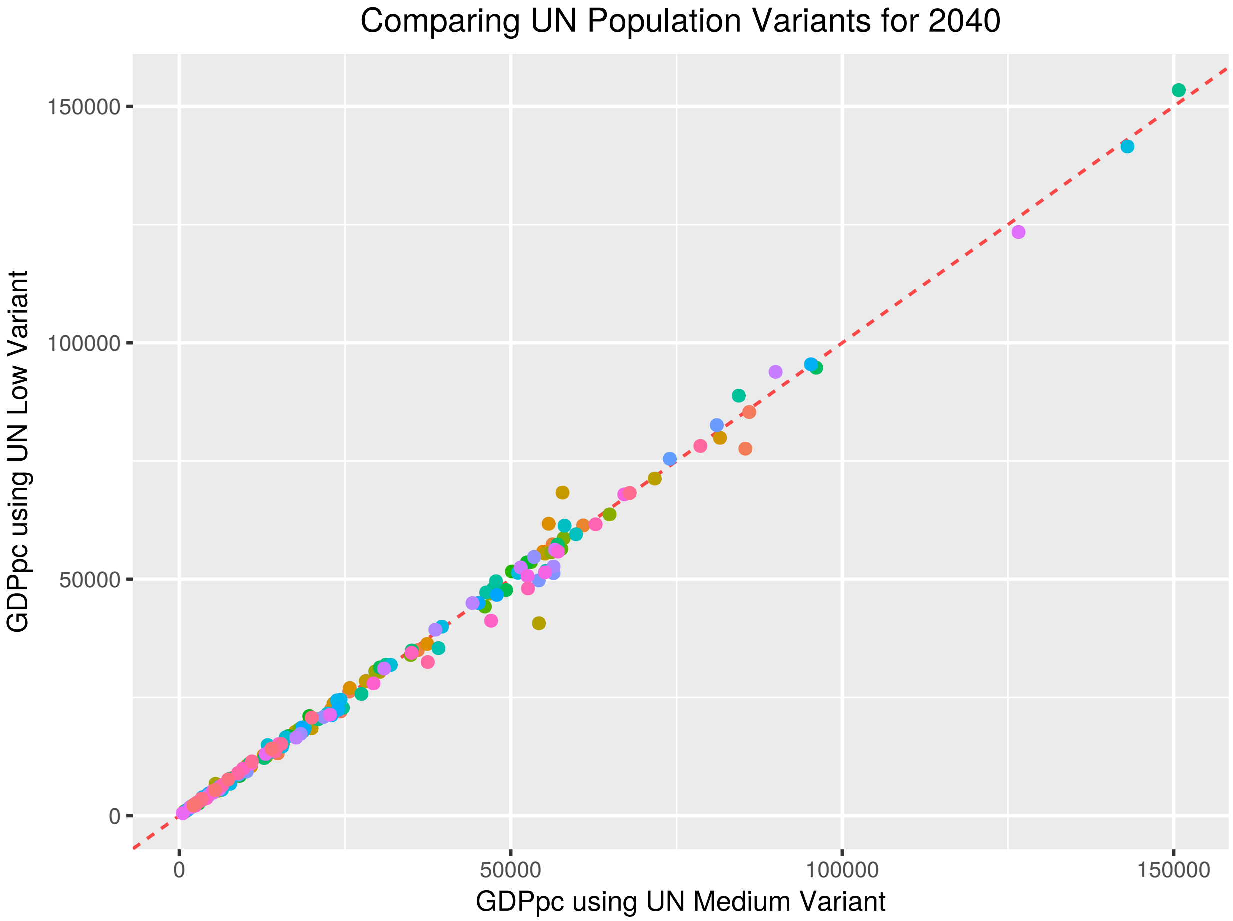

Figure 10: Comparing GDP per capita forecasts between the Low and Medium UN Variants for 2040

References

[1] Global Burden of Disease Financing Global Health Collaborator Network. Tracking spending on health and HIV/AIDS: 188 countries, 1995-2015. *Submitted Paper*, 2017.

[2] Emmanuela Gakidou, Ashkan Afshin, Amanuel Alemu Abajobir, Kalkidan Hassen Abate, Abbafati, Ben Zipkin, Sanjay Zodpey, Stephen S Lim, and Christopher J L Murray. Global, regional, and national comparative risk assessment of 84 behavioural, environmental and occupational, and metabolic risks or clusters of risks, 1990-2016: a systematic analysis for the Global Burden of Disease Study 2016. *The Lancet*, 390 (10100):1345–1422, nov 2017. ISSN 0140-6736. doi: 10.1016/S0140-6736(17)32366-8. URL `http://dx.doi.org/10.1016/S0140-6736(17)32366-8`.

[3] Measuring progress and projecting attainment on the basis of past trends of the health-related sustainable development goals in 188 countries: an analysis from the global burden of disease study 2016. *The Lancet*, 390(10100):1423 – 1459, 2017. ISSN 0140-6736. doi: https://doi.org/10.1016/S0140-6736(17)32336-X. URL `http://www.sciencedirect.com/science/article/pii/S014067361732336X`.

[4] Adrian E Raftery, Leontine Alkema, and Patrick Gerland. Bayesian population projections for the united nations. *Statistical science: a review journal of the Institute of Mathematical Statistics*, 29(1):58, 2014.

[5] United Nations. United nations, department of economic and social affairs, population division (2017). world population prospects: The 2017 revision, methodology of the united nations population estimates and projections. *Working Paper No. ESA/P/WP.250*, 2017.

[6] US census bureau. `https://www.census.gov/topics/population.html`. Accessed: 2017-12-20.

[7] Spencer L James, Paul Gubbins, Christopher JL Murray, and Emmanuela Gakidou. Developing a comprehensive time series of gdp per capita for 210 countries from 1950 to 2015. *Population health metrics*, 10(1):12, 2012.

[8] Emmanuela Gakidou, Krycia Cowling, Rafael Lozano, and Christopher JL Murray. Increased educational attainment and its effect on child mortality in 175 countries between 1970 and 2009: a systematic analysis. *The Lancet*, 376(9745):959–974, 2010.

[9] R Core Team. *R: A Language and Environment for Statistical Computing.* R Foundation for Statistical Computing, Vienna, Austria, 2017. URL `https://www.R-project.org/`. Accessed: 2017-12-20.

[10] Finn Lindgren and Håvard Rue. Bayesian spatial modelling with R-INLA. *Journal of Statistical Software*, 63(19):1–25, 2015. URL `http://www.jstatsoft.org/v63/i19/`.

[11] Thiago G. Martins, Daniel Simpson, Finn Lindgren, and Håvard Rue. Bayesian computing with INLA: New features. *Computational Statistics and Data Analysis*, 67:68–83, 2013.

[12] Håvard Rue, Andrea I. Riebler, Sigrunn H. Sørbye, Janine B. Illian, Daniel P. Simpson, and Finn K. Lindgren. Bayesian computing with INLA: A review. *Annual Reviews of Statistics and Its Applications*, 4(March):395–421, 2017. URL `http://arxiv.org/abs/1604.00860`.

[13] Håvard Rue, Sara Martino, and Nicholas Chopin. Approximate Bayesian inference for latent Gaussian models using integrated nested Laplace approximations (with discussion). *Journal of the Royal Statistical Society B*, 71:319–392, 2009.

[14] Finn Lindgren, Håvard Rue, and Johan Lindström. An explicit link between Gaussian fields and Gaussian Markov random fields: The stochastic partial differential equation approach (with discussion). *Journal of the Royal Statistical Society B*, 73(4):423–498, 2011.

[15] Kyle J Foreman, Rafael Lozano, Alan D Lopez, and Christopher JL Murray. Modeling causes of death: an integrated approach using codem. *Population health metrics*, 10(1): 1, 2012.

[16] Stephen S Lim, Kate Allen, Zulfiqar A Bhutta, Lalit Dandona, Mohammad H Forouzanfar, Nancy Fullman, Peter W Gething, Ellen M Goldberg, Simon I Hay, Mollie Holmberg, et al. Measuring the health-related sustainable development goals in 188 countries: a baseline analysis from the global burden of disease study 2015. *The Lancet*, 388(10053): 1813–1850, 2016.

[17] George Edward Battese and Tim J Coelli. A model for technical inefficiency effects in a stochastic frontier production function for panel data. *Empirical economics*, 20(2): 325–332, 1995.

[18] George E Battese and Tim J Coelli. Frontier production functions, technical efficiency and panel data: with application to paddy farmers in india. *Journal of productivity analysis*, 3(1-2):153–169, 1992.

[19] Prithwis Das Gupta. *Standardization and decomposition of rates: a user's manual.* Number 186. US Department of Commerce, Economics and Statistics Administration, Bureau of the Census, 1993.

B Additional Tables and Figures

The following are the list of additional tables and figures containing our data and results from our analysis.

B.1 Table: Future Health Spending in 2015 and 2030

This table shows the total health spending per capita values (2017 purchasing power parity US$) in 2015 and 2030, the share of each health expenditure component per total spending, and the annualized rate of change of the components of health spending per capita between 2015 and 2030.

			Health spending per total, 2030				Per capita annualized rate of change, 2015-2030				
Location name	Total health spending per capita 2015 ($)	Total health spending per capita 2030 ($)	Government (%)	Pre-paid private (%)	Out-of-pocket (%)	Development assistance for health (%)	Total (%)	Government (%)	Pre-paid private (%)	Out-of-pocket (%)	Development assistance for health (%)
**Global**	1332 (1325 to 1343)	1846 (1710 to 1965)	60.6 (57.8 to 64.6)	14.8 (10.2 to 17.4)	24.1 (22.2 to 26.2)	0.5 (0.5 to 0.6)	2.2 (1.7 to 2.6)	2.3 (1.8 to 2.9)	1.0 (-1.7 to 2.2)	2.7 (2.3 to 3.2)	2.1 (1.5 to 2.7)
**World Bank Income Groups**											
**High-income**	5551 (5503 to 5605)	7229 (6432 to 7800)	66.0 (62.0 to 72.9)	19.9 (12.2 to 24.0)	14.1 (12.7 to 16.1)	0.0 (0.0 to 0.0)	1.8 (1.0 to 2.3)	2.0 (1.4 to 2.5)	1.1 (-2.6 to 2.6)	1.7 (1.2 to 2.3)	-
**Upper-middle-income**	949 (942 to 959)	1796 (1594 to 2081)	61.5 (56.0 to 67.3)	7.9 (6.1 to 10.3)	30.5 (25.8 to 35.6)	0.1 (0.1 to 0.2)	4.3 (3.5 to 5.4)	4.8 (3.5 to 6.4)	2.5 (0.9 to 4.5)	4.0 (3.0 to 5.0)	1.6 (0.2 to 3.3)
**Lower-middle-income**	266 (263 to 268)	484 (448 to 524)	32.4 (28.8 to 35.8)	8.0 (6.6 to 9.5)	57.6 (53.9 to 61.5)	2.1 (1.8 to 2.4)	4.1 (3.5 to 4.6)	4.2 (3.4 to 5.0)	4.6 (3.3 to 5.9)	4.0 (3.2 to 5.0)	1.3 (0.5 to 2.2)
**Low-income**	110 (108 to 111)	147 (135 to 162)	28.6 (23.8 to 33.6)	9.4 (6.6 to 13.9)	36.3 (32.3 to 40.8)	25.7 (22.8 to 28.7)	2.0 (1.3 to 2.6)	3.9 (2.4 to 5.5)	3.8 (1.3 to 7.0)	1.5 (0.7 to 2.3)	0.9 (0.1 to 1.8)
**GBD Super-Regions**											
**Central Europe, Eastern Europe, and Central Asia**	1288 (1273 to 1300)	1789 (1599 to 2010)	57.9 (52.2 to 63.2)	3.0 (2.4 to 3.7)	38.7 (33.5 to 44.6)	0.4 (0.3 to 0.5)	2.2 (1.4 to 3.0)	1.7 (0.7 to 2.9)	2.2 (0.7 to 3.8)	2.9 (1.8 to 4.2)	4.4 (3.2 to 5.8)
**GBD high-income**	5839 (5785 to 5897)	7561 (6681 to 8179)	66.0 (61.9 to 73.3)	20.4 (12.3 to 24.7)	13.6 (12.2 to 15.6)	0.0 (0.0 to 0.0)	1.7 (0.9 to 2.3)	1.9 (1.4 to 2.5)	1.0 (-2.8 to 2.6)	1.6 (1.1 to 2.2)	-40.0 (-91.7 to 0.8)
**Latin America and Caribbean**	1065 (1051 to 1077)	1323 (1187 to 1458)	50.4 (45.3 to 55.4)	18.2 (13.6 to 21.9)	31.0 (27.0 to 35.5)	0.4 (0.3 to 0.6)	1.4 (0.7 to 2.1)	1.5 (0.4 to 2.6)	1.7 (-0.4 to 3.0)	1.2 (0.3 to 2.2)	-1.6 (-3.0 to 0.0)
**North Africa and Middle East**	888 (872 to 905)	1246 (1089 to 1425)	58.1 (52.0 to 64.3)	7.5 (5.5 to 10.2)	34.0 (28.1 to 39.7)	0.4 (0.3 to 0.5)	2.3 (1.4 to 3.2)	2.1 (0.7 to 3.5)	2.5 (0.6 to 4.8)	2.5 (1.3 to 3.9)	1.8 (0.4 to 3.4)
**South Asia**	210 (207 to 212)	432 (379 to 496)	28.6 (23.4 to 33.9)	9.2 (6.6 to 12.0)	61.3 (55.3 to 67.1)	0.9 (0.7 to 1.2)	4.9 (4.0 to 5.9)	5.7 (4.1 to 7.2)	6.0 (3.8 to 8.1)	4.5 (3.3 to 5.8)	-0.7 (-2.2 to 0.9)
**Southeast Asia, East Asia, and Oceania**	672 (663 to 682)	1591 (1332 to 1955)	61.3 (53.9 to 69.3)	6.2 (4.0 to 9.3)	32.3 (25.4 to 39.3)	0.2 (0.1 to 0.2)	5.9 (4.7 to 7.4)	6.5 (4.6 to 8.8)	3.5 (0.9 to 6.7)	5.3 (4.1 to 6.6)	1.2 (-0.1 to 2.7)
**Sub-Saharan Africa**	202 (199 to 206)	251 (230 to 275)	35.2 (31.0 to 40.1)	11.4 (9.3 to 13.9)	38.4 (33.5 to 43.3)	15.0 (13.3 to 16.8)	1.4 (0.8 to 2.1)	1.6 (0.6 to 2.7)	-0.7 (-2.0 to 0.9)	2.3 (1.1 to 3.6)	1.2 (0.5 to 2.0)
**Countries**											
**Afghanistan**	168 (160 to 174)	134 (104 to 174)	11.8 (10.8 to 12.3)	0.6 (0.5 to 0.8)	66.2 (60.6 to 71.7)	20.6 (18.5 to 24.2)	-1.6 (-3.1 to 0.2)	3.5 (1.0 to 5.6)	-2.0 (-6.3 to 2.3)	-2.5 (-4.7 to -0.2)	0.2 (-1.9 to 2.6)
**Albania**	848 (796 to 908)	1410 (1039 to 1918)	46.2 (43.4 to 47.5)	3.4 (2.0 to 5.6)	49.2 (40.1 to 60.2)	0.0 (0.0 to 0.1)	3.4 (1.2 to 5.6)	4.1 (1.7 to 6.5)	5.6 (-3.4 to 14.7)	2.6 (-0.7 to 6.3)	-39.1 (-100.0 to -3.9)
**Algeria**	1026 (998 to 1055)	1298 (919 to 1790)	69.6 (58.4 to 78.5)	1.1 (1.0 to 1.2)	28.7 (25.1 to 32.1)	0.0 (0.0 to 0.0)	1.5 (-0.8 to 3.7)	1.3 (-2.0 to 4.4)	0.4 (-1.3 to 2.3)	1.8 (0.3 to 3.2)	-27.1 (-100.0 to -0.1)
**Andorra**	9203 (8659 to 9745)	8905 (7556 to 10370)	53.1 (48.7 to 58.9)	6.9 (6.2 to 7.7)	39.7 (36.6 to 42.3)	0.0 (0.0 to 0.0)	-0.2 (-1.4 to 0.9)	-0.7 (-2.5 to 1.2)	-0.8 (-2.6 to 1.1)	0.4 (-1.2 to 2.0)	-
**Angola**	197 (177 to 216)	253 (151 to 414)	51.2 (33.3 to 67.0)	5.1 (4.1 to 5.5)	38.0 (31.1 to 47.0)	2.6 (2.4 to 2.9)	1.5 (-1.7 to 5.1)	0.4 (-5.4 to 6.0)	2.5 (-3.4 to 7.9)	2.6 (-1.9 to 7.6)	-1.2 (-3.3 to 1.4)

			Health spending per total, 2030				Per capita annualized rate of change, 2015-2030				
Location name	Total health spending per capita 2015 ($)	Total health spending per capita 2030 ($)	Government (%)	Pre-paid private (%)	Out-of-pocket (%)	Development assistance for health (%)	Total (%)	Government (%)	Pre-paid private (%)	Out-of-pocket (%)	Development assistance for health (%)
**Antigua and Barbuda**	1198 (1149 to 1251)	1820 (1153 to 2744)	64.0 (47.3 to 76.9)	13.1 (11.8 to 15.0)	21.4 (18.0 to 24.9)	0.0 (0.0 to 0.0)	2.7 (-0.2 to 5.7)	2.2 (-2.6 to 6.6)	5.5 (1.8 to 9.6)	2.1 (0.2 to 4.0)	-68.7 (-69.4 to -68.0)
**Argentina**	1457 (1393 to 1528)	1845 (1278 to 2536)	74.9 (69.9 to 80.7)	6.9 (6.5 to 7.8)	17.1 (13.3 to 22.9)	0.1 (0.0 to 0.4)	1.5 (-0.9 to 3.7)	1.9 (-1.0 to 4.7)	-1.2 (-4.2 to 2.1)	1.1 (-2.9 to 5.4)	-50.6 (-100.0 to 1.0)
**Armenia**	849 (766 to 932)	1232 (697 to 2113)	21.4 (18.1 to 23.7)	1.2 (1.0 to 1.4)	74.2 (57.5 to 85.7)	1.7 (1.5 to 2.0)	2.3 (-1.5 to 6.4)	3.6 (0.4 to 6.5)	1.2 (-4.5 to 6.4)	1.8 (-3.5 to 6.9)	3.0 (0.3 to 6.1)
**Australia**	4400 (4263 to 4559)	5437 (4913 to 6083)	67.4 (65.0 to 68.9)	13.0 (9.9 to 16.6)	19.5 (18.4 to 20.6)	0.0 (0.0 to 0.0)	1.4 (0.7 to 2.2)	1.4 (0.5 to 2.3)	1.4 (-1.1 to 4.0)	1.5 (0.0 to 2.9)	-
**Austria**	5183 (5116 to 5236)	6166 (5573 to 6918)	75.5 (73.7 to 77.7)	6.3 (5.4 to 7.2)	18.1 (17.7 to 18.5)	0.0 (0.0 to 0.0)	1.2 (0.5 to 1.9)	1.1 (0.3 to 2.1)	0.9 (-0.9 to 2.6)	1.2 (0.4 to 2.1)	-
**Azerbaijan**	1221 (1132 to 1322)	1928 (1057 to 3435)	22.5 (21.3 to 23.1)	0.5 (0.4 to 0.6)	74.9 (61.4 to 87.0)	0.3 (0.2 to 0.3)	2.8 (-0.9 to 7.2)	3.6 (-0.5 to 7.6)	0.8 (-1.8 to 3.6)	2.5 (-2.6 to 8.0)	3.1 (-0.9 to 6.7)
**Bahrain**	2470 (2363 to 2572)	2827 (1862 to 4063)	64.5 (55.2 to 72.4)	9.1 (6.6 to 14.2)	24.5 (19.3 to 30.9)	0.0 (0.0 to 0.0)	0.8 (-1.9 to 3.3)	0.6 (-3.1 to 4.1)	0.9 (-3.9 to 6.9)	0.7 (-3.5 to 5.0)	-
**Bangladesh**	90 (86 to 94)	191 (166 to 223)	18.1 (14.7 to 21.5)	2.5 (1.9 to 3.2)	76.1 (74.3 to 78.0)	3.1 (2.4 to 4.0)	5.2 (4.1 to 6.3)	6.4 (3.9 to 8.8)	4.7 (1.7 to 8.0)	5.3 (4.1 to 6.7)	-1.3 (-3.6 to 1.5)
**Barbados**	1237 (1175 to 1293)	1452 (1147 to 1777)	49.7 (40.8 to 57.2)	6.9 (6.5 to 7.6)	42.9 (42.5 to 44.0)	0.0 (0.0 to 0.0)	1.0 (-0.6 to 2.5)	1.3 (-1.5 to 3.8)	0.7 (-1.2 to 2.7)	0.7 (-1.0 to 2.3)	-
**Belarus**	1232 (1184 to 1275)	1738 (1255 to 2400)	51.3 (48.8 to 52.7)	1.9 (1.8 to 2.0)	44.8 (31.3 to 60.3)	0.9 (0.8 to 1.0)	2.2 (0.1 to 4.6)	1.0 (-1.1 to 2.9)	-0.5 (-3.1 to 2.2)	3.9 (-0.5 to 8.5)	4.2 (1.7 to 7.0)
**Belgium**	4939 (4782 to 5095)	5737 (4986 to 6604)	81.5 (79.0 to 83.3)	0.0 (0.0 to 0.1)	18.3 (16.6 to 20.1)	0.0 (0.0 to 0.0)	1.0 (0.1 to 1.9)	0.9 (-0.2 to 2.0)	5.8 (3.5 to 12.0)	1.1 (-0.7 to 3.1)	-
**Belize**	544 (519 to 572)	670 (514 to 856)	63.2 (56.3 to 69.7)	9.3 (4.8 to 15.1)	22.9 (22.3 to 23.4)	3.8 (2.9 to 5.1)	1.3 (-0.4 to 3.2)	1.1 (-1.5 to 3.6)	4.4 (-1.5 to 10.0)	1.3 (-0.6 to 3.1)	-0.2 (-3.9 to 3.3)
**Benin**	82 (79 to 85)	97 (76 to 120)	28.8 (17.9 to 41.8)	5.7 (5.6 to 6.0)	44.6 (43.8 to 45.3)	19.9 (17.6 to 23.9)	1.1 (-0.5 to 2.7)	3.1 (-1.6 to 7.5)	1.5 (-0.1 to 3.5)	1.4 (-0.2 to 3.0)	-1.7 (-3.7 to 0.7)
**Bhutan**	285 (272 to 298)	433 (250 to 678)	73.0 (63.0 to 82.3)	1.2 (1.1 to 1.3)	24.1 (23.2 to 25.3)	0.3 (0.0 to 0.7)	2.6 (-0.9 to 5.9)	2.7 (-1.7 to 6.9)	3.4 (0.0 to 6.9)	4.0 (0.6 to 7.7)	-27.3 (-100.0 to -4.5)
**Bolivia**	450 (432 to 464)	673 (498 to 875)	72.1 (64.6 to 78.1)	2.4 (1.8 to 3.2)	24.3 (24.0 to 25.0)	0.5 (0.3 to 0.7)	2.7 (0.7 to 4.5)	3.0 (0.3 to 5.4)	1.3 (-2.4 to 5.1)	2.1 (0.0 to 4.2)	-16.0 (-100.0 to -4.6)
**Bosnia and Herzegovina**	1076 (999 to 1174)	1594 (1020 to 2331)	71.2 (63.3 to 77.2)	1.4 (1.1 to 2.4)	24.7 (19.4 to 32.7)	1.1 (1.0 to 1.4)	2.5 (-0.3 to 5.4)	2.7 (-0.8 to 6.1)	4.6 (-4.1 to 13.9)	1.4 (-3.5 to 6.4)	2.2 (-2.2 to 8.4)
**Botswana**	1019 (946 to 1127)	1612 (1201 to 2149)	48.5 (41.9 to 54.1)	33.5 (24.2 to 47.1)	7.4 (4.8 to 10.6)	9.1 (8.4 to 10.5)	3.0 (1.0 to 5.2)	2.1 (-0.8 to 5.2)	3.4 (-0.7 to 8.1)	5.3 (0.5 to 10.1)	1.1 (-19.7 to 6.1)
**Brazil**	1431 (1407 to 1453)	1638 (1299 to 1966)	43.1 (32.4 to 51.0)	30.2 (24.5 to 32.3)	26.0 (25.6 to 27.2)	0.0 (0.0 to 0.1)	0.9 (-0.6 to 2.1)	0.8 (-2.5 to 3.3)	1.3 (-1.6 to 3.1)	0.3 (-1.0 to 1.6)	-1.6 (-5.6 to 1.5)
**Brunei**	2092 (1942 to 2276)	2188 (1266 to 3520)	86.6 (78.5 to 92.0)	4.5 (3.7 to 5.2)	7.9 (5.9 to 10.1)	0.0 (0.0 to 0.0)	0.1 (-3.3 to 3.6)	-0.1 (-4.2 to 3.8)	-0.5 (-2.9 to 1.8)	1.7 (-3.9 to 7.3)	-

			Health spending per total, 2030				Per capita annualized rate of change, 2015-2030				
Location name	Total health spending per capita 2015 ($)	Total health spending per capita 2030 ($)	Government (%)	Pre-paid private (%)	Out-of-pocket (%)	Development assistance for health (%)	Total (%)	Government (%)	Pre-paid private (%)	Out-of-pocket (%)	Development assistance for health (%)
**Bulgaria**	1620 (1566 to 1672)	2546 (1988 to 3188)	55.7 (46.0 to 63.5)	1.5 (1.1 to 2.2)	42.4 (41.2 to 45.1)	0.0 (0.0 to 0.0)	3.0 (1.4 to 4.6)	3.5 (0.5 to 6.1)	4.3 (0.3 to 8.9)	2.3 (1.0 to 4.0)	-42.6 (-100.0 to -3.9)
**Burkina Faso**	94 (91 to 97)	136 (105 to 174)	38.8 (32.2 to 46.3)	7.6 (4.1 to 13.5)	33.7 (26.5 to 42.5)	18.3 (16.6 to 21.3)	2.4 (0.7 to 4.2)	4.2 (1.2 to 7.3)	3.6 (-2.0 to 10.0)	1.9 (-1.3 to 5.5)	-0.1 (-2.1 to 2.4)
**Burundi**	67 (63 to 71)	79 (60 to 106)	25.5 (19.9 to 30.5)	1.1 (0.6 to 1.8)	20.8 (15.0 to 28.6)	51.1 (46.3 to 59.0)	1.1 (-0.8 to 3.1)	-0.3 (-3.8 to 3.1)	-3.0 (-9.3 to 3.1)	1.3 (-3.1 to 5.9)	1.5 (-0.5 to 4.2)
**Cambodia**	213 (199 to 229)	380 (269 to 538)	19.2 (14.8 to 22.5)	0.5 (0.4 to 0.5)	71.5 (65.8 to 78.6)	7.7 (7.4 to 8.4)	3.8 (1.4 to 6.4)	3.1 (-1.0 to 7.2)	4.5 (0.2 to 8.2)	4.9 (1.9 to 8.1)	-0.9 (-3.0 to 1.6)
**Cameroon**	156 (148 to 163)	201 (170 to 237)	18.9 (13.3 to 25.4)	3.6 (2.1 to 5.8)	67.3 (64.9 to 69.1)	9.7 (7.8 to 12.3)	1.7 (0.6 to 2.9)	3.2 (-0.4 to 6.6)	2.7 (-4.2 to 10.3)	1.5 (0.2 to 2.9)	0.5 (-1.5 to 3.0)
**Canada**	4921 (4835 to 5031)	6151 (5209 to 7194)	72.9 (68.1 to 76.6)	13.1 (11.8 to 13.9)	13.8 (13.0 to 14.6)	0.0 (0.0 to 0.0)	1.5 (0.3 to 2.6)	1.4 (-0.2 to 2.8)	2.1 (0.3 to 3.7)	1.2 (0.5 to 1.9)	-
**Cape Verde**	356 (340 to 372)	416 (283 to 596)	62.6 (48.8 to 72.9)	3.4 (3.1 to 3.6)	31.8 (27.5 to 35.1)	1.3 (0.0 to 3.1)	0.9 (-1.5 to 3.4)	1.0 (-3.0 to 4.8)	3.4 (0.1 to 6.7)	3.5 (1.6 to 5.2)	-44.8 (-100.0 to -3.5)
**Central African Republic**	28 (27 to 30)	36 (26 to 48)	13.5 (6.6 to 23.5)	4.4 (2.4 to 7.2)	28.8 (27.4 to 30.2)	51.6 (44.3 to 61.1)	1.5 (-0.7 to 3.7)	0.9 (-5.6 to 7.2)	0.9 (-5.3 to 7.0)	-1.5 (-4.0 to 1.0)	3.0 (-0.4 to 6.3)
**Chad**	103 (97 to 110)	116 (88 to 153)	22.8 (12.5 to 38.4)	4.4 (2.7 to 6.3)	60.8 (57.4 to 63.6)	10.5 (9.2 to 12.5)	0.7 (-1.2 to 2.8)	-0.8 (-6.3 to 4.9)	-1.1 (-5.8 to 3.9)	1.0 (-1.3 to 3.3)	1.2 (-1.1 to 4.4)
**Chile**	1950 (1921 to 1984)	2284 (1921 to 2732)	57.4 (52.8 to 62.0)	8.0 (4.5 to 12.8)	34.1 (32.5 to 35.6)	0.0 (0.0 to 0.0)	1.0 (-0.1 to 2.3)	0.7 (-1.0 to 2.4)	2.0 (-2.8 to 6.8)	1.3 (-0.2 to 2.9)	-71.1 (-71.7 to -70.5)
**China**	779 (765 to 794)	2051 (1640 to 2629)	65.3 (59.2 to 72.4)	5.3 (3.7 to 7.4)	28.8 (28.2 to 29.1)	0.0 (0.0 to 0.0)	6.6 (5.1 to 8.5)	7.3 (5.1 to 10.0)	3.8 (-0.2 to 8.0)	5.7 (4.1 to 7.3)	-55.5 (-100.0 to -9.3)
**Colombia**	861 (806 to 914)	1166 (867 to 1517)	71.5 (66.1 to 77.3)	12.8 (12.6 to 13.1)	14.7 (8.5 to 23.9)	0.0 (0.0 to 0.0)	2.0 (0.0 to 4.0)	2.1 (-0.4 to 4.6)	2.9 (1.0 to 4.9)	0.2 (-5.1 to 5.7)	-78.8 (-100.0 to -4.6)
**Comoros**	131 (123 to 138)	118 (97 to 145)	24.5 (15.2 to 33.6)	5.1 (4.5 to 5.7)	65.0 (64.4 to 66.1)	4.6 (1.0 to 8.3)	-0.7 (-2.0 to 0.7)	3.5 (-0.9 to 7.7)	1.5 (-0.7 to 3.6)	-1.5 (-2.7 to 0.0)	-22.1 (-100.0 to 0.1)
**Congo**	181 (171 to 194)	221 (136 to 342)	53.8 (39.7 to 67.7)	1.8 (1.7 to 1.8)	38.8 (34.8 to 44.2)	3.2 (3.0 to 3.3)	1.1 (-1.9 to 4.4)	1.9 (-3.1 to 6.8)	0.3 (-3.1 to 3.6)	0.1 (-3.7 to 4.3)	-3.0 (-8.1 to 0.9)
**Costa Rica**	1339 (1300 to 1375)	1737 (1391 to 2151)	71.2 (64.5 to 77.2)	3.6 (2.8 to 4.8)	24.5 (23.8 to 24.9)	0.3 (0.0 to 2.1)	1.7 (0.3 to 3.3)	1.3 (-0.8 to 3.4)	4.4 (1.1 to 8.1)	2.4 (1.0 to 3.9)	-50.5 (-100.0 to 18.0)
**Cote d'Ivoire**	131 (108 to 162)	193 (157 to 236)	33.0 (22.7 to 43.0)	3.1 (1.6 to 5.2)	44.4 (43.6 to 45.1)	18.5 (15.6 to 23.0)	2.6 (0.6 to 4.8)	2.4 (-2.2 to 7.2)	2.8 (-5.3 to 11.8)	2.2 (-0.2 to 4.6)	3.3 (1.3 to 5.9)
**Croatia**	1736 (1660 to 1813)	2482 (2038 to 3034)	73.6 (72.6 to 74.5)	11.4 (5.4 to 20.3)	14.3 (11.0 to 17.6)	0.1 (0.0 to 0.7)	2.4 (1.1 to 3.8)	2.0 (0.7 to 3.4)	5.3 (-1.3 to 12.2)	2.0 (-0.9 to 4.9)	-
**Cuba**	977 (870 to 1083)	1309 (999 to 1620)	91.7 (89.5 to 93.5)	1.6 (1.0 to 2.7)	6.2 (4.9 to 8.1)	0.3 (0.3 to 0.4)	1.9 (0.1 to 3.7)	1.8 (-0.2 to 3.7)	1.0 (-5.2 to 7.5)	3.9 (0.3 to 7.8)	2.1 (-0.7 to 5.6)
**Cyprus**	2821 (2504 to 3127)	3673 (2923 to 4587)	75.6 (69.7 to 80.7)	4.6 (4.2 to 5.0)	19.5 (18.7 to 20.6)	0.0 (0.0 to 0.0)	1.7 (0.0 to 3.5)	1.9 (-0.2 to 4.2)	1.8 (-0.4 to 4.1)	1.1 (-1.3 to 3.6)	-

			Health spending per total, 2030				Per capita annualized rate of change, 2015-2030				
Location name	Total health spending per capita 2015 ($)	Total health spending per capita 2030 ($)	Government (%)	Pre-paid private (%)	Out-of-pocket (%)	Development assistance for health (%)	Total (%)	Government (%)	Pre-paid private (%)	Out-of-pocket (%)	Development assistance for health (%)
**Czech Republic**	2534 (2092 to 2924)	3451 (2830 to 4155)	71.7 (68.0 to 75.2)	3.3 (1.9 to 5.7)	24.7 (23.9 to 25.5)	0.0 (0.0 to 0.0)	2.1 (0.5 to 3.7)	2.0 (0.1 to 3.9)	4.1 (-4.3 to 12.9)	2.3 (-0.8 to 5.7)	-
**Democratic Republic of the Congo**	44 (42 to 47)	50 (35 to 69)	30.2 (15.6 to 45.4)	4.8 (3.3 to 6.7)	31.3 (27.3 to 35.6)	31.7 (29.9 to 35.0)	0.7 (-1.4 to 3.2)	5.1 (-1.6 to 10.8)	-1.8 (-7.1 to 3.4)	-0.3 (-3.6 to 2.9)	-0.6 (-2.6 to 1.9)
**Denmark**	5144 (5049 to 5264)	6109 (5387 to 6875)	84.5 (82.4 to 86.2)	2.4 (2.1 to 2.8)	13.0 (12.9 to 13.1)	0.0 (0.0 to 0.0)	1.1 (0.3 to 2.0)	1.2 (0.1 to 2.2)	2.1 (0.1 to 3.7)	0.8 (-0.1 to 1.6)	-
**Djibouti**	147 (140 to 156)	192 (121 to 282)	70.7 (55.6 to 79.4)	2.0 (1.9 to 2.1)	23.4 (22.7 to 24.0)	2.6 (1.2 to 3.7)	1.6 (-1.3 to 4.5)	3.0 (-1.5 to 6.8)	3.2 (0.5 to 5.9)	2.1 (-0.9 to 5.1)	-29.0 (-100.0 to -5.1)
**Dominica**	606 (591 to 620)	750 (540 to 1018)	68.2 (57.6 to 77.0)	1.6 (0.8 to 2.5)	28.7 (26.8 to 30.8)	0.7 (0.0 to 2.0)	1.3 (-0.8 to 3.6)	1.4 (-1.8 to 4.5)	2.6 (-5.2 to 10.1)	1.2 (-0.5 to 2.9)	-16.7 (-100.0 to 4.9)
**Dominican Republic**	932 (905 to 968)	1577 (1192 to 2111)	43.8 (34.8 to 55.8)	8.1 (6.7 to 9.9)	39.4 (36.1 to 41.7)	7.5 (6.5 to 8.9)	3.5 (1.6 to 5.5)	4.0 (0.8 to 7.9)	3.3 (0.0 to 6.7)	2.8 (0.3 to 5.2)	-33.7 (-100.0 to 5.5)
**Ecuador**	1028 (992 to 1077)	1215 (919 to 1582)	55.5 (46.5 to 62.5)	5.7 (4.5 to 7.2)	37.9 (33.5 to 42.5)	0.0 (0.0 to 0.1)	1.1 (-0.8 to 2.9)	1.7 (-1.3 to 4.4)	0.5 (-2.9 to 4.3)	0.1 (-2.5 to 2.8)	-69.3 (-100.0 to -7.3)
**Egypt**	484 (460 to 505)	716 (597 to 855)	29.2 (23.4 to 34.8)	10.3 (6.9 to 15.5)	59.8 (57.4 to 61.9)	0.1 (0.1 to 0.2)	2.6 (1.4 to 3.9)	2.4 (-0.3 to 5.0)	4.5 (0.8 to 9.0)	2.4 (0.8 to 4.0)	-7.9 (-100.0 to -1.7)
**El Salvador**	598 (570 to 623)	742 (625 to 883)	65.4 (60.9 to 70.6)	8.2 (5.9 to 10.8)	24.8 (24.1 to 25.4)	1.2 (1.0 to 1.7)	1.4 (0.3 to 2.7)	1.5 (-0.1 to 3.3)	3.7 (0.4 to 7.1)	0.6 (-1.0 to 2.2)	-1.9 (-5.1 to 1.4)
**Equatorial Guinea**	1089 (988 to 1192)	1719 (1027 to 2749)	29.6 (16.8 to 42.7)	12.7 (9.5 to 16.7)	53.5 (44.0 to 65.4)	0.4 (0.0 to 1.9)	2.9 (-0.4 to 6.4)	4.8 (-2.3 to 11.3)	5.1 (-1.6 to 11.5)	1.3 (-3.3 to 6.2)	-57.7 (-100.0 to 7.6)
**Eritrea**	41 (37 to 45)	57 (40 to 79)	50.5 (40.4 to 59.8)	5.7 (4.4 to 7.6)	38.0 (31.7 to 45.4)	4.0 (2.9 to 5.1)	2.2 (-0.2 to 4.5)	7.5 (2.9 to 11.8)	4.1 (-0.1 to 8.6)	-0.3 (-3.8 to 3.3)	-23.6 (-100.0 to -5.1)
**Estonia**	1946 (1922 to 1969)	2802 (2170 to 3597)	68.6 (63.3 to 73.5)	1.4 (1.0 to 1.7)	29.5 (27.3 to 32.0)	0.0 (0.0 to 0.0)	2.4 (0.7 to 4.2)	1.8 (-0.4 to 4.0)	0.6 (-2.7 to 3.9)	4.1 (1.8 to 6.5)	-
**Ethiopia**	81 (77 to 85)	167 (122 to 228)	22.0 (13.8 to 30.9)	22.9 (12.2 to 37.6)	37.3 (32.3 to 41.0)	15.6 (14.6 to 17.3)	4.9 (2.8 to 7.2)	5.0 (-0.1 to 10.0)	7.4 (1.2 to 13.6)	5.7 (2.6 to 8.7)	0.8 (-1.2 to 3.3)
**Federated States of Micronesia**	239 (230 to 247)	159 (106 to 225)	79.4 (75.9 to 81.1)	0.5 (0.5 to 0.6)	7.5 (7.1 to 8.4)	11.0 (0.0 to 31.8)	-2.8 (-5.3 to -0.4)	1.0 (-1.6 to 3.3)	1.3 (-1.0 to 3.6)	1.0 (-0.9 to 3.2)	-32.2 (-100.0 to 0.1)
**Fiji**	342 (328 to 358)	516 (397 to 670)	57.4 (50.3 to 62.4)	14.5 (9.2 to 24.2)	26.8 (23.1 to 29.6)	0.2 (0.0 to 0.9)	2.7 (1.0 to 4.6)	2.3 (-0.3 to 4.8)	3.5 (-1.2 to 9.3)	4.6 (1.8 to 7.3)	-52.1 (-100.0 to -3.2)
**Finland**	4101 (4035 to 4163)	5280 (4471 to 6201)	78.3 (75.5 to 81.5)	3.0 (2.4 to 3.6)	18.5 (17.9 to 19.1)	0.0 (0.0 to 0.0)	1.7 (0.6 to 2.8)	1.7 (0.4 to 3.1)	2.2 (-0.5 to 4.8)	1.3 (-0.1 to 2.5)	-
**France**	4741 (4677 to 4799)	5417 (4927 to 5978)	77.6 (76.0 to 79.6)	14.7 (13.5 to 15.6)	7.6 (7.0 to 8.1)	0.0 (0.0 to 0.0)	0.9 (0.3 to 1.5)	0.8 (0.0 to 1.6)	1.1 (-0.1 to 2.2)	1.6 (0.5 to 2.8)	-
**Gabon**	487 (448 to 524)	604 (423 to 871)	57.6 (46.4 to 70.0)	12.4 (8.9 to 16.0)	28.4 (27.1 to 29.0)	0.0 (0.0 to 0.1)	1.3 (-0.9 to 4.0)	1.2 (-2.5 to 5.3)	0.7 (-3.7 to 5.1)	1.9 (-0.9 to 4.7)	-55.9 (-100.0 to -6.2)
**Georgia**	803 (754 to 860)	1195 (767 to 1800)	40.5 (28.8 to 49.9)	2.2 (1.1 to 3.9)	53.9 (44.4 to 64.7)	1.0 (0.9 to 1.1)	2.5 (-0.3 to 5.6)	2.9 (-2.2 to 7.6)	4.5 (-4.7 to 14.8)	2.1 (-2.3 to 6.5)	-3.4 (-8.2 to 0.2)

			Health spending per total, 2030				Per capita annualized rate of change, 2015-2030				
Location name	Total health spending per capita 2015 ($)	Total health spending per capita 2030 ($)	Government (%)	Pre-paid private (%)	Out-of-pocket (%)	Development assistance for health (%)	Total (%)	Government (%)	Pre-paid private (%)	Out-of-pocket (%)	Development assistance for health (%)
**Germany**	5532 (5366 to 5764)	6323 (5532 to 7198)	83.4 (81.5 to 85.5)	1.9 (1.1 to 2.9)	14.6 (14.2 to 15.0)	0.0 (0.0 to 0.0)	0.9 (0.0 to 1.8)	0.8 (-0.2 to 1.9)	-2.6 (-8.9 to 3.2)	1.9 (0.8 to 3.0)	-
**Ghana**	242 (234 to 250)	414 (298 to 576)	49.5 (33.1 to 64.1)	2.9 (1.9 to 4.0)	36.6 (29.1 to 43.7)	10.1 (9.6 to 11.1)	3.6 (1.3 to 5.9)	5.3 (0.4 to 9.6)	1.7 (-3.4 to 6.5)	2.8 (1.8 to 3.6)	0.7 (-1.5 to 3.2)
**Greece**	2352 (2181 to 2515)	2728 (2389 to 3158)	66.1 (64.1 to 68.2)	3.7 (3.3 to 3.8)	30.0 (26.7 to 33.9)	0.0 (0.0 to 0.0)	1.0 (0.0 to 2.1)	1.3 (0.1 to 2.7)	1.4 (-0.3 to 2.7)	0.2 (-2.0 to 2.5)	-
**Grenada**	715 (671 to 773)	985 (739 to 1259)	37.4 (28.1 to 46.5)	7.7 (5.1 to 11.6)	53.1 (51.4 to 56.8)	0.7 (0.0 to 1.8)	2.1 (0.1 to 3.9)	1.9 (-2.1 to 5.1)	5.1 (-1.0 to 12.4)	1.9 (-0.2 to 4.1)	-44.2 (-100.0 to 4.9)
**Guatemala**	487 (459 to 514)	594 (496 to 707)	33.7 (30.8 to 37.5)	10.8 (7.5 to 14.6)	53.9 (49.0 to 59.1)	1.1 (0.5 to 1.5)	1.3 (0.1 to 2.6)	1.7 (-0.1 to 3.7)	5.1 (1.5 to 8.7)	1.5 (-0.5 to 3.5)	-9.5 (-13.2 to -6.5)
**Guinea**	102 (99 to 104)	114 (92 to 143)	21.4 (13.5 to 31.0)	4.4 (2.3 to 5.7)	44.9 (44.1 to 45.4)	28.2 (22.2 to 37.6)	0.7 (-0.7 to 2.3)	4.7 (0.1 to 9.3)	5.3 (-0.5 to 8.9)	1.3 (0.0 to 2.8)	-0.6 (-2.8 to 2.2)
**Guinea-Bissau**	121 (117 to 129)	124 (93 to 176)	25.0 (12.3 to 47.0)	2.4 (1.6 to 3.6)	36.3 (33.2 to 37.2)	34.6 (31.4 to 37.7)	0.1 (-1.8 to 2.5)	-0.2 (-6.4 to 6.9)	2.5 (-4.8 to 9.7)	0.8 (-1.0 to 2.7)	-0.4 (-2.5 to 2.2)
**Guyana**	318 (298 to 335)	486 (308 to 730)	48.0 (41.3 to 52.8)	0.1 (0.1 to 0.2)	48.6 (37.6 to 61.0)	1.2 (0.5 to 2.0)	2.7 (-0.2 to 5.7)	2.0 (-1.8 to 5.7)	5.9 (3.0 to 11.4)	4.1 (-0.5 to 8.7)	-19.4 (-100.0 to -1.4)
**Haiti**	135 (130 to 140)	156 (122 to 202)	11.2 (6.8 to 15.2)	4.0 (2.2 to 6.7)	29.6 (27.1 to 30.6)	54.2 (47.9 to 63.6)	0.9 (-0.7 to 2.7)	1.7 (-3.1 to 5.7)	0.6 (-5.5 to 6.8)	0.1 (-2.3 to 2.2)	1.5 (-0.6 to 4.0)
**Honduras**	370 (351 to 397)	491 (365 to 642)	44.2 (38.3 to 50.1)	5.9 (4.9 to 7.5)	47.1 (40.4 to 54.6)	1.7 (1.6 to 2.0)	1.8 (-0.1 to 3.8)	2.5 (-0.6 to 5.4)	2.8 (-0.3 to 6.5)	1.3 (-1.6 to 4.3)	-4.0 (-6.5 to -1.2)
**Hungary**	2031 (1969 to 2100)	2926 (2352 to 3646)	61.6 (60.7 to 62.2)	4.0 (2.8 to 5.4)	33.7 (24.2 to 45.6)	0.0 (0.0 to 0.0)	2.4 (1.0 to 4.0)	1.9 (0.3 to 3.5)	1.9 (-2.0 to 5.7)	3.4 (-0.2 to 7.2)	-
**Iceland**	4205 (4085 to 4323)	5861 (4933 to 6826)	80.5 (76.8 to 83.1)	3.3 (2.9 to 3.9)	15.9 (14.8 to 17.5)	0.0 (0.0 to 0.0)	2.2 (1.1 to 3.3)	2.3 (0.8 to 3.5)	2.0 (0.0 to 4.2)	1.9 (0.2 to 3.7)	-
**India**	236 (233 to 239)	502 (435 to 584)	28.8 (26.1 to 31.3)	9.9 (8.0 to 11.3)	60.7 (57.7 to 64.4)	0.3 (0.3 to 0.4)	5.1 (4.1 to 6.2)	5.8 (4.2 to 7.6)	6.2 (3.8 to 8.4)	4.7 (3.4 to 6.2)	-2.3 (-4.4 to 0.2)
**Indonesia**	383 (365 to 398)	770 (564 to 1087)	38.4 (36.1 to 39.0)	9.3 (7.9 to 10.3)	51.1 (40.3 to 63.9)	0.2 (0.2 to 0.3)	4.7 (2.6 to 7.2)	4.7 (2.6 to 6.9)	2.4 (1.0 to 3.8)	5.1 (1.4 to 9.3)	-2.9 (-5.5 to -0.3)
**Iran**	1232 (1171 to 1295)	1926 (1330 to 2759)	42.8 (33.3 to 50.6)	9.6 (5.8 to 14.1)	45.5 (37.2 to 54.3)	0.0 (0.0 to 0.0)	2.9 (0.5 to 5.5)	2.0 (-2.0 to 6.0)	4.4 (-1.1 to 10.3)	3.2 (-0.5 to 7.1)	-92.2 (-100.0 to -10.0)
**Iraq**	562 (502 to 644)	957 (575 to 1453)	43.8 (33.0 to 56.4)	0.1 (0.1 to 0.1)	53.8 (49.7 to 61.8)	0.1 (0.1 to 0.1)	3.4 (0.1 to 6.6)	3.9 (-1.3 to 8.9)	10.6 (4.6 to 16.5)	2.8 (-1.3 to 6.9)	-4.7 (-8.1 to -1.0)
**Ireland**	5371 (5146 to 5576)	7603 (5866 to 9702)	68.5 (60.3 to 74.7)	12.3 (10.1 to 14.7)	18.7 (18.1 to 20.1)	0.0 (0.0 to 0.0)	2.3 (0.6 to 4.0)	1.9 (-0.6 to 4.1)	2.0 (-1.1 to 5.0)	4.0 (2.5 to 5.5)	-
**Israel**	2560 (2417 to 2745)	3183 (2784 to 3582)	61.8 (59.3 to 64.3)	16.2 (12.1 to 21.5)	21.7 (21.3 to 22.0)	0.0 (0.0 to 0.0)	1.5 (0.4 to 2.4)	1.1 (-0.2 to 2.3)	3.8 (0.8 to 6.8)	1.0 (-0.6 to 2.7)	-
**Italy**	3445 (3357 to 3526)	3984 (3523 to 4449)	77.6 (75.8 to 79.3)	2.9 (2.4 to 3.3)	19.4 (17.8 to 21.3)	0.0 (0.0 to 0.0)	1.0 (0.2 to 1.7)	1.2 (0.2 to 2.1)	2.9 (0.7 to 4.8)	-0.1 (-1.5 to 1.3)	-

			Health spending per total, 2030				Per capita annualized rate of change, 2015-2030				
Location name	Total health spending per capita 2015 ($)	Total health spending per capita 2030 ($)	Government (%)	Pre-paid private (%)	Out-of-pocket (%)	Development assistance for health (%)	Total (%)	Government (%)	Pre-paid private (%)	Out-of-pocket (%)	Development assistance for health (%)
**Jamaica**	510 (479 to 542)	513 (381 to 675)	53.0 (41.8 to 63.9)	18.7 (17.9 to 19.8)	27.0 (26.2 to 27.9)	0.4 (0.0 to 0.6)	0.0 (-2.0 to 1.9)	-0.6 (-4.2 to 2.5)	1.1 (-1.1 to 3.6)	0.5 (-1.8 to 2.6)	-42.4 (-100.0 to -3.6)
**Japan**	4286 (4163 to 4465)	4596 (3897 to 5431)	83.9 (81.2 to 86.3)	0.0 (0.0 to 0.0)	16.0 (15.7 to 16.1)	0.0 (0.0 to 0.0)	0.4 (-0.7 to 1.6)	0.2 (-1.1 to 1.5)	9.1 (4.2 to 15.7)	1.7 (0.6 to 2.8)	-
**Jordan**	730 (687 to 774)	984 (708 to 1297)	62.9 (57.2 to 67.0)	9.2 (5.2 to 15.8)	25.9 (18.4 to 37.4)	0.5 (0.3 to 0.6)	1.9 (-0.2 to 3.9)	1.8 (-1.0 to 4.2)	0.7 (-4.8 to 6.6)	2.4 (-2.0 to 7.1)	-5.3 (-10.9 to -1.7)
**Kazakhstan**	1017 (997 to 1040)	1574 (1100 to 2142)	59.2 (52.3 to 67.7)	0.6 (0.5 to 0.7)	39.0 (32.8 to 41.4)	0.2 (0.0 to 0.4)	2.9 (0.5 to 5.1)	2.6 (-0.6 to 5.7)	1.6 (-0.2 to 3.1)	3.2 (-0.3 to 5.8)	-18.4 (-100.0 to 3.6)
**Kenya**	187 (185 to 190)	251 (221 to 286)	30.2 (27.3 to 32.7)	15.3 (13.6 to 17.1)	30.4 (29.8 to 30.9)	23.7 (18.6 to 31.4)	2.0 (1.1 to 2.9)	1.9 (0.3 to 3.4)	3.1 (1.4 to 4.8)	2.0 (1.0 to 3.0)	1.5 (-0.5 to 4.0)
**Kiribati**	189 (171 to 212)	296 (243 to 369)	69.1 (66.0 to 71.3)	0.0 (0.0 to 0.1)	3.5 (2.4 to 4.7)	26.9 (17.7 to 37.2)	3.0 (1.4 to 4.7)	2.3 (0.8 to 3.9)	3.5 (0.3 to 8.8)	1.1 (-3.8 to 6.3)	4.3 (0.0 to 7.9)
**Kuwait**	2640 (2425 to 2869)	2821 (1390 to 4546)	74.3 (55.5 to 84.4)	1.5 (1.5 to 1.6)	22.4 (21.5 to 25.0)	0.0 (0.0 to 0.0)	0.2 (-4.3 to 3.8)	-0.6 (-6.7 to 3.8)	-0.5 (-4.8 to 3.6)	2.8 (-1.0 to 6.6)	-
**Kyrgyzstan**	308 (293 to 331)	412 (278 to 594)	41.2 (32.5 to 49.1)	1.3 (0.3 to 3.2)	52.6 (47.0 to 59.2)	3.2 (3.1 to 3.5)	1.8 (-0.6 to 4.4)	1.4 (-2.5 to 5.5)	2.7 (-13.2 to 22.8)	2.6 (-0.7 to 6.1)	-4.4 (-7.2 to -1.5)
**Laos**	178 (167 to 195)	318 (234 to 419)	45.5 (40.5 to 50.3)	4.0 (2.1 to 7.4)	39.1 (29.5 to 49.9)	9.9 (9.2 to 11.3)	3.9 (1.7 to 6.0)	5.9 (2.8 to 8.9)	6.4 (-0.8 to 14.2)	2.9 (-1.1 to 6.7)	0.2 (-1.9 to 2.7)
**Latvia**	1683 (1593 to 1771)	2610 (2071 to 3289)	58.2 (55.5 to 62.6)	1.0 (0.4 to 1.9)	40.2 (33.0 to 46.9)	0.0 (0.0 to 0.0)	2.9 (1.3 to 4.7)	2.5 (0.6 to 4.7)	3.0 (-4.9 to 12.0)	3.4 (0.4 to 6.3)	-
**Lebanon**	1207 (1102 to 1312)	1331 (831 to 2080)	50.8 (30.8 to 66.9)	16.2 (15.9 to 17.0)	30.3 (28.8 to 31.5)	0.4 (0.1 to 0.4)	0.5 (-2.5 to 3.7)	0.3 (-5.7 to 5.7)	0.4 (-2.4 to 3.7)	0.1 (-3.2 to 3.7)	-4.2 (-100.0 to 3.3)
**Lesotho**	262 (254 to 270)	467 (371 to 594)	48.3 (41.6 to 52.6)	1.1 (0.9 to 1.4)	13.0 (12.0 to 13.7)	36.9 (32.2 to 44.4)	3.9 (2.4 to 5.6)	3.2 (0.7 to 5.6)	-0.5 (-3.9 to 2.9)	2.0 (0.8 to 3.2)	4.9 (2.8 to 7.7)
**Liberia**	481 (474 to 488)	265 (191 to 380)	5.9 (3.2 to 9.0)	0.7 (0.3 to 1.3)	13.3 (10.5 to 15.2)	79.0 (75.0 to 84.9)	-4.0 (-6.0 to -1.6)	2.0 (-4.2 to 8.4)	2.4 (-6.4 to 11.6)	1.7 (-2.0 to 5.6)	-2.0 (-3.9 to 0.8)
**Libya**	502 (435 to 582)	597 (413 to 837)	60.7 (53.4 to 69.3)	13.8 (9.0 to 17.5)	23.2 (17.0 to 33.5)	0.3 (0.2 to 0.4)	1.1 (-1.6 to 3.7)	2.2 (-1.0 to 5.7)	3.6 (-2.0 to 8.9)	-2.4 (-7.3 to 2.7)	4.1 (-0.7 to 7.8)
**Lithuania**	1941 (1872 to 2010)	3184 (2514 to 3954)	61.1 (54.7 to 67.2)	0.8 (0.5 to 1.2)	37.7 (37.2 to 37.9)	0.0 (0.0 to 0.0)	3.3 (1.7 to 4.9)	2.7 (0.4 to 5.0)	2.0 (-2.4 to 6.3)	4.4 (2.7 to 6.0)	-
**Luxembourg**	6530 (6288 to 6784)	9624 (7823 to 11651)	80.4 (77.3 to 83.2)	6.7 (5.2 to 8.9)	12.6 (11.0 to 14.6)	0.0 (0.0 to 0.0)	2.6 (1.2 to 3.9)	2.3 (0.8 to 3.9)	3.6 (0.6 to 6.8)	3.7 (1.2 to 6.4)	-
**Macedonia**	921 (758 to 1196)	1115 (818 to 1496)	56.3 (53.5 to 58.8)	3.9 (2.2 to 6.2)	38.6 (29.9 to 49.6)	0.0 (0.0 to 0.1)	1.3 (-1.3 to 3.7)	0.7 (-2.6 to 3.9)	4.2 (-4.5 to 13.3)	1.9 (-1.8 to 6.0)	-65.6 (-100.0 to -5.5)
**Madagascar**	78 (74 to 81)	100 (70 to 136)	56.2 (41.6 to 67.9)	5.7 (4.8 to 6.8)	20.9 (20.2 to 21.8)	16.0 (15.1 to 17.5)	1.6 (-0.7 to 3.8)	3.4 (-0.9 to 7.1)	0.7 (-2.6 to 4.0)	1.1 (-0.8 to 3.1)	-1.8 (-3.8 to 0.7)
**Malawi**	135 (132 to 138)	156 (116 to 215)	17.6 (10.4 to 24.3)	5.8 (3.0 to 9.4)	7.3 (4.9 to 9.8)	67.8 (63.8 to 73.6)	0.9 (-1.1 to 3.2)	0.1 (-4.9 to 4.6)	2.1 (-4.2 to 8.6)	-0.1 (-4.6 to 4.5)	1.1 (-0.9 to 3.5)

			Health spending per total, 2030				Per capita annualized rate of change, 2015-2030				
Location name	Total health spending per capita 2015 ($)	Total health spending per capita 2030 ($)	Government (%)	Pre-paid private (%)	Out-of-pocket (%)	Development assistance for health (%)	Total (%)	Government (%)	Pre-paid private (%)	Out-of-pocket (%)	Development assistance for health (%)
**Malaysia**	1072 (1041 to 1105)	1874 (1541 to 2241)	47.4 (42.1 to 51.2)	9.2 (9.0 to 9.5)	42.9 (39.3 to 49.3)	0.0 (0.0 to 0.0)	3.8 (2.5 to 5.1)	3.0 (0.9 to 4.9)	2.7 (1.2 to 4.2)	4.9 (2.9 to 7.1)	-61.0 (-100.0 to -4.0)
**Maldives**	1850 (1719 to 1990)	2223 (1542 to 3001)	80.4 (74.5 to 83.8)	1.3 (0.8 to 2.0)	17.1 (11.6 to 25.0)	0.2 (0.0 to 0.4)	1.2 (-1.2 to 3.4)	1.2 (-1.7 to 3.8)	-0.6 (-6.5 to 5.6)	0.7 (-4.1 to 5.8)	-24.0 (-100.0 to 7.3)
**Mali**	110 (105 to 115)	159 (125 to 197)	21.4 (12.7 to 31.0)	7.7 (4.1 to 10.8)	41.7 (39.9 to 44.1)	27.9 (24.5 to 34.5)	2.5 (0.9 to 4.1)	4.4 (-0.7 to 9.2)	5.7 (-0.1 to 10.2)	1.6 (-0.4 to 3.7)	1.4 (-0.6 to 4.1)
**Malta**	3642 (3494 to 3766)	5909 (5080 to 6847)	62.9 (61.3 to 64.2)	2.4 (2.0 to 2.9)	34.4 (30.3 to 39.6)	0.0 (0.0 to 0.0)	3.3 (2.3 to 4.3)	3.5 (2.3 to 4.7)	4.3 (2.2 to 6.8)	2.7 (0.7 to 4.9)	-
**Marshall Islands**	604 (565 to 646)	578 (326 to 891)	72.6 (55.8 to 82.9)	3.4 (3.0 to 3.9)	20.8 (18.5 to 25.2)	1.7 (0.0 to 4.3)	-0.5 (-4.0 to 2.7)	0.2 (-4.9 to 4.5)	-0.3 (-3.2 to 2.3)	2.7 (0.3 to 5.2)	-20.9 (-100.0 to -2.9)
**Mauritania**	184 (174 to 194)	228 (160 to 322)	43.3 (31.8 to 55.4)	5.3 (3.0 to 7.5)	46.3 (41.8 to 51.3)	3.4 (3.1 to 3.9)	1.3 (-1.0 to 3.7)	2.0 (-2.3 to 6.3)	2.8 (-3.4 to 7.9)	1.0 (-2.0 to 4.2)	-4.6 (-9.2 to -1.2)
**Mauritius**	1094 (1047 to 1137)	2088 (1463 to 2968)	38.2 (36.3 to 39.5)	0.7 (0.6 to 0.7)	60.2 (47.5 to 70.6)	0.0 (0.0 to 0.1)	4.3 (1.9 to 6.8)	3.0 (0.9 to 5.1)	2.6 (0.7 to 4.7)	5.2 (1.3 to 8.9)	-53.9 (-100.0 to 4.8)
**Mexico**	1081 (1050 to 1112)	1548 (1304 to 1819)	49.6 (49.0 to 49.9)	9.2 (6.9 to 11.9)	40.7 (34.5 to 48.1)	0.0 (0.0 to 0.1)	2.4 (1.3 to 3.6)	2.1 (0.9 to 3.3)	4.8 (1.8 to 7.7)	2.3 (0.0 to 4.7)	-22.2 (-100.0 to -2.7)
**Moldova**	543 (516 to 574)	713 (504 to 1003)	48.4 (43.6 to 50.4)	0.8 (0.6 to 1.1)	43.5 (31.4 to 56.2)	5.8 (5.3 to 6.5)	1.7 (-0.5 to 4.2)	2.0 (-1.0 to 4.7)	0.9 (-3.6 to 5.2)	1.4 (-3.1 to 5.7)	1.2 (-1.5 to 4.1)
**Mongolia**	496 (475 to 522)	938 (605 to 1421)	43.5 (37.9 to 47.2)	2.5 (2.3 to 2.7)	47.6 (36.0 to 60.1)	4.4 (4.3 to 4.6)	4.2 (1.2 to 7.3)	3.0 (-0.9 to 6.4)	2.3 (-1.0 to 6.1)	5.6 (0.8 to 10.5)	1.2 (-1.2 to 3.8)
**Montenegro**	985 (954 to 1017)	1269 (1100 to 1456)	63.9 (62.8 to 64.7)	0.5 (0.3 to 0.7)	34.5 (30.7 to 38.8)	0.9 (0.5 to 1.5)	1.7 (0.7 to 2.6)	1.4 (0.3 to 2.4)	1.7 (-3.5 to 7.8)	2.1 (0.2 to 4.0)	3.3 (-2.1 to 9.1)
**Morocco**	454 (438 to 472)	787 (631 to 979)	42.9 (38.6 to 47.9)	1.9 (1.4 to 2.4)	51.2 (46.2 to 55.6)	3.4 (2.8 to 4.4)	3.7 (2.1 to 5.3)	3.7 (1.3 to 6.1)	0.5 (-3.0 to 4.3)	3.5 (1.3 to 5.6)	9.2 (7.0 to 11.9)
**Mozambique**	72 (71 to 74)	127 (94 to 173)	24.2 (16.1 to 31.3)	3.7 (3.4 to 3.9)	8.8 (8.2 to 9.2)	62.2 (57.9 to 68.1)	3.8 (1.8 to 6.1)	7.2 (2.2 to 12.0)	3.8 (1.3 to 6.4)	5.9 (3.5 to 8.7)	2.0 (0.0 to 4.5)
**Myanmar**	301 (270 to 339)	703 (515 to 948)	25.7 (24.8 to 26.3)	1.4 (1.3 to 1.4)	67.1 (58.5 to 76.3)	5.0 (4.7 to 5.7)	5.8 (3.5 to 8.2)	6.9 (4.4 to 9.6)	5.2 (2.6 to 7.8)	5.3 (2.1 to 8.8)	4.7 (2.5 to 7.5)
**Namibia**	1033 (991 to 1084)	1180 (930 to 1466)	62.0 (57.9 to 66.5)	16.7 (11.4 to 23.0)	11.3 (7.5 to 17.5)	9.0 (7.8 to 11.2)	0.8 (-0.7 to 2.4)	0.7 (-1.3 to 2.8)	-0.6 (-4.6 to 3.3)	2.6 (-1.8 to 7.5)	1.2 (-1.3 to 4.1)
**Nepal**	160 (153 to 167)	224 (157 to 324)	18.8 (17.6 to 19.4)	14.8 (12.7 to 16.9)	57.1 (42.9 to 69.2)	7.9 (7.6 to 8.3)	2.2 (0.0 to 4.8)	2.8 (0.0 to 5.6)	4.4 (0.8 to 8.2)	2.1 (-2.0 to 6.1)	-1.2 (-3.3 to 1.3)
**Netherlands**	5579 (5360 to 5835)	6446 (5469 to 7630)	84.8 (83.9 to 85.7)	4.0 (3.3 to 4.8)	10.9 (6.4 to 16.8)	0.0 (0.0 to 0.0)	0.9 (-0.2 to 2.1)	1.3 (0.1 to 2.5)	-2.8 (-5.3 to -0.5)	0.0 (-4.2 to 4.4)	-
**New Zealand**	3648 (3481 to 3856)	4395 (3868 to 5009)	78.7 (76.3 to 81.0)	8.1 (7.1 to 9.1)	13.0 (12.8 to 13.4)	0.0 (0.0 to 0.0)	1.2 (0.3 to 2.2)	1.1 (-0.1 to 2.3)	1.9 (0.0 to 3.9)	1.5 (0.4 to 2.7)	-
**Nicaragua**	432 (413 to 454)	540 (428 to 680)	53.4 (45.0 to 60.5)	2.4 (1.7 to 3.1)	36.5 (36.2 to 36.9)	7.1 (6.0 to 8.7)	1.5 (-0.1 to 3.2)	1.3 (-1.3 to 4.0)	1.8 (-2.1 to 5.5)	1.9 (0.3 to 3.4)	0.0 (-2.1 to 2.6)

			Health spending per total, 2030				Per capita annualized rate of change, 2015-2030				
Location name	Total health spending per capita 2015 ($)	Total health spending per capita 2030 ($)	Government (%)	Pre-paid private (%)	Out-of-pocket (%)	Development assistance for health (%)	Total (%)	Government (%)	Pre-paid private (%)	Out-of-pocket (%)	Development assistance for health (%)
**Niger**	67 (65 to 69)	85 (68 to 104)	31.1 (22.5 to 38.7)	1.4 (1.1 to 1.8)	51.4 (49.1 to 54.6)	15.3 (13.2 to 18.9)	1.5 (0.1 to 3.0)	2.9 (-0.6 to 5.9)	1.6 (-1.4 to 4.6)	1.1 (-0.6 to 2.9)	0.0 (-2.1 to 2.5)
**Nigeria**	216 (201 to 234)	299 (212 to 412)	13.3 (4.5 to 26.8)	1.1 (1.0 to 1.1)	77.8 (74.8 to 80.1)	6.6 (6.2 to 7.3)	2.1 (-0.2 to 4.4)	0.3 (-8.3 to 8.2)	-1.3 (-3.9 to 1.3)	2.5 (-0.1 to 5.1)	0.7 (-1.3 to 3.4)
**North Korea**	134 (128 to 139)	119 (111 to 127)	41.4 (40.9 to 41.7)	3.7 (2.6 to 4.9)	53.5 (51.4 to 55.9)	1.4 (1.0 to 2.0)	-0.8 (-1.3 to -0.3)	-0.3 (-1.0 to 0.4)	-3.7 (-7.0 to -0.3)	-1.0 (-1.8 to -0.2)	4.0 (1.8 to 6.5)
**Norway**	7024 (6810 to 7268)	7884 (5729 to 9986)	85.1 (79.7 to 88.6)	0.4 (0.4 to 0.5)	14.3 (13.4 to 15.8)	0.0 (0.0 to 0.0)	0.7 (-1.4 to 2.5)	0.7 (-1.7 to 2.7)	1.0 (-0.2 to 2.1)	0.7 (-0.7 to 2.0)	-
**Oman**	1684 (1555 to 1799)	2166 (1362 to 3247)	82.1 (74.8 to 88.4)	4.2 (3.8 to 4.6)	12.6 (10.4 to 14.5)	0.0 (0.0 to 0.0)	1.5 (-1.6 to 4.6)	1.0 (-2.6 to 4.5)	0.1 (-3.6 to 3.7)	6.3 (1.9 to 10.5)	-
**Pakistan**	142 (136 to 150)	222 (166 to 290)	31.6 (23.0 to 41.3)	2.0 (2.0 to 2.1)	59.5 (55.6 to 63.4)	5.8 (5.4 to 6.7)	2.9 (1.0 to 4.9)	4.1 (0.1 to 8.0)	2.2 (0.5 to 4.0)	2.4 (0.0 to 4.7)	2.0 (-0.2 to 4.6)
**Palestine**	390 (345 to 435)	527 (426 to 642)	42.6 (40.6 to 46.0)	18.6 (13.9 to 24.0)	38.0 (31.9 to 43.2)	0.1 (0.0 to 0.2)	2.0 (0.5 to 3.7)	2.5 (0.7 to 4.7)	1.8 (-1.9 to 5.5)	1.6 (-1.2 to 4.3)	-45.6 (-100.0 to -6.3)
**Panama**	1588 (1535 to 1649)	2732 (2198 to 3372)	59.7 (52.3 to 66.3)	8.9 (7.3 to 10.5)	30.9 (30.4 to 31.4)	0.0 (0.0 to 0.0)	3.6 (2.2 to 5.2)	3.4 (1.1 to 5.7)	5.4 (2.6 to 8.2)	3.7 (2.3 to 5.2)	-37.7 (-100.0 to -7.7)
**Papua New Guinea**	121 (114 to 131)	127 (92 to 169)	84.6 (79.1 to 88.4)	0.0 (0.0 to 0.0)	7.5 (5.9 to 9.8)	7.3 (6.0 to 8.7)	0.3 (-1.9 to 2.3)	1.1 (-1.6 to 3.5)	18.4 (11.6 to 40.7)	2.2 (-1.9 to 6.1)	-5.8 (-9.1 to -2.9)
**Paraguay**	738 (706 to 777)	1274 (978 to 1583)	56.5 (49.4 to 64.5)	10.5 (6.2 to 17.7)	32.1 (31.7 to 32.7)	0.0 (0.0 to 0.1)	3.7 (1.9 to 5.3)	4.0 (1.3 to 6.6)	4.0 (-1.2 to 9.7)	2.8 (1.1 to 4.5)	-53.4 (-100.0 to -4.9)
**Peru**	683 (669 to 698)	993 (778 to 1256)	61.5 (56.0 to 68.7)	6.0 (4.7 to 7.4)	31.9 (30.4 to 32.8)	0.1 (0.0 to 0.2)	2.5 (0.9 to 4.1)	2.7 (0.4 to 5.1)	1.7 (-1.5 to 4.8)	2.7 (0.7 to 4.6)	-41.1 (-100.0 to -10.4)
**Philippines**	333 (324 to 347)	652 (525 to 792)	25.8 (22.4 to 29.2)	16.8 (14.7 to 19.4)	56.3 (50.5 to 62.3)	0.5 (0.5 to 0.7)	4.6 (3.1 to 6.0)	3.6 (1.2 to 5.9)	5.5 (3.1 to 7.9)	4.9 (2.6 to 7.1)	-4.1 (-6.2 to -1.6)
**Poland**	1757 (1671 to 1837)	2709 (2375 to 3111)	67.7 (64.4 to 70.8)	7.5 (5.4 to 9.7)	24.7 (24.3 to 25.0)	0.0 (0.0 to 0.0)	2.9 (2.0 to 3.9)	2.6 (1.2 to 3.9)	5.8 (1.8 to 9.8)	3.2 (2.3 to 4.1)	-
**Portugal**	2712 (2621 to 2819)	3753 (3263 to 4287)	64.5 (60.9 to 67.2)	9.4 (7.2 to 11.7)	25.8 (23.8 to 28.2)	0.0 (0.0 to 0.0)	2.2 (1.2 to 3.1)	2.0 (0.6 to 3.3)	5.0 (2.2 to 7.9)	1.7 (0.2 to 3.3)	-
**Qatar**	3251 (3050 to 3450)	4138 (2197 to 7341)	72.4 (58.7 to 85.2)	8.6 (7.7 to 8.9)	16.4 (13.5 to 19.2)	0.0 (0.0 to 0.0)	1.3 (-2.5 to 5.6)	0.3 (-4.8 to 5.8)	1.2 (-2.9 to 5.0)	7.2 (0.9 to 13.5)	-
**Romania**	1128 (1051 to 1198)	2112 (1530 to 2844)	77.9 (72.0 to 83.0)	0.6 (0.6 to 0.6)	21.0 (19.8 to 21.8)	0.0 (0.0 to 0.0)	4.2 (2.0 to 6.4)	4.2 (1.4 to 6.9)	3.3 (0.3 to 6.3)	4.1 (1.7 to 6.7)	-59.1 (-100.0 to 3.8)
**Russia**	1544 (1523 to 1564)	1988 (1503 to 2615)	54.6 (46.4 to 62.1)	1.7 (1.3 to 2.1)	42.8 (38.5 to 46.7)	0.0 (0.0 to 0.0)	1.6 (-0.2 to 3.5)	0.8 (-2.1 to 3.6)	-1.5 (-5.1 to 2.1)	2.9 (0.3 to 5.4)	-26.5 (-100.0 to -2.5)
**Rwanda**	149 (143 to 155)	239 (191 to 291)	27.6 (21.1 to 34.4)	9.0 (5.1 to 14.4)	35.8 (30.6 to 42.2)	26.3 (22.8 to 32.9)	3.2 (1.6 to 4.6)	4.0 (0.4 to 7.1)	3.3 (-1.9 to 8.5)	5.4 (2.8 to 8.1)	0.7 (-1.5 to 3.3)
**Saint Lucia**	714 (658 to 793)	957 (745 to 1240)	34.5 (24.8 to 45.2)	4.1 (3.5 to 4.6)	52.2 (48.8 to 53.9)	8.2 (6.6 to 10.2)	1.9 (0.2 to 3.7)	0.9 (-2.9 to 4.6)	1.3 (-1.3 to 3.7)	2.1 (-0.2 to 4.3)	2.7 (-6.4 to 7.8)

Location name	Total health spending per capita 2015 ($)	Total health spending per capita 2030 ($)	Health spending per total, 2030				Per capita annualized rate of change, 2015-2030				
			Government (%)	Pre-paid private (%)	Out-of-pocket (%)	Development assistance for health (%)	Total (%)	Government (%)	Pre-paid private (%)	Out-of-pocket (%)	Development assistance for health (%)
**Saint Vincent and the Grenadines**	523 (506 to 537)	693 (532 to 902)	61.5 (54.0 to 69.0)	2.4 (2.2 to 2.6)	18.2 (17.9 to 18.5)	17.2 (13.8 to 21.8)	1.8 (0.1 to 3.7)	1.4 (-1.2 to 4.0)	2.5 (-0.1 to 5.1)	1.5 (-0.6 to 3.5)	4.1 (1.0 to 7.3)
**Samoa**	342 (319 to 364)	464 (280 to 763)	57.0 (36.3 to 74.8)	0.8 (0.7 to 0.9)	10.6 (8.7 to 12.3)	29.6 (27.4 to 32.0)	1.9 (-1.4 to 5.5)	0.6 (-5.3 to 6.0)	2.2 (-0.6 to 4.9)	2.0 (-0.2 to 4.3)	3.7 (1.5 to 6.5)
**Sao Tome and Principe**	216 (206 to 225)	253 (158 to 402)	49.9 (33.3 to 67.2)	1.9 (1.0 to 2.2)	19.3 (15.2 to 23.3)	26.0 (24.5 to 27.3)	0.9 (-2.0 to 4.2)	1.1 (-4.4 to 6.6)	0.5 (-6.9 to 5.9)	1.5 (-3.0 to 6.2)	-0.4 (-4.2 to 2.7)
**Saudi Arabia**	3138 (2975 to 3318)	3913 (2364 to 6183)	68.6 (54.7 to 80.4)	14.4 (12.9 to 15.6)	15.1 (14.2 to 15.9)	0.0 (0.0 to 0.0)	1.3 (-1.9 to 4.7)	1.0 (-3.6 to 5.4)	1.8 (-1.9 to 5.5)	1.4 (-2.1 to 4.9)	-
**Senegal**	119 (113 to 123)	147 (124 to 172)	35.7 (29.6 to 41.5)	12.7 (8.3 to 18.5)	37.4 (36.5 to 38.1)	13.5 (11.0 to 17.4)	1.4 (0.3 to 2.6)	3.3 (0.8 to 5.5)	2.5 (-1.4 to 6.4)	1.5 (0.1 to 3.1)	-2.5 (-4.5 to - 0.1)
**Serbia**	1398 (1349 to 1459)	2111 (1647 to 2749)	61.0 (54.8 to 65.1)	1.0 (0.5 to 1.6)	36.9 (23.8 to 50.3)	0.6 (0.5 to 0.7)	2.7 (1.1 to 4.7)	3.0 (1.8 to 4.2)	-0.1 (-6.5 to 6.6)	2.0 (-2.2 to 6.4)	10.0 (7.6 to 12.9)
**Seychelles**	957 (870 to 1057)	1416 (602 to 2414)	96.9 (92.9 to 98.5)	0.3 (0.2 to 0.4)	2.3 (1.7 to 3.7)	0.0 (0.0 to 0.0)	2.3 (-3.0 to 6.4)	2.3 (-3.3 to 6.5)	7.9 (4.9 to 11.2)	1.7 (-6.4 to 10.9)	-70.5 (-71.1 to - 69.8)
**Sierra Leone**	248 (232 to 260)	214 (164 to 274)	13.6 (7.4 to 24.1)	9.8 (5.0 to 16.1)	42.3 (40.3 to 44.9)	32.8 (28.2 to 40.9)	-1.0 (-2.8 to 0.7)	1.2 (-4.4 to 7.1)	4.0 (-2.0 to 9.8)	-1.8 (-3.8 to 0.5)	-0.4 (-2.4 to 2.4)
**Singapore**	3657 (3529 to 3810)	4651 (3516 to 6053)	49.3 (38.0 to 59.8)	17.5 (16.8 to 17.7)	32.3 (31.5 to 32.9)	0.0 (0.0 to 0.0)	1.6 (-0.3 to 3.5)	1.2 (-2.2 to 4.4)	1.9 (-0.2 to 3.8)	1.7 (-0.3 to 3.7)	-
**Slovakia**	2216 (2085 to 2350)	3539 (2718 to 4524)	73.8 (70.9 to 77.2)	2.8 (1.6 to 4.9)	22.7 (17.1 to 29.8)	0.0 (0.0 to 0.0)	3.1 (1.3 to 4.9)	2.6 (0.5 to 4.7)	5.1 (-3.6 to 15.0)	4.4 (0.7 to 8.6)	-
**Slovenia**	2806 (2744 to 2884)	3993 (3408 to 4632)	67.9 (67.3 to 68.5)	19.4 (12.9 to 27.5)	12.4 (10.8 to 13.9)	0.0 (0.0 to 0.0)	2.4 (1.3 to 3.4)	2.0 (0.9 to 3.2)	3.5 (-0.1 to 7.1)	2.2 (0.2 to 4.2)	-
**Solomon Islands**	157 (144 to 166)	216 (144 to 308)	66.5 (54.5 to 75.8)	0.2 (0.2 to 0.2)	3.7 (3.4 to 4.1)	28.2 (26.8 to 31.3)	2.0 (-0.7 to 4.6)	2.2 (-1.9 to 5.8)	1.3 (-1.8 to 4.6)	2.6 (-0.8 to 5.9)	1.7 (-0.7 to 4.9)
**Somalia**	42 (42 to 43)	73 (57 to 101)	7.9 (6.7 to 8.4)	1.6 (1.3 to 1.7)	21.8 (16.9 to 25.6)	68.5 (60.4 to 77.7)	3.6 (2.0 to 5.9)	0.6 (-0.6 to 1.9)	0.0 (-1.2 to 1.1)	-0.2 (-0.7 to 0.4)	4.8 (2.7 to 7.6)
**South Africa**	1109 (1091 to 1128)	1207 (1036 to 1404)	59.0 (55.5 to 62.8)	27.3 (23.5 to 31.1)	9.4 (8.6 to 9.5)	3.9 (3.1 to 5.2)	0.5 (-0.5 to 1.6)	1.2 (-0.2 to 2.7)	-1.4 (-3.3 to 0.6)	1.8 (0.2 to 3.0)	3.6 (1.5 to 6.3)
**South Korea**	2835 (2785 to 2884)	4956 (4050 to 6036)	52.8 (48.9 to 56.4)	8.2 (5.8 to 10.3)	38.4 (35.4 to 43.2)	0.0 (0.0 to 0.0)	3.8 (2.4 to 5.2)	3.3 (1.4 to 5.2)	5.0 (1.3 to 8.1)	4.0 (2.2 to 6.3)	-
**South Sudan**	81 (79 to 84)	126 (109 to 147)	45.6 (39.5 to 51.1)	2.7 (2.7 to 2.8)	37.0 (34.8 to 38.9)	14.3 (11.2 to 19.1)	2.9 (1.9 to 4.0)	6.4 (4.3 to 8.4)	-0.1 (-1.3 to 1.2)	-0.1 (-0.7 to 0.6)	3.1 (1.1 to 5.7)
**Spain**	3363 (3262 to 3450)	4359 (3827 to 4942)	71.4 (67.8 to 74.3)	5.1 (4.8 to 5.3)	23.4 (22.1 to 24.5)	0.0 (0.0 to 0.0)	1.7 (0.9 to 2.6)	1.8 (0.5 to 2.9)	2.1 (0.8 to 3.4)	1.5 (0.3 to 2.7)	-
**Sri Lanka**	360 (348 to 370)	664 (481 to 897)	50.3 (42.7 to 59.1)	5.5 (5.3 to 5.5)	42.1 (38.1 to 46.7)	1.1 (1.0 to 1.2)	4.1 (1.9 to 6.4)	3.6 (0.3 to 6.9)	2.7 (0.3 to 4.9)	5.1 (2.3 to 8.1)	-2.9 (-4.6 to 0.5)
**Sudan**	282 (262 to 306)	371 (226 to 566)	28.2 (19.7 to 37.7)	2.8 (2.7 to 3.0)	62.6 (51.1 to 74.0)	3.9 (3.8 to 4.3)	1.7 (-1.5 to 4.9)	1.2 (-4.2 to 6.7)	0.8 (-1.9 to 3.6)	1.5 (-3.0 to 5.8)	3.6 (1.5 to 6.3)

			Health spending per total, 2030				Per capita annualized rate of change, 2015-2030				
Location name	Total health spending per capita 2015 ($)	Total health spending per capita 2030 ($)	Government (%)	Pre-paid private (%)	Out-of-pocket (%)	Development assistance for health (%)	Total (%)	Government (%)	Pre-paid private (%)	Out-of-pocket (%)	Development assistance for health (%)
**Suriname**	993 (904 to 1074)	879 (556 to 1346)	42.6 (23.9 to 62.5)	39.4 (36.3 to 40.7)	15.6 (14.7 to 16.1)	0.1 (0.0 to 0.2)	-1.0 (-3.8 to 2.1)	-2.4 (-8.7 to 3.4)	-0.2 (-3.4 to 2.6)	1.2 (-1.8 to 4.4)	-43.2 (-100.0 to -6.5)
**Swaziland**	693 (661 to 729)	1043 (720 to 1436)	57.9 (46.6 to 68.4)	3.9 (3.8 to 3.9)	8.7 (8.4 to 9.0)	28.3 (26.9 to 31.8)	2.7 (0.1 to 5.0)	2.2 (-1.7 to 5.9)	-2.8 (-5.1 to -0.5)	1.2 (-0.9 to 3.3)	4.3 (2.1 to 7.0)
**Sweden**	5550 (5346 to 5748)	7051 (5887 to 8374)	81.3 (78.3 to 83.7)	1.1 (1.0 to 1.2)	17.4 (16.8 to 18.2)	0.0 (0.0 to 0.0)	1.6 (0.4 to 2.8)	1.4 (0.0 to 2.8)	1.4 (-0.6 to 3.2)	2.5 (1.1 to 4.0)	-
**Switzerland**	7465 (7252 to 7662)	7486 (6586 to 8425)	68.9 (66.1 to 72.0)	5.8 (5.0 to 6.7)	25.2 (24.5 to 26.3)	0.0 (0.0 to 0.0)	0.0 (-0.8 to 0.8)	-0.2 (-1.3 to 1.0)	-0.8 (-2.6 to 1.1)	0.6 (-0.4 to 1.8)	-
**Syria**	241 (207 to 284)	263 (188 to 364)	41.5 (29.5 to 51.5)	6.4 (4.1 to 10.0)	48.0 (42.9 to 51.5)	2.5 (2.3 to 2.8)	0.5 (-2.0 to 2.9)	0.6 (-3.8 to 4.4)	0.8 (-5.0 to 6.9)	0.2 (-3.2 to 3.5)	1.1 (-1.5 to 3.9)
**Taiwan**	2535 (2513 to 2555)	3577 (3004 to 4204)	57.1 (53.4 to 61.1)	13.1 (10.4 to 16.2)	29.4 (26.2 to 33.2)	0.0 (0.0 to 0.0)	2.3 (1.1 to 3.4)	2.0 (0.4 to 3.5)	2.5 (-0.8 to 5.9)	2.8 (0.7 to 4.9)	-
**Tajikistan**	200 (192 to 209)	305 (214 to 416)	26.5 (17.7 to 35.4)	0.4 (0.2 to 0.6)	63.2 (57.8 to 71.7)	8.5 (8.1 to 9.5)	2.8 (0.4 to 5.0)	2.1 (-2.6 to 6.4)	3.2 (-5.4 to 11.7)	2.7 (-0.1 to 5.9)	3.5 (1.4 to 6.1)
**Tanzania**	161 (147 to 176)	291 (202 to 412)	48.6 (34.2 to 62.6)	1.5 (1.4 to 1.5)	25.5 (24.8 to 25.9)	22.9 (21.8 to 24.5)	3.9 (1.5 to 6.6)	5.8 (0.9 to 10.6)	1.2 (-1.6 to 3.8)	3.2 (0.4 to 5.9)	1.5 (-0.5 to 4.0)
**Thailand**	614 (588 to 643)	940 (712 to 1231)	80.6 (75.7 to 84.7)	9.1 (8.6 to 9.5)	9.5 (7.0 to 12.7)	0.2 (0.2 to 0.3)	2.8 (1.0 to 4.7)	3.0 (0.8 to 5.3)	2.6 (0.4 to 4.7)	1.1 (-3.0 to 5.6)	-0.1 (-2.6 to 3.0)
**The Bahamas**	1818 (1713 to 1935)	2288 (1815 to 2942)	49.9 (41.4 to 57.9)	21.6 (20.7 to 22.0)	27.8 (23.8 to 30.6)	0.0 (0.0 to 0.0)	1.5 (-0.1 to 3.3)	1.9 (-0.9 to 4.7)	0.9 (-1.1 to 2.8)	1.2 (-1.5 to 3.6)	-
**The Gambia**	141 (135 to 148)	176 (129 to 235)	36.8 (23.8 to 50.0)	3.0 (1.7 to 5.0)	13.5 (11.9 to 15.0)	45.3 (42.2 to 50.9)	1.4 (-0.6 to 3.5)	1.7 (-3.0 to 6.1)	-1.6 (-7.2 to 4.0)	0.0 (-1.3 to 1.2)	1.9 (-0.2 to 4.4)
**Timor-Leste**	103 (96 to 112)	183 (142 to 233)	52.6 (48.2 to 56.2)	1.0 (0.6 to 1.5)	6.3 (4.0 to 9.5)	39.2 (34.8 to 47.1)	3.9 (2.2 to 5.7)	3.5 (1.1 to 6.0)	-1.0 (-5.8 to 4.2)	0.3 (-4.2 to 5.2)	4.3 (2.2 to 7.0)
**Togo**	96 (92 to 101)	128 (99 to 168)	30.5 (19.3 to 45.4)	6.4 (5.5 to 6.7)	48.7 (46.0 to 50.6)	13.2 (11.8 to 15.1)	1.9 (0.1 to 3.8)	2.2 (-2.5 to 7.1)	2.0 (-0.7 to 4.4)	1.1 (-1.0 to 3.3)	2.3 (0.2 to 4.9)
**Tonga**	241 (229 to 255)	468 (330 to 621)	51.9 (42.9 to 59.4)	7.0 (3.7 to 12.2)	8.3 (8.0 to 8.8)	31.2 (25.2 to 40.3)	4.4 (2.1 to 6.6)	3.5 (-0.1 to 6.6)	6.2 (-1.0 to 13.9)	1.5 (-0.9 to 4.2)	5.2 (1.7 to 8.8)
**Trinidad and Tobago**	2024 (1917 to 2158)	2725 (1882 to 3680)	56.0 (46.4 to 64.7)	6.7 (4.6 to 9.3)	35.7 (30.9 to 42.4)	0.0 (0.0 to 0.0)	1.9 (-0.6 to 4.2)	2.1 (-1.6 to 5.4)	-0.1 (-4.8 to 4.5)	1.6 (-1.7 to 4.9)	-
**Tunisia**	791 (770 to 817)	1012 (869 to 1181)	52.9 (50.2 to 57.6)	5.4 (2.5 to 10.1)	41.1 (39.5 to 42.3)	0.2 (0.1 to 0.3)	1.6 (0.6 to 2.7)	1.2 (-0.2 to 2.8)	4.4 (-2.6 to 11.0)	1.9 (0.6 to 3.2)	-14.4 (-100.0 to 3.9)
**Turkey**	1029 (989 to 1074)	1905 (1302 to 2551)	80.8 (73.2 to 85.0)	3.5 (3.1 to 4.0)	15.1 (14.0 to 16.7)	0.0 (0.0 to 0.0)	4.1 (1.5 to 6.3)	4.4 (1.1 to 6.9)	1.8 (-1.6 to 5.2)	3.3 (0.2 to 6.2)	-83.1 (-100.0 to -9.8)
**Turkmenistan**	1171 (1078 to 1281)	1922 (1095 to 3148)	25.8 (21.7 to 28.9)	4.5 (3.4 to 5.6)	67.0 (56.0 to 78.9)	0.2 (0.2 to 0.2)	3.1 (-0.6 to 6.8)	3.4 (-1.2 to 7.9)	2.8 (-2.5 to 8.2)	2.8 (-2.0 to 7.7)	-2.7 (-100.0 to 4.6)
**Uganda**	159 (146 to 168)	215 (168 to 277)	13.5 (10.2 to 17.0)	12.6 (6.2 to 23.4)	40.9 (37.7 to 44.4)	31.7 (28.0 to 38.0)	2.0 (0.3 to 3.8)	2.0 (-1.7 to 5.5)	2.1 (-4.6 to 8.7)	2.2 (-0.1 to 4.7)	1.3 (-0.8 to 3.7)

Location name	Total health spending per capita 2015 ($)	Total health spending per capita 2030 ($)	Health spending per total, 2030 Government (%)	Pre-paid private (%)	Out-of-pocket (%)	Development assistance for health (%)	Per capita annualized rate of change, 2015-2030 Total (%)	Government (%)	Pre-paid private (%)	Out-of-pocket (%)	Development assistance for health (%)
**Ukraine**	598 (575 to 624)	734 (597 to 890)	44.8 (41.1 to 50.5)	2.4 (1.8 to 3.0)	49.7 (45.7 to 53.2)	2.6 (2.2 to 3.3)	1.3 (0.0 to 2.7)	0.8 (-1.1 to 3.1)	-1.2 (-4.3 to 1.7)	1.7 (-0.2 to 3.6)	5.2 (3.0 to 8.0)
**United Arab Emirates**	2489 (2354 to 2636)	3304 (2024 to 5086)	65.2 (53.5 to 75.5)	9.4 (9.2 to 9.8)	23.2 (16.5 to 31.0)	0.0 (0.0 to 0.0)	1.7 (-1.4 to 4.9)	0.9 (-3.6 to 5.1)	2.5 (-0.7 to 5.9)	3.4 (-2.3 to 9.1)	-
**United Kingdom**	4285 (4160 to 4409)	4948 (4041 to 5844)	78.2 (74.7 to 80.6)	5.3 (5.0 to 5.5)	16.2 (13.0 to 20.2)	0.0 (0.0 to 0.0)	0.9 (-0.4 to 2.1)	0.7 (-0.9 to 2.2)	1.5 (-0.2 to 2.9)	1.6 (-1.5 to 4.5)	-
**United States**	9839 (9677 to 9983)	13297 (10539 to 15279)	57.9 (56.6 to 61.0)	31.5 (19.6 to 35.3)	10.2 (9.9 to 10.7)	0.0 (0.0 to 0.0)	2.0 (0.5 to 3.0)	2.9 (1.7 to 4.1)	0.6 (-3.9 to 2.4)	1.4 (0.2 to 2.6)	-
**Uruguay**	2038 (1943 to 2116)	2644 (2154 to 3242)	72.4 (68.6 to 76.1)	10.9 (7.9 to 15.2)	16.1 (13.3 to 19.0)	0.0 (0.0 to 0.0)	1.7 (0.3 to 3.1)	2.0 (0.3 to 3.7)	0.0 (-3.4 to 3.6)	1.6 (-1.0 to 4.3)	-70.5 (-71.1 to -69.8)
**Uzbekistan**	451 (439 to 463)	777 (572 to 1032)	50.2 (41.2 to 58.3)	3.3 (3.0 to 3.6)	42.8 (41.1 to 47.8)	2.6 (2.5 to 3.0)	3.6 (1.7 to 5.7)	3.2 (-0.2 to 6.3)	5.3 (2.6 to 8.1)	3.7 (1.4 to 6.5)	7.1 (4.9 to 9.8)
**Vanuatu**	147 (136 to 161)	139 (103 to 181)	57.3 (48.3 to 65.2)	2.3 (2.3 to 2.3)	7.9 (7.7 to 8.2)	31.4 (27.6 to 37.4)	-0.4 (-2.4 to 1.5)	-0.1 (-3.3 to 2.8)	0.1 (-1.9 to 2.0)	1.3 (-0.8 to 3.4)	-1.3 (-4.5 to 1.8)
**Venezuela**	590 (559 to 616)	443 (259 to 700)	45.4 (32.9 to 53.2)	5.6 (4.9 to 6.4)	46.0 (35.7 to 60.0)	0.0 (0.0 to 0.0)	-2.1 (-5.3 to 1.2)	-2.4 (-7.6 to 1.9)	-1.4 (-5.5 to 2.8)	-2.3 (-7.2 to 2.9)	-74.4 (-75.0 to -73.9)
**Vietnam**	320 (308 to 334)	685 (514 to 913)	44.1 (39.8 to 48.1)	3.1 (2.9 to 3.2)	49.9 (40.8 to 59.9)	1.8 (1.7 to 2.1)	5.1 (3.1 to 7.2)	4.8 (2.1 to 7.6)	4.7 (2.5 to 7.1)	5.4 (2.1 to 8.9)	2.0 (0.0 to 4.6)
**Yemen**	179 (157 to 199)	172 (109 to 262)	19.0 (18.1 to 19.5)	1.2 (1.2 to 1.3)	74.6 (62.8 to 83.5)	4.0 (3.8 to 4.4)	-0.5 (-3.4 to 2.7)	2.1 (-1.3 to 5.6)	0.0 (-3.5 to 3.1)	-0.8 (-4.8 to 3.1)	-2.7 (-4.7 to -0.3)
**Zambia**	241 (231 to 251)	309 (240 to 408)	30.5 (18.4 to 43.8)	7.2 (6.6 to 7.6)	26.9 (25.4 to 27.6)	34.1 (30.6 to 39.1)	1.6 (-0.1 to 3.6)	1.3 (-3.8 to 5.9)	-0.9 (-3.2 to 1.5)	2.0 (0.4 to 3.6)	1.5 (-0.6 to 4.1)
**Zimbabwe**	191 (181 to 201)	188 (138 to 256)	26.5 (12.4 to 43.4)	14.9 (8.8 to 22.0)	29.1 (26.9 to 30.3)	27.6 (25.7 to 30.6)	-0.2 (-2.2 to 2.0)	-0.1 (-6.7 to 5.7)	-1.4 (-6.8 to 3.7)	-0.1 (-2.1 to 1.7)	0.2 (-1.8 to 2.6)

B.2 Table: Future Health Spending in 2015 and 2040

This table shows the total health spending per capita values (2017 purchasing power parity US$) in 2015 and 2040, the share of each health expenditure component per total spending, and the annualized rate of change of the components of health spending per capita between 2015 and 2040.

			Health spending per total, 2040				Per capita annualized rate of change, 2015-2040				
Location name	Total health spending per capita 2015 ($)	Total health spending per capita 2040 ($)	Government (%)	Pre-paid private (%)	Out-of-pocket (%)	Development assistance for health (%)	Total (%)	Government (%)	Pre-paid private (%)	Out-of-pocket (%)	Development assistance for health (%)
**Global**	1332 (1325 to 1343)	2318 (2099 to 2540)	61.3 (57.2 to 66.3)	13.5 (8.3 to 16.9)	24.7 (21.9 to 27.6)	0.5 (0.5 to 0.6)	2.2 (1.8 to 2.6)	2.3 (1.9 to 2.9)	1.1 (-0.9 to 2.1)	2.6 (2.3 to 3.0)	2.3 (1.9 to 2.9)
**World Bank Income Groups**											
**High-income**	5551 (5503 to 5605)	8666 (7430 to 9657)	67.3 (61.7 to 76.1)	19.2 (9.9 to 24.8)	13.4 (11.5 to 16.0)	0.0 (0.0 to 0.0)	1.8 (1.2 to 2.2)	2.0 (1.5 to 2.5)	1.2 (-1.7 to 2.5)	1.6 (1.1 to 2.1)	-
**Upper-middle-income**	949 (942 to 959)	2670 (2217 to 3302)	64.2 (56.7 to 71.3)	6.9 (4.7 to 10.1)	28.8 (22.4 to 35.5)	0.1 (0.1 to 0.2)	4.2 (3.4 to 5.1)	4.6 (3.5 to 5.9)	2.6 (1.2 to 4.3)	3.7 (3.0 to 4.5)	1.6 (-0.1 to 3.4)
**Lower-middle-income**	266 (263 to 268)	714 (638 to 801)	31.9 (27.5 to 37.1)	8.4 (6.3 to 10.8)	57.9 (52.7 to 63.0)	1.8 (1.4 to 2.2)	4.0 (3.6 to 4.5)	4.0 (3.3 to 4.7)	4.5 (3.4 to 5.6)	4.0 (3.3 to 4.8)	1.8 (1.1 to 2.5)
**Low-income**	110 (108 to 111)	190 (166 to 219)	29.8 (23.2 to 37.7)	11.8 (6.9 to 20.2)	35.7 (29.7 to 41.7)	22.7 (18.6 to 26.7)	2.2 (1.7 to 2.8)	3.5 (2.3 to 4.9)	4.1 (1.9 to 7.0)	1.8 (1.2 to 2.6)	1.0 (0.3 to 1.8)
**GBD Super-Regions**											
**Central Europe, Eastern Europe, and Central Asia**	1288 (1273 to 1300)	2120 (1847 to 2427)	56.3 (49.5 to 62.7)	3.3 (2.4 to 4.3)	39.9 (33.4 to 46.9)	0.5 (0.3 to 0.6)	2.0 (1.4 to 2.6)	1.6 (0.9 to 2.4)	2.4 (1.3 to 3.5)	2.5 (1.7 to 3.5)	4.1 (3.1 to 5.4)
**GBD high-income**	5839 (5785 to 5897)	9054 (7715 to 10101)	67.5 (61.8 to 76.9)	19.6 (9.9 to 25.5)	12.8 (10.9 to 15.5)	0.0 (0.0 to 0.0)	1.8 (1.1 to 2.2)	2.0 (1.5 to 2.5)	1.1 (-1.9 to 2.4)	1.5 (1.0 to 2.0)	-40.0 (-77.4 to 1.5)
**Latin America and Caribbean**	1065 (1051 to 1077)	1550 (1356 to 1751)	51.2 (44.9 to 57.6)	18.6 (12.4 to 23.7)	29.9 (25.1 to 35.7)	0.3 (0.2 to 0.6)	1.5 (1.0 to 2.0)	1.6 (0.8 to 2.4)	1.7 (0.1 to 2.8)	1.2 (0.6 to 2.0)	-1.7 (-3.7 to 0.9)
**North Africa and Middle East**	888 (872 to 905)	1496 (1254 to 1806)	56.9 (48.5 to 65.4)	7.8 (4.8 to 12.3)	34.9 (27.5 to 42.8)	0.4 (0.3 to 0.6)	2.1 (1.4 to 2.9)	1.9 (0.8 to 3.1)	2.3 (0.6 to 4.3)	2.3 (1.4 to 3.4)	1.9 (0.7 to 3.3)
**South Asia**	210 (207 to 212)	692 (587 to 828)	28.9 (22.3 to 36.6)	9.9 (6.2 to 14.4)	60.6 (52.8 to 67.9)	0.6 (0.4 to 0.9)	4.9 (4.2 to 5.6)	5.4 (4.1 to 6.6)	5.8 (3.8 to 7.6)	4.6 (3.7 to 5.6)	0.0 (-1.3 to 1.6)
**Southeast Asia, East Asia, and Oceania**	672 (663 to 682)	2632 (2015 to 3454)	63.6 (53.8 to 72.9)	5.3 (3.0 to 9.0)	31.0 (22.6 to 40.4)	0.1 (0.1 to 0.2)	5.6 (4.5 to 6.8)	6.1 (4.4 to 7.7)	3.5 (1.4 to 6.0)	5.0 (4.1 to 6.0)	1.7 (0.6 to 3.2)
**Sub-Saharan Africa**	202 (199 to 206)	289 (260 to 327)	34.5 (28.9 to 41.1)	11.0 (8.1 to 15.7)	39.4 (33.4 to 45.0)	15.1 (12.7 to 17.5)	1.4 (1.0 to 1.9)	1.4 (0.6 to 2.4)	0.0 (-1.2 to 1.6)	2.1 (1.3 to 2.9)	1.3 (0.7 to 1.9)
**Countries**											
**Afghanistan**	168 (160 to 174)	131 (94 to 175)	13.3 (11.5 to 15.8)	0.6 (0.4 to 0.8)	61.6 (54.7 to 68.6)	23.2 (18.8 to 30.7)	-1.0 (-2.3 to 0.2)	2.4 (0.5 to 4.4)	-1.6 (-4.8 to 1.3)	-1.9 (-3.6 to -0.2)	0.2 (-1.9 to 2.6)
**Albania**	848 (796 to 908)	1932 (1332 to 2793)	48.6 (42.6 to 51.6)	4.0 (2.7 to 5.9)	45.6 (33.6 to 58.0)	0.0 (0.0 to 0.1)	3.3 (1.8 to 4.9)	3.9 (1.9 to 5.8)	5.3 (0.0 to 10.7)	2.5 (-0.1 to 5.2)	-39.1 (-100.0 to -3.9)
**Algeria**	1026 (998 to 1055)	1426 (873 to 2276)	68.4 (52.3 to 80.1)	1.1 (0.9 to 1.2)	29.6 (22.9 to 35.8)	0.0 (0.0 to 0.0)	1.2 (-0.6 to 3.2)	1.0 (-1.9 to 3.7)	0.4 (-1.0 to 1.9)	1.5 (0.4 to 2.5)	-27.1 (-100.0 to -0.1)
**Andorra**	9203 (8659 to 9745)	9043 (7425 to 10975)	51.8 (47.0 to 57.9)	6.5 (5.6 to 7.5)	41.3 (37.7 to 44.8)	0.0 (0.0 to 0.0)	-0.1 (-0.9 to 0.8)	-0.4 (-1.7 to 0.9)	-0.7 (-2.1 to 0.7)	0.5 (-0.8 to 1.7)	-
**Angola**	197 (177 to 216)	246 (125 to 458)	42.7 (21.9 to 63.2)	6.1 (5.2 to 6.4)	43.6 (33.6 to 52.0)	2.5 (2.4 to 2.7)	0.7 (-1.8 to 3.4)	-0.7 (-5.6 to 3.7)	2.0 (-2.0 to 5.6)	1.9 (-1.7 to 5.6)	-1.2 (-3.3 to 1.4)

			Health spending per total, 2040				Per capita annualized rate of change, 2015-2040				
Location name	Total health spending per capita 2015 ($)	Total health spending per capita 2040 ($)	Government (%)	Pre-paid private (%)	Out-of-pocket (%)	Development assistance for health (%)	Total (%)	Government (%)	Pre-paid private (%)	Out-of-pocket (%)	Development assistance for health (%)
**Antigua and Barbuda**	1198 (1149 to 1251)	2302 (1271 to 4098)	60.7 (40.0 to 79.0)	16.4 (13.2 to 19.1)	20.1 (15.5 to 23.3)	0.0 (0.0 to 0.0)	2.5 (0.3 to 5.0)	1.9 (-2.0 to 5.7)	5.1 (1.9 to 8.4)	1.9 (0.3 to 3.4)	-68.7 (-69.4 to -68.0)
**Argentina**	1457 (1393 to 1528)	2234 (1435 to 3162)	77.9 (71.2 to 83.3)	5.2 (4.5 to 7.0)	15.5 (11.3 to 22.8)	0.1 (0.0 to 0.3)	1.6 (0.0 to 3.2)	2.0 (0.0 to 4.0)	-1.1 (-3.5 to 1.6)	1.0 (-2.0 to 4.1)	-50.6 (-100.0 to 1.0)
**Armenia**	849 (766 to 932)	1699 (711 to 3421)	19.7 (15.3 to 23.2)	1.0 (0.9 to 1.1)	75.2 (53.7 to 89.5)	1.5 (1.4 to 1.6)	2.5 (-0.7 to 5.8)	3.0 (0.4 to 5.3)	1.0 (-2.8 to 4.7)	2.2 (-2.5 to 6.3)	3.0 (0.3 to 6.1)
**Australia**	4400 (4263 to 4559)	6007 (5249 to 6910)	67.6 (64.5 to 69.8)	13.1 (9.2 to 18.4)	18.9 (17.7 to 20.1)	0.0 (0.0 to 0.0)	1.2 (0.7 to 1.8)	1.2 (0.5 to 2.0)	1.3 (-0.6 to 3.3)	1.1 (0.1 to 2.1)	-
**Austria**	5183 (5116 to 5236)	6654 (5774 to 7727)	75.3 (72.1 to 78.0)	6.3 (5.1 to 7.5)	18.3 (18.0 to 18.6)	0.0 (0.0 to 0.0)	1.0 (0.4 to 1.6)	1.0 (0.3 to 1.7)	0.8 (-0.6 to 2.2)	1.1 (0.4 to 1.7)	-
**Azerbaijan**	1221 (1132 to 1322)	2579 (1086 to 5564)	19.9 (17.1 to 21.0)	0.4 (0.3 to 0.5)	76.4 (59.0 to 91.3)	0.3 (0.2 to 0.3)	2.7 (-0.4 to 6.3)	2.6 (-0.4 to 5.5)	0.4 (-1.5 to 2.4)	2.5 (-1.6 to 6.9)	3.1 (-0.9 to 6.7)
**Bahrain**	2470 (2363 to 2572)	3039 (1679 to 4826)	63.4 (51.0 to 74.5)	9.3 (6.4 to 16.1)	23.9 (18.0 to 31.4)	0.0 (0.0 to 0.0)	0.7 (-1.4 to 2.8)	0.5 (-2.6 to 3.2)	0.8 (-2.8 to 5.2)	0.6 (-2.6 to 3.8)	-
**Bangladesh**	90 (86 to 94)	306 (248 to 385)	18.4 (14.7 to 21.8)	2.5 (1.7 to 3.5)	76.8 (74.0 to 79.6)	1.7 (1.2 to 2.7)	5.0 (4.1 to 6.0)	5.8 (4.0 to 7.7)	4.7 (2.2 to 7.2)	5.2 (4.1 to 6.3)	-1.3 (-3.6 to 1.5)
**Barbados**	1237 (1175 to 1293)	1521 (1112 to 1983)	50.1 (37.3 to 58.9)	6.9 (6.3 to 7.7)	42.0 (41.0 to 44.5)	0.0 (0.0 to 0.0)	0.8 (-0.5 to 1.9)	1.0 (-1.4 to 2.9)	0.6 (-1.1 to 2.2)	0.5 (-0.9 to 1.8)	-
**Belarus**	1232 (1184 to 1275)	1911 (1273 to 2975)	48.4 (43.2 to 51.0)	1.6 (1.4 to 1.7)	47.1 (29.8 to 66.9)	1.2 (1.0 to 1.4)	1.7 (0.1 to 3.6)	0.7 (-0.7 to 2.1)	-0.6 (-2.7 to 1.5)	2.9 (-0.4 to 6.3)	4.2 (1.7 to 7.0)
**Belgium**	4939 (4782 to 5095)	6097 (5027 to 7308)	81.2 (78.8 to 83.9)	0.0 (0.0 to 0.1)	18.6 (17.1 to 20.4)	0.0 (0.0 to 0.0)	0.8 (0.1 to 1.5)	0.8 (-0.1 to 1.7)	4.9 (3.2 to 9.7)	1.0 (-0.3 to 2.2)	-
**Belize**	544 (519 to 572)	760 (536 to 1041)	60.3 (52.5 to 69.1)	12.5 (5.1 to 25.3)	21.7 (21.4 to 22.3)	3.8 (2.0 to 6.2)	1.3 (-0.1 to 2.7)	0.9 (-1.0 to 2.8)	4.2 (-0.5 to 8.9)	1.0 (-0.4 to 2.5)	-0.2 (-3.9 to 3.3)
**Benin**	82 (79 to 85)	113 (81 to 156)	31.4 (15.7 to 49.4)	5.9 (5.4 to 6.3)	46.3 (44.3 to 47.3)	14.8 (11.9 to 19.2)	1.3 (0.0 to 2.6)	2.7 (-1.2 to 6.2)	1.6 (0.0 to 3.2)	1.6 (0.4 to 2.8)	-1.7 (-3.7 to 0.7)
**Bhutan**	285 (272 to 298)	618 (334 to 1002)	71.7 (55.2 to 82.0)	1.2 (1.2 to 1.3)	24.6 (23.1 to 27.2)	0.3 (0.0 to 0.6)	3.0 (0.6 to 5.2)	3.0 (-0.5 to 5.7)	3.6 (1.2 to 6.2)	3.9 (1.3 to 6.5)	-27.3 (-100.0 to -4.5)
**Bolivia**	450 (432 to 464)	938 (607 to 1342)	74.5 (62.8 to 82.1)	2.1 (1.5 to 3.0)	22.2 (21.8 to 22.8)	0.2 (0.0 to 0.3)	2.9 (1.2 to 4.5)	3.3 (0.9 to 5.3)	1.4 (-1.5 to 4.4)	2.2 (0.6 to 3.8)	-16.0 (-100.0 to -4.6)
**Bosnia and Herzegovina**	1076 (999 to 1174)	1916 (1057 to 3081)	71.4 (57.5 to 79.5)	1.7 (1.4 to 2.4)	23.8 (17.2 to 32.6)	0.6 (0.3 to 1.5)	2.2 (0.0 to 4.3)	2.3 (-0.7 to 4.9)	4.1 (-1.1 to 9.4)	1.3 (-2.3 to 5.0)	2.2 (-2.2 to 8.4)
**Botswana**	1019 (946 to 1127)	2012 (1346 to 2998)	46.2 (37.6 to 48.8)	33.4 (21.7 to 50.5)	8.5 (5.0 to 13.3)	9.1 (0.0 to 12.1)	2.7 (1.0 to 4.5)	1.9 (-0.5 to 4.1)	2.9 (-0.4 to 6.4)	4.6 (1.0 to 8.4)	1.1 (-19.7 to 6.1)
**Brazil**	1431 (1407 to 1453)	1858 (1373 to 2356)	44.3 (31.1 to 53.2)	30.5 (21.2 to 33.9)	24.1 (23.1 to 26.1)	0.0 (0.0 to 0.0)	1.0 (-0.1 to 2.0)	1.1 (-1.5 to 2.9)	1.3 (-1.3 to 2.8)	0.3 (-0.5 to 1.2)	-1.6 (-5.6 to 1.5)
**Brunei**	2092 (1942 to 2276)	2052 (1008 to 3645)	84.7 (76.5 to 90.8)	4.4 (3.7 to 5.2)	9.1 (5.6 to 13.2)	0.0 (0.0 to 0.0)	-0.3 (-2.9 to 2.2)	-0.5 (-3.6 to 2.3)	-0.7 (-2.6 to 1.1)	1.3 (-3.1 to 5.5)	-

			Health spending per total, 2040				Per capita annualized rate of change, 2015-2040				
Location name	Total health spending per capita 2015 ($)	Total health spending per capita 2040 ($)	Government (%)	Pre-paid private (%)	Out-of-pocket (%)	Development assistance for health (%)	Total (%)	Government (%)	Pre-paid private (%)	Out-of-pocket (%)	Development assistance for health (%)
**Bulgaria**	1620 (1566 to 1672)	3435 (2421 to 4721)	57.4 (44.3 to 68.4)	1.7 (1.1 to 2.7)	40.2 (37.4 to 44.8)	0.0 (0.0 to 0.0)	3.0 (1.6 to 4.4)	3.4 (1.0 to 5.6)	4.3 (1.2 to 7.8)	2.3 (1.4 to 3.6)	-42.6 (-100.0 to -3.9)
**Burkina Faso**	94 (91 to 97)	192 (133 to 274)	41.5 (30.2 to 54.1)	8.8 (3.8 to 18.4)	33.3 (26.2 to 42.4)	13.6 (11.5 to 17.1)	2.8 (1.4 to 4.4)	4.2 (1.4 to 6.8)	4.0 (-0.5 to 9.1)	2.5 (0.1 to 5.0)	-0.1 (-2.1 to 2.4)
**Burundi**	67 (63 to 71)	85 (58 to 130)	22.6 (17.0 to 27.3)	0.8 (0.3 to 1.5)	19.6 (12.7 to 26.3)	54.5 (46.8 to 66.6)	0.9 (-0.6 to 2.7)	-0.4 (-2.9 to 2.2)	-3.0 (-7.5 to 1.6)	0.8 (-2.6 to 4.1)	1.5 (-0.5 to 4.2)
**Cambodia**	213 (199 to 229)	602 (368 to 942)	18.9 (14.2 to 23.0)	0.5 (0.5 to 0.5)	74.1 (65.4 to 83.9)	4.8 (4.5 to 5.4)	4.1 (2.1 to 6.2)	3.6 (0.5 to 6.8)	4.7 (1.9 to 7.3)	4.9 (2.3 to 7.4)	-0.9 (-3.0 to 1.6)
**Cameroon**	156 (148 to 163)	252 (199 to 309)	18.8 (10.8 to 28.3)	3.8 (2.4 to 5.6)	67.4 (65.0 to 71.2)	9.0 (6.7 to 13.2)	1.9 (1.0 to 2.8)	2.7 (-0.4 to 5.5)	2.8 (-1.4 to 6.9)	1.8 (0.8 to 2.9)	0.5 (-1.5 to 3.0)
**Canada**	4921 (4835 to 5031)	7108 (5323 to 8917)	73.9 (65.4 to 79.4)	13.3 (12.0 to 14.2)	12.4 (11.0 to 14.5)	0.0 (0.0 to 0.0)	1.4 (0.3 to 2.4)	1.5 (-0.2 to 2.7)	1.9 (0.4 to 3.1)	0.8 (0.3 to 1.3)	-
**Cape Verde**	356 (340 to 372)	472 (286 to 728)	58.9 (40.8 to 73.7)	3.9 (3.3 to 4.5)	34.8 (29.4 to 39.6)	0.7 (0.0 to 2.8)	1.0 (-0.9 to 2.9)	0.8 (-2.5 to 3.6)	3.1 (0.4 to 5.7)	2.9 (1.5 to 4.1)	-44.8 (-100.0 to -3.5)
**Central African Republic**	28 (27 to 30)	40 (24 to 65)	11.3 (5.9 to 20.0)	4.3 (2.3 to 7.3)	26.0 (24.5 to 27.4)	54.9 (39.5 to 72.1)	1.3 (-0.8 to 3.3)	0.1 (-4.2 to 4.7)	0.8 (-3.7 to 5.5)	-0.9 (-2.8 to 0.9)	3.0 (-0.4 to 6.3)
**Chad**	103 (97 to 110)	120 (84 to 173)	22.0 (9.6 to 40.4)	4.2 (2.4 to 7.1)	62.0 (59.2 to 63.4)	9.6 (7.4 to 14.1)	0.5 (-0.8 to 2.1)	-0.6 (-5.2 to 3.7)	-0.8 (-4.2 to 3.3)	0.8 (-0.8 to 2.4)	1.2 (-1.1 to 4.4)
**Chile**	1950 (1921 to 1984)	2445 (1920 to 3060)	55.7 (50.6 to 62.6)	8.7 (3.8 to 15.7)	34.8 (33.7 to 36.3)	0.0 (0.0 to 0.0)	0.9 (-0.1 to 1.8)	0.5 (-0.8 to 1.9)	1.7 (-2.5 to 5.3)	1.2 (0.1 to 2.2)	-71.1 (-71.7 to -70.5)
**China**	779 (765 to 794)	3597 (2617 to 4937)	68.6 (59.6 to 76.5)	4.4 (2.7 to 6.9)	26.2 (25.0 to 27.0)	0.0 (0.0 to 0.0)	6.3 (5.0 to 7.7)	6.9 (5.0 to 8.8)	3.7 (0.5 to 7.1)	5.3 (4.0 to 6.5)	-55.5 (-100.0 to -9.3)
**Colombia**	861 (806 to 914)	1411 (971 to 1954)	72.6 (66.2 to 78.1)	12.9 (12.0 to 14.1)	13.0 (6.6 to 23.7)	0.0 (0.0 to 0.0)	1.9 (0.5 to 3.3)	2.1 (0.2 to 3.8)	2.5 (0.8 to 4.3)	0.3 (-3.6 to 4.4)	-78.8 (-100.0 to -4.6)
**Comoros**	131 (123 to 138)	116 (89 to 151)	26.7 (13.3 to 41.0)	5.7 (4.5 to 6.8)	63.2 (61.2 to 65.9)	3.1 (0.0 to 8.6)	-0.5 (-1.6 to 0.6)	2.3 (-1.4 to 5.4)	1.2 (-0.7 to 3.1)	-1.1 (-2.1 to -0.1)	-22.1 (-100.0 to 0.1)
**Congo**	181 (171 to 194)	218 (109 to 375)	52.4 (37.3 to 67.5)	1.8 (1.8 to 1.9)	39.8 (34.9 to 48.9)	2.1 (1.0 to 3.0)	0.5 (-2.1 to 2.9)	0.9 (-3.0 to 4.4)	0.2 (-2.3 to 2.5)	0.0 (-3.0 to 3.3)	-3.0 (-8.1 to 0.9)
**Costa Rica**	1339 (1300 to 1375)	2139 (1611 to 2831)	69.4 (62.1 to 76.4)	4.5 (3.1 to 6.6)	25.2 (24.5 to 25.7)	0.1 (0.0 to 1.8)	1.9 (0.7 to 3.1)	1.5 (-0.1 to 3.1)	4.3 (1.7 to 7.3)	2.4 (1.3 to 3.5)	-50.5 (-100.0 to 18.0)
**Cote d'Ivoire**	131 (108 to 162)	235 (183 to 314)	32.9 (19.6 to 43.2)	3.1 (1.6 to 4.8)	44.4 (41.7 to 45.2)	18.3 (14.2 to 25.0)	2.4 (1.0 to 3.9)	2.2 (-1.2 to 5.7)	2.4 (-2.6 to 7.8)	2.1 (0.5 to 3.7)	3.3 (1.3 to 5.9)
**Croatia**	1736 (1660 to 1813)	3121 (2439 to 4070)	70.9 (67.3 to 72.4)	14.6 (5.7 to 29.6)	13.5 (9.8 to 17.3)	0.1 (0.0 to 0.3)	2.3 (1.4 to 3.5)	2.0 (1.0 to 2.9)	5.0 (0.3 to 10.2)	1.9 (-0.3 to 4.1)	-
**Cuba**	977 (870 to 1083)	1512 (1067 to 1978)	91.1 (88.7 to 93.2)	1.5 (0.9 to 2.6)	6.6 (5.0 to 8.8)	0.3 (0.2 to 0.6)	1.7 (0.2 to 3.0)	1.6 (0.0 to 3.0)	1.0 (-3.2 to 5.5)	3.1 (0.4 to 5.9)	2.1 (-0.7 to 5.6)
**Cyprus**	2821 (2504 to 3127)	4200 (3162 to 5545)	75.8 (69.9 to 82.4)	4.7 (4.0 to 5.5)	19.1 (16.8 to 21.2)	0.0 (0.0 to 0.0)	1.6 (0.4 to 2.9)	1.7 (0.2 to 3.3)	1.7 (-0.1 to 3.6)	1.1 (-0.4 to 2.6)	-

			Health spending per total, 2040				Per capita annualized rate of change, 2015-2040				
Location name	Total health spending per capita 2015 ($)	Total health spending per capita 2040 ($)	Government (%)	Pre-paid private (%)	Out-of-pocket (%)	Development assistance for health (%)	Total (%)	Government (%)	Pre-paid private (%)	Out-of-pocket (%)	Development assistance for health (%)
**Czech Republic**	2534 (2092 to 2924)	4223 (3359 to 5254)	70.5 (64.8 to 75.9)	4.0 (2.3 to 6.5)	25.1 (24.0 to 26.2)	0.0 (0.0 to 0.0)	2.1 (1.0 to 3.2)	1.9 (0.5 to 3.3)	4.0 (-0.7 to 9.0)	2.2 (0.5 to 4.2)	-
**Democratic Republic of the Congo**	44 (42 to 47)	50 (32 to 76)	31.0 (14.0 to 48.4)	3.9 (2.3 to 5.8)	30.7 (25.7 to 34.9)	31.1 (28.5 to 37.6)	0.4 (-1.3 to 2.2)	3.1 (-1.9 to 7.1)	-1.9 (-5.7 to 1.7)	-0.3 (-2.8 to 2.0)	-0.6 (-2.6 to 1.9)
**Denmark**	5144 (5049 to 5264)	6421 (5380 to 7561)	84.0 (80.9 to 86.5)	2.7 (2.0 to 3.2)	13.2 (13.0 to 13.3)	0.0 (0.0 to 0.0)	0.9 (0.2 to 1.6)	0.9 (0.0 to 1.7)	1.8 (0.0 to 3.2)	0.7 (0.0 to 1.3)	-
**Djibouti**	147 (140 to 156)	233 (121 to 373)	71.1 (49.7 to 81.3)	2.2 (2.1 to 2.2)	23.8 (23.1 to 26.2)	0.7 (0.0 to 2.0)	1.7 (-0.9 to 3.8)	2.5 (-1.4 to 5.2)	3.0 (0.4 to 5.1)	2.1 (-0.2 to 4.5)	-29.0 (-100.0 to -5.1)
**Dominica**	606 (591 to 620)	835 (527 to 1264)	67.9 (54.8 to 78.5)	1.9 (0.8 to 2.8)	28.1 (25.2 to 30.4)	0.8 (0.0 to 3.0)	1.2 (-0.6 to 2.9)	1.2 (-1.4 to 3.6)	2.4 (-3.6 to 6.8)	1.0 (-0.4 to 2.3)	-16.7 (-100.0 to 4.9)
**Dominican Republic**	932 (905 to 968)	2173 (1487 to 3104)	47.5 (36.3 to 62.3)	8.3 (6.0 to 11.0)	39.6 (37.2 to 41.3)	2.4 (0.0 to 8.6)	3.4 (1.9 to 4.9)	4.0 (1.4 to 6.8)	3.3 (0.4 to 6.1)	3.0 (1.3 to 4.7)	-33.7 (-100.0 to 5.5)
**Ecuador**	1028 (992 to 1077)	1341 (902 to 1909)	57.1 (44.9 to 68.0)	5.4 (3.9 to 7.3)	35.6 (31.3 to 40.9)	0.0 (0.0 to 0.0)	1.0 (-0.5 to 2.5)	1.5 (-1.0 to 3.8)	0.5 (-2.4 to 3.3)	0.2 (-1.9 to 2.2)	-69.3 (-100.0 to -7.3)
**Egypt**	484 (460 to 505)	886 (682 to 1113)	27.6 (21.1 to 34.5)	11.8 (6.7 to 22.0)	59.4 (57.3 to 61.9)	0.1 (0.0 to 0.1)	2.4 (1.4 to 3.4)	2.0 (-0.1 to 4.0)	4.0 (0.8 to 7.9)	2.3 (1.0 to 3.4)	-7.9 (-100.0 to -1.7)
**El Salvador**	598 (570 to 623)	865 (689 to 1064)	64.7 (59.5 to 70.7)	9.5 (5.9 to 14.1)	24.3 (23.3 to 25.4)	0.8 (0.4 to 1.4)	1.5 (0.6 to 2.3)	1.5 (0.3 to 2.7)	3.4 (0.6 to 6.0)	0.9 (-0.2 to 2.0)	-1.9 (-5.1 to 1.4)
**Equatorial Guinea**	1089 (988 to 1192)	2305 (1100 to 3901)	28.3 (14.3 to 45.4)	16.0 (11.8 to 23.7)	50.0 (39.8 to 63.5)	0.2 (0.0 to 1.4)	2.9 (0.1 to 5.3)	3.7 (-1.6 to 8.5)	5.1 (0.5 to 9.8)	1.6 (-2.1 to 5.0)	-57.7 (-100.0 to 7.6)
**Eritrea**	41 (37 to 45)	67 (42 to 100)	56.0 (43.8 to 64.6)	6.6 (4.4 to 10.0)	34.1 (27.6 to 44.7)	0.6 (0.0 to 1.9)	1.9 (0.0 to 3.7)	5.4 (2.4 to 8.2)	3.6 (0.2 to 7.2)	-0.1 (-2.7 to 2.7)	-23.6 (-100.0 to -5.1)
**Estonia**	1946 (1922 to 1969)	3362 (2420 to 4575)	66.1 (57.0 to 72.9)	1.2 (0.9 to 1.8)	31.7 (29.3 to 34.4)	0.0 (0.0 to 0.0)	2.2 (0.9 to 3.5)	1.6 (-0.3 to 3.4)	0.6 (-1.9 to 3.6)	3.4 (1.8 to 5.1)	-
**Ethiopia**	81 (77 to 85)	290 (191 to 449)	21.1 (12.4 to 30.2)	27.2 (11.3 to 48.1)	37.3 (33.7 to 39.9)	10.6 (9.5 to 12.2)	5.2 (3.5 to 7.1)	5.0 (1.3 to 8.7)	7.3 (2.2 to 12.0)	5.7 (3.5 to 7.8)	0.8 (-1.2 to 3.3)
**Federated States of Micronesia**	239 (230 to 247)	173 (102 to 287)	78.6 (69.2 to 81.9)	0.5 (0.4 to 0.6)	6.9 (6.5 to 7.5)	11.3 (0.0 to 43.9)	-1.4 (-3.4 to 0.7)	0.9 (-1.4 to 2.5)	0.9 (-0.5 to 2.5)	0.5 (-1.3 to 3.0)	-32.2 (-100.0 to 0.1)
**Fiji**	342 (328 to 358)	704 (493 to 993)	53.1 (45.5 to 58.2)	15.1 (7.9 to 27.7)	29.6 (21.4 to 35.5)	0.2 (0.0 to 0.8)	2.9 (1.5 to 4.4)	2.3 (0.2 to 4.1)	3.4 (-0.6 to 7.8)	4.4 (1.7 to 6.7)	-52.1 (-100.0 to -3.2)
**Finland**	4101 (4035 to 4163)	5864 (4658 to 7293)	78.4 (74.2 to 82.9)	3.2 (2.4 to 4.3)	18.1 (17.5 to 18.6)	0.0 (0.0 to 0.0)	1.4 (0.5 to 2.3)	1.5 (0.4 to 2.6)	2.0 (-0.1 to 4.1)	1.1 (0.1 to 2.0)	-
**France**	4741 (4677 to 4799)	5824 (5108 to 6592)	76.9 (75.0 to 78.9)	15.2 (13.3 to 17.4)	7.8 (7.1 to 8.5)	0.0 (0.0 to 0.0)	0.8 (0.3 to 1.3)	0.7 (0.1 to 1.3)	1.1 (0.0 to 2.1)	1.4 (0.5 to 2.2)	-
**Gabon**	487 (448 to 524)	653 (417 to 1031)	55.0 (41.3 to 69.6)	12.5 (7.8 to 17.3)	30.0 (28.0 to 30.8)	0.0 (0.0 to 0.1)	1.1 (-0.6 to 3.1)	0.8 (-2.0 to 3.8)	0.6 (-2.8 to 4.2)	1.6 (-0.4 to 3.6)	-55.9 (-100.0 to -6.2)
**Georgia**	803 (754 to 860)	1615 (796 to 2910)	39.5 (28.6 to 47.4)	2.4 (1.5 to 4.0)	52.8 (40.9 to 72.1)	0.7 (0.3 to 0.9)	2.6 (0.0 to 5.4)	2.7 (-1.2 to 6.2)	4.2 (-1.8 to 10.5)	2.3 (-1.3 to 6.3)	-3.4 (-8.2 to 0.2)

			Health spending per total, 2040				Per capita annualized rate of change, 2015-2040				
Location name	Total health spending per capita 2015 ($)	Total health spending per capita 2040 ($)	Government (%)	Pre-paid private (%)	Out-of-pocket (%)	Development assistance for health (%)	Total (%)	Government (%)	Pre-paid private (%)	Out-of-pocket (%)	Development assistance for health (%)
**Germany**	5532 (5366 to 5764)	6772 (5729 to 7967)	82.8 (80.2 to 85.7)	1.4 (0.8 to 2.5)	15.6 (15.1 to 16.1)	0.0 (0.0 to 0.0)	0.8 (0.2 to 1.5)	0.7 (0.0 to 1.6)	-2.5 (-6.3 to 1.4)	1.7 (0.9 to 2.5)	-
**Ghana**	242 (234 to 250)	616 (395 to 976)	53.2 (32.1 to 72.6)	2.5 (1.4 to 3.6)	34.5 (25.2 to 43.0)	8.2 (7.4 to 9.3)	3.7 (2.0 to 5.8)	5.0 (1.2 to 8.5)	2.0 (-1.9 to 5.6)	3.0 (2.2 to 3.7)	0.7 (-1.5 to 3.2)
**Greece**	2352 (2181 to 2515)	2881 (2435 to 3411)	66.5 (64.2 to 70.1)	3.9 (3.2 to 4.3)	29.2 (24.4 to 33.9)	0.0 (0.0 to 0.0)	0.8 (0.1 to 1.5)	1.0 (0.2 to 2.0)	1.2 (-0.2 to 2.4)	0.2 (-1.2 to 1.8)	-
**Grenada**	715 (671 to 773)	1272 (885 to 1801)	36.6 (23.4 to 50.1)	9.4 (6.0 to 14.1)	51.5 (48.1 to 56.2)	0.4 (0.0 to 1.9)	2.3 (0.7 to 3.8)	2.0 (-1.2 to 5.0)	4.9 (0.9 to 9.4)	2.0 (0.3 to 3.9)	-44.2 (-100.0 to 4.9)
**Guatemala**	487 (459 to 514)	694 (542 to 883)	32.7 (29.2 to 36.4)	13.9 (9.0 to 19.8)	51.9 (46.2 to 57.1)	0.6 (0.3 to 1.0)	1.4 (0.4 to 2.4)	1.5 (0.1 to 3.0)	4.6 (1.8 to 7.4)	1.4 (-0.1 to 2.8)	-9.5 (-13.2 to -6.5)
**Guinea**	102 (99 to 104)	150 (112 to 204)	23.9 (11.7 to 36.0)	5.6 (2.1 to 7.6)	41.7 (40.2 to 42.5)	26.9 (20.1 to 38.7)	1.5 (0.4 to 2.8)	4.3 (0.3 to 7.6)	5.1 (-0.1 to 8.0)	1.6 (0.5 to 2.7)	-0.6 (-2.8 to 2.2)
**Guinea-Bissau**	121 (117 to 129)	139 (92 to 223)	23.4 (8.8 to 50.0)	2.8 (1.8 to 3.7)	37.3 (32.5 to 38.9)	33.6 (28.6 to 38.7)	0.4 (-1.2 to 2.5)	-0.2 (-5.1 to 5.6)	2.5 (-2.0 to 7.0)	1.0 (-0.4 to 2.4)	-0.4 (-2.5 to 2.2)
**Guyana**	318 (298 to 335)	630 (327 to 1051)	47.1 (39.9 to 52.6)	0.1 (0.1 to 0.2)	48.8 (34.7 to 64.3)	0.6 (0.0 to 1.5)	2.6 (0.1 to 4.9)	2.1 (-0.9 to 5.0)	5.3 (3.3 to 9.5)	3.4 (-0.3 to 7.0)	-19.4 (-100.0 to -1.4)
**Haiti**	135 (130 to 140)	183 (130 to 270)	11.4 (6.4 to 15.6)	3.6 (1.8 to 6.5)	25.7 (23.9 to 26.4)	57.4 (47.2 to 69.6)	1.1 (-0.1 to 2.8)	1.7 (-1.9 to 4.7)	0.5 (-3.8 to 4.9)	0.1 (-1.4 to 1.5)	1.5 (-0.6 to 4.0)
**Honduras**	370 (351 to 397)	596 (411 to 856)	45.2 (38.3 to 51.2)	6.5 (4.7 to 8.7)	45.7 (37.0 to 55.5)	0.9 (0.7 to 1.2)	1.9 (0.4 to 3.4)	2.3 (0.2 to 4.4)	2.8 (0.1 to 5.6)	1.4 (-0.8 to 3.8)	-4.0 (-6.5 to -1.2)
**Hungary**	2031 (1969 to 2100)	3723 (2751 to 5051)	59.7 (57.6 to 60.8)	4.0 (2.5 to 5.9)	35.0 (23.9 to 50.4)	0.0 (0.0 to 0.0)	2.4 (1.2 to 3.7)	1.9 (0.6 to 3.3)	2.1 (-1.0 to 5.2)	3.1 (0.4 to 6.1)	-
**Iceland**	4205 (4085 to 4323)	7519 (6052 to 9070)	81.2 (76.6 to 84.2)	3.2 (2.6 to 4.0)	15.3 (14.0 to 17.4)	0.0 (0.0 to 0.0)	2.3 (1.5 to 3.1)	2.4 (1.3 to 3.3)	2.0 (0.4 to 3.8)	2.0 (0.7 to 3.3)	-
**India**	236 (233 to 239)	820 (680 to 1002)	28.9 (24.6 to 32.6)	10.6 (7.8 to 13.0)	59.8 (56.7 to 63.7)	0.2 (0.1 to 0.3)	5.1 (4.3 to 6.0)	5.5 (4.1 to 7.0)	6.0 (3.9 to 7.8)	4.8 (3.8 to 5.9)	-2.3 (-4.4 to 0.2)
**Indonesia**	383 (365 to 398)	1220 (788 to 1960)	37.2 (33.8 to 38.3)	7.9 (6.5 to 8.7)	53.1 (38.1 to 70.1)	0.1 (0.1 to 0.1)	4.6 (2.9 to 6.7)	4.5 (2.8 to 6.2)	2.6 (1.3 to 3.9)	5.0 (2.0 to 8.3)	-2.9 (-5.5 to -0.3)
**Iran**	1232 (1171 to 1295)	2550 (1573 to 4239)	41.7 (31.6 to 54.2)	11.1 (5.4 to 17.8)	43.7 (33.5 to 51.0)	0.0 (0.0 to 0.0)	2.8 (1.0 to 5.1)	2.2 (-0.8 to 5.5)	4.3 (-0.3 to 8.8)	2.8 (-0.1 to 5.8)	-92.2 (-100.0 to -10.0)
**Iraq**	562 (502 to 644)	1228 (636 to 2100)	42.2 (26.8 to 55.7)	0.1 (0.1 to 0.2)	54.2 (47.8 to 61.6)	0.0 (0.0 to 0.1)	3.0 (0.5 to 5.3)	3.1 (-1.2 to 6.8)	9.2 (4.7 to 13.4)	2.7 (-0.3 to 5.7)	-4.7 (-8.1 to -1.0)
**Ireland**	5371 (5146 to 5576)	10189 (7296 to 13783)	66.2 (55.5 to 75.1)	12.7 (9.9 to 16.3)	20.2 (19.1 to 22.6)	0.0 (0.0 to 0.0)	2.5 (1.2 to 3.8)	2.2 (0.1 to 4.0)	2.5 (0.2 to 4.9)	3.9 (2.9 to 4.9)	-
**Israel**	2560 (2417 to 2745)	3643 (3062 to 4287)	59.4 (56.8 to 62.1)	19.2 (13.4 to 27.3)	21.0 (20.6 to 21.2)	0.0 (0.0 to 0.0)	1.4 (0.7 to 2.1)	1.0 (0.1 to 1.9)	3.5 (1.4 to 5.8)	1.0 (-0.1 to 2.1)	-
**Italy**	3445 (3357 to 3526)	4412 (3736 to 5146)	78.0 (75.4 to 80.3)	3.4 (2.4 to 4.1)	18.3 (16.5 to 20.5)	0.0 (0.0 to 0.0)	1.0 (0.3 to 1.6)	1.1 (0.3 to 1.9)	2.8 (0.6 to 4.3)	0.1 (-1.0 to 1.2)	-

			Health spending per total, 2040				Per capita annualized rate of change, 2015-2040				
Location name	Total health spending per capita 2015 ($)	Total health spending per capita 2040 ($)	Government (%)	Pre-paid private (%)	Out-of-pocket (%)	Development assistance for health (%)	Total (%)	Government (%)	Pre-paid private (%)	Out-of-pocket (%)	Development assistance for health (%)
**Jamaica**	510 (479 to 542)	549 (370 to 800)	51.4 (35.8 to 65.3)	19.7 (18.2 to 21.7)	26.9 (25.2 to 27.6)	0.1 (0.0 to 0.4)	0.2 (-1.3 to 1.8)	-0.3 (-3.2 to 2.4)	1.1 (-0.7 to 3.1)	0.5 (-1.3 to 2.3)	-42.4 (-100.0 to -3.6)
**Japan**	4286 (4163 to 4465)	5052 (4185 to 6182)	83.0 (80.4 to 85.9)	0.0 (0.0 to 0.1)	16.7 (16.0 to 17.4)	0.0 (0.0 to 0.0)	0.6 (-0.1 to 1.5)	0.5 (-0.4 to 1.5)	8.5 (4.2 to 13.3)	1.6 (0.7 to 2.5)	-
**Jordan**	730 (687 to 774)	1104 (708 to 1606)	60.6 (53.1 to 64.8)	8.8 (4.4 to 17.0)	27.9 (17.6 to 43.0)	0.2 (0.1 to 0.3)	1.6 (-0.1 to 3.1)	1.3 (-0.9 to 3.1)	0.6 (-3.7 to 5.2)	2.1 (-1.4 to 5.8)	-5.3 (-10.9 to -1.7)
**Kazakhstan**	1017 (997 to 1040)	1945 (1154 to 2921)	57.4 (50.3 to 66.3)	0.6 (0.5 to 0.7)	39.5 (27.0 to 49.4)	0.1 (0.0 to 0.2)	2.5 (0.5 to 4.3)	2.2 (-0.3 to 4.6)	1.5 (0.3 to 2.7)	2.7 (-0.8 to 5.5)	-18.4 (-100.0 to 3.6)
**Kenya**	187 (185 to 190)	310 (259 to 371)	28.9 (25.8 to 32.1)	16.3 (13.9 to 19.1)	30.2 (29.4 to 31.7)	23.8 (16.7 to 35.3)	2.0 (1.3 to 2.8)	1.8 (0.6 to 3.0)	2.9 (1.6 to 4.4)	2.0 (1.2 to 3.0)	1.5 (-0.5 to 4.0)
**Kiribati**	189 (171 to 212)	339 (255 to 469)	64.2 (54.1 to 71.9)	0.0 (0.0 to 0.1)	3.2 (2.1 to 4.2)	31.7 (14.1 to 50.5)	2.3 (1.0 to 3.7)	1.6 (0.7 to 2.5)	2.9 (0.7 to 6.9)	0.8 (-2.5 to 4.1)	4.3 (0.0 to 7.9)
**Kuwait**	2640 (2425 to 2869)	2681 (1135 to 4757)	70.7 (47.1 to 83.8)	1.4 (1.4 to 1.6)	24.9 (23.9 to 29.3)	0.0 (0.0 to 0.0)	-0.2 (-3.3 to 2.4)	-0.8 (-5.5 to 2.4)	-0.8 (-4.1 to 2.4)	1.8 (-0.8 to 4.5)	-
**Kyrgyzstan**	308 (293 to 331)	504 (287 to 825)	36.6 (27.0 to 42.3)	1.2 (0.5 to 2.4)	57.6 (45.9 to 70.6)	1.6 (1.3 to 2.1)	1.9 (-0.2 to 4.0)	1.1 (-2.2 to 4.0)	2.2 (-7.0 to 13.4)	2.7 (-0.3 to 5.9)	-4.4 (-7.2 to -1.5)
**Laos**	178 (167 to 195)	513 (326 to 746)	50.8 (44.2 to 58.2)	4.9 (2.0 to 10.3)	34.7 (23.3 to 49.3)	6.8 (6.2 to 8.3)	4.2 (2.4 to 6.0)	5.9 (3.4 to 8.3)	6.4 (1.0 to 11.8)	3.1 (-0.2 to 6.4)	0.2 (-1.9 to 2.7)
**Latvia**	1683 (1593 to 1771)	3266 (2412 to 4335)	57.3 (53.3 to 61.7)	1.0 (0.4 to 2.2)	40.6 (32.5 to 48.6)	0.0 (0.0 to 0.0)	2.6 (1.4 to 3.9)	2.4 (0.9 to 3.9)	2.8 (-2.6 to 8.7)	3.0 (0.9 to 5.0)	-
**Lebanon**	1207 (1102 to 1312)	1359 (738 to 2257)	50.3 (27.8 to 71.0)	15.3 (15.1 to 15.8)	30.6 (29.1 to 32.3)	0.4 (0.0 to 0.5)	0.3 (-1.9 to 2.6)	0.1 (-4.3 to 4.0)	0.1 (-2.1 to 2.4)	0.1 (-2.4 to 2.8)	-4.2 (-100.0 to 3.3)
**Lesotho**	262 (254 to 270)	641 (462 to 885)	47.2 (40.0 to 51.2)	0.9 (0.6 to 1.2)	12.4 (11.1 to 13.5)	38.3 (31.0 to 51.8)	3.6 (2.3 to 5.0)	3.1 (1.1 to 4.8)	-0.1 (-2.6 to 2.4)	2.3 (1.4 to 3.2)	4.9 (2.8 to 7.7)
**Liberia**	481 (474 to 488)	340 (214 to 582)	5.1 (2.3 to 7.4)	0.6 (0.3 to 1.1)	10.8 (8.4 to 12.4)	81.9 (76.8 to 89.2)	-1.5 (-3.2 to 0.8)	1.5 (-3.1 to 5.7)	1.9 (-4.0 to 7.8)	1.1 (-1.8 to 4.1)	-2.0 (-3.9 to 0.8)
**Libya**	502 (435 to 582)	798 (499 to 1225)	60.7 (53.8 to 74.1)	16.6 (9.1 to 24.1)	19.3 (13.1 to 28.3)	0.3 (0.1 to 0.5)	1.8 (-0.1 to 3.7)	2.5 (0.2 to 5.3)	4.0 (-0.6 to 8.1)	-1.1 (-4.7 to 2.4)	4.1 (-0.7 to 7.8)
**Lithuania**	1941 (1872 to 2010)	4026 (2987 to 5256)	59.1 (48.8 to 67.7)	0.8 (0.5 to 1.3)	39.4 (39.1 to 40.0)	0.0 (0.0 to 0.0)	2.9 (1.7 to 4.1)	2.4 (0.5 to 4.1)	1.9 (-1.3 to 5.4)	3.7 (2.6 to 4.9)	-
**Luxembourg**	6530 (6288 to 6784)	11363 (8802 to 14095)	78.8 (75.3 to 81.9)	7.5 (5.1 to 11.0)	13.1 (10.8 to 16.0)	0.0 (0.0 to 0.0)	2.2 (1.2 to 3.2)	2.0 (0.7 to 3.1)	3.2 (0.7 to 5.9)	3.0 (1.2 to 4.9)	-
**Macedonia**	921 (758 to 1196)	1319 (878 to 1851)	53.1 (51.8 to 54.9)	4.8 (3.2 to 7.4)	40.4 (27.4 to 56.3)	0.0 (0.0 to 0.1)	1.4 (-0.4 to 3.1)	0.8 (-1.2 to 2.9)	4.0 (-1.2 to 9.5)	1.9 (-1.1 to 5.0)	-65.6 (-100.0 to -5.5)
**Madagascar**	78 (74 to 81)	112 (70 to 161)	58.5 (38.1 to 71.0)	5.7 (4.6 to 7.3)	21.2 (20.5 to 22.7)	12.7 (11.6 to 16.0)	1.4 (-0.5 to 3.0)	2.6 (-0.9 to 5.1)	0.8 (-1.8 to 3.4)	1.1 (-0.4 to 2.7)	-1.8 (-3.8 to 0.7)
**Malawi**	135 (132 to 138)	179 (119 to 278)	15.8 (8.2 to 23.1)	6.0 (2.7 to 10.0)	7.0 (4.2 to 10.2)	68.6 (61.9 to 77.6)	1.0 (-0.5 to 2.9)	0.1 (-4.0 to 3.8)	1.8 (-2.9 to 6.6)	0.3 (-3.2 to 3.8)	1.1 (-0.9 to 3.5)

			Health spending per total, 2040				Per capita annualized rate of change, 2015-2040				
Location name	Total health spending per capita 2015 ($)	Total health spending per capita 2040 ($)	Government (%)	Pre-paid private (%)	Out-of-pocket (%)	Development assistance for health (%)	Total (%)	Government (%)	Pre-paid private (%)	Out-of-pocket (%)	Development assistance for health (%)
**Malaysia**	1072 (1041 to 1105)	2587 (1920 to 3459)	42.8 (38.7 to 45.8)	8.2 (7.9 to 9.1)	47.9 (38.8 to 57.4)	0.0 (0.0 to 0.0)	3.5 (2.3 to 4.8)	2.7 (1.0 to 4.1)	2.4 (1.0 to 4.1)	4.7 (2.6 to 6.8)	-61.0 (-100.0 to -4.0)
**Maldives**	1850 (1719 to 1990)	2465 (1533 to 3684)	79.7 (72.1 to 83.2)	1.2 (0.7 to 2.0)	17.2 (10.1 to 28.0)	0.2 (0.0 to 0.5)	1.1 (-0.9 to 2.9)	1.0 (-1.3 to 3.0)	-0.3 (-4.4 to 4.0)	0.8 (-3.1 to 4.6)	-24.0 (-100.0 to 7.3)
**Mali**	110 (105 to 115)	211 (151 to 287)	22.8 (9.6 to 39.2)	10.4 (4.0 to 15.7)	40.2 (37.6 to 43.0)	24.1 (20.1 to 33.2)	2.6 (1.2 to 4.0)	3.9 (-0.7 to 7.9)	5.7 (0.6 to 9.1)	1.9 (0.4 to 3.6)	1.4 (-0.6 to 4.1)
**Malta**	3642 (3494 to 3766)	8586 (7170 to 10252)	63.9 (61.3 to 65.3)	2.7 (2.1 to 3.4)	33.0 (27.6 to 40.1)	0.0 (0.0 to 0.0)	3.5 (2.7 to 4.3)	3.7 (2.8 to 4.5)	4.5 (2.7 to 6.3)	3.0 (1.5 to 4.7)	-
**Marshall Islands**	604 (565 to 646)	615 (319 to 1015)	68.8 (45.9 to 82.4)	3.0 (2.9 to 3.2)	23.9 (22.3 to 27.3)	1.7 (0.0 to 5.1)	-0.1 (-2.6 to 2.1)	0.1 (-4.0 to 3.1)	-0.5 (-2.9 to 1.7)	2.4 (0.4 to 4.7)	-20.9 (-100.0 to -2.9)
**Mauritania**	184 (174 to 194)	261 (152 to 428)	44.2 (28.4 to 64.2)	5.7 (2.6 to 8.4)	44.8 (40.7 to 46.1)	1.9 (0.9 to 2.6)	1.3 (-0.7 to 3.5)	1.7 (-2.1 to 5.6)	2.4 (-2.9 to 6.6)	1.0 (-1.4 to 3.2)	-4.6 (-9.2 to -1.2)
**Mauritius**	1094 (1047 to 1137)	3148 (1893 to 4947)	34.8 (31.9 to 36.8)	0.6 (0.5 to 0.7)	63.1 (48.9 to 76.6)	0.0 (0.0 to 0.1)	4.2 (2.2 to 6.2)	3.0 (1.3 to 4.7)	2.6 (1.3 to 4.2)	4.9 (1.9 to 7.8)	-53.9 (-100.0 to 4.8)
**Mexico**	1081 (1050 to 1112)	1852 (1472 to 2301)	47.7 (46.9 to 49.1)	11.8 (7.2 to 16.8)	39.6 (32.6 to 48.6)	0.0 (0.0 to 0.0)	2.2 (1.2 to 3.1)	1.8 (0.8 to 2.8)	4.6 (1.7 to 7.2)	2.0 (0.3 to 3.7)	-22.2 (-100.0 to -2.7)
**Moldova**	543 (516 to 574)	822 (508 to 1231)	48.8 (43.1 to 51.4)	0.8 (0.5 to 1.1)	41.0 (26.5 to 56.1)	6.9 (5.5 to 8.9)	1.6 (-0.2 to 3.4)	1.8 (-0.6 to 3.8)	0.8 (-2.7 to 4.1)	1.1 (-2.4 to 4.3)	1.2 (-1.5 to 4.1)
**Mongolia**	496 (475 to 522)	1293 (713 to 2195)	38.8 (33.0 to 41.2)	2.1 (2.0 to 2.3)	52.3 (32.7 to 72.6)	3.3 (3.2 to 3.6)	3.7 (1.4 to 6.1)	2.6 (-0.3 to 5.1)	2.0 (-0.6 to 4.8)	4.9 (0.7 to 8.8)	1.2 (-1.2 to 3.8)
**Montenegro**	985 (954 to 1017)	1472 (1194 to 1774)	63.5 (61.2 to 66.2)	0.5 (0.3 to 0.8)	34.5 (29.8 to 40.6)	1.0 (0.2 to 2.3)	1.6 (0.7 to 2.4)	1.4 (0.4 to 2.4)	1.6 (-1.8 to 5.3)	1.8 (0.4 to 3.4)	3.3 (-2.1 to 9.1)
**Morocco**	454 (438 to 472)	1056 (788 to 1406)	43.1 (38.2 to 53.4)	1.6 (1.0 to 2.2)	50.4 (44.9 to 54.7)	4.0 (3.1 to 5.3)	3.4 (2.2 to 4.7)	3.4 (1.6 to 5.6)	0.8 (-2.3 to 3.5)	3.2 (1.5 to 4.8)	9.2 (7.0 to 11.9)
**Mozambique**	72 (71 to 74)	159 (107 to 235)	26.7 (16.4 to 40.4)	4.0 (3.6 to 4.5)	9.9 (9.1 to 10.1)	57.1 (51.2 to 68.3)	3.1 (1.6 to 4.9)	5.5 (2.0 to 9.2)	3.4 (1.5 to 5.8)	4.9 (3.0 to 6.8)	2.0 (0.0 to 4.5)
**Myanmar**	301 (270 to 339)	1185 (755 to 1804)	26.2 (25.2 to 26.8)	1.3 (1.2 to 1.3)	66.8 (53.6 to 79.3)	4.3 (4.0 to 5.2)	5.5 (3.7 to 7.5)	6.3 (4.2 to 8.3)	5.0 (3.1 to 6.7)	5.3 (2.5 to 8.1)	4.7 (2.5 to 7.5)
**Namibia**	1033 (991 to 1084)	1260 (941 to 1654)	61.3 (57.5 to 66.0)	15.6 (9.1 to 24.2)	12.3 (6.4 to 20.7)	9.1 (6.4 to 13.9)	0.8 (-0.4 to 1.9)	0.6 (-0.8 to 2.1)	-0.4 (-3.6 to 2.6)	2.1 (-1.5 to 5.7)	1.2 (-1.3 to 4.1)
**Nepal**	160 (153 to 167)	292 (185 to 479)	18.7 (16.9 to 19.7)	16.2 (13.3 to 18.8)	56.7 (41.6 to 70.6)	6.0 (5.5 to 6.7)	2.3 (0.6 to 4.5)	2.7 (0.5 to 4.7)	4.0 (1.4 to 6.9)	2.2 (-0.7 to 5.3)	-1.2 (-3.3 to 1.3)
**Netherlands**	5579 (5360 to 5835)	7202 (5793 to 8840)	85.6 (84.6 to 86.4)	3.1 (2.3 to 4.3)	10.8 (5.4 to 19.9)	0.0 (0.0 to 0.0)	1.0 (0.1 to 1.9)	1.2 (0.4 to 2.1)	-2.4 (-4.3 to -0.2)	0.3 (-3.1 to 3.9)	-
**New Zealand**	3648 (3481 to 3856)	5001 (4201 to 5884)	78.2 (75.1 to 81.4)	8.7 (7.3 to 10.4)	12.9 (12.6 to 13.5)	0.0 (0.0 to 0.0)	1.3 (0.6 to 2.0)	1.2 (0.3 to 2.1)	1.9 (0.5 to 3.5)	1.4 (0.5 to 2.2)	-
**Nicaragua**	432 (413 to 454)	651 (482 to 871)	53.7 (44.0 to 64.4)	2.4 (1.6 to 3.5)	36.9 (36.1 to 37.6)	6.0 (4.6 to 8.5)	1.6 (0.4 to 2.9)	1.5 (-0.5 to 3.6)	1.8 (-1.1 to 4.7)	1.9 (0.7 to 3.1)	0.0 (-2.1 to 2.6)

			Health spending per total, 2040				Per capita annualized rate of change, 2015-2040				
Location name	Total health spending per capita 2015 ($)	Total health spending per capita 2040 ($)	Government (%)	Pre-paid private (%)	Out-of-pocket (%)	Development assistance for health (%)	Total (%)	Government (%)	Pre-paid private (%)	Out-of-pocket (%)	Development assistance for health (%)
**Niger**	67 (65 to 69)	97 (71 to 125)	33.8 (22.7 to 42.8)	1.4 (1.1 to 1.8)	50.3 (48.1 to 52.9)	13.2 (10.5 to 18.3)	1.4 (0.2 to 2.5)	2.6 (-0.2 to 4.7)	1.3 (-0.9 to 3.8)	1.1 (-0.3 to 2.4)	0.0 (-2.1 to 2.5)
**Nigeria**	216 (201 to 234)	332 (217 to 509)	12.3 (1.9 to 37.7)	0.9 (0.8 to 0.9)	77.9 (74.9 to 79.1)	6.8 (6.1 to 8.3)	1.7 (0.0 to 3.5)	-0.3 (-8.2 to 7.2)	-1.2 (-3.3 to 0.7)	1.9 (0.2 to 3.7)	0.7 (-1.3 to 3.4)
**North Korea**	134 (128 to 139)	114 (103 to 125)	41.4 (41.2 to 41.8)	3.1 (2.1 to 4.4)	53.0 (50.1 to 56.2)	2.3 (1.4 to 3.7)	-0.6 (-1.0 to -0.2)	-0.3 (-0.8 to 0.1)	-3.0 (-5.4 to -0.8)	-0.8 (-1.4 to -0.2)	4.0 (1.8 to 6.5)
**Norway**	7024 (6810 to 7268)	8101 (5263 to 11180)	84.5 (77.1 to 89.1)	0.4 (0.4 to 0.5)	14.8 (13.4 to 17.3)	0.0 (0.0 to 0.0)	0.5 (-1.2 to 1.9)	0.5 (-1.6 to 2.1)	0.9 (-0.1 to 1.7)	0.6 (-0.4 to 1.6)	-
**Oman**	1684 (1555 to 1799)	2492 (1411 to 3922)	78.5 (68.6 to 86.2)	3.8 (3.1 to 4.8)	15.7 (12.0 to 20.4)	0.0 (0.0 to 0.0)	1.4 (-0.7 to 3.5)	0.9 (-1.7 to 3.4)	0.2 (-2.6 to 3.2)	5.1 (1.8 to 8.5)	-
**Pakistan**	142 (136 to 150)	305 (210 to 442)	32.9 (21.1 to 44.9)	2.0 (1.8 to 2.1)	57.8 (52.3 to 62.5)	5.6 (4.7 to 7.0)	3.0 (1.6 to 4.6)	3.9 (0.7 to 6.8)	2.5 (1.1 to 3.8)	2.6 (0.7 to 4.5)	2.0 (-0.2 to 4.6)
**Palestine**	390 (345 to 435)	662 (501 to 881)	43.9 (39.8 to 50.1)	18.7 (13.1 to 24.5)	36.0 (29.6 to 41.7)	0.0 (0.0 to 0.1)	2.1 (0.9 to 3.4)	2.5 (1.0 to 4.2)	2.0 (-0.7 to 4.7)	1.6 (-0.4 to 3.7)	-45.6 (-100.0 to -6.3)
**Panama**	1588 (1535 to 1649)	3944 (2964 to 5076)	58.9 (48.9 to 67.0)	10.0 (7.6 to 12.8)	30.2 (29.8 to 30.5)	0.0 (0.0 to 0.0)	3.7 (2.5 to 4.8)	3.5 (1.6 to 5.2)	5.2 (2.9 to 7.4)	3.6 (2.5 to 4.7)	-37.7 (-100.0 to -7.7)
**Papua New Guinea**	121 (114 to 131)	145 (94 to 211)	87.1 (80.8 to 91.2)	0.0 (0.0 to 0.1)	8.1 (5.6 to 11.8)	3.9 (2.4 to 5.4)	0.6 (-1.0 to 2.3)	1.3 (-0.7 to 3.1)	16.2 (11.1 to 32.1)	2.0 (-1.1 to 5.3)	-5.8 (-9.1 to -2.9)
**Paraguay**	738 (706 to 777)	1838 (1283 to 2497)	59.4 (50.6 to 67.2)	10.5 (5.1 to 20.6)	28.8 (28.2 to 29.9)	0.0 (0.0 to 0.0)	3.7 (2.2 to 5.0)	4.1 (2.0 to 6.0)	3.8 (-0.4 to 8.2)	2.7 (1.5 to 3.9)	-53.4 (-100.0 to -4.9)
**Peru**	683 (669 to 698)	1302 (937 to 1729)	62.7 (54.6 to 71.6)	5.5 (4.0 to 7.3)	30.8 (29.3 to 32.2)	0.0 (0.0 to 0.1)	2.6 (1.3 to 3.8)	2.8 (0.9 to 4.6)	1.7 (-0.8 to 4.2)	2.5 (1.0 to 4.0)	-41.1 (-100.0 to -10.4)
**Philippines**	333 (324 to 347)	1003 (737 to 1355)	23.6 (20.5 to 26.2)	16.8 (14.1 to 19.6)	58.0 (48.5 to 67.9)	0.3 (0.2 to 0.4)	4.5 (3.3 to 5.8)	3.5 (1.8 to 5.3)	5.0 (3.1 to 7.1)	4.8 (2.9 to 6.8)	-4.1 (-6.2 to -1.6)
**Poland**	1757 (1671 to 1837)	3557 (2938 to 4341)	65.8 (61.4 to 70.0)	9.2 (5.8 to 11.9)	24.6 (24.3 to 25.0)	0.0 (0.0 to 0.0)	2.8 (2.1 to 3.7)	2.5 (1.4 to 3.6)	5.4 (2.5 to 8.1)	3.0 (2.3 to 3.7)	-
**Portugal**	2712 (2621 to 2819)	4606 (3796 to 5451)	62.7 (58.6 to 66.2)	11.9 (8.7 to 16.7)	24.9 (22.9 to 27.6)	0.0 (0.0 to 0.0)	2.1 (1.3 to 2.9)	1.9 (0.9 to 2.9)	4.8 (2.7 to 7.0)	1.7 (0.5 to 2.8)	-
**Qatar**	3251 (3050 to 3450)	4130 (1964 to 8493)	67.1 (45.0 to 85.6)	8.5 (7.4 to 8.9)	19.7 (13.9 to 22.8)	0.0 (0.0 to 0.0)	0.6 (-2.1 to 3.9)	-0.3 (-4.4 to 4.1)	0.5 (-2.7 to 3.7)	4.9 (0.5 to 9.4)	-
**Romania**	1128 (1051 to 1198)	3085 (2067 to 4733)	77.4 (69.8 to 83.7)	0.6 (0.6 to 0.6)	21.1 (19.8 to 22.3)	0.0 (0.0 to 0.0)	4.0 (2.5 to 5.9)	4.0 (2.0 to 6.1)	3.3 (1.2 to 5.5)	4.0 (2.1 to 6.0)	-59.1 (-100.0 to 3.8)
**Russia**	1544 (1523 to 1564)	2142 (1487 to 2970)	52.8 (44.3 to 60.0)	1.4 (1.0 to 2.0)	44.4 (37.9 to 51.5)	0.0 (0.0 to 0.0)	1.3 (-0.1 to 2.7)	0.6 (-1.4 to 2.5)	-1.3 (-4.1 to 1.5)	2.1 (0.1 to 4.2)	-26.5 (-100.0 to -2.5)
**Rwanda**	149 (143 to 155)	348 (258 to 465)	29.2 (21.5 to 38.0)	9.1 (4.4 to 16.0)	38.2 (31.6 to 45.9)	21.3 (16.0 to 29.2)	3.4 (2.2 to 4.6)	4.1 (1.6 to 6.6)	3.4 (-0.6 to 7.3)	5.0 (3.0 to 7.1)	0.7 (-1.5 to 3.3)
**Saint Lucia**	714 (658 to 793)	1093 (790 to 1501)	33.7 (22.5 to 47.7)	4.0 (2.9 to 4.6)	51.2 (49.3 to 52.5)	9.2 (0.8 to 13.7)	1.7 (0.4 to 3.1)	0.9 (-1.9 to 3.8)	1.2 (-1.4 to 3.1)	1.7 (0.2 to 3.2)	2.7 (-6.4 to 7.8)

			Health spending per total, 2040				Per capita annualized rate of change, 2015-2040				
Location name	Total health spending per capita 2015 ($)	Total health spending per capita 2040 ($)	Government (%)	Pre-paid private (%)	Out-of-pocket (%)	Development assistance for health (%)	Total (%)	Government (%)	Pre-paid private (%)	Out-of-pocket (%)	Development assistance for health (%)
**Saint Vincent and the Grenadines**	523 (506 to 537)	831 (587 to 1161)	56.9 (48.8 to 66.2)	2.4 (2.2 to 2.7)	16.6 (16.3 to 16.9)	22.5 (14.5 to 32.8)	1.8 (0.5 to 3.3)	1.2 (-0.7 to 3.2)	2.3 (0.4 to 4.3)	1.2 (-0.3 to 2.8)	4.1 (1.0 to 7.3)
**Samoa**	342 (319 to 364)	577 (308 to 1006)	52.4 (28.3 to 73.2)	0.8 (0.7 to 0.9)	10.3 (8.9 to 11.6)	32.9 (31.8 to 34.5)	1.9 (-0.5 to 4.3)	0.8 (-3.9 to 4.7)	2.1 (0.0 to 3.9)	1.9 (0.0 to 3.9)	3.7 (1.5 to 6.5)
**Sao Tome and Principe**	216 (206 to 225)	305 (151 to 619)	49.5 (26.8 to 77.6)	1.8 (0.8 to 2.1)	20.4 (15.2 to 23.6)	22.3 (16.2 to 24.1)	1.1 (-1.4 to 4.3)	1.2 (-3.8 to 6.3)	0.7 (-5.2 to 4.7)	1.7 (-2.0 to 5.3)	-0.4 (-4.2 to 2.7)
**Saudi Arabia**	3138 (2975 to 3318)	4248 (2203 to 8072)	66.7 (51.6 to 84.2)	15.0 (12.0 to 16.9)	15.0 (13.8 to 15.8)	0.0 (0.0 to 0.0)	1.0 (-1.4 to 3.9)	0.7 (-2.7 to 4.5)	1.5 (-1.8 to 4.6)	1.0 (-1.6 to 3.7)	-
**Senegal**	119 (113 to 123)	190 (149 to 234)	38.2 (28.4 to 46.4)	14.2 (8.2 to 23.5)	37.6 (36.7 to 38.8)	8.8 (6.4 to 12.7)	1.9 (0.9 to 2.8)	3.3 (1.1 to 5.1)	2.9 (-0.1 to 6.1)	2.0 (0.8 to 3.1)	-2.5 (-4.5 to -0.1)
**Serbia**	1398 (1349 to 1459)	2653 (1951 to 3683)	62.2 (56.3 to 66.0)	0.8 (0.4 to 1.6)	35.2 (21.3 to 50.0)	0.8 (0.6 to 1.0)	2.5 (1.3 to 4.0)	2.8 (1.8 to 3.8)	0.0 (-4.3 to 4.9)	1.9 (-1.2 to 4.9)	10.0 (7.6 to 12.9)
**Seychelles**	957 (870 to 1057)	1911 (646 to 3945)	96.6 (91.2 to 98.9)	0.4 (0.3 to 0.6)	2.1 (1.4 to 3.7)	0.0 (0.0 to 0.0)	2.4 (-1.7 to 5.9)	2.4 (-1.9 to 6.0)	7.4 (4.9 to 9.9)	1.6 (-4.3 to 8.2)	-70.5 (-71.1 to -69.8)
**Sierra Leone**	248 (232 to 260)	241 (172 to 342)	13.0 (5.7 to 22.7)	10.9 (4.3 to 21.2)	36.2 (34.9 to 37.0)	37.2 (30.2 to 50.6)	-0.2 (-1.5 to 1.3)	0.9 (-3.6 to 5.1)	3.1 (-1.4 to 7.8)	-1.2 (-2.7 to 0.3)	-0.4 (-2.4 to 2.4)
**Singapore**	3657 (3529 to 3810)	5212 (3658 to 7186)	49.3 (37.4 to 61.6)	17.3 (16.5 to 18.2)	32.1 (29.7 to 33.0)	0.0 (0.0 to 0.0)	1.4 (0.0 to 2.7)	1.1 (-1.3 to 3.4)	1.5 (0.0 to 3.1)	1.4 (-0.3 to 2.9)	-
**Slovakia**	2216 (2085 to 2350)	4680 (3291 to 6354)	72.1 (67.6 to 77.2)	3.3 (2.2 to 5.2)	23.5 (16.9 to 32.5)	0.0 (0.0 to 0.0)	3.0 (1.6 to 4.3)	2.6 (0.9 to 4.2)	4.8 (-0.1 to 10.8)	3.9 (1.1 to 6.8)	-
**Slovenia**	2806 (2744 to 2884)	5093 (4094 to 6378)	66.3 (64.4 to 67.0)	21.2 (11.9 to 34.2)	11.8 (9.9 to 13.4)	0.0 (0.0 to 0.0)	2.4 (1.5 to 3.4)	2.1 (1.1 to 3.0)	3.4 (0.2 to 6.4)	2.1 (0.6 to 3.6)	-
**Solomon Islands**	157 (144 to 166)	262 (151 to 425)	62.9 (47.8 to 75.5)	0.2 (0.2 to 0.2)	4.0 (3.6 to 4.4)	30.2 (27.0 to 38.5)	1.9 (-0.1 to 4.1)	1.8 (-1.4 to 4.7)	1.3 (-1.2 to 3.5)	2.6 (0.0 to 5.3)	1.7 (-0.7 to 4.9)
**Somalia**	42 (42 to 43)	89 (61 to 147)	6.5 (4.7 to 7.5)	1.3 (1.0 to 1.4)	18.1 (11.5 to 23.2)	73.6 (63.5 to 84.8)	2.9 (1.5 to 5.1)	0.3 (-0.5 to 1.3)	-0.1 (-1.3 to 1.0)	-0.1 (-0.6 to 0.2)	4.8 (2.7 to 7.6)
**South Africa**	1109 (1091 to 1128)	1220 (995 to 1484)	60.0 (55.3 to 64.4)	24.1 (19.9 to 28.9)	10.1 (9.2 to 10.2)	5.2 (3.8 to 7.9)	0.4 (-0.4 to 1.2)	0.8 (-0.3 to 1.9)	-1.3 (-2.8 to 0.3)	1.4 (0.2 to 2.2)	3.6 (1.5 to 6.3)
**South Korea**	2835 (2785 to 2884)	6565 (4753 to 8976)	51.5 (49.1 to 52.8)	8.5 (5.0 to 11.7)	38.6 (32.5 to 50.7)	0.0 (0.0 to 0.0)	3.4 (2.1 to 4.7)	3.0 (1.6 to 4.3)	4.2 (0.8 to 7.0)	3.5 (1.6 to 6.1)	-
**South Sudan**	81 (79 to 84)	127 (103 to 157)	46.2 (38.6 to 53.7)	2.6 (2.5 to 2.8)	35.6 (32.2 to 38.5)	14.9 (11.0 to 22.1)	1.8 (1.0 to 2.7)	3.9 (2.2 to 5.4)	-0.2 (-1.2 to 1.0)	-0.2 (-0.7 to 0.3)	3.1 (1.1 to 5.7)
**Spain**	3363 (3262 to 3450)	5411 (4516 to 6353)	71.7 (67.2 to 75.1)	5.4 (4.9 to 5.9)	22.7 (21.3 to 24.1)	0.0 (0.0 to 0.0)	1.9 (1.2 to 2.6)	1.9 (1.0 to 2.9)	2.4 (1.3 to 3.5)	1.6 (0.7 to 2.6)	-
**Sri Lanka**	360 (348 to 370)	1026 (677 to 1546)	48.0 (39.3 to 58.5)	4.9 (4.4 to 5.0)	44.6 (37.7 to 52.4)	0.6 (0.5 to 0.8)	4.2 (2.6 to 6.0)	3.7 (1.2 to 6.4)	2.9 (0.8 to 4.8)	5.0 (2.7 to 7.6)	-2.9 (-4.6 to 0.5)
**Sudan**	282 (262 to 306)	427 (223 to 731)	26.8 (17.6 to 38.1)	2.6 (2.5 to 2.8)	61.8 (49.4 to 76.2)	4.9 (4.6 to 5.4)	1.5 (-0.9 to 3.9)	1.0 (-3.1 to 4.9)	0.6 (-1.6 to 2.8)	1.4 (-2.0 to 4.6)	3.6 (1.5 to 6.3)

			Health spending per total, 2040				Per capita annualized rate of change, 2015-2040				
Location name	Total health spending per capita 2015 ($)	Total health spending per capita 2040 ($)	Government (%)	Pre-paid private (%)	Out-of-pocket (%)	Development assistance for health (%)	Total (%)	Government (%)	Pre-paid private (%)	Out-of-pocket (%)	Development assistance for health (%)
**Suriname**	993 (904 to 1074)	907 (489 to 1550)	37.3 (15.4 to 64.3)	42.7 (37.3 to 44.2)	16.2 (14.8 to 17.5)	0.1 (0.0 to 0.3)	-0.5 (-2.8 to 1.9)	-2.1 (-7.4 to 2.8)	0.3 (-2.5 to 2.4)	0.9 (-1.0 to 3.1)	-43.2 (-100.0 to -6.5)
**Swaziland**	693 (661 to 729)	1210 (740 to 1808)	54.9 (40.8 to 65.5)	2.8 (2.5 to 3.1)	8.3 (8.0 to 8.9)	31.9 (29.4 to 40.4)	2.2 (0.2 to 3.9)	1.7 (-1.4 to 4.1)	-2.3 (-4.6 to -0.4)	1.1 (-0.5 to 2.7)	4.3 (2.1 to 7.0)
**Sweden**	5550 (5346 to 5748)	8004 (6432 to 9856)	80.4 (76.5 to 83.8)	1.1 (0.9 to 1.3)	18.2 (17.5 to 19.0)	0.0 (0.0 to 0.0)	1.5 (0.6 to 2.3)	1.3 (0.2 to 2.4)	1.4 (-0.3 to 2.9)	2.2 (1.2 to 3.2)	-
**Switzerland**	7465 (7252 to 7662)	7634 (6552 to 8808)	68.3 (64.3 to 71.5)	5.3 (4.2 to 6.5)	26.1 (24.8 to 27.3)	0.0 (0.0 to 0.0)	0.1 (-0.5 to 0.7)	0.0 (-0.9 to 0.8)	-0.7 (-2.3 to 0.8)	0.6 (-0.2 to 1.4)	-
**Syria**	241 (207 to 284)	261 (170 to 385)	40.1 (27.3 to 55.3)	6.5 (3.8 to 11.7)	47.8 (42.2 to 51.0)	3.0 (2.3 to 3.8)	0.2 (-1.5 to 2.0)	0.1 (-3.0 to 3.1)	0.4 (-3.8 to 4.8)	0.0 (-2.4 to 2.3)	1.1 (-1.5 to 3.9)
**Taiwan**	2535 (2513 to 2555)	3813 (2983 to 4740)	56.1 (50.9 to 64.0)	13.7 (10.7 to 17.4)	29.4 (24.7 to 33.1)	0.0 (0.0 to 0.0)	1.6 (0.7 to 2.5)	1.3 (0.0 to 2.8)	1.9 (-0.3 to 4.3)	1.9 (0.3 to 3.4)	-
**Tajikistan**	200 (192 to 209)	413 (238 to 651)	22.7 (14.6 to 29.8)	0.3 (0.3 to 0.4)	65.6 (52.9 to 78.2)	8.5 (8.0 to 9.9)	2.8 (0.7 to 4.9)	1.8 (-2.0 to 5.0)	2.8 (-2.5 to 7.6)	2.9 (-0.1 to 5.8)	3.5 (1.4 to 6.1)
**Tanzania**	161 (147 to 176)	413 (263 to 667)	51.8 (34.4 to 68.5)	1.2 (1.1 to 1.2)	25.6 (24.3 to 26.4)	19.0 (17.8 to 21.2)	3.7 (1.8 to 5.9)	5.1 (1.6 to 8.7)	1.2 (-0.8 to 3.3)	3.3 (1.3 to 5.4)	1.5 (-0.5 to 4.0)
**Thailand**	614 (588 to 643)	1180 (832 to 1658)	81.6 (75.6 to 86.4)	8.8 (7.9 to 9.8)	8.5 (4.8 to 13.1)	0.2 (0.1 to 0.2)	2.6 (1.2 to 4.1)	2.8 (1.1 to 4.5)	2.3 (0.5 to 4.3)	1.0 (-2.6 to 4.5)	-0.1 (-2.6 to 3.0)
**The Bahamas**	1818 (1713 to 1935)	2503 (1844 to 3316)	52.1 (42.4 to 63.4)	20.8 (20.0 to 21.8)	25.8 (21.0 to 31.5)	0.0 (0.0 to 0.0)	1.2 (0.1 to 2.5)	1.6 (-0.3 to 3.7)	0.7 (-0.7 to 2.2)	0.7 (-1.4 to 2.9)	-
**The Gambia**	141 (135 to 148)	215 (141 to 321)	36.3 (22.2 to 50.0)	2.3 (1.1 to 4.1)	11.4 (9.2 to 13.2)	47.5 (42.0 to 56.8)	1.6 (0.0 to 3.4)	1.7 (-1.9 to 4.9)	-1.4 (-5.6 to 2.9)	0.1 (-0.9 to 0.9)	1.9 (-0.2 to 4.4)
**Timor-Leste**	103 (96 to 112)	222 (158 to 311)	47.9 (43.2 to 50.4)	0.8 (0.4 to 1.3)	5.6 (3.1 to 10.2)	44.0 (36.3 to 56.9)	3.1 (1.7 to 4.5)	2.5 (0.6 to 4.2)	-0.7 (-4.4 to 3.3)	0.4 (-3.1 to 4.3)	4.3 (2.2 to 7.0)
**Togo**	96 (92 to 101)	162 (109 to 246)	33.0 (16.7 to 57.7)	6.3 (5.2 to 6.7)	46.3 (43.5 to 47.5)	11.8 (10.1 to 14.0)	2.0 (0.5 to 3.9)	2.4 (-1.6 to 6.8)	2.1 (-0.3 to 4.0)	1.4 (-0.3 to 3.1)	2.3 (0.2 to 4.9)
**Tonga**	241 (229 to 255)	688 (447 to 1013)	53.0 (43.0 to 59.0)	8.7 (3.9 to 16.5)	6.6 (6.2 to 7.7)	28.8 (18.6 to 43.7)	4.2 (2.5 to 5.9)	3.7 (1.2 to 5.8)	6.1 (0.9 to 11.6)	1.5 (-0.4 to 3.8)	5.2 (1.7 to 8.8)
**Trinidad and Tobago**	2024 (1917 to 2158)	2983 (1765 to 4543)	56.4 (43.8 to 69.2)	6.4 (4.3 to 10.1)	34.3 (29.6 to 41.7)	0.0 (0.0 to 0.0)	1.4 (-0.6 to 3.3)	1.6 (-1.4 to 4.4)	0.1 (-3.4 to 3.8)	1.1 (-1.4 to 3.8)	-
**Tunisia**	791 (770 to 817)	1110 (907 to 1419)	51.5 (47.7 to 62.1)	6.7 (2.4 to 13.9)	41.0 (39.1 to 42.1)	0.2 (0.0 to 0.4)	1.3 (0.6 to 2.4)	0.9 (-0.1 to 2.8)	3.7 (-1.2 to 8.8)	1.5 (0.5 to 2.4)	-14.4 (-100.0 to 3.9)
**Turkey**	1029 (989 to 1074)	2727 (1567 to 4029)	81.1 (70.7 to 86.8)	3.0 (2.6 to 3.8)	14.8 (13.9 to 16.1)	0.0 (0.0 to 0.0)	3.9 (1.6 to 5.6)	4.0 (1.2 to 6.1)	1.9 (-0.8 to 4.8)	3.3 (1.0 to 5.4)	-83.1 (-100.0 to -9.8)
**Turkmenistan**	1171 (1078 to 1281)	2793 (1203 to 5565)	25.9 (21.4 to 28.5)	4.3 (3.2 to 5.3)	65.4 (51.1 to 84.0)	0.1 (0.0 to 0.1)	3.3 (0.2 to 6.5)	3.5 (-0.4 to 6.7)	2.9 (-1.4 to 7.1)	2.9 (-1.1 to 7.2)	-2.7 (-100.0 to 4.6)
**Uganda**	159 (146 to 168)	263 (192 to 353)	13.4 (9.7 to 17.7)	13.2 (5.1 to 27.0)	41.7 (37.3 to 45.8)	29.4 (23.8 to 39.2)	2.0 (0.7 to 3.3)	1.9 (-0.6 to 4.5)	2.1 (-2.7 to 7.0)	2.2 (0.4 to 4.0)	1.3 (-0.8 to 3.7)

			Health spending per total, 2040				Per capita annualized rate of change, 2015-2040				
Location name	Total health spending per capita 2015 ($)	Total health spending per capita 2040 ($)	Government (%)	Pre-paid private (%)	Out-of-pocket (%)	Development assistance for health (%)	Total (%)	Government (%)	Pre-paid private (%)	Out-of-pocket (%)	Development assistance for health (%)
**Ukraine**	598 (575 to 624)	740 (582 to 936)	42.6 (36.8 to 49.5)	2.1 (1.4 to 2.8)	50.3 (45.1 to 54.2)	4.2 (3.1 to 6.2)	0.8 (-0.1 to 1.9)	0.3 (-1.2 to 1.9)	-1.3 (-3.6 to 1.0)	1.1 (-0.3 to 2.4)	5.2 (3.0 to 8.0)
**United Arab Emirates**	2489 (2354 to 2636)	3705 (1971 to 6299)	61.8 (43.6 to 77.3)	9.7 (9.2 to 10.9)	24.5 (16.4 to 34.2)	0.0 (0.0 to 0.0)	1.4 (-0.9 to 3.8)	0.7 (-3.0 to 4.0)	2.0 (-0.5 to 4.9)	2.6 (-1.5 to 6.6)	-
**United Kingdom**	4285 (4160 to 4409)	5348 (4100 to 6761)	77.1 (72.4 to 80.1)	5.7 (5.1 to 6.2)	16.7 (12.8 to 21.1)	0.0 (0.0 to 0.0)	0.9 (-0.2 to 1.8)	0.7 (-0.6 to 1.8)	1.5 (0.0 to 2.8)	1.4 (-0.7 to 3.4)	-
**United States**	9839 (9677 to 9983)	16362 (12281 to 19551)	61.9 (60.5 to 65.9)	28.4 (14.2 to 33.6)	9.1 (8.8 to 9.6)	0.0 (0.0 to 0.0)	2.0 (0.9 to 2.8)	2.9 (2.0 to 3.8)	0.7 (-3.0 to 2.2)	1.2 (0.2 to 2.2)	-
**Uruguay**	2038 (1943 to 2116)	3074 (2329 to 3941)	72.7 (67.7 to 76.6)	9.7 (6.1 to 14.7)	16.8 (13.4 to 20.8)	0.0 (0.0 to 0.0)	1.6 (0.5 to 2.7)	1.8 (0.4 to 3.0)	0.0 (-2.8 to 2.9)	1.7 (-0.3 to 3.7)	-70.5 (-71.1 to -69.8)
**Uzbekistan**	451 (439 to 463)	1073 (712 to 1634)	49.1 (37.6 to 56.8)	3.5 (3.1 to 3.9)	42.6 (38.4 to 52.4)	2.7 (2.5 to 3.3)	3.4 (1.9 to 5.3)	3.1 (0.4 to 5.5)	4.7 (2.5 to 7.0)	3.4 (1.4 to 6.2)	7.1 (4.9 to 9.8)
**Vanuatu**	147 (136 to 161)	140 (90 to 200)	57.6 (47.9 to 66.8)	2.3 (2.2 to 2.4)	8.7 (8.1 to 9.5)	29.4 (19.1 to 40.9)	-0.3 (-1.9 to 1.2)	-0.1 (-2.5 to 2.1)	0.0 (-1.7 to 1.7)	1.1 (-0.8 to 3.1)	-1.3 (-4.5 to 1.8)
**Venezuela**	590 (559 to 616)	462 (231 to 783)	44.1 (29.3 to 52.3)	6.0 (4.9 to 7.6)	45.6 (32.7 to 64.6)	0.0 (0.0 to 0.0)	-1.2 (-3.7 to 1.2)	-1.5 (-5.6 to 1.5)	-0.5 (-3.8 to 2.9)	-1.4 (-5.0 to 2.6)	-74.4 (-75.0 to -73.9)
**Vietnam**	320 (308 to 334)	1151 (766 to 1731)	43.3 (38.3 to 46.1)	3.0 (2.5 to 3.3)	50.5 (37.7 to 63.4)	1.3 (1.2 to 1.6)	5.2 (3.5 to 7.0)	4.9 (2.7 to 7.0)	4.8 (3.2 to 6.4)	5.4 (2.6 to 8.2)	2.0 (0.0 to 4.6)
**Yemen**	179 (157 to 199)	181 (97 to 316)	20.7 (18.7 to 21.7)	1.3 (1.1 to 1.4)	72.4 (56.3 to 84.0)	3.6 (3.4 to 3.8)	-0.1 (-2.4 to 2.5)	1.7 (-0.8 to 4.0)	0.3 (-2.1 to 2.4)	-0.5 (-3.7 to 2.8)	-2.7 (-4.7 to -0.3)
**Zambia**	241 (231 to 251)	349 (248 to 492)	29.0 (15.1 to 44.3)	6.3 (5.6 to 7.0)	29.6 (27.2 to 30.6)	33.1 (27.6 to 42.7)	1.4 (0.1 to 2.8)	1.0 (-2.9 to 4.3)	-0.6 (-2.5 to 1.3)	2.1 (0.8 to 3.2)	1.5 (-0.6 to 4.1)
**Zimbabwe**	191 (181 to 201)	194 (135 to 278)	25.1 (8.8 to 43.5)	13.9 (6.6 to 24.8)	28.9 (26.4 to 30.3)	29.2 (25.0 to 35.7)	0.0 (-1.4 to 1.6)	-0.3 (-5.5 to 3.9)	-1.1 (-5.2 to 3.0)	0.0 (-1.3 to 1.3)	0.2 (-1.8 to 2.6)

B.3 Table: Future Health Scenarios in 2030

This table contains our projection of the future health scenarios (reference, better and worse scenarios) for pooled health spending per capita, universal health coverage index and the number of lives covered by our predicted universal health care, in 2030.

	Pooled health spending per capita ($)				Universal Health Coverage index				Covered lives			
	2015 Observed	2030 Worse Scenario	2030 Reference Scenario	2030 Better Scenario	2015 Observed	2030 Worse Scenario	2030 Reference Scenario	2030 Better Scenario	2015 Observed	2030 Worse Scenario	2030 Reference Scenario	2030 Better Scenario
									**Covered lives (millions)**			
**Global**	1036 (999 to 1076)	989 (747 to 1256)	1401 (1015 to 1818)	1917 (1414 to 2468)	59.2 (58.2 to 60.1)	61.4 (58.7 to 63.5)	64.8 (61.8 to 67.0)	67.1 (64.1 to 69.5)	4325 (4250 to 4390)	5109 (4887 to 5283)	5390 (5147 to 5579)	5586 (5335 to 5782)
**World Bank Income Groups**									**Covered lives (millions)**			
**High-income**	4768 (4605 to 4941)	4775 (3755 to 5762)	6213 (4653 to 7613)	8950 (6874 to 10912)	76.8 (75.7 to 77.6)	77.8 (75.6 to 79.4)	79.9 (77.3 to 81.8)	84.5 (81.9 to 86.5)	893 (880 to 902)	942 (915 to 962)	967 (936 to 990)	1023 (992 to 1047)
**Upper-middle-income**	646 (622 to 672)	715 (488 to 1011)	1251 (850 to 1787)	1537 (1038 to 2193)	65.6 (64.5 to 66.6)	67.1 (64.0 to 69.8)	72.4 (68.9 to 75.4)	74.3 (70.8 to 77.4)	1677 (1649 to 1702)	1788 (1705 to 1860)	1929 (1838 to 2009)	1982 (1888 to 2064)
**Lower-middle-income**	113 (106 to 120)	136 (94 to 191)	205 (143 to 287)	254 (174 to 362)	50.3 (49.1 to 51.5)	55.2 (52.6 to 57.1)	58.2 (55.3 to 60.2)	59.9 (56.9 to 62.0)	1482 (1445 to 1516)	1912 (1822 to 1976)	2014 (1917 to 2085)	2074 (1971 to 2146)
**Low-income**	67 (63 to 72)	74 (44 to 122)	94 (54 to 157)	141 (80 to 238)	42.7 (41.6 to 43.9)	47.5 (44.7 to 50.5)	48.7 (45.7 to 51.8)	51.5 (48.2 to 55.0)	273 (266 to 281)	467 (439 to 497)	479 (449 to 510)	507 (474 to 540)
**GBD Super-regions**									**Covered lives (millions)**			
**Central Europe, Eastern Europe, and Central Asia**	839 (801 to 885)	918 (652 to 1261)	1096 (756 to 1534)	1677 (1175 to 2336)	63.8 (61.9 to 65.6)	67.3 (63.8 to 70.3)	68.6 (64.8 to 71.9)	72.9 (68.9 to 76.2)	263 (256 to 271)	282 (268 to 295)	288 (272 to 302)	306 (289 to 320)
**GBD high-income**	5036 (4873 to 5208)	5015 (3974 to 5988)	6538 (4929 to 7925)	9403 (7278 to 11338)	77.0 (75.8 to 77.8)	77.5 (75.5 to 79.1)	79.6 (77.1 to 81.4)	84.2 (81.7 to 86.1)	812 (800 to 821)	853 (831 to 871)	876 (849 to 896)	927 (900 to 947)
**Latin America and Caribbean**	723 (693 to 755)	721 (493 to 960)	913 (611 to 1231)	1442 (960 to 1948)	60.7 (59.5 to 61.7)	62.5 (59.6 to 64.5)	64.3 (61.2 to 66.5)	68.3 (65.0 to 70.6)	344 (337 to 349)	403 (385 to 416)	415 (395 to 429)	441 (420 to 456)
**North Africa and Middle East**	597 (560 to 638)	639 (362 to 1019)	823 (449 to 1344)	1182 (648 to 1925)	59.5 (58.5 to 60.6)	63.5 (59.8 to 67.1)	65.3 (61.2 to 69.3)	68.8 (64.5 to 72.9)	336 (330 to 342)	447 (421 to 473)	460 (432 to 489)	485 (455 to 514)
**South Asia**	74 (71 to 77)	94 (70 to 123)	167 (124 to 219)	175 (129 to 231)	48.8 (47.1 to 50.2)	54.6 (52.3 to 56.5)	58.5 (56.0 to 60.6)	59.1 (56.5 to 61.2)	820 (792 to 844)	1021 (978 to 1057)	1094 (1047 to 1133)	1105 (1056 to 1145)
**Southeast Asia, East Asia, and**	439 (423 to 457)	491 (350 to 691)	1080 (764 to 1532)	1143 (807 to 1621)	63.8 (62.7 to 64.7)	65.1 (62.4 to 67.4)	71.7 (68.7 to 74.3)	72.5 (69.5 to 75.1)	1320 (1298 to 1340)	1382 (1326 to 1432)	1522 (1459 to 1578)	1539 (1476 to 1596)
**Sub-Saharan Africa**	134 (127 to 142)	131 (84 to 204)	155 (96 to 245)	258 (160 to 407)	45.1 (43.9 to 46.3)	49.4 (45.9 to 52.8)	50.3 (46.7 to 53.9)	53.7 (49.7 to 57.5)	430 (419 to 442)	720 (670 to 770)	734 (681 to 787)	783 (725 to 839)
**Countries**									**Covered lives (thousands)**			
**Afghanistan**	39 (38 to 41)	37 (25 to 54)	45 (31 to 64)	72 (48 to 107)	30.8 (27.5 to 35.3)	37.9 (34.0 to 42.1)	38.8 (34.9 to 43.2)	41.1 (36.9 to 45.6)	996 (891 to 1141)	1849 (1662 to 2056)	1896 (1705 to 2110)	2007 (1800 to 2229)
**Albania**	383 (356 to 430)	462 (314 to 662)	704 (470 to 1021)	823 (544 to 1204)	66.2 (63.8 to 68.7)	68.8 (64.9 to 72.2)	72.7 (68.6 to 76.4)	74.2 (70.0 to 78.0)	192 (185 to 199)	201 (190 to 212)	213 (201 to 224)	217 (205 to 229)
**Algeria**	744 (715 to 769)	855 (522 to 1279)	929 (546 to 1410)	1562 (932 to 2357)	63.2 (60.6 to 65.5)	65.6 (60.5 to 71.2)	66.2 (60.6 to 72.0)	71.1 (65.3 to 77.2)	2511 (2410 to 2604)	3102 (2859 to 3366)	3128 (2866 to 3402)	3362 (3088 to 3648)
**Andorra**	5897 (5345 to 6447)	5074 (4002 to 6392)	5363 (4143 to 6909)	10977 (8622 to 13909)	81.4 (78.0 to 84.9)	77.8 (74.1 to 81.5)	78.3 (74.5 to 82.1)	86.3 (82.3 to 90.4)	6 (6 to 7)	6 (6 to 6)	6 (6 to 6)	7 (6 to 7)

**Angola**	134 (113 to 160)	148 (64 to 284)	155 (61 to 306)	306 (127 to 595)	43.7 (38.5 to 48.1)	52.0 (45.6 to 58.5)	52.2 (45.7 to 59.2)	57.0 (50.0 to 64.5)	1097 (966 to 1205)	2168 (1901 to 2442)	2177 (1907 to 2471)	2379 (2086 to 2689)
**Antigua and Barbuda**	921 (860 to 975)	1048 (530 to 1782)	1439 (683 to 2514)	1969 (957 to 3405)	62.2 (60.0 to 64.6)	63.3 (58.0 to 67.9)	65.9 (60.0 to 71.0)	68.8 (62.8 to 74.0)	6 (5 to 6)	6 (6 to 7)	7 (6 to 7)	7 (6 to 7)
**Argentina**	1193 (1114 to 1274)	1296 (832 to 1892)	1522 (970 to 2236)	2408 (1523 to 3569)	61.0 (59.2 to 62.8)	61.1 (57.7 to 64.1)	62.6 (59.0 to 65.6)	66.2 (62.5 to 69.5)	2641 (2564 to 2717)	2972 (2806 to 3117)	3041 (2869 to 3191)	3219 (3037 to 3381)
**Armenia**	171 (161 to 182)	230 (139 to 347)	294 (178 to 444)	395 (234 to 606)	64.0 (62.1 to 66.0)	67.7 (63.2 to 72.1)	69.9 (65.3 to 74.4)	72.7 (67.8 to 77.6)	194 (188 to 200)	209 (196 to 223)	216 (202 to 230)	225 (210 to 240)
**Australia**	3545 (3455 to 3638)	3614 (3127 to 4180)	4375 (3672 to 5210)	6705 (5711 to 7855)	81.5 (79.9 to 83.1)	83.2 (81.1 to 85.4)	85.2 (82.8 to 87.5)	90.4 (87.9 to 92.8)	1937 (1899 to 1974)	2288 (2228 to 2346)	2341 (2275 to 2404)	2484 (2416 to 2550)
**Austria**	4255 (4184 to 4341)	4332 (3817 to 4987)	5050 (4411 to 5867)	8024 (7036 to 9300)	81.4 (79.9 to 83.0)	84.9 (82.7 to 86.9)	86.6 (84.3 to 88.8)	92.1 (89.7 to 94.4)	701 (688 to 715)	753 (734 to 771)	769 (748 to 788)	818 (796 to 838)
**Azerbaijan**	258 (243 to 274)	342 (185 to 572)	448 (239 to 754)	586 (313 to 986)	56.8 (53.6 to 59.8)	62.3 (56.8 to 67.8)	64.7 (58.9 to 70.5)	66.9 (60.9 to 73.0)	556 (525 to 586)	686 (626 to 748)	713 (649 to 778)	737 (672 to 804)
**Bahrain**	1864 (1742 to 1986)	1970 (1120 to 3118)	2117 (1137 to 3537)	3704 (2028 to 6054)	67.4 (64.4 to 70.4)	73.1 (67.5 to 78.5)	73.8 (67.7 to 79.4)	79.6 (73.2 to 85.5)	92 (88 to 96)	143 (132 to 153)	144 (132 to 155)	156 (143 to 167)
**Bangladesh**	23 (21 to 26)	30 (21 to 42)	46 (31 to 64)	54 (37 to 76)	53.9 (51.4 to 56.3)	63.8 (59.9 to 67.5)	67.3 (63.1 to 71.0)	68.8 (64.5 to 72.7)	8634 (8233 to 9023)	11380 (10679 to 12027)	11997 (11258 to 12662)	12265 (11494 to 12954)
**Barbados**	676 (621 to 720)	781 (517 to 1074)	828 (544 to 1150)	1417 (929 to 1968)	62.6 (60.0 to 64.9)	65.7 (62.1 to 68.8)	66.2 (62.5 to 69.4)	71.1 (67.1 to 74.5)	18 (17 to 18)	19 (18 to 19)	19 (18 to 20)	20 (19 to 21)
**Belarus**	804 (769 to 838)	909 (678 to 1183)	937 (677 to 1242)	1646 (1216 to 2138)	69.3 (66.6 to 72.0)	74.5 (70.5 to 78.1)	74.7 (70.5 to 78.4)	80.7 (76.3 to 84.5)	658 (633 to 685)	694 (657 to 727)	696 (657 to 730)	751 (711 to 787)
**Belgium**	4049 (3967 to 4134)	4162 (3514 to 4859)	4682 (3912 to 5497)	7682 (6439 to 9001)	79.5 (77.7 to 81.3)	81.3 (78.9 to 83.6)	82.5 (80.0 to 84.8)	88.3 (85.7 to 90.8)	897 (876 to 918)	963 (935 to 990)	977 (947 to 1004)	1045 (1015 to 1075)
**Belize**	419 (395 to 445)	475 (317 to 672)	517 (329 to 772)	888 (580 to 1286)	55.2 (52.3 to 58.0)	57.6 (53.9 to 60.9)	58.2 (54.3 to 61.7)	62.4 (58.3 to 66.1)	20 (19 to 21)	29 (27 to 31)	29 (27 to 31)	32 (29 to 33)
**Benin**	47 (45 to 49)	53 (31 to 85)	54 (31 to 87)	98 (57 to 159)	45.6 (43.6 to 47.5)	49.2 (45.9 to 52.6)	49.2 (45.9 to 52.6)	53.5 (49.9 to 57.0)	503 (481 to 524)	874 (814 to 934)	874 (814 to 934)	949 (885 to 1012)
**Bhutan**	228 (215 to 243)	274 (140 to 463)	328 (162 to 571)	504 (252 to 863)	55.2 (51.9 to 58.4)	62.8 (56.7 to 67.7)	64.3 (57.9 to 69.5)	68.2 (61.4 to 73.5)	44 (41 to 46)	57 (52 to 62)	58 (53 to 63)	62 (56 to 67)
**Bolivia**	331 (316 to 346)	389 (255 to 544)	509 (333 to 715)	713 (458 to 1012)	51.6 (48.0 to 55.2)	57.0 (52.4 to 61.4)	59.1 (54.2 to 63.6)	61.8 (56.6 to 66.5)	562 (523 to 601)	812 (746 to 874)	842 (772 to 906)	881 (806 to 948)
**Bosnia and Herzegovina**	761 (723 to 815)	876 (494 to 1386)	1189 (667 to 1891)	1595 (875 to 2571)	64.7 (62.0 to 67.3)	65.0 (60.2 to 68.8)	67.4 (62.4 to 71.4)	70.2 (65.0 to 74.5)	247 (236 to 257)	228 (212 to 242)	237 (219 to 251)	247 (228 to 262)
**Botswana**	965 (879 to 1091)	1043 (674 to 1586)	1490 (896 to 2406)	1979 (1228 to 3106)	56.9 (50.1 to 67.3)	61.8 (54.7 to 73.6)	64.7 (56.9 to 77.1)	67.2 (59.3 to 80.1)	129 (113 to 152)	180 (159 to 214)	188 (166 to 225)	196 (173 to 233)
**Brazil**	1024 (994 to 1059)	944 (585 to 1268)	1213 (738 to 1639)	2027 (1234 to 2736)	61.7 (60.4 to 62.7)	62.4 (58.8 to 64.5)	64.5 (60.7 to 66.8)	68.7 (64.7 to 71.2)	12869 (12600 to 13081)	14172 (13362 to 14668)	14668 (13805 to 15189)	15625 (14698 to 16192)
**Brunei**	1963 (1786 to 2154)	1975 (1057 to 3268)	2009 (1058 to 3358)	3971 (2105 to 6606)	64.5 (61.7 to 67.6)	66.6 (60.9 to 72.0)	66.8 (60.8 to 72.3)	73.1 (66.6 to 79.1)	27 (26 to 29)	33 (30 to 35)	33 (30 to 35)	36 (33 to 39)
**Bulgaria**	856 (818 to 900)	1005 (630 to 1425)	1469 (914 to 2099)	1792 (1106 to 2581)	62.7 (60.2 to 65.2)	63.7 (59.6 to 67.4)	67.0 (62.5 to 70.8)	68.8 (64.2 to 72.9)	455 (437 to 473)	409 (383 to 432)	430 (401 to 454)	442 (412 to 468)
**Burkina Faso**	60 (58 to 63)	74 (46 to 116)	89 (55 to 141)	132 (79 to 216)	46.0 (44.0 to 48.3)	51.2 (47.8 to 54.2)	52.5 (49.0 to 55.6)	55.3 (51.5 to 58.8)	833 (796 to 874)	1462 (1366 to 1549)	1502 (1401 to 1590)	1580 (1471 to 1680)

**Burundi**	53 (50 to 57)	59 (38 to 93)	62 (40 to 97)	121 (74 to 194)	42.9 (40.4 to 45.9)	48.4 (44.3 to 53.2)	48.4 (44.4 to 53.1)	53.2 (48.5 to 58.5)	481 (452 to 514)	880 (805 to 967)	880 (806 to 965)	966 (880 to 1062)
**Cambodia**	82 (74 to 92)	98 (59 to 153)	106 (61 to 168)	164 (97 to 261)	49.2 (47.5 to 51.1)	61.9 (58.4 to 65.1)	62.6 (58.8 to 65.9)	66.4 (62.5 to 69.8)	772 (744 to 801)	1195 (1126 to 1256)	1208 (1135 to 1272)	1281 (1206 to 1348)
**Cameroon**	48 (42 to 56)	61 (37 to 96)	66 (39 to 103)	106 (62 to 169)	44.6 (41.4 to 48.0)	49.2 (45.4 to 53.1)	49.6 (45.8 to 53.5)	52.8 (48.7 to 57.0)	1043 (970 to 1122)	1726 (1595 to 1864)	1741 (1608 to 1880)	1853 (1711 to 2002)
**Canada**	4211 (4117 to 4333)	4259 (3415 to 5146)	5304 (4154 to 6522)	7968 (6310 to 9715)	79.2 (77.9 to 80.5)	79.8 (77.3 to 82.3)	82.1 (79.3 to 84.8)	86.8 (83.9 to 89.5)	2843 (2798 to 2891)	3232 (3129 to 3330)	3324 (3209 to 3431)	3513 (3397 to 3624)
**Cape Verde**	278 (264 to 295)	280 (156 to 445)	285 (148 to 475)	591 (324 to 947)	61.3 (58.6 to 64.2)	66.9 (62.1 to 70.7)	67.0 (61.7 to 71.2)	73.1 (67.8 to 77.5)	33 (32 to 35)	45 (42 to 48)	45 (42 to 48)	49 (46 to 52)
**Central African Republic**	16 (15 to 16)	23 (12 to 42)	25 (14 to 45)	41 (19 to 78)	29.9 (25.9 to 34.6)	31.7 (27.3 to 36.4)	31.7 (27.6 to 36.2)	33.9 (29.3 to 38.8)	147 (127 to 170)	208 (179 to 238)	208 (180 to 237)	221 (191 to 254)
**Chad**	43 (36 to 49)	44 (21 to 83)	45 (21 to 88)	106 (49 to 205)	36.3 (34.0 to 38.4)	40.1 (36.6 to 43.9)	40.1 (36.6 to 44.0)	45.0 (40.8 to 49.3)	506 (474 to 535)	981 (896 to 1075)	981 (895 to 1078)	1100 (999 to 1208)
**Chile**	1315 (1285 to 1351)	1398 (1076 to 1817)	1503 (1093 to 2046)	2645 (1986 to 3506)	70.4 (66.7 to 73.8)	71.8 (67.4 to 76.2)	72.2 (67.5 to 76.9)	78.2 (73.2 to 83.1)	1266 (1199 to 1328)	1416 (1328 to 1503)	1424 (1331 to 1515)	1542 (1443 to 1639)
**China**	522 (505 to 542)	602 (424 to 863)	1460 (1027 to 2091)	1489 (1043 to 2141)	68.5 (67.2 to 69.6)	69.6 (66.7 to 72.4)	78.3 (75.0 to 81.4)	78.5 (75.1 to 81.7)	93359 (91564 to 94911)	92326 (88371 to 95951)	103828 (99419 to 107915)	104112 (99609 to 108271)
**Colombia**	701 (643 to 754)	774 (537 to 1063)	988 (682 to 1363)	1438 (988 to 2002)	64.9 (63.0 to 66.6)	70.1 (66.9 to 73.0)	72.5 (69.1 to 75.6)	76.0 (72.3 to 79.3)	3124 (3032 to 3207)	3686 (3514 to 3837)	3812 (3631 to 3972)	3996 (3801 to 4166)
**Comoros**	35 (31 to 38)	34 (16 to 57)	42 (20 to 69)	72 (35 to 121)	44.7 (42.0 to 47.5)	47.6 (42.5 to 52.2)	48.4 (43.4 to 52.9)	52.3 (47.0 to 57.3)	34 (32 to 36)	45 (40 to 49)	46 (41 to 50)	50 (45 to 54)
**Congo**	100 (91 to 110)	129 (58 to 242)	134 (60 to 249)	233 (102 to 440)	46.1 (42.5 to 49.9)	51.0 (45.3 to 56.8)	51.3 (45.6 to 57.1)	55.0 (48.8 to 61.4)	212 (195 to 229)	350 (311 to 390)	352 (313 to 392)	377 (335 to 422)
**Costa Rica**	1044 (1004 to 1083)	1157 (848 to 1555)	1313 (935 to 1815)	2129 (1548 to 2886)	68.7 (66.7 to 70.5)	69.4 (66.6 to 72.3)	70.2 (67.2 to 73.4)	75.2 (72.1 to 78.4)	327 (318 to 336)	357 (343 to 372)	361 (346 to 378)	387 (371 to 404)
**Cote d'Ivoire**	69 (48 to 102)	94 (55 to 146)	107 (62 to 168)	165 (95 to 263)	42.6 (40.2 to 44.8)	46.6 (43.6 to 49.3)	46.9 (43.8 to 49.6)	49.7 (46.5 to 52.7)	959 (904 to 1009)	1590 (1488 to 1681)	1598 (1495 to 1692)	1697 (1587 to 1798)
**Croatia**	1477 (1359 to 1600)	1578 (1263 to 2003)	2123 (1626 to 2872)	2957 (2313 to 3890)	72.0 (70.2 to 74.2)	75.3 (72.7 to 78.0)	78.0 (75.0 to 81.1)	81.8 (78.7 to 84.9)	305 (297 to 314)	286 (276 to 297)	297 (285 to 309)	311 (300 to 323)
**Cuba**	932 (814 to 1056)	1013 (753 to 1283)	1226 (903 to 1562)	1903 (1405 to 2434)	67.1 (65.5 to 68.7)	64.6 (62.6 to 66.8)	66.2 (64.1 to 68.5)	70.3 (68.0 to 72.6)	766 (748 to 784)	710 (687 to 734)	728 (704 to 753)	772 (747 to 798)
**Cyprus**	2205 (1979 to 2434)	2351 (1734 to 3108)	2959 (2164 to 3935)	4335 (3157 to 5785)	77.2 (75.6 to 78.8)	81.7 (78.8 to 84.7)	84.1 (81.2 to 87.3)	88.6 (85.5 to 92.0)	70 (68 to 71)	82 (79 to 85)	84 (81 to 88)	89 (86 to 92)
**Czech Republic**	1911 (1606 to 2318)	2058 (1606 to 2581)	2598 (1985 to 3330)	3776 (2901 to 4800)	75.7 (74.1 to 77.2)	78.5 (76.9 to 80.3)	80.9 (79.1 to 82.8)	85.2 (83.3 to 87.1)	800 (783 to 816)	822 (804 to 840)	847 (828 to 866)	891 (872 to 912)
**Democratic Republic of the**	28 (26 to 30)	29 (15 to 53)	34 (17 to 60)	61 (30 to 114)	42.7 (40.4 to 45.4)	45.2 (41.6 to 49.4)	46.0 (42.3 to 50.2)	49.3 (45.2 to 54.1)	3285 (3110 to 3492)	6002 (5521 to 6563)	6109 (5621 to 6665)	6550 (6009 to 7187)
**Denmark**	4436 (4345 to 4559)	4502 (3869 to 5145)	5314 (4535 to 6114)	8367 (7145 to 9604)	79.0 (76.9 to 81.1)	81.4 (78.8 to 84.1)	83.1 (80.4 to 85.8)	88.5 (85.5 to 91.3)	448 (437 to 461)	490 (474 to 506)	500 (484 to 517)	532 (515 to 549)
**Djibouti**	115 (107 to 124)	138 (67 to 224)	147 (71 to 238)	253 (121 to 414)	45.6 (41.9 to 50.0)	50.0 (43.9 to 55.9)	50.4 (44.3 to 56.4)	54.2 (47.5 to 60.6)	44 (40 to 48)	71 (62 to 79)	71 (63 to 80)	77 (67 to 86)
**Dominica**	428 (412 to 446)	504 (303 to 766)	536 (315 to 829)	918 (540 to 1423)	56.4 (54.0 to 58.7)	56.1 (52.1 to 59.8)	56.5 (52.3 to 60.4)	60.8 (56.4 to 64.9)	4 (4 to 4)	5 (4 to 5)	5 (4 to 5)	5 (5 to 5)

**Dominican Republic**	525 (498 to 564)	622 (377 to 1020)	952 (572 to 1559)	1121 (668 to 1844)	61.5 (58.9 to 64.3)	61.0 (56.9 to 65.1)	64.4 (59.9 to 68.8)	65.9 (61.3 to 70.5)	641 (613 to 671)	716 (667 to 763)	755 (703 to 806)	773 (718 to 826)
**Ecuador**	581 (549 to 618)	678 (424 to 998)	751 (466 to 1112)	1229 (748 to 1846)	60.5 (58.8 to 62.3)	59.2 (55.8 to 62.0)	60.1 (56.6 to 62.9)	64.1 (60.3 to 67.3)	987 (959 to 1017)	1219 (1149 to 1275)	1235 (1164 to 1293)	1319 (1241 to 1384)
**Egypt**	184 (167 to 202)	228 (151 to 328)	287 (182 to 432)	416 (266 to 615)	59.9 (57.6 to 62.4)	66.6 (63.0 to 70.2)	68.6 (64.6 to 72.7)	72.1 (68.1 to 76.3)	5415 (5208 to 5639)	7414 (7018 to 7816)	7636 (7196 to 8089)	8029 (7586 to 8490)
**El Salvador**	429 (413 to 446)	496 (384 to 640)	557 (422 to 734)	909 (693 to 1189)	62.4 (59.6 to 64.8)	65.3 (62.2 to 68.1)	66.2 (62.9 to 69.0)	70.8 (67.4 to 73.9)	384 (367 to 399)	411 (391 to 428)	416 (396 to 434)	445 (424 to 465)
**Equatorial Guinea**	351 (274 to 464)	433 (140 to 980)	776 (269 to 1663)	815 (271 to 1854)	51.1 (44.3 to 58.9)	57.4 (48.1 to 66.6)	62.5 (52.8 to 72.3)	62.5 (52.5 to 72.5)	42 (36 to 48)	81 (68 to 94)	88 (74 to 102)	88 (74 to 102)
**Eritrea**	18 (16 to 22)	22 (10 to 39)	35 (19 to 57)	43 (21 to 76)	38.9 (36.3 to 41.9)	47.1 (42.3 to 51.7)	50.0 (45.5 to 54.5)	51.3 (46.2 to 56.4)	203 (189 to 219)	328 (295 to 361)	348 (317 to 380)	358 (322 to 393)
**Estonia**	1495 (1480 to 1512)	1631 (1190 to 2189)	1972 (1398 to 2713)	2995 (2159 to 4053)	73.9 (71.8 to 76.0)	78.9 (75.0 to 82.7)	80.8 (76.5 to 85.0)	85.6 (81.3 to 89.8)	97 (94 to 100)	97 (93 to 102)	100 (94 to 105)	106 (100 to 111)
**Ethiopia**	54 (50 to 59)	67 (35 to 122)	104 (49 to 196)	124 (61 to 231)	39.3 (36.4 to 42.1)	48.0 (43.3 to 53.4)	51.0 (45.6 to 57.0)	52.0 (46.7 to 58.0)	3912 (3630 to 4199)	7185 (6481 to 7980)	7623 (6824 to 8524)	7783 (6988 to 8682)
**Federated States of Micronesia**	229 (220 to 237)	145 (86 to 238)	147 (85 to 245)	488 (291 to 785)	44.6 (40.2 to 49.4)	45.9 (41.1 to 51.0)	45.9 (41.1 to 51.1)	49.9 (44.8 to 55.3)	5 (4 to 5)	5 (4 to 5)	5 (4 to 5)	5 (5 to 6)
**Fiji**	272 (255 to 296)	313 (213 to 459)	377 (236 to 585)	584 (379 to 886)	46.5 (43.1 to 50.4)	48.4 (44.3 to 53.4)	49.7 (45.1 to 55.2)	52.5 (47.9 to 58.2)	40 (37 to 43)	39 (36 to 43)	40 (37 to 45)	43 (39 to 47)
**Finland**	3292 (3221 to 3368)	3424 (2800 to 4155)	4300 (3473 to 5265)	6318 (5122 to 7727)	84.5 (82.8 to 86.1)	88.0 (85.2 to 90.5)	90.7 (87.7 to 93.3)	95.5 (92.5 to 98.3)	462 (453 to 471)	501 (485 to 515)	516 (500 to 531)	544 (527 to 560)
**France**	4419 (4342 to 4485)	4412 (3941 to 4950)	5005 (4414 to 5691)	8287 (7358 to 9368)	80.1 (78.5 to 81.6)	82.8 (81.0 to 84.5)	84.1 (82.1 to 85.9)	90.1 (88.0 to 92.0)	5171 (5066 to 5263)	5585 (5463 to 5701)	5674 (5540 to 5796)	6075 (5938 to 6204)
**Gabon**	359 (330 to 387)	405 (230 to 683)	433 (231 to 757)	778 (422 to 1346)	48.9 (45.5 to 52.5)	56.6 (51.7 to 62.0)	57.0 (51.9 to 62.7)	61.7 (56.3 to 67.9)	84 (79 to 91)	141 (128 to 154)	142 (129 to 156)	153 (140 to 169)
**Georgia**	344 (302 to 395)	425 (193 to 773)	539 (237 to 997)	752 (328 to 1397)	58.7 (56.0 to 61.4)	56.1 (51.0 to 60.4)	57.7 (52.3 to 62.4)	60.4 (54.7 to 65.3)	239 (227 to 249)	229 (208 to 246)	235 (213 to 254)	246 (223 to 266)
**Germany**	4839 (4587 to 5196)	4879 (4152 to 5722)	5397 (4563 to 6388)	9085 (7687 to 10771)	78.9 (77.0 to 80.7)	80.9 (78.8 to 82.9)	81.7 (79.5 to 83.9)	87.9 (85.5 to 90.2)	6431 (6278 to 6577)	6616 (6441 to 6781)	6683 (6501 to 6856)	7188 (6994 to 7371)
**Ghana**	144 (135 to 153)	178 (90 to 308)	265 (133 to 455)	318 (154 to 561)	51.5 (49.2 to 54.0)	55.6 (50.7 to 59.6)	58.8 (53.6 to 63.1)	59.9 (54.4 to 64.4)	1425 (1362 to 1494)	2155 (1965 to 2313)	2282 (2079 to 2446)	2322 (2111 to 2497)
**Greece**	1558 (1425 to 1685)	1711 (1440 to 2028)	1907 (1597 to 2270)	3107 (2599 to 3711)	78.3 (76.5 to 79.9)	80.7 (78.7 to 82.7)	81.9 (79.9 to 84.0)	87.4 (85.3 to 89.7)	854 (835 to 872)	825 (805 to 846)	838 (817 to 860)	894 (872 to 917)
**Grenada**	322 (270 to 383)	394 (218 to 618)	460 (245 to 756)	707 (382 to 1144)	54.5 (51.9 to 57.0)	56.9 (53.0 to 60.3)	58.1 (53.9 to 61.8)	61.3 (57.0 to 65.2)	6 (5 to 6)	6 (6 to 6)	6 (6 to 7)	6 (6 to 7)
**Guatemala**	232 (223 to 242)	236 (174 to 322)	273 (191 to 382)	463 (334 to 641)	53.8 (50.1 to 57.8)	58.2 (53.8 to 62.7)	59.3 (54.7 to 64.0)	62.9 (58.2 to 67.8)	873 (813 to 937)	1227 (1135 to 1322)	1251 (1153 to 1349)	1327 (1227 to 1429)
**Guinea**	60 (58 to 62)	42 (24 to 72)	63 (35 to 106)	78 (42 to 135)	39.2 (36.7 to 41.4)	42.9 (39.0 to 46.8)	43.4 (39.5 to 47.4)	44.8 (40.6 to 49.0)	492 (461 to 520)	810 (737 to 884)	819 (744 to 895)	845 (766 to 924)
**Guinea-Bissau**	82 (76 to 92)	74 (39 to 143)	79 (42 to 154)	173 (84 to 352)	37.8 (35.3 to 40.5)	41.7 (38.2 to 45.4)	42.1 (38.5 to 45.8)	46.4 (42.2 to 50.8)	70 (66 to 75)	113 (104 to 124)	114 (105 to 125)	126 (115 to 138)
**Guyana**	192 (175 to 211)	229 (131 to 362)	243 (130 to 396)	409 (228 to 650)	49.7 (47.4 to 52.0)	54.7 (50.8 to 58.1)	55.1 (50.8 to 58.8)	59.1 (54.8 to 62.8)	38 (36 to 40)	44 (40 to 46)	44 (40 to 47)	47 (44 to 50)
**Haiti**	90 (85 to 95)	105 (66 to 166)	110 (70 to 173)	191 (112 to 312)	39.7 (36.2 to 43.3)	45.1 (40.8 to 49.3)	45.4 (41.1 to 49.6)	48.8 (44.0 to 53.5)	436 (398 to 475)	635 (575 to 694)	639 (579 to 698)	687 (620 to 754)

**Honduras**	182 (167 to 201)	223 (143 to 330)	257 (163 to 385)	400 (252 to 602)	54.3 (50.1 to 58.3)	58.7 (53.8 to 63.5)	59.8 (54.7 to 64.7)	63.5 (58.0 to 68.8)	445 (410 to 478)	607 (556 to 657)	618 (566 to 669)	657 (600 to 712)
**Hungary**	1443 (1388 to 1522)	1570 (1259 to 1944)	1923 (1491 to 2470)	2869 (2272 to 3621)	69.6 (67.4 to 71.8)	73.3 (70.1 to 76.7)	75.3 (71.9 to 79.1)	79.5 (75.9 to 83.3)	688 (667 to 709)	678 (648 to 709)	696 (665 to 731)	735 (702 to 770)
**Iceland**	3504 (3390 to 3615)	3611 (2916 to 4324)	4923 (3933 to 5953)	6690 (5350 to 8080)	85.1 (83.1 to 86.9)	88.6 (86.0 to 90.9)	91.9 (89.1 to 94.4)	96.0 (93.1 to 98.6)	28 (27 to 29)	32 (31 to 32)	33 (32 to 34)	34 (33 to 35)
**India**	84 (81 to 87)	106 (81 to 135)	197 (149 to 251)	200 (151 to 256)	49.0 (47.0 to 50.5)	54.2 (52.1 to 56.2)	58.6 (56.4 to 60.8)	58.7 (56.5 to 60.9)	63760 (61186 to 65787)	77157 (74178 to 79970)	83439 (80248 to 86509)	83627 (80394 to 86709)
**Indonesia**	198 (190 to 209)	234 (177 to 305)	368 (275 to 481)	432 (326 to 565)	49.7 (48.4 to 51.0)	52.6 (50.5 to 54.6)	55.8 (53.6 to 58.0)	57.0 (54.8 to 59.2)	12728 (12395 to 13052)	15039 (14438 to 15610)	15972 (15323 to 16606)	16322 (15667 to 16938)
**Iran**	693 (663 to 727)	800 (445 to 1316)	1032 (521 to 1813)	1462 (770 to 2480)	66.8 (63.4 to 70.0)	67.4 (61.8 to 73.4)	68.9 (62.5 to 75.5)	72.4 (66.2 to 79.2)	5375 (5099 to 5632)	6414 (5882 to 6985)	6552 (5946 to 7186)	6892 (6294 to 7536)
**Iraq**	230 (211 to 247)	301 (134 to 562)	435 (191 to 819)	527 (228 to 1004)	51.4 (48.0 to 55.1)	55.5 (49.7 to 61.3)	57.9 (51.7 to 64.0)	59.5 (52.9 to 65.9)	1964 (1832 to 2104)	3562 (3186 to 3933)	3715 (3315 to 4108)	3817 (3397 to 4229)
**Ireland**	4581 (4336 to 4802)	4628 (3261 to 6238)	6184 (4132 to 8625)	8661 (5969 to 11874)	79.9 (77.5 to 82.0)	84.3 (80.5 to 87.6)	87.7 (83.2 to 91.5)	91.6 (87.3 to 95.4)	369 (358 to 379)	425 (406 to 442)	442 (419 to 461)	462 (440 to 481)
**Israel**	1963 (1807 to 2105)	2074 (1712 to 2469)	2490 (1987 to 3075)	3887 (3162 to 4711)	76.1 (72.9 to 79.1)	79.3 (76.0 to 82.5)	81.1 (77.7 to 84.3)	86.2 (82.7 to 89.7)	614 (588 to 638)	808 (774 to 840)	825 (792 to 859)	878 (842 to 913)
**Italy**	2661 (2577 to 2742)	2805 (2427 to 3208)	3210 (2764 to 3682)	5155 (4428 to 5911)	80.5 (78.8 to 82.1)	83.8 (81.4 to 85.9)	85.2 (82.8 to 87.4)	90.9 (88.3 to 93.2)	4851 (4750 to 4947)	4829 (4695 to 4953)	4911 (4772 to 5039)	5238 (5091 to 5374)
**Jamaica**	382 (349 to 411)	334 (206 to 501)	374 (228 to 568)	814 (503 to 1229)	61.1 (57.9 to 64.3)	61.4 (57.3 to 65.5)	62.3 (58.0 to 66.5)	69.3 (64.6 to 73.9)	175 (166 to 184)	186 (174 to 199)	189 (176 to 202)	210 (196 to 224)
**Japan**	3719 (3599 to 3897)	3808 (3138 to 4570)	3861 (3146 to 4685)	7084 (5811 to 8524)	82.4 (81.0 to 83.5)	83.6 (81.7 to 85.2)	83.6 (81.7 to 85.3)	90.9 (88.8 to 92.7)	10350 (10169 to 10493)	9663 (9451 to 9854)	9663 (9442 to 9860)	10504 (10271 to 10714)
**Jordan**	555 (504 to 604)	624 (400 to 908)	722 (441 to 1095)	1156 (712 to 1746)	65.2 (61.5 to 68.9)	70.0 (64.9 to 74.7)	71.3 (65.9 to 76.5)	75.9 (70.1 to 81.4)	494 (465 to 521)	651 (604 to 696)	664 (614 to 712)	707 (653 to 758)
**Kazakhstan**	638 (621 to 654)	747 (472 to 1131)	955 (582 to 1468)	1345 (833 to 2051)	61.8 (59.1 to 64.6)	67.6 (62.8 to 72.0)	69.9 (64.7 to 74.5)	73.2 (67.9 to 77.9)	1090 (1041 to 1139)	1425 (1323 to 1516)	1472 (1364 to 1570)	1541 (1430 to 1642)
**Kenya**	131 (129 to 133)	155 (117 to 205)	175 (131 to 232)	286 (216 to 376)	54.4 (51.9 to 57.4)	58.3 (54.9 to 61.9)	59.3 (55.8 to 63.0)	63.2 (59.5 to 67.1)	2471 (2357 to 2607)	3723 (3509 to 3956)	3785 (3566 to 4021)	4033 (3803 to 4284)
**Kiribati**	180 (162 to 202)	213 (161 to 284)	285 (217 to 379)	400 (307 to 528)	40.5 (37.4 to 43.2)	44.4 (41.2 to 47.3)	45.8 (42.4 to 48.8)	48.0 (44.5 to 51.2)	5 (4 to 5)	6 (5 to 6)	6 (6 to 6)	6 (6 to 7)
**Kuwait**	2237 (2028 to 2461)	2177 (863 to 3732)	2193 (785 to 3870)	4556 (1768 to 7847)	71.6 (67.9 to 75.6)	74.9 (66.7 to 81.9)	74.9 (65.8 to 82.3)	82.8 (73.5 to 90.5)	274 (260 to 290)	345 (307 to 378)	345 (303 to 380)	382 (339 to 417)
**Kyrgyzstan**	164 (148 to 192)	190 (104 to 317)	193 (100 to 331)	357 (186 to 625)	58.7 (57.0 to 60.6)	62.3 (57.6 to 65.8)	62.3 (57.2 to 66.0)	67.7 (62.3 to 71.5)	346 (336 to 357)	456 (422 to 482)	456 (419 to 483)	496 (457 to 524)
**Laos**	99 (85 to 115)	121 (75 to 184)	191 (120 to 288)	213 (129 to 335)	42.5 (39.6 to 45.3)	55.7 (51.4 to 60.3)	59.5 (55.0 to 64.4)	60.0 (55.3 to 65.0)	300 (280 to 320)	558 (515 to 604)	596 (550 to 644)	601 (554 to 651)
**Latvia**	1051 (1004 to 1103)	1191 (905 to 1593)	1550 (1146 to 2129)	2146 (1605 to 2923)	68.6 (66.5 to 70.7)	72.7 (69.5 to 76.2)	75.3 (71.8 to 79.1)	78.6 (75.0 to 82.5)	137 (132 to 141)	132 (126 to 138)	137 (130 to 144)	143 (136 to 150)
**Lebanon**	820 (743 to 916)	823 (348 to 1508)	926 (395 to 1717)	1725 (728 to 3199)	73.6 (70.9 to 76.0)	78.4 (70.7 to 85.9)	79.6 (71.8 to 87.4)	86.6 (78.1 to 95.2)	419 (404 to 433)	387 (349 to 424)	393 (355 to 432)	427 (385 to 470)
**Lesotho**	217 (207 to 229)	266 (181 to 385)	407 (278 to 588)	488 (329 to 706)	41.8 (37.5 to 46.7)	40.2 (36.1 to 44.6)	41.8 (37.6 to 46.3)	42.8 (38.5 to 47.5)	88 (79 to 99)	104 (94 to 115)	108 (97 to 120)	111 (100 to 123)

**Liberia**	454 (450 to 459)	213 (138 to 338)	229 (150 to 362)	420 (264 to 685)	45.8 (43.8 to 47.9)	44.2 (41.4 to 47.6)	44.7 (41.8 to 48.1)	47.5 (44.3 to 51.3)	206 (197 to 215)	291 (272 to 313)	294 (275 to 316)	313 (292 to 337)
**Libya**	304 (265 to 362)	388 (225 to 626)	454 (259 to 738)	666 (356 to 1105)	64.4 (62.0 to 66.7)	73.4 (68.3 to 78.3)	75.0 (69.7 to 80.0)	78.5 (72.7 to 84.1)	395 (380 to 409)	480 (446 to 511)	490 (456 to 523)	513 (475 to 549)
**Lithuania**	1313 (1251 to 1379)	1466 (1069 to 1941)	1984 (1388 to 2699)	2660 (1910 to 3568)	67.4 (65.9 to 68.9)	68.1 (65.0 to 71.1)	70.8 (67.2 to 74.0)	73.8 (70.2 to 77.0)	197 (192 to 201)	183 (174 to 190)	190 (180 to 198)	198 (188 to 206)
**Luxembourg**	5836 (5549 to 6085)	5815 (4613 to 7231)	8404 (6475 to 10750)	10945 (8586 to 13816)	82.2 (80.1 to 84.2)	85.2 (82.3 to 87.8)	89.4 (86.2 to 92.4)	92.7 (89.5 to 95.7)	47 (45 to 48)	57 (55 to 59)	60 (58 to 62)	62 (60 to 64)
**Macedonia**	600 (421 to 895)	651 (462 to 900)	676 (459 to 972)	1251 (870 to 1759)	63.2 (61.3 to 65.0)	66.1 (63.8 to 68.1)	66.4 (64.3 to 68.4)	71.9 (69.5 to 74.1)	131 (127 to 135)	134 (130 to 138)	135 (130 to 139)	146 (141 to 150)
**Madagascar**	60 (56 to 65)	73 (40 to 116)	79 (44 to 125)	136 (73 to 220)	38.4 (35.3 to 41.7)	41.6 (37.4 to 46.1)	41.9 (37.7 to 46.4)	45.2 (40.6 to 50.1)	932 (857 to 1012)	1467 (1319 to 1625)	1478 (1329 to 1636)	1592 (1431 to 1766)
**Malawi**	124 (121 to 127)	137 (84 to 221)	145 (89 to 231)	274 (155 to 458)	48.3 (45.0 to 52.2)	50.7 (46.0 to 55.9)	51.0 (46.3 to 56.2)	55.6 (50.3 to 61.5)	840 (781 to 907)	1397 (1267 to 1542)	1405 (1276 to 1551)	1533 (1386 to 1697)
**Malaysia**	680 (654 to 709)	765 (579 to 965)	1065 (788 to 1370)	1403 (1049 to 1786)	63.7 (62.2 to 65.2)	65.3 (62.9 to 67.4)	68.1 (65.3 to 70.4)	70.8 (68.0 to 73.1)	1940 (1894 to 1986)	2316 (2230 to 2391)	2416 (2317 to 2498)	2511 (2413 to 2594)
**Maldives**	1517 (1362 to 1693)	1638 (1056 to 2299)	1831 (1159 to 2588)	3026 (1906 to 4303)	72.0 (69.0 to 75.0)	72.6 (68.4 to 76.4)	73.2 (69.1 to 77.2)	78.7 (74.2 to 83.0)	26 (25 to 27)	31 (29 to 32)	31 (29 to 33)	33 (31 to 35)
**Mali**	58 (54 to 62)	74 (43 to 120)	92 (52 to 151)	129 (71 to 212)	43.6 (40.6 to 46.8)	49.1 (44.4 to 53.3)	50.3 (45.4 to 54.7)	52.7 (47.5 to 57.4)	759 (707 to 814)	1388 (1255 to 1509)	1424 (1286 to 1549)	1492 (1345 to 1624)
**Malta**	2295 (2238 to 2347)	2494 (2090 to 2919)	3867 (3221 to 4579)	4503 (3753 to 5327)	76.6 (73.9 to 79.4)	79.8 (76.6 to 83.1)	84.9 (81.4 to 88.4)	86.4 (82.8 to 90.0)	32 (31 to 33)	34 (33 to 35)	36 (35 to 38)	37 (35 to 38)
**Marshall Islands**	525 (486 to 574)	455 (210 to 778)	460 (197 to 817)	1107 (503 to 1901)	43.4 (39.9 to 47.1)	47.6 (42.8 to 51.6)	47.6 (42.4 to 51.8)	52.6 (47.1 to 57.0)	3 (3 to 3)	5 (4 to 5)	5 (4 to 5)	5 (4 to 5)
**Mauritania**	95 (85 to 106)	118 (60 to 204)	122 (61 to 213)	213 (105 to 376)	49.9 (46.3 to 54.1)	56.6 (50.9 to 63.0)	56.8 (51.1 to 63.2)	61.1 (54.8 to 67.9)	198 (184 to 215)	318 (287 to 355)	320 (288 to 356)	344 (309 to 382)
**Mauritius**	517 (493 to 543)	620 (465 to 820)	809 (593 to 1099)	1066 (795 to 1423)	64.6 (62.2 to 66.9)	66.8 (63.6 to 69.7)	69.0 (65.4 to 72.0)	71.8 (68.2 to 74.8)	82 (79 to 85)	86 (82 to 90)	89 (84 to 93)	93 (88 to 97)
**Mexico**	634 (608 to 656)	726 (599 to 861)	914 (731 to 1120)	1320 (1077 to 1594)	59.8 (58.3 to 61.1)	61.8 (60.1 to 63.4)	63.7 (61.8 to 65.4)	67.0 (65.0 to 68.7)	7602 (7420 to 7769)	9114 (8860 to 9339)	9394 (9105 to 9647)	9871 (9578 to 10124)
**Moldova**	297 (271 to 320)	356 (226 to 516)	395 (250 to 574)	630 (399 to 918)	62.9 (60.9 to 65.0)	66.7 (62.7 to 70.4)	67.7 (63.6 to 71.5)	71.7 (67.4 to 75.7)	256 (248 to 265)	263 (247 to 277)	266 (250 to 281)	282 (265 to 298)
**Mongolia**	303 (281 to 327)	363 (212 to 550)	477 (270 to 751)	632 (365 to 968)	58.4 (55.5 to 61.2)	64.1 (59.6 to 67.7)	66.3 (61.3 to 70.2)	68.9 (63.9 to 72.9)	174 (166 to 183)	242 (225 to 256)	251 (232 to 266)	261 (242 to 276)
**Montenegro**	666 (640 to 698)	763 (649 to 883)	830 (700 to 964)	1382 (1172 to 1602)	69.1 (67.2 to 70.8)	74.2 (71.9 to 76.5)	74.8 (72.5 to 77.2)	80.3 (77.9 to 82.8)	43 (42 to 44)	46 (45 to 48)	47 (45 to 48)	50 (48 to 52)
**Morocco**	213 (198 to 233)	275 (193 to 383)	382 (266 to 533)	488 (338 to 685)	57.8 (55.0 to 60.2)	61.8 (57.9 to 65.9)	64.2 (60.2 to 68.6)	66.4 (62.2 to 71.0)	1924 (1831 to 2005)	2178 (2044 to 2325)	2264 (2122 to 2421)	2342 (2194 to 2505)
**Mozambique**	67 (66 to 69)	84 (56 to 127)	116 (73 to 179)	156 (101 to 241)	45.2 (42.0 to 48.2)	49.5 (45.2 to 54.3)	50.0 (45.5 to 55.0)	52.2 (47.6 to 57.4)	1267 (1179 to 1351)	2194 (2005 to 2407)	2217 (2018 to 2440)	2314 (2111 to 2544)
**Myanmar**	86 (76 to 96)	124 (86 to 174)	226 (157 to 315)	244 (170 to 340)	48.9 (46.4 to 51.1)	51.7 (48.8 to 54.2)	55.8 (52.7 to 58.5)	56.4 (53.2 to 59.2)	2645 (2509 to 2762)	3153 (2976 to 3307)	3409 (3216 to 3574)	3446 (3250 to 3613)
**Namibia**	945 (875 to 1019)	955 (685 to 1314)	1043 (714 to 1475)	1910 (1336 to 2658)	54.8 (50.3 to 61.0)	61.7 (56.4 to 69.0)	62.4 (57.0 to 69.9)	67.5 (61.7 to 75.6)	134 (123 to 149)	213 (195 to 238)	216 (197 to 241)	233 (213 to 261)
**Nepal**	68 (64 to 72)	77 (50 to 118)	93 (59 to 145)	144 (91 to 224)	51.2 (48.4 to 53.9)	58.7 (54.9 to 62.2)	60.3 (56.3 to 63.9)	63.5 (59.2 to 67.3)	1518 (1435 to 1599)	2215 (2070 to 2347)	2273 (2122 to 2411)	2394 (2235 to 2539)

**Netherlands**	4902 (4682 to 5177)	4899 (4110 to 5841)	5728 (4794 to 6829)	9132 (7587 to 10979)	81.9 (80.1 to 83.8)	83.9 (81.6 to 86.0)	85.4 (83.1 to 87.7)	91.1 (88.5 to 93.5)	1396 (1364 to 1427)	1477 (1436 to 1515)	1504 (1463 to 1543)	1604 (1558 to 1647)
**New Zealand**	3189 (2988 to 3424)	3271 (2805 to 3827)	3821 (3224 to 4512)	6098 (5193 to 7157)	77.6 (75.6 to 79.5)	79.1 (76.8 to 81.4)	80.6 (78.1 to 82.9)	85.9 (83.4 to 88.4)	350 (341 to 359)	396 (385 to 408)	403 (391 to 415)	430 (418 to 443)
**Nicaragua**	283 (263 to 304)	335 (225 to 470)	343 (225 to 491)	608 (404 to 862)	64.4 (61.7 to 67.1)	68.2 (64.2 to 71.9)	68.2 (64.1 to 72.0)	74.0 (69.6 to 78.1)	393 (376 to 409)	478 (450 to 504)	478 (449 to 504)	519 (488 to 547)
**Niger**	30 (29 to 32)	39 (24 to 59)	41 (25 to 62)	68 (41 to 103)	42.3 (39.6 to 45.1)	49.8 (45.6 to 53.9)	50.0 (45.8 to 54.2)	53.5 (49.0 to 58.0)	816 (764 to 870)	1694 (1551 to 1835)	1703 (1560 to 1845)	1822 (1667 to 1975)
**Nigeria**	57 (51 to 62)	64 (25 to 133)	66 (25 to 145)	135 (50 to 292)	47.8 (44.7 to 51.2)	51.2 (45.3 to 57.3)	51.4 (45.4 to 57.8)	56.5 (49.6 to 63.5)	8607 (8057 to 9237)	14056 (12432 to 15728)	14092 (12445 to 15856)	15489 (13621 to 17411)
**North Korea**	60 (54 to 67)	55 (49 to 61)	55 (49 to 62)	142 (124 to 163)	56.2 (53.5 to 58.6)	58.0 (55.2 to 60.5)	58.0 (55.2 to 60.5)	65.9 (62.8 to 68.7)	1482 (1411 to 1545)	1771 (1685 to 1848)	1773 (1687 to 1849)	2012 (1918 to 2098)
**Norway**	6019 (5804 to 6268)	6088 (4181 to 7940)	6764 (4592 to 8889)	11302 (7698 to 14815)	83.1 (81.1 to 85.0)	85.5 (81.4 to 88.5)	86.2 (82.0 to 89.3)	92.8 (88.3 to 96.1)	431 (421 to 441)	504 (480 to 522)	508 (483 to 526)	547 (520 to 566)
**Oman**	1576 (1449 to 1707)	1701 (1017 to 2642)	1887 (1080 to 3039)	3160 (1851 to 4972)	74.7 (72.8 to 76.5)	78.0 (73.6 to 82.6)	79.1 (74.2 to 84.2)	84.6 (79.5 to 89.7)	339 (331 to 348)	486 (458 to 514)	493 (462 to 524)	527 (495 to 559)
**Pakistan**	51 (47 to 55)	70 (40 to 113)	89 (51 to 145)	121 (70 to 197)	42.7 (40.0 to 45.4)	49.1 (44.6 to 53.2)	50.6 (46.0 to 54.9)	52.8 (48.0 to 57.3)	8010 (7505 to 8506)	11268 (10256 to 12229)	11618 (10559 to 12610)	12128 (11028 to 13175)
**Palestine**	233 (203 to 266)	268 (197 to 366)	325 (234 to 450)	503 (360 to 699)	58.7 (56.7 to 60.5)	60.4 (58.5 to 62.2)	61.9 (59.9 to 63.9)	65.6 (63.5 to 67.6)	295 (285 to 304)	527 (510 to 543)	540 (523 to 557)	573 (554 to 590)
**Panama**	1102 (1041 to 1169)	1220 (875 to 1654)	1887 (1302 to 2629)	2262 (1592 to 3124)	62.2 (59.6 to 64.8)	62.5 (59.3 to 65.7)	66.2 (62.6 to 69.6)	67.9 (64.3 to 71.3)	243 (233 to 253)	287 (272 to 301)	304 (287 to 319)	311 (295 to 327)
**Papua New Guinea**	114 (107 to 124)	116 (78 to 161)	118 (78 to 164)	257 (173 to 355)	38.3 (34.7 to 42.1)	41.6 (37.4 to 45.5)	41.6 (37.4 to 45.6)	45.8 (41.2 to 50.1)	294 (266 to 323)	439 (395 to 480)	439 (395 to 481)	483 (434 to 528)
**Paraguay**	470 (439 to 506)	545 (351 to 798)	865 (545 to 1303)	1000 (627 to 1507)	55.5 (53.2 to 57.8)	54.2 (50.9 to 57.2)	57.7 (54.1 to 61.1)	58.7 (54.9 to 62.2)	367 (351 to 382)	410 (385 to 433)	437 (410 to 463)	444 (416 to 471)
**Peru**	471 (458 to 485)	539 (386 to 753)	676 (473 to 956)	988 (693 to 1397)	65.3 (62.3 to 68.2)	70.3 (66.1 to 74.6)	72.6 (68.2 to 77.1)	76.2 (71.6 to 80.9)	2076 (1980 to 2169)	2879 (2706 to 3056)	2971 (2791 to 3155)	3121 (2932 to 3313)
**Philippines**	154 (150 to 159)	181 (129 to 243)	283 (197 to 390)	340 (239 to 462)	49.0 (46.6 to 51.1)	51.0 (47.9 to 53.6)	54.1 (50.8 to 56.9)	55.4 (52.0 to 58.3)	4940 (4702 to 5161)	6471 (6081 to 6800)	6863 (6443 to 7219)	7035 (6605 to 7395)
**Poland**	1339 (1243 to 1464)	1458 (1218 to 1746)	2041 (1654 to 2501)	2707 (2222 to 3289)	71.4 (69.2 to 73.3)	75.9 (73.5 to 78.0)	79.4 (76.9 to 81.8)	82.4 (79.8 to 84.9)	2751 (2667 to 2825)	2802 (2715 to 2879)	2932 (2839 to 3019)	3043 (2948 to 3132)
**Portugal**	1962 (1859 to 2078)	2102 (1723 to 2507)	2780 (2225 to 3387)	3904 (3147 to 4718)	76.5 (74.9 to 77.9)	81.6 (79.4 to 83.7)	84.8 (82.2 to 87.2)	88.6 (86.0 to 91.1)	801 (785 to 816)	794 (773 to 814)	825 (800 to 849)	862 (836 to 886)
**Qatar**	3018 (2798 to 3248)	3222 (1593 to 6015)	3441 (1479 to 6766)	5889 (2781 to 11269)	76.6 (72.1 to 80.9)	83.2 (75.3 to 91.2)	83.7 (74.8 to 92.5)	90.1 (81.1 to 99.1)	170 (160 to 180)	248 (225 to 272)	250 (223 to 276)	269 (242 to 296)
**Romania**	890 (820 to 959)	998 (677 to 1409)	1667 (1117 to 2361)	1837 (1235 to 2600)	65.6 (63.6 to 67.5)	67.8 (63.9 to 71.5)	72.5 (68.2 to 76.4)	73.5 (69.2 to 77.5)	1277 (1238 to 1314)	1219 (1150 to 1286)	1303 (1227 to 1374)	1321 (1244 to 1393)
**Russian Federation**	993 (984 to 1003)	1125 (753 to 1610)	1132 (716 to 1666)	2045 (1347 to 2977)	62.3 (57.8 to 66.5)	66.6 (60.6 to 72.4)	66.6 (60.3 to 72.5)	72.2 (65.6 to 78.6)	9024 (8383 to 9637)	9553 (8699 to 10388)	9553 (8651 to 10411)	10358 (9416 to 11277)
**Rwanda**	110 (103 to 119)	130 (83 to 198)	152 (94 to 238)	236 (145 to 370)	51.0 (48.4 to 53.7)	60.9 (56.7 to 65.0)	62.3 (57.7 to 66.6)	65.5 (60.7 to 70.0)	601 (571 to 633)	1022 (950 to 1090)	1044 (968 to 1117)	1099 (1019 to 1175)
**Saint Lucia**	348 (327 to 373)	427 (254 to 672)	455 (260 to 739)	781 (459 to 1240)	59.0 (56.7 to 61.1)	61.7 (57.6 to 65.4)	62.0 (57.6 to 65.8)	66.9 (62.3 to 70.9)	11 (10 to 11)	12 (11 to 12)	12 (11 to 12)	12 (12 to 13)
**Saint Vincent and the Grenadines**	422 (408 to 438)	491 (326 to 721)	567 (372 to 842)	903 (600 to 1324)	54.6 (52.5 to 56.7)	55.7 (52.2 to 58.6)	56.8 (53.2 to 59.9)	60.2 (56.5 to 63.4)	6 (6 to 6)	6 (6 to 7)	6 (6 to 7)	7 (6 to 7)

**Samoa**	307 (283 to 329)	368 (172 to 683)	416 (195 to 782)	683 (320 to 1277)	47.5 (44.5 to 50.9)	49.4 (44.4 to 54.1)	50.3 (45.1 to 55.0)	53.6 (48.1 to 58.6)	9 (9 to 10)	11 (10 to 12)	11 (10 to 12)	12 (11 to 13)
**Sao Tome and Principe**	178 (166 to 187)	198 (90 to 371)	203 (92 to 386)	389 (175 to 732)	54.7 (51.7 to 58.1)	59.6 (53.5 to 66.5)	59.7 (53.6 to 66.8)	65.2 (58.3 to 72.9)	11 (10 to 11)	17 (15 to 19)	17 (15 to 19)	18 (16 to 20)
**Saudi Arabia**	2672 (2487 to 2867)	2832 (1440 to 4875)	3320 (1601 to 5907)	5284 (2609 to 9306)	71.1 (69.5 to 72.8)	73.7 (67.9 to 79.9)	75.2 (68.6 to 81.9)	80.1 (73.4 to 86.9)	2204 (2153 to 2256)	2642 (2435 to 2863)	2695 (2460 to 2935)	2869 (2630 to 3116)
**Senegal**	75 (71 to 80)	85 (57 to 123)	92 (61 to 133)	159 (103 to 233)	44.2 (42.3 to 46.1)	49.2 (46.6 to 51.8)	49.7 (47.1 to 52.3)	53.4 (50.5 to 56.3)	664 (635 to 692)	1114 (1056 to 1173)	1125 (1067 to 1184)	1209 (1143 to 1274)
**Serbia**	836 (793 to 876)	954 (789 to 1139)	1314 (1090 to 1569)	1714 (1405 to 2072)	64.7 (63.1 to 66.3)	67.7 (65.4 to 70.2)	70.5 (68.0 to 73.1)	73.2 (70.5 to 75.9)	570 (557 to 585)	550 (531 to 570)	573 (553 to 594)	595 (573 to 617)
**Seychelles**	931 (840 to 1018)	1065 (436 to 1843)	1381 (565 to 2391)	2006 (817 to 3482)	59.1 (56.6 to 61.4)	63.6 (57.1 to 68.4)	65.7 (59.0 to 70.7)	69.2 (62.1 to 74.5)	6 (5 to 6)	7 (6 to 7)	7 (6 to 7)	7 (6 to 8)
**Sierra Leone**	131 (125 to 138)	100 (54 to 183)	123 (67 to 222)	174 (88 to 331)	43.2 (40.6 to 46.2)	48.6 (44.6 to 51.9)	48.6 (44.7 to 51.8)	51.0 (46.6 to 54.7)	280 (262 to 299)	470 (432 to 502)	470 (433 to 501)	494 (451 to 529)
**Singapore**	2500 (2328 to 2662)	2609 (1624 to 3813)	3146 (1918 to 4661)	4819 (2964 to 7123)	80.7 (77.7 to 83.4)	80.8 (76.1 to 85.3)	82.6 (77.7 to 87.5)	87.6 (82.3 to 92.7)	315 (303 to 326)	363 (342 to 384)	372 (349 to 393)	394 (370 to 417)
**Slovakia**	1803 (1684 to 1943)	1927 (1431 to 2529)	2722 (1961 to 3690)	3561 (2608 to 4734)	69.4 (67.2 to 71.6)	70.3 (67.1 to 73.1)	73.5 (70.0 to 76.6)	76.3 (72.7 to 79.5)	378 (366 to 390)	372 (355 to 387)	389 (370 to 406)	404 (385 to 421)
**Slovenia**	2453 (2362 to 2559)	2531 (2080 to 3077)	3496 (2730 to 4468)	4799 (3851 to 6001)	79.4 (77.1 to 81.7)	84.3 (81.3 to 87.2)	87.9 (84.4 to 91.3)	91.8 (88.3 to 95.2)	164 (159 to 169)	168 (162 to 174)	176 (169 to 182)	183 (176 to 190)
**Solomon Islands**	151 (139 to 160)	182 (102 to 290)	208 (118 to 331)	342 (193 to 542)	39.5 (36.1 to 43.1)	42.6 (38.5 to 46.5)	43.4 (39.3 to 47.4)	45.8 (41.4 to 49.9)	23 (21 to 25)	32 (29 to 34)	32 (29 to 35)	34 (31 to 37)
**Somalia**	26 (26 to 27)	40 (28 to 61)	58 (40 to 86)	71 (48 to 109)	26.5 (23.8 to 29.6)	27.9 (24.8 to 31.7)	28.6 (25.4 to 32.5)	29.4 (26.1 to 33.5)	268 (240 to 299)	388 (345 to 441)	398 (354 to 452)	410 (364 to 467)
**South Africa**	1022 (999 to 1046)	993 (791 to 1251)	1094 (852 to 1390)	2052 (1606 to 2596)	52.6 (51.0 to 54.2)	54.8 (52.8 to 56.9)	55.5 (53.4 to 57.6)	60.4 (58.1 to 62.7)	2771 (2689 to 2859)	3476 (3348 to 3605)	3520 (3386 to 3654)	3832 (3686 to 3974)
**South Korea**	1793 (1731 to 1855)	1950 (1493 to 2494)	3042 (2209 to 4029)	3606 (2669 to 4712)	80.5 (76.6 to 84.2)	81.1 (76.6 to 85.7)	85.8 (80.8 to 90.9)	87.9 (82.9 to 93.1)	4043 (3843 to 4228)	3975 (3755 to 4201)	4207 (3962 to 4455)	4310 (4065 to 4562)
**South Sudan**	35 (33 to 36)	49 (35 to 69)	79 (58 to 107)	93 (66 to 129)	35.3 (31.2 to 39.5)	38.4 (33.6 to 43.5)	40.4 (35.4 to 45.7)	41.3 (36.2 to 46.8)	463 (409 to 518)	920 (805 to 1042)	967 (848 to 1093)	989 (866 to 1119)
**Spain**	2548 (2463 to 2633)	2689 (2260 to 3143)	3336 (2774 to 3936)	4943 (4120 to 5822)	82.1 (80.7 to 83.5)	85.6 (83.1 to 87.9)	87.9 (85.3 to 90.4)	92.8 (90.1 to 95.4)	3812 (3747 to 3880)	3917 (3803 to 4022)	4024 (3906 to 4136)	4248 (4125 to 4365)
**Sri Lanka**	229 (215 to 241)	275 (177 to 407)	382 (236 to 594)	501 (318 to 754)	67.9 (64.6 to 71.2)	74.6 (69.6 to 79.6)	78.1 (72.7 to 83.7)	80.7 (75.2 to 86.4)	1402 (1335 to 1470)	1546 (1443 to 1651)	1620 (1508 to 1735)	1674 (1559 to 1792)
**Sudan**	102 (91 to 119)	130 (62 to 240)	134 (61 to 253)	244 (113 to 455)	46.2 (44.0 to 48.2)	50.8 (46.3 to 54.7)	50.8 (46.2 to 54.9)	55.3 (50.4 to 59.8)	1779 (1693 to 1858)	2427 (2214 to 2617)	2427 (2210 to 2626)	2644 (2411 to 2859)
**Suriname**	881 (777 to 994)	607 (275 to 1084)	743 (341 to 1334)	1820 (815 to 3266)	55.2 (52.8 to 57.5)	56.9 (51.9 to 61.1)	58.5 (53.4 to 62.9)	64.7 (59.0 to 69.5)	30 (29 to 31)	33 (30 to 36)	34 (31 to 37)	38 (35 to 41)
**Swaziland**	619 (581 to 661)	704 (418 to 1117)	952 (567 to 1508)	1312 (774 to 2084)	49.3 (43.5 to 56.0)	51.7 (45.3 to 59.5)	52.8 (46.2 to 60.7)	55.2 (48.3 to 63.6)	65 (58 to 74)	95 (83 to 109)	97 (85 to 111)	101 (89 to 116)
**Sweden**	4705 (4495 to 4901)	4783 (3879 to 5779)	5819 (4674 to 7102)	8869 (7163 to 10762)	82.8 (80.6 to 84.8)	82.2 (79.7 to 84.8)	84.1 (81.4 to 86.7)	89.3 (86.5 to 92.1)	810 (788 to 830)	886 (859 to 913)	906 (878 to 935)	962 (933 to 992)
**Switzerland**	5750 (5487 to 5980)	5391 (4586 to 6300)	5598 (4685 to 6635)	10704 (9047 to 12571)	85.3 (81.8 to 88.5)	85.9 (82.0 to 89.6)	86.0 (82.1 to 89.8)	94.2 (89.9 to 98.4)	706 (678 to 733)	778 (743 to 812)	779 (743 to 814)	853 (814 to 891)
**Syria**	119 (105 to 133)	131 (67 to 222)	136 (67 to 233)	276 (136 to 477)	67.2 (65.1 to 69.3)	71.0 (65.7 to 76.1)	71.2 (65.7 to 76.5)	78.5 (72.4 to 84.3)	1227 (1189 to 1267)	1698 (1570 to 1818)	1702 (1570 to 1827)	1877 (1730 to 2015)

**Taiwan**	1841 (1740 to 1957)	1951 (1549 to 2436)	2520 (1921 to 3246)	3601 (2787 to 4568)	72.3 (70.1 to 74.5)	74.6 (71.5 to 77.6)	77.1 (73.6 to 80.5)	80.9 (77.4 to 84.4)	1704 (1654 to 1756)	1712 (1641 to 1782)	1769 (1690 to 1848)	1858 (1778 to 1937)
**Tajikistan**	73 (69 to 77)	102 (53 to 170)	111 (56 to 189)	174 (88 to 295)	55.0 (52.3 to 57.7)	58.9 (53.6 to 63.6)	59.1 (53.6 to 64.0)	63.1 (57.3 to 68.2)	463 (439 to 485)	686 (624 to 740)	688 (624 to 745)	734 (667 to 794)
**Tanzania**	115 (105 to 126)	143 (79 to 240)	217 (118 to 366)	257 (139 to 436)	47.8 (45.2 to 50.6)	51.0 (47.0 to 55.2)	53.6 (49.2 to 57.9)	54.9 (50.4 to 59.4)	2530 (2394 to 2676)	4106 (3780 to 4440)	4313 (3961 to 4662)	4419 (4058 to 4784)
**Thailand**	539 (515 to 559)	604 (431 to 822)	849 (601 to 1154)	1128 (798 to 1546)	67.7 (65.7 to 69.8)	70.5 (67.1 to 73.9)	73.6 (70.0 to 77.1)	76.7 (72.9 to 80.4)	4565 (4426 to 4704)	4680 (4455 to 4902)	4885 (4648 to 5117)	5086 (4835 to 5333)
**The Bahamas**	1286 (1176 to 1395)	1353 (925 to 1905)	1648 (1124 to 2332)	2527 (1713 to 3583)	60.5 (57.9 to 63.1)	62.4 (58.8 to 65.6)	64.1 (60.4 to 67.3)	67.8 (63.9 to 71.3)	24 (23 to 25)	29 (27 to 30)	30 (28 to 31)	31 (29 to 33)
**The Gambia**	117 (111 to 125)	142 (80 to 236)	152 (87 to 249)	263 (142 to 450)	50.1 (47.6 to 52.5)	50.8 (47.1 to 55.2)	51.2 (47.6 to 55.6)	55.1 (50.8 to 60.0)	99 (94 to 104)	161 (149 to 175)	162 (150 to 176)	174 (161 to 190)
**Timor-Leste**	92 (84 to 101)	114 (78 to 165)	171 (118 to 245)	212 (143 to 309)	45.2 (41.2 to 50.5)	52.8 (47.5 to 59.5)	55.5 (49.9 to 62.5)	57.1 (51.3 to 64.3)	52 (47 to 58)	75 (68 to 85)	79 (71 to 89)	81 (73 to 91)
**Togo**	44 (40 to 48)	61 (33 to 105)	65 (36 to 113)	108 (59 to 188)	44.6 (42.5 to 46.9)	47.8 (44.2 to 51.1)	47.8 (44.3 to 51.2)	51.4 (47.5 to 55.0)	322 (307 to 339)	511 (473 to 547)	512 (474 to 548)	550 (509 to 589)
**Tonga**	210 (196 to 227)	255 (143 to 410)	430 (238 to 702)	475 (262 to 780)	53.5 (50.6 to 56.6)	55.4 (50.9 to 59.8)	58.4 (53.6 to 63.2)	59.2 (54.3 to 64.1)	6 (5 to 6)	7 (6 to 7)	7 (6 to 8)	7 (6 to 8)
**Trinidad and Tobago**	1274 (1176 to 1391)	1418 (806 to 2189)	1739 (957 to 2745)	2592 (1416 to 4100)	58.1 (55.1 to 60.7)	58.3 (53.7 to 62.0)	59.9 (55.0 to 63.9)	63.1 (57.9 to 67.4)	78 (74 to 81)	76 (70 to 80)	78 (71 to 83)	82 (75 to 87)
**Tunisia**	478 (456 to 511)	561 (453 to 722)	595 (458 to 803)	1015 (803 to 1337)	65.6 (62.6 to 68.7)	68.4 (64.5 to 72.2)	68.8 (64.6 to 73.0)	74.0 (69.6 to 78.3)	730 (697 to 765)	812 (766 to 857)	816 (767 to 866)	878 (827 to 930)
**Turkey**	853 (812 to 908)	950 (590 to 1344)	1615 (998 to 2290)	1762 (1066 to 2518)	66.4 (63.5 to 69.3)	73.5 (68.6 to 77.6)	78.9 (73.7 to 83.3)	79.8 (74.4 to 84.3)	5199 (4970 to 5420)	6455 (6023 to 6817)	6929 (6469 to 7315)	7009 (6537 to 7405)
**Turkmenistan**	345 (319 to 379)	434 (211 to 769)	601 (282 to 1085)	776 (365 to 1399)	54.8 (52.8 to 56.7)	58.9 (53.5 to 64.5)	61.6 (55.9 to 67.6)	63.5 (57.7 to 69.7)	297 (286 to 307)	394 (359 to 432)	412 (374 to 453)	426 (386 to 467)
**Uganda**	96 (85 to 110)	115 (70 to 191)	127 (74 to 215)	210 (118 to 364)	43.3 (40.8 to 45.9)	49.0 (45.2 to 53.0)	49.3 (45.4 to 53.4)	52.8 (48.6 to 57.4)	1694 (1594 to 1794)	3148 (2905 to 3403)	3169 (2917 to 3433)	3395 (3120 to 3686)
**Ukraine**	318 (297 to 338)	365 (274 to 493)	368 (271 to 506)	695 (515 to 946)	62.2 (58.2 to 66.0)	65.6 (60.8 to 70.2)	65.6 (60.7 to 70.3)	71.6 (66.3 to 76.7)	2829 (2649 to 3001)	2790 (2585 to 2987)	2790 (2583 to 2989)	3046 (2818 to 3262)
**United Arab Emirates**	2039 (1898 to 2160)	2189 (1219 to 3589)	2508 (1279 to 4340)	4060 (2187 to 6773)	65.5 (61.8 to 69.2)	68.8 (63.3 to 74.1)	69.9 (63.9 to 75.7)	74.7 (68.5 to 80.6)	622 (587 to 657)	824 (758 to 888)	838 (766 to 907)	894 (821 to 965)
**United Kingdom**	3659 (3546 to 3787)	3744 (2971 to 4530)	4140 (3222 to 5038)	6958 (5464 to 8448)	77.0 (75.8 to 78.1)	78.3 (76.0 to 80.4)	79.2 (76.7 to 81.3)	85.0 (82.5 to 87.4)	4999 (4925 to 5069)	5447 (5287 to 5593)	5507 (5336 to 5659)	5917 (5738 to 6078)
**United States**	8744 (8482 to 8978)	8263 (6284 to 9868)	11948 (8492 to 14499)	15588 (11417 to 18833)	72.6 (71.4 to 73.5)	71.2 (68.7 to 72.8)	74.7 (71.4 to 76.6)	77.4 (74.4 to 79.3)	23242 (22865 to 23543)	24953 (24096 to 25525)	26187 (25057 to 26850)	27150 (26090 to 27820)
**Uruguay**	1706 (1608 to 1805)	1802 (1371 to 2342)	2213 (1647 to 2951)	3357 (2498 to 4477)	64.2 (62.4 to 65.9)	65.4 (62.9 to 67.9)	67.0 (64.3 to 69.6)	71.0 (68.2 to 73.9)	220 (214 to 226)	234 (225 to 243)	240 (230 to 249)	254 (244 to 264)
**Uzbekistan**	258 (249 to 268)	322 (199 to 479)	443 (267 to 675)	578 (354 to 871)	59.6 (56.9 to 62.1)	61.9 (57.6 to 65.3)	64.3 (59.6 to 67.9)	66.8 (62.0 to 70.5)	1791 (1710 to 1866)	2244 (2089 to 2365)	2330 (2161 to 2461)	2422 (2248 to 2555)
**Vanuatu**	138 (127 to 152)	122 (77 to 181)	128 (81 to 192)	307 (196 to 450)	38.4 (34.6 to 41.5)	35.7 (32.2 to 38.7)	35.8 (32.4 to 38.9)	40.4 (36.6 to 43.8)	10 (9 to 11)	13 (12 to 14)	13 (12 to 14)	15 (13 to 16)
**Venezuela**	310 (294 to 326)	229 (97 to 407)	232 (98 to 413)	686 (297 to 1206)	59.5 (56.5 to 62.3)	59.7 (53.9 to 64.1)	59.8 (54.0 to 64.3)	68.1 (61.6 to 73.3)	1848 (1754 to 1935)	2146 (1940 to 2307)	2150 (1942 to 2311)	2451 (2215 to 2637)
**Vietnam**	167 (156 to 180)	209 (144 to 293)	338 (229 to 487)	369 (251 to 525)	60.4 (57.9 to 62.8)	65.1 (61.8 to 68.4)	69.4 (65.8 to 73.0)	70.3 (66.7 to 73.8)	5633 (5404 to 5863)	6664 (6324 to 6995)	7102 (6728 to 7466)	7187 (6820 to 7552)

**Yemen**	38 (33 to 44)	37 (23 to 56)	41 (26 to 62)	78 (48 to 119)	43.6 (40.7 to 46.7)	54.6 (50.6 to 58.5)	55.5 (51.5 to 59.4)	59.7 (55.4 to 64.0)	1201 (1123 to 1287)	2097 (1945 to 2248)	2131 (1977 to 2281)	2294 (2127 to 2457)
**Zambia**	180 (168 to 192)	212 (124 to 343)	226 (133 to 368)	395 (228 to 648)	44.0 (40.2 to 47.9)	49.3 (44.0 to 54.7)	49.3 (43.9 to 54.7)	53.3 (47.5 to 59.2)	710 (648 to 773)	1266 (1129 to 1404)	1266 (1128 to 1404)	1369 (1219 to 1520)
**Zimbabwe**	135 (126 to 145)	124 (60 to 228)	134 (65 to 245)	295 (132 to 555)	44.6 (41.1 to 48.1)	48.2 (43.1 to 53.0)	48.6 (43.6 to 53.5)	53.7 (47.7 to 59.2)	695 (641 to 750)	1109 (993 to 1221)	1121 (1004 to 1233)	1237 (1100 to 1365)

B.4 Table: Future Health Scenarios in 2040

This table contains our projection of the future health scenarios (reference, better and worse scenarios) for pooled health spending per capita, universal health coverage index and the number of lives covered by our predicted universal health care, in 2040.

	Pooled health spending per capita ($)				Universal Health Coverage index				Covered lives			
	2015 Observed	2040 Worse Scenario	2040 Reference Scenario	2040 Better Scenario	2015 Observed	2040 Worse Scenario	2040 Reference Scenario	2040 Better Scenario	2015 Observed	2040 Worse Scenario	2040 Reference Scenario	2040 Better Scenario
									Covered lives (millions)			
**Global**	1036 (999 to 1076)	970 (682 to 1321)	1747 (1137 to 2467)	2837 (1924 to 3963)	59.2 (58.2 to 60.1)	61.8 (58.2 to 65.0)	67.3 (63.0 to 71.0)	71.7 (67.2 to 75.5)	4325 (4250 to 4390)	5447 (5123 to 5722)	5928 (5554 to 6254)	6317 (5916 to 6654)
**World Bank Income Groups**									Covered lives (millions)			
**High-income**	4768 (4605 to 4941)	4827 (3624 to 6147)	7508 (5197 to 9808)	13453 (9809 to 17451)	76.8 (75.7 to 77.6)	77.9 (75.3 to 80.1)	81.6 (78.3 to 84.3)	89.6 (86.2 to 92.4)	893 (880 to 902)	941 (909 to 968)	986 (946 to 1019)	1083 (1041 to 1116)
**Upper-middle-income**	646 (622 to 672)	771 (465 to 1175)	1908 (1128 to 2958)	2588 (1522 to 4016)	65.6 (64.5 to 66.6)	67.5 (63.3 to 71.1)	76.1 (70.9 to 80.3)	79.8 (74.4 to 84.2)	1677 (1649 to 1702)	1793 (1679 to 1887)	2018 (1882 to 2131)	2117 (1975 to 2235)
**Lower-middle-income**	113 (106 to 120)	154 (94 to 242)	300 (184 to 471)	427 (251 to 701)	50.3 (49.1 to 51.5)	56.9 (53.3 to 59.6)	61.9 (57.9 to 65.1)	65.3 (60.9 to 68.7)	1482 (1445 to 1516)	2091 (1960 to 2190)	2274 (2127 to 2394)	2400 (2240 to 2525)
**Low-income**	67 (63 to 72)	81 (39 to 155)	122 (56 to 241)	236 (107 to 467)	42.7 (41.6 to 43.9)	49.0 (44.9 to 53.2)	51.1 (46.7 to 55.7)	56.5 (51.4 to 61.7)	273 (266 to 281)	622 (570 to 676)	649 (593 to 707)	718 (652 to 784)
**GBD Super-regions**									Covered lives (millions)			
**Central Europe, Eastern Europe, and Central Asia**	839 (801 to 885)	944 (619 to 1373)	1275 (789 to 1936)	2594 (1651 to 3849)	63.8 (61.9 to 65.6)	68.3 (64.0 to 72.0)	70.6 (65.6 to 74.9)	78.4 (73.2 to 82.9)	263 (256 to 271)	285 (267 to 300)	294 (274 to 312)	327 (305 to 346)
**GBD high-income**	5036 (4873 to 5208)	5045 (3826 to 6318)	7896 (5511 to 10157)	14059 (10362 to 17916)	77.0 (75.8 to 77.8)	77.5 (75.0 to 79.5)	81.2 (78.1 to 83.6)	89.1 (85.9 to 91.6)	812 (800 to 821)	855 (828 to 878)	896 (862 to 923)	984 (948 to 1011)
**Latin America and Caribbean**	723 (693 to 755)	764 (474 to 1095)	1087 (632 to 1603)	2250 (1311 to 3316)	60.7 (59.5 to 61.7)	63.4 (59.8 to 66.1)	66.3 (62.2 to 69.5)	73.3 (68.8 to 76.7)	344 (337 to 349)	428 (404 to 446)	448 (420 to 469)	495 (464 to 518)
**North Africa and Middle East**	597 (560 to 638)	656 (316 to 1205)	976 (430 to 1872)	1808 (824 to 3438)	59.5 (58.5 to 60.6)	64.5 (59.5 to 69.7)	67.4 (61.6 to 73.5)	74.0 (67.8 to 80.3)	336 (330 to 342)	518 (479 to 560)	542 (495 to 591)	595 (545 to 646)
**South Asia**	74 (71 to 77)	110 (73 to 159)	273 (179 to 392)	294 (190 to 430)	48.8 (47.1 to 50.2)	56.7 (53.6 to 59.0)	63.5 (59.9 to 66.2)	64.6 (60.8 to 67.4)	820 (792 to 844)	1065 (1007 to 1108)	1193 (1124 to 1242)	1213 (1142 to 1265)
**Southeast Asia, East Asia, and Oceania**	439 (423 to 457)	527 (331 to 793)	1828 (1127 to 2783)	1984 (1221 to 3033)	63.8 (62.7 to 64.7)	65.4 (61.8 to 68.6)	76.4 (71.9 to 80.0)	77.8 (73.3 to 81.6)	1320 (1298 to 1340)	1356 (1281 to 1421)	1583 (1491 to 1659)	1614 (1519 to 1692)
**Sub-Saharan Africa**	134 (127 to 142)	130 (69 to 237)	175 (88 to 336)	406 (206 to 770)	45.1 (43.9 to 46.3)	50.5 (45.7 to 55.6)	52.3 (47.2 to 57.9)	58.7 (52.6 to 64.9)	430 (419 to 442)	939 (849 to 1032)	972 (877 to 1076)	1090 (977 to 1206)
**Countries**									Covered lives (thousands)			
**Afghanistan**	39 (38 to 41)	38 (22 to 68)	49 (29 to 83)	118 (64 to 216)	30.8 (27.5 to 35.3)	39.9 (35.5 to 43.9)	41.3 (36.7 to 45.6)	46.3 (41.0 to 51.0)	996 (891 to 1141)	2570 (2289 to 2831)	2666 (2370 to 2943)	2984 (2644 to 3290)
**Albania**	383 (356 to 430)	522 (314 to 787)	1027 (600 to 1584)	1348 (779 to 2109)	66.2 (63.8 to 68.7)	70.3 (65.6 to 74.2)	77.0 (71.8 to 81.7)	80.0 (74.4 to 84.9)	192 (185 to 199)	196 (183 to 207)	215 (200 to 227)	223 (207 to 236)
**Algeria**	744 (715 to 769)	936 (463 to 1630)	1014 (465 to 1833)	2526 (1202 to 4494)	63.2 (60.6 to 65.5)	66.8 (60.4 to 74.0)	67.3 (60.3 to 75.1)	76.6 (68.9 to 85.1)	2511 (2410 to 2604)	3353 (3029 to 3712)	3376 (3025 to 3766)	3841 (3459 to 4268)

	Pooled health spending per capita ($)				Universal Health Coverage index				Covered lives			
	2015 Observed	2040 Worse Scenario	2040 Reference Scenario	2040 Better Scenario	2015 Observed	2040 Worse Scenario	2040 Reference Scenario	2040 Better Scenario	2015 Observed	2040 Worse Scenario	2040 Reference Scenario	2040 Better Scenario
**Andorra**	5897 (5345 to 6447)	4907 (3772 to 6368)	5294 (3903 to 7242)	16369 (12472 to 21556)	81.4 (78.0 to 84.9)	76.3 (72.4 to 80.1)	77.1 (72.9 to 81.1)	90.1 (85.5 to 94.4)	6 (6 to 7)	5 (5 to 6)	5 (5 to 6)	6 (6 to 7)
**Angola**	134 (113 to 160)	125 (42 to 275)	135 (38 to 329)	520 (169 to 1154)	43.7 (38.5 to 48.1)	52.7 (45.5 to 60.2)	53.0 (45.0 to 61.5)	63.9 (54.9 to 73.2)	1097 (966 to 1205)	2817 (2430 to 3217)	2834 (2403 to 3289)	3416 (2935 to 3914)
**Antigua and Barbuda**	921 (860 to 975)	1136 (457 to 2312)	1854 (679 to 4028)	3190 (1202 to 6727)	62.2 (60.0 to 64.6)	63.8 (57.2 to 69.9)	68.0 (60.1 to 75.4)	73.4 (65.4 to 81.0)	6 (5 to 6)	6 (6 to 7)	7 (6 to 8)	7 (7 to 8)
**Argentina**	1193 (1114 to 1274)	1367 (794 to 2109)	1875 (1079 to 2903)	3765 (2120 to 5940)	61.0 (59.2 to 62.8)	60.9 (56.9 to 64.2)	63.7 (59.5 to 67.3)	69.8 (65.0 to 73.9)	2641 (2564 to 2717)	3124 (2920 to 3294)	3269 (3052 to 3450)	3580 (3334 to 3788)
**Armenia**	171 (161 to 182)	270 (140 to 449)	361 (180 to 609)	661 (327 to 1129)	64.0 (62.1 to 66.0)	69.6 (63.9 to 75.3)	72.3 (66.0 to 78.5)	78.7 (71.8 to 85.6)	194 (188 to 200)	213 (195 to 230)	221 (202 to 240)	240 (219 to 261)
**Australia**	3545 (3455 to 3638)	3677 (3065 to 4383)	4867 (3864 to 6096)	10099 (8224 to 12301)	81.5 (79.9 to 83.1)	83.8 (81.4 to 86.1)	86.8 (83.8 to 89.5)	96.2 (93.1 to 99.1)	1937 (1899 to 1974)	2426 (2356 to 2494)	2514 (2426 to 2591)	2787 (2697 to 2870)
**Austria**	4255 (4184 to 4341)	4384 (3696 to 5229)	5438 (4471 to 6597)	12051 (10025 to 14511)	81.4 (79.9 to 83.0)	85.8 (83.3 to 88.1)	88.4 (85.6 to 90.9)	98.6 (95.6 to 101.4)	701 (688 to 715)	760 (737 to 780)	783 (758 to 805)	873 (846 to 898)
**Azerbaijan**	258 (243 to 274)	402 (188 to 729)	517 (228 to 978)	980 (444 to 1816)	56.8 (53.6 to 59.8)	64.7 (58.0 to 71.3)	67.0 (59.7 to 74.1)	73.1 (65.3 to 80.7)	556 (525 to 586)	763 (684 to 841)	790 (704 to 874)	862 (771 to 952)
**Bahrain**	1864 (1742 to 1986)	2058 (972 to 3605)	2281 (958 to 4335)	5763 (2550 to 10581)	67.4 (64.4 to 70.4)	75.4 (68.3 to 82.0)	76.3 (68.2 to 83.9)	86.7 (78.0 to 94.9)	92 (88 to 96)	166 (150 to 180)	168 (150 to 185)	191 (171 to 209)
**Bangladesh**	23 (21 to 26)	36 (22 to 56)	70 (43 to 107)	93 (56 to 145)	53.9 (51.4 to 56.3)	67.6 (62.9 to 71.9)	73.8 (68.7 to 78.7)	76.8 (71.1 to 81.9)	8634 (8233 to 9023)	12177 (11327 to 12957)	13296 (12366 to 14172)	13828 (12814 to 14748)
**Barbados**	676 (621 to 720)	820 (472 to 1205)	880 (484 to 1320)	2270 (1251 to 3401)	62.6 (60.0 to 64.9)	66.6 (61.7 to 70.2)	67.2 (62.1 to 71.0)	76.6 (70.8 to 80.9)	18 (17 to 18)	18 (17 to 19)	18 (17 to 19)	21 (19 to 22)
**Belarus**	804 (769 to 838)	943 (666 to 1312)	971 (662 to 1376)	2603 (1820 to 3648)	69.3 (66.6 to 72.0)	75.8 (71.3 to 79.8)	76.0 (71.2 to 80.2)	87.2 (81.9 to 91.8)	658 (633 to 685)	683 (642 to 719)	684 (641 to 722)	785 (738 to 826)
**Belgium**	4049 (3967 to 4134)	4225 (3414 to 5144)	4960 (3979 to 6117)	11582 (9276 to 14236)	79.5 (77.7 to 81.3)	81.8 (79.0 to 84.4)	83.5 (80.5 to 86.2)	94.0 (90.7 to 96.9)	897 (876 to 918)	967 (934 to 997)	987 (952 to 1018)	1111 (1073 to 1146)
**Belize**	419 (395 to 445)	509 (308 to 799)	595 (320 to 1047)	1443 (846 to 2372)	55.2 (52.3 to 58.0)	58.6 (54.2 to 62.5)	59.7 (54.6 to 64.7)	67.3 (62.1 to 72.3)	20 (19 to 21)	34 (32 to 37)	35 (32 to 38)	40 (37 to 43)
**Benin**	47 (45 to 49)	61 (27 to 116)	61 (27 to 117)	165 (75 to 316)	45.6 (43.6 to 47.5)	50.8 (45.8 to 55.6)	50.8 (45.8 to 55.6)	58.4 (52.9 to 63.8)	503 (481 to 524)	1173 (1058 to 1284)	1173 (1058 to 1284)	1349 (1223 to 1475)
**Bhutan**	228 (215 to 243)	306 (136 to 536)	464 (188 to 838)	835 (353 to 1493)	55.2 (51.9 to 58.4)	65.4 (58.2 to 70.7)	69.1 (61.0 to 75.1)	75.0 (66.2 to 81.3)	44 (41 to 46)	60 (54 to 65)	64 (56 to 69)	69 (61 to 75)
**Bolivia**	331 (316 to 346)	434 (235 to 682)	730 (393 to 1152)	1176 (609 to 1906)	51.6 (48.0 to 55.2)	58.8 (53.1 to 64.0)	63.2 (57.0 to 68.8)	67.4 (60.6 to 73.5)	562 (523 to 601)	975 (880 to 1062)	1047 (945 to 1141)	1117 (1004 to 1218)
**Bosnia and Herzegovina**	761 (723 to 815)	952 (430 to 1670)	1439 (628 to 2559)	2559 (1094 to 4599)	64.7 (62.0 to 67.3)	65.3 (59.0 to 70.2)	68.7 (62.0 to 73.9)	74.6 (67.2 to 80.3)	247 (236 to 257)	205 (186 to 221)	216 (195 to 232)	234 (211 to 252)
**Botswana**	965 (879 to 1091)	1099 (536 to 1851)	1833 (796 to 3358)	3100 (1481 to 5375)	56.9 (50.1 to 67.3)	63.0 (55.0 to 75.4)	67.3 (58.2 to 80.9)	72.5 (63.2 to 86.8)	129 (113 to 152)	208 (182 to 249)	223 (192 to 268)	240 (209 to 287)

	Pooled health spending per capita ($)				Universal Health Coverage index				Covered lives			
	2015 Observed	2040 Worse Scenario	2040 Reference Scenario	2040 Better Scenario	2015 Observed	2040 Worse Scenario	2040 Reference Scenario	2040 Better Scenario	2015 Observed	2040 Worse Scenario	2040 Reference Scenario	2040 Better Scenario
**Brazil**	1024 (994 to 1059)	998 (551 to 1434)	1413 (717 to 2059)	3145 (1615 to 4623)	61.7 (60.4 to 62.7)	63.2 (58.7 to 66.0)	66.2 (61.0 to 69.4)	73.5 (67.8 to 77.2)	12869 (12600 to 13081)	14536 (13521 to 15183)	15247 (14049 to 15976)	16925 (15601 to 17759)
**Brunei**	1963 (1786 to 2154)	1810 (846 to 3261)	1852 (842 to 3429)	6186 (2869 to 11222)	64.5 (61.7 to 67.6)	66.6 (59.7 to 72.8)	66.7 (59.6 to 73.1)	78.8 (70.8 to 86.3)	27 (26 to 29)	34 (31 to 37)	34 (31 to 38)	40 (36 to 44)
**Bulgaria**	856 (818 to 900)	1112 (609 to 1773)	2064 (1101 to 3332)	2890 (1516 to 4718)	62.7 (60.2 to 65.2)	64.1 (59.1 to 68.7)	69.7 (64.0 to 74.8)	73.1 (67.0 to 78.5)	455 (437 to 473)	366 (337 to 392)	398 (365 to 427)	417 (382 to 448)
**Burkina Faso**	60 (58 to 63)	85 (42 to 163)	127 (61 to 246)	226 (101 to 458)	46.0 (44.0 to 48.3)	53.3 (48.6 to 57.8)	56.4 (51.3 to 61.2)	60.9 (54.9 to 66.5)	833 (796 to 874)	1990 (1814 to 2159)	2104 (1913 to 2284)	2272 (2050 to 2483)
**Burundi**	53 (50 to 57)	61 (33 to 114)	68 (37 to 124)	207 (104 to 392)	42.9 (40.4 to 45.9)	50.7 (45.5 to 56.7)	50.7 (45.6 to 56.5)	59.2 (53.0 to 66.4)	481 (452 to 514)	1220 (1096 to 1365)	1220 (1099 to 1361)	1426 (1277 to 1599)
**Cambodia**	82 (74 to 92)	112 (57 to 199)	149 (70 to 276)	272 (133 to 489)	49.2 (47.5 to 51.1)	66.1 (61.2 to 70.6)	68.7 (63.1 to 74.0)	74.6 (68.9 to 79.9)	772 (744 to 801)	1396 (1292 to 1491)	1451 (1333 to 1563)	1576 (1455 to 1687)
**Cameroon**	48 (42 to 56)	73 (36 to 129)	82 (40 to 146)	184 (87 to 330)	44.6 (41.4 to 48.0)	51.0 (46.5 to 55.7)	51.7 (47.1 to 56.5)	57.8 (52.6 to 63.1)	1043 (970 to 1122)	2212 (2017 to 2413)	2243 (2044 to 2449)	2504 (2281 to 2735)
**Canada**	4211 (4117 to 4333)	4317 (3008 to 5649)	6232 (4124 to 8388)	12014 (8153 to 15939)	79.2 (77.9 to 80.5)	80.1 (76.3 to 83.5)	84.2 (79.7 to 88.0)	92.2 (87.4 to 96.2)	2843 (2798 to 2891)	3385 (3222 to 3530)	3556 (3367 to 3718)	3895 (3695 to 4066)
**Cape Verde**	278 (264 to 295)	300 (143 to 525)	311 (126 to 591)	981 (445 to 1757)	61.3 (58.6 to 64.2)	68.5 (62.4 to 73.4)	68.7 (61.2 to 74.6)	79.7 (72.2 to 85.6)	33 (32 to 35)	51 (46 to 54)	51 (45 to 55)	59 (53 to 63)
**Central African Republic**	16 (15 to 16)	25 (8 to 58)	30 (11 to 64)	74 (22 to 180)	29.9 (25.9 to 34.6)	33.5 (27.9 to 39.1)	33.5 (28.3 to 38.6)	37.9 (31.5 to 44.2)	147 (127 to 170)	251 (209 to 293)	251 (212 to 290)	284 (236 to 332)
**Chad**	43 (36 to 49)	42 (16 to 95)	45 (16 to 107)	186 (65 to 423)	36.3 (34.0 to 38.4)	40.6 (36.1 to 45.4)	40.8 (36.2 to 45.9)	49.5 (43.6 to 55.5)	506 (474 to 535)	1411 (1253 to 1579)	1417 (1257 to 1596)	1721 (1517 to 1928)
**Chile**	1315 (1285 to 1351)	1433 (1034 to 1982)	1591 (1051 to 2396)	4139 (2878 to 5962)	70.4 (66.7 to 73.8)	72.4 (67.5 to 77.2)	73.1 (67.7 to 78.6)	83.7 (77.9 to 89.6)	1266 (1199 to 1328)	1469 (1369 to 1567)	1483 (1374 to 1595)	1699 (1580 to 1817)
**China**	522 (505 to 542)	662 (406 to 1011)	2657 (1627 to 4060)	2723 (1650 to 4204)	68.5 (67.2 to 69.6)	70.1 (65.8 to 73.7)	84.8 (79.6 to 89.1)	85.0 (79.7 to 89.6)	93359 (91564 to 94911)	86674 (81337 to 91154)	104773 (98335 to 110157)	105118 (98568 to 110700)
**Colombia**	701 (643 to 754)	826 (525 to 1221)	1215 (760 to 1807)	2276 (1409 to 3412)	64.9 (63.0 to 66.6)	71.9 (67.9 to 75.3)	75.8 (71.5 to 79.6)	82.4 (77.6 to 86.6)	3124 (3032 to 3207)	3791 (3585 to 3975)	4001 (3772 to 4202)	4348 (4093 to 4569)
**Comoros**	35 (31 to 38)	35 (13 to 72)	43 (16 to 85)	128 (50 to 258)	44.7 (42.0 to 47.5)	49.8 (43.2 to 55.7)	50.5 (43.9 to 56.3)	59.1 (51.7 to 65.8)	34 (32 to 36)	50 (43 to 56)	51 (44 to 57)	60 (52 to 66)
**Congo**	100 (91 to 110)	127 (45 to 262)	129 (44 to 270)	399 (135 to 843)	46.1 (42.5 to 49.9)	51.6 (44.7 to 58.3)	51.7 (44.6 to 58.6)	60.3 (52.0 to 68.6)	212 (195 to 229)	467 (404 to 527)	467 (403 to 530)	545 (470 to 620)
**Costa Rica**	1044 (1004 to 1083)	1230 (860 to 1756)	1600 (1055 to 2400)	3354 (2296 to 4849)	68.7 (66.7 to 70.5)	69.7 (66.6 to 73.1)	71.9 (68.1 to 75.8)	79.9 (76.1 to 84.0)	327 (318 to 336)	355 (339 to 372)	366 (347 to 386)	407 (388 to 428)
**Cote d'Ivoire**	69 (48 to 102)	109 (55 to 188)	131 (65 to 229)	282 (136 to 493)	42.6 (40.2 to 44.8)	48.3 (44.8 to 51.5)	48.9 (45.3 to 52.2)	54.4 (50.4 to 58.1)	959 (904 to 1009)	2076 (1924 to 2213)	2101 (1947 to 2243)	2340 (2165 to 2498)
**Croatia**	1477 (1359 to 1600)	1647 (1247 to 2226)	2692 (1887 to 3948)	4615 (3405 to 6485)	72.0 (70.2 to 74.2)	76.4 (73.4 to 79.3)	81.3 (77.5 to 85.3)	87.9 (84.2 to 91.7)	305 (297 to 314)	267 (256 to 277)	284 (271 to 298)	307 (294 to 320)

	Pooled health spending per capita ($)				Universal Health Coverage index				Covered lives			
	2015 Observed	2040 Worse Scenario	2040 Reference Scenario	2040 Better Scenario	2015 Observed	2040 Worse Scenario	2040 Reference Scenario	2040 Better Scenario	2015 Observed	2040 Worse Scenario	2040 Reference Scenario	2040 Better Scenario
**Cuba**	932 (814 to 1056)	1068 (733 to 1422)	1410 (960 to 1896)	3006 (2043 to 4040)	67.1 (65.5 to 68.7)	63.9 (61.3 to 66.4)	66.3 (63.5 to 69.0)	73.7 (70.5 to 76.7)	766 (748 to 784)	664 (636 to 690)	689 (660 to 717)	765 (732 to 796)
**Cyprus**	2205 (1979 to 2434)	2445 (1720 to 3450)	3405 (2354 to 4909)	6676 (4623 to 9605)	77.2 (75.6 to 78.8)	82.9 (79.7 to 86.8)	86.7 (83.0 to 91.0)	95.2 (91.3 to 99.9)	70 (68 to 71)	87 (83 to 91)	91 (87 to 95)	99 (95 to 104)
**Czech Republic**	1911 (1606 to 2318)	2152 (1597 to 2854)	3164 (2258 to 4360)	5840 (4246 to 7899)	75.7 (74.1 to 77.2)	79.5 (77.6 to 81.5)	83.7 (81.3 to 86.0)	91.2 (88.8 to 93.5)	800 (783 to 816)	796 (776 to 815)	838 (814 to 861)	913 (889 to 936)
**Democratic Republic of the Congo**	28 (26 to 30)	29 (12 to 61)	34 (14 to 69)	105 (39 to 225)	42.7 (40.4 to 45.4)	45.5 (40.9 to 50.9)	46.5 (41.9 to 51.7)	53.7 (47.9 to 60.3)	3285 (3110 to 3492)	8974 (8062 to 10047)	9171 (8274 to 10200)	10594 (9457 to 11901)
**Denmark**	4436 (4345 to 4559)	4540 (3688 to 5466)	5575 (4470 to 6797)	12565 (10110 to 15251)	79.0 (76.9 to 81.1)	82.1 (78.9 to 85.0)	84.3 (80.9 to 87.3)	94.4 (90.8 to 97.8)	448 (437 to 461)	503 (484 to 521)	517 (496 to 535)	579 (556 to 600)
**Djibouti**	115 (107 to 124)	160 (58 to 284)	178 (63 to 321)	433 (150 to 784)	45.6 (41.9 to 50.0)	51.8 (44.1 to 58.5)	52.6 (44.6 to 59.5)	59.3 (50.3 to 67.1)	44 (40 to 48)	88 (75 to 100)	89 (76 to 101)	101 (86 to 114)
**Dominica**	428 (412 to 446)	559 (289 to 954)	603 (295 to 1059)	1504 (741 to 2611)	56.4 (54.0 to 58.7)	56.2 (51.2 to 60.7)	56.7 (51.3 to 61.5)	64.3 (58.3 to 69.7)	4 (4 to 4)	5 (4 to 5)	5 (4 to 5)	5 (5 to 6)
**Dominican Republic**	525 (498 to 564)	664 (321 to 1315)	1310 (628 to 2560)	1760 (819 to 3496)	61.5 (58.9 to 64.3)	60.8 (55.2 to 66.8)	66.6 (60.4 to 73.0)	69.4 (62.8 to 76.3)	641 (613 to 671)	734 (666 to 806)	804 (729 to 881)	838 (758 to 921)
**Ecuador**	581 (549 to 618)	752 (393 to 1245)	857 (438 to 1441)	1995 (992 to 3428)	60.5 (58.8 to 62.3)	58.1 (53.6 to 61.8)	59.1 (54.4 to 63.0)	66.4 (60.8 to 71.0)	987 (959 to 1017)	1346 (1242 to 1432)	1369 (1260 to 1460)	1538 (1410 to 1645)
**Egypt**	184 (167 to 202)	264 (155 to 418)	358 (188 to 630)	698 (386 to 1173)	59.9 (57.6 to 62.4)	69.3 (64.9 to 74.0)	72.1 (66.7 to 78.1)	79.2 (73.6 to 85.3)	5415 (5208 to 5639)	8764 (8200 to 9355)	9112 (8436 to 9877)	10006 (9305 to 10782)
**El Salvador**	429 (413 to 446)	542 (396 to 725)	654 (455 to 914)	1467 (1044 to 2011)	62.4 (59.6 to 64.8)	66.6 (63.3 to 69.6)	68.2 (64.6 to 71.3)	76.4 (72.5 to 79.9)	384 (367 to 399)	404 (384 to 422)	414 (392 to 432)	463 (440 to 484)
**Equatorial Guinea**	351 (274 to 464)	496 (115 to 1264)	1106 (288 to 2769)	1396 (331 to 3693)	51.1 (44.3 to 58.9)	59.6 (48.6 to 70.2)	66.9 (55.0 to 78.5)	68.5 (56.1 to 80.7)	42 (36 to 48)	113 (93 to 134)	127 (105 to 150)	131 (107 to 154)
**Eritrea**	18 (16 to 22)	26 (10 to 53)	43 (20 to 76)	71 (26 to 148)	38.9 (36.3 to 41.9)	50.4 (44.3 to 56.0)	53.8 (48.2 to 59.2)	57.5 (50.4 to 64.1)	203 (189 to 219)	396 (348 to 440)	422 (378 to 465)	451 (396 to 503)
**Estonia**	1495 (1480 to 1512)	1719 (1119 to 2457)	2288 (1385 to 3431)	4674 (2988 to 6797)	73.9 (71.8 to 76.0)	80.4 (75.7 to 85.1)	83.5 (77.8 to 88.9)	92.3 (86.6 to 97.8)	97 (94 to 100)	92 (86 to 97)	95 (89 to 101)	105 (99 to 111)
**Ethiopia**	54 (50 to 59)	78 (33 to 164)	181 (63 to 408)	220 (82 to 485)	39.3 (36.4 to 42.1)	51.0 (45.0 to 58.1)	57.2 (49.7 to 65.9)	58.6 (51.3 to 67.3)	3912 (3630 to 4199)	9547 (8435 to 10874)	10708 (9303 to 12338)	10965 (9598 to 12607)
**Federated States of Micronesia**	229 (220 to 237)	154 (79 to 299)	161 (78 to 326)	813 (431 to 1520)	44.6 (40.2 to 49.4)	46.2 (41.0 to 51.5)	46.4 (41.0 to 52.1)	53.6 (47.7 to 59.7)	5 (4 to 5)	5 (4 to 6)	5 (4 to 6)	6 (5 to 6)
**Fiji**	272 (255 to 296)	346 (216 to 545)	491 (261 to 860)	955 (552 to 1602)	46.5 (43.1 to 50.4)	49.4 (44.9 to 55.0)	51.8 (46.5 to 58.0)	56.6 (51.2 to 63.3)	40 (37 to 43)	37 (33 to 41)	38 (34 to 43)	42 (38 to 47)
**Finland**	3292 (3221 to 3368)	3507 (2664 to 4576)	4802 (3588 to 6393)	9599 (7195 to 12734)	84.5 (82.8 to 86.1)	89.1 (85.7 to 92.2)	92.9 (89.2 to 96.3)	102.3 (98.3 to 105.9)	462 (453 to 471)	504 (485 to 521)	525 (504 to 545)	579 (556 to 599)
**France**	4419 (4342 to 4485)	4417 (3825 to 5102)	5369 (4532 to 6352)	12397 (10645 to 14455)	80.1 (78.5 to 81.6)	83.4 (81.2 to 85.3)	85.6 (83.2 to 87.7)	96.1 (93.6 to 98.4)	5171 (5066 to 5263)	5655 (5508 to 5782)	5801 (5639 to 5946)	6515 (6343 to 6668)

	Pooled health spending per capita ($)				Universal Health Coverage index				Covered lives			
	2015 Observed	2040 Worse Scenario	2040 Reference Scenario	2040 Better Scenario	2015 Observed	2040 Worse Scenario	2040 Reference Scenario	2040 Better Scenario	2015 Observed	2040 Worse Scenario	2040 Reference Scenario	2040 Better Scenario
**Gabon**	359 (330 to 387)	411 (204 to 759)	457 (205 to 898)	1270 (596 to 2463)	48.9 (45.5 to 52.5)	58.0 (52.6 to 64.1)	58.7 (52.8 to 65.4)	67.7 (61.1 to 75.2)	84 (79 to 91)	188 (170 to 208)	190 (171 to 212)	219 (198 to 243)
**Georgia**	344 (302 to 395)	485 (178 to 968)	719 (246 to 1506)	1246 (431 to 2593)	58.7 (56.0 to 61.4)	55.5 (49.3 to 60.9)	58.4 (51.4 to 64.5)	63.1 (55.6 to 69.6)	239 (227 to 249)	227 (201 to 249)	239 (210 to 264)	258 (227 to 284)
**Germany**	4839 (4587 to 5196)	4897 (4031 to 5930)	5713 (4639 to 7027)	13588 (11077 to 16717)	78.9 (77.0 to 80.7)	81.4 (79.0 to 83.6)	82.9 (80.3 to 85.1)	93.7 (90.8 to 96.2)	6431 (6278 to 6577)	6530 (6339 to 6706)	6647 (6444 to 6830)	7512 (7286 to 7715)
**Ghana**	144 (135 to 153)	207 (82 to 436)	411 (161 to 853)	544 (202 to 1173)	51.5 (49.2 to 54.0)	57.3 (50.8 to 63.0)	63.2 (56.1 to 69.4)	65.2 (57.4 to 72.1)	1425 (1362 to 1494)	2597 (2302 to 2858)	2864 (2542 to 3147)	2954 (2602 to 3268)
**Greece**	1558 (1425 to 1685)	1811 (1489 to 2248)	2033 (1643 to 2556)	4833 (3899 to 6092)	78.3 (76.5 to 79.9)	81.9 (79.7 to 84.2)	83.2 (80.9 to 85.7)	93.7 (91.1 to 96.4)	854 (835 to 872)	785 (764 to 808)	798 (776 to 822)	898 (874 to 925)
**Grenada**	322 (270 to 383)	448 (203 to 819)	612 (257 to 1188)	1170 (511 to 2226)	54.5 (51.9 to 57.0)	58.1 (53.2 to 62.6)	60.7 (55.2 to 65.9)	66.1 (60.4 to 71.4)	6 (5 to 6)	6 (5 to 6)	6 (5 to 7)	7 (6 to 7)
**Guatemala**	232 (223 to 242)	258 (176 to 365)	332 (209 to 506)	767 (507 to 1118)	53.8 (50.1 to 57.8)	59.8 (55.1 to 64.5)	61.8 (56.7 to 67.0)	68.6 (63.0 to 74.0)	873 (813 to 937)	1407 (1297 to 1519)	1456 (1336 to 1578)	1614 (1483 to 1743)
**Guinea**	60 (58 to 62)	51 (23 to 98)	88 (38 to 168)	138 (55 to 271)	39.2 (36.7 to 41.4)	44.7 (39.7 to 49.9)	46.2 (40.8 to 51.5)	49.1 (43.2 to 55.2)	492 (461 to 520)	1053 (936 to 1177)	1088 (963 to 1214)	1159 (1019 to 1301)
**Guinea-Bissau**	82 (76 to 92)	77 (30 to 171)	88 (36 to 206)	294 (99 to 711)	37.8 (35.3 to 40.5)	42.9 (38.1 to 47.9)	43.6 (38.9 to 49.0)	51.1 (44.8 to 57.8)	70 (66 to 75)	142 (126 to 159)	145 (129 to 163)	170 (149 to 192)
**Guyana**	192 (175 to 211)	261 (126 to 457)	308 (136 to 578)	679 (320 to 1207)	49.7 (47.4 to 52.0)	56.7 (52.0 to 61.1)	57.9 (52.3 to 63.0)	64.7 (59.1 to 69.8)	38 (36 to 40)	43 (40 to 47)	44 (40 to 48)	50 (45 to 54)
**Haiti**	90 (85 to 95)	121 (61 to 228)	136 (72 to 248)	321 (145 to 639)	39.7 (36.2 to 43.3)	47.3 (42.3 to 52.6)	48.2 (43.1 to 53.3)	54.0 (48.1 to 60.3)	436 (398 to 475)	755 (674 to 838)	768 (688 to 850)	862 (766 to 961)
**Honduras**	182 (167 to 201)	254 (149 to 406)	319 (181 to 522)	666 (372 to 1104)	54.3 (50.1 to 58.3)	60.5 (54.9 to 65.9)	62.3 (56.4 to 68.0)	69.0 (62.4 to 75.5)	445 (410 to 478)	691 (628 to 753)	712 (644 to 777)	789 (713 to 863)
**Hungary**	1443 (1388 to 1522)	1657 (1218 to 2202)	2380 (1655 to 3340)	4461 (3175 to 6116)	69.6 (67.4 to 71.8)	74.5 (70.7 to 78.7)	78.3 (73.8 to 83.1)	85.3 (80.7 to 90.3)	688 (667 to 709)	646 (613 to 683)	679 (640 to 721)	740 (700 to 783)
**Iceland**	3504 (3390 to 3615)	3675 (2816 to 4503)	6364 (4790 to 7936)	10118 (7636 to 12582)	85.1 (83.1 to 86.9)	89.5 (86.3 to 92.1)	96.0 (92.5 to 98.9)	102.6 (98.8 to 105.8)	28 (27 to 29)	32 (31 to 33)	35 (33 to 36)	37 (36 to 38)
**India**	84 (81 to 87)	125 (86 to 172)	328 (221 to 461)	335 (225 to 471)	49.0 (47.0 to 50.5)	56.1 (53.5 to 58.3)	63.8 (60.6 to 66.4)	64.0 (60.7 to 66.6)	63760 (61186 to 65787)	79224 (75483 to 82323)	90077 (85492 to 93719)	90334 (85718 to 93990)
**Indonesia**	198 (190 to 209)	262 (179 to 371)	548 (363 to 791)	708 (476 to 1008)	49.7 (48.4 to 51.0)	53.8 (51.0 to 56.4)	59.5 (56.3 to 62.4)	61.7 (58.4 to 64.7)	12728 (12395 to 13052)	15867 (15051 to 16630)	17546 (16593 to 18413)	18178 (17228 to 19065)
**Iran**	693 (663 to 727)	882 (434 to 1708)	1403 (582 to 3069)	2371 (1076 to 4883)	66.8 (63.4 to 70.0)	67.8 (61.4 to 75.3)	71.2 (63.3 to 80.5)	76.9 (69.0 to 86.2)	5375 (5099 to 5632)	7029 (6364 to 7816)	7384 (6564 to 8347)	7974 (7156 to 8941)
**Iraq**	230 (211 to 247)	353 (120 to 706)	549 (171 to 1158)	894 (282 to 1871)	51.4 (48.0 to 55.1)	57.4 (49.8 to 64.5)	60.4 (51.9 to 68.3)	64.8 (55.8 to 73.2)	1964 (1832 to 2104)	5496 (4766 to 6176)	5785 (4973 to 6538)	6205 (5340 to 7011)
**Ireland**	4581 (4336 to 4802)	4675 (3054 to 6646)	8147 (4767 to 12421)	13183 (8251 to 19197)	79.9 (77.5 to 82.0)	85.2 (80.7 to 89.3)	92.0 (86.0 to 97.3)	98.2 (92.4 to 103.3)	369 (358 to 379)	454 (430 to 476)	490 (459 to 519)	523 (492 to 550)

	Pooled health spending per capita ($)				Universal Health Coverage index				Covered lives			
	2015 Observed	2040 Worse Scenario	2040 Reference Scenario	2040 Better Scenario	2015 Observed	2040 Worse Scenario	2040 Reference Scenario	2040 Better Scenario	2015 Observed	2040 Worse Scenario	2040 Reference Scenario	2040 Better Scenario
**Israel**	1963 (1807 to 2105)	2157 (1738 to 2660)	2880 (2159 to 3831)	6008 (4685 to 7659)	76.1 (72.9 to 79.1)	80.3 (77.0 to 83.5)	83.3 (79.6 to 86.8)	92.4 (88.6 to 96.1)	614 (588 to 638)	944 (906 to 982)	979 (936 to 1021)	1087 (1042 to 1130)
**Italy**	2661 (2577 to 2742)	2895 (2375 to 3475)	3599 (2902 to 4366)	7875 (6368 to 9554)	80.5 (78.8 to 82.1)	84.8 (82.1 to 87.3)	87.3 (84.4 to 89.9)	97.3 (94.1 to 100.3)	4851 (4750 to 4947)	4660 (4510 to 4796)	4796 (4637 to 4942)	5347 (5170 to 5510)
**Jamaica**	382 (349 to 411)	337 (173 to 568)	401 (200 to 696)	1325 (670 to 2265)	61.1 (57.9 to 64.3)	61.9 (56.7 to 66.7)	63.3 (57.7 to 68.6)	74.7 (68.3 to 80.7)	175 (166 to 184)	188 (172 to 202)	192 (175 to 208)	227 (207 to 245)
**Japan**	3719 (3599 to 3897)	3862 (3144 to 4823)	4205 (3372 to 5306)	10687 (8655 to 13408)	82.4 (81.0 to 83.5)	84.0 (82.0 to 85.8)	84.8 (82.7 to 86.8)	96.6 (94.3 to 98.8)	10350 (10169 to 10493)	8850 (8638 to 9041)	8936 (8708 to 9144)	10178 (9929 to 10402)
**Jordan**	555 (504 to 604)	674 (385 to 1037)	781 (408 to 1286)	1845 (986 to 3011)	65.2 (61.5 to 68.9)	71.7 (66.0 to 77.2)	73.1 (66.7 to 79.2)	82.3 (75.2 to 88.9)	494 (465 to 521)	808 (743 to 870)	823 (751 to 892)	926 (847 to 1001)
**Kazakhstan**	638 (621 to 654)	813 (442 to 1303)	1148 (593 to 1963)	2147 (1136 to 3505)	61.8 (59.1 to 64.6)	69.6 (63.8 to 74.7)	72.8 (66.4 to 78.7)	79.5 (72.7 to 85.6)	1090 (1041 to 1139)	1635 (1501 to 1756)	1712 (1561 to 1850)	1868 (1710 to 2013)
**Kenya**	131 (129 to 133)	173 (119 to 252)	216 (146 to 321)	475 (328 to 693)	54.4 (51.9 to 57.4)	59.9 (55.9 to 63.8)	61.7 (57.6 to 65.9)	68.6 (64.0 to 73.2)	2471 (2357 to 2607)	4543 (4240 to 4840)	4684 (4367 to 4997)	5208 (4857 to 5554)
**Kiribati**	180 (162 to 202)	237 (163 to 352)	327 (219 to 492)	666 (468 to 973)	40.5 (37.4 to 43.2)	45.7 (42.2 to 48.8)	47.3 (43.7 to 50.7)	52.4 (48.5 to 56.0)	5 (4 to 5)	6 (6 to 7)	7 (6 to 7)	7 (7 to 8)
**Kuwait**	2237 (2028 to 2461)	1997 (662 to 3734)	2018 (549 to 4048)	7121 (2309 to 13385)	71.6 (67.9 to 75.6)	74.7 (64.7 to 82.5)	74.7 (63.2 to 83.3)	89.1 (77.1 to 98.6)	274 (260 to 290)	362 (314 to 400)	362 (307 to 404)	432 (374 to 478)
**Kyrgyzstan**	164 (148 to 192)	199 (94 to 343)	205 (83 to 385)	589 (258 to 1071)	58.7 (57.0 to 60.6)	63.5 (57.7 to 67.5)	63.5 (56.5 to 68.2)	73.5 (66.3 to 78.2)	346 (336 to 357)	536 (487 to 570)	536 (477 to 576)	621 (560 to 661)
**Laos**	99 (85 to 115)	139 (72 to 249)	327 (172 to 574)	368 (180 to 690)	42.5 (39.6 to 45.3)	59.3 (53.8 to 65.3)	67.0 (61.0 to 73.6)	67.6 (61.1 to 74.9)	300 (280 to 320)	732 (664 to 805)	827 (753 to 908)	834 (754 to 924)
**Latvia**	1051 (1004 to 1103)	1284 (918 to 1793)	1918 (1304 to 2774)	3391 (2356 to 4843)	68.6 (66.5 to 70.7)	74.4 (70.6 to 78.6)	78.6 (74.3 to 83.3)	85.0 (80.5 to 89.9)	137 (132 to 141)	129 (122 to 136)	136 (128 to 144)	147 (139 to 156)
**Lebanon**	820 (743 to 916)	809 (294 to 1644)	941 (322 to 1987)	2760 (945 to 5759)	73.6 (70.9 to 76.0)	79.4 (69.8 to 88.5)	80.9 (71.0 to 90.9)	93.9 (82.1 to 105.2)	419 (404 to 433)	362 (318 to 404)	369 (324 to 415)	428 (375 to 480)
**Lesotho**	217 (207 to 229)	297 (175 to 479)	562 (331 to 909)	811 (471 to 1329)	41.8 (37.5 to 46.7)	39.5 (35.2 to 44.0)	42.3 (37.8 to 47.2)	44.6 (39.7 to 49.7)	88 (79 to 99)	112 (100 to 125)	121 (108 to 134)	127 (113 to 141)
**Liberia**	454 (450 to 459)	239 (129 to 460)	302 (170 to 569)	702 (358 to 1406)	45.8 (43.8 to 47.9)	44.0 (40.2 to 48.4)	45.5 (41.8 to 50.0)	50.0 (45.4 to 55.3)	206 (197 to 215)	348 (318 to 383)	360 (331 to 395)	396 (359 to 438)
**Libya**	304 (265 to 362)	408 (209 to 789)	637 (314 to 1232)	1098 (522 to 2190)	64.4 (62.0 to 66.7)	75.2 (69.1 to 81.8)	80.1 (73.6 to 87.2)	85.7 (78.4 to 93.8)	395 (380 to 409)	487 (447 to 530)	518 (476 to 564)	555 (507 to 607)
**Lithuania**	1313 (1251 to 1379)	1565 (1020 to 2230)	2440 (1469 to 3621)	4179 (2632 to 6081)	67.4 (65.9 to 68.9)	68.3 (64.5 to 71.7)	72.4 (67.6 to 76.6)	78.1 (73.5 to 82.4)	197 (192 to 201)	169 (160 to 178)	179 (167 to 190)	193 (182 to 204)
**Luxembourg**	5836 (5549 to 6085)	5799 (4388 to 7387)	9860 (7039 to 13125)	16330 (12065 to 21186)	82.2 (80.1 to 84.2)	85.7 (82.4 to 88.7)	92.1 (88.0 to 95.7)	98.8 (94.7 to 102.5)	47 (45 to 48)	62 (59 to 64)	66 (63 to 69)	71 (68 to 74)
**Macedonia**	600 (421 to 895)	717 (490 to 1015)	768 (488 to 1156)	1994 (1316 to 2894)	63.2 (61.3 to 65.0)	68.2 (65.9 to 70.3)	68.8 (66.6 to 70.9)	78.2 (75.6 to 80.6)	131 (127 to 135)	133 (128 to 137)	134 (129 to 138)	152 (147 to 157)
**Madagascar**	60 (56 to 65)	84 (36 to 145)	88 (38 to 152)	233 (98 to 405)	38.4 (35.3 to 41.7)	43.0 (37.7 to 48.2)	43.1 (37.9 to 48.3)	49.4 (43.2 to 55.4)	932 (857 to 1012)	1790 (1570 to 2005)	1795 (1578 to 2010)	2056 (1797 to 2304)

	Pooled health spending per capita ($)				Universal Health Coverage index				Covered lives			
	2015 Observed	2040 Worse Scenario	2040 Reference Scenario	2040 Better Scenario	2015 Observed	2040 Worse Scenario	2040 Reference Scenario	2040 Better Scenario	2015 Observed	2040 Worse Scenario	2040 Reference Scenario	2040 Better Scenario
**Malawi**	124 (121 to 127)	150 (75 to 284)	166 (86 to 311)	460 (201 to 909)	48.3 (45.0 to 52.2)	51.6 (46.2 to 58.0)	52.3 (46.9 to 58.6)	60.2 (53.2 to 68.1)	840 (781 to 907)	1828 (1635 to 2053)	1852 (1660 to 2074)	2130 (1884 to 2411)
**Malaysia**	680 (654 to 709)	828 (588 to 1117)	1328 (882 to 1861)	2224 (1545 to 3041)	63.7 (62.2 to 65.2)	65.9 (62.8 to 68.4)	70.1 (66.3 to 73.2)	75.4 (71.7 to 78.5)	1940 (1894 to 1986)	2480 (2366 to 2577)	2639 (2497 to 2758)	2840 (2699 to 2956)
**Maldives**	1517 (1362 to 1693)	1720 (942 to 2677)	2015 (1087 to 3184)	4713 (2526 to 7450)	72.0 (69.0 to 75.0)	72.6 (67.6 to 77.2)	73.7 (68.3 to 78.5)	83.3 (77.2 to 88.7)	26 (25 to 27)	32 (30 to 34)	32 (30 to 34)	36 (34 to 39)
**Mali**	58 (54 to 62)	86 (37 to 170)	126 (50 to 253)	219 (84 to 448)	43.6 (40.6 to 46.8)	50.9 (45.0 to 56.6)	53.4 (46.8 to 59.5)	57.7 (50.5 to 64.4)	759 (707 to 814)	1927 (1704 to 2143)	2023 (1775 to 2253)	2187 (1913 to 2440)
**Malta**	2295 (2238 to 2347)	2619 (2109 to 3143)	5726 (4529 to 6949)	6960 (5516 to 8427)	76.6 (73.9 to 79.4)	80.7 (77.1 to 84.3)	90.2 (86.1 to 94.2)	92.3 (88.2 to 96.5)	32 (31 to 33)	34 (32 to 35)	38 (36 to 39)	39 (37 to 40)
**Marshall Islands**	525 (486 to 574)	462 (184 to 853)	470 (155 to 915)	1795 (691 to 3312)	43.4 (39.9 to 47.1)	48.7 (43.1 to 53.4)	48.7 (42.4 to 53.8)	57.6 (50.7 to 63.0)	3 (3 to 3)	5 (5 to 6)	5 (5 to 6)	6 (5 to 7)
**Mauritania**	95 (85 to 106)	136 (50 to 301)	144 (49 to 327)	375 (124 to 860)	49.9 (46.3 to 54.1)	59.0 (51.6 to 67.9)	59.4 (51.5 to 68.6)	67.6 (58.5 to 78.3)	198 (184 to 215)	392 (343 to 451)	394 (342 to 455)	449 (388 to 520)
**Mauritius**	517 (493 to 543)	691 (469 to 985)	1104 (708 to 1603)	1736 (1172 to 2477)	64.6 (62.2 to 66.9)	68.1 (64.2 to 71.3)	72.3 (67.9 to 76.0)	77.2 (72.7 to 80.9)	82 (79 to 85)	87 (82 to 92)	93 (87 to 98)	99 (93 to 104)
**Mexico**	634 (608 to 656)	793 (617 to 1002)	1109 (797 to 1511)	2118 (1597 to 2770)	59.8 (58.3 to 61.1)	62.8 (60.7 to 64.7)	65.7 (62.8 to 68.2)	71.9 (69.2 to 74.3)	7602 (7420 to 7769)	9710 (9379 to 10007)	10154 (9715 to 10546)	11109 (10692 to 11481)
**Moldova**	297 (271 to 320)	402 (220 to 634)	470 (253 to 751)	1037 (553 to 1663)	62.9 (60.9 to 65.0)	68.4 (63.4 to 72.9)	69.9 (64.6 to 74.7)	77.6 (71.7 to 83.0)	256 (248 to 265)	253 (235 to 270)	259 (239 to 277)	288 (266 to 308)
**Mongolia**	303 (281 to 327)	401 (208 to 677)	579 (273 to 1024)	1048 (531 to 1789)	58.4 (55.5 to 61.2)	65.8 (60.4 to 70.2)	68.9 (62.6 to 74.1)	75.0 (68.6 to 80.2)	174 (166 to 183)	283 (259 to 302)	296 (269 to 318)	322 (295 to 345)
**Montenegro**	666 (640 to 698)	829 (648 to 1036)	960 (730 to 1226)	2207 (1718 to 2787)	69.1 (67.2 to 70.8)	75.6 (72.9 to 78.3)	77.0 (74.0 to 79.8)	86.5 (83.3 to 89.6)	43 (42 to 44)	46 (44 to 48)	47 (45 to 48)	53 (51 to 54)
**Morocco**	213 (198 to 233)	318 (206 to 517)	520 (333 to 856)	819 (520 to 1355)	57.8 (55.0 to 60.2)	63.7 (59.2 to 69.3)	67.8 (63.0 to 74.0)	72.3 (66.9 to 78.9)	1924 (1831 to 2005)	2184 (2031 to 2376)	2325 (2159 to 2536)	2477 (2295 to 2705)
**Mozambique**	67 (66 to 69)	96 (55 to 171)	143 (75 to 268)	266 (145 to 486)	45.2 (42.0 to 48.2)	51.4 (46.4 to 57.1)	52.5 (47.1 to 58.7)	57.4 (51.6 to 64.0)	1267 (1179 to 1351)	2900 (2618 to 3225)	2962 (2656 to 3312)	3237 (2913 to 3613)
**Myanmar**	86 (76 to 96)	150 (92 to 232)	377 (234 to 577)	428 (263 to 659)	48.9 (46.4 to 51.1)	52.9 (49.4 to 55.9)	59.8 (55.9 to 63.2)	60.9 (56.8 to 64.3)	2645 (2509 to 2762)	3395 (3171 to 3593)	3843 (3593 to 4062)	3910 (3650 to 4132)
**Namibia**	945 (875 to 1019)	957 (644 to 1412)	1098 (690 to 1728)	2988 (1958 to 4502)	54.8 (50.3 to 61.0)	62.9 (57.3 to 70.7)	64.0 (58.0 to 72.3)	73.4 (66.7 to 82.6)	134 (123 to 149)	268 (244 to 301)	273 (247 to 308)	313 (284 to 352)
**Nepal**	68 (64 to 72)	89 (49 to 149)	121 (66 to 208)	242 (131 to 412)	51.2 (48.4 to 53.9)	61.3 (56.7 to 65.4)	64.0 (59.0 to 68.6)	70.0 (64.5 to 75.0)	1518 (1435 to 1599)	2592 (2399 to 2767)	2708 (2496 to 2900)	2961 (2729 to 3171)
**Netherlands**	4902 (4682 to 5177)	4900 (3910 to 6082)	6396 (5086 to 7972)	13593 (10741 to 17075)	81.9 (80.1 to 83.8)	84.3 (81.6 to 86.7)	87.2 (84.4 to 89.7)	96.9 (93.6 to 99.8)	1396 (1364 to 1427)	1471 (1425 to 1513)	1522 (1473 to 1566)	1692 (1635 to 1742)
**New Zealand**	3189 (2988 to 3424)	3326 (2720 to 4003)	4355 (3472 to 5400)	9233 (7433 to 11266)	77.6 (75.6 to 79.5)	79.6 (77.0 to 82.0)	82.4 (79.6 to 85.1)	91.6 (88.4 to 94.4)	350 (341 to 359)	415 (402 to 428)	430 (415 to 444)	478 (461 to 492)
**Nicaragua**	283 (263 to 304)	376 (233 to 587)	411 (242 to 662)	996 (600 to 1584)	64.4 (61.7 to 67.1)	70.6 (65.9 to 74.9)	71.0 (65.9 to 75.6)	80.6 (75.0 to 85.7)	393 (376 to 409)	515 (480 to 546)	518 (481 to 551)	588 (547 to 625)

	Pooled health spending per capita ($)				Universal Health Coverage index				Covered lives			
	2015 Observed	2040 Worse Scenario	2040 Reference Scenario	2040 Better Scenario	2015 Observed	2040 Worse Scenario	2040 Reference Scenario	2040 Better Scenario	2015 Observed	2040 Worse Scenario	2040 Reference Scenario	2040 Better Scenario
**Niger**	30 (29 to 32)	46 (23 to 75)	48 (24 to 79)	117 (58 to 195)	42.3 (39.6 to 45.1)	52.4 (47.1 to 57.5)	52.6 (47.4 to 57.7)	59.5 (53.3 to 65.5)	816 (764 to 870)	2509 (2256 to 2755)	2521 (2271 to 2767)	2850 (2556 to 3136)
**Nigeria**	57 (51 to 62)	66 (17 to 184)	73 (19 to 238)	252 (55 to 766)	47.8 (44.7 to 51.2)	51.6 (43.7 to 59.8)	52.1 (44.5 to 61.8)	61.7 (51.6 to 72.5)	8607 (8057 to 9237)	17609 (14906 to 20427)	17795 (15179 to 21107)	21059 (17628 to 24755)
**North Korea**	60 (54 to 67)	53 (46 to 61)	54 (47 to 62)	246 (208 to 293)	56.2 (53.5 to 58.6)	58.1 (55.4 to 60.6)	58.3 (55.6 to 60.7)	71.8 (68.7 to 74.9)	1482 (1411 to 1545)	1880 (1793 to 1960)	1884 (1798 to 1965)	2323 (2220 to 2424)
**Norway**	6019 (5804 to 6268)	6123 (3725 to 8779)	6920 (4084 to 10079)	16927 (10136 to 24488)	83.1 (81.1 to 85.0)	85.9 (80.7 to 89.7)	86.8 (81.3 to 90.8)	98.7 (92.5 to 103.2)	431 (421 to 441)	533 (501 to 557)	539 (505 to 564)	613 (575 to 641)
**Oman**	1576 (1449 to 1707)	1778 (944 to 2898)	2087 (1015 to 3633)	4989 (2569 to 8264)	74.7 (72.8 to 76.5)	79.5 (74.0 to 85.1)	81.2 (74.7 to 87.7)	91.4 (84.9 to 98.0)	339 (331 to 348)	517 (481 to 553)	528 (486 to 570)	594 (552 to 637)
**Pakistan**	51 (47 to 55)	85 (40 to 159)	127 (58 to 239)	214 (100 to 403)	42.7 (40.0 to 45.4)	51.3 (45.8 to 56.6)	54.1 (48.2 to 59.8)	58.2 (52.0 to 64.4)	8010 (7505 to 8506)	12466 (11141 to 13767)	13141 (11714 to 14533)	14147 (12638 to 15663)
**Palestine**	233 (203 to 266)	299 (196 to 453)	420 (265 to 654)	823 (513 to 1283)	58.7 (56.7 to 60.5)	61.5 (59.0 to 63.7)	64.4 (61.4 to 67.1)	70.6 (67.3 to 73.5)	295 (285 to 304)	810 (777 to 839)	849 (809 to 883)	930 (887 to 968)
**Panama**	1102 (1041 to 1169)	1304 (853 to 1825)	2755 (1668 to 4092)	3596 (2243 to 5216)	62.2 (59.6 to 64.8)	62.5 (58.7 to 65.9)	69.2 (64.6 to 73.2)	71.8 (67.2 to 75.8)	243 (233 to 253)	302 (284 to 319)	334 (312 to 354)	347 (325 to 367)
**Papua New Guinea**	114 (107 to 124)	131 (80 to 198)	133 (78 to 202)	434 (259 to 653)	38.3 (34.7 to 42.1)	43.1 (38.6 to 47.2)	43.1 (38.6 to 47.2)	50.2 (45.0 to 55.0)	294 (266 to 323)	546 (489 to 599)	547 (490 to 599)	637 (571 to 698)
**Paraguay**	470 (439 to 506)	602 (345 to 966)	1310 (722 to 2205)	1623 (876 to 2757)	55.5 (53.2 to 57.8)	54.3 (50.3 to 57.7)	60.5 (55.9 to 64.6)	62.2 (57.2 to 66.3)	367 (351 to 382)	427 (396 to 454)	476 (440 to 508)	489 (450 to 522)
**Peru**	471 (458 to 485)	593 (373 to 879)	899 (548 to 1377)	1600 (978 to 2445)	65.3 (62.3 to 68.2)	72.3 (67.4 to 77.1)	76.6 (71.2 to 81.8)	82.8 (76.8 to 88.4)	2076 (1980 to 2169)	3476 (3241 to 3709)	3685 (3422 to 3936)	3982 (3694 to 4254)
**Philippines**	154 (150 to 159)	204 (135 to 296)	413 (257 to 629)	559 (359 to 831)	49.0 (46.6 to 51.1)	52.0 (48.5 to 54.8)	57.1 (52.9 to 60.6)	59.6 (55.4 to 63.0)	4940 (4702 to 5161)	7596 (7086 to 8014)	8354 (7734 to 8853)	8714 (8104 to 9216)
**Poland**	1339 (1243 to 1464)	1538 (1202 to 1955)	2683 (1977 to 3536)	4251 (3230 to 5528)	71.4 (69.2 to 73.3)	77.4 (74.7 to 79.8)	83.5 (80.1 to 86.4)	89.0 (85.7 to 91.9)	2751 (2667 to 2825)	2652 (2561 to 2734)	2863 (2747 to 2962)	3049 (2938 to 3151)
**Portugal**	1962 (1859 to 2078)	2198 (1722 to 2736)	3454 (2536 to 4534)	6066 (4617 to 7739)	76.5 (74.9 to 77.9)	83.0 (80.3 to 85.5)	88.4 (85.1 to 91.5)	95.4 (92.0 to 98.5)	801 (785 to 816)	745 (721 to 768)	794 (764 to 822)	856 (826 to 884)
**Qatar**	3018 (2798 to 3248)	3012 (1244 to 6534)	3285 (1024 to 8089)	9430 (3694 to 20979)	76.6 (72.1 to 80.9)	83.8 (74.6 to 94.4)	84.3 (72.8 to 96.8)	98.0 (86.9 to 110.6)	170 (160 to 180)	278 (248 to 313)	280 (242 to 322)	326 (289 to 367)
**Romania**	890 (820 to 959)	1071 (658 to 1708)	2429 (1446 to 3917)	2921 (1745 to 4685)	65.6 (63.6 to 67.5)	68.9 (64.3 to 73.6)	76.9 (71.6 to 82.4)	78.9 (73.6 to 84.5)	1277 (1238 to 1314)	1193 (1113 to 1276)	1332 (1241 to 1427)	1368 (1275 to 1464)
**Russian Federation**	993 (984 to 1003)	1167 (742 to 1708)	1179 (679 to 1842)	3233 (1977 to 4840)	62.3 (57.8 to 66.5)	67.8 (61.4 to 74.0)	67.8 (60.8 to 74.3)	78.0 (70.4 to 85.5)	9024 (8383 to 9637)	9450 (8556 to 10315)	9450 (8478 to 10366)	10882 (9818 to 11924)
**Rwanda**	110 (103 to 119)	147 (78 to 257)	213 (108 to 387)	394 (195 to 716)	51.0 (48.4 to 53.7)	64.3 (58.8 to 69.6)	67.8 (61.7 to 73.7)	73.1 (66.4 to 79.4)	601 (571 to 633)	1281 (1171 to 1386)	1349 (1228 to 1467)	1456 (1323 to 1581)
**Saint Lucia**	348 (327 to 373)	476 (211 to 845)	531 (207 to 991)	1288 (575 to 2312)	59.0 (56.7 to 61.1)	63.1 (57.0 to 68.1)	63.7 (56.7 to 69.3)	72.3 (65.4 to 78.2)	11 (10 to 11)	11 (10 to 12)	12 (10 to 13)	13 (12 to 14)
**Saint Vincent and the Grenadines**	422 (408 to 438)	540 (312 to 895)	693 (385 to 1180)	1473 (851 to 2445)	54.6 (52.5 to 56.7)	56.0 (51.9 to 59.9)	58.0 (53.4 to 62.3)	64.1 (59.3 to 68.7)	6 (6 to 6)	6 (6 to 7)	7 (6 to 7)	7 (7 to 8)

	Pooled health spending per capita ($)				Universal Health Coverage index				Covered lives			
	2015 Observed	2040 Worse Scenario	2040 Reference Scenario	2040 Better Scenario	2015 Observed	2040 Worse Scenario	2040 Reference Scenario	2040 Better Scenario	2015 Observed	2040 Worse Scenario	2040 Reference Scenario	2040 Better Scenario
**Samoa**	307 (283 to 329)	409 (150 to 835)	519 (194 to 1083)	1135 (416 to 2319)	47.5 (44.5 to 50.9)	50.3 (44.0 to 56.1)	52.0 (45.6 to 58.1)	57.7 (50.5 to 64.4)	9 (9 to 10)	13 (11 to 15)	13 (12 to 15)	15 (13 to 17)
**Sao Tome and Principe**	178 (166 to 187)	224 (64 to 563)	241 (66 to 634)	667 (177 to 1717)	54.7 (51.7 to 58.1)	61.3 (52.3 to 71.4)	61.7 (52.4 to 72.2)	71.1 (60.3 to 83.2)	11 (10 to 11)	21 (18 to 24)	21 (18 to 25)	24 (21 to 29)
**Saudi Arabia**	2672 (2487 to 2867)	2968 (1339 to 6252)	3610 (1402 to 8065)	8238 (3486 to 17803)	71.1 (69.5 to 72.8)	74.5 (67.3 to 83.4)	76.3 (67.9 to 86.5)	85.6 (76.9 to 96.3)	2204 (2153 to 2256)	2693 (2432 to 3014)	2757 (2455 to 3126)	3094 (2778 to 3481)
**Senegal**	75 (71 to 80)	98 (54 to 158)	118 (64 to 194)	266 (140 to 445)	44.2 (42.3 to 46.1)	50.8 (47.1 to 54.3)	52.2 (48.3 to 55.9)	58.2 (53.7 to 62.4)	664 (635 to 692)	1416 (1314 to 1513)	1454 (1347 to 1557)	1622 (1497 to 1740)
**Serbia**	836 (793 to 876)	1034 (796 to 1325)	1683 (1296 to 2172)	2715 (2044 to 3589)	64.7 (63.1 to 66.3)	69.0 (66.2 to 72.1)	73.5 (70.5 to 76.9)	78.7 (75.3 to 82.6)	570 (557 to 585)	521 (500 to 545)	556 (533 to 581)	595 (569 to 624)
**Seychelles**	931 (840 to 1018)	1161 (359 to 2414)	1866 (578 to 3878)	3278 (993 to 6855)	59.1 (56.6 to 61.4)	65.7 (57.0 to 72.6)	70.0 (60.8 to 77.3)	75.8 (65.5 to 83.8)	6 (5 to 6)	7 (6 to 8)	8 (7 to 8)	8 (7 to 9)
**Sierra Leone**	131 (125 to 138)	113 (48 to 241)	154 (69 to 323)	302 (113 to 697)	43.2 (40.6 to 46.2)	50.0 (44.8 to 54.7)	50.8 (45.8 to 55.3)	55.7 (49.5 to 61.7)	280 (262 to 299)	600 (538 to 657)	609 (550 to 664)	669 (594 to 741)
**Singapore**	2500 (2328 to 2662)	2713 (1597 to 4180)	3533 (1980 to 5737)	7348 (4195 to 11627)	80.7 (77.7 to 83.4)	80.6 (75.4 to 85.8)	83.3 (77.5 to 89.1)	92.3 (86.1 to 98.7)	315 (303 to 326)	377 (352 to 401)	389 (362 to 417)	432 (403 to 461)
**Slovakia**	1803 (1684 to 1943)	2010 (1384 to 2795)	3549 (2289 to 5206)	5507 (3702 to 7830)	69.4 (67.2 to 71.6)	70.6 (66.9 to 73.8)	76.2 (71.8 to 80.0)	81.0 (76.7 to 84.9)	378 (366 to 390)	351 (332 to 367)	378 (357 to 398)	403 (381 to 422)
**Slovenia**	2453 (2362 to 2559)	2593 (2019 to 3360)	4486 (3132 to 6315)	7376 (5486 to 10004)	79.4 (77.1 to 81.7)	85.5 (82.1 to 88.7)	92.0 (87.5 to 96.3)	98.6 (94.3 to 103.0)	164 (159 to 169)	162 (155 to 168)	174 (166 to 182)	187 (178 to 195)
**Solomon Islands**	151 (139 to 160)	205 (94 to 394)	252 (112 to 488)	578 (266 to 1100)	39.5 (36.1 to 43.1)	43.8 (38.8 to 48.3)	45.1 (39.9 to 49.8)	49.9 (44.2 to 55.0)	23 (21 to 25)	37 (33 to 41)	38 (34 to 42)	42 (37 to 46)
**Somalia**	26 (26 to 27)	48 (28 to 89)	74 (44 to 132)	126 (70 to 236)	26.5 (23.8 to 29.6)	28.4 (25.0 to 33.0)	29.4 (26.0 to 34.1)	31.7 (27.9 to 37.1)	268 (240 to 299)	471 (414 to 547)	488 (430 to 565)	526 (462 to 614)
**South Africa**	1022 (999 to 1046)	949 (703 to 1272)	1096 (786 to 1500)	3203 (2326 to 4323)	52.6 (51.0 to 54.2)	55.0 (52.7 to 57.2)	56.1 (53.5 to 58.4)	65.1 (62.1 to 67.7)	2771 (2689 to 2859)	3841 (3679 to 3993)	3915 (3734 to 4077)	4542 (4333 to 4727)
**South Korea**	1793 (1731 to 1855)	2046 (1459 to 2727)	3960 (2564 to 5683)	5554 (3776 to 7723)	80.5 (76.6 to 84.2)	81.3 (76.5 to 86.2)	88.8 (82.7 to 94.6)	93.2 (87.2 to 99.0)	4043 (3843 to 4228)	3659 (3442 to 3876)	3992 (3720 to 4255)	4191 (3922 to 4455)
**South Sudan**	35 (33 to 36)	57 (36 to 90)	82 (54 to 124)	151 (95 to 236)	35.3 (31.2 to 39.5)	40.0 (34.7 to 45.7)	41.4 (36.0 to 47.2)	45.1 (39.1 to 51.5)	463 (409 to 518)	1386 (1201 to 1583)	1434 (1247 to 1634)	1562 (1356 to 1786)
**Spain**	2548 (2463 to 2633)	2781 (2209 to 3363)	4181 (3262 to 5147)	7556 (5918 to 9252)	82.1 (80.7 to 83.5)	86.7 (83.8 to 89.4)	91.5 (88.3 to 94.6)	99.4 (96.0 to 102.7)	3812 (3747 to 3880)	3876 (3748 to 3999)	4093 (3948 to 4231)	4446 (4291 to 4594)
**Sri Lanka**	229 (215 to 241)	309 (177 to 509)	560 (299 to 994)	828 (462 to 1397)	67.9 (64.6 to 71.2)	76.6 (70.8 to 82.8)	83.3 (76.5 to 90.5)	87.6 (80.7 to 94.7)	1402 (1335 to 1470)	1548 (1431 to 1673)	1684 (1546 to 1829)	1771 (1632 to 1913)
**Sudan**	102 (91 to 119)	145 (56 to 299)	154 (57 to 334)	423 (161 to 896)	46.2 (44.0 to 48.2)	52.3 (47.0 to 57.4)	52.5 (46.8 to 57.9)	60.7 (54.3 to 66.7)	1779 (1693 to 1858)	2686 (2409 to 2943)	2693 (2403 to 2972)	3114 (2784 to 3422)
**Suriname**	881 (777 to 994)	530 (176 to 1127)	762 (266 to 1622)	2893 (934 to 6222)	55.2 (52.8 to 57.5)	56.4 (49.7 to 62.1)	59.5 (52.7 to 65.4)	70.0 (61.5 to 77.0)	30 (29 to 31)	33 (29 to 36)	35 (31 to 38)	41 (36 to 45)
**Swaziland**	619 (581 to 661)	758 (372 to 1332)	1110 (544 to 1937)	2106 (1018 to 3707)	49.3 (43.5 to 56.0)	52.8 (45.6 to 61.1)	54.6 (47.2 to 63.2)	59.8 (51.6 to 69.2)	65 (58 to 74)	115 (100 to 133)	119 (103 to 138)	130 (113 to 151)

	Pooled health spending per capita ($)				Universal Health Coverage index				Covered lives			
	2015 Observed	2040 Worse Scenario	2040 Reference Scenario	2040 Better Scenario	2015 Observed	2040 Worse Scenario	2040 Reference Scenario	2040 Better Scenario	2015 Observed	2040 Worse Scenario	2040 Reference Scenario	2040 Better Scenario
**Sweden**	4705 (4495 to 4901)	4823 (3746 to 6090)	6544 (4976 to 8402)	13306 (10239 to 16936)	82.8 (80.6 to 84.8)	82.0 (79.1 to 84.8)	85.2 (82.0 to 88.2)	94.3 (90.8 to 97.6)	810 (788 to 830)	937 (904 to 969)	973 (936 to 1008)	1077 (1037 to 1115)
**Switzerland**	5750 (5487 to 5980)	5373 (4424 to 6392)	5640 (4499 to 6877)	15936 (12917 to 19156)	85.3 (81.8 to 88.5)	86.2 (82.2 to 90.1)	86.4 (82.2 to 90.3)	100.1 (95.3 to 104.5)	706 (678 to 733)	797 (760 to 833)	799 (760 to 836)	926 (882 to 967)
**Syria**	119 (105 to 133)	127 (57 to 250)	135 (57 to 273)	474 (201 to 960)	67.2 (65.1 to 69.3)	71.3 (64.8 to 78.2)	71.7 (64.8 to 79.0)	85.5 (77.4 to 94.1)	1227 (1189 to 1267)	1867 (1696 to 2047)	1876 (1695 to 2067)	2238 (2026 to 2462)
**Taiwan**	1841 (1740 to 1957)	2040 (1509 to 2758)	2684 (1842 to 3876)	5542 (3973 to 7771)	72.3 (70.1 to 74.5)	75.4 (71.8 to 79.1)	78.1 (73.8 to 82.6)	86.5 (82.0 to 91.1)	1704 (1654 to 1756)	1623 (1545 to 1703)	1681 (1588 to 1778)	1862 (1764 to 1961)
**Tajikistan**	73 (69 to 77)	122 (53 to 227)	135 (56 to 263)	300 (125 to 573)	55.0 (52.3 to 57.7)	60.8 (54.1 to 66.5)	61.1 (54.1 to 67.4)	68.5 (60.8 to 75.3)	463 (439 to 485)	859 (765 to 940)	863 (765 to 952)	968 (859 to 1063)
**Tanzania**	115 (105 to 126)	164 (77 to 323)	307 (140 to 604)	433 (196 to 874)	47.8 (45.2 to 50.6)	52.5 (47.2 to 57.9)	56.8 (51.1 to 62.7)	59.6 (53.4 to 66.0)	2530 (2394 to 2676)	5252 (4727 to 5797)	5688 (5115 to 6272)	5970 (5349 to 6602)
**Thailand**	539 (515 to 559)	650 (425 to 953)	1075 (696 to 1600)	1807 (1139 to 2713)	67.7 (65.7 to 69.8)	71.6 (67.5 to 75.6)	76.5 (71.9 to 80.9)	82.4 (77.3 to 87.2)	4565 (4426 to 4704)	4487 (4230 to 4739)	4795 (4509 to 5069)	5163 (4841 to 5465)
**The Bahamas**	1286 (1176 to 1395)	1422 (898 to 2149)	1847 (1151 to 2823)	3897 (2415 to 6011)	60.5 (57.9 to 63.1)	63.2 (59.1 to 66.7)	65.5 (61.1 to 69.3)	72.5 (67.6 to 76.8)	24 (23 to 25)	32 (29 to 33)	33 (30 to 35)	36 (34 to 38)
**The Gambia**	117 (111 to 125)	162 (74 to 306)	191 (91 to 355)	446 (189 to 879)	50.1 (47.6 to 52.5)	50.9 (46.1 to 56.4)	52.1 (47.4 to 57.6)	58.4 (52.5 to 65.2)	99 (94 to 104)	203 (184 to 225)	207 (189 to 230)	233 (209 to 260)
**Timor-Leste**	92 (84 to 101)	129 (77 to 210)	208 (125 to 335)	359 (207 to 591)	45.2 (41.2 to 50.5)	55.3 (49.2 to 62.9)	58.7 (52.3 to 66.7)	63.3 (56.3 to 71.9)	52 (47 to 58)	92 (82 to 104)	98 (87 to 111)	105 (93 to 119)
**Togo**	44 (40 to 48)	73 (29 to 165)	87 (35 to 195)	193 (77 to 438)	44.6 (42.5 to 46.9)	49.3 (44.1 to 54.7)	50.0 (44.8 to 55.5)	56.0 (50.1 to 62.2)	322 (307 to 339)	654 (585 to 726)	664 (595 to 737)	743 (665 to 826)
**Tonga**	210 (196 to 227)	284 (132 to 525)	642 (293 to 1195)	790 (362 to 1486)	53.5 (50.6 to 56.6)	56.4 (50.8 to 61.8)	62.0 (55.8 to 68.1)	63.8 (57.4 to 70.2)	6 (5 to 6)	7 (6 to 8)	8 (7 to 9)	8 (7 to 9)
**Trinidad and Tobago**	1274 (1176 to 1391)	1530 (713 to 2729)	1937 (842 to 3602)	4107 (1789 to 7606)	58.1 (55.1 to 60.7)	58.4 (52.6 to 63.2)	60.3 (53.8 to 65.7)	66.8 (59.7 to 72.7)	78 (74 to 81)	71 (64 to 76)	73 (65 to 79)	81 (72 to 88)
**Tunisia**	478 (456 to 511)	598 (455 to 898)	655 (455 to 1095)	1647 (1199 to 2601)	65.6 (62.6 to 68.7)	69.3 (65.0 to 74.6)	70.0 (65.1 to 76.7)	79.7 (74.4 to 86.5)	730 (697 to 765)	829 (777 to 892)	837 (778 to 917)	953 (889 to 1034)
**Turkey**	853 (812 to 908)	1023 (506 to 1600)	2321 (1132 to 3639)	2821 (1362 to 4471)	66.4 (63.5 to 69.3)	75.6 (69.0 to 80.8)	84.5 (76.9 to 90.4)	86.8 (78.8 to 93.0)	5199 (4970 to 5420)	7093 (6480 to 7583)	7934 (7222 to 8485)	8149 (7401 to 8727)
**Turkmenistan**	345 (319 to 379)	497 (181 to 979)	869 (298 to 1782)	1281 (443 to 2639)	54.8 (52.8 to 56.7)	60.8 (53.8 to 67.5)	65.7 (57.8 to 73.4)	69.0 (60.8 to 77.1)	297 (286 to 307)	459 (406 to 510)	496 (436 to 554)	521 (459 to 582)
**Uganda**	96 (85 to 110)	127 (65 to 241)	152 (74 to 297)	348 (159 to 698)	43.3 (40.8 to 45.9)	50.5 (46.0 to 55.5)	51.4 (46.7 to 56.8)	57.7 (52.2 to 63.9)	1694 (1594 to 1794)	4212 (3838 to 4626)	4290 (3896 to 4733)	4812 (4358 to 5330)
**Ukraine**	318 (297 to 338)	357 (245 to 519)	366 (240 to 548)	1145 (770 to 1669)	62.2 (58.2 to 66.0)	66.3 (61.1 to 71.4)	66.3 (60.9 to 71.6)	77.9 (71.6 to 84.0)	2829 (2649 to 3001)	2670 (2460 to 2878)	2672 (2453 to 2884)	3137 (2886 to 3382)
**United Arab Emirates**	2039 (1898 to 2160)	2288 (1040 to 4229)	2746 (1048 to 5537)	6300 (2690 to 12055)	65.5 (61.8 to 69.2)	69.8 (62.9 to 76.1)	71.3 (63.3 to 78.6)	80.2 (71.8 to 87.7)	622 (587 to 657)	961 (866 to 1047)	982 (871 to 1081)	1103 (988 to 1207)
**United Kingdom**	3659 (3546 to 3787)	3800 (2809 to 4881)	4439 (3186 to 5835)	10500 (7645 to 13605)	77.0 (75.8 to 78.1)	78.8 (75.9 to 81.6)	80.3 (77.0 to 83.4)	90.6 (87.1 to 93.9)	4999 (4925 to 5069)	5607 (5400 to 5806)	5714 (5476 to 5935)	6447 (6197 to 6680)

	Pooled health spending per capita ($)				Universal Health Coverage index				Covered lives			
	2015 Observed	2040 Worse Scenario	2040 Reference Scenario	2040 Better Scenario	2015 Observed	2040 Worse Scenario	2040 Reference Scenario	2040 Better Scenario	2015 Observed	2040 Worse Scenario	2040 Reference Scenario	2040 Better Scenario
**United States**	8744 (8482 to 8978)	8094 (5930 to 10163)	14876 (9805 to 19166)	22685 (15934 to 29084)	72.6 (71.4 to 73.5)	70.6 (67.9 to 72.6)	76.7 (72.7 to 79.2)	81.3 (77.7 to 83.8)	23242 (22865 to 23543)	25611 (24610 to 26315)	27806 (26369 to 28714)	29488 (28160 to 30408)
**Uruguay**	1706 (1608 to 1805)	1873 (1315 to 2545)	2550 (1725 to 3602)	5181 (3535 to 7280)	64.2 (62.4 to 65.9)	66.0 (63.1 to 68.8)	68.6 (65.2 to 71.9)	75.8 (72.2 to 79.5)	220 (214 to 226)	239 (228 to 249)	248 (236 to 260)	275 (262 to 288)
**Uzbekistan**	258 (249 to 268)	364 (194 to 607)	607 (306 to 1023)	956 (499 to 1619)	59.6 (56.9 to 62.1)	63.1 (57.7 to 67.4)	67.3 (61.0 to 72.2)	71.9 (65.3 to 76.9)	1791 (1710 to 1866)	2512 (2296 to 2682)	2680 (2427 to 2875)	2862 (2599 to 3063)
**Vanuatu**	138 (127 to 152)	116 (60 to 201)	127 (62 to 222)	515 (275 to 860)	38.4 (34.6 to 41.5)	34.2 (30.7 to 37.5)	34.5 (30.8 to 38.0)	42.1 (37.8 to 45.9)	10 (9 to 11)	14 (13 to 16)	15 (13 to 16)	18 (16 to 19)
**Venezuela**	310 (294 to 326)	231 (75 to 451)	240 (79 to 470)	1134 (392 to 2171)	59.5 (56.5 to 62.3)	60.3 (53.0 to 65.8)	60.7 (53.4 to 66.3)	74.0 (65.3 to 80.6)	1848 (1754 to 1935)	2282 (2005 to 2490)	2294 (2018 to 2505)	2798 (2470 to 3048)
**Vietnam**	167 (156 to 180)	239 (148 to 366)	552 (324 to 867)	624 (374 to 974)	60.4 (57.9 to 62.8)	67.2 (62.9 to 71.1)	75.3 (70.2 to 80.0)	76.6 (71.6 to 81.3)	5633 (5404 to 5863)	7049 (6602 to 7466)	7902 (7372 to 8396)	8038 (7515 to 8529)
**Yemen**	38 (33 to 44)	43 (23 to 70)	46 (25 to 75)	136 (71 to 227)	43.6 (40.7 to 46.7)	58.4 (53.8 to 63.0)	59.0 (54.4 to 63.7)	67.8 (62.4 to 73.3)	1201 (1123 to 1287)	2535 (2335 to 2738)	2563 (2364 to 2765)	2945 (2709 to 3184)
**Zambia**	180 (168 to 192)	219 (106 to 407)	246 (119 to 465)	647 (304 to 1231)	44.0 (40.2 to 47.9)	50.6 (44.5 to 56.8)	50.9 (44.7 to 57.2)	58.3 (51.2 to 65.5)	710 (648 to 773)	1749 (1538 to 1963)	1757 (1543 to 1974)	2014 (1767 to 2264)
**Zimbabwe**	135 (126 to 145)	121 (45 to 256)	139 (55 to 289)	491 (165 to 1063)	44.6 (41.1 to 48.1)	48.9 (42.7 to 54.8)	49.9 (43.8 to 55.8)	58.9 (50.7 to 66.1)	695 (641 to 750)	1433 (1250 to 1606)	1461 (1283 to 1636)	1725 (1486 to 1937)

B.5 Figure: Comparison of Future Health Scenarios

This set of figures included show six panels of the dependent variables that we forecasted, starting with UHC, THE, and DAH, GHE, OOP and PPP per capita. Each variable contain their respective reference, better and worse scenarios.

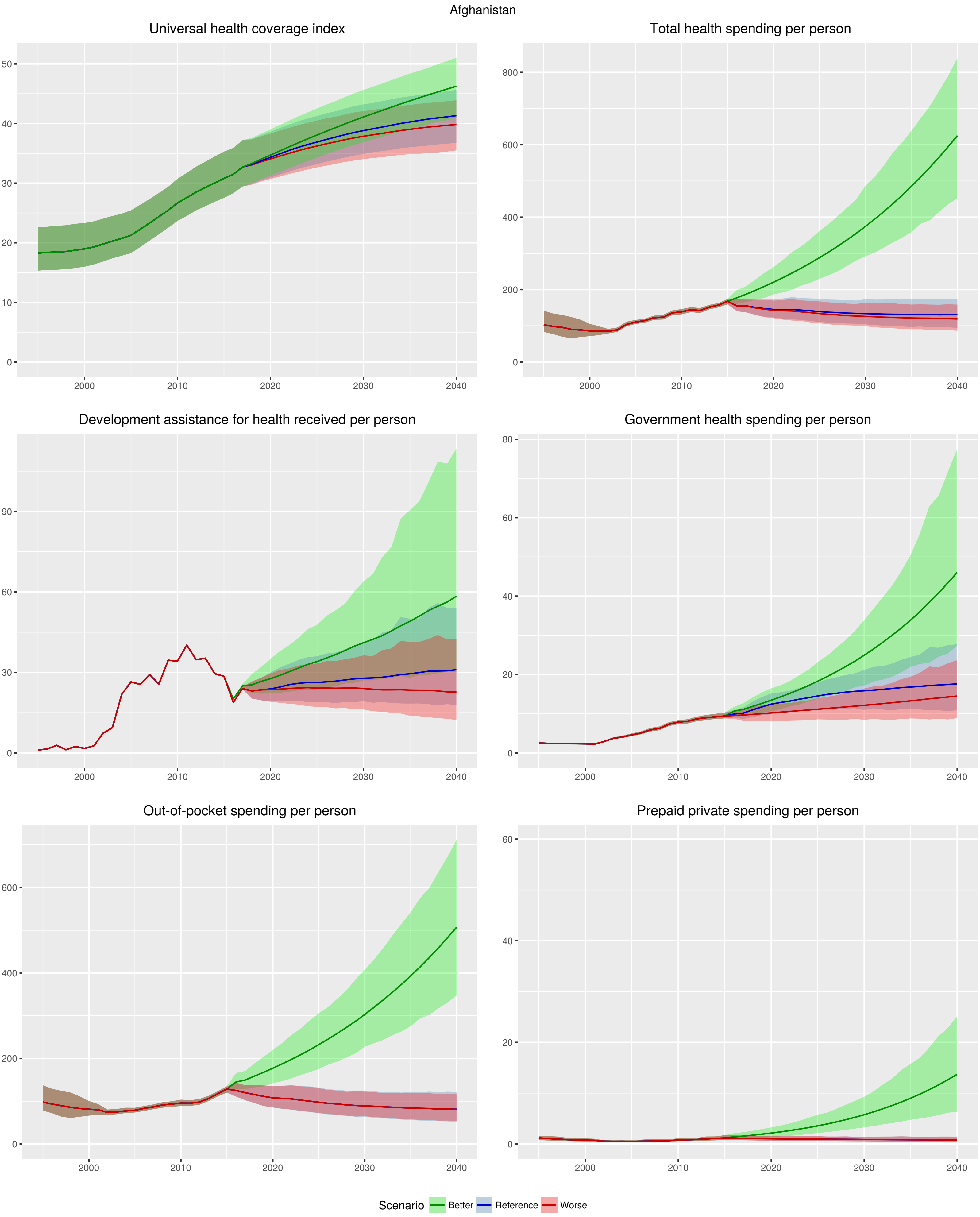
Afghanistan
Universal health coverage index
Total health spending per person
Development assistance for health received per person
Government health spending per person
Out-of-pocket spending per person
Prepaid private spending per person
2000
2010
2020
2030
2040
Scenario
Better
Reference
Worse

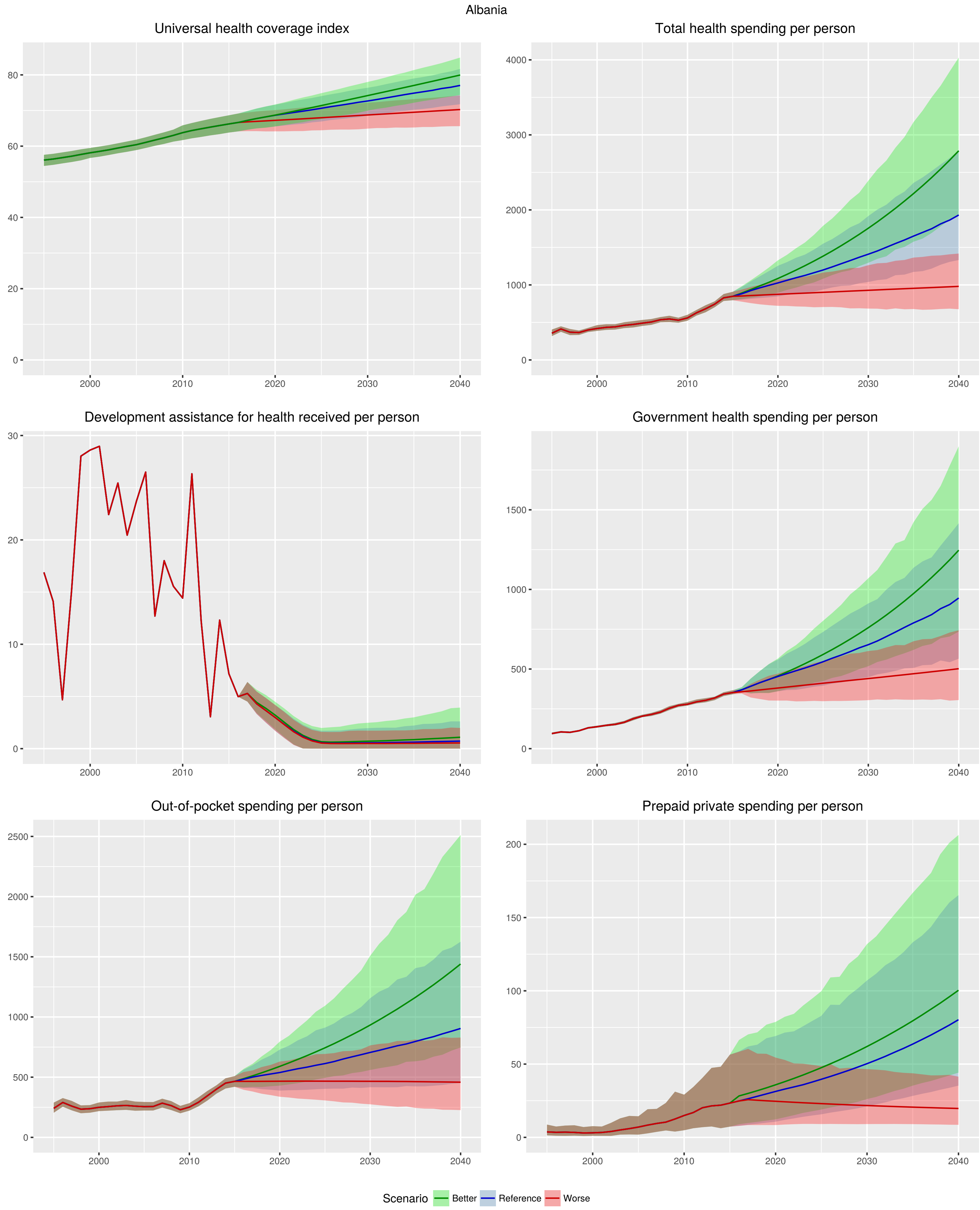

Albania
Universal health coverage index
Total health spending per person
Development assistance for health received per person
Government health spending per person
Out-of-pocket spending per person
Prepaid private spending per person
0
20
40
60
80
1000
2000
3000
4000
10
30
500
1500
2500
50
100
150
200
2000
2010
2020
2030
2040
Scenario
Better
Reference
Worse

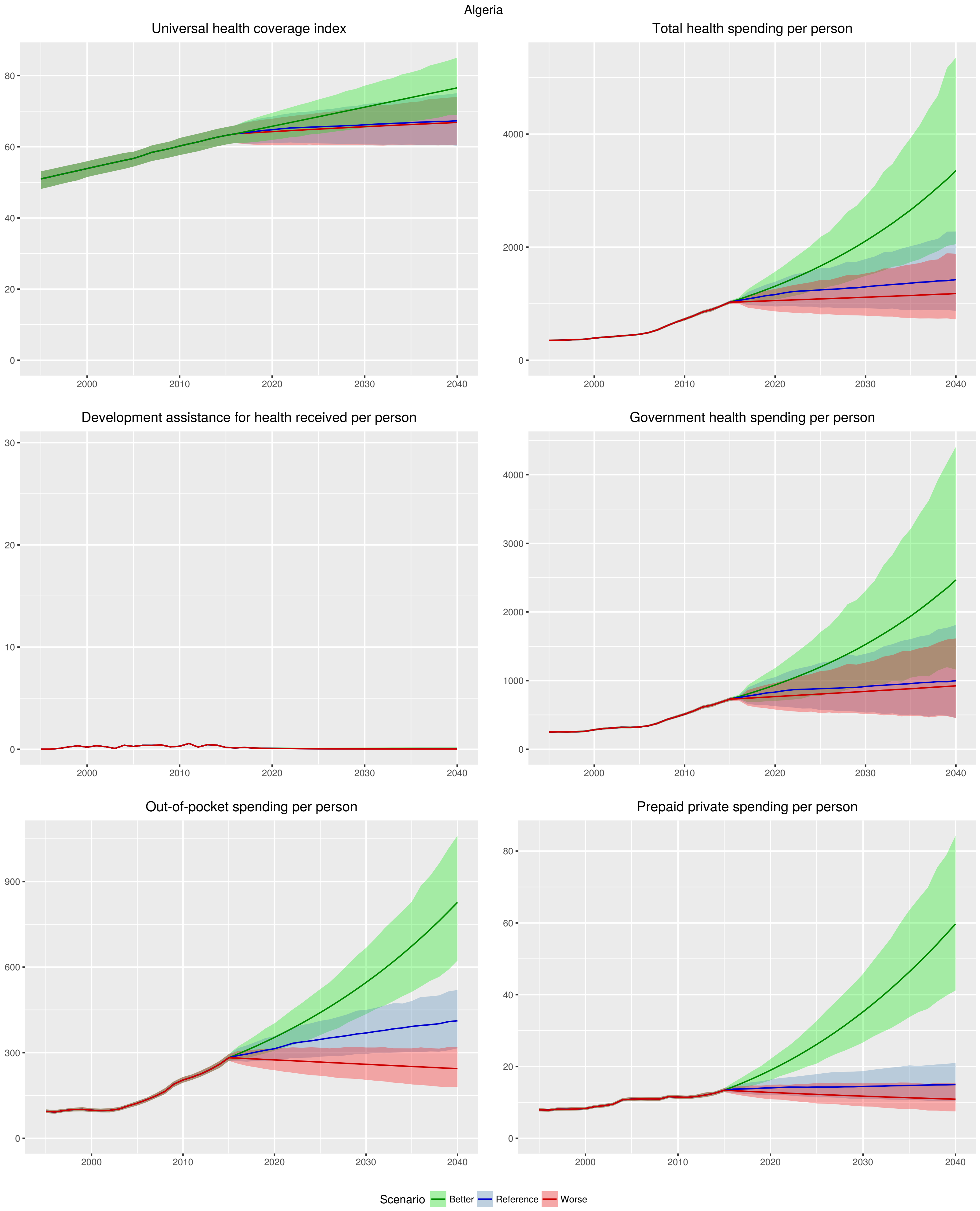

Algeria
Universal health coverage index
Total health spending per person
Development assistance for health received per person
Government health spending per person
Out-of-pocket spending per person
Prepaid private spending per person
Scenario
Better
Reference
Worse

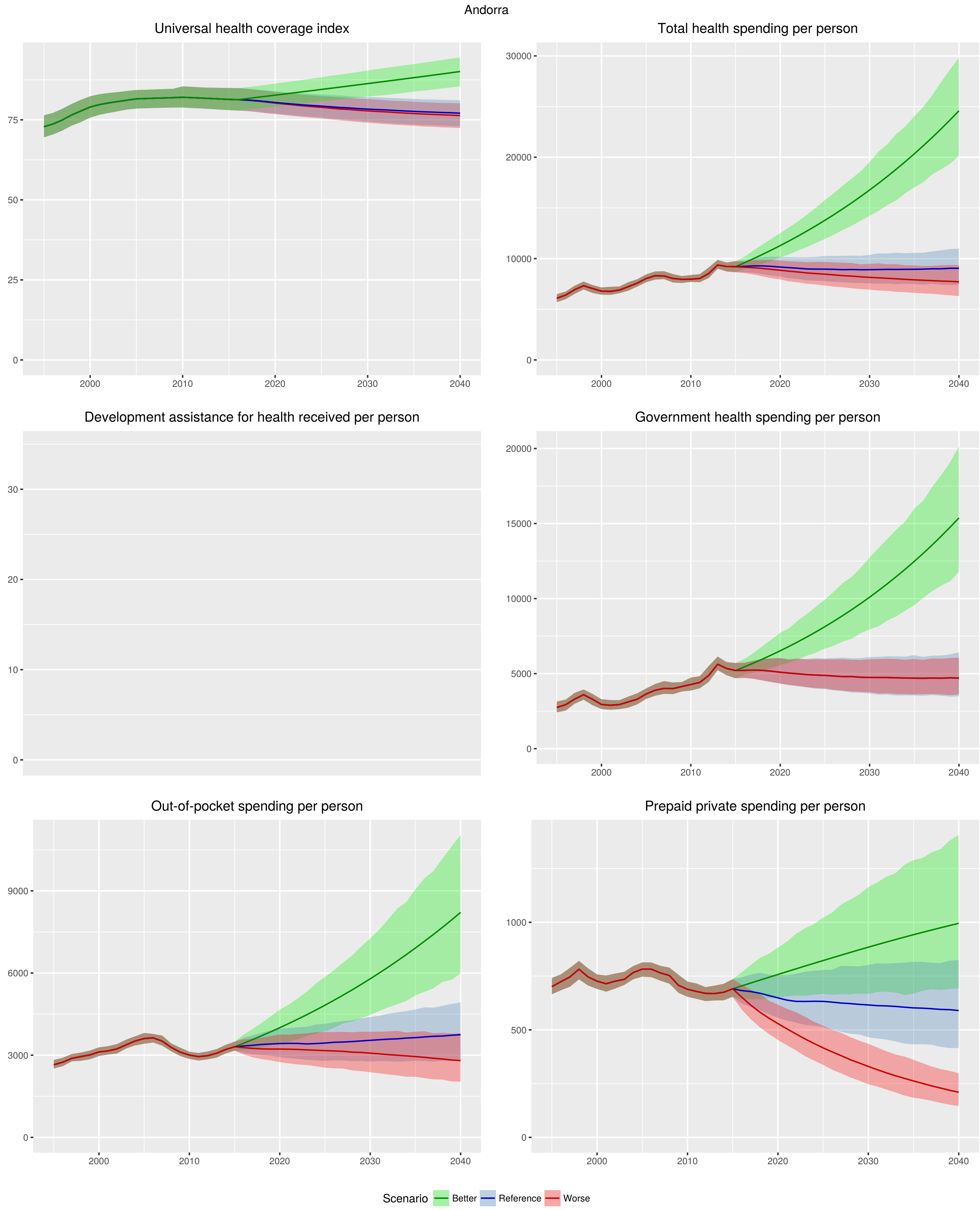

Andorra
Universal health coverage index
Total health spending per person
Development assistance for health received per person
Government health spending per person
Out-of-pocket spending per person
Prepaid private spending per person
0
25
50
75
0
10000
20000
30000
0
10
20
30
0
5000
10000
15000
20000
0
3000
6000
9000
0
500
1000
2000
2010
2020
2030
2040
Scenario
Better
Reference
Worse

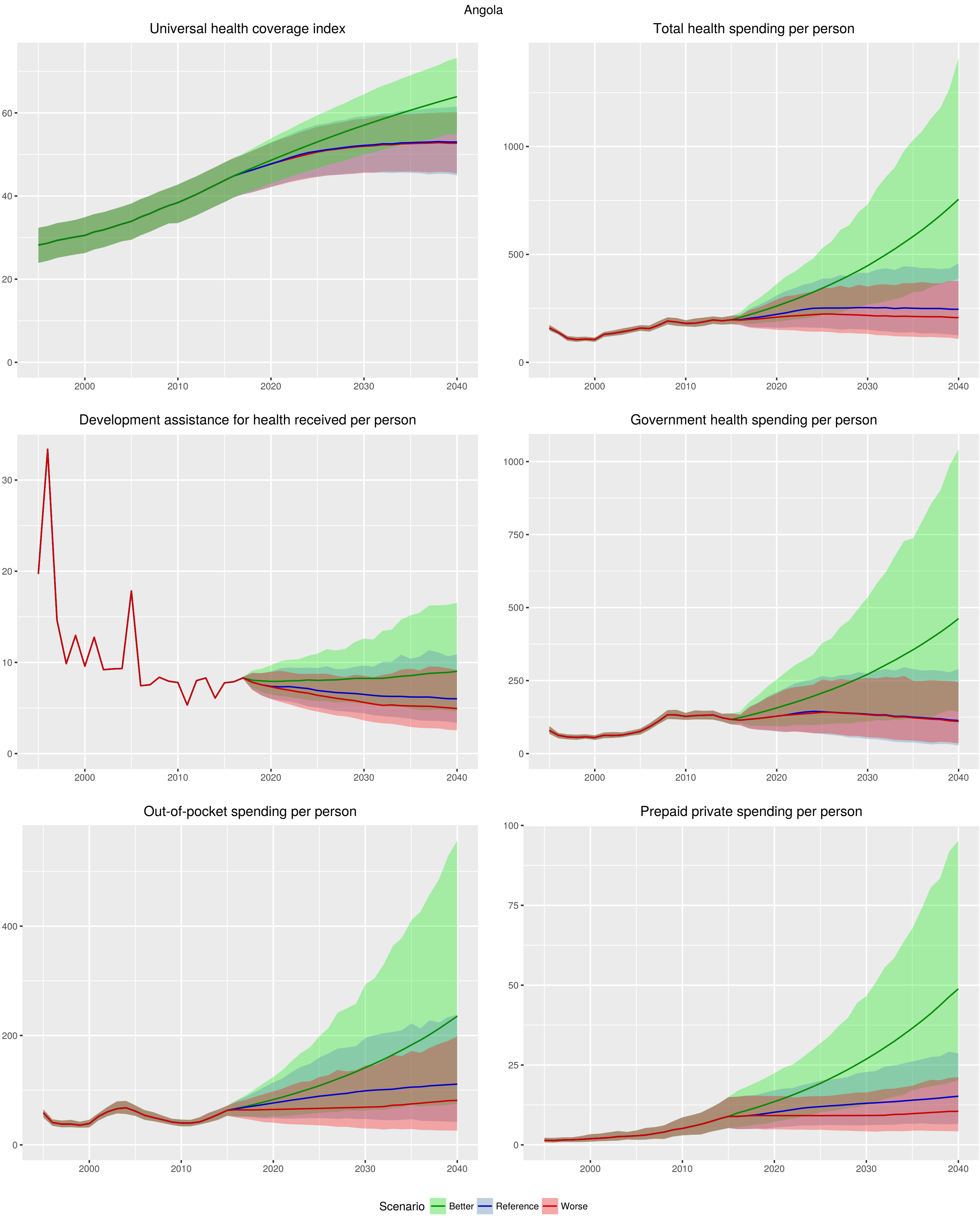

Angola
Universal health coverage index
Total health spending per person
Development assistance for health received per person
Government health spending per person
Out-of-pocket spending per person
Prepaid private spending per person
Scenario
Better
Reference
Worse

Antigua and Barbuda

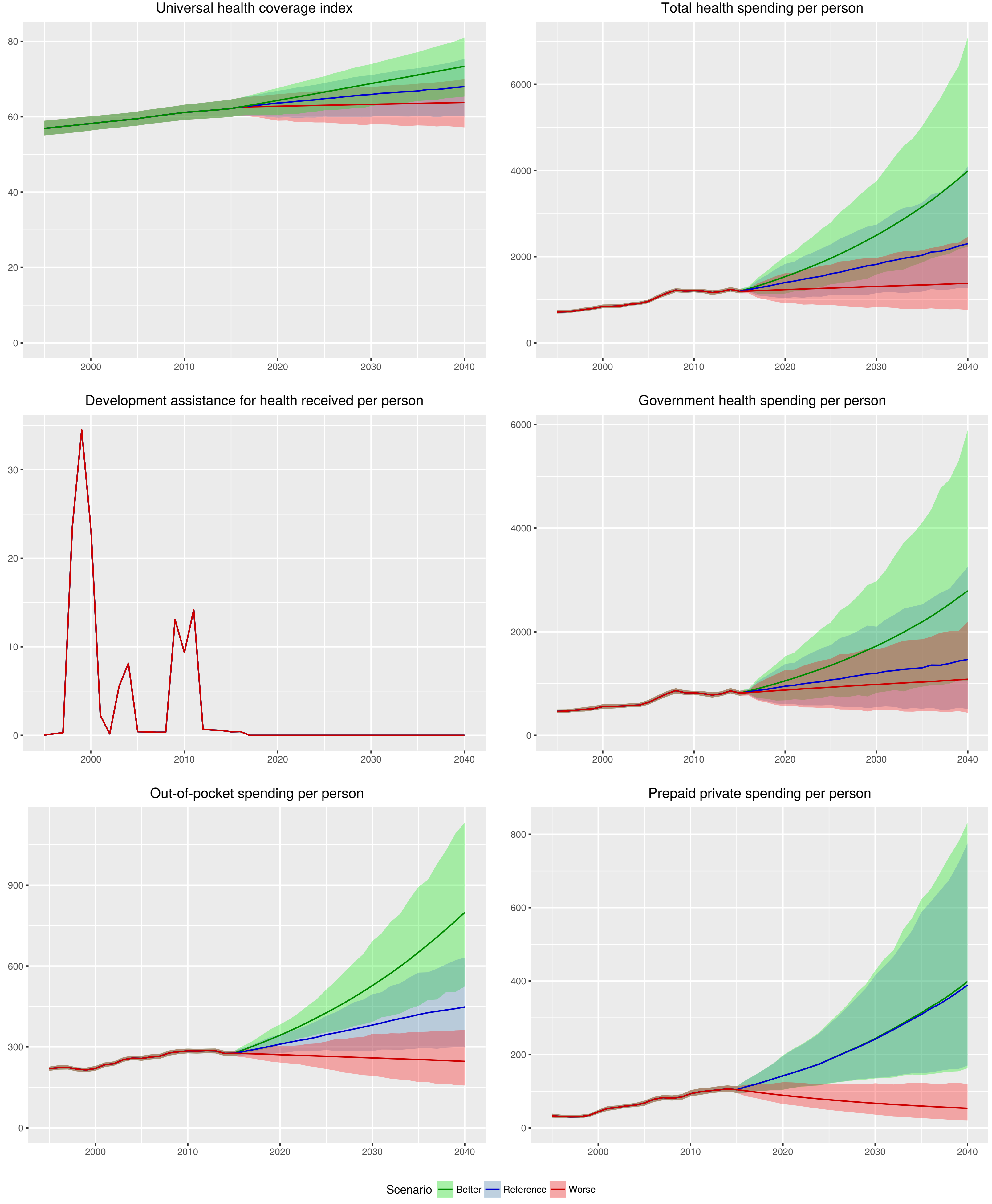

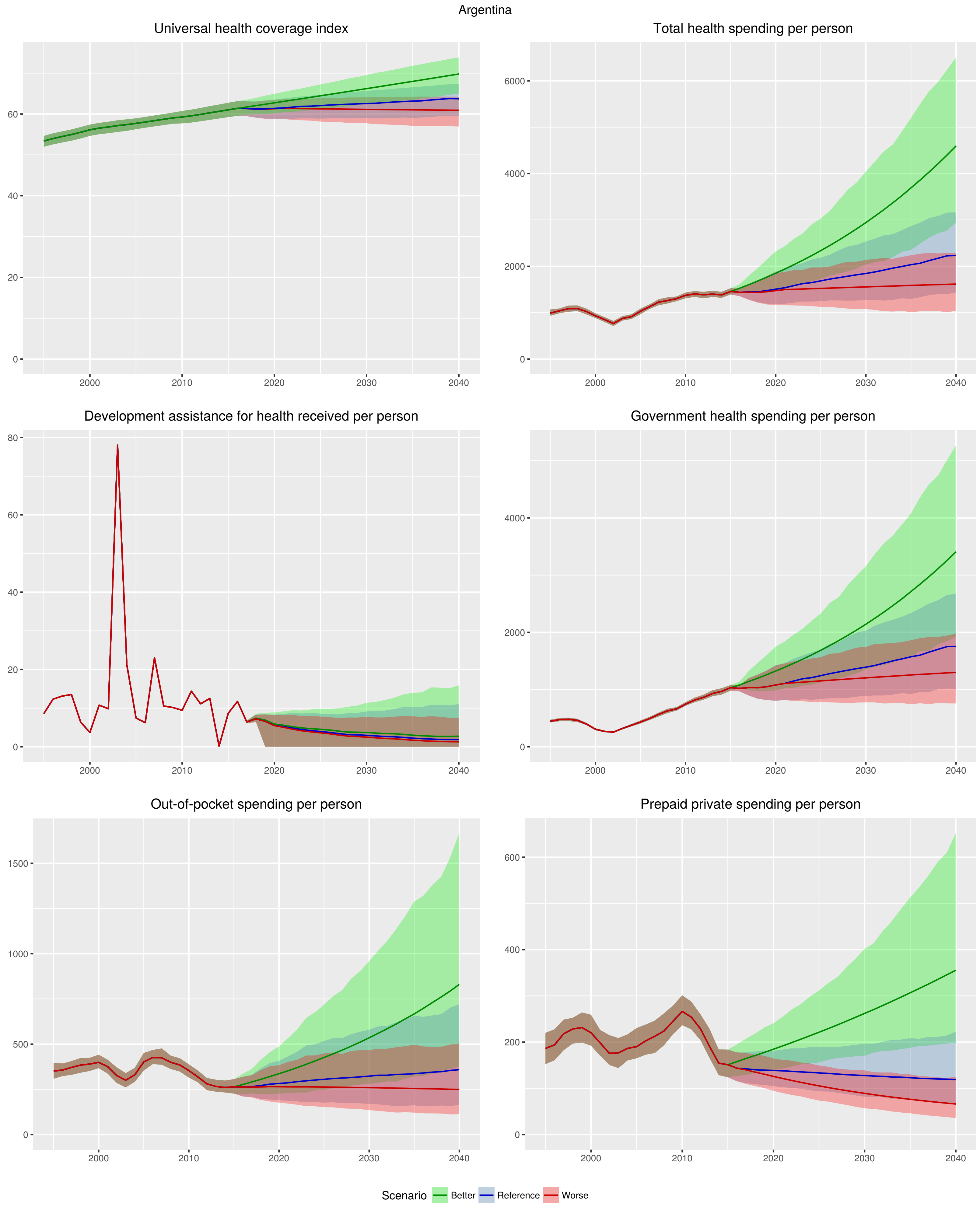
Argentina
Universal health coverage index
Total health spending per person
Development assistance for health received per person
Government health spending per person
Out-of-pocket spending per person
Prepaid private spending per person
0
20
40
60
2000
2010
2020
2030
2040
2000
4000
6000
80
1500
1000
500
200
400
600
Scenario
Better
Reference
Worse

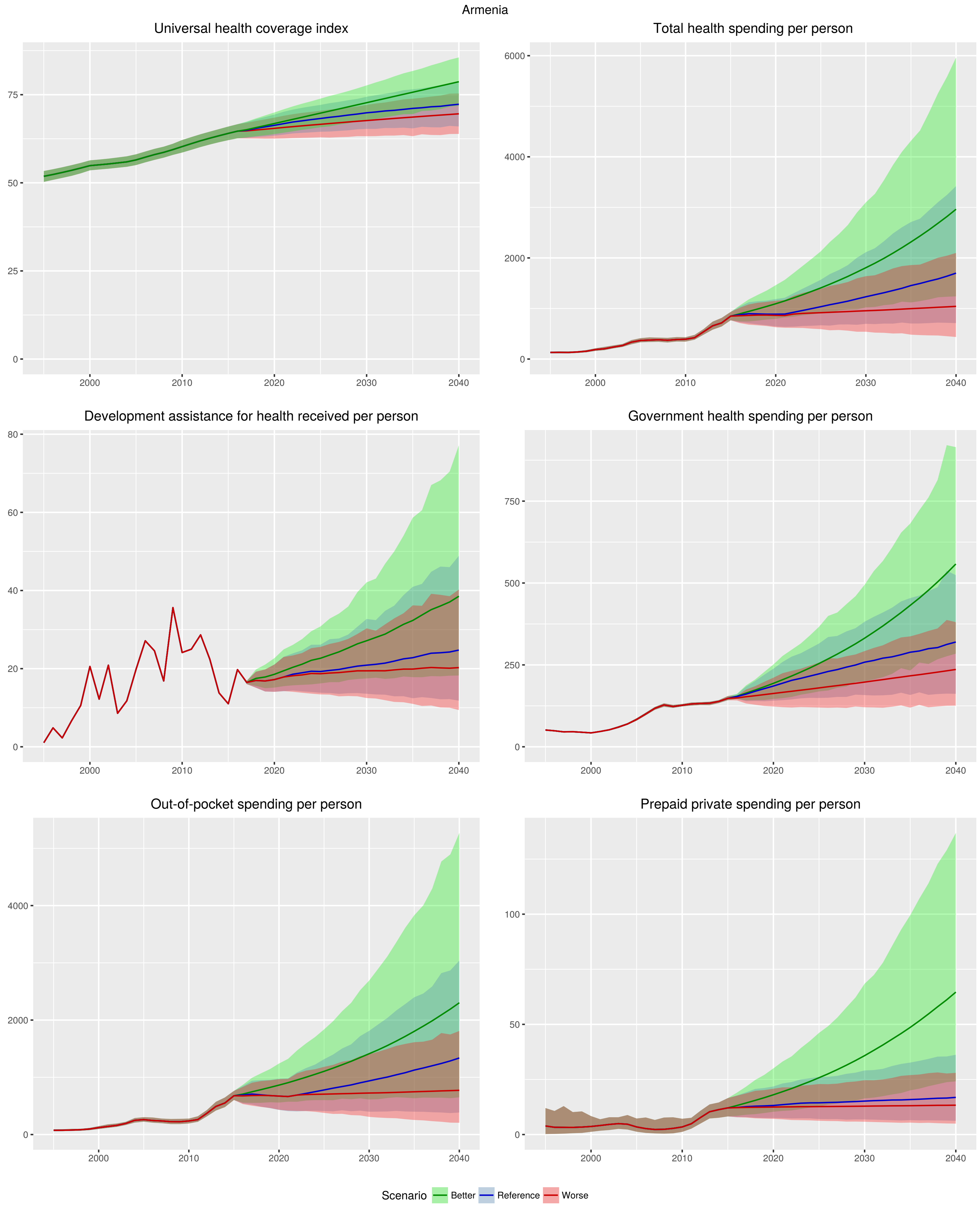

Armenia
Universal health coverage index
Total health spending per person
Development assistance for health received per person
Government health spending per person
Out-of-pocket spending per person
Prepaid private spending per person
0
25
50
75
2000
4000
6000
20
40
60
80
250
500
750
100
2000
2010
2020
2030
2040
Scenario
Better
Reference
Worse

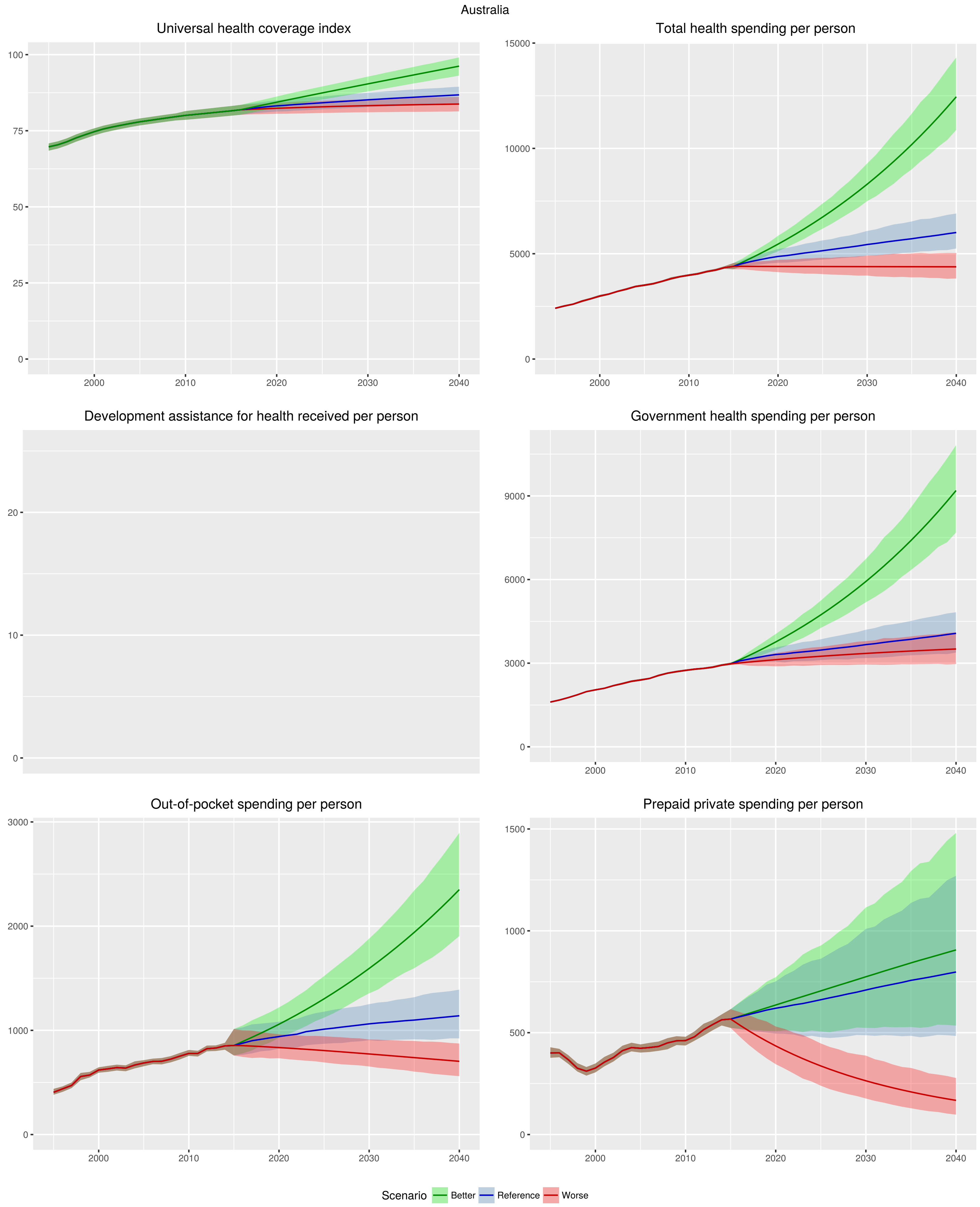

Australia
Universal health coverage index
100
75
50
25
0
2000
2010
2020
2030
2040
Total health spending per person
15000
10000
5000
0
2000
2010
2020
2030
2040
Development assistance for health received per person
20
10
0
Government health spending per person
9000
6000
3000
0
2000
2010
2020
2030
2040
Out-of-pocket spending per person
3000
2000
1000
0
2000
2010
2020
2030
2040
Prepaid private spending per person
1500
1000
500
0
2000
2010
2020
2030
2040
Scenario
Better
Reference
Worse

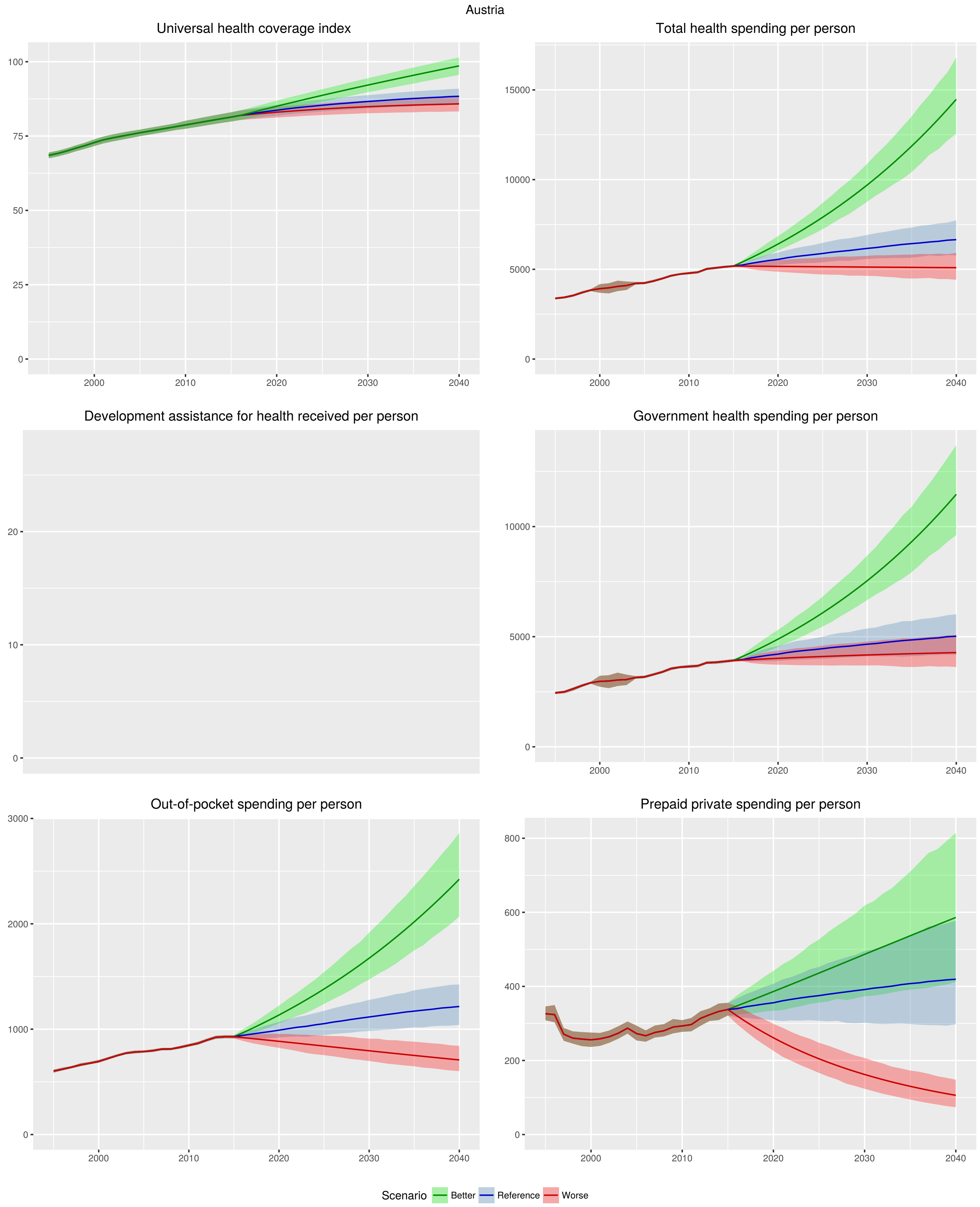
Austria
Universal health coverage index
Total health spending per person
Development assistance for health received per person
Government health spending per person
Out-of-pocket spending per person
Prepaid private spending per person
0
25
50
75
100
5000
10000
15000
10
20
3000
2000
1000
200
400
600
800
2000
2010
2020
2030
2040
Scenario
Better
Reference
Worse

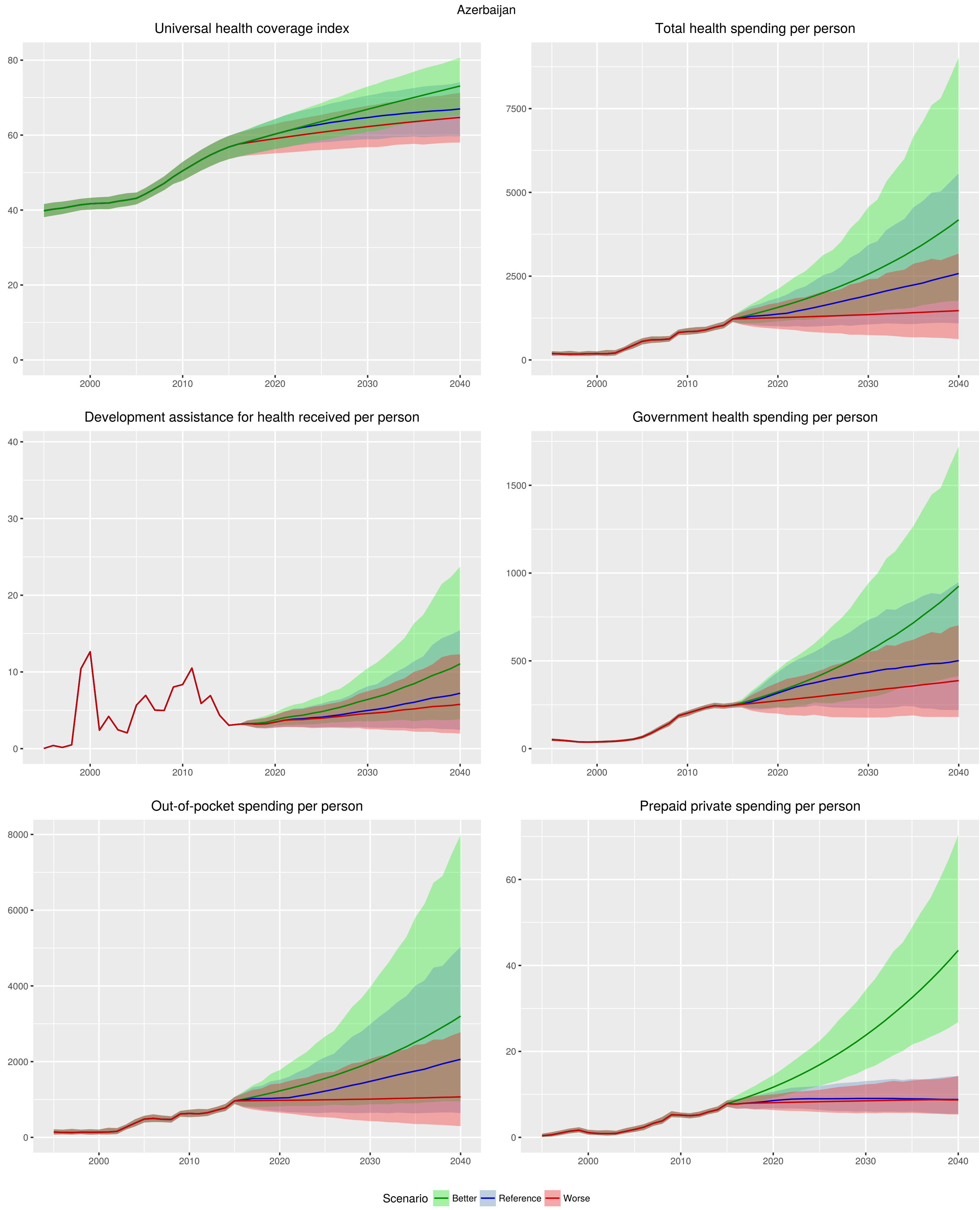

Azerbaijan
Universal health coverage index
Total health spending per person
Development assistance for health received per person
Government health spending per person
Out-of-pocket spending per person
Prepaid private spending per person
Scenario
Better
Reference
Worse

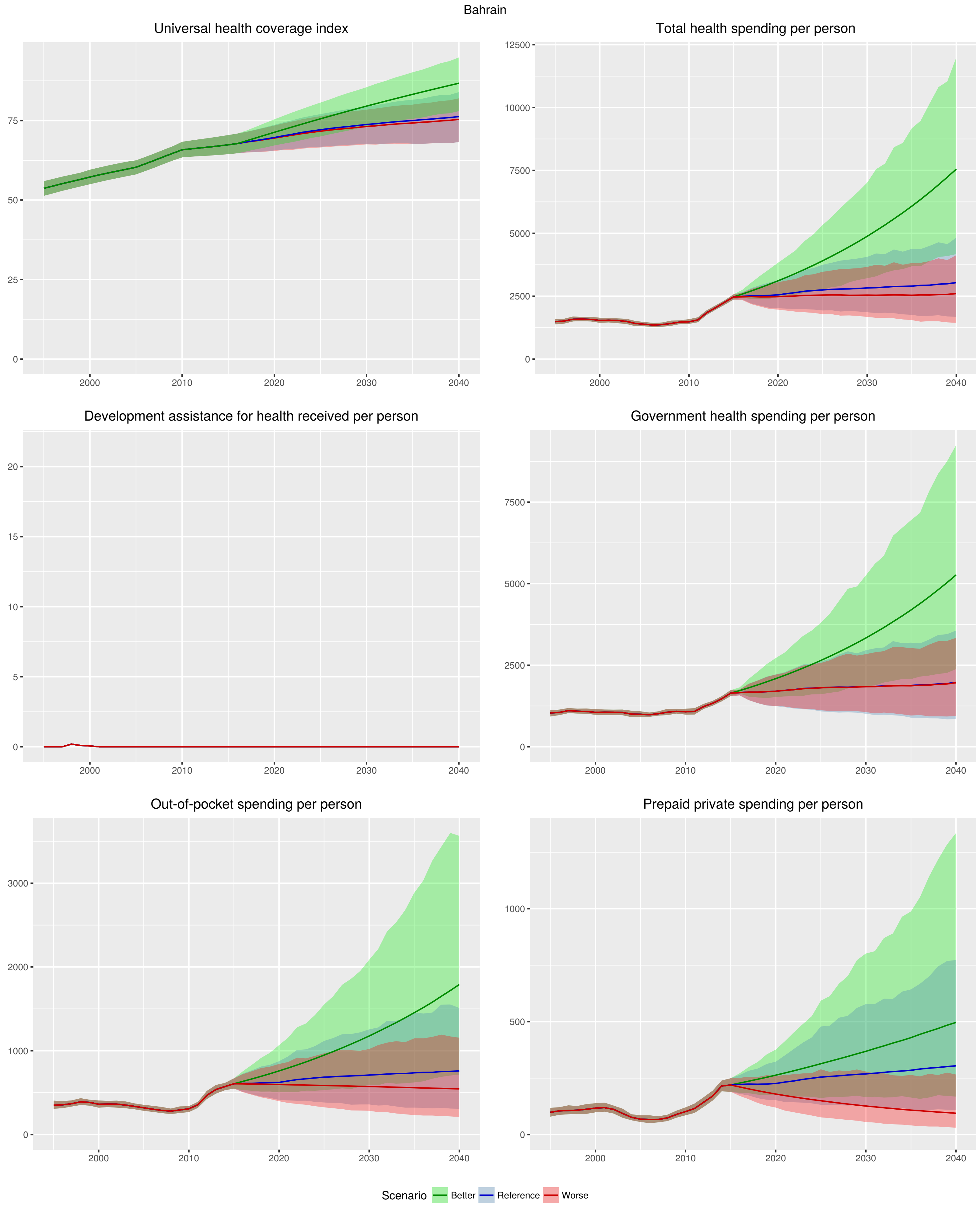
Bahrain
Universal health coverage index
0
25
50
75
2000
2010
2020
2030
2040
Total health spending per person
0
2500
5000
7500
10000
12500
2000
2010
2020
2030
2040
Development assistance for health received per person
0
5
10
15
20
2000
2010
2020
2030
2040
Government health spending per person
0
2500
5000
7500
2000
2010
2020
2030
2040
Out-of-pocket spending per person
0
1000
2000
3000
2000
2010
2020
2030
2040
Prepaid private spending per person
0
500
1000
2000
2010
2020
2030
2040
Scenario
Better
Reference
Worse

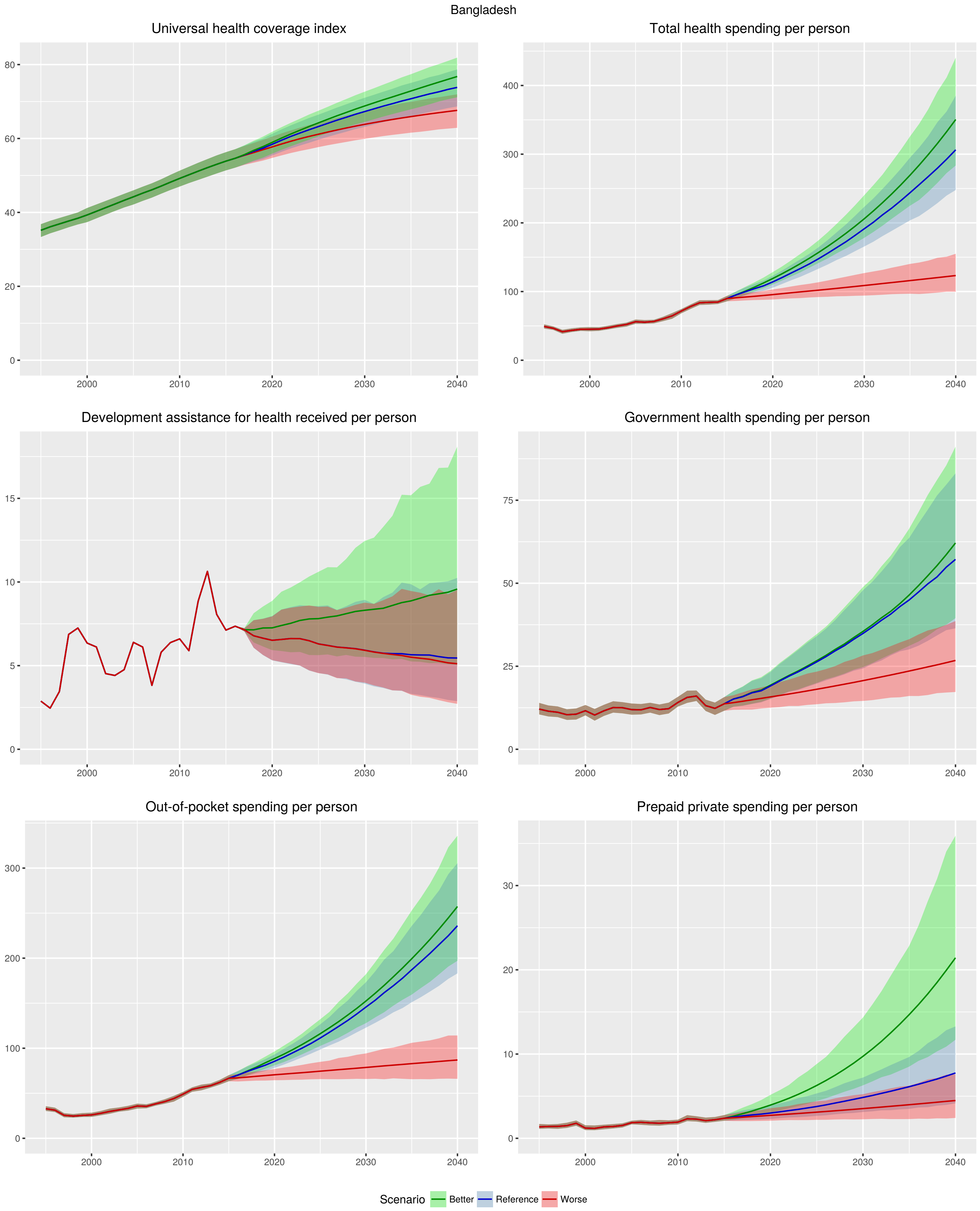
Bangladesh
Universal health coverage index
Total health spending per person
Development assistance for health received per person
Government health spending per person
Out-of-pocket spending per person
Prepaid private spending per person
2000
2010
2020
2030
2040
Scenario
Better
Reference
Worse

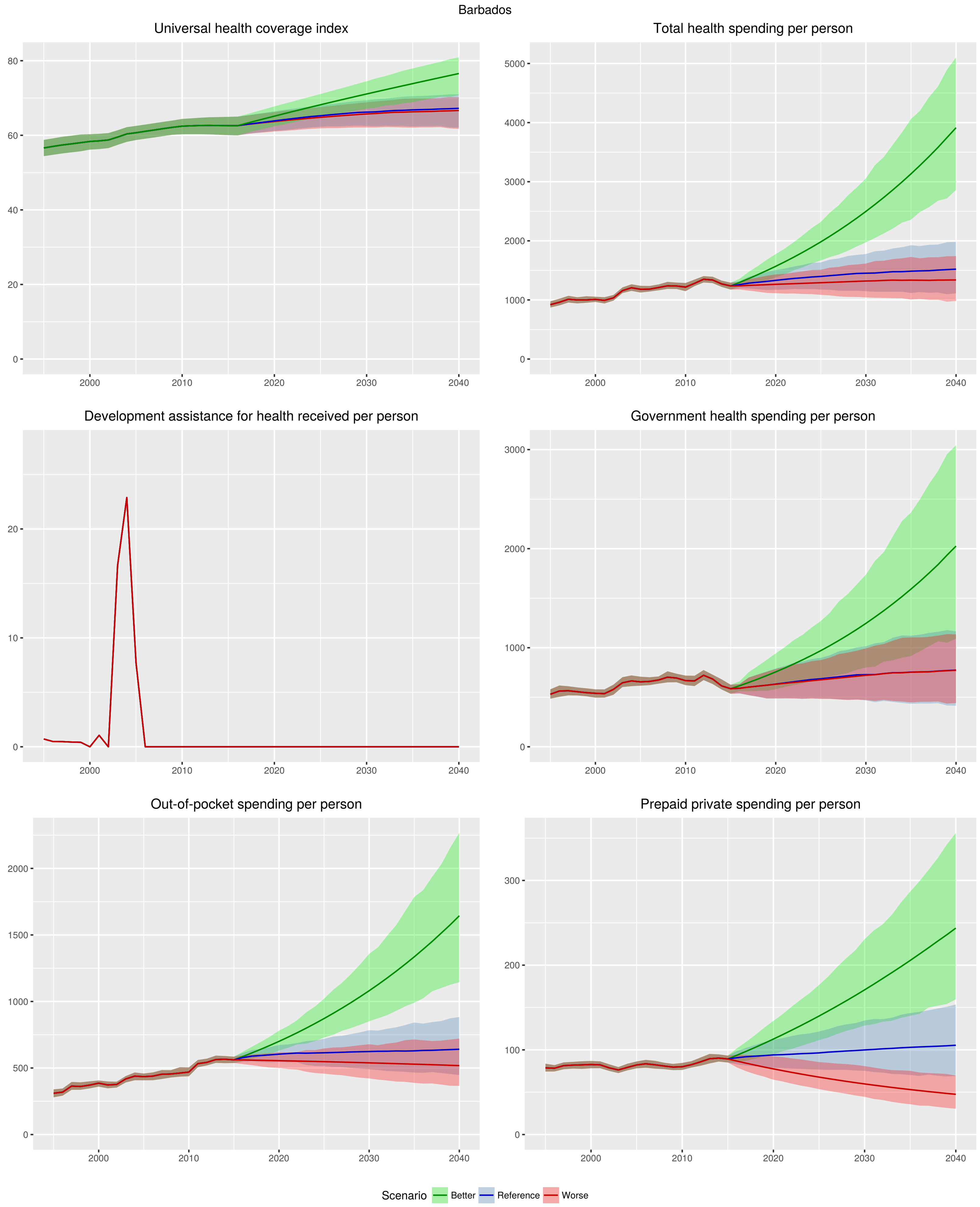

Barbados
Universal health coverage index
Total health spending per person
Development assistance for health received per person
Government health spending per person
Out-of-pocket spending per person
Prepaid private spending per person
0
20
40
60
80
1000
2000
3000
4000
5000
10
1500
100
200
300
2000
2010
2020
2030
2040
Scenario
Better
Reference
Worse

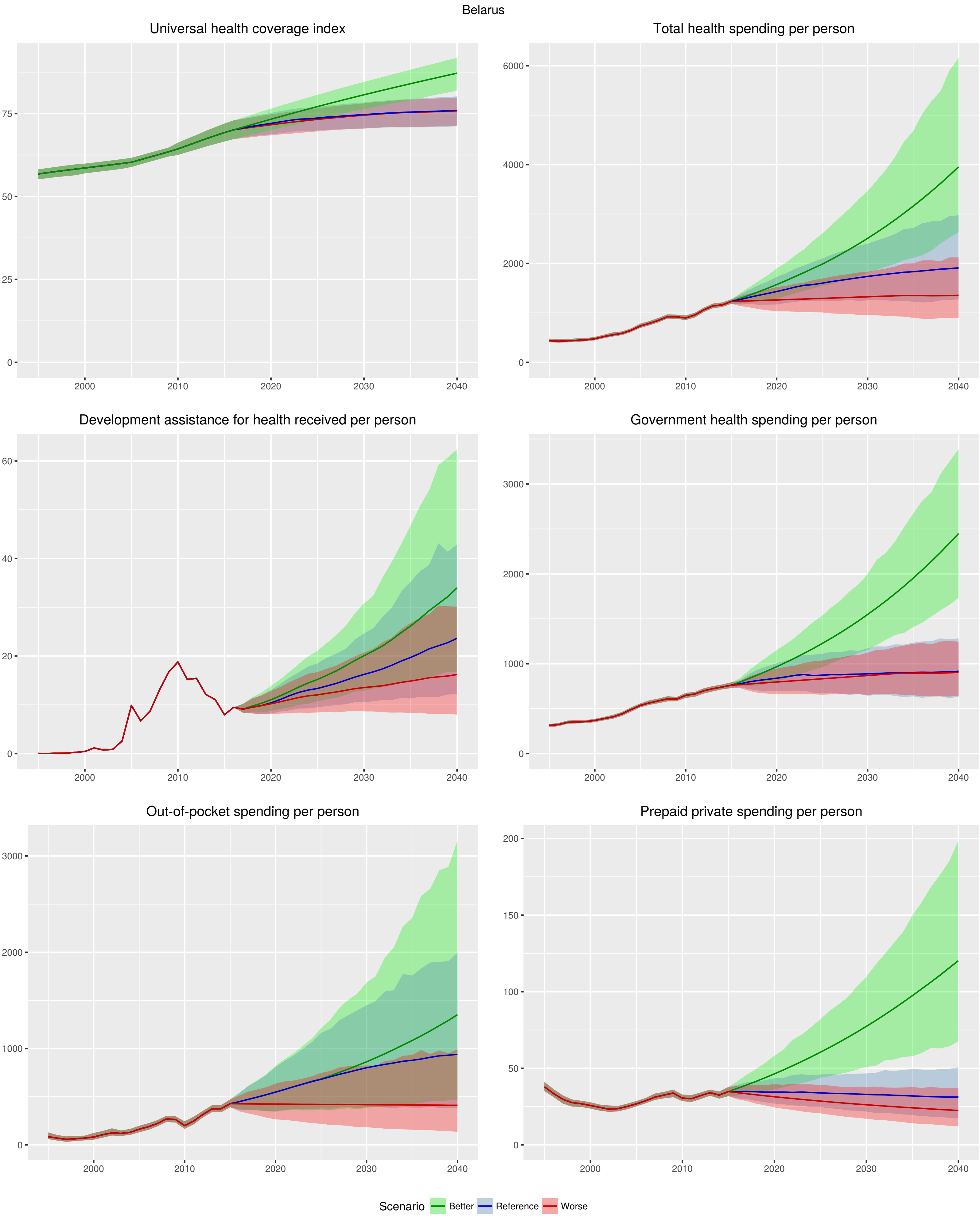

Belarus
Universal health coverage index
Total health spending per person
Development assistance for health received per person
Government health spending per person
Out-of-pocket spending per person
Prepaid private spending per person
Scenario
Better
Reference
Worse

Belgium

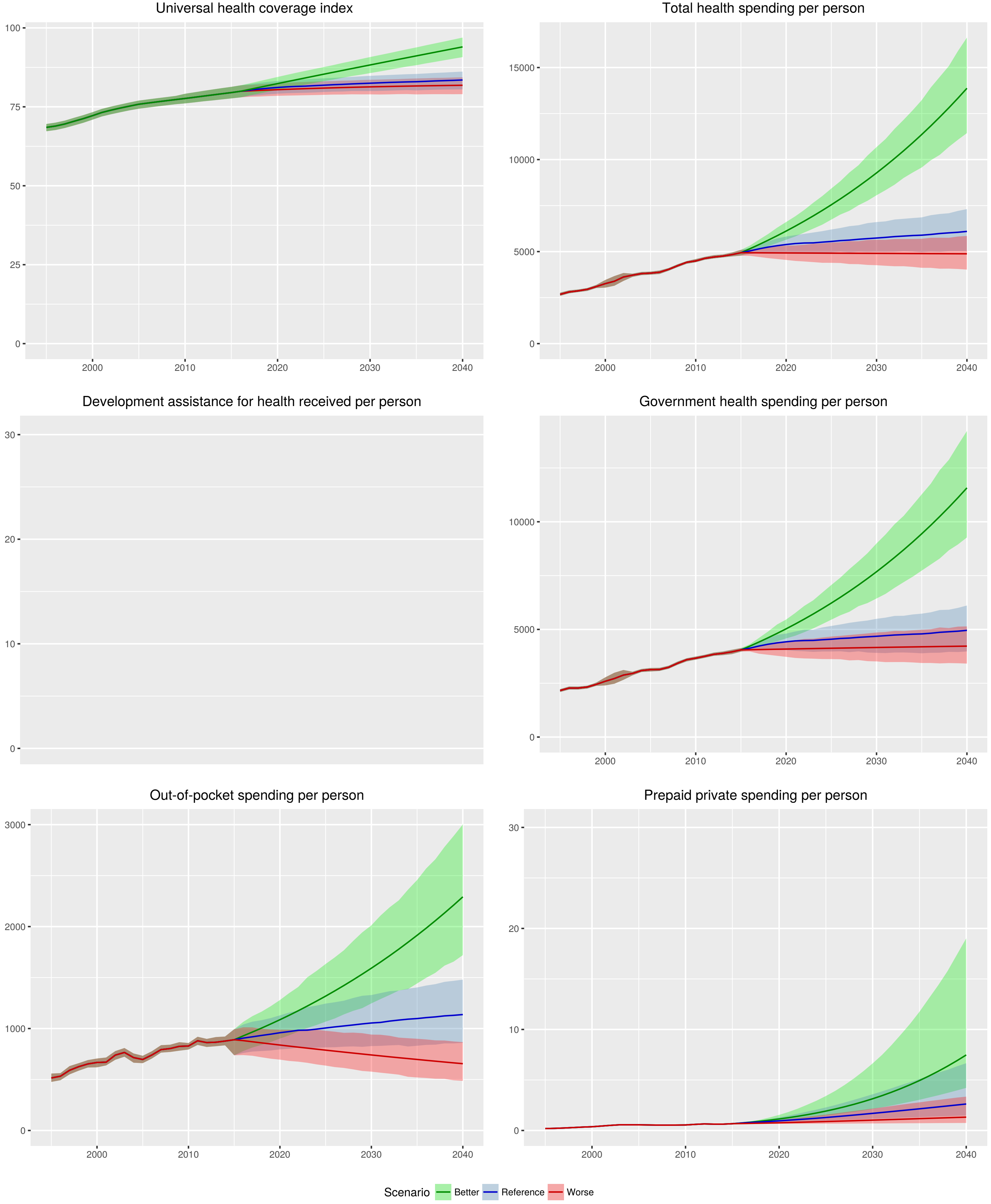
Universal health coverage index
Total health spending per person
Development assistance for health received per person
Government health spending per person
Out-of-pocket spending per person
Prepaid private spending per person
Scenario
Better
Reference
Worse

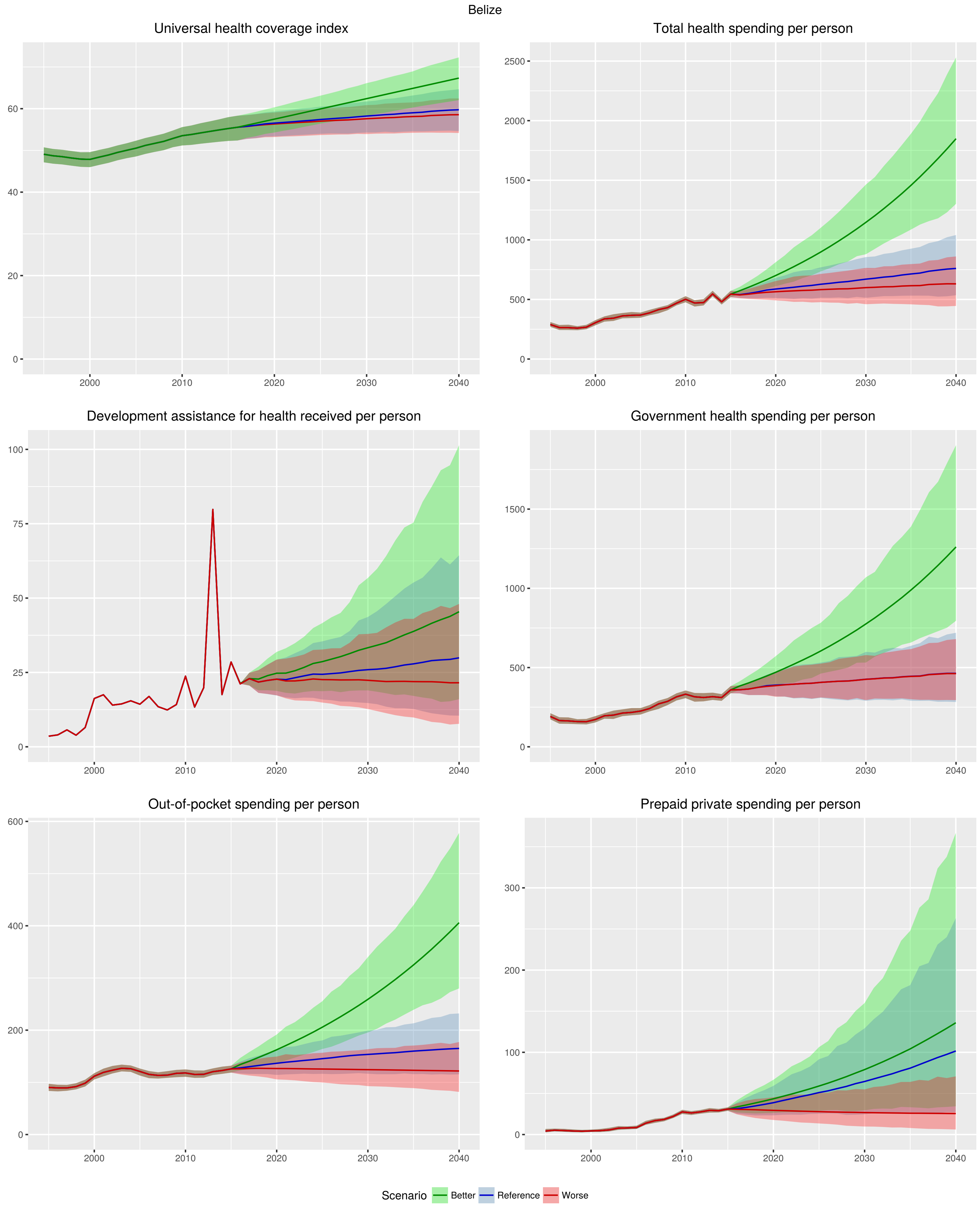
Belize
Universal health coverage index
0
20
40
60
2000
2010
2020
2030
2040
Total health spending per person
0
500
1000
1500
2000
2500
2000
2010
2020
2030
2040
Development assistance for health received per person
0
25
50
75
100
2000
2010
2020
2030
2040
Government health spending per person
0
500
1000
1500
2000
2010
2020
2030
2040
Out-of-pocket spending per person
0
200
400
600
2000
2010
2020
2030
2040
Prepaid private spending per person
0
100
200
300
2000
2010
2020
2030
2040
Scenario
Better
Reference
Worse

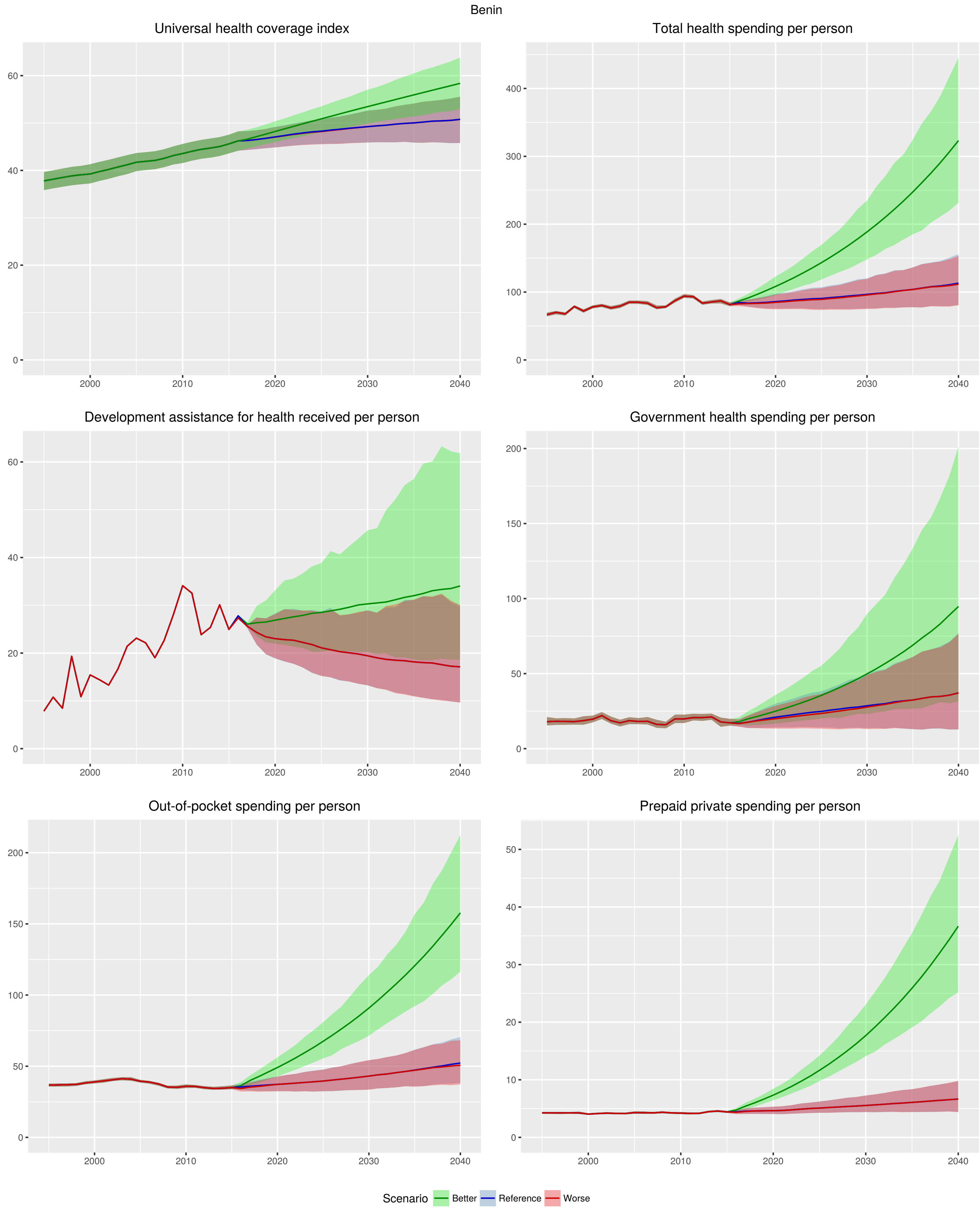

Benin
Universal health coverage index
Total health spending per person
Development assistance for health received per person
Government health spending per person
Out-of-pocket spending per person
Prepaid private spending per person
Scenario
Better
Reference
Worse

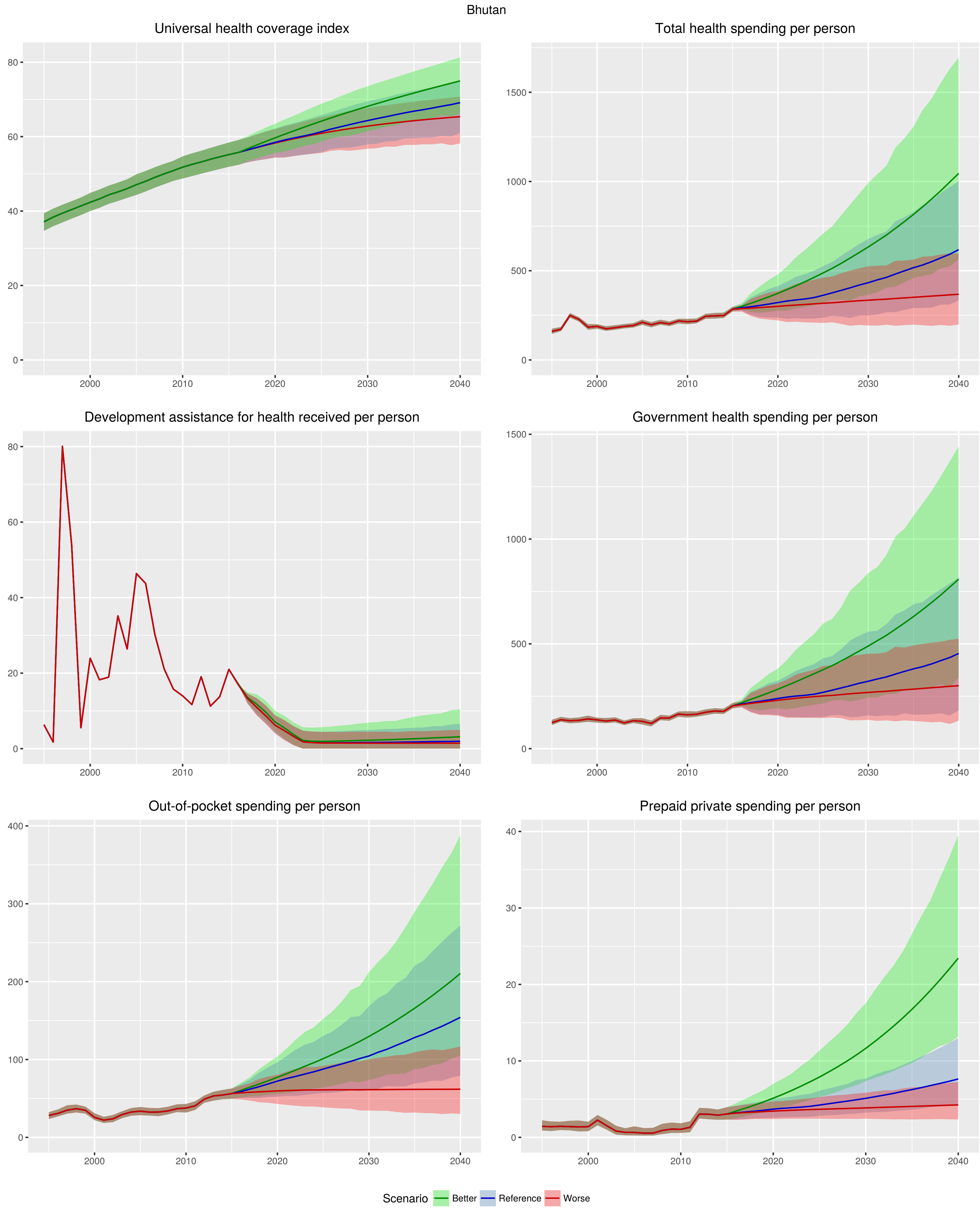
Bhutan
Universal health coverage index
Total health spending per person
Development assistance for health received per person
Government health spending per person
Out-of-pocket spending per person
Prepaid private spending per person
0
20
40
60
80
500
1000
1500
100
200
300
400
10
30
2000
2010
2020
2030
2040
Scenario
Better
Reference
Worse

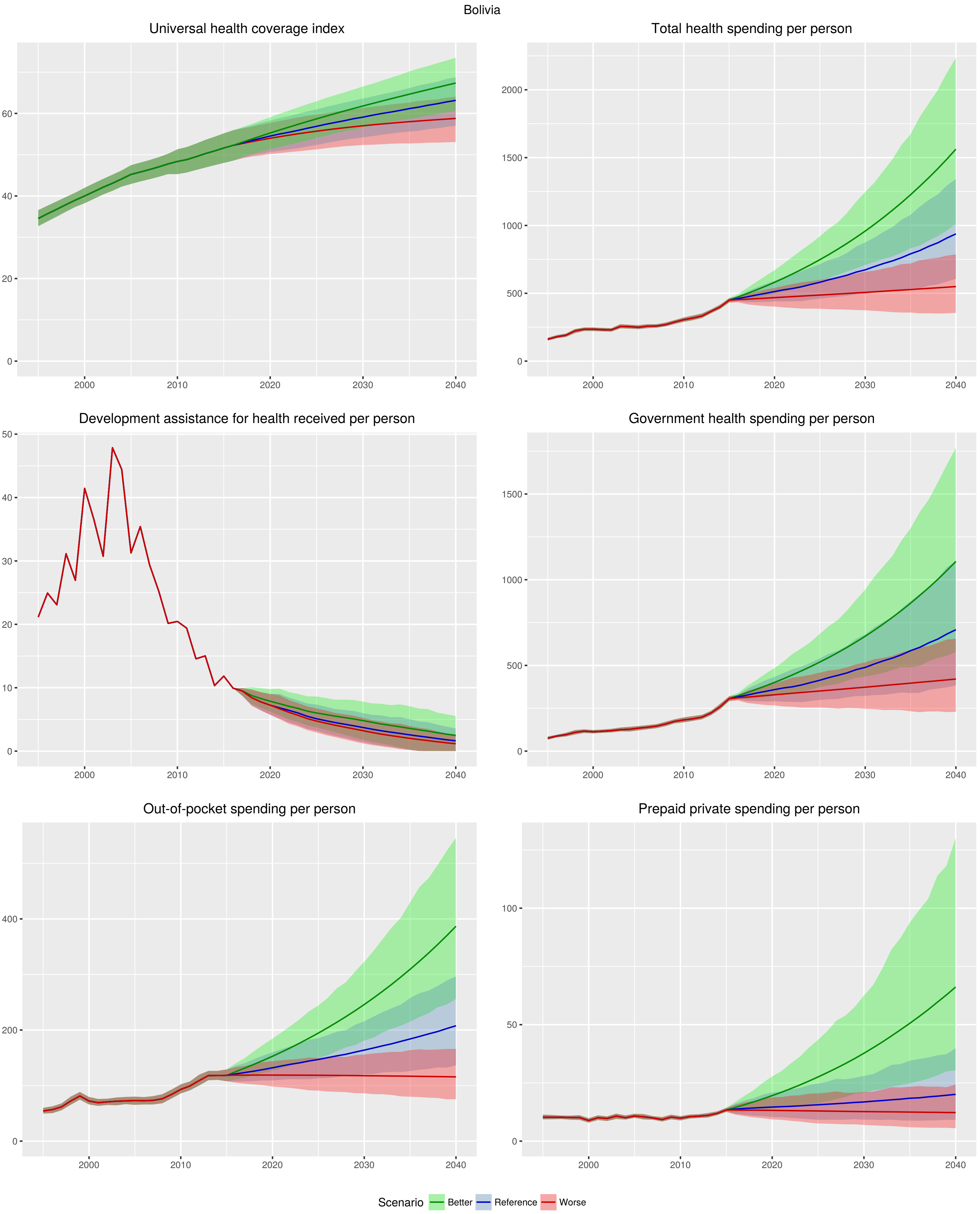
Bolivia
Universal health coverage index
0
20
40
60
2000
2010
2020
2030
2040
Total health spending per person
0
500
1000
1500
2000
2000
2010
2020
2030
2040
Development assistance for health received per person
0
10
20
30
40
50
2000
2010
2020
2030
2040
Government health spending per person
0
500
1000
1500
2000
2010
2020
2030
2040
Out-of-pocket spending per person
0
200
400
2000
2010
2020
2030
2040
Prepaid private spending per person
0
50
100
2000
2010
2020
2030
2040
Scenario
Better
Reference
Worse

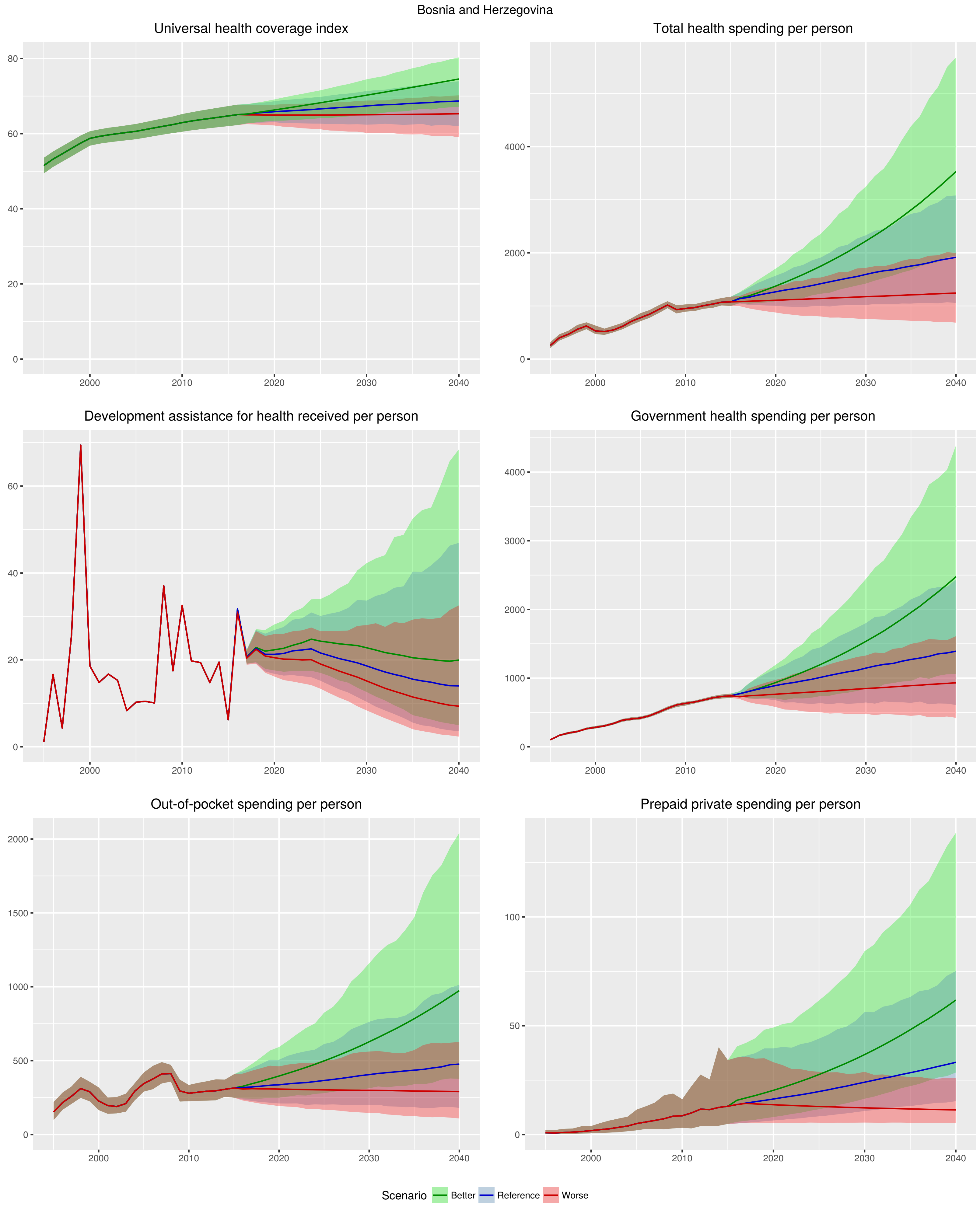

Bosnia and Herzegovina
Universal health coverage index
Total health spending per person
Development assistance for health received per person
Government health spending per person
Out-of-pocket spending per person
Prepaid private spending per person
Scenario
Better
Reference
Worse

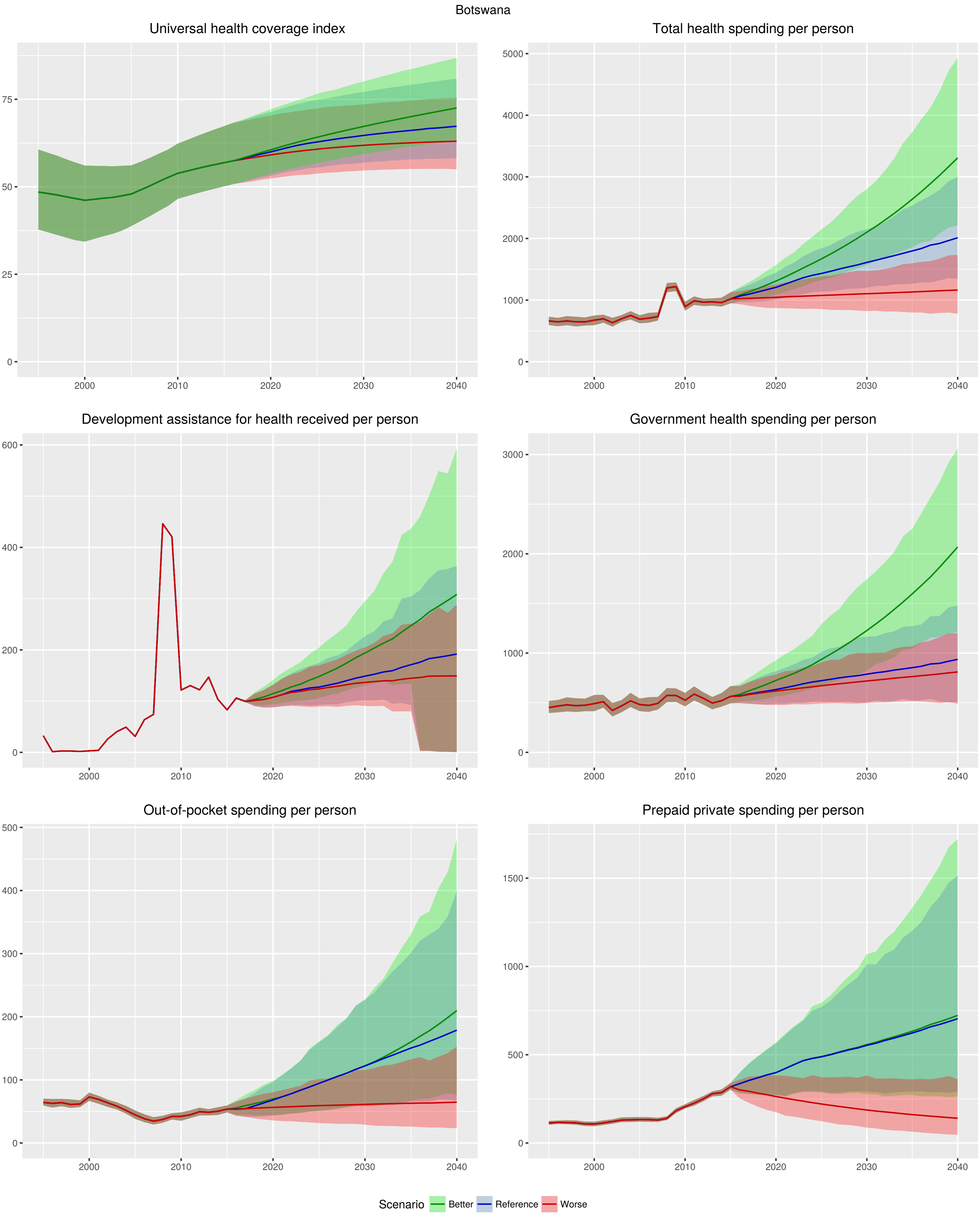
Botswana
Universal health coverage index
Total health spending per person
Development assistance for health received per person
Government health spending per person
Out-of-pocket spending per person
Prepaid private spending per person
2000
2010
2020
2030
2040
Scenario
Better
Reference
Worse

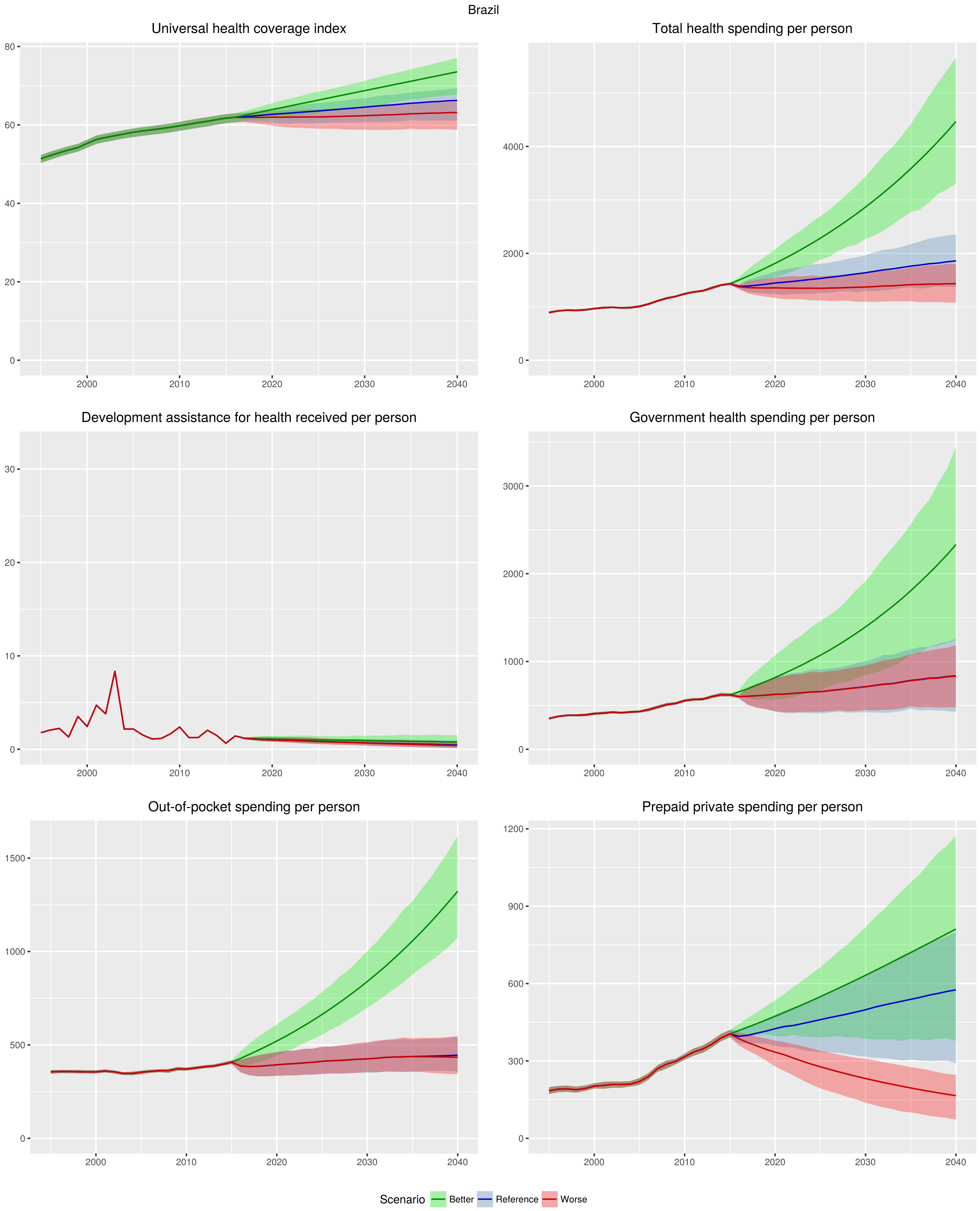

Brazil
Universal health coverage index
Total health spending per person
Development assistance for health received per person
Government health spending per person
Out-of-pocket spending per person
Prepaid private spending per person
Scenario
Better
Reference
Worse

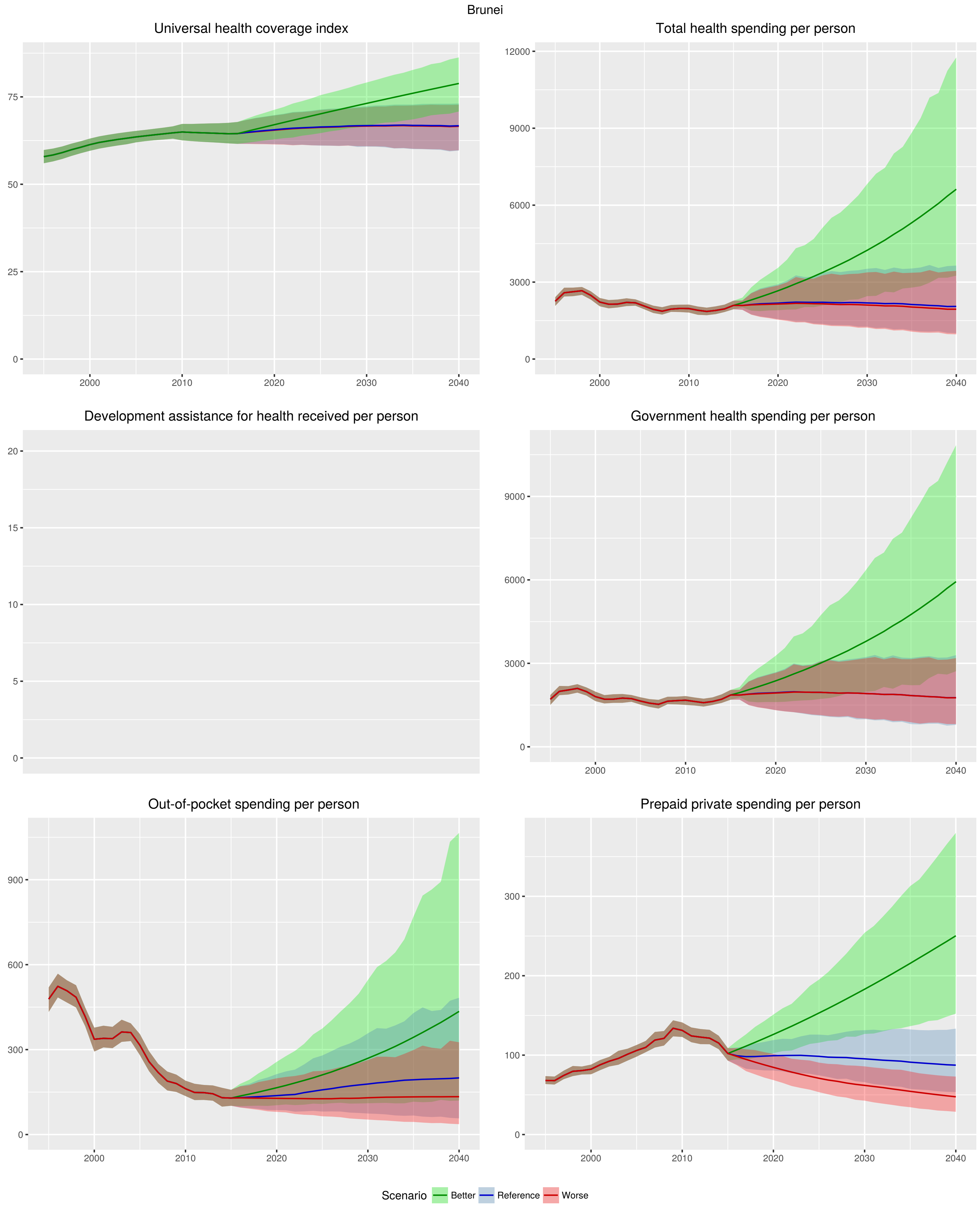
Brunei
Universal health coverage index
Total health spending per person
Development assistance for health received per person
Government health spending per person
Out-of-pocket spending per person
Prepaid private spending per person
Scenario
Better
Reference
Worse

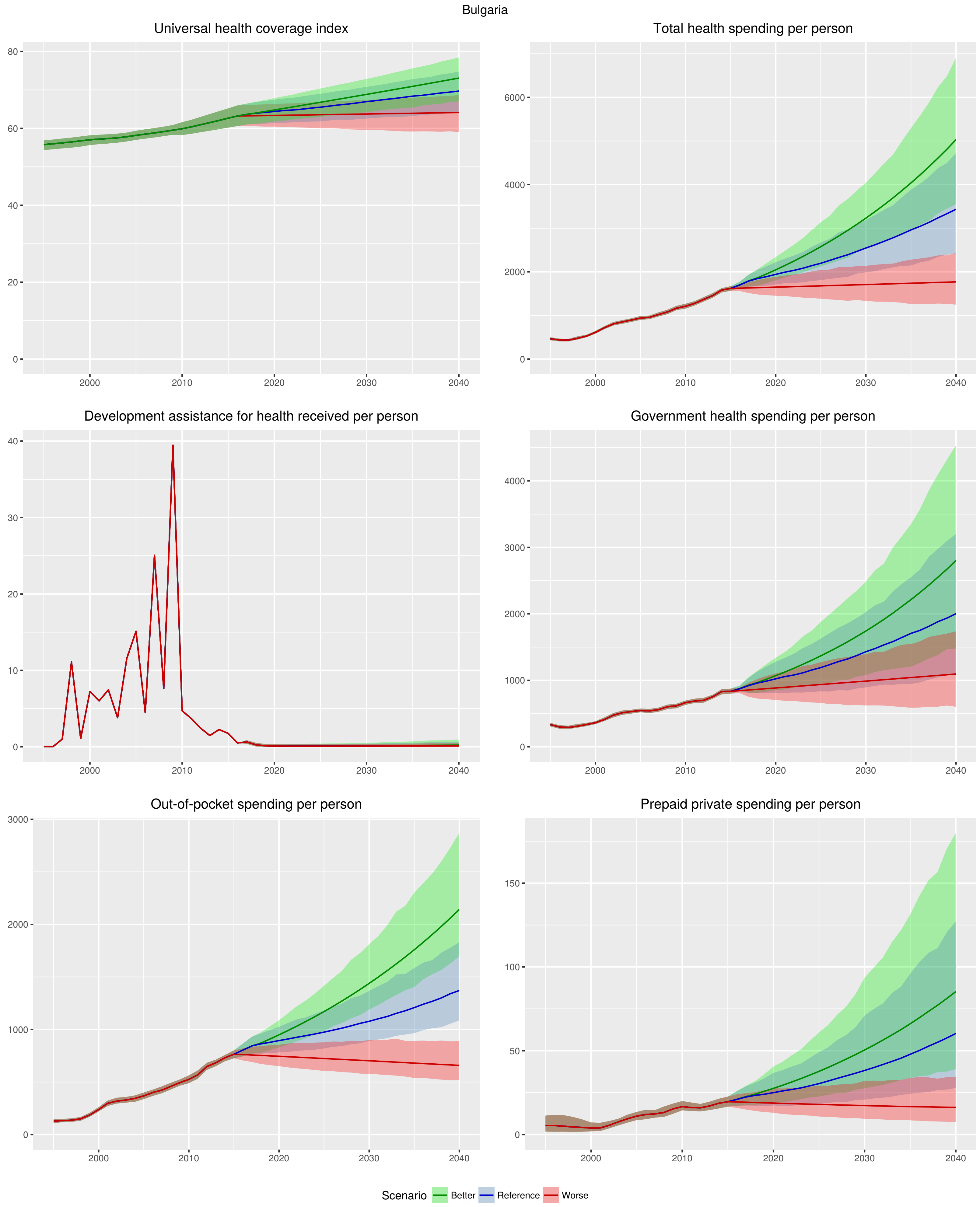
Bulgaria
Universal health coverage index
Total health spending per person
Development assistance for health received per person
Government health spending per person
Out-of-pocket spending per person
Prepaid private spending per person
2000
2010
2020
2030
2040
Scenario
Better
Reference
Worse

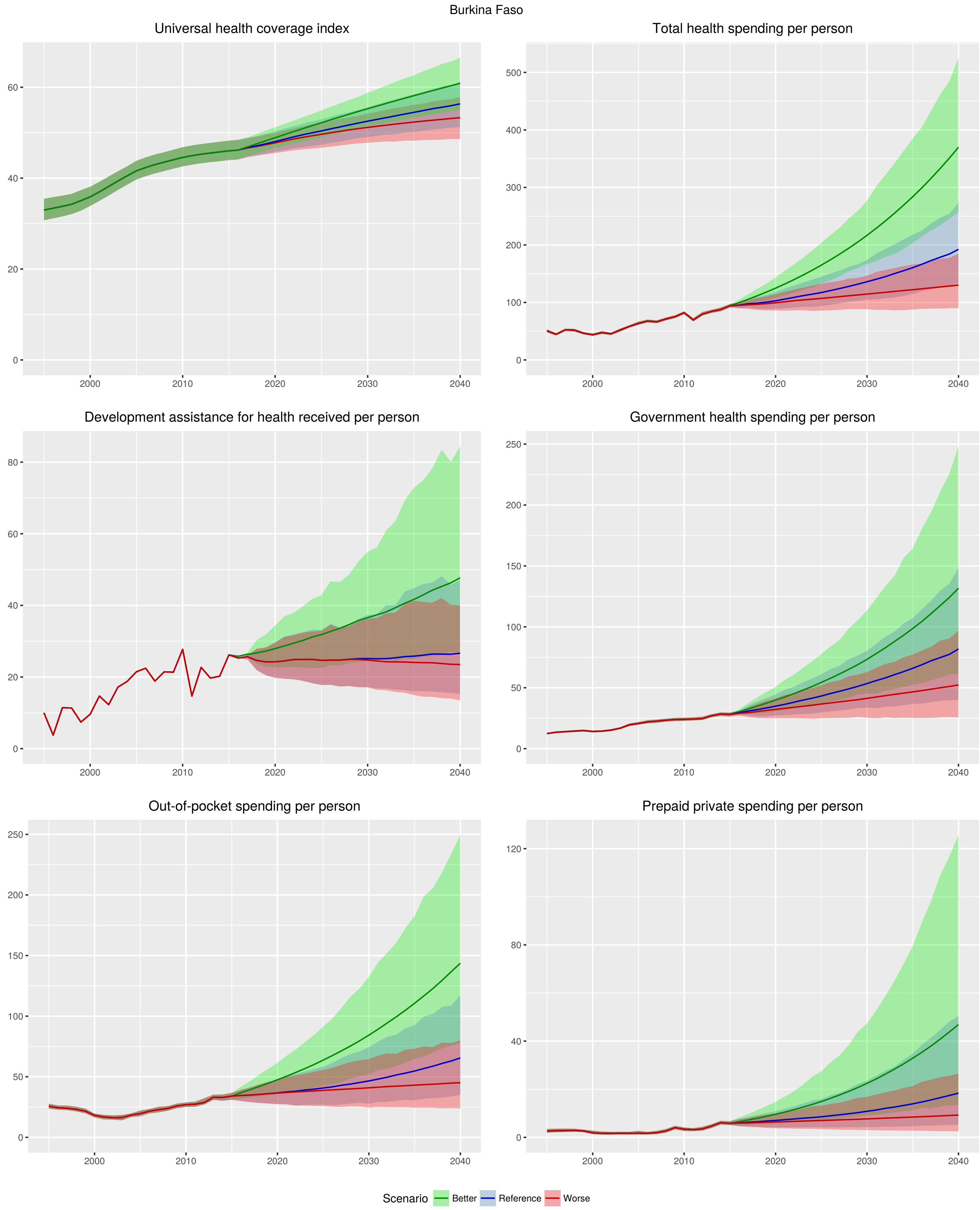
Burkina Faso
Universal health coverage index
Total health spending per person
Development assistance for health received per person
Government health spending per person
Out-of-pocket spending per person
Prepaid private spending per person
0
20
40
60
80
100
120
150
200
250
300
400
500
2000
2010
2020
2030
2040
Scenario
Better
Reference
Worse

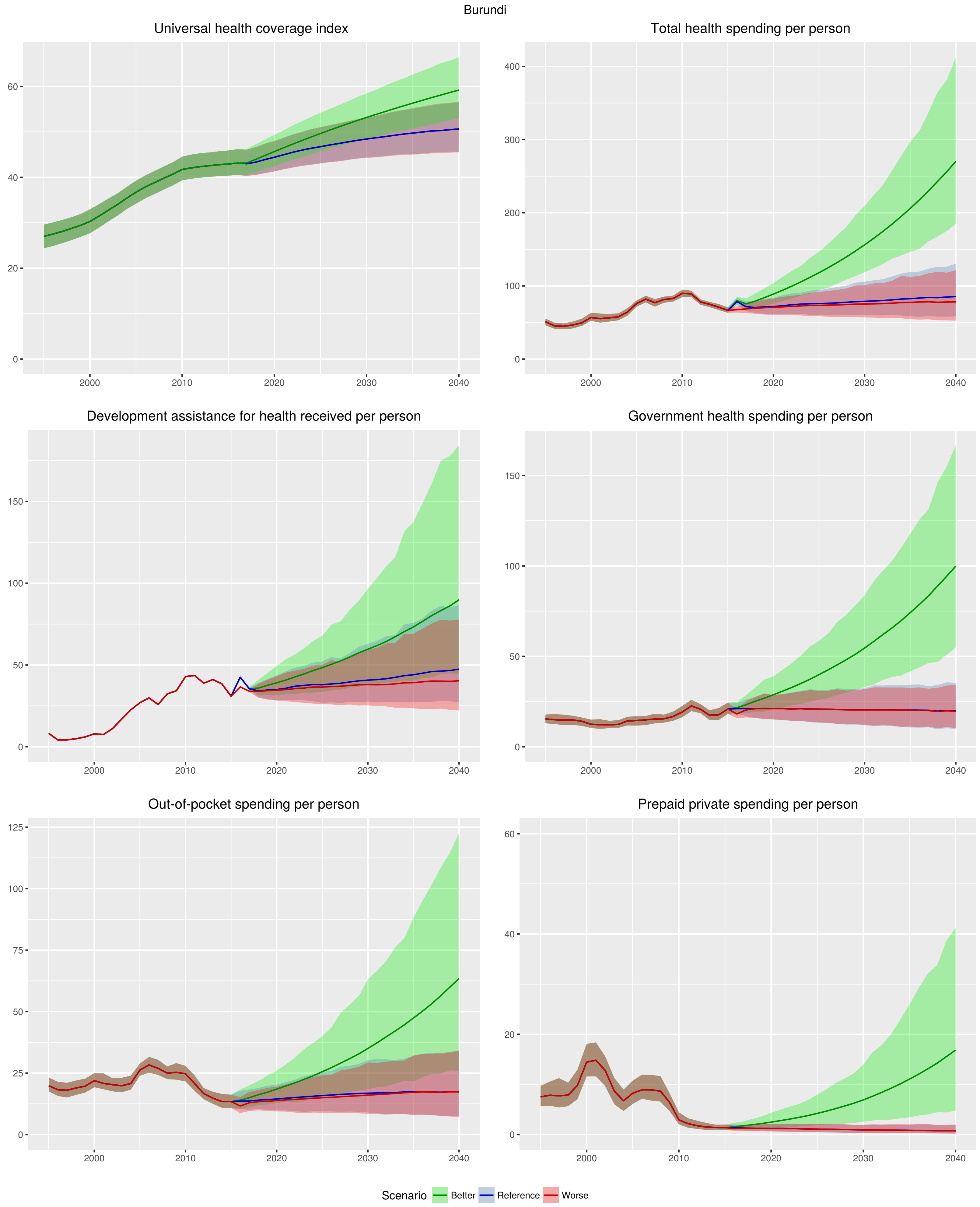
Burundi
Universal health coverage index
0
20
40
60
2000
2010
2020
2030
2040
Total health spending per person
0
100
200
300
400
2000
2010
2020
2030
2040
Development assistance for health received per person
0
50
100
150
2000
2010
2020
2030
2040
Government health spending per person
0
50
100
150
2000
2010
2020
2030
2040
Out-of-pocket spending per person
0
25
50
75
100
125
2000
2010
2020
2030
2040
Prepaid private spending per person
0
20
40
60
2000
2010
2020
2030
2040
Scenario
Better
Reference
Worse

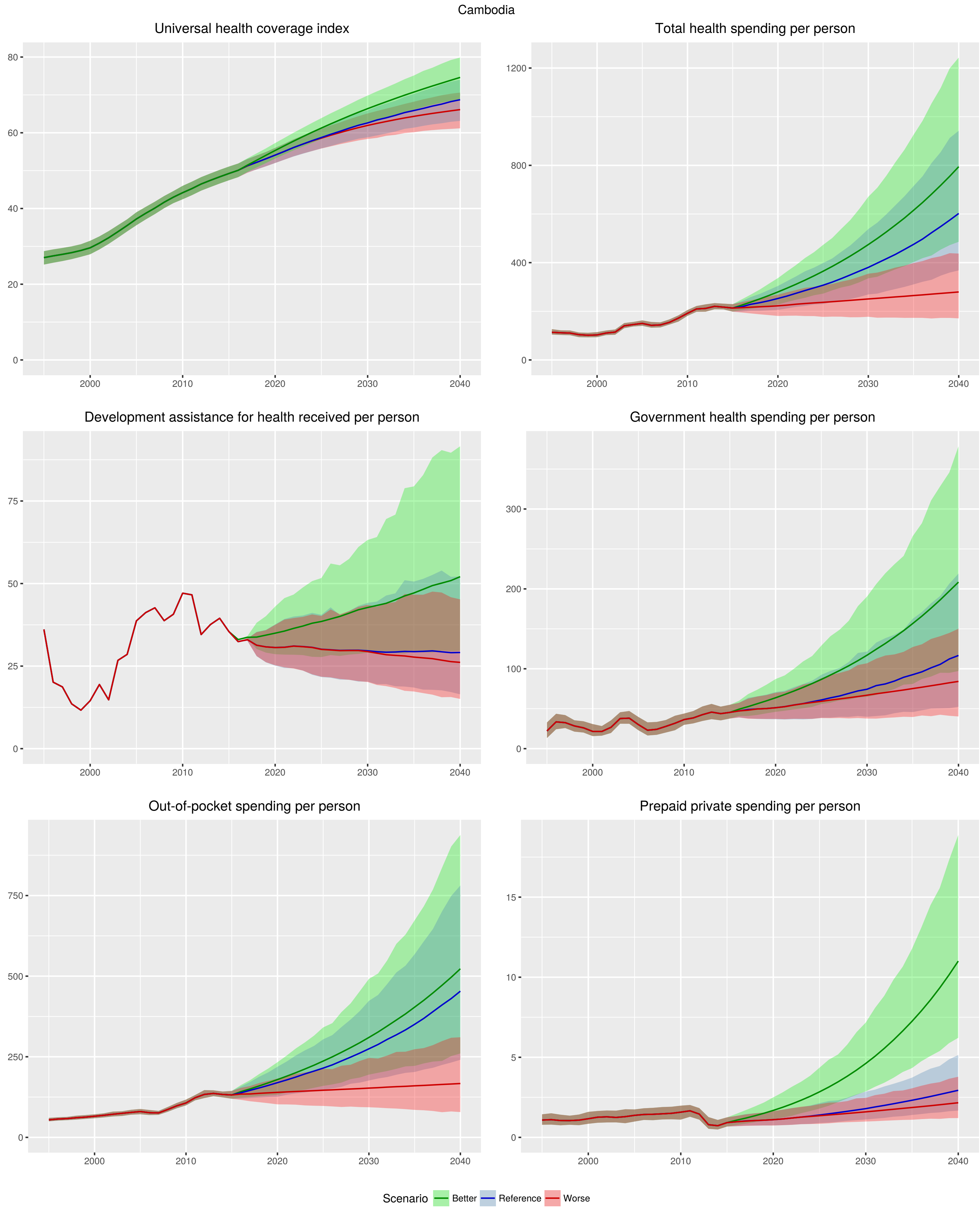

Cambodia
Universal health coverage index
0
20
40
60
80
2000
2010
2020
2030
2040
Total health spending per person
0
400
800
1200
2000
2010
2020
2030
2040
Development assistance for health received per person
0
25
50
75
2000
2010
2020
2030
2040
Government health spending per person
0
100
200
300
2000
2010
2020
2030
2040
Out-of-pocket spending per person
0
250
500
750
2000
2010
2020
2030
2040
Prepaid private spending per person
0
5
10
15
2000
2010
2020
2030
2040
Scenario
Better
Reference
Worse

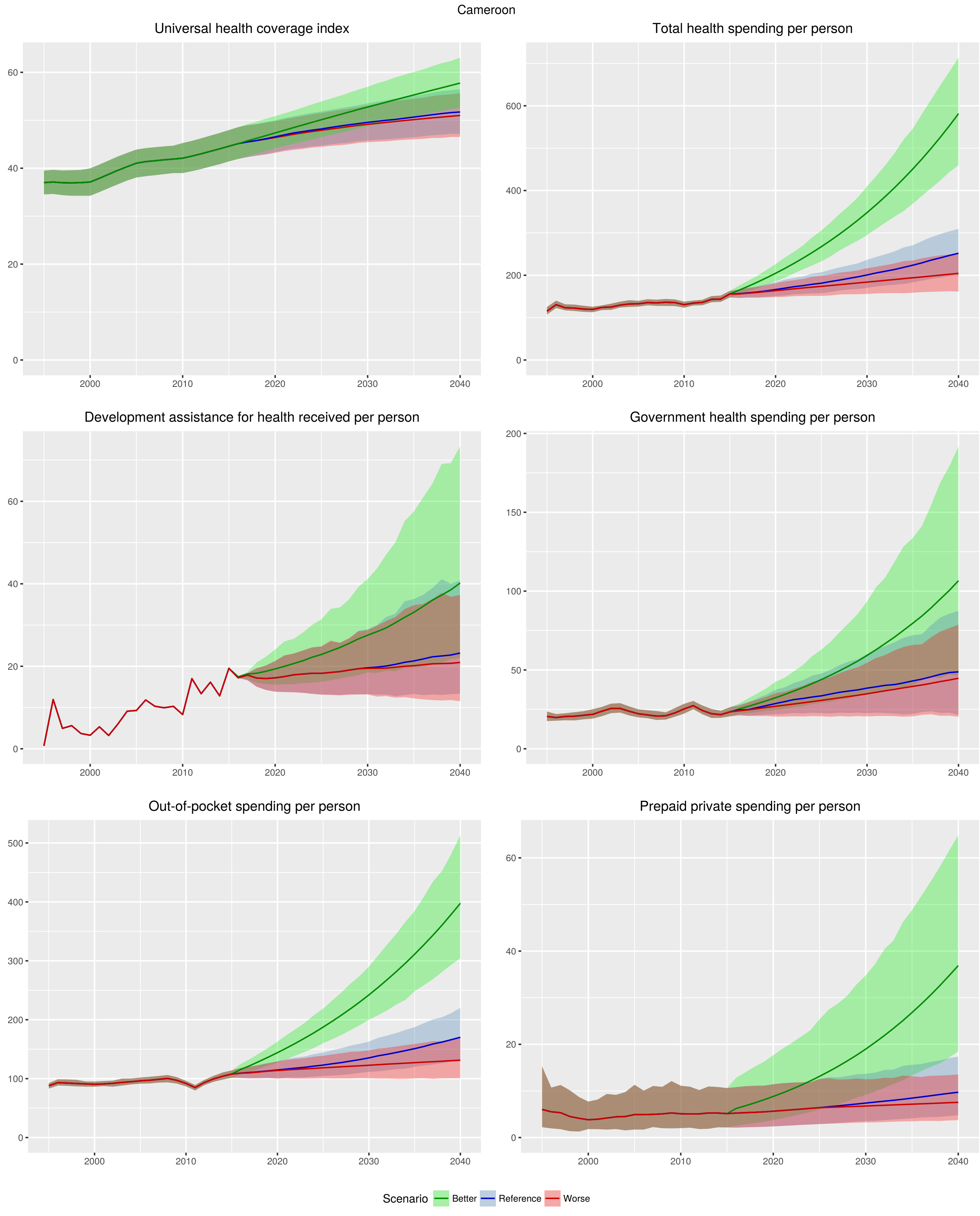

Cameroon
Universal health coverage index
Total health spending per person
Development assistance for health received per person
Government health spending per person
Out-of-pocket spending per person
Prepaid private spending per person
Scenario
Better
Reference
Worse

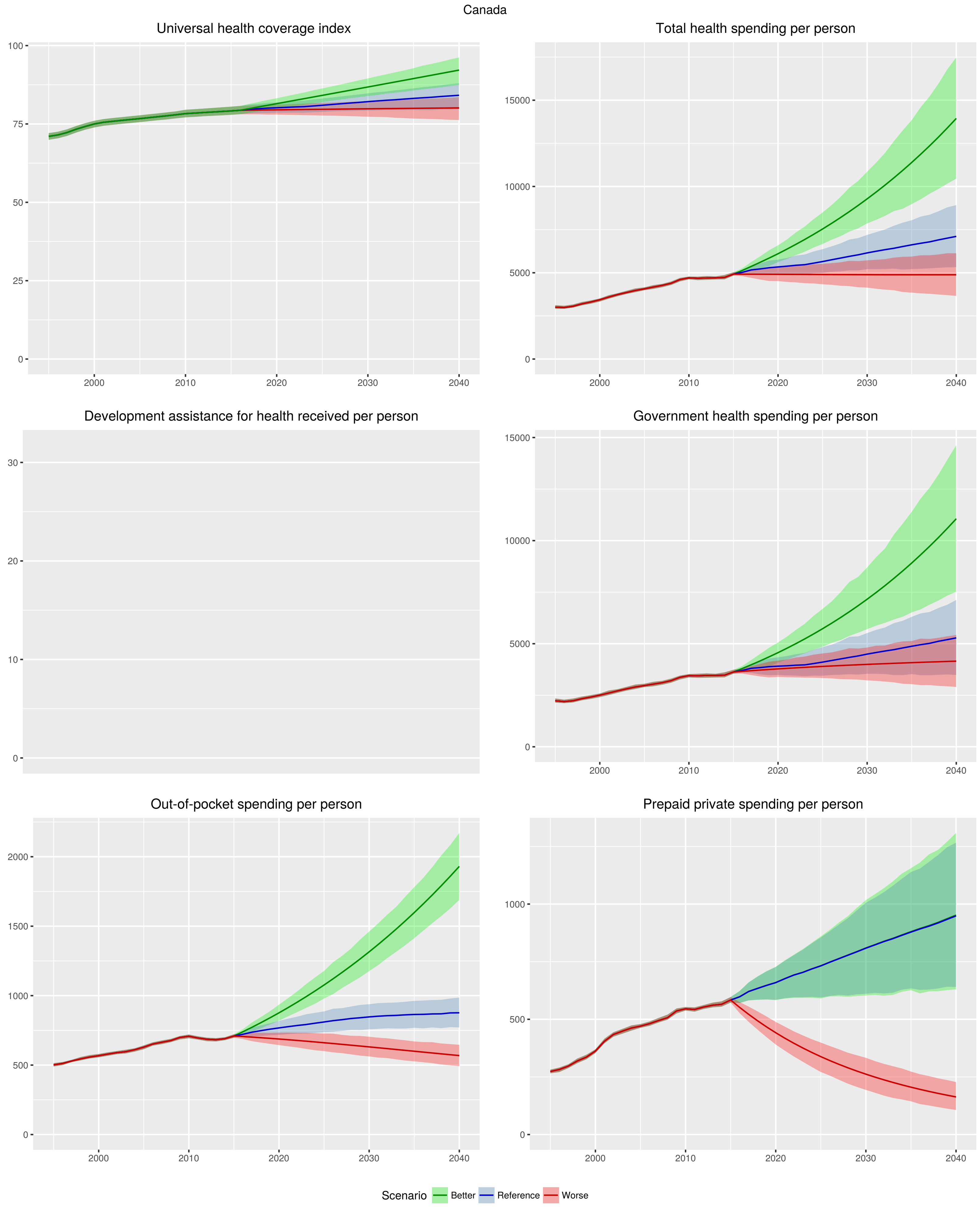
Canada
Universal health coverage index
100
75
50
25
0
2000
2010
2020
2030
2040
Total health spending per person
15000
10000
5000
0
2000
2010
2020
2030
2040
Development assistance for health received per person
30
20
10
0
Government health spending per person
15000
10000
5000
0
2000
2010
2020
2030
2040
Out-of-pocket spending per person
2000
1500
1000
500
0
2000
2010
2020
2030
2040
Prepaid private spending per person
1000
500
0
2000
2010
2020
2030
2040
Scenario
Better
Reference
Worse

Cape Verde

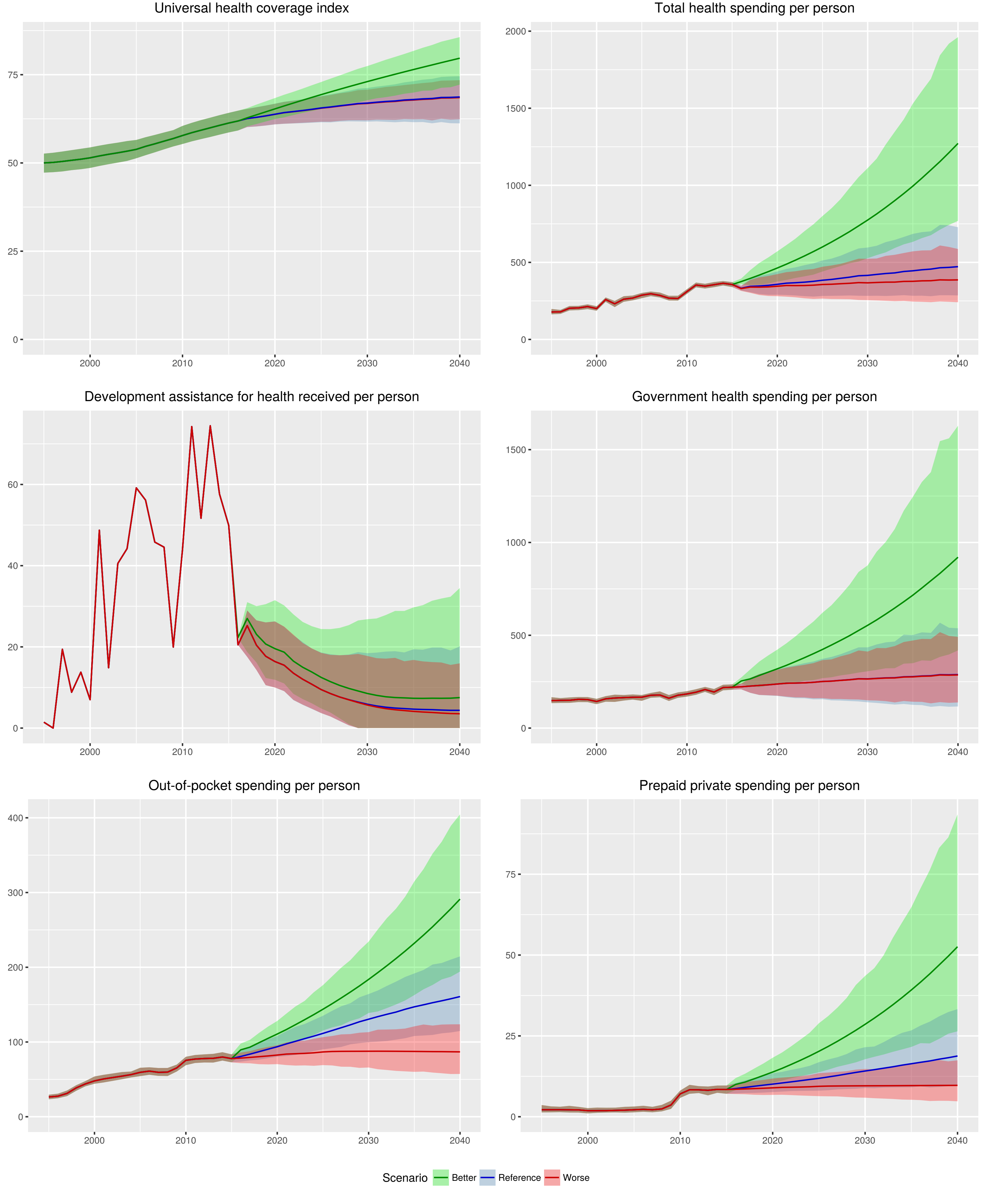

Universal health coverage index
Total health spending per person
Development assistance for health received per person
Government health spending per person
Out-of-pocket spending per person
Prepaid private spending per person
Scenario
Better
Reference
Worse

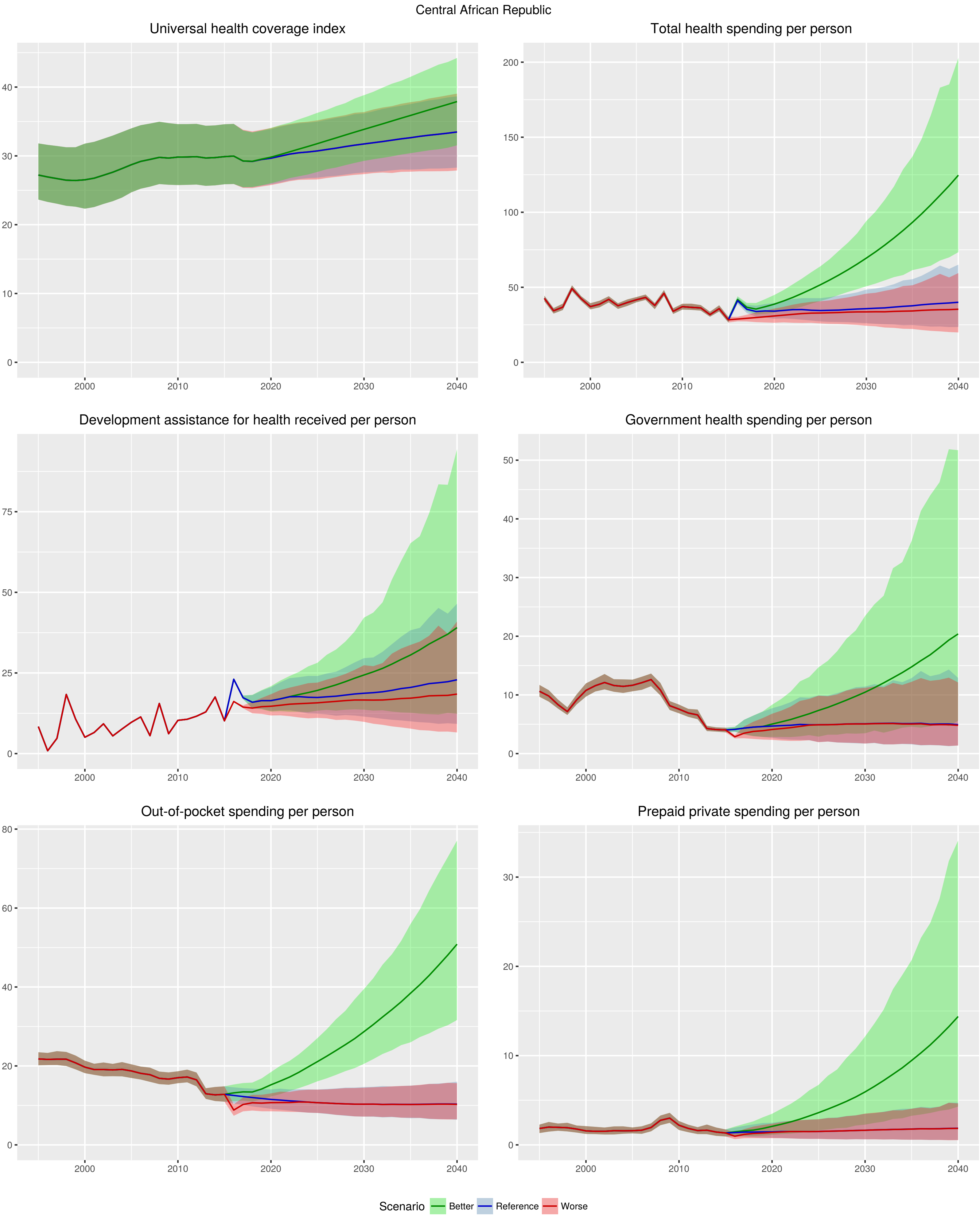

Central African Republic
Universal health coverage index
Total health spending per person
Development assistance for health received per person
Government health spending per person
Out-of-pocket spending per person
Prepaid private spending per person
0
10
20
30
40
50
75
100
150
200
2000
2010
2020
2030
2040
Scenario
Better
Reference
Worse

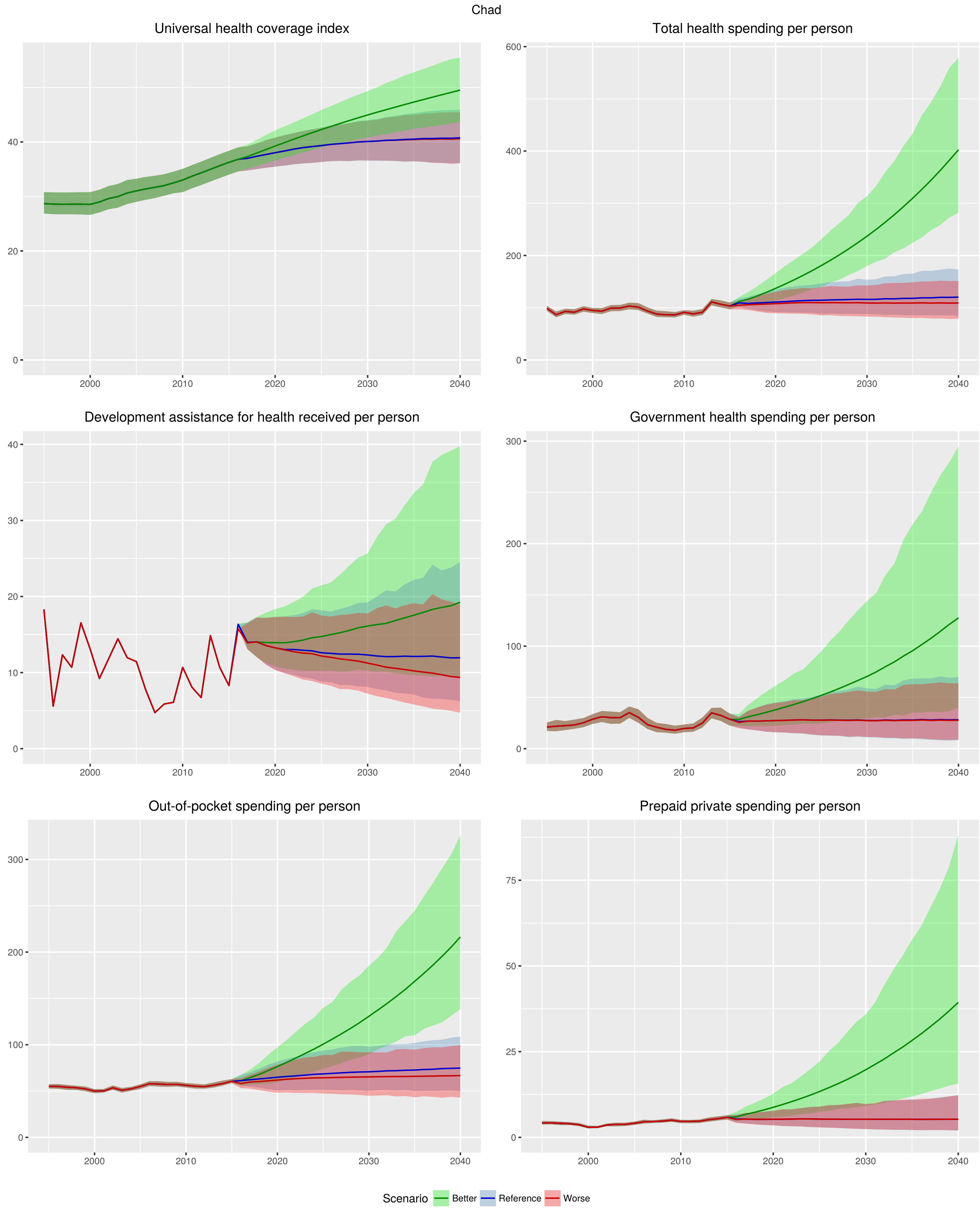
Chad
Universal health coverage index
Total health spending per person
Development assistance for health received per person
Government health spending per person
Out-of-pocket spending per person
Prepaid private spending per person
0
20
40
200
400
600
10
30
100
300
25
50
75
2000
2010
2020
2030
2040
Scenario
Better
Reference
Worse

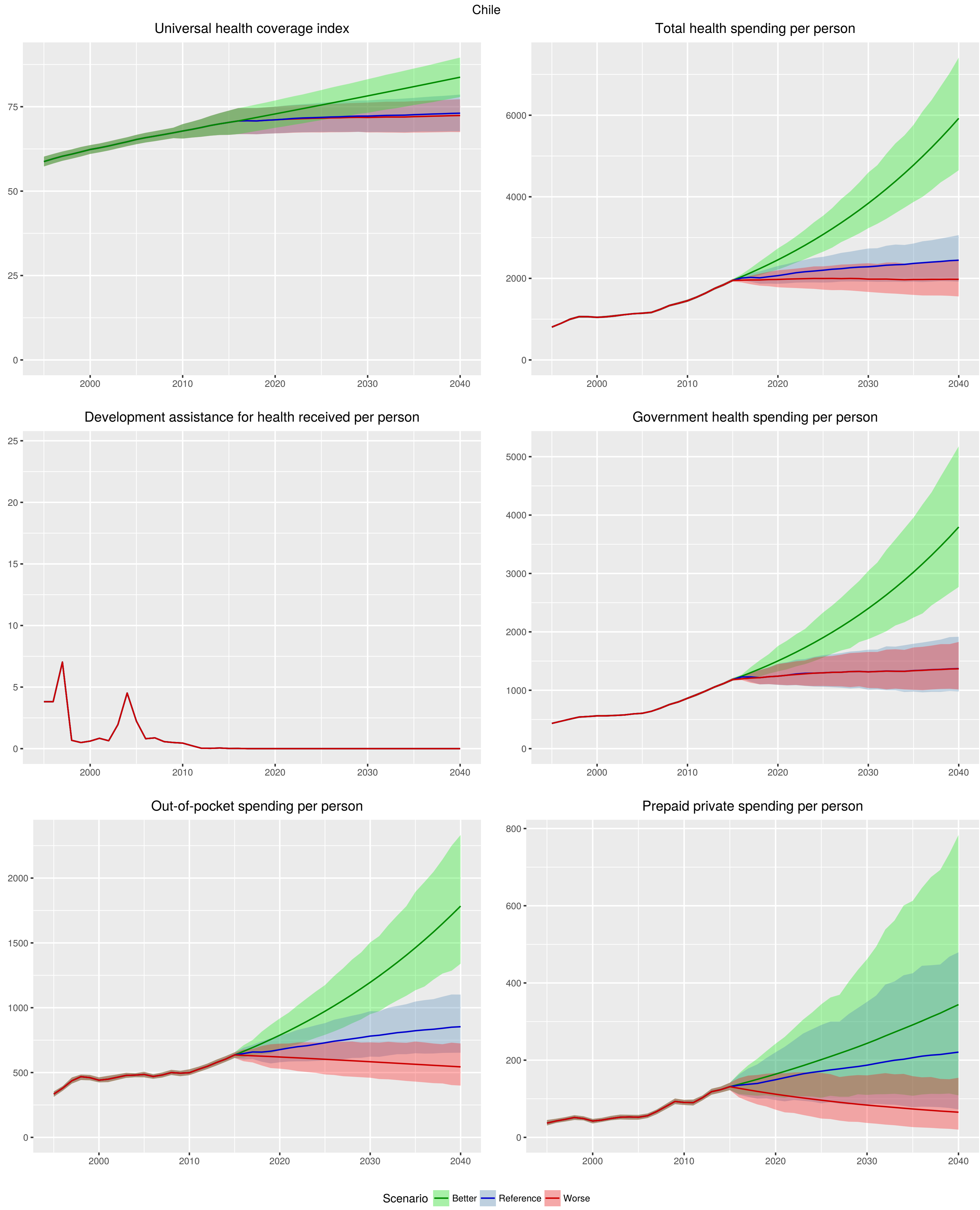
Chile
Universal health coverage index
0
25
50
75
2000
2010
2020
2030
2040
Total health spending per person
0
2000
4000
6000
2000
2010
2020
2030
2040
Development assistance for health received per person
0
5
10
15
20
25
2000
2010
2020
2030
2040
Government health spending per person
0
1000
2000
3000
4000
5000
2000
2010
2020
2030
2040
Out-of-pocket spending per person
0
500
1000
1500
2000
2000
2010
2020
2030
2040
Prepaid private spending per person
0
200
400
600
800
2000
2010
2020
2030
2040
Scenario
Better
Reference
Worse

China

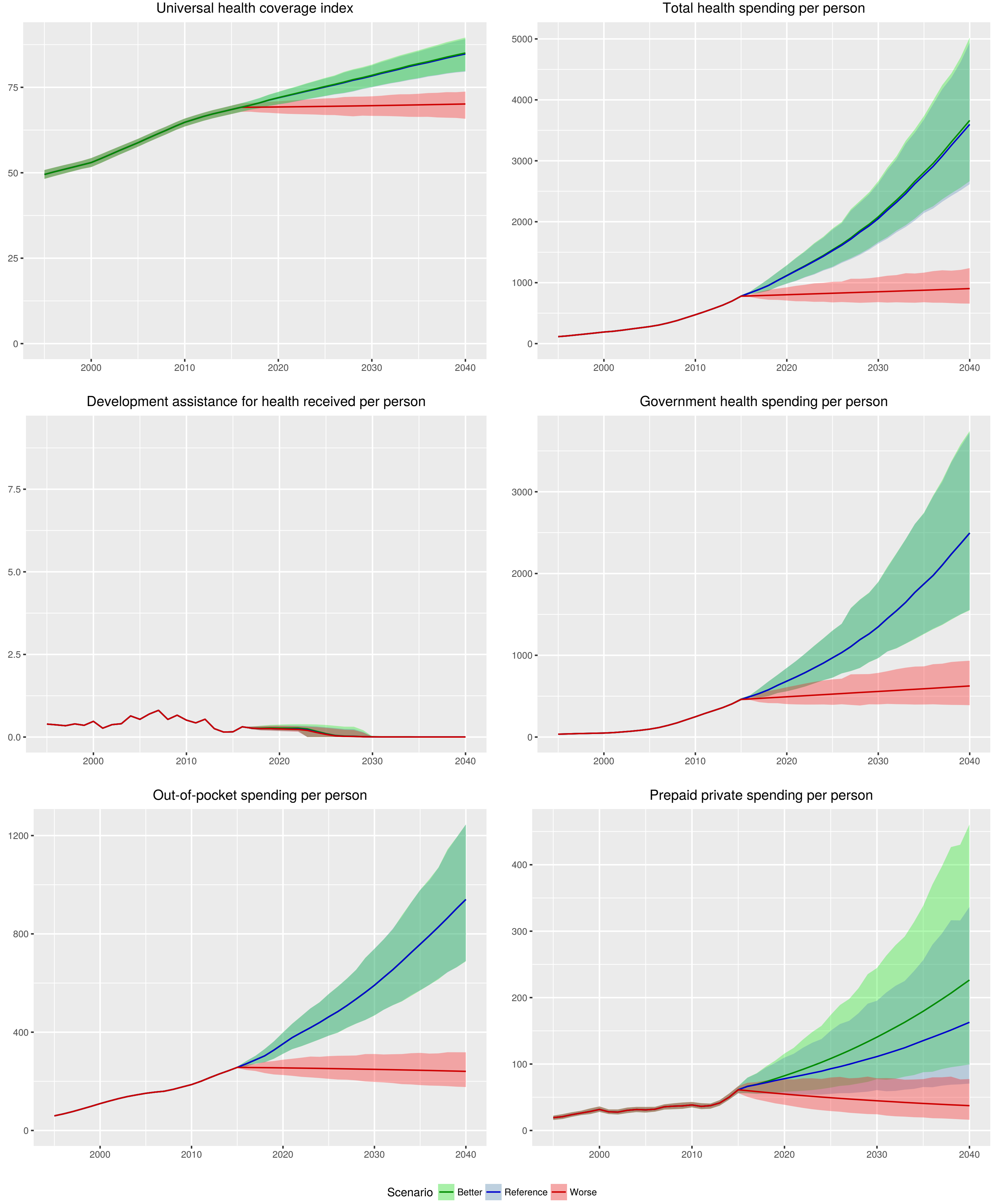
Universal health coverage index
Total health spending per person
Development assistance for health received per person
Government health spending per person
Out-of-pocket spending per person
Prepaid private spending per person
0
25
50
75
1000
2000
3000
4000
5000
0.0
2.5
5.0
7.5
400
800
1200
100
200
300
400
2000
2010
2020
2030
2040
Scenario
Better
Reference
Worse

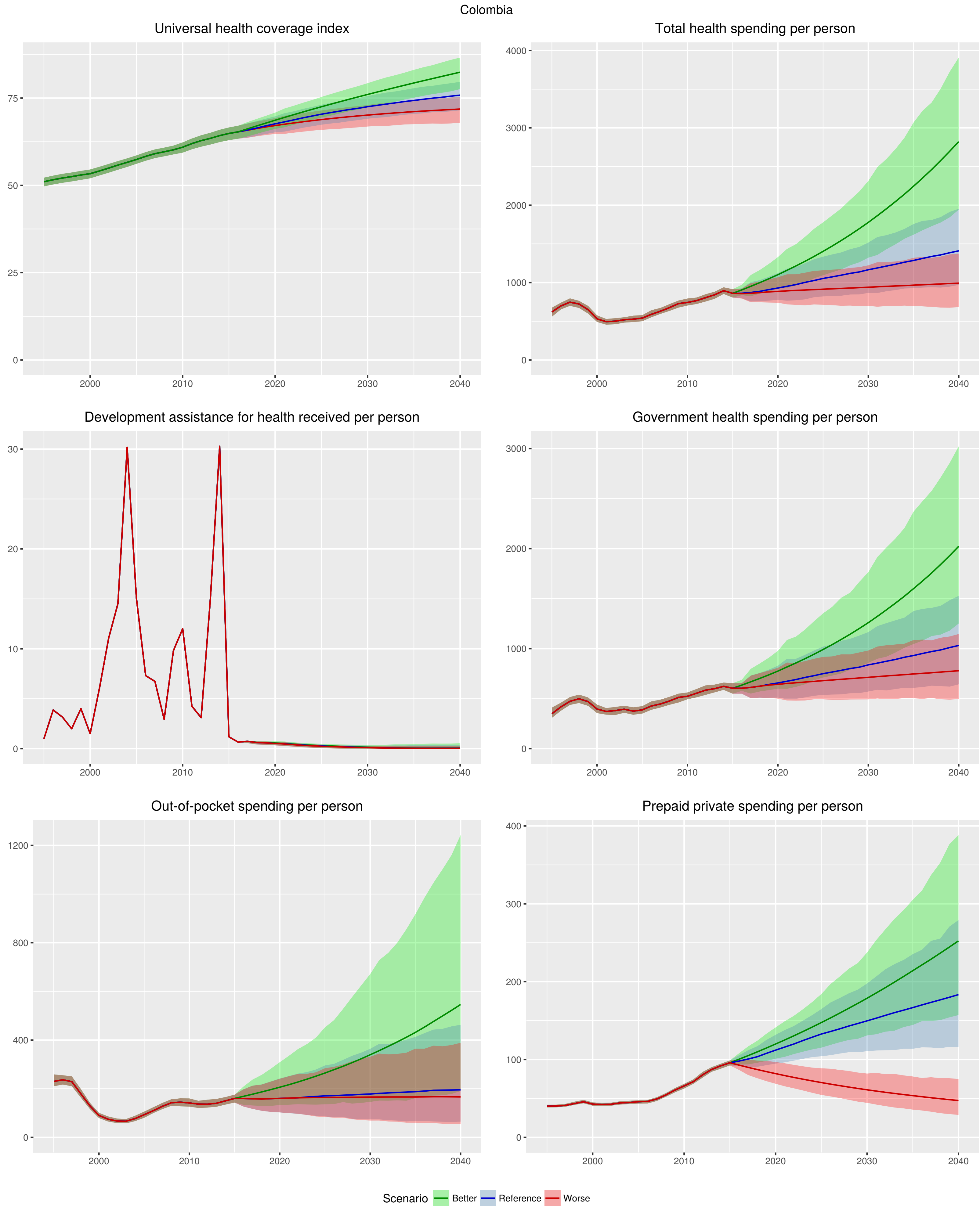

Colombia
Universal health coverage index
Total health spending per person
Development assistance for health received per person
Government health spending per person
Out-of-pocket spending per person
Prepaid private spending per person
Scenario
Better
Reference
Worse

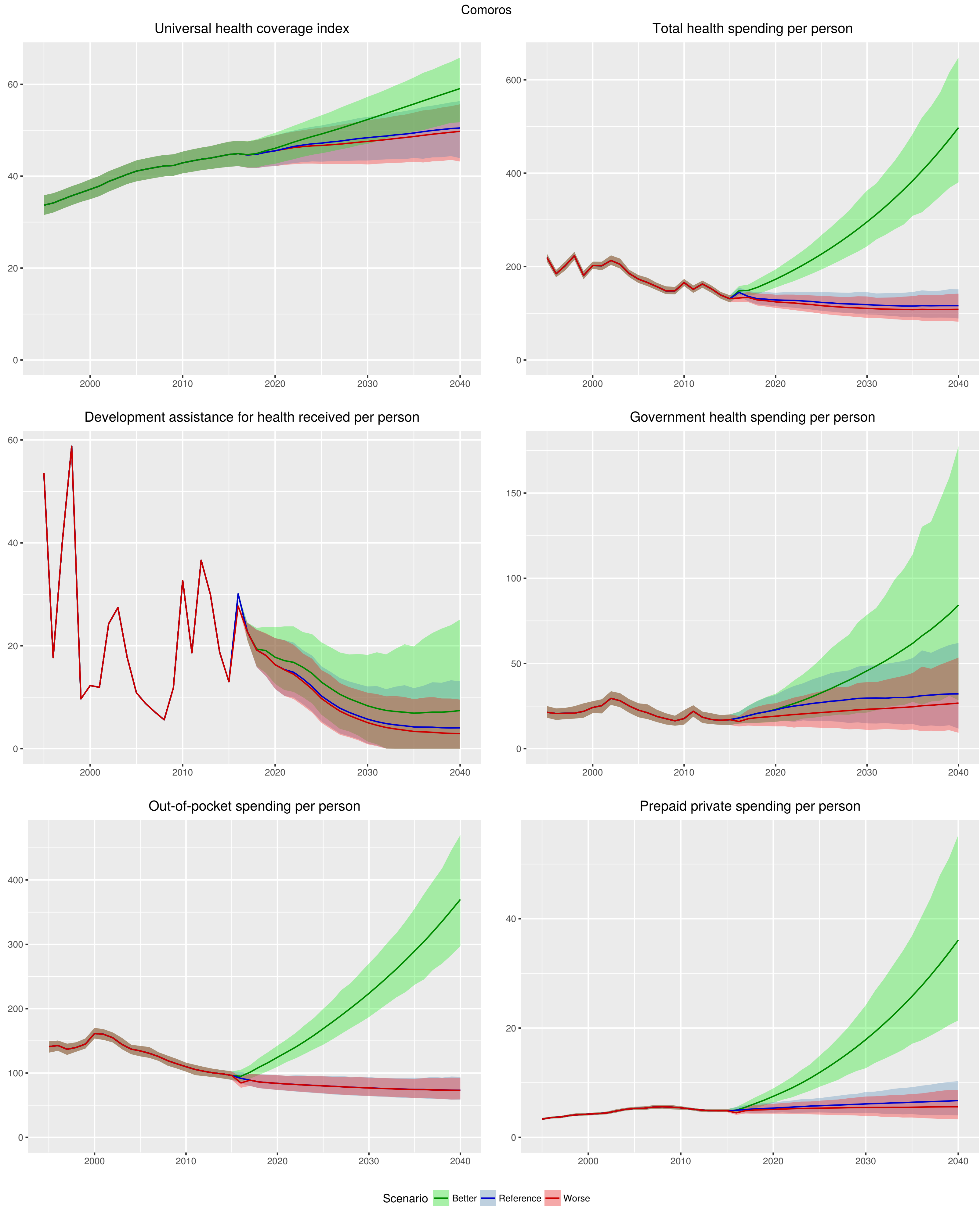

Comoros
Universal health coverage index
Total health spending per person
Development assistance for health received per person
Government health spending per person
Out-of-pocket spending per person
Prepaid private spending per person
Scenario
Better
Reference
Worse

Congo

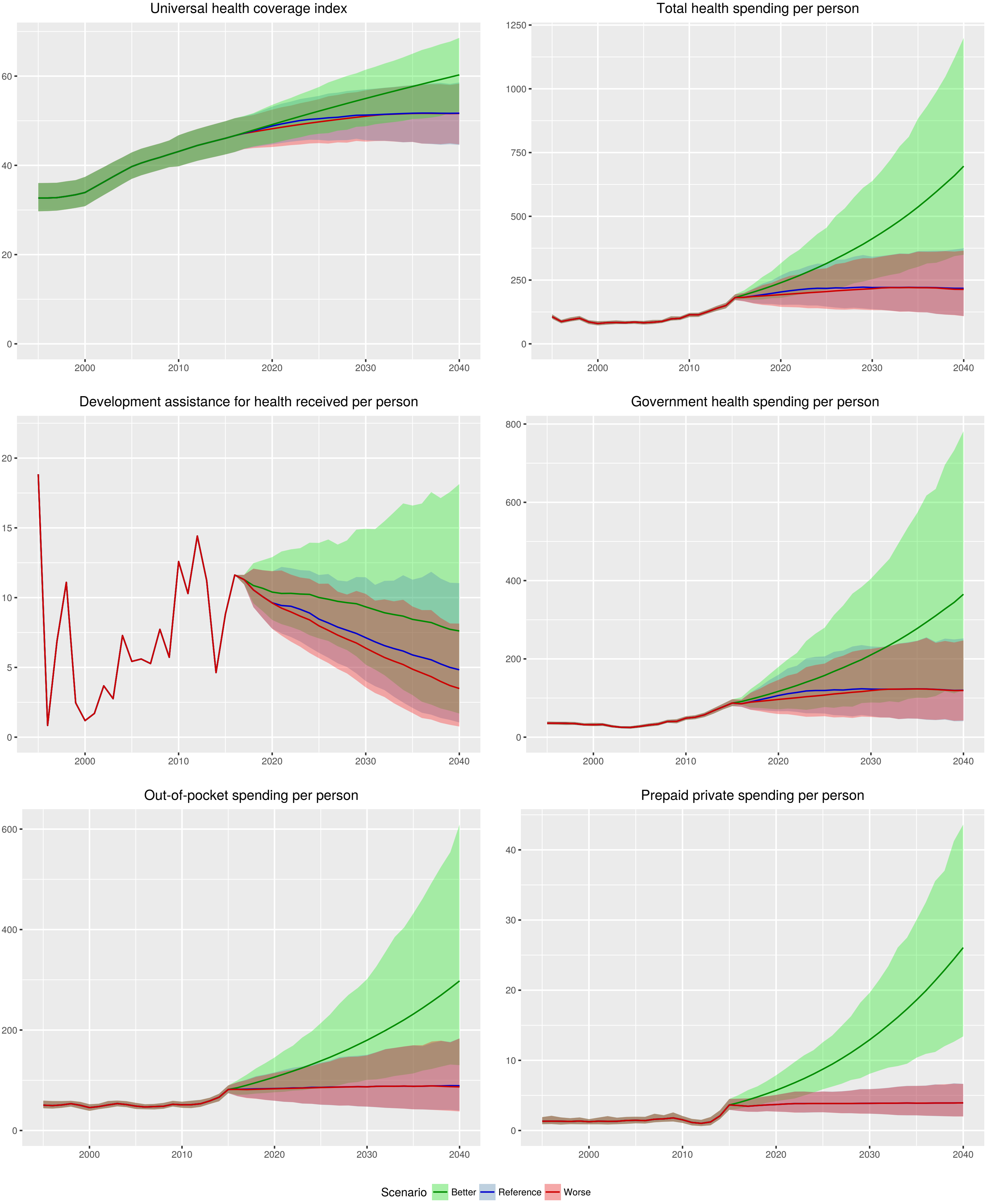
Universal health coverage index
Total health spending per person
Development assistance for health received per person
Government health spending per person
Out-of-pocket spending per person
Prepaid private spending per person
2000
2010
2020
2030
2040
0
20
40
60
250
500
750
1000
1250
5
10
15
200
400
600
800
30
Scenario
Better
Reference
Worse

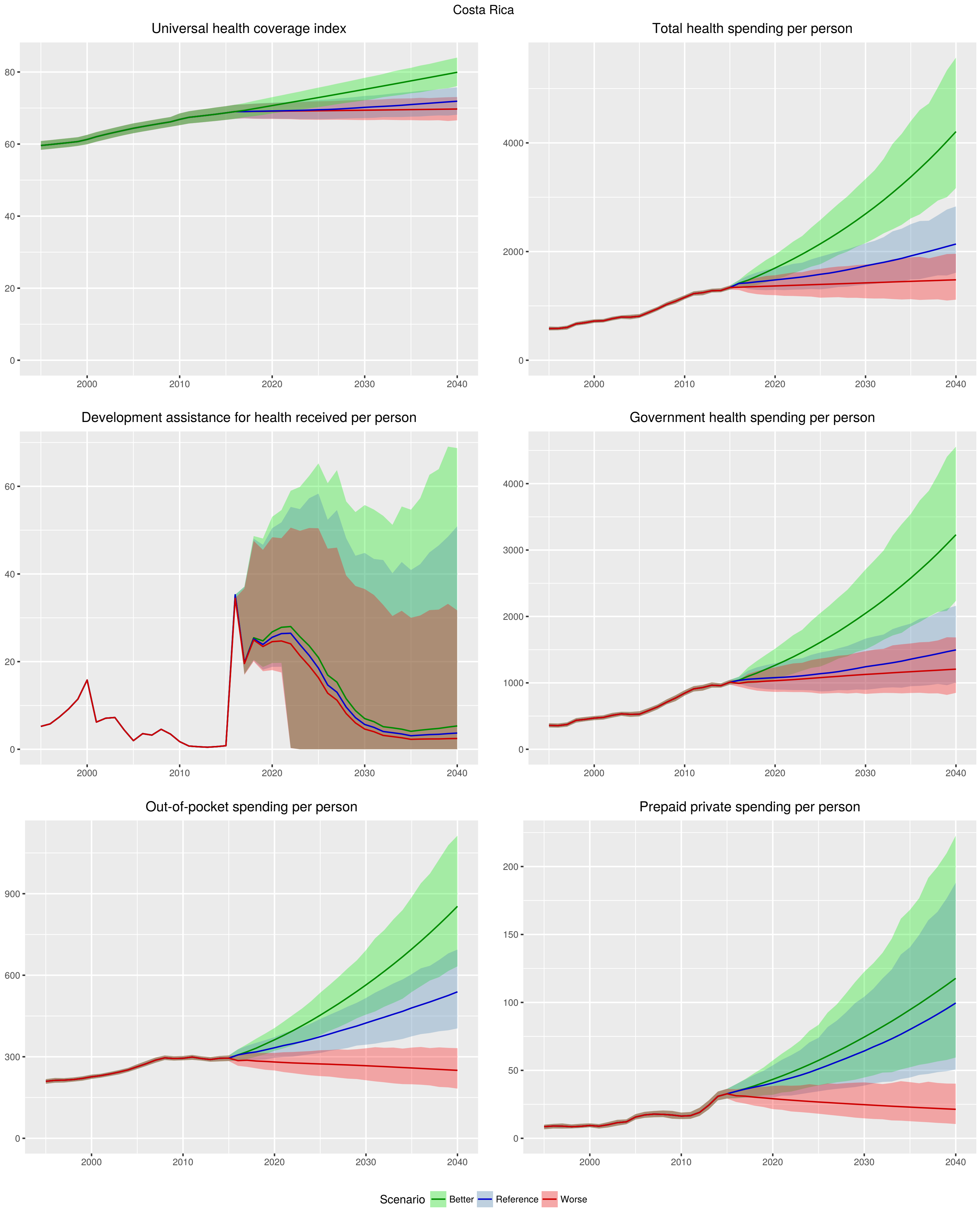
Costa Rica
Universal health coverage index
0
20
40
60
80
2000
2010
2020
2030
2040
Total health spending per person
0
2000
4000
Development assistance for health received per person
0
20
40
60
Government health spending per person
0
1000
2000
3000
4000
Out-of-pocket spending per person
0
300
600
900
Prepaid private spending per person
0
50
100
150
200
Scenario
Better
Reference
Worse

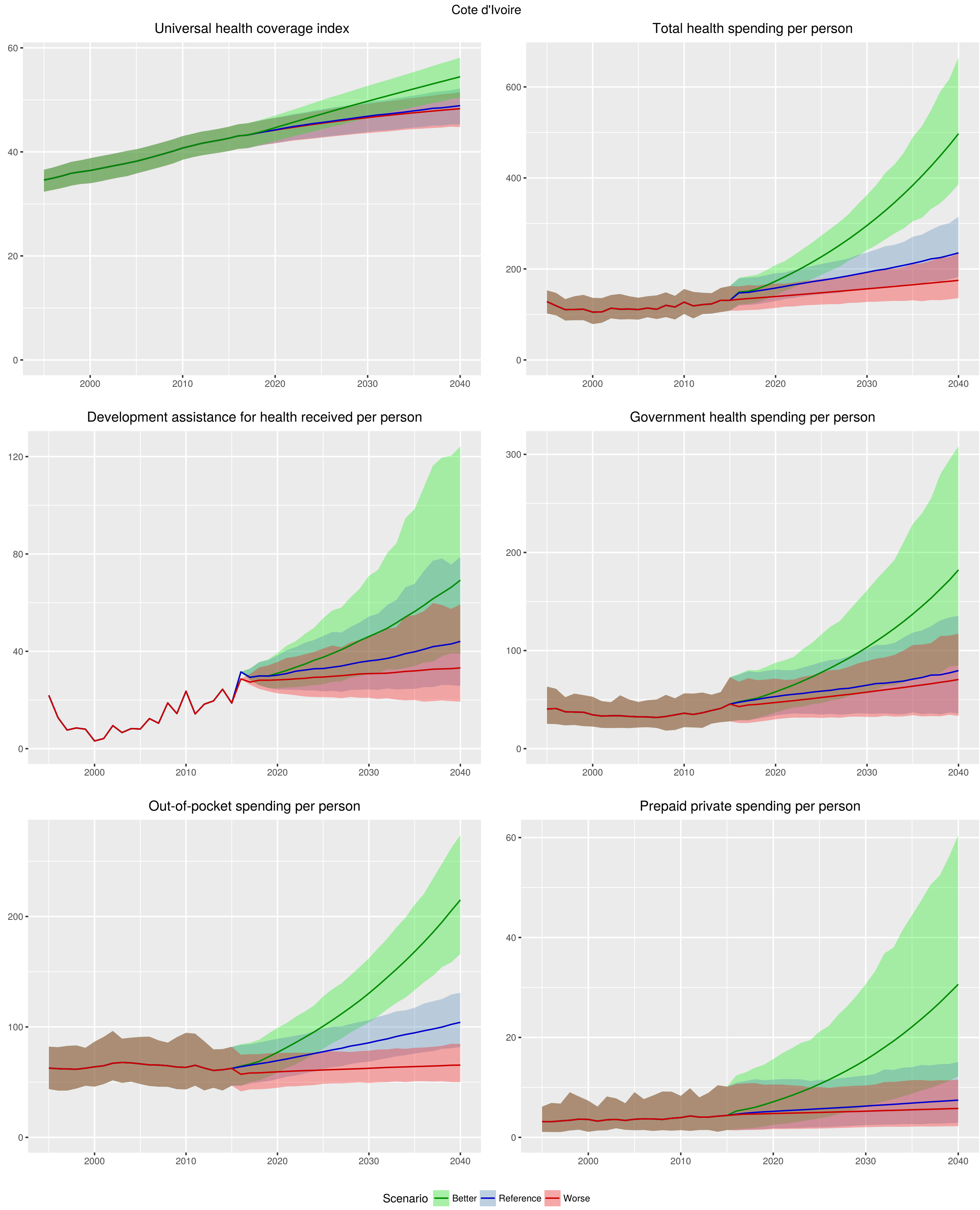

Cote d'Ivoire
Universal health coverage index
Total health spending per person
Development assistance for health received per person
Government health spending per person
Out-of-pocket spending per person
Prepaid private spending per person
Scenario
Better
Reference
Worse

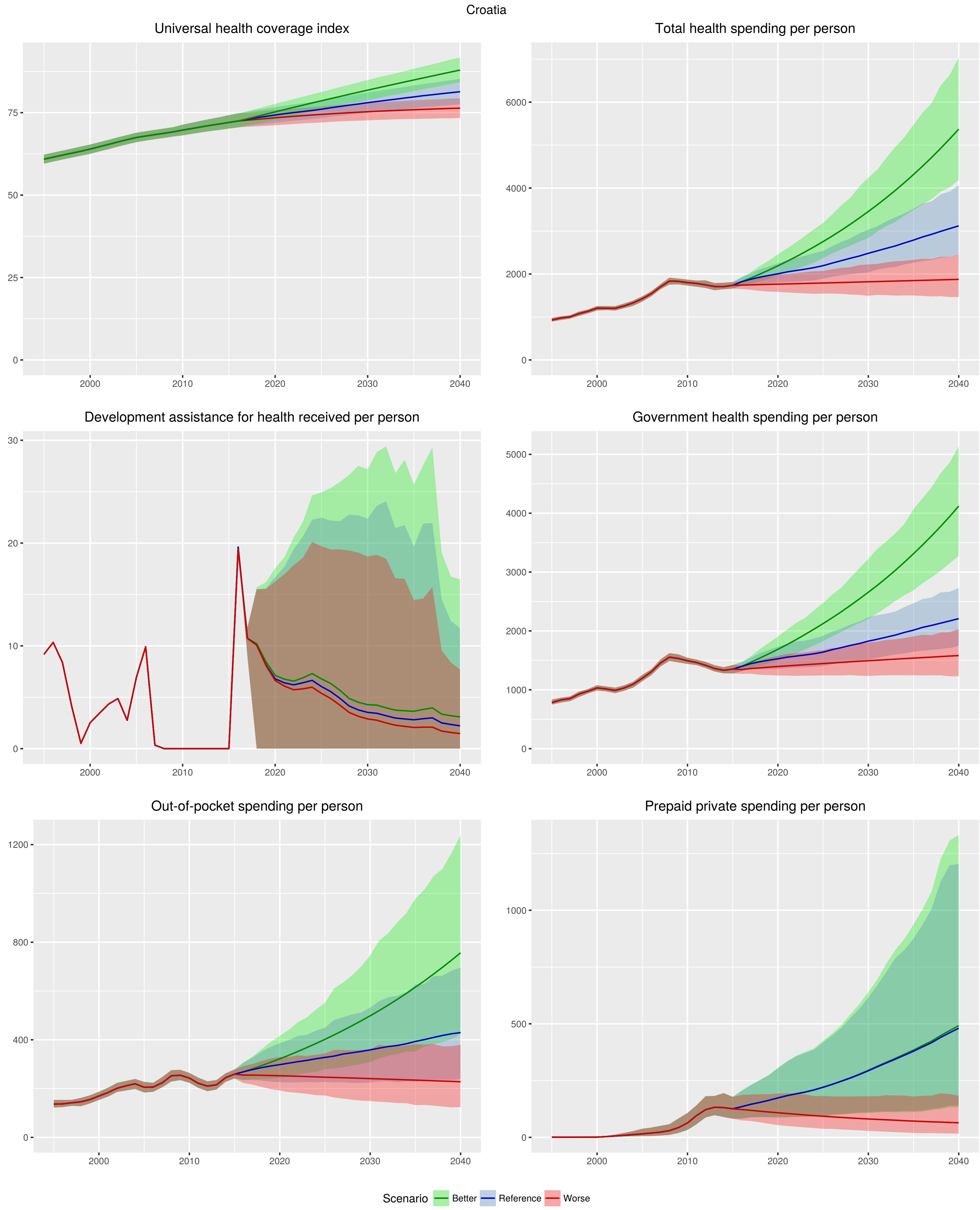

Croatia
Universal health coverage index
Total health spending per person
Development assistance for health received per person
Government health spending per person
Out-of-pocket spending per person
Prepaid private spending per person
0
25
50
75
0
2000
4000
6000
0
10
20
30
0
1000
2000
3000
4000
5000
0
400
800
1200
0
500
1000
2000
2010
2020
2030
2040
Scenario
Better
Reference
Worse

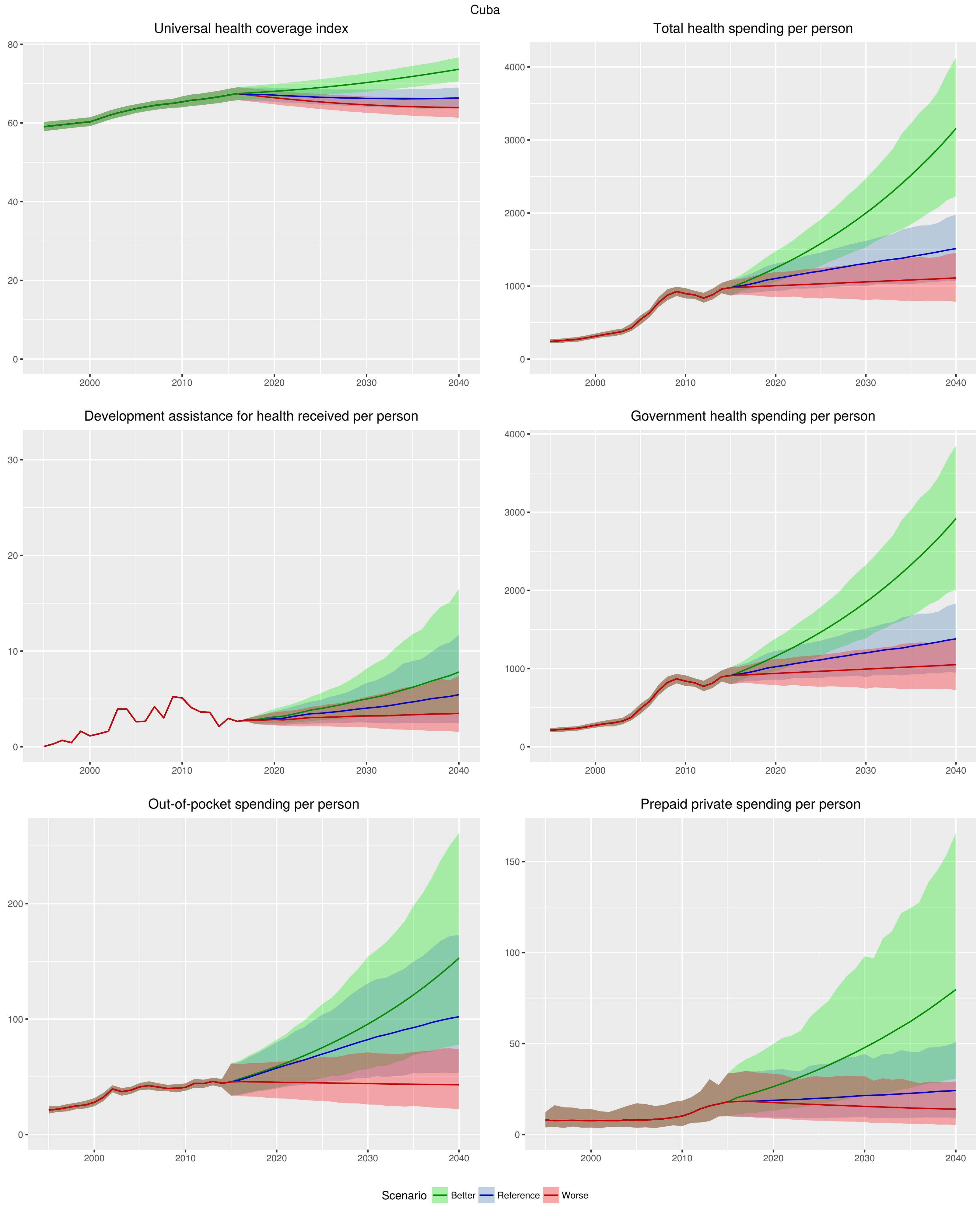
Cuba
Universal health coverage index
Total health spending per person
Development assistance for health received per person
Government health spending per person
Out-of-pocket spending per person
Prepaid private spending per person
0
20
40
60
80
1000
2000
3000
4000
10
30
50
100
150
200
2000
2010
2020
2030
2040
Scenario
Better
Reference
Worse

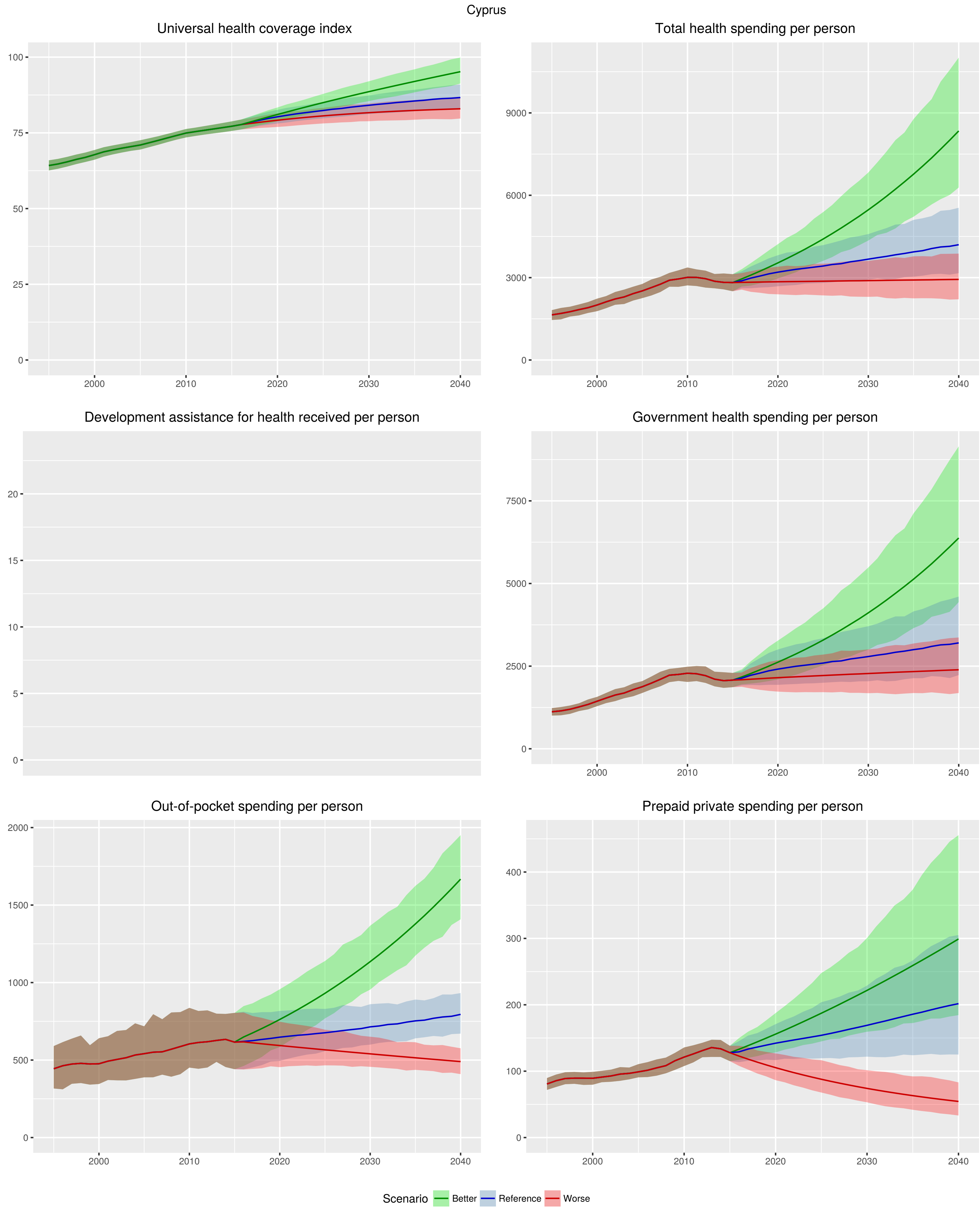
Cyprus
Universal health coverage index
Total health spending per person
Development assistance for health received per person
Government health spending per person
Out-of-pocket spending per person
Prepaid private spending per person
Scenario
Better
Reference
Worse

Czech Republic

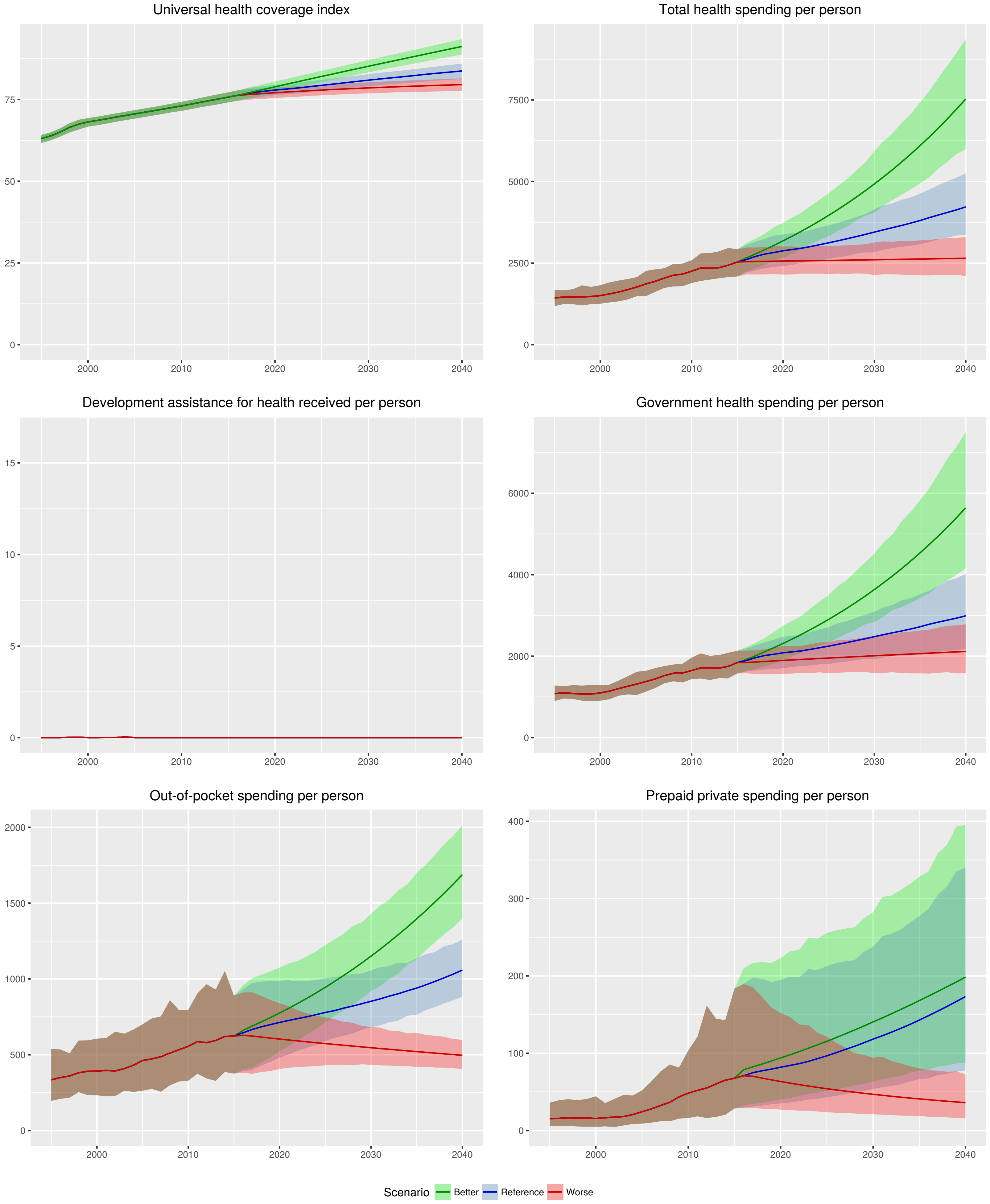

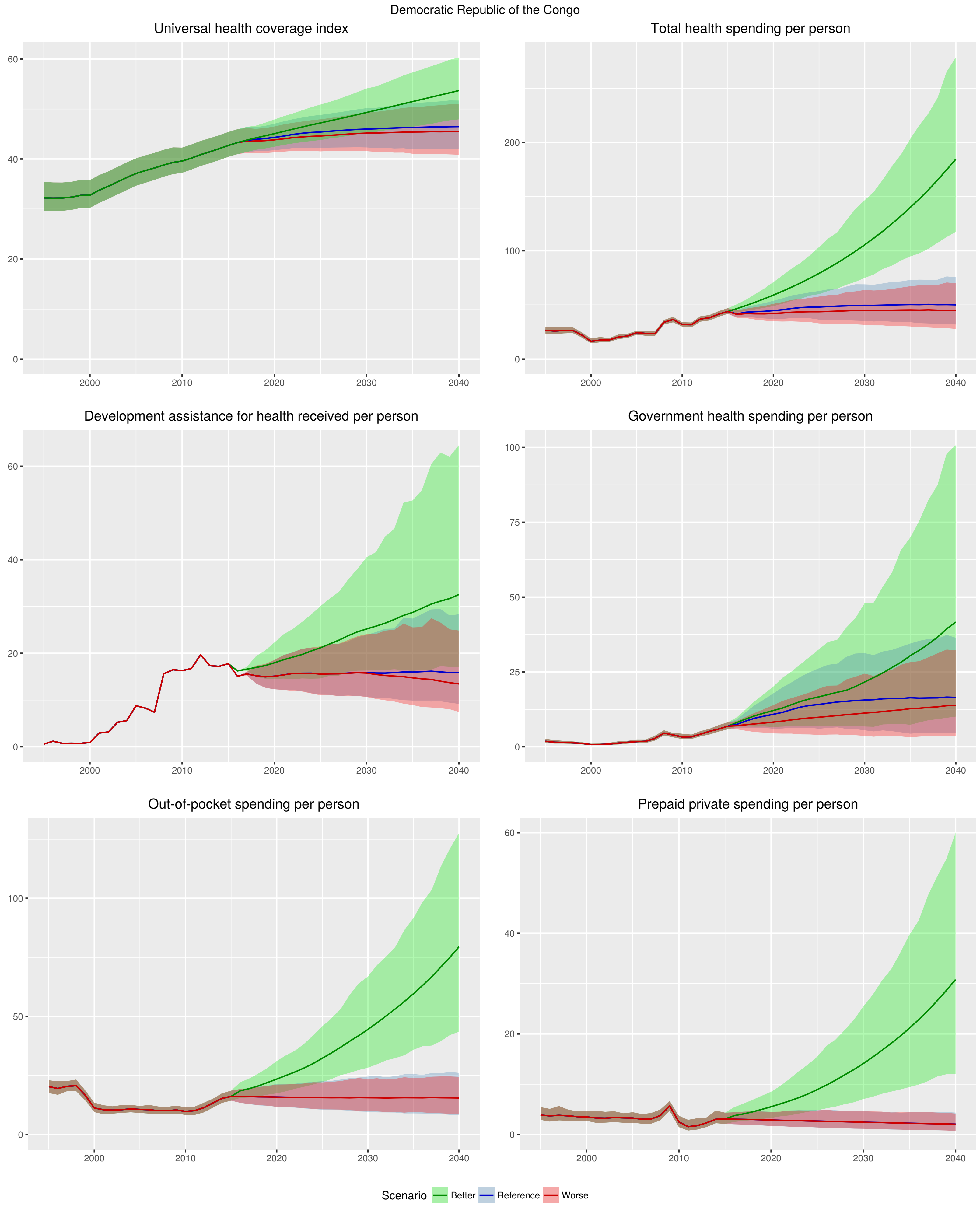
Democratic Republic of the Congo
Universal health coverage index
Total health spending per person
Development assistance for health received per person
Government health spending per person
Out-of-pocket spending per person
Prepaid private spending per person
0
20
40
60
100
200
75
25
50
2000
2010
2020
2030
2040
Scenario
Better
Reference
Worse

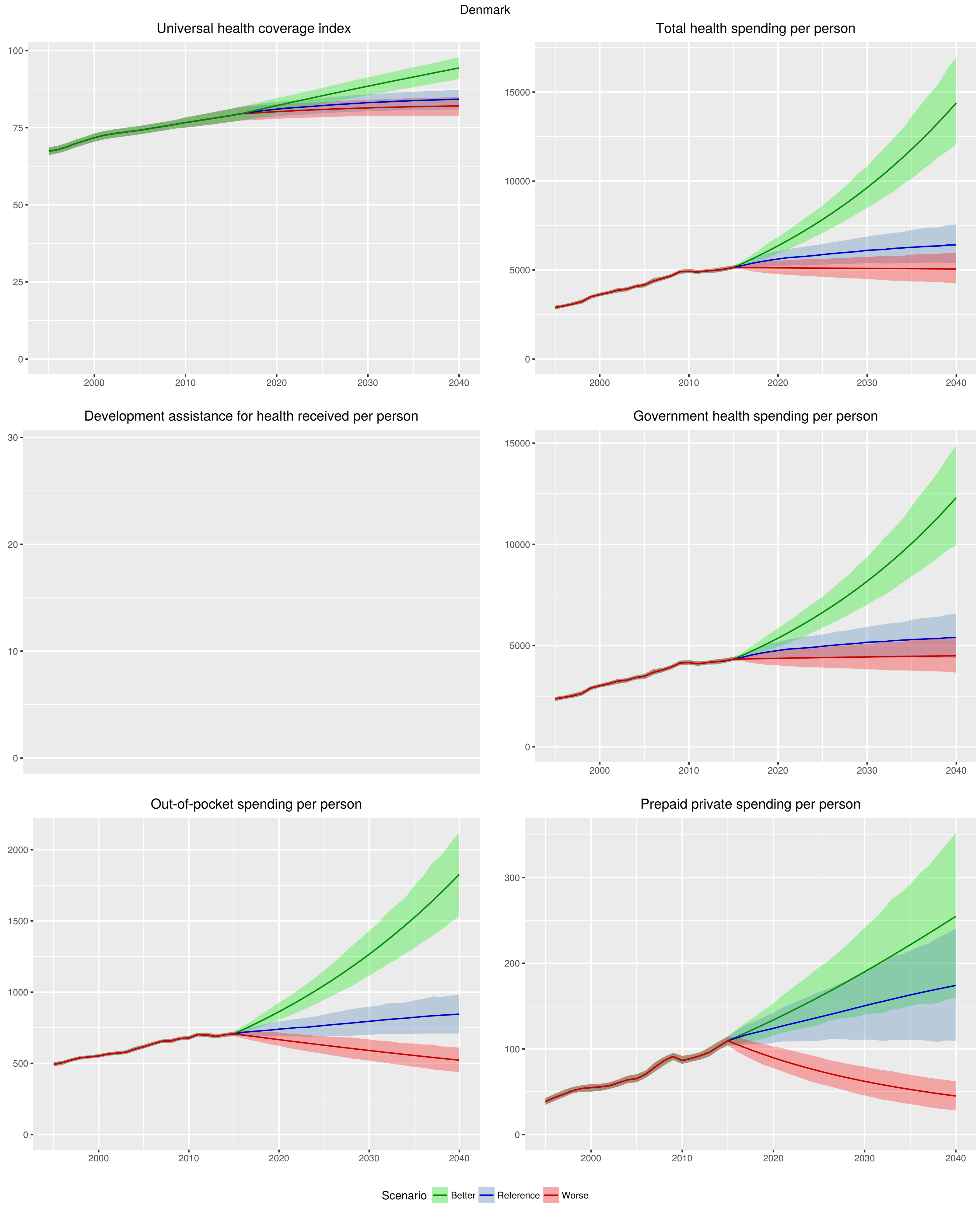

Denmark
Universal health coverage index
Total health spending per person
Development assistance for health received per person
Government health spending per person
Out-of-pocket spending per person
Prepaid private spending per person
Scenario
Better
Reference
Worse

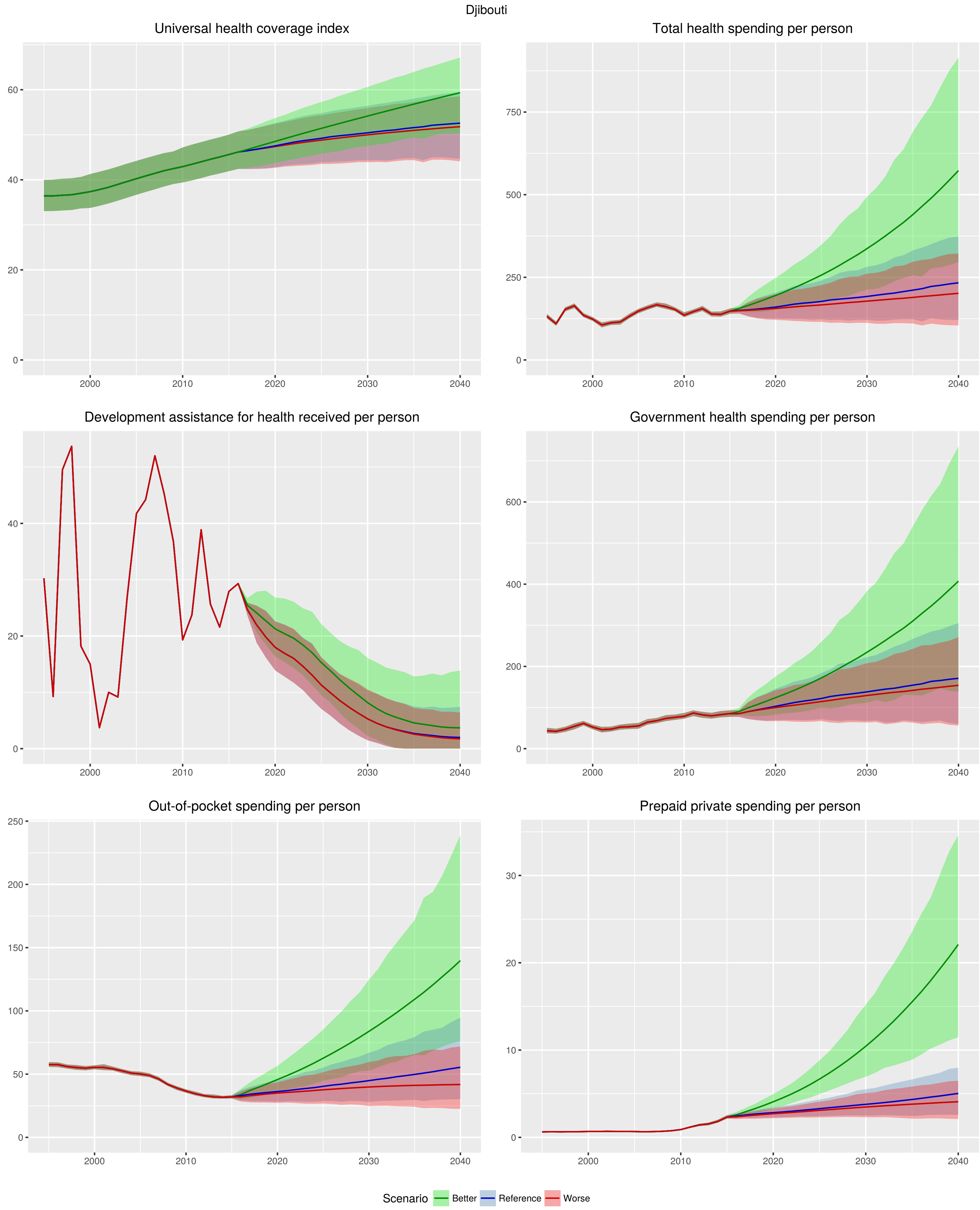

Djibouti
Universal health coverage index
Total health spending per person
Development assistance for health received per person
Government health spending per person
Out-of-pocket spending per person
Prepaid private spending per person
Scenario
Better
Reference
Worse

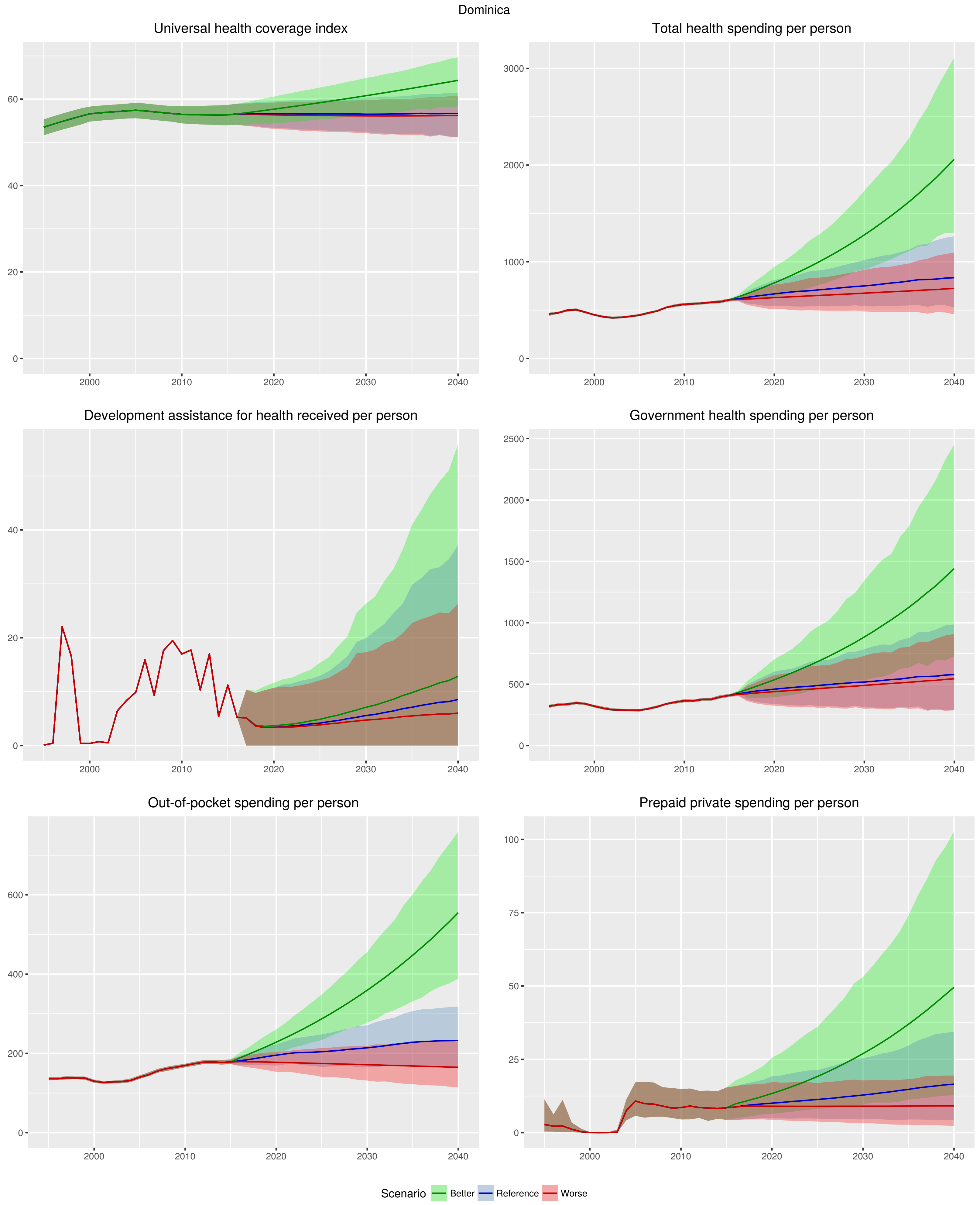

Dominica
Universal health coverage index
Total health spending per person
Development assistance for health received per person
Government health spending per person
Out-of-pocket spending per person
Prepaid private spending per person
Scenario
Better
Reference
Worse

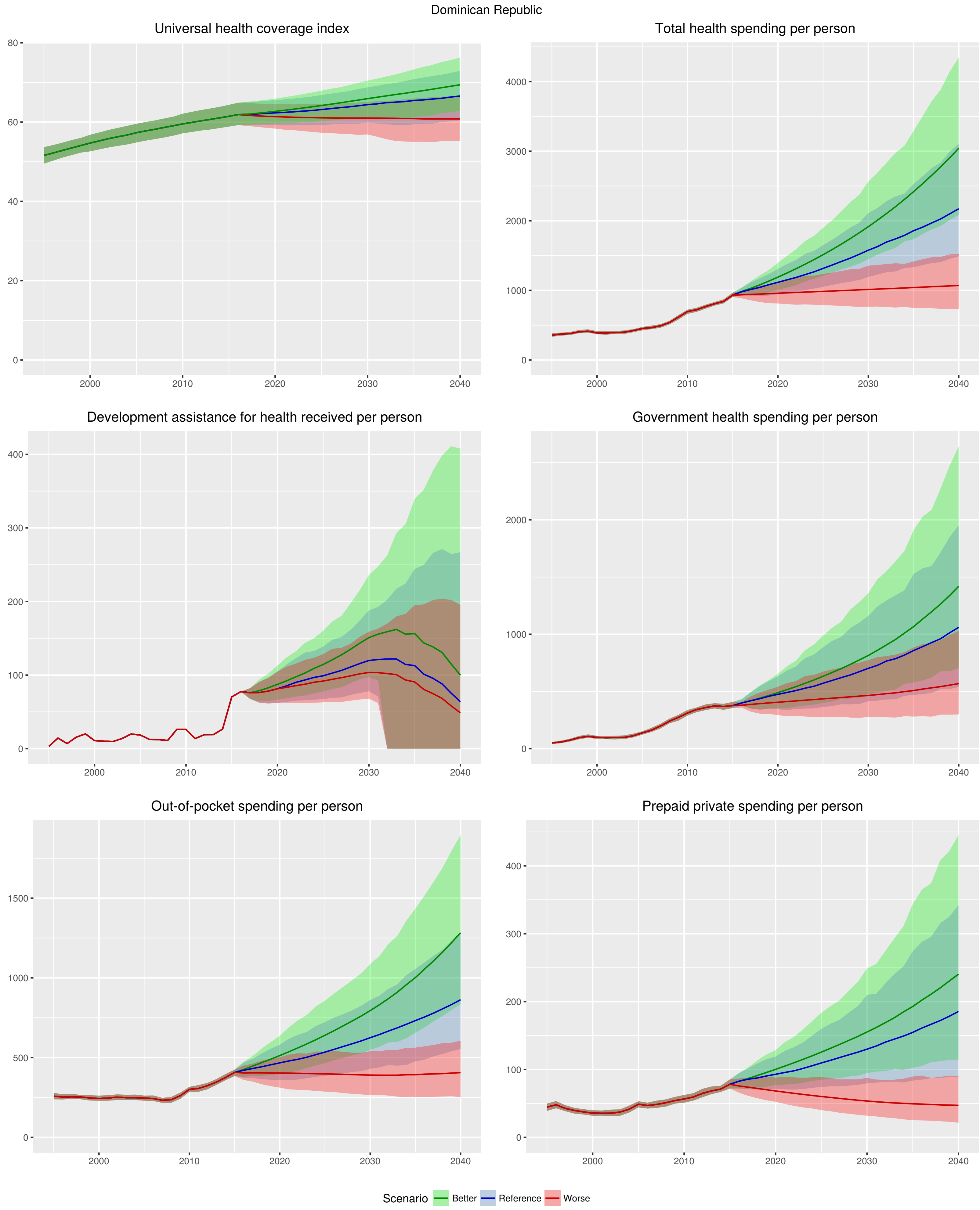

Dominican Republic
Universal health coverage index
Total health spending per person
Development assistance for health received per person
Government health spending per person
Out-of-pocket spending per person
Prepaid private spending per person
Scenario
Better
Reference
Worse

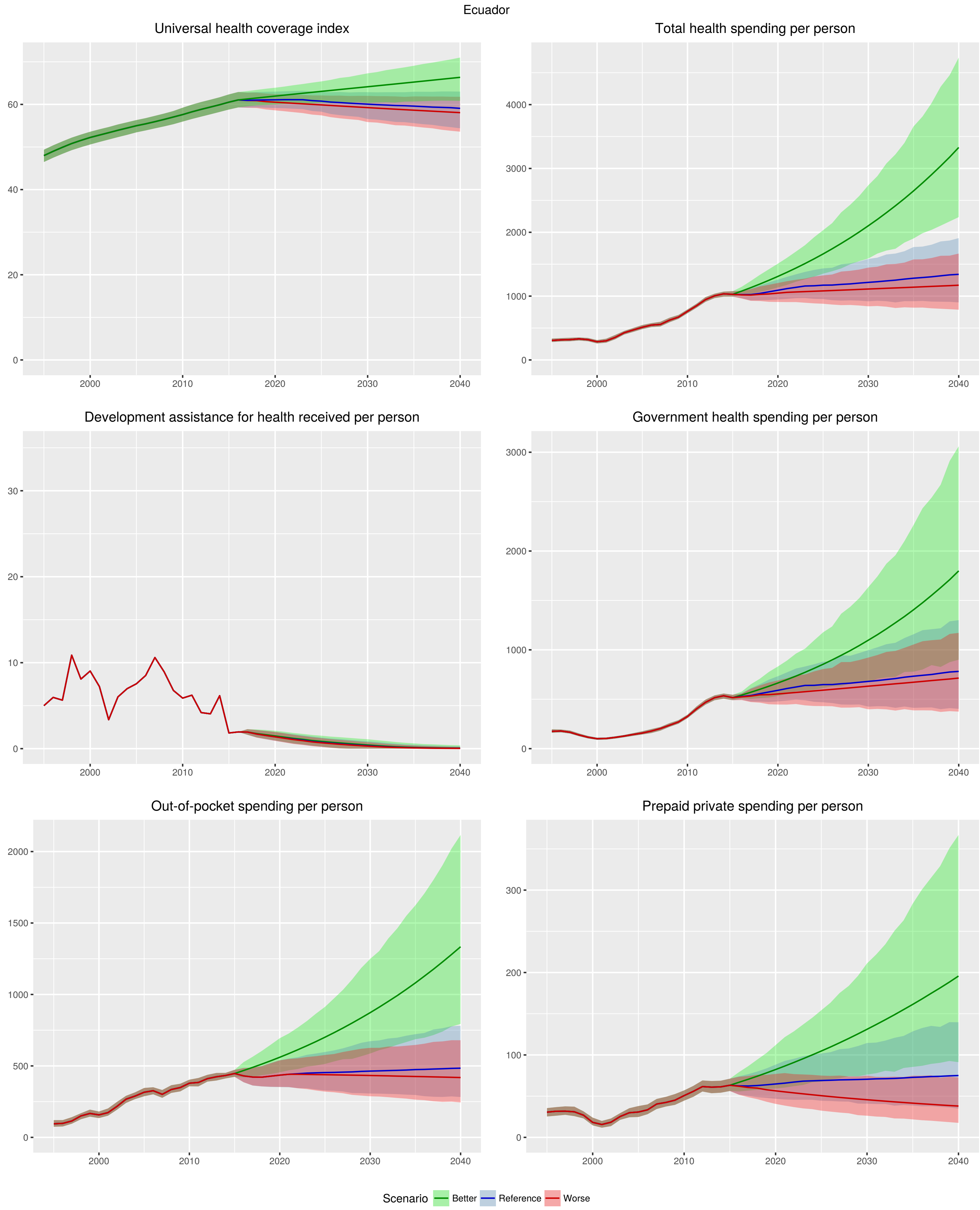
Ecuador
Universal health coverage index
Total health spending per person
Development assistance for health received per person
Government health spending per person
Out-of-pocket spending per person
Prepaid private spending per person
0
20
40
60
1000
2000
3000
4000
10
30
500
1500
100
200
300
2000
2010
2020
2030
2040
Scenario
Better
Reference
Worse

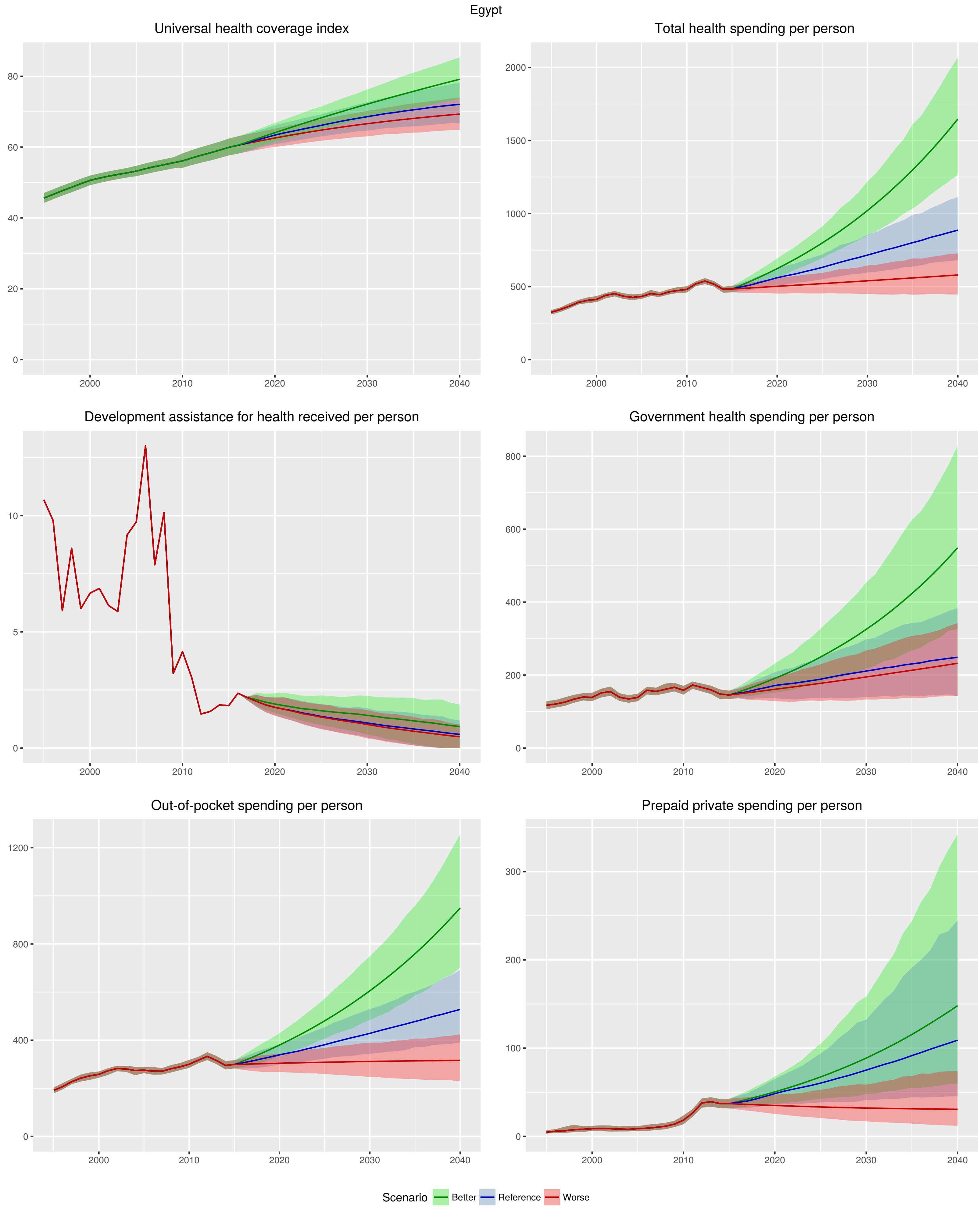

Egypt
Universal health coverage index
0
20
40
60
80
2000
2010
2020
2030
2040
Total health spending per person
0
500
1000
1500
2000
2000
2010
2020
2030
2040
Development assistance for health received per person
0
5
10
2000
2010
2020
2030
2040
Government health spending per person
0
200
400
600
800
2000
2010
2020
2030
2040
Out-of-pocket spending per person
0
400
800
1200
2000
2010
2020
2030
2040
Prepaid private spending per person
0
100
200
300
2000
2010
2020
2030
2040
Scenario
Better
Reference
Worse

El Salvador

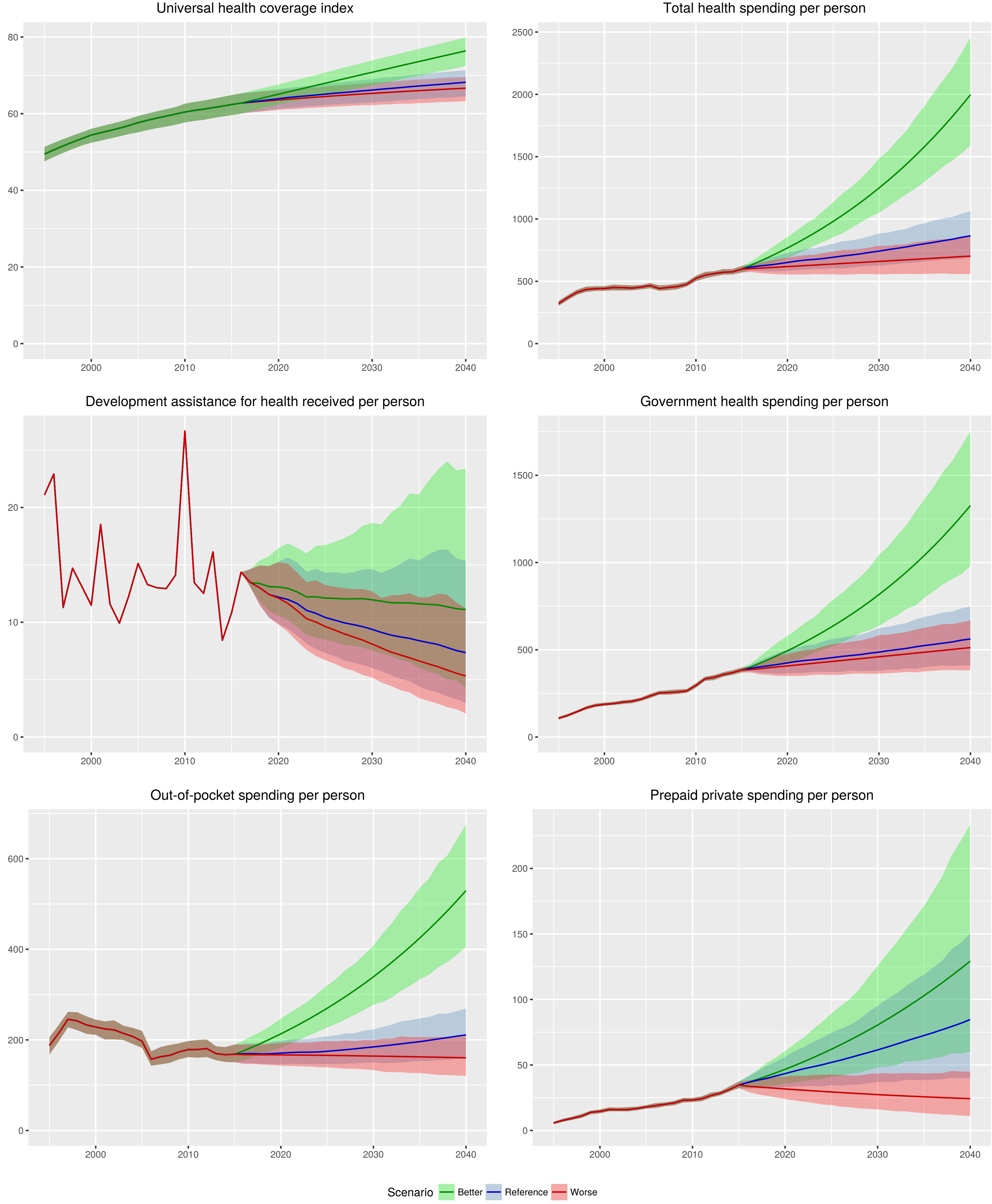

Universal health coverage index
Total health spending per person
Development assistance for health received per person
Government health spending per person
Out-of-pocket spending per person
Prepaid private spending per person
Scenario
Better
Reference
Worse

Equatorial Guinea

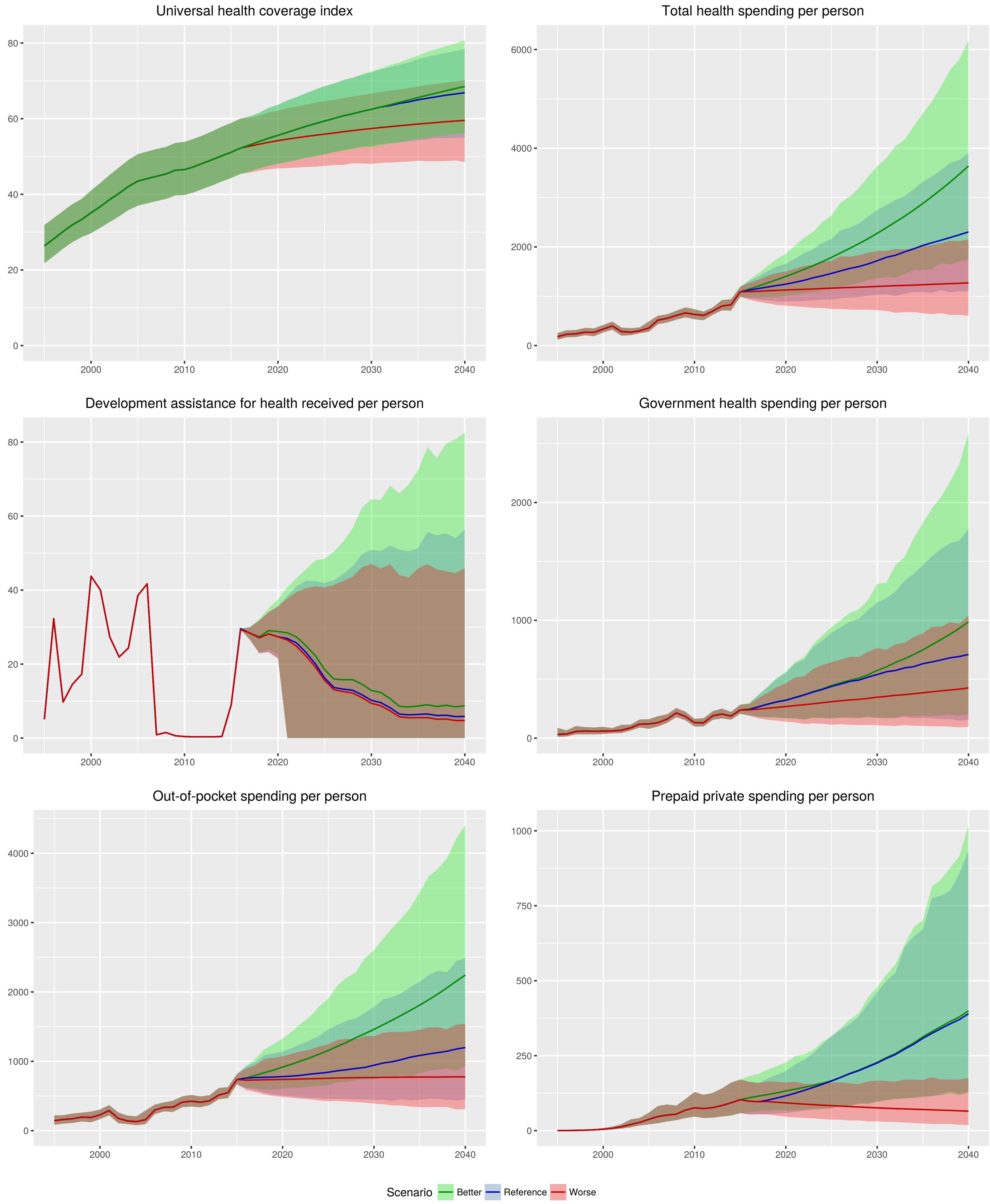

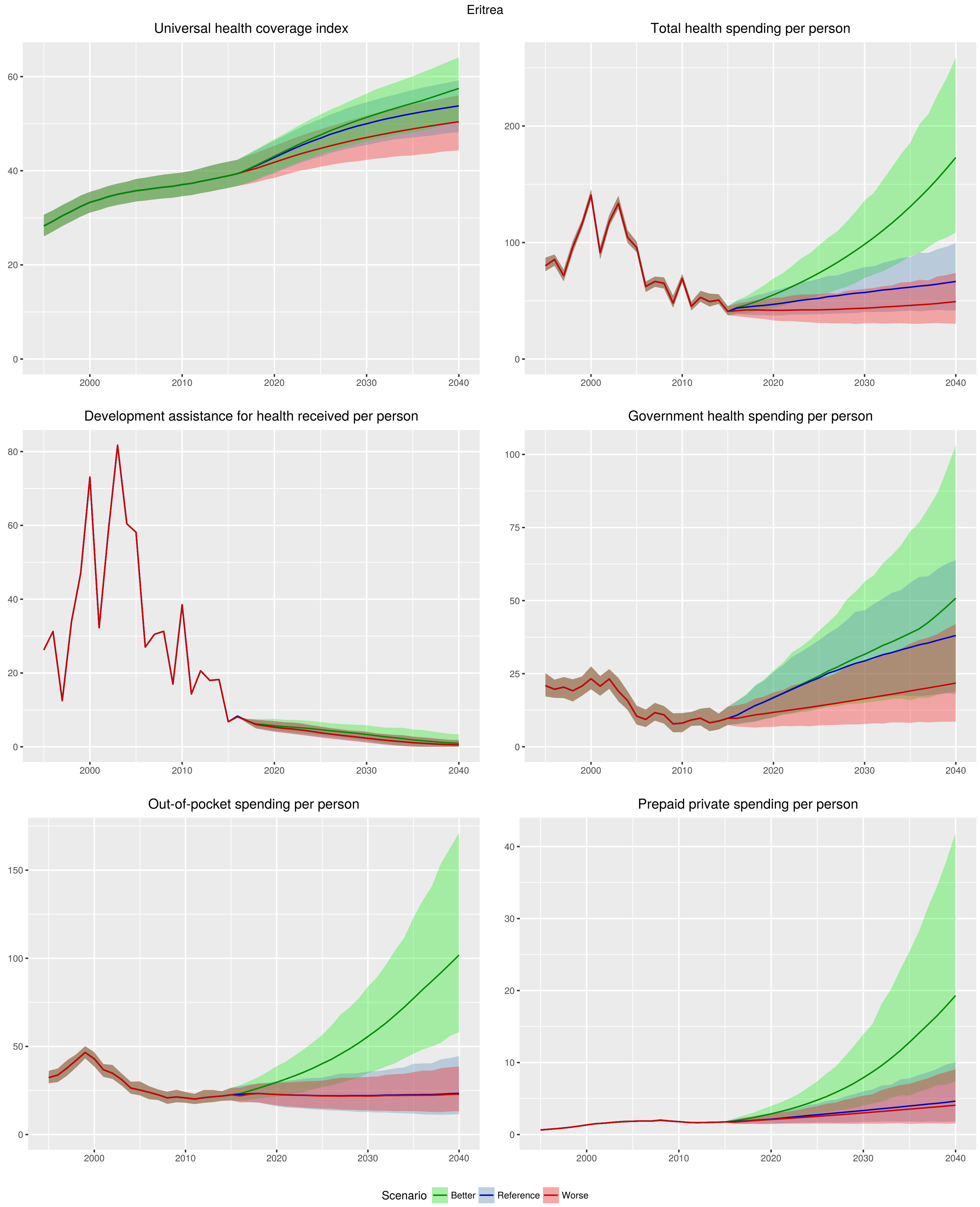

Eritrea
Universal health coverage index
Total health spending per person
Development assistance for health received per person
Government health spending per person
Out-of-pocket spending per person
Prepaid private spending per person
Scenario
Better
Reference
Worse

Estonia

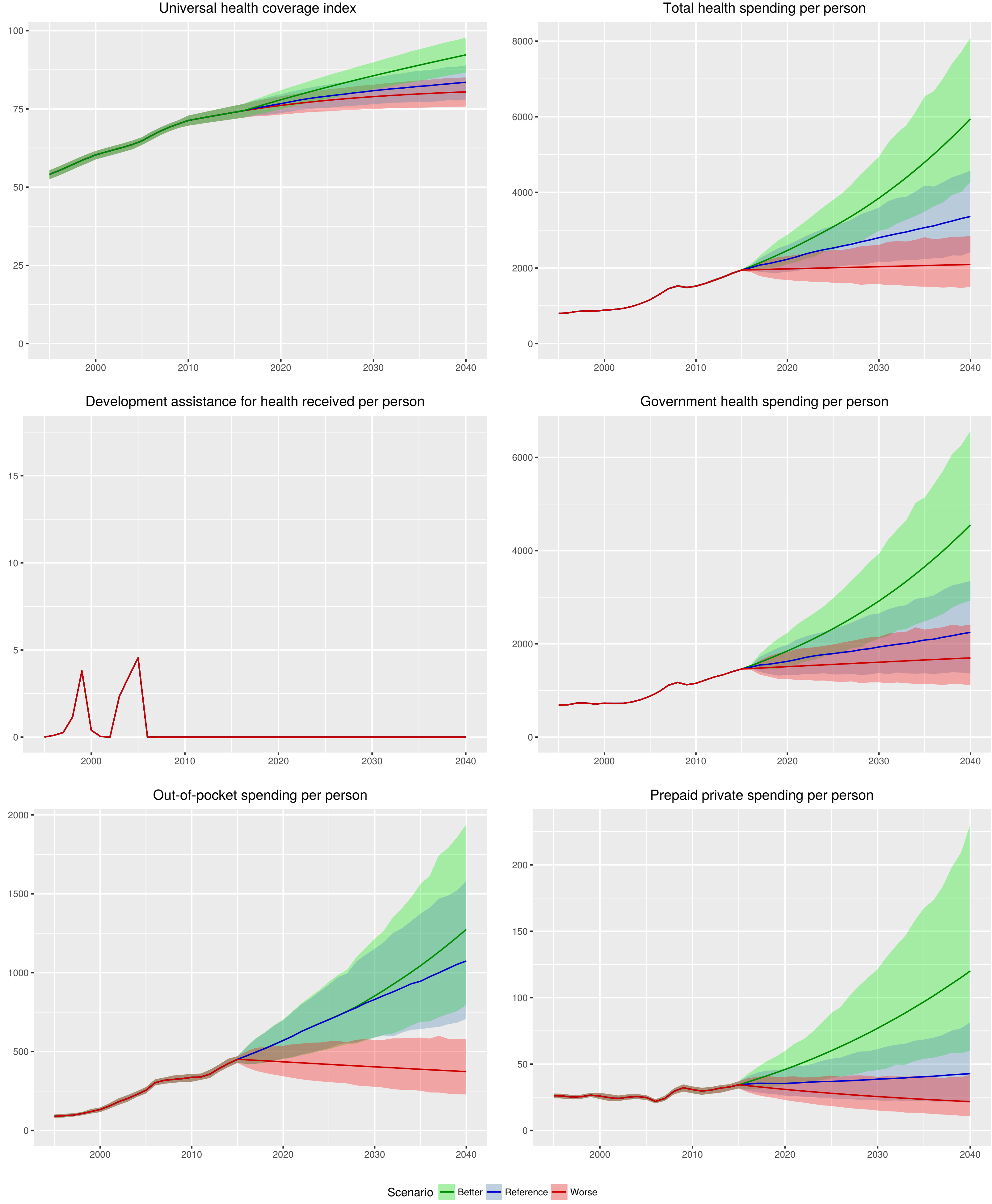

Universal health coverage index
Total health spending per person
Development assistance for health received per person
Government health spending per person
Out-of-pocket spending per person
Prepaid private spending per person
2000
2010
2020
2030
2040
Scenario
Better
Reference
Worse

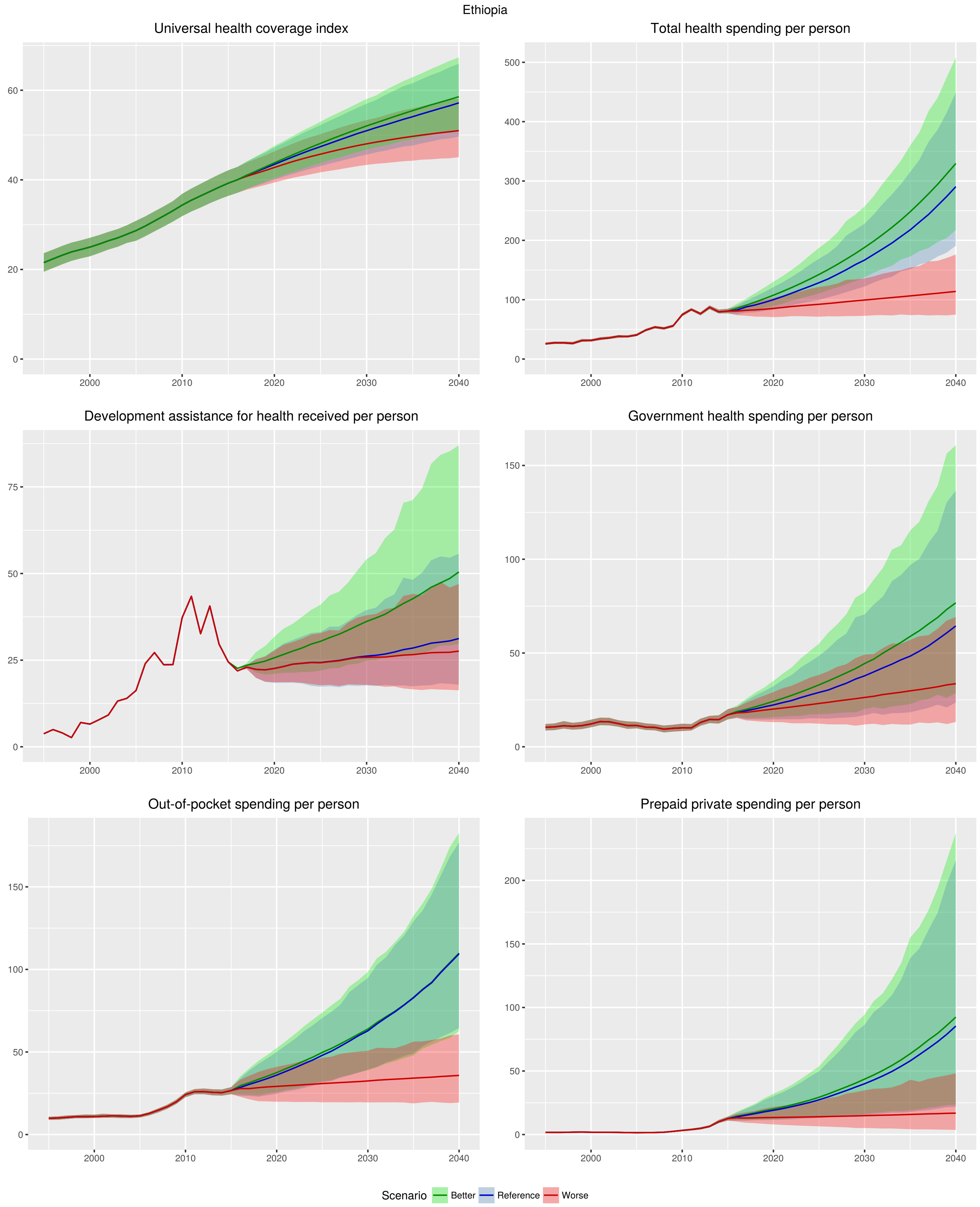

Ethiopia
Universal health coverage index
Total health spending per person
Development assistance for health received per person
Government health spending per person
Out-of-pocket spending per person
Prepaid private spending per person
Scenario
Better
Reference
Worse

Federated States of Micronesia

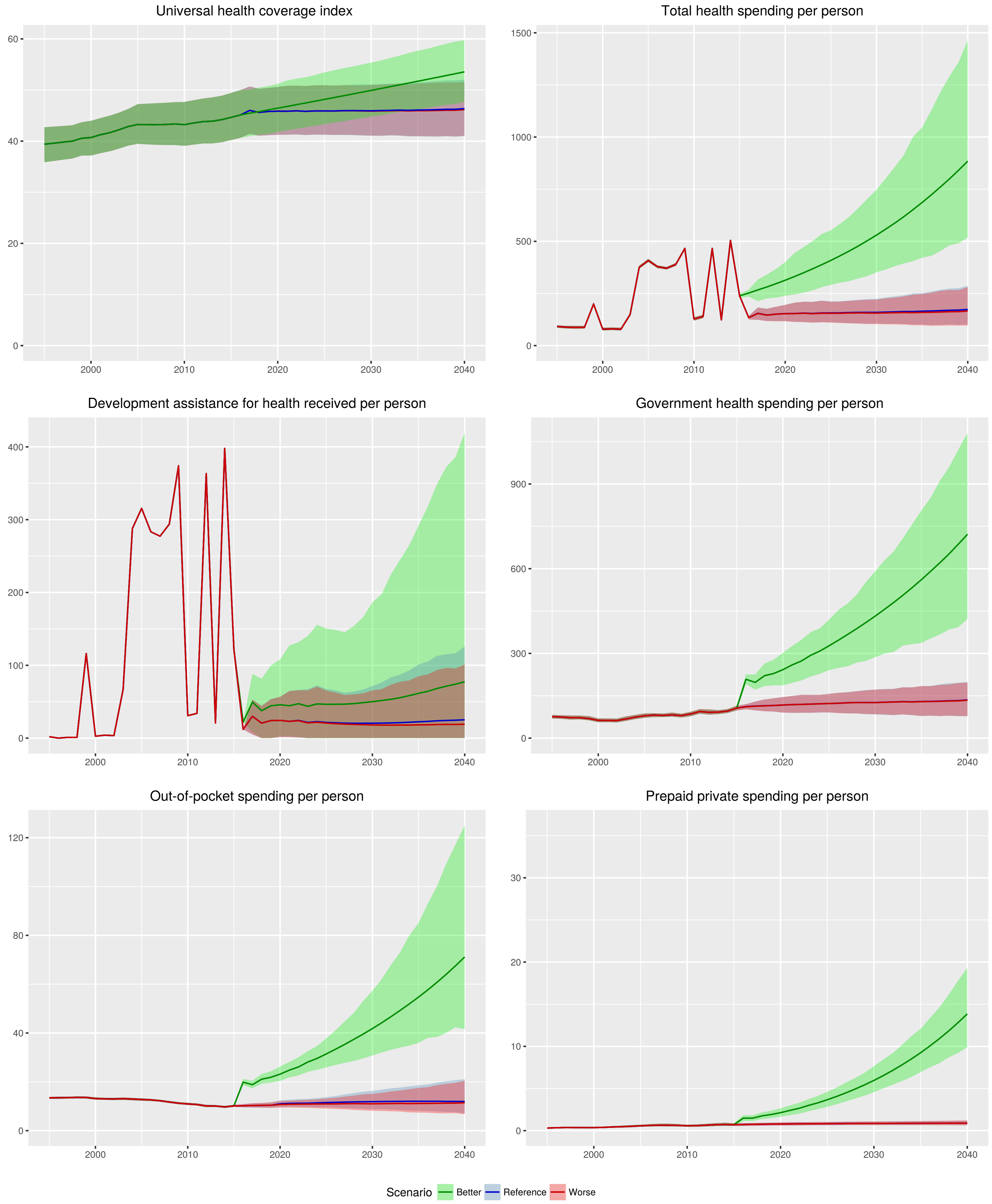

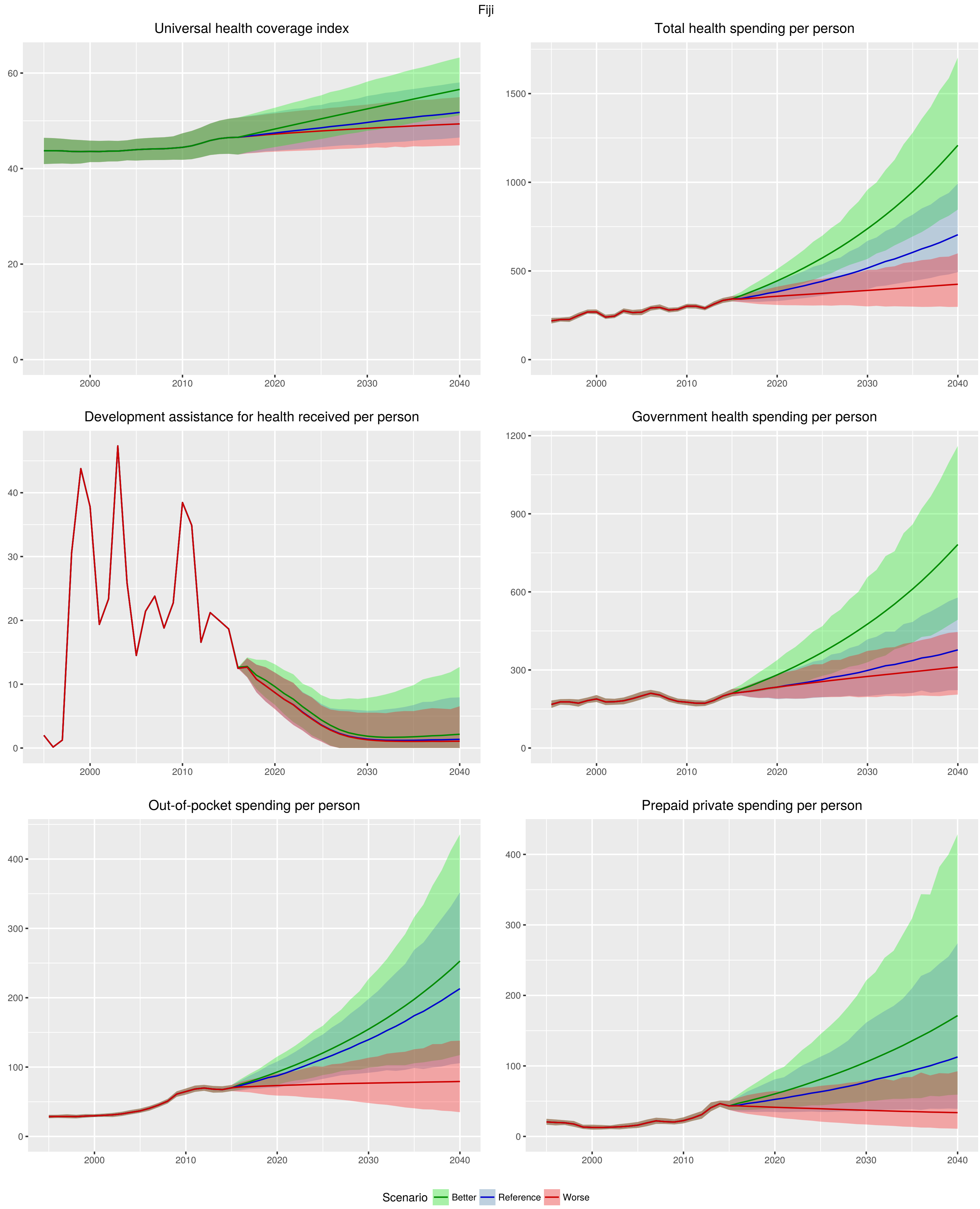
Fiji
Universal health coverage index
Total health spending per person
Development assistance for health received per person
Government health spending per person
Out-of-pocket spending per person
Prepaid private spending per person
Scenario
Better
Reference
Worse

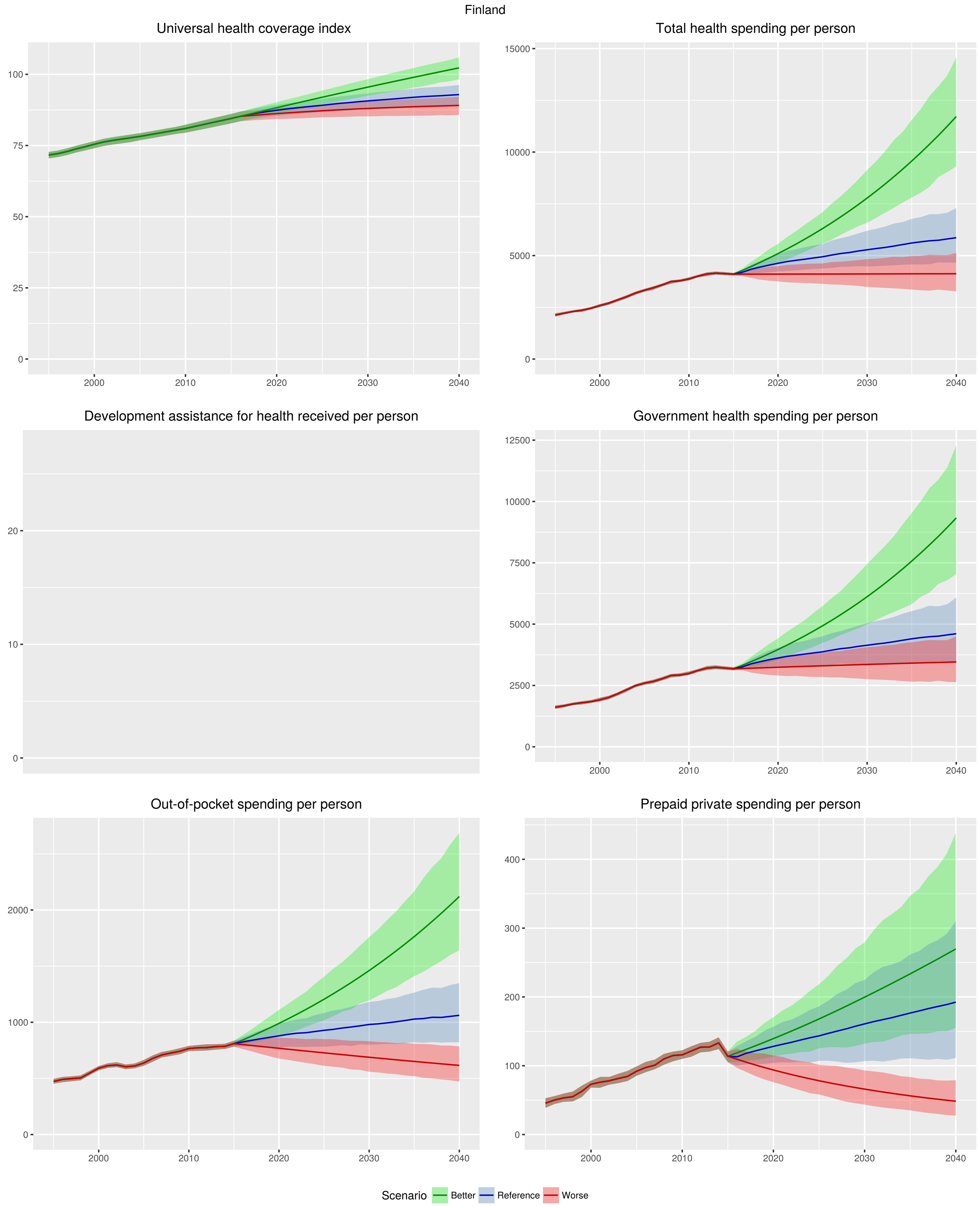

Finland
Universal health coverage index
Total health spending per person
Development assistance for health received per person
Government health spending per person
Out-of-pocket spending per person
Prepaid private spending per person
Scenario
Better
Reference
Worse

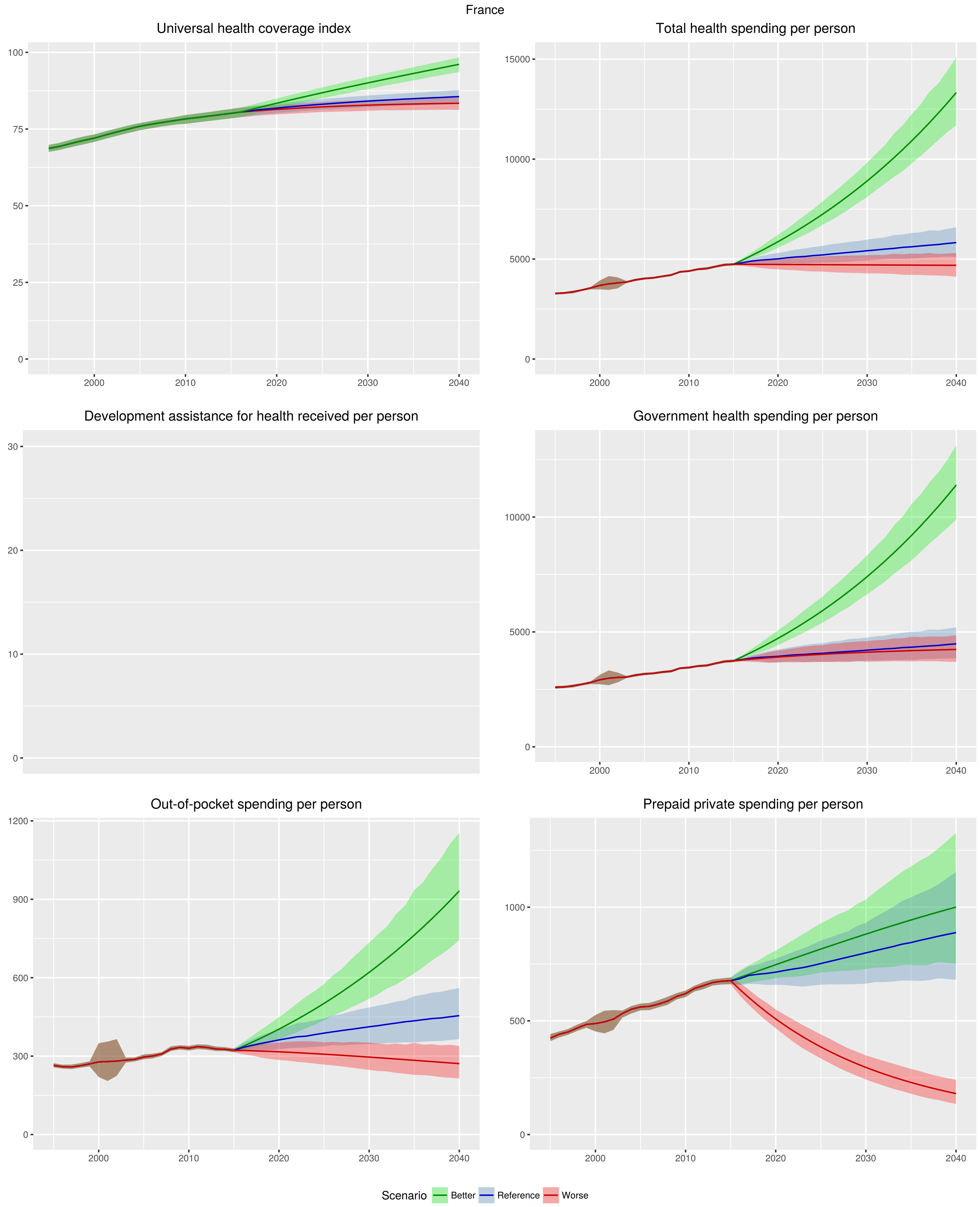

France
Universal health coverage index
Total health spending per person
Development assistance for health received per person
Government health spending per person
Out-of-pocket spending per person
Prepaid private spending per person
Scenario
Better
Reference
Worse

Gabon

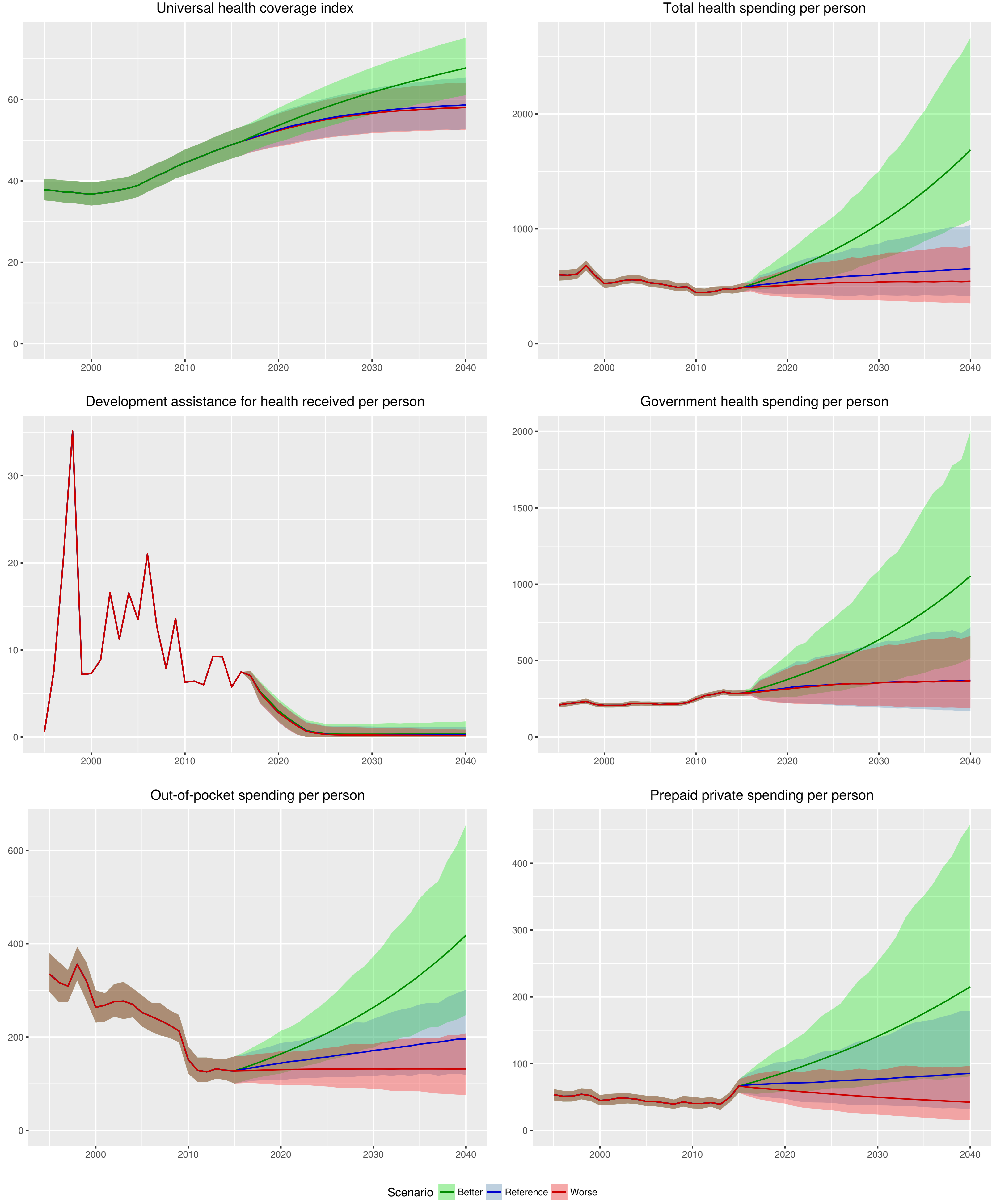

Universal health coverage index
0
20
40
60
2000
2010
2020
2030
2040
Total health spending per person
0
1000
2000
Development assistance for health received per person
0
10
20
30
Government health spending per person
0
500
1000
1500
2000
Out-of-pocket spending per person
0
200
400
600
Prepaid private spending per person
0
100
200
300
400
Scenario
Better
Reference
Worse

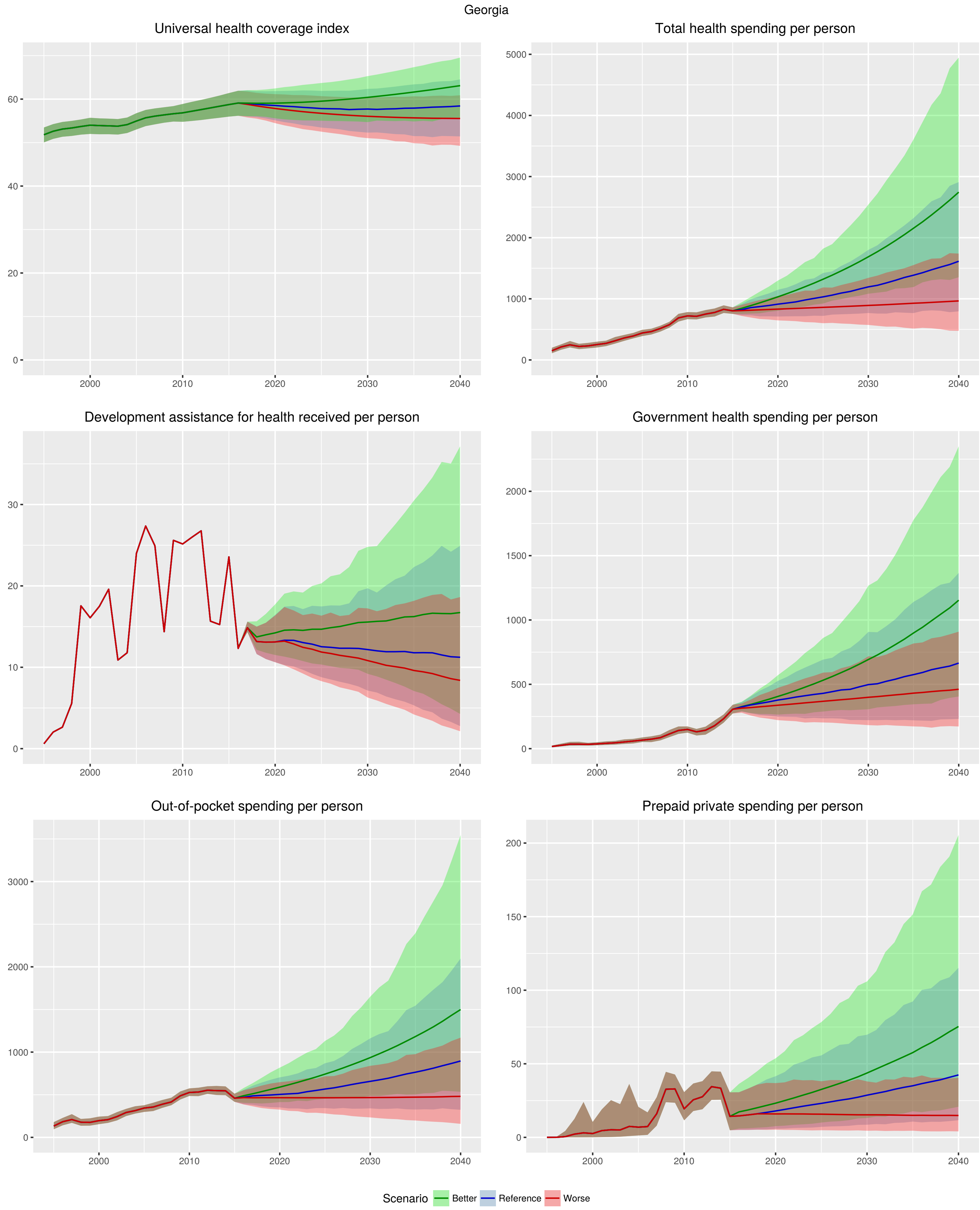

Georgia
Universal health coverage index
Total health spending per person
Development assistance for health received per person
Government health spending per person
Out-of-pocket spending per person
Prepaid private spending per person
0
20
40
60
1000
2000
3000
4000
5000
10
30
500
1500
50
100
150
200
2000
2010
2020
2030
2040
Scenario
Better
Reference
Worse

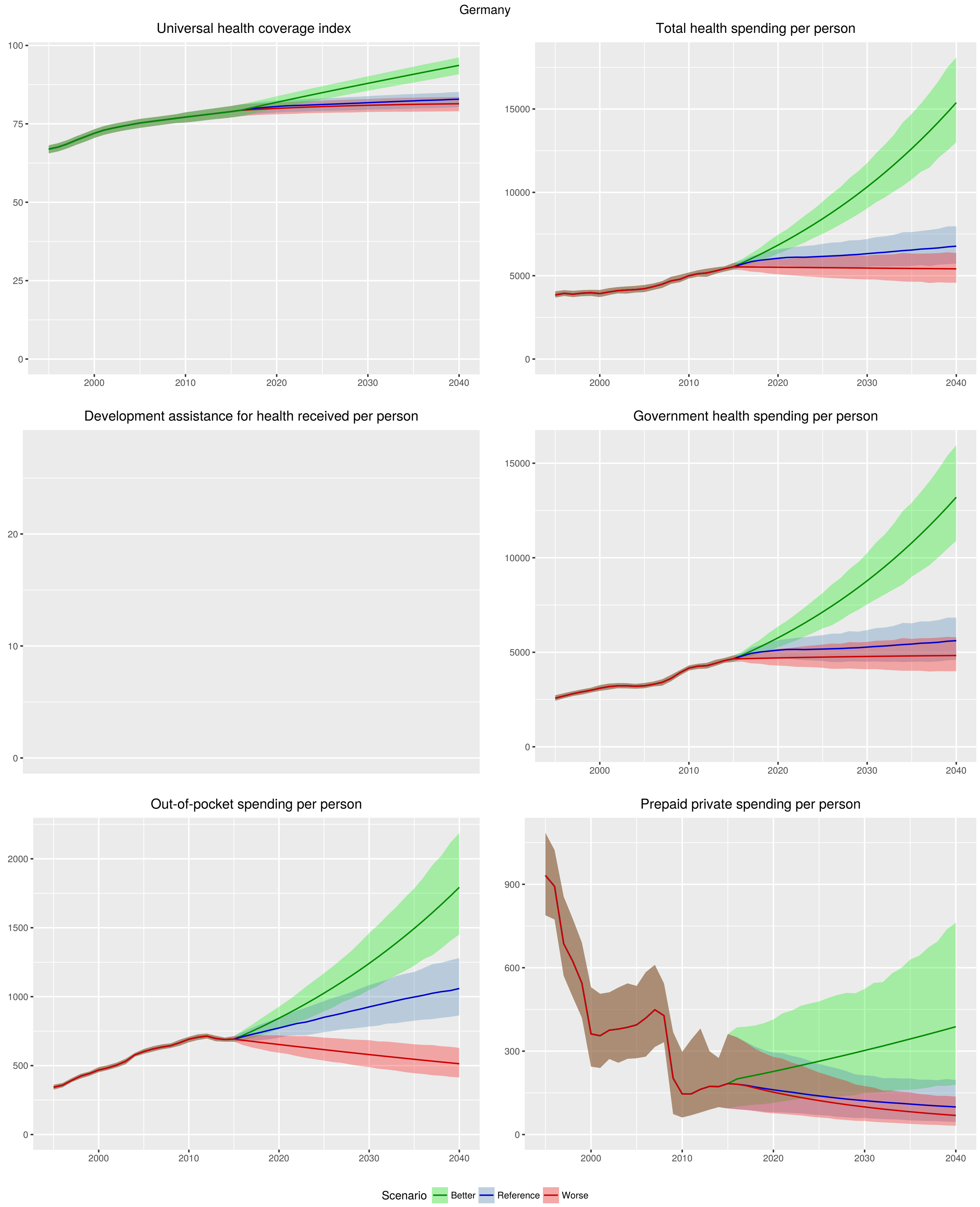
Germany
Universal health coverage index
Total health spending per person
Development assistance for health received per person
Government health spending per person
Out-of-pocket spending per person
Prepaid private spending per person
Scenario
Better
Reference
Worse

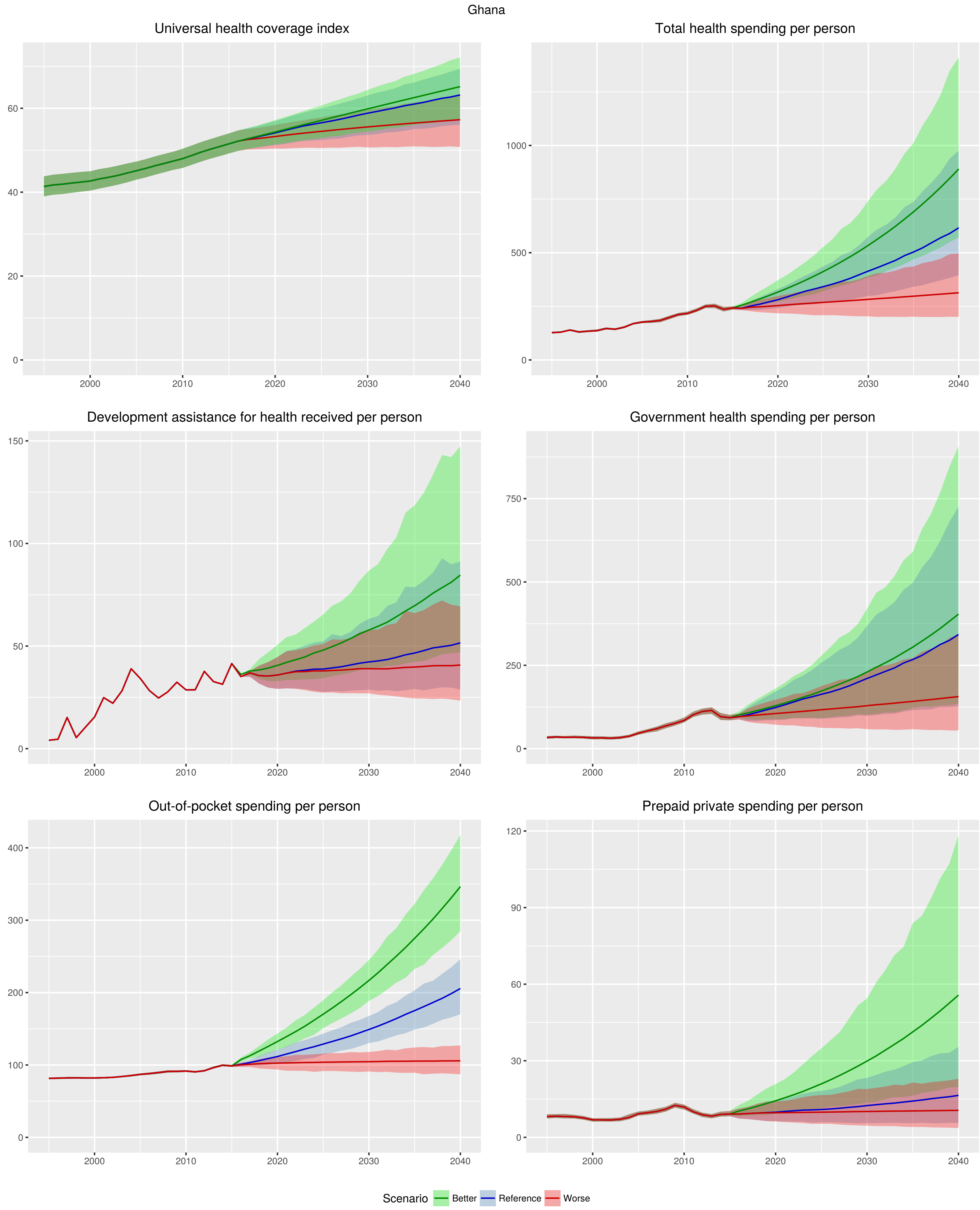

Ghana
Universal health coverage index
Total health spending per person
Development assistance for health received per person
Government health spending per person
Out-of-pocket spending per person
Prepaid private spending per person
Scenario
Better
Reference
Worse

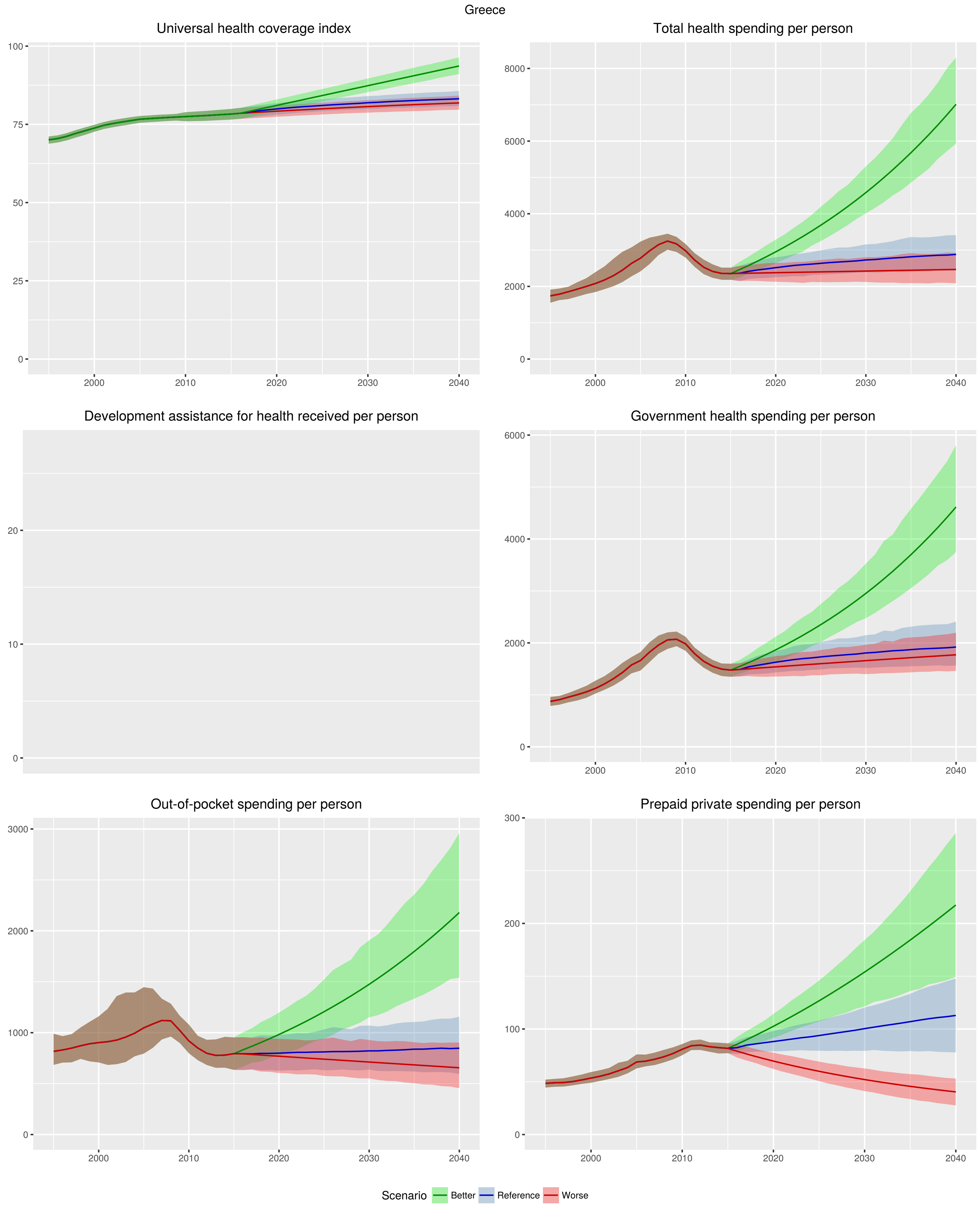

Greece
Universal health coverage index
Total health spending per person
Development assistance for health received per person
Government health spending per person
Out-of-pocket spending per person
Prepaid private spending per person
Scenario
Better
Reference
Worse

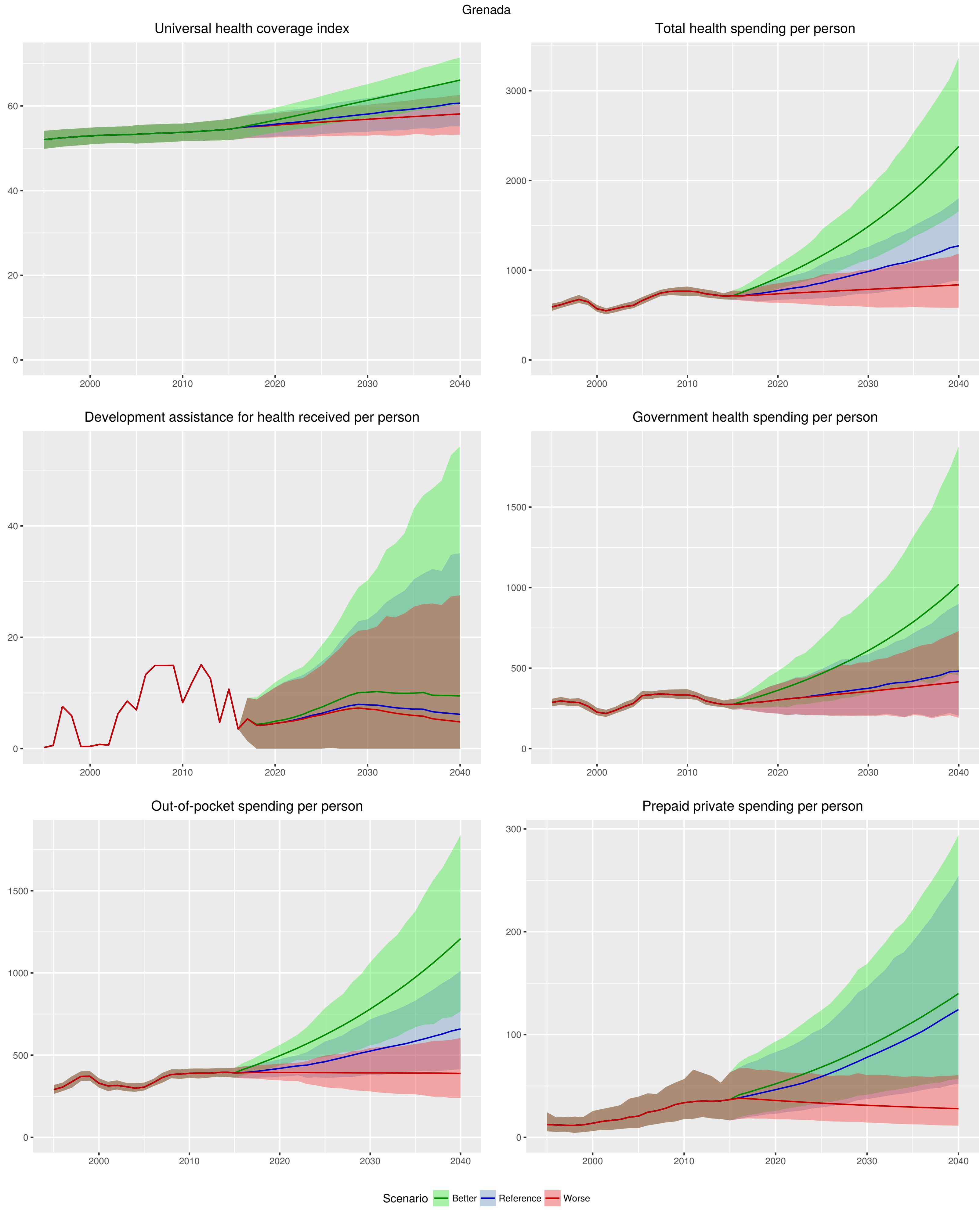

Grenada
Universal health coverage index
Total health spending per person
Development assistance for health received per person
Government health spending per person
Out-of-pocket spending per person
Prepaid private spending per person
Scenario
Better
Reference
Worse

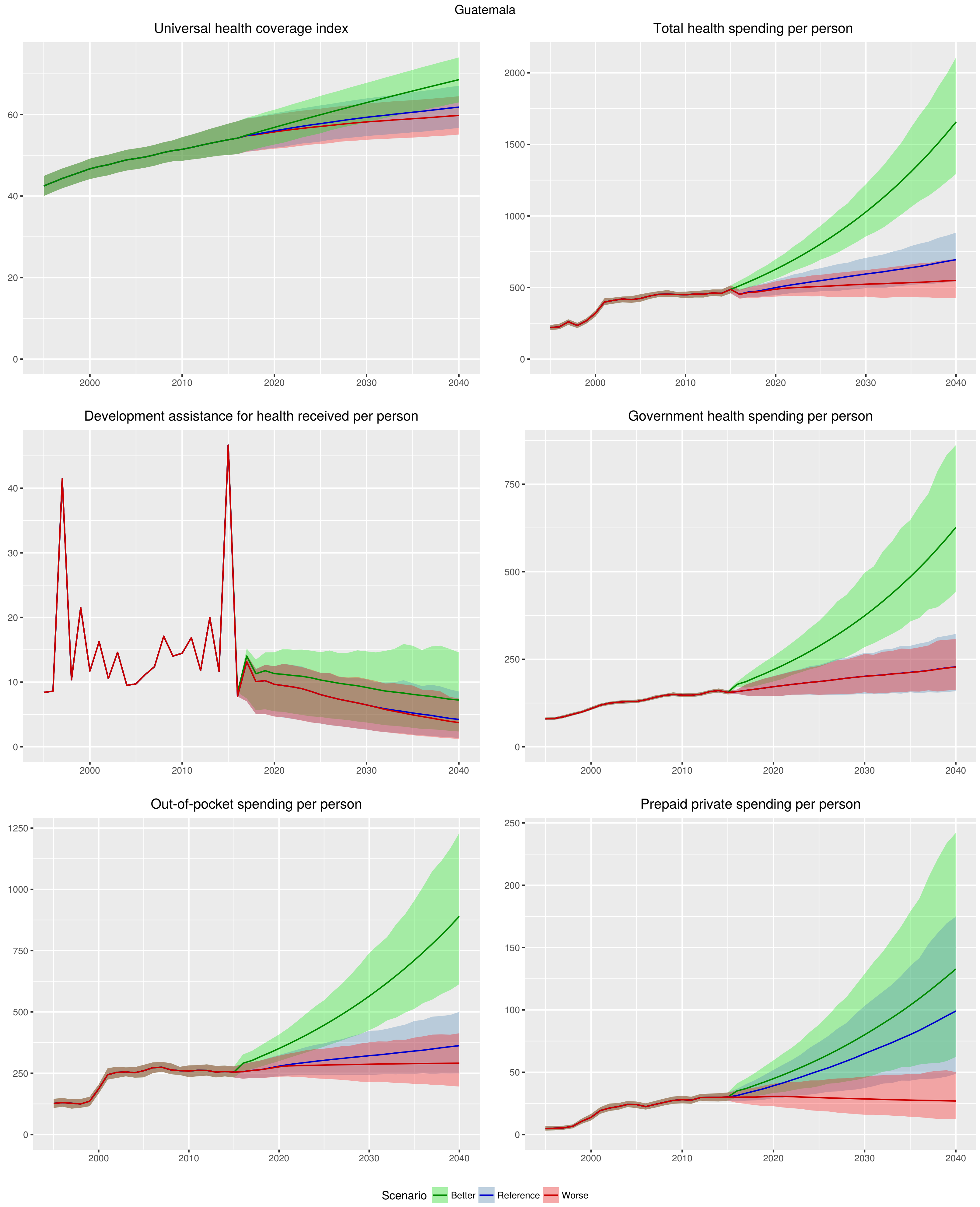

Guatemala
Universal health coverage index
Total health spending per person
Development assistance for health received per person
Government health spending per person
Out-of-pocket spending per person
Prepaid private spending per person
0
20
40
60
0
500
1000
1500
2000
0
10
20
30
40
0
250
500
750
0
250
500
750
1000
1250
0
50
100
150
200
250
2000
2010
2020
2030
2040
Scenario
Better
Reference
Worse

Guinea

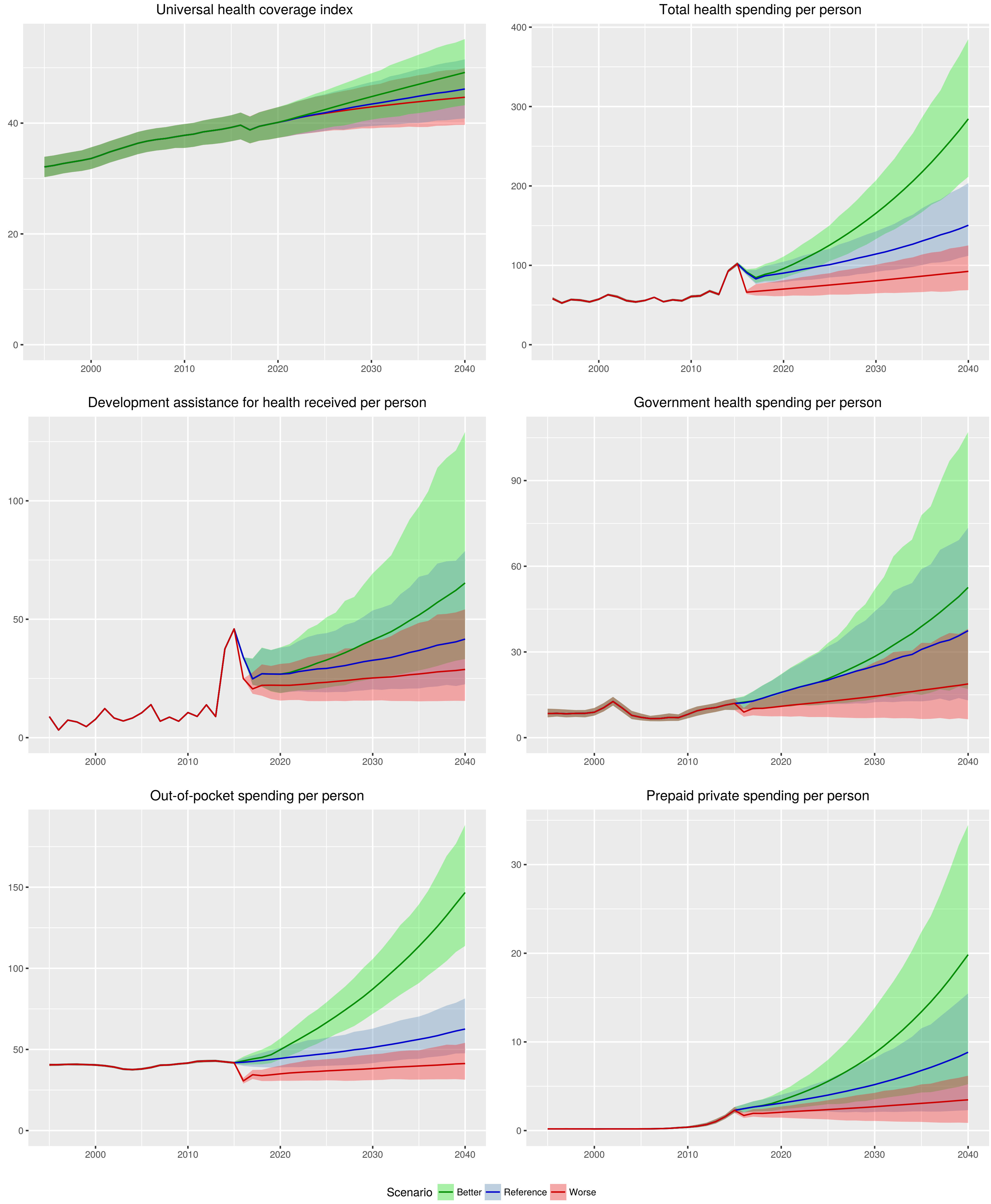
Universal health coverage index
Total health spending per person
Development assistance for health received per person
Government health spending per person
Out-of-pocket spending per person
Prepaid private spending per person
2000
2010
2020
2030
2040
Scenario
Better
Reference
Worse

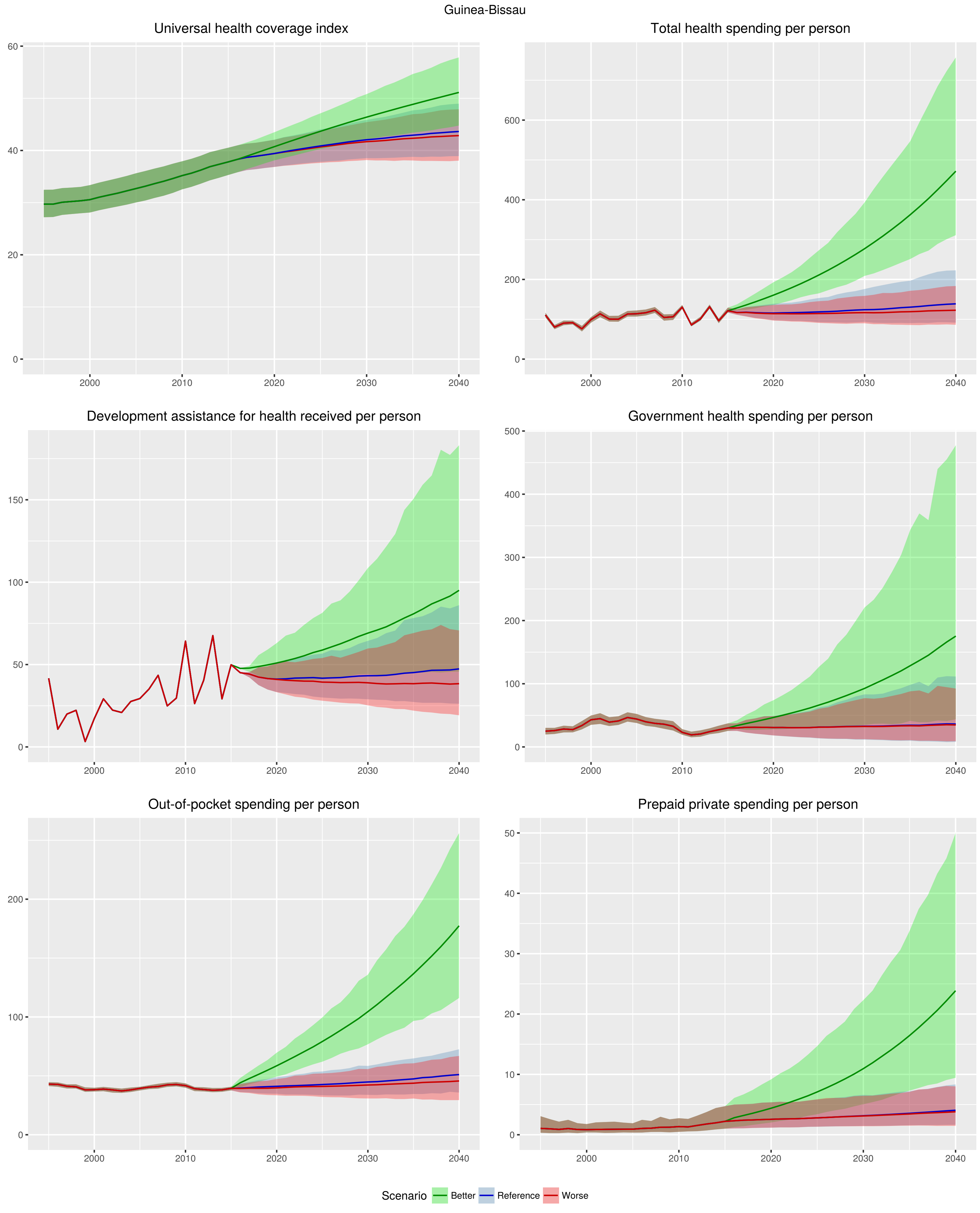
Guinea-Bissau
Universal health coverage index
Total health spending per person
Development assistance for health received per person
Government health spending per person
Out-of-pocket spending per person
Prepaid private spending per person
Scenario
Better
Reference
Worse

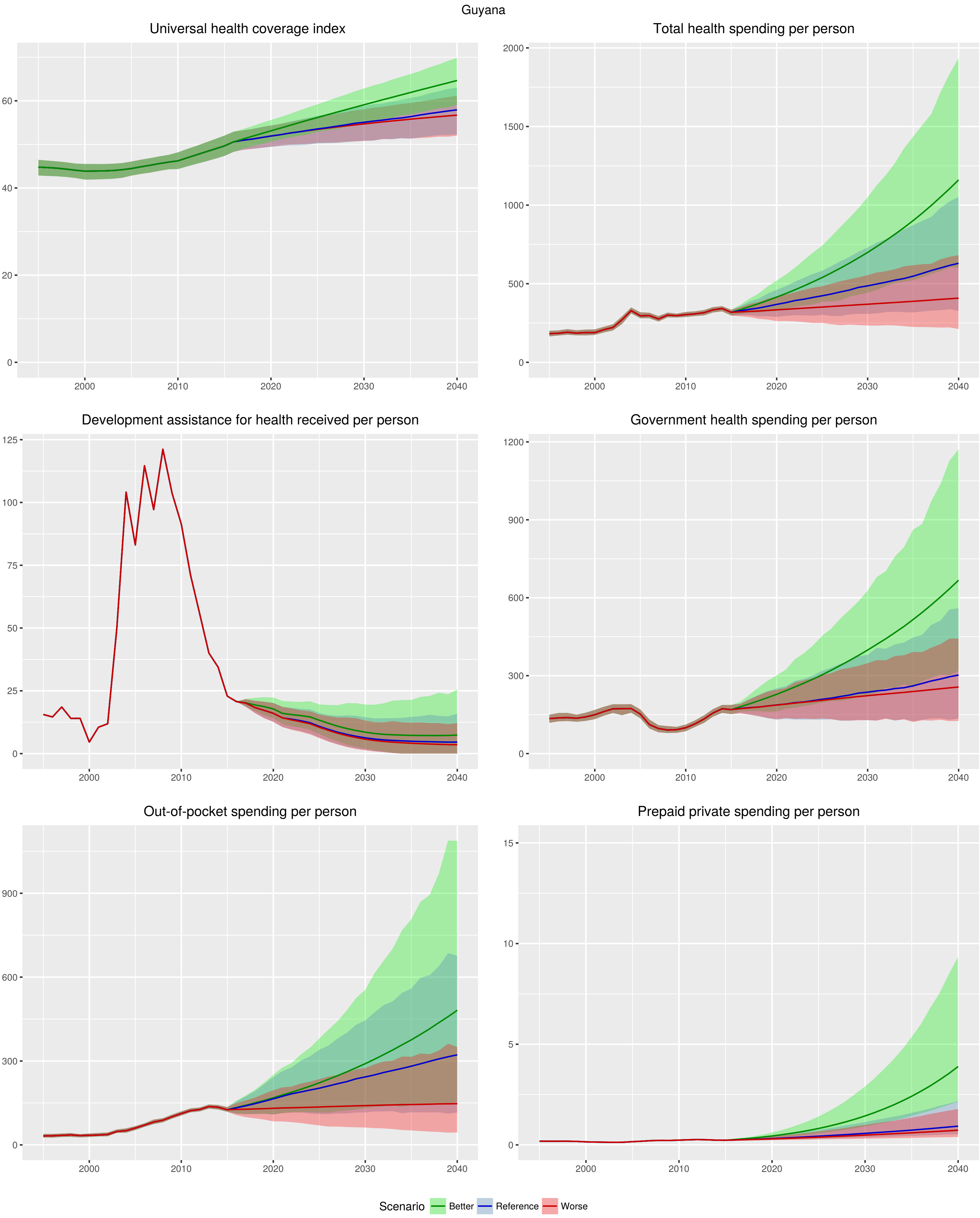

Guyana
Universal health coverage index
Total health spending per person
Development assistance for health received per person
Government health spending per person
Out-of-pocket spending per person
Prepaid private spending per person
Scenario
Better
Reference
Worse

Haiti

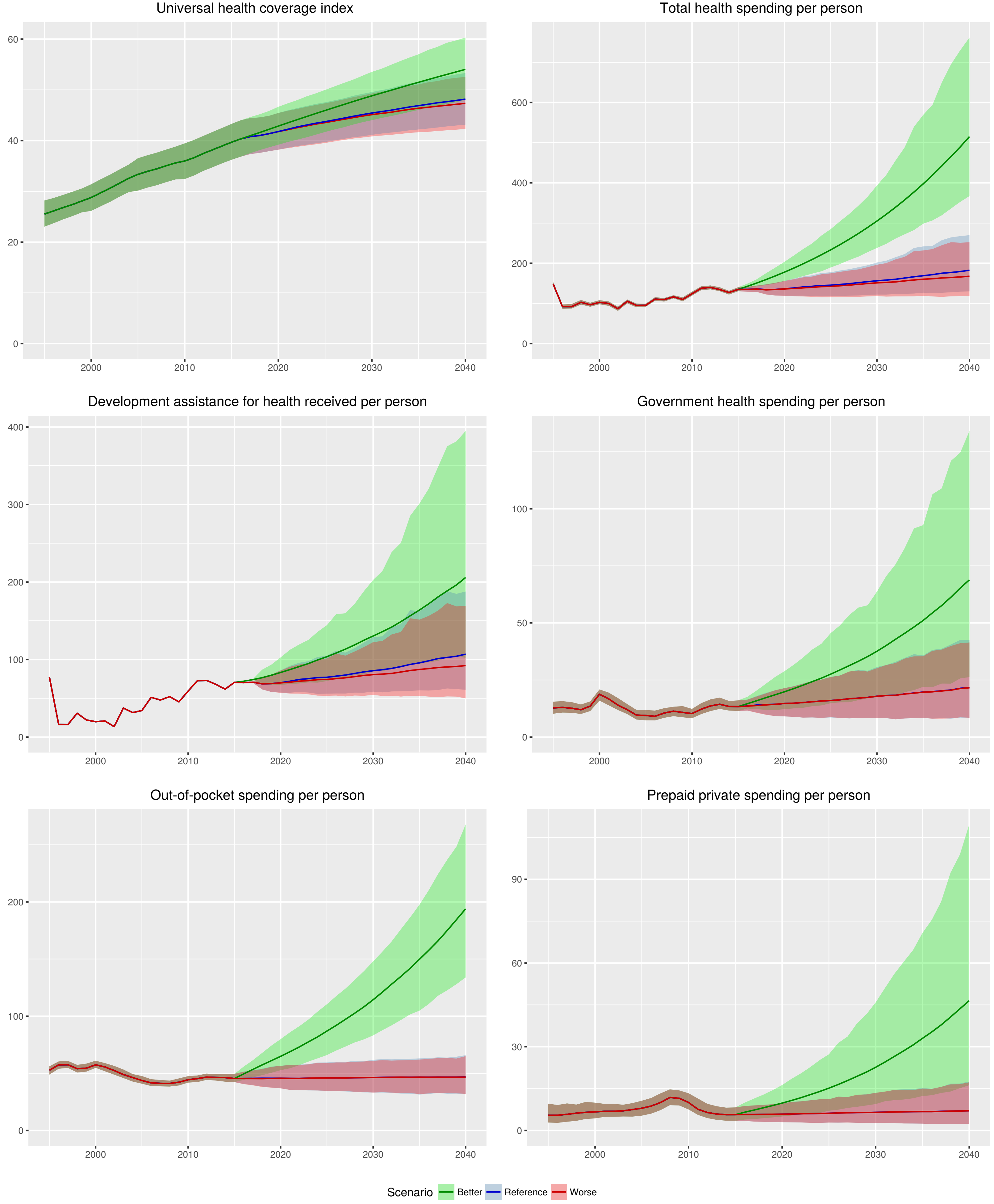

Universal health coverage index
0
20
40
60
2000
2010
2020
2030
2040
Total health spending per person
0
200
400
600
2000
2010
2020
2030
2040
Development assistance for health received per person
0
100
200
300
400
2000
2010
2020
2030
2040
Government health spending per person
0
50
100
2000
2010
2020
2030
2040
Out-of-pocket spending per person
0
100
200
2000
2010
2020
2030
2040
Prepaid private spending per person
0
30
60
90
2000
2010
2020
2030
2040
Scenario
Better
Reference
Worse

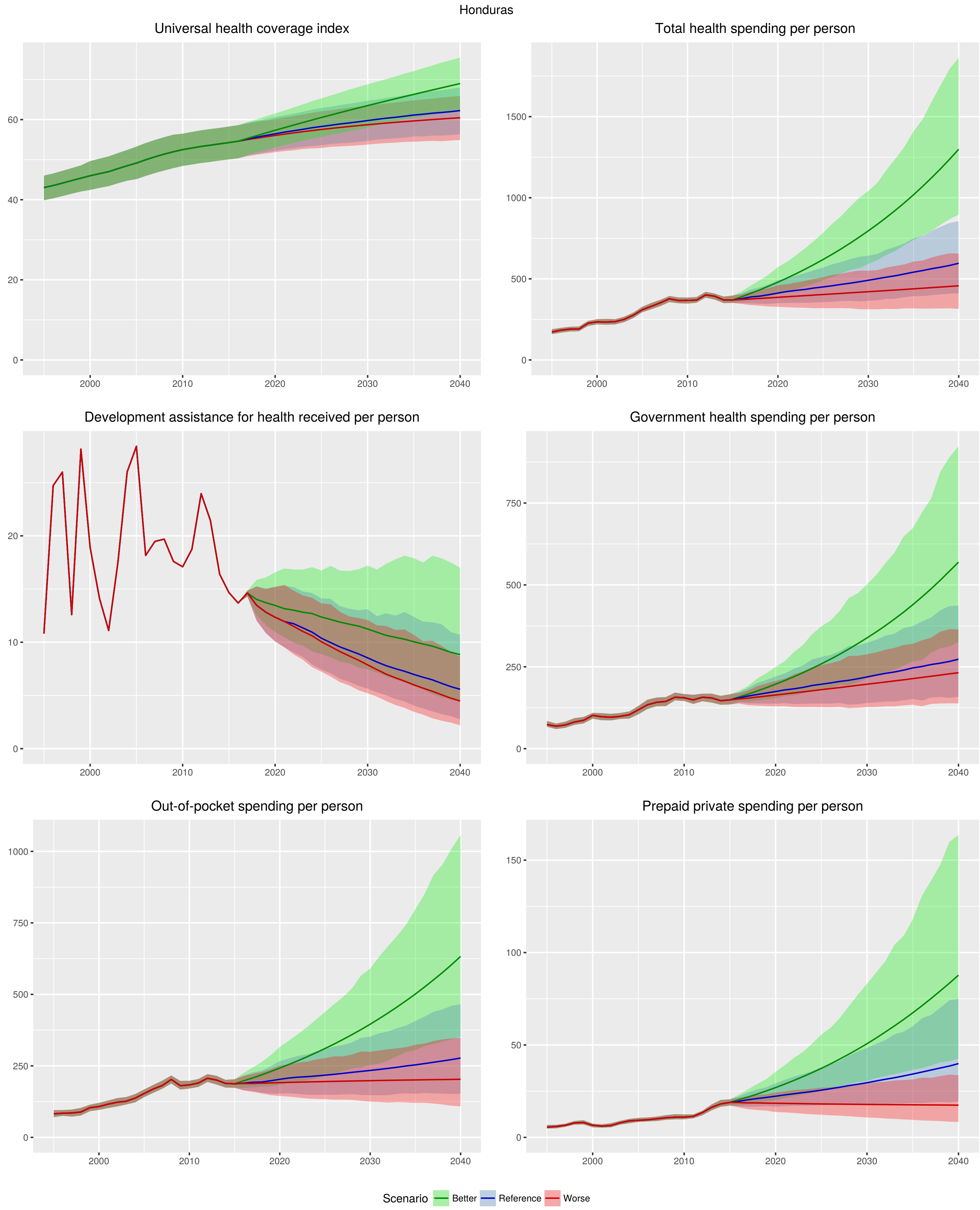

Honduras
Universal health coverage index
Total health spending per person
Development assistance for health received per person
Government health spending per person
Out-of-pocket spending per person
Prepaid private spending per person
0
20
40
60
500
1000
1500
10
250
750
50
100
150
2000
2010
2020
2030
2040
Scenario
Better
Reference
Worse

Hungary

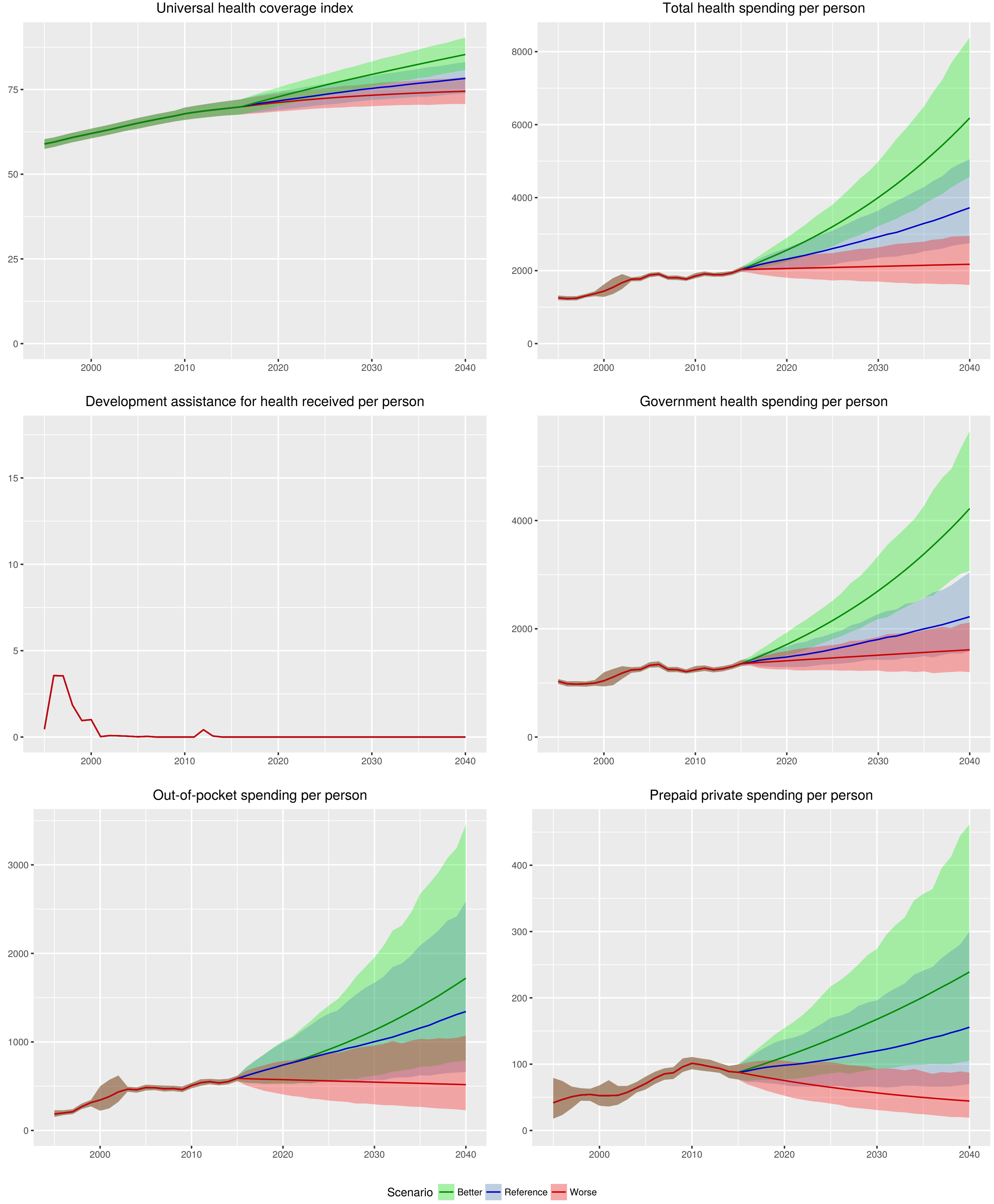

Universal health coverage index
0
25
50
75
2000
2010
2020
2030
2040
Total health spending per person
0
2000
4000
6000
8000
2000
2010
2020
2030
2040
Development assistance for health received per person
0
5
10
15
2000
2010
2020
2030
2040
Government health spending per person
0
2000
4000
2000
2010
2020
2030
2040
Out-of-pocket spending per person
0
1000
2000
3000
2000
2010
2020
2030
2040
Prepaid private spending per person
0
100
200
300
400
2000
2010
2020
2030
2040
Scenario
Better
Reference
Worse

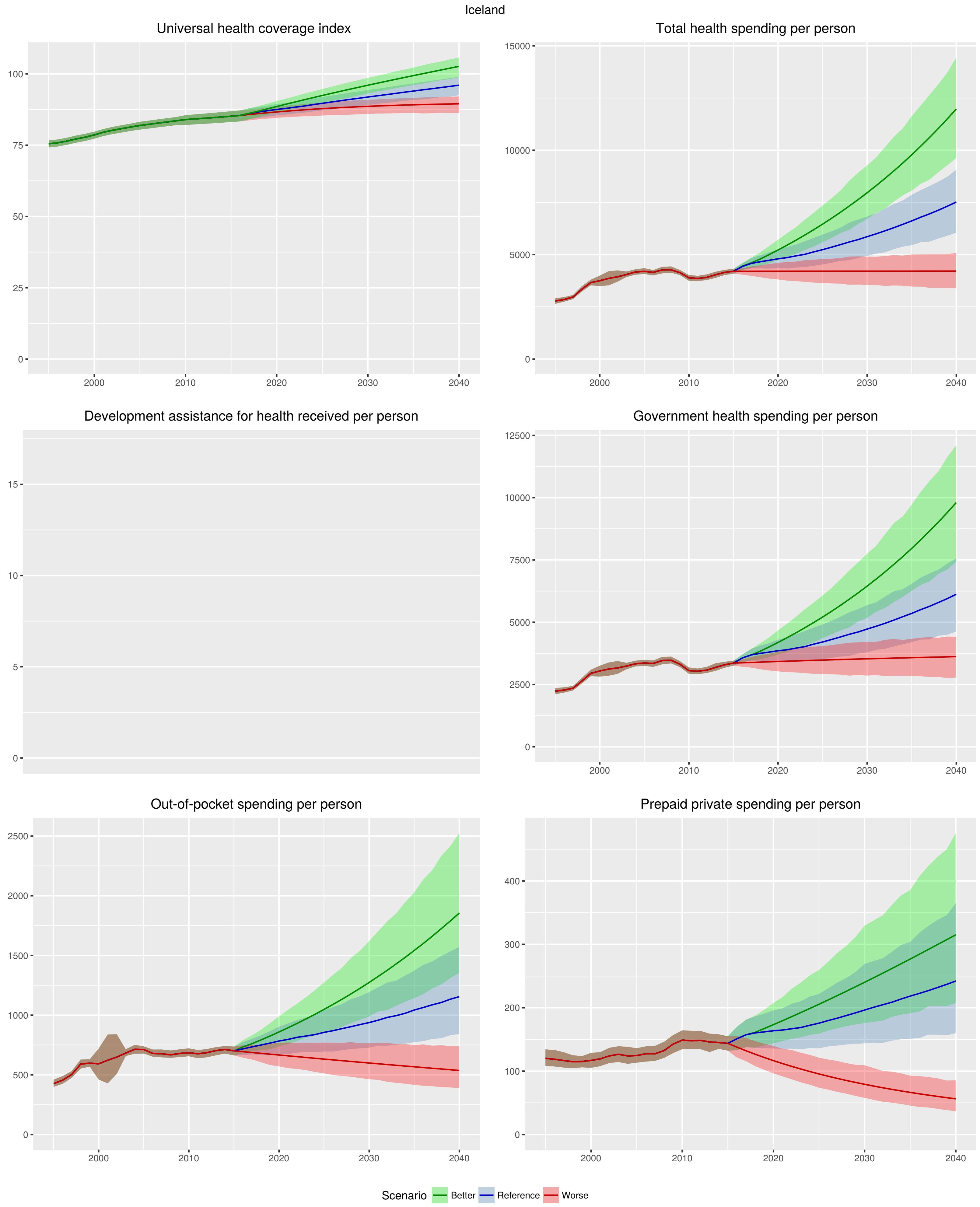
Iceland
Universal health coverage index
Total health spending per person
Development assistance for health received per person
Government health spending per person
Out-of-pocket spending per person
Prepaid private spending per person
Scenario
Better
Reference
Worse

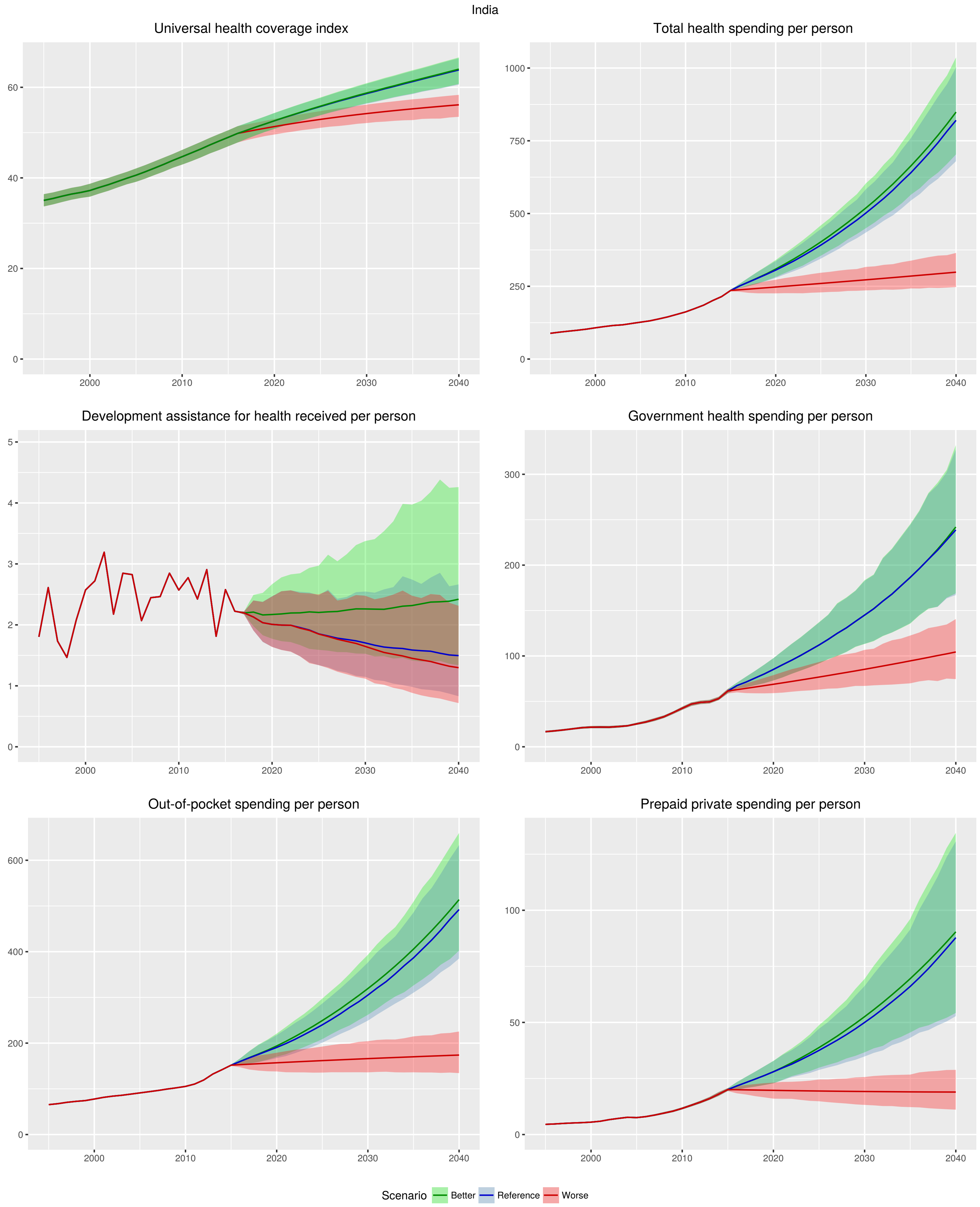
India
Universal health coverage index
Total health spending per person
Development assistance for health received per person
Government health spending per person
Out-of-pocket spending per person
Prepaid private spending per person
2000
2010
2020
2030
2040
0
20
40
60
250
500
750
1000
1
2
3
4
5
100
200
300
400
600
50
Scenario
Better
Reference
Worse

Indonesia

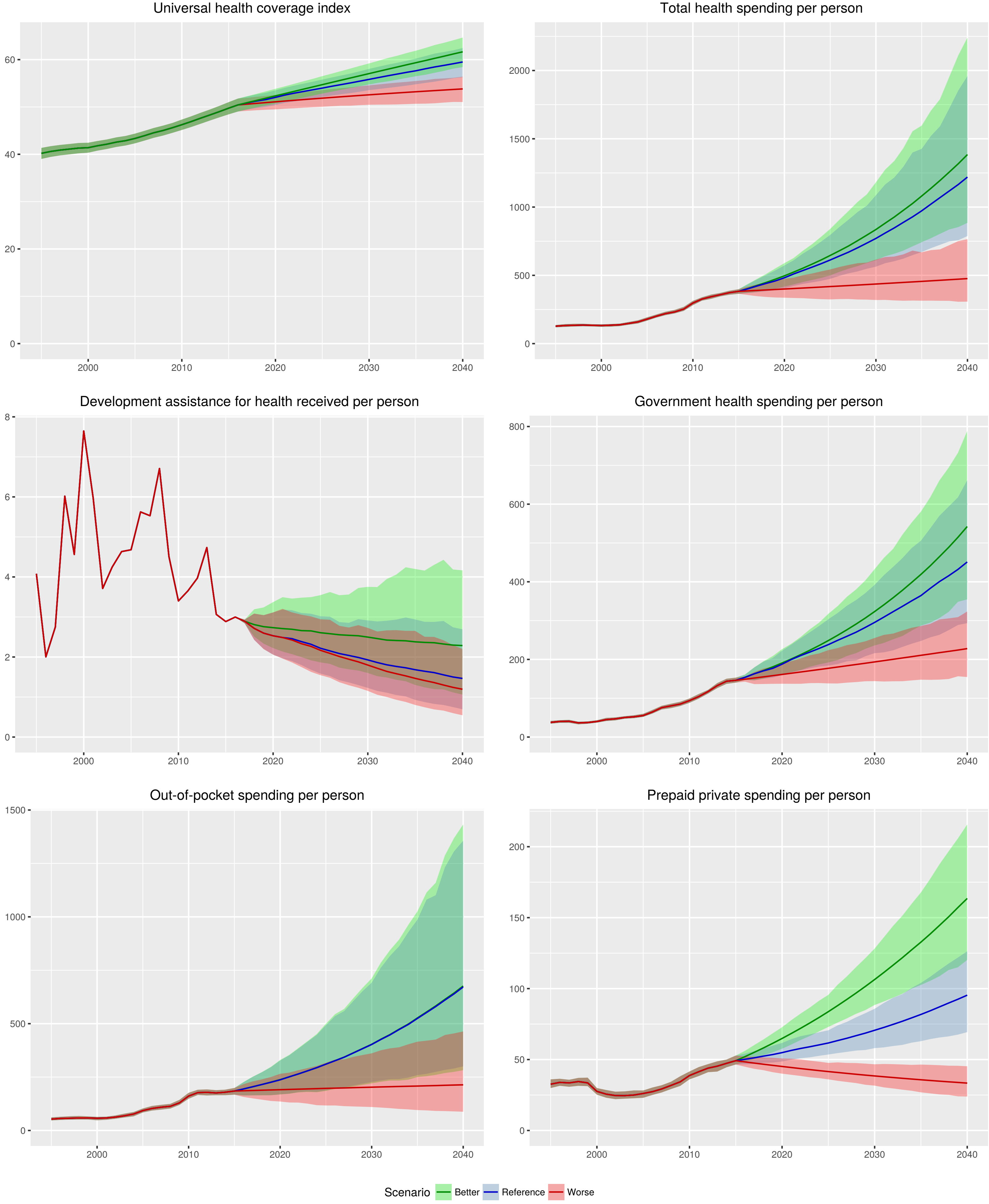

Universal health coverage index
Total health spending per person
Development assistance for health received per person
Government health spending per person
Out-of-pocket spending per person
Prepaid private spending per person
Scenario
Better
Reference
Worse

Iran

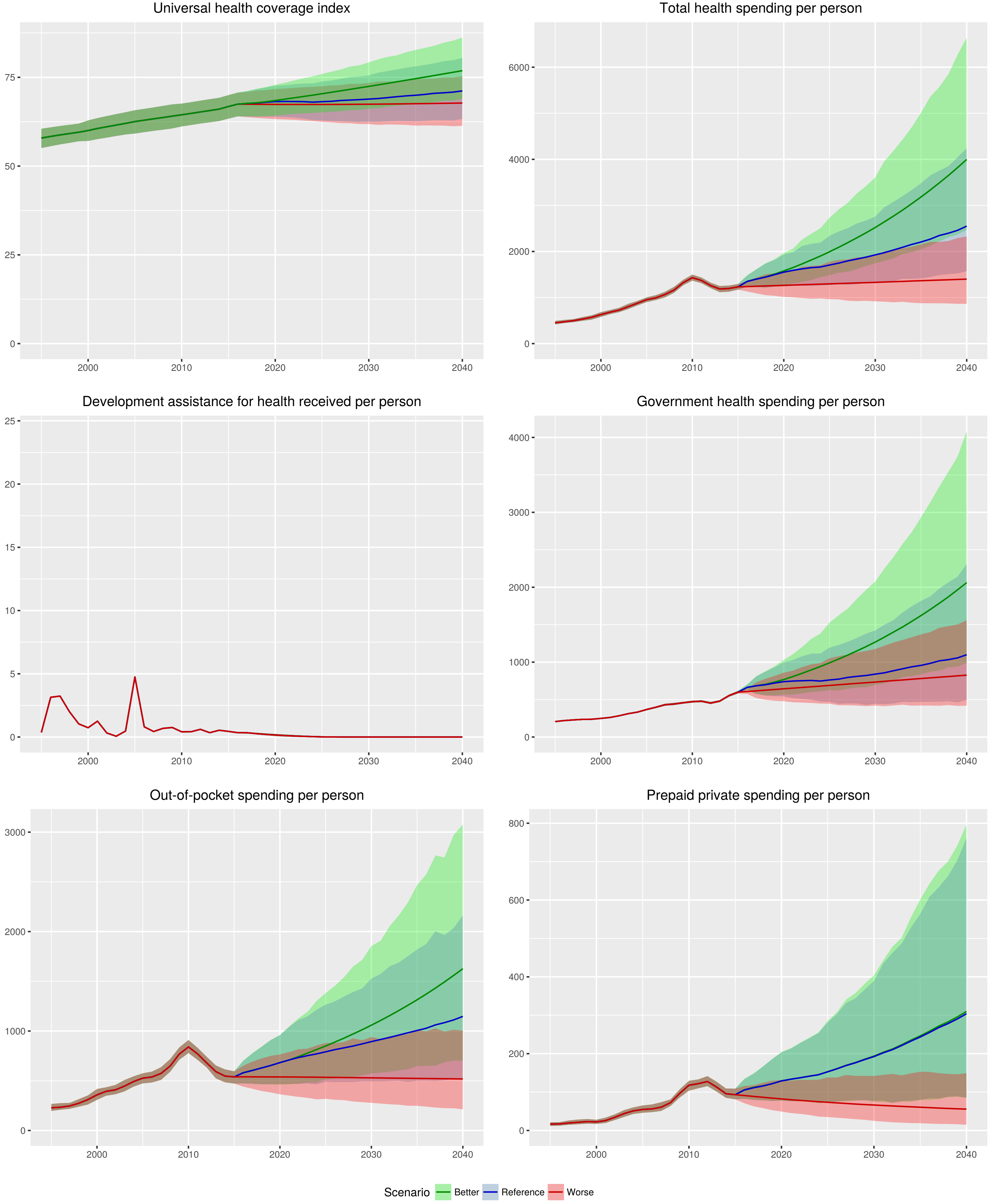
Universal health coverage index
Total health spending per person
Development assistance for health received per person
Government health spending per person
Out-of-pocket spending per person
Prepaid private spending per person
0
25
50
75
0
2000
4000
6000
0
5
10
15
20
25
0
1000
2000
3000
4000
0
1000
2000
3000
0
200
400
600
800
2000
2010
2020
2030
2040
Scenario
Better
Reference
Worse

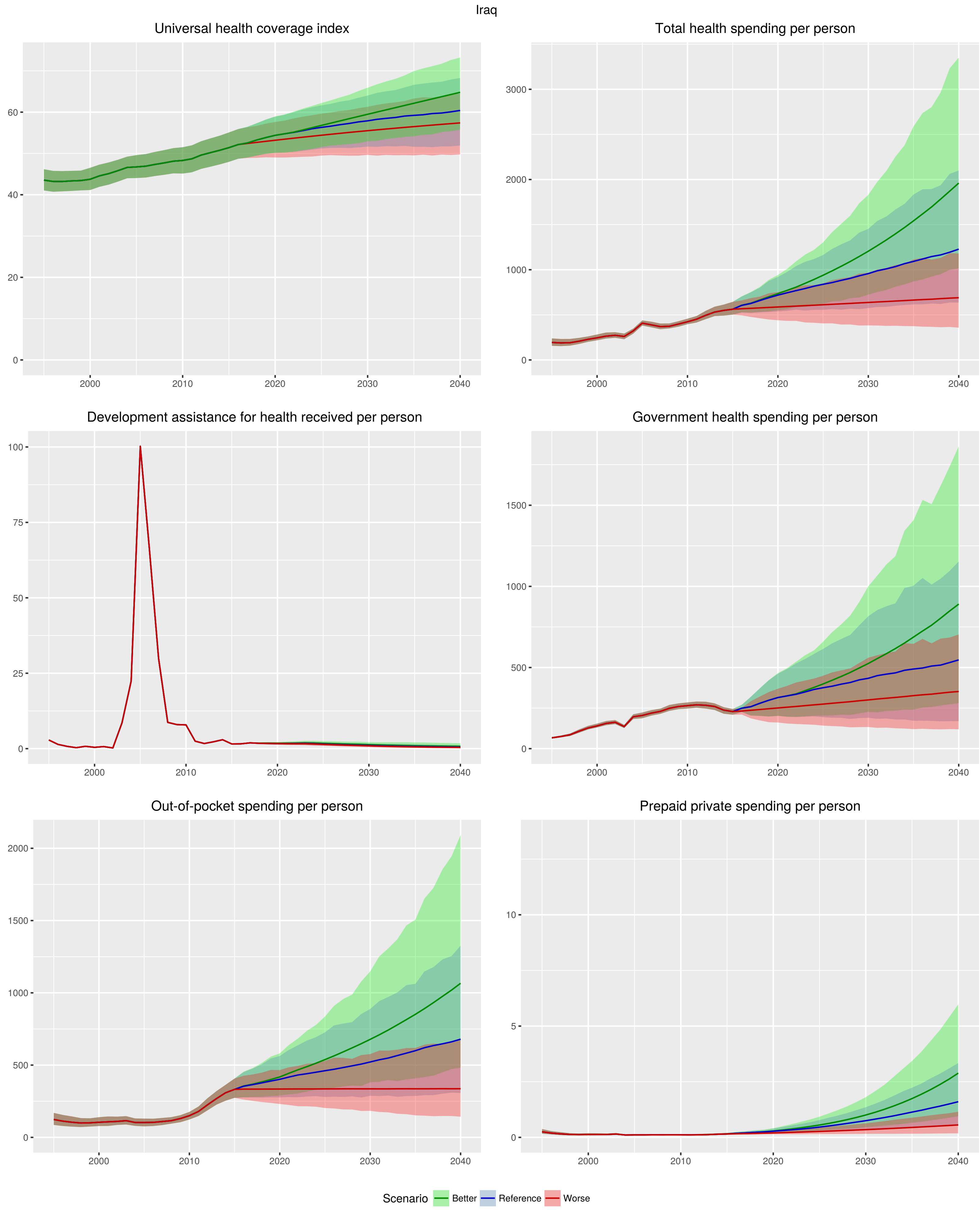
Iraq
Universal health coverage index
0
20
40
60
2000
2010
2020
2030
2040
Total health spending per person
0
1000
2000
3000
Development assistance for health received per person
0
25
50
75
100
Government health spending per person
0
500
1000
1500
Out-of-pocket spending per person
0
500
1000
1500
2000
Prepaid private spending per person
0
5
10
Scenario
Better
Reference
Worse

Ireland

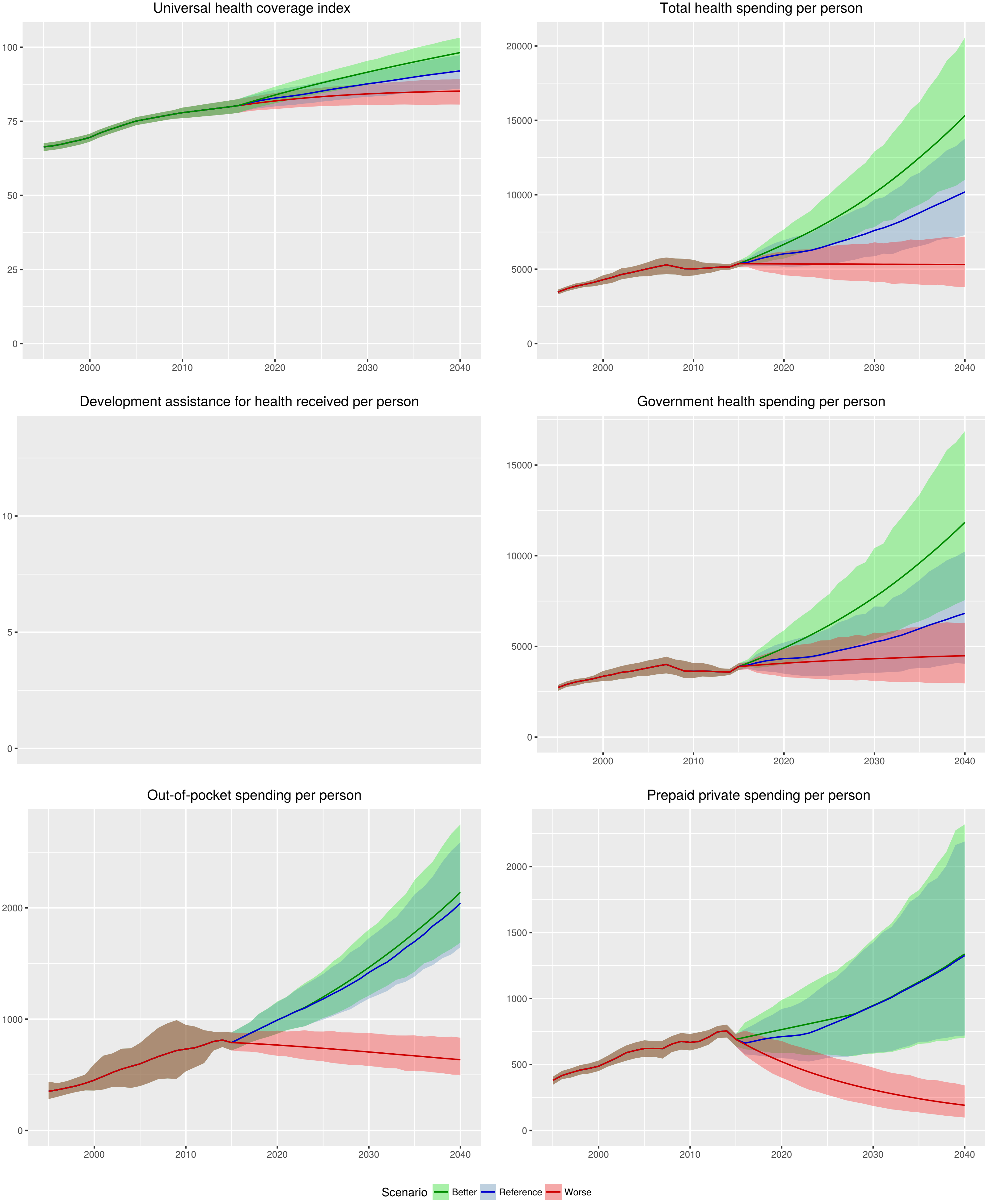

Universal health coverage index
Total health spending per person
Development assistance for health received per person
Government health spending per person
Out-of-pocket spending per person
Prepaid private spending per person
0
25
50
75
100
5000
10000
15000
20000
5
10
500
1000
1500
2000
2010
2020
2030
2040
Scenario
Better
Reference
Worse

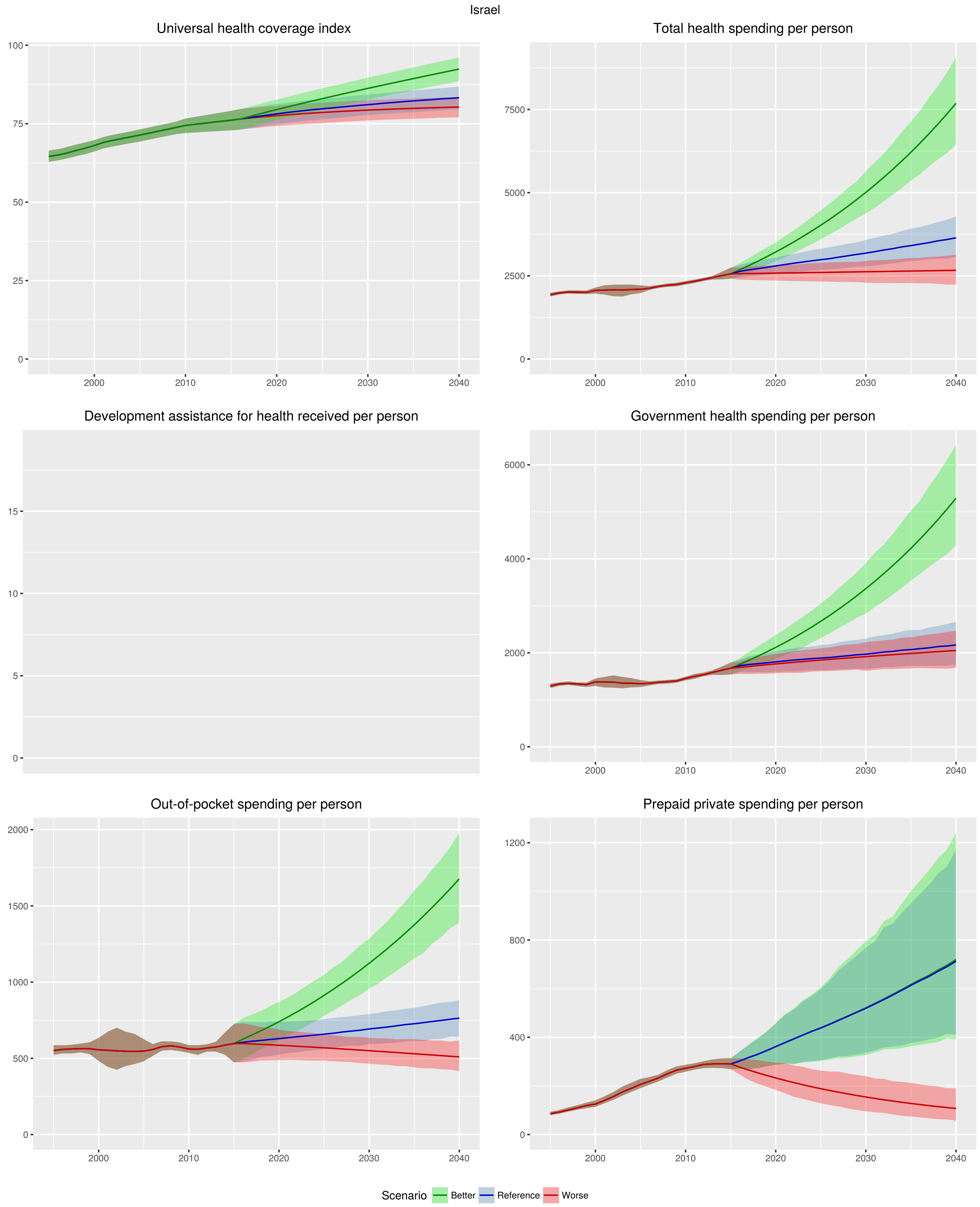
Israel
Universal health coverage index
Total health spending per person
Development assistance for health received per person
Government health spending per person
Out-of-pocket spending per person
Prepaid private spending per person
Scenario
Better
Reference
Worse

Italy

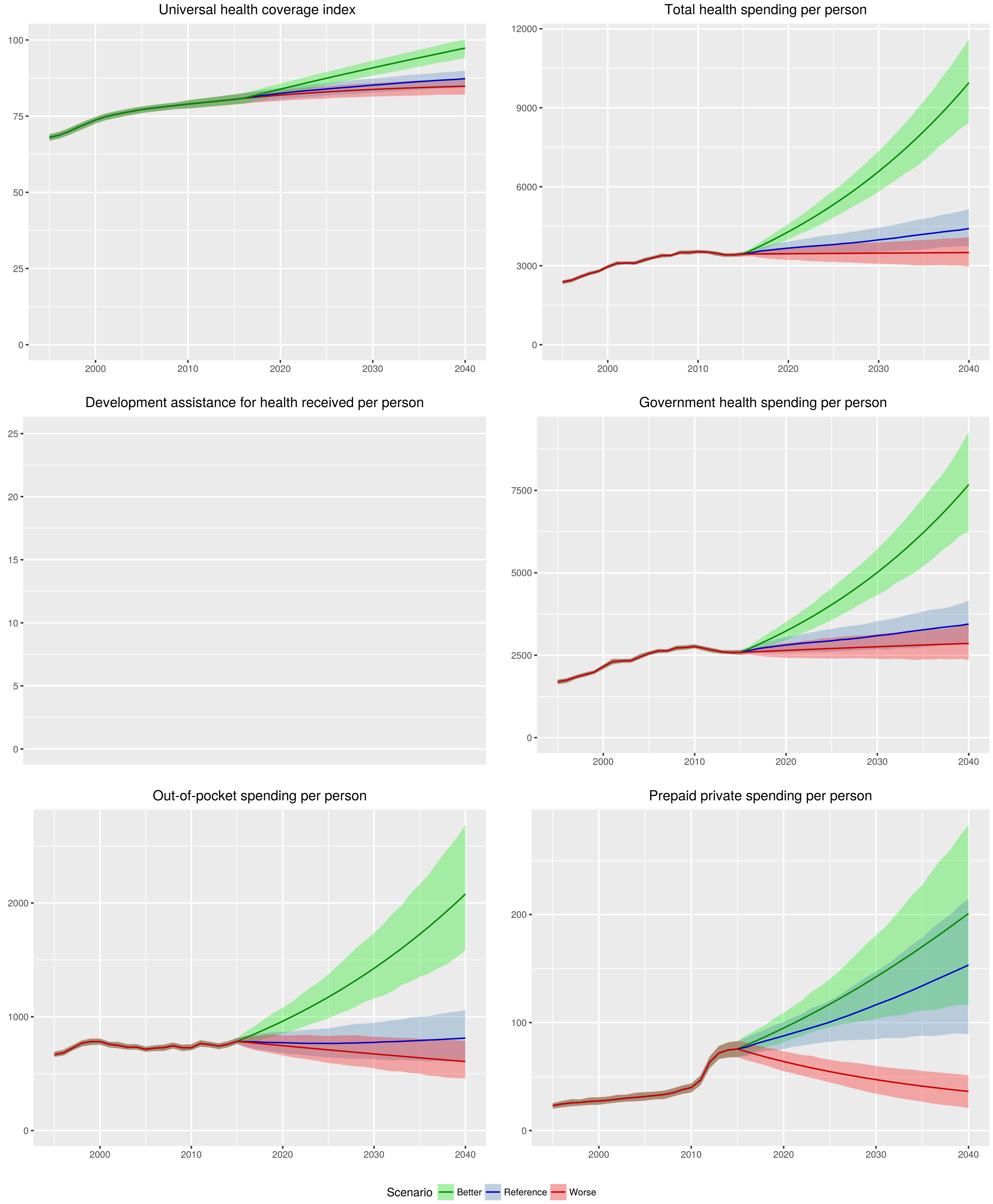

Universal health coverage index
100
75
50
25
0
2000
2010
2020
2030
2040
Total health spending per person
12000
9000
6000
3000
0
Development assistance for health received per person
25
20
15
10
5
0
Government health spending per person
7500
5000
2500
0
Out-of-pocket spending per person
2000
1000
0
Prepaid private spending per person
200
100
0
Scenario
Better
Reference
Worse

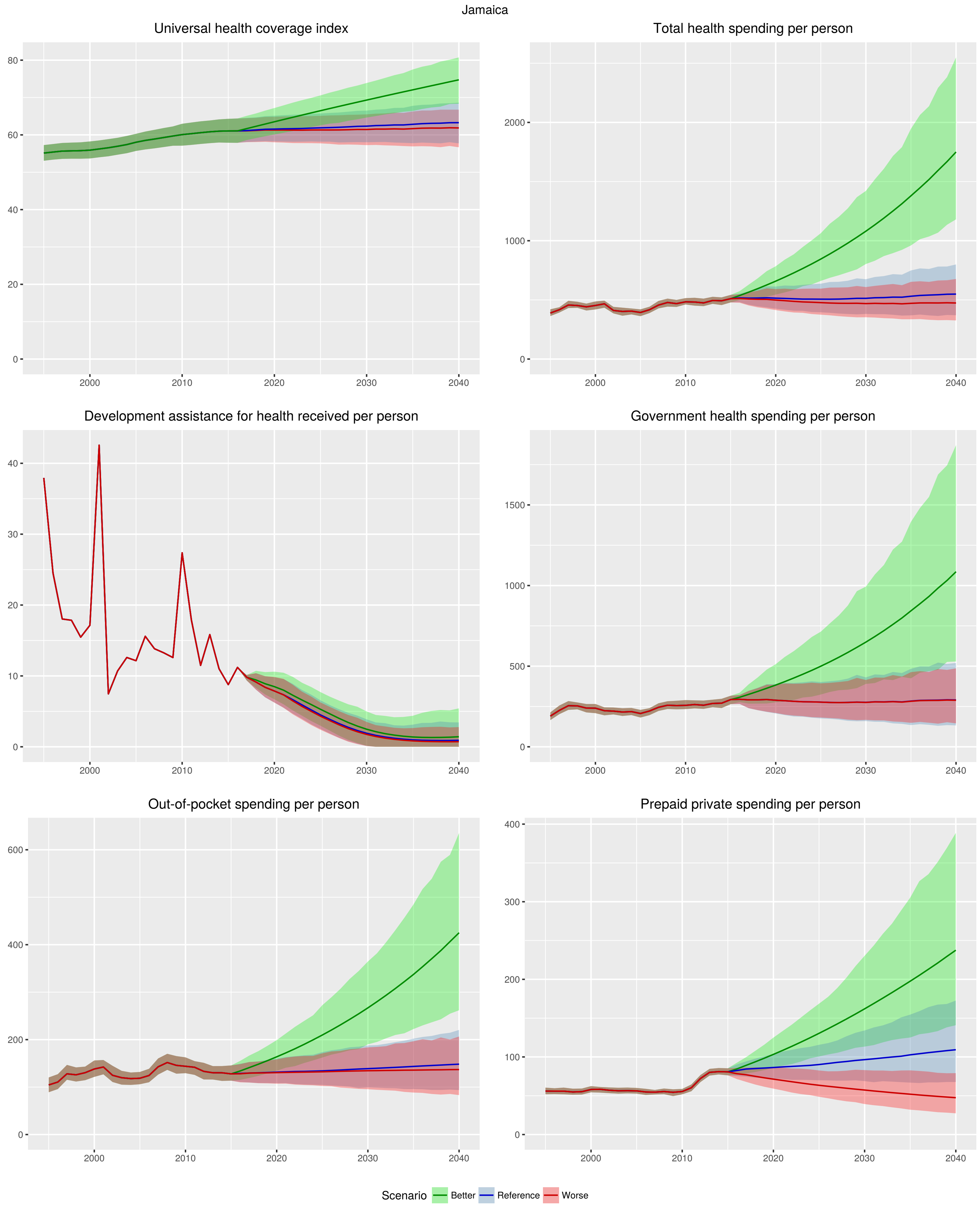

Jamaica
Universal health coverage index
Total health spending per person
Development assistance for health received per person
Government health spending per person
Out-of-pocket spending per person
Prepaid private spending per person
Scenario
Better
Reference
Worse

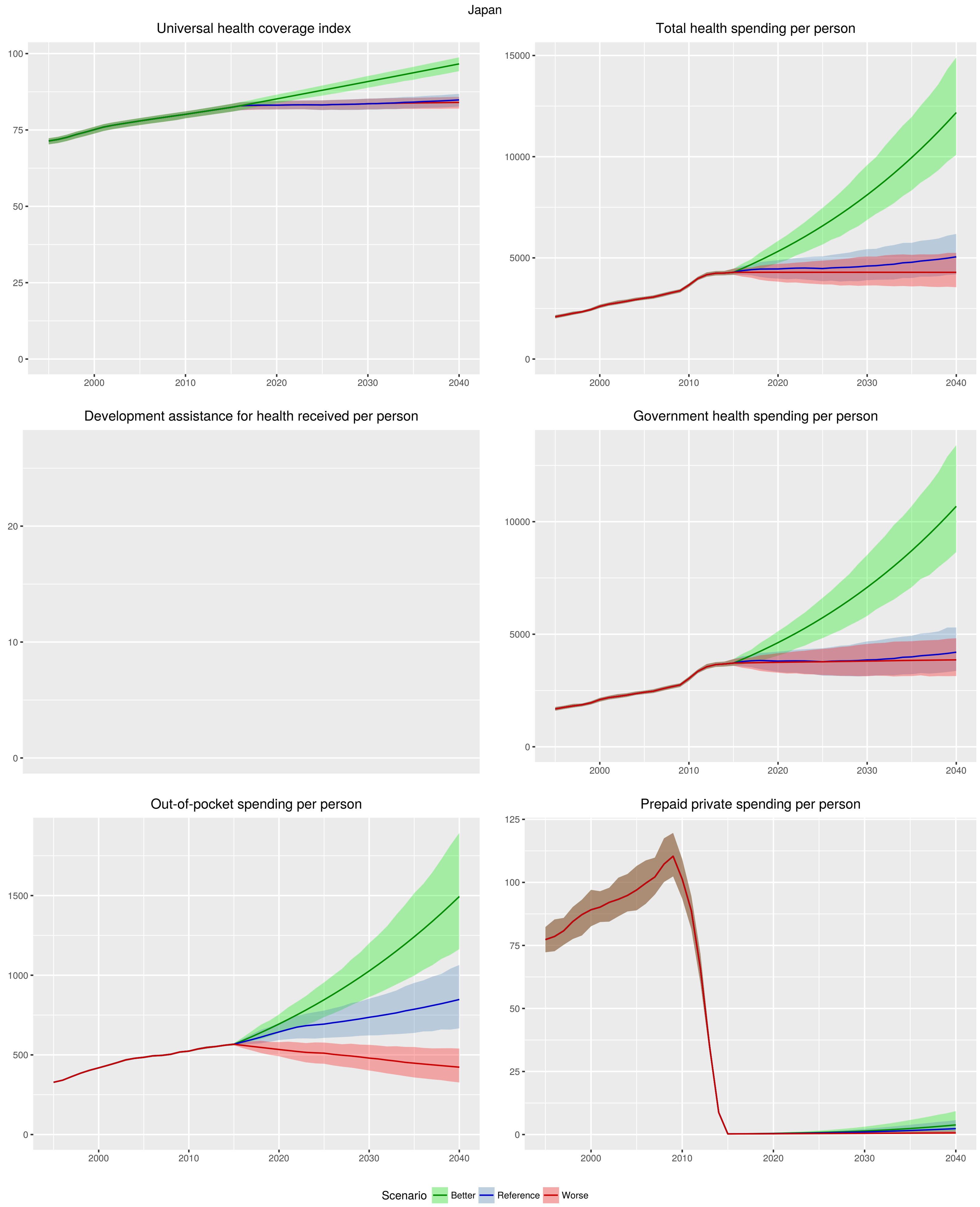
Japan
Universal health coverage index
Total health spending per person
Development assistance for health received per person
Government health spending per person
Out-of-pocket spending per person
Prepaid private spending per person
Scenario
Better
Reference
Worse

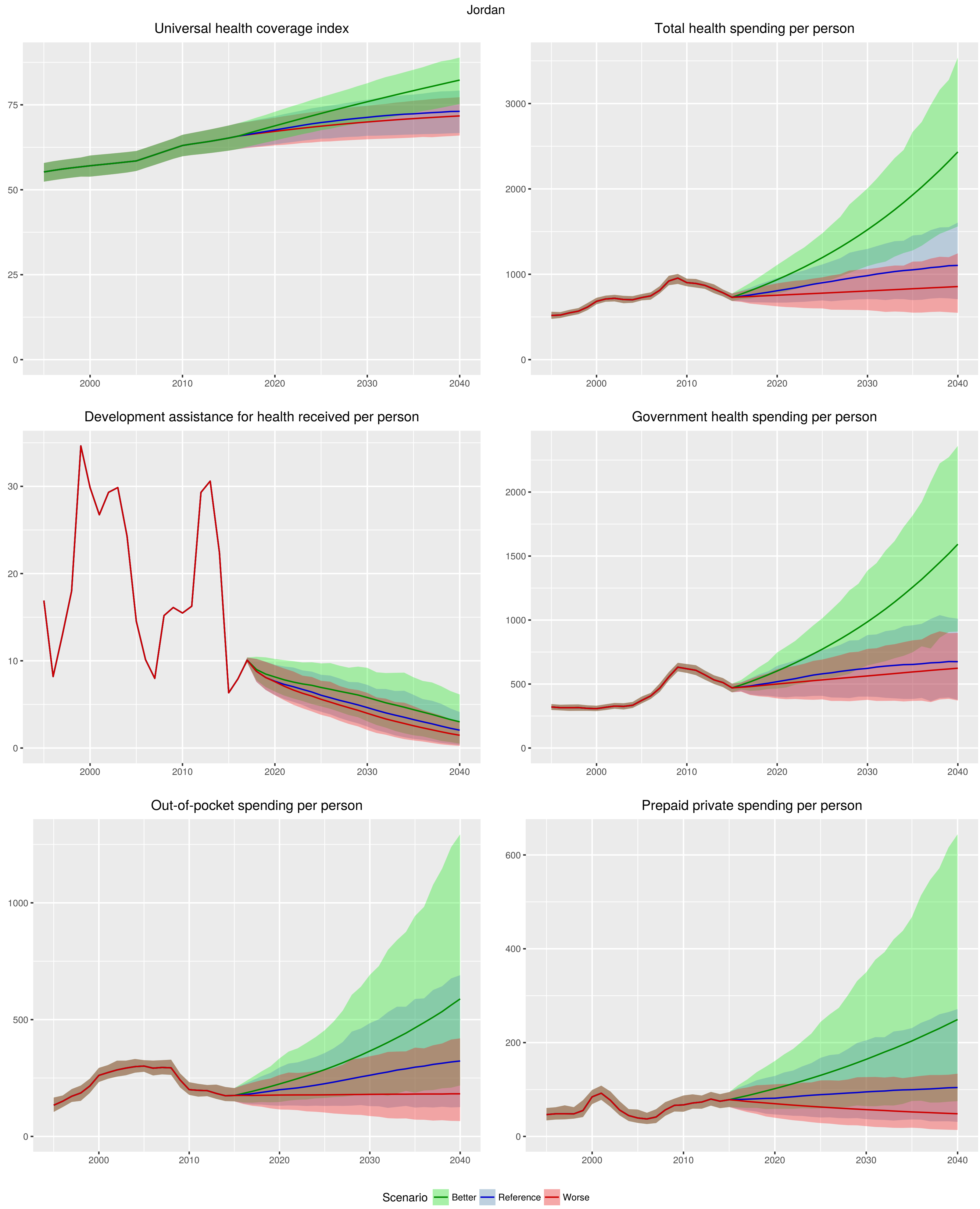
Jordan
Universal health coverage index
Total health spending per person
Development assistance for health received per person
Government health spending per person
Out-of-pocket spending per person
Prepaid private spending per person
0
25
50
75
0
1000
2000
3000
0
10
20
30
0
500
1000
1500
2000
0
500
1000
0
200
400
600
2000
2010
2020
2030
2040
Scenario
Better
Reference
Worse

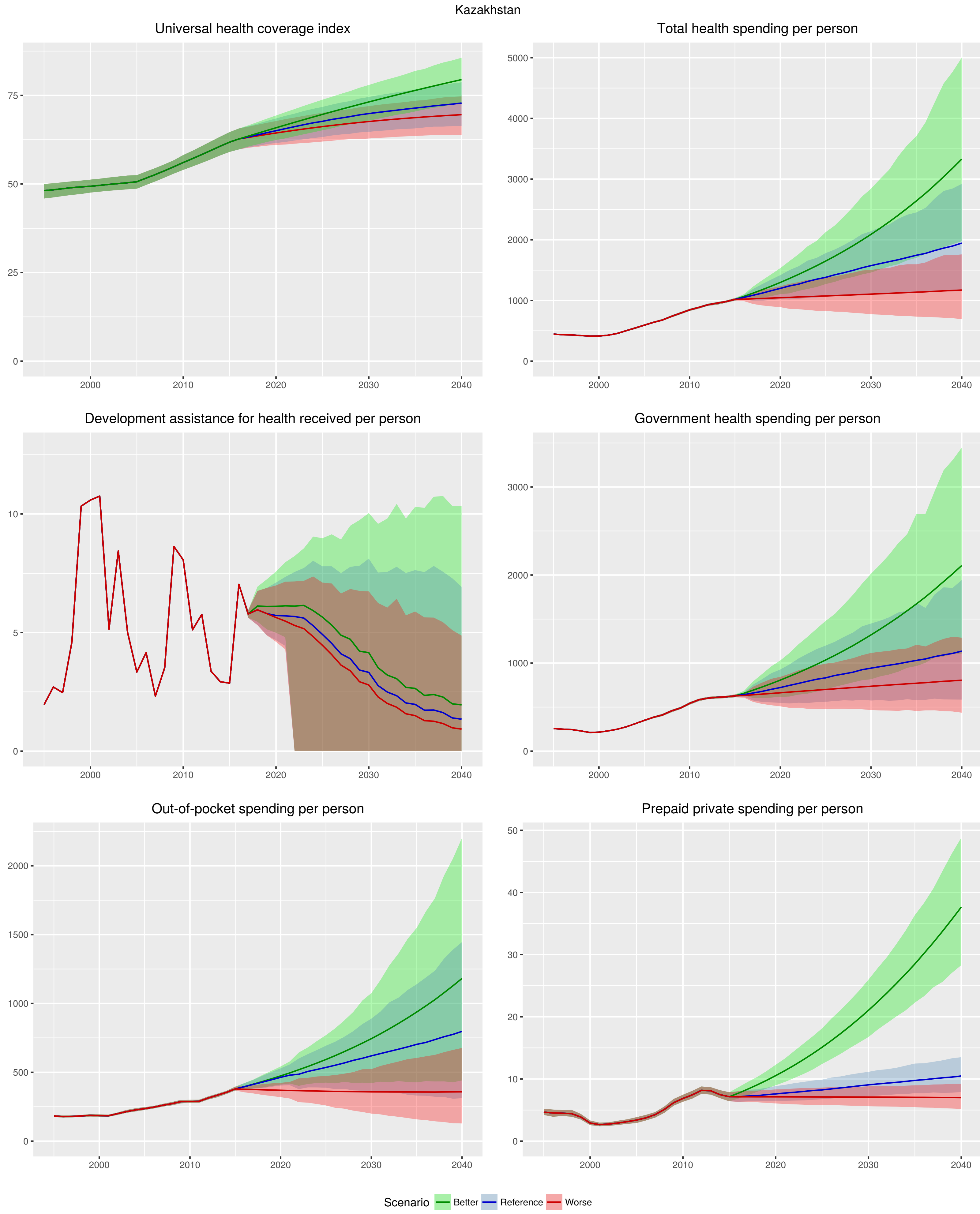
Kazakhstan
Universal health coverage index
0
25
50
75
2000
2010
2020
2030
2040
Total health spending per person
0
1000
2000
3000
4000
5000
2000
2010
2020
2030
2040
Development assistance for health received per person
0
5
10
2000
2010
2020
2030
2040
Government health spending per person
0
1000
2000
3000
2000
2010
2020
2030
2040
Out-of-pocket spending per person
0
500
1000
1500
2000
2000
2010
2020
2030
2040
Prepaid private spending per person
0
10
20
30
40
50
2000
2010
2020
2030
2040
Scenario
Better
Reference
Worse

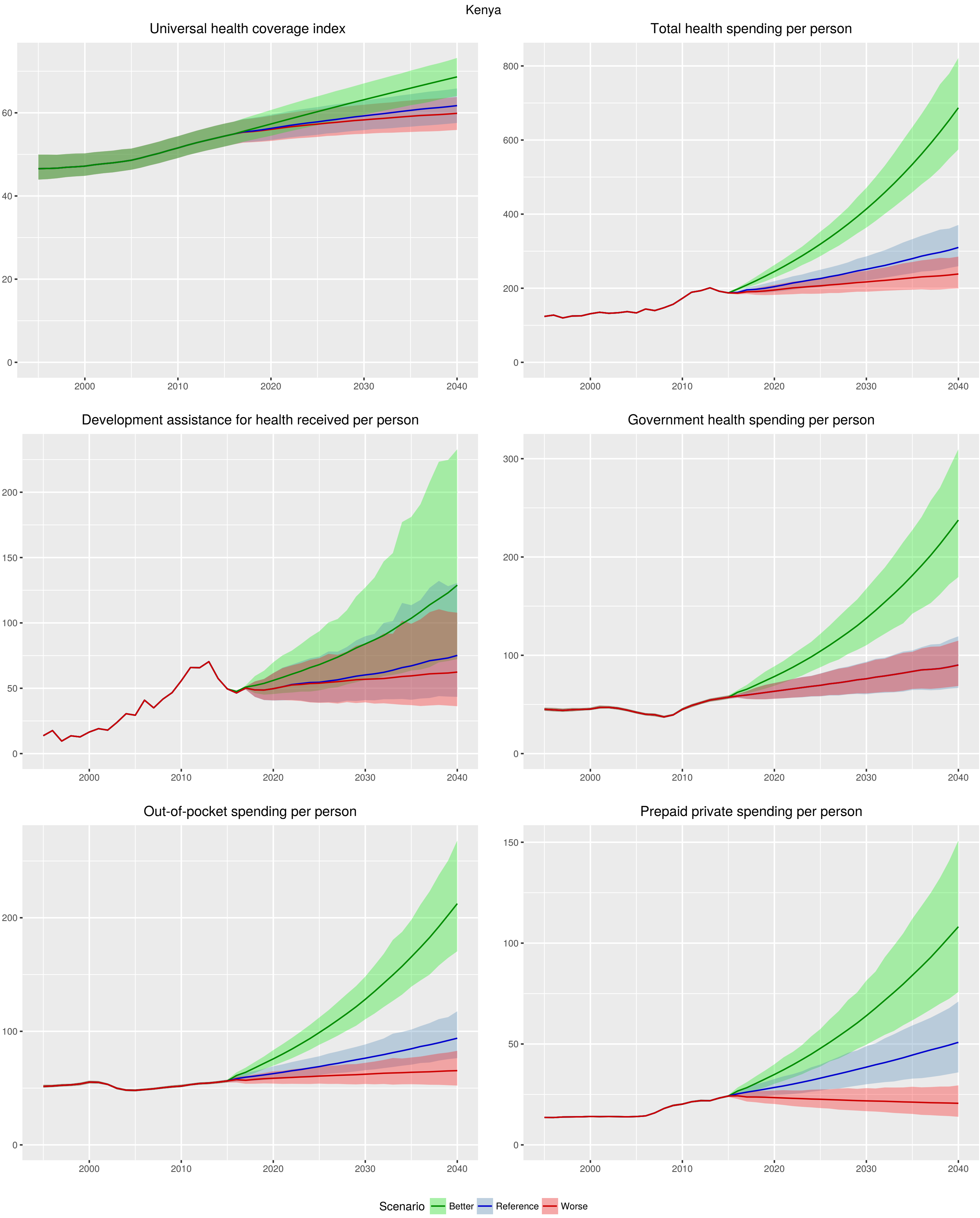

Kenya
Universal health coverage index
Total health spending per person
Development assistance for health received per person
Government health spending per person
Out-of-pocket spending per person
Prepaid private spending per person
Scenario
Better
Reference
Worse

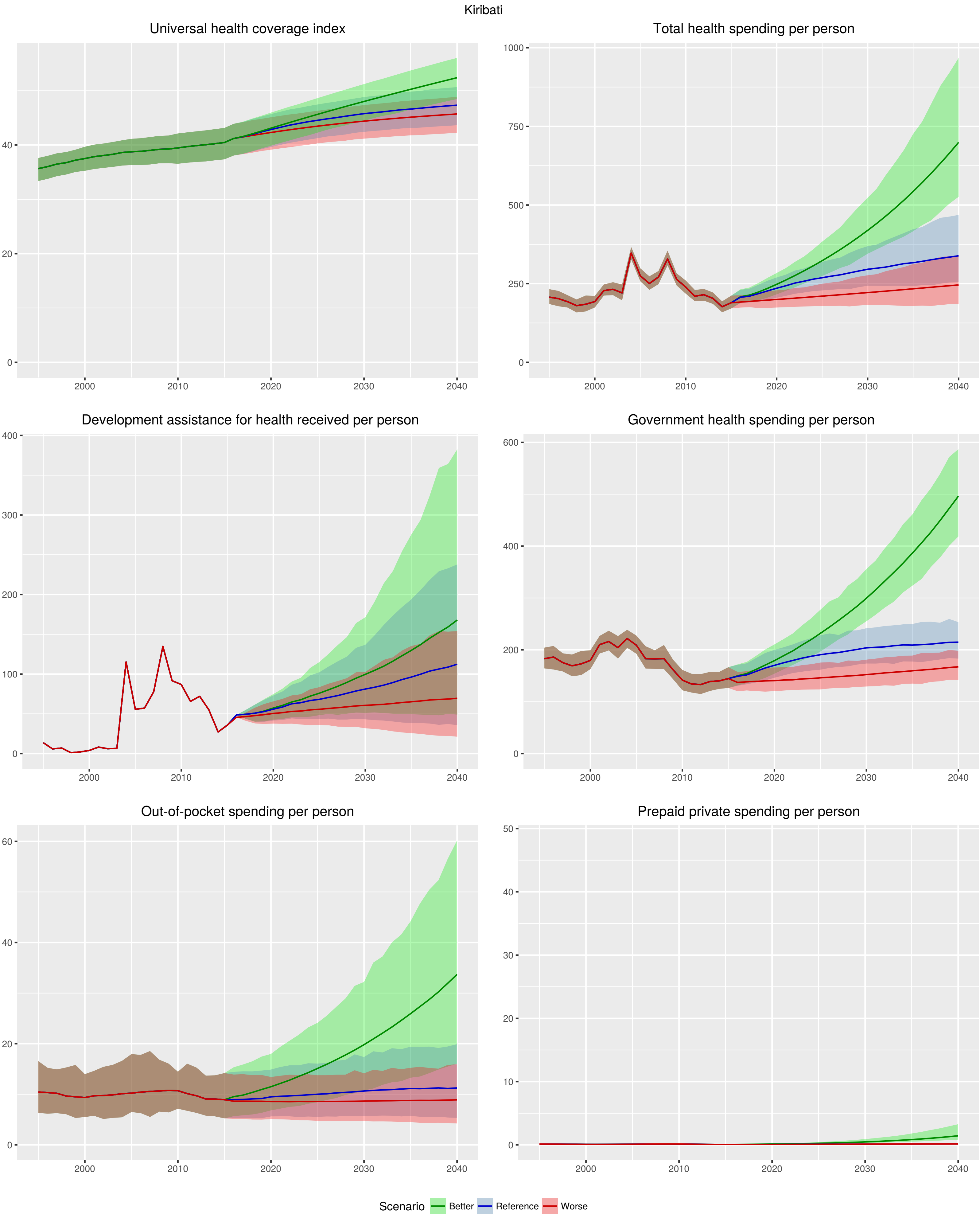
Kiribati
Universal health coverage index
Total health spending per person
Development assistance for health received per person
Government health spending per person
Out-of-pocket spending per person
Prepaid private spending per person
0
20
40
0
250
500
750
1000
0
100
200
300
400
0
200
400
600
0
20
40
60
0
10
20
30
40
50
2000
2010
2020
2030
2040
Scenario
Better
Reference
Worse

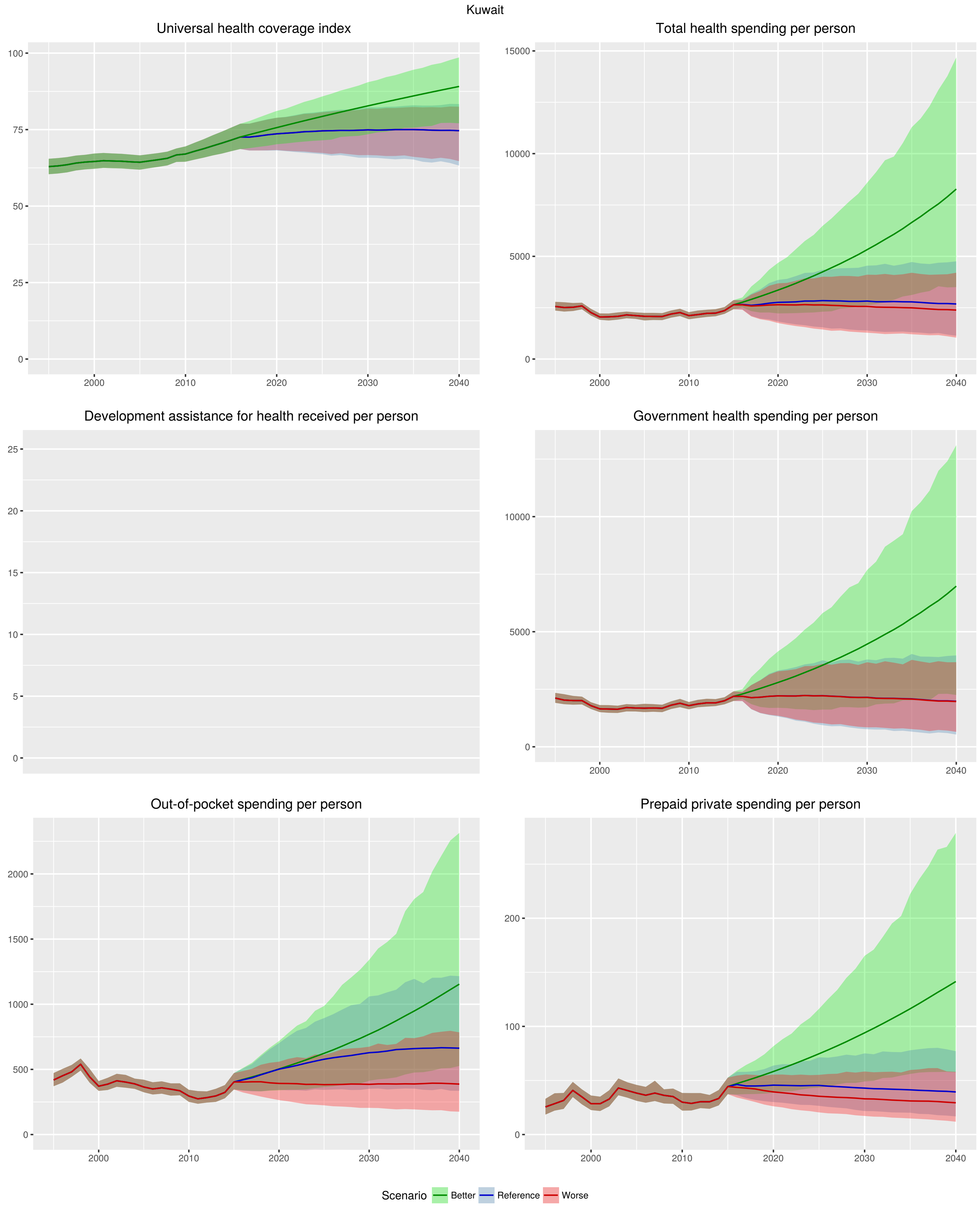

Kuwait
Universal health coverage index
Total health spending per person
Development assistance for health received per person
Government health spending per person
Out-of-pocket spending per person
Prepaid private spending per person
0
25
50
75
100
5000
10000
15000
5
10
15
20
2000
2010
2020
2030
2040
500
1000
1500
200
Scenario
Better
Reference
Worse

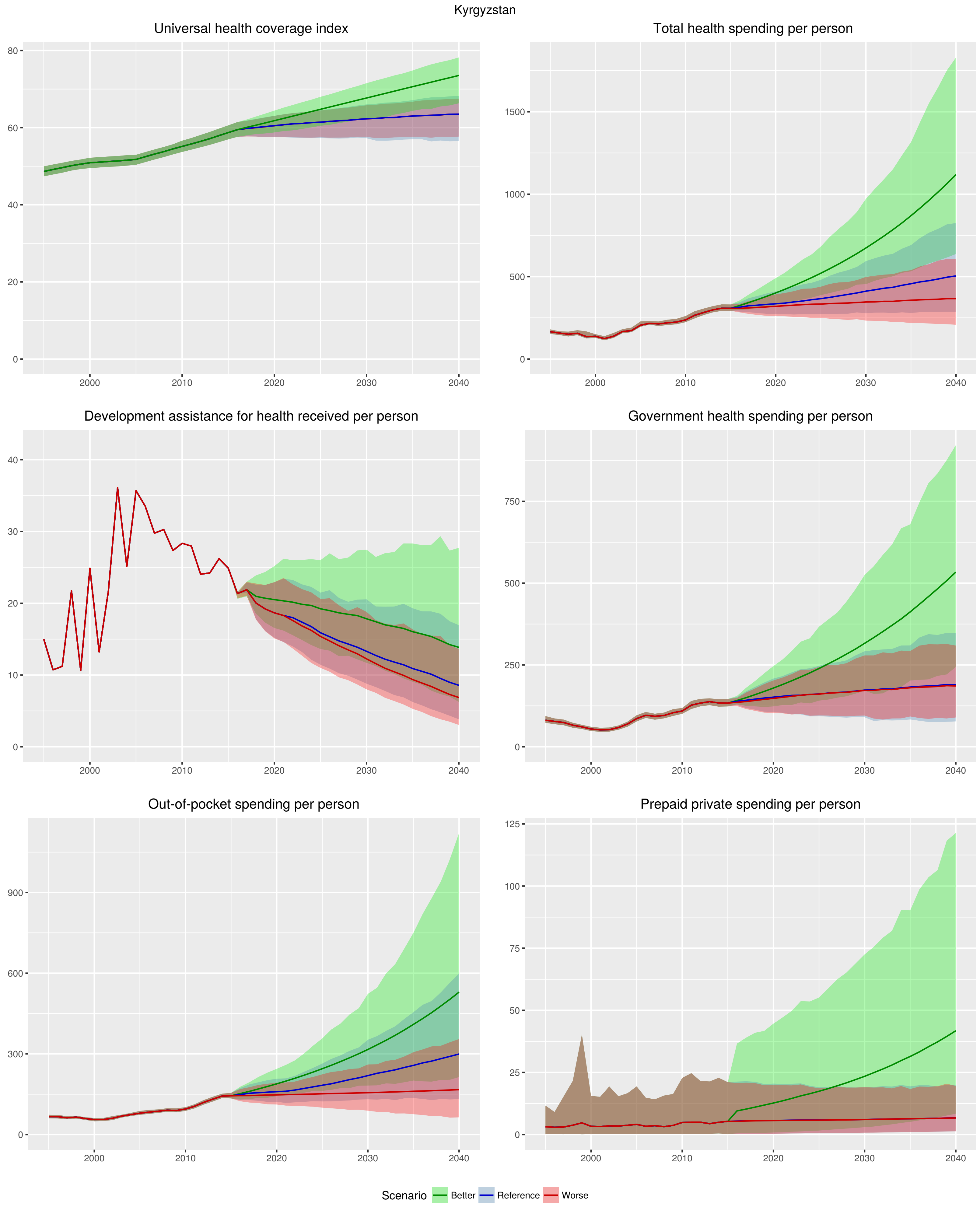

Kyrgyzstan
Universal health coverage index
Total health spending per person
Development assistance for health received per person
Government health spending per person
Out-of-pocket spending per person
Prepaid private spending per person
Scenario
Better
Reference
Worse

Laos

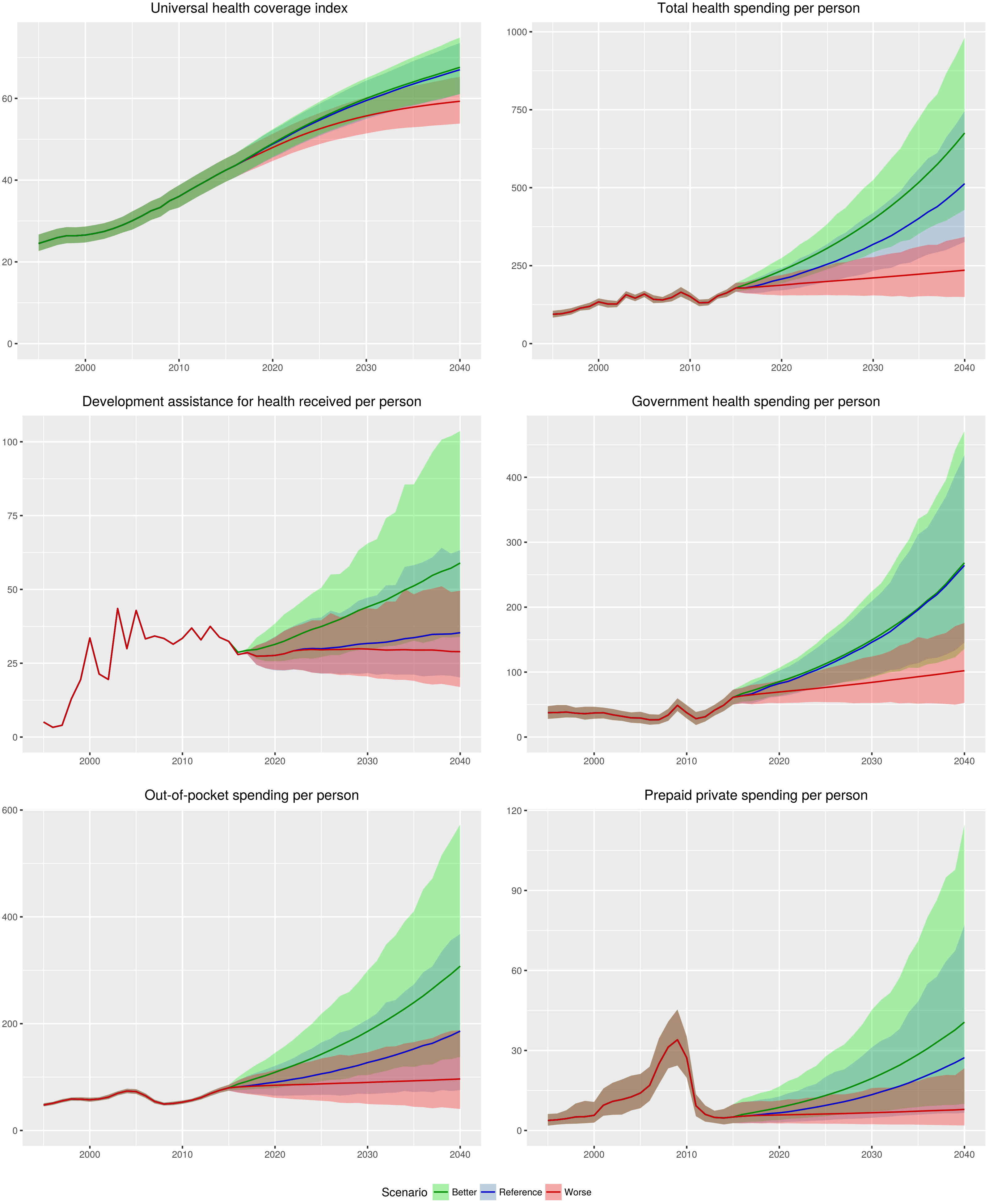

Universal health coverage index
Total health spending per person
Development assistance for health received per person
Government health spending per person
Out-of-pocket spending per person
Prepaid private spending per person
2000
2010
2020
2030
2040
Scenario
Better
Reference
Worse

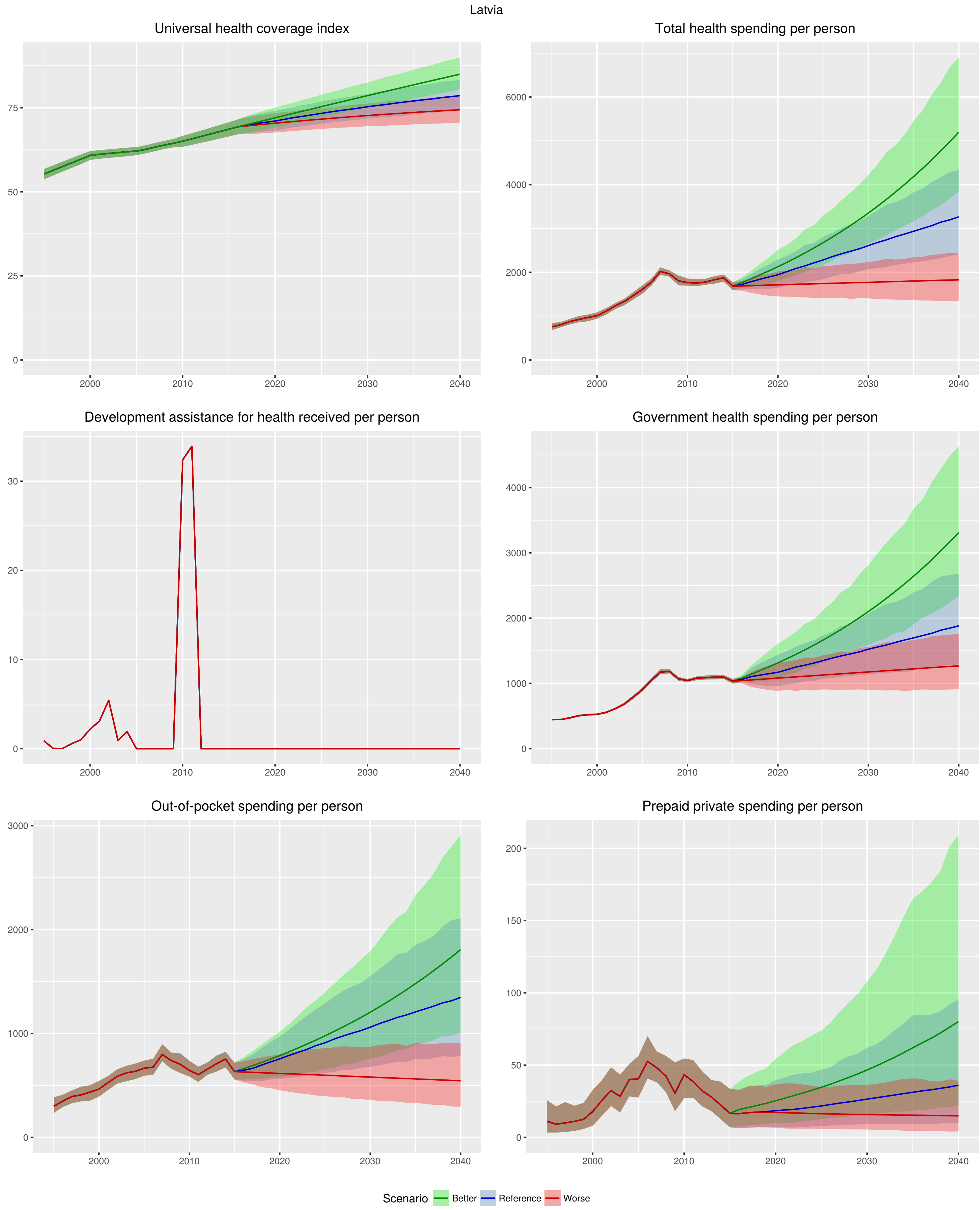
Latvia
Universal health coverage index
Total health spending per person
Development assistance for health received per person
Government health spending per person
Out-of-pocket spending per person
Prepaid private spending per person
Scenario
Better
Reference
Worse

Lebanon

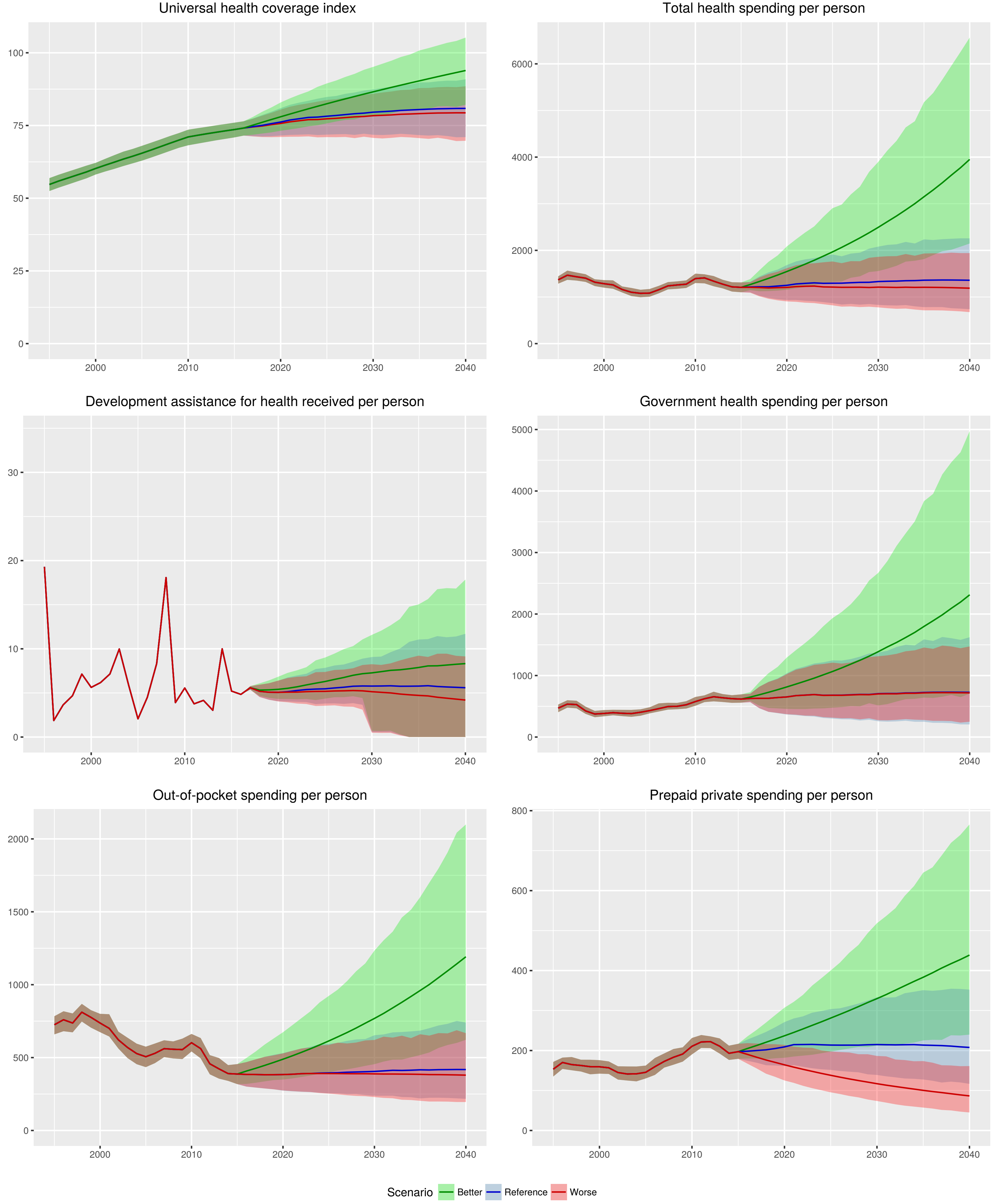
Universal health coverage index
0
25
50
75
100
2000
2010
2020
2030
2040
Total health spending per person
0
2000
4000
6000
Development assistance for health received per person
0
10
20
30
Government health spending per person
0
1000
2000
3000
4000
5000
Out-of-pocket spending per person
0
500
1000
1500
2000
Prepaid private spending per person
0
200
400
600
800
Scenario
Better
Reference
Worse

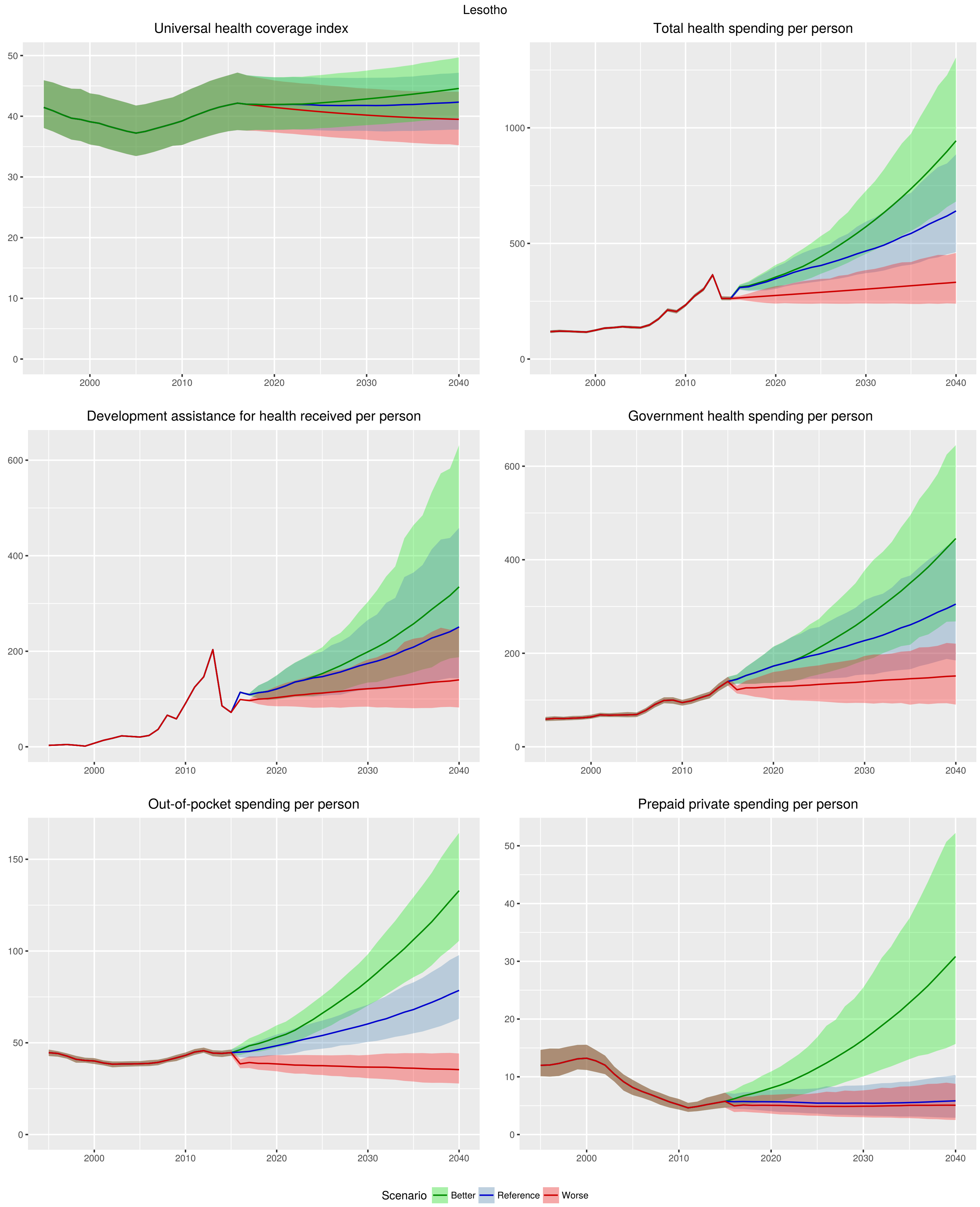

Lesotho
Universal health coverage index
Total health spending per person
Development assistance for health received per person
Government health spending per person
Out-of-pocket spending per person
Prepaid private spending per person
Scenario
Better
Reference
Worse

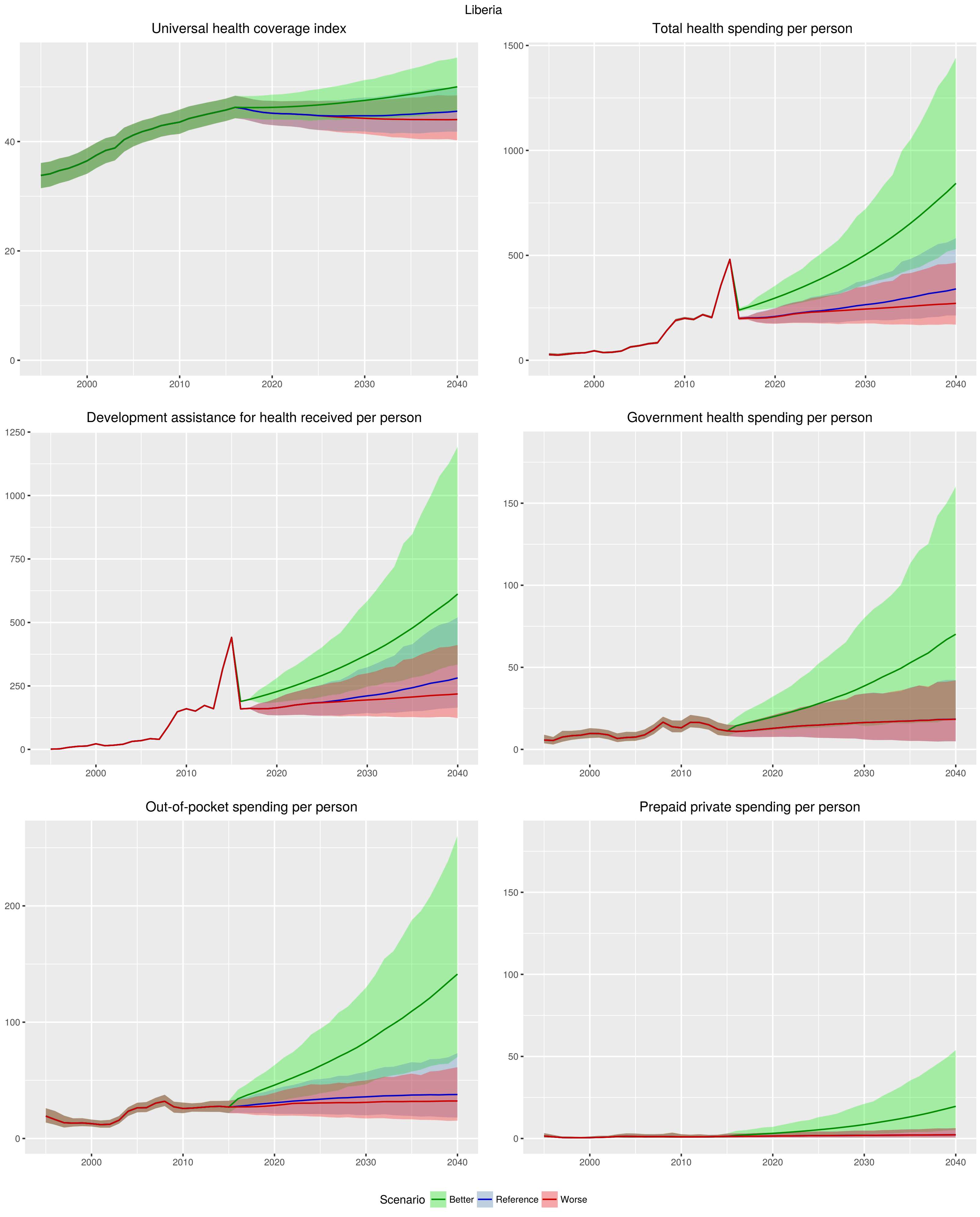

Liberia
Universal health coverage index
Total health spending per person
Development assistance for health received per person
Government health spending per person
Out-of-pocket spending per person
Prepaid private spending per person
Scenario
Better
Reference
Worse

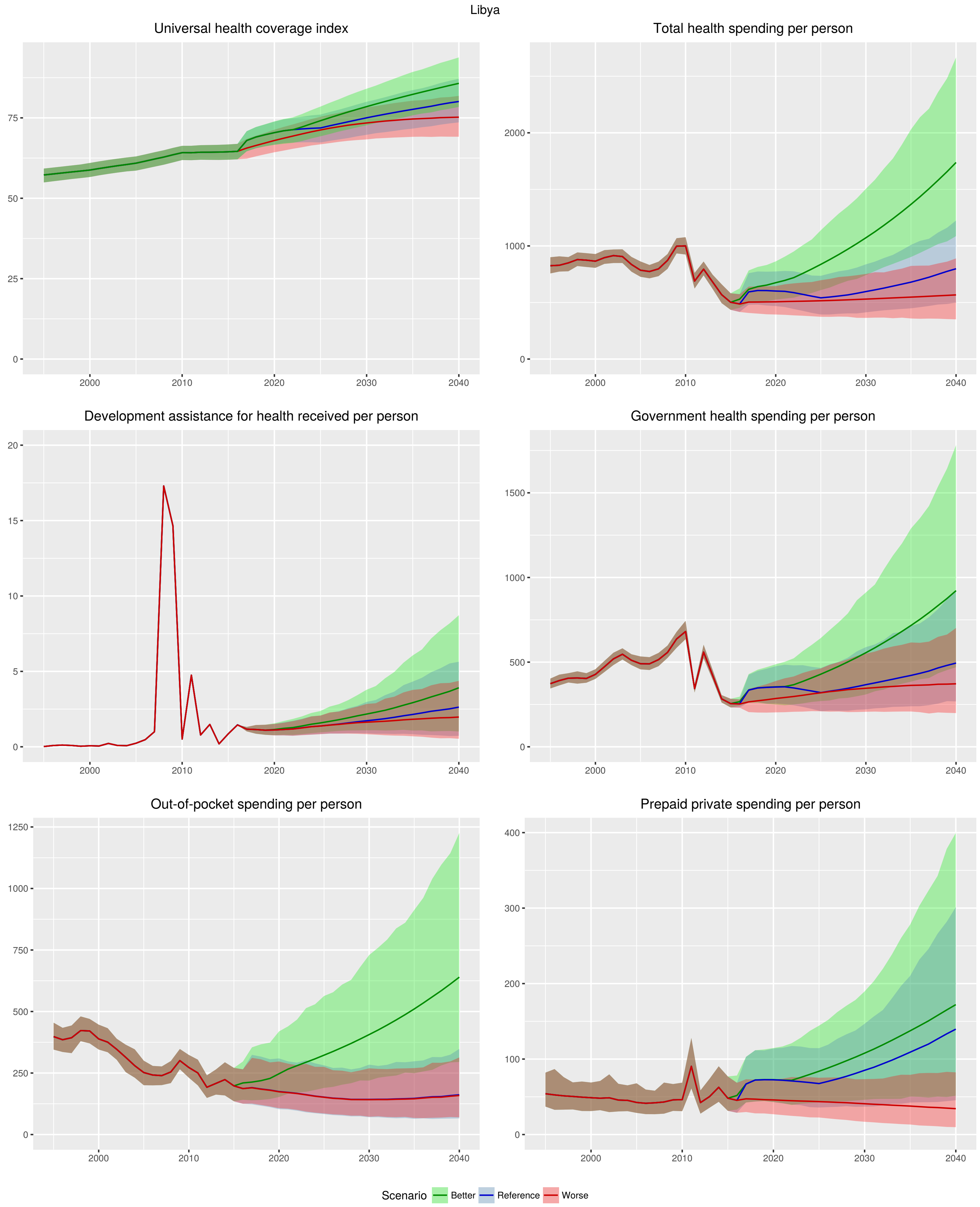
Libya
Universal health coverage index
Total health spending per person
Development assistance for health received per person
Government health spending per person
Out-of-pocket spending per person
Prepaid private spending per person
0
25
50
75
1000
2000
5
10
15
20
500
1500
250
750
1250
100
200
300
400
2000
2010
2020
2030
2040
Scenario
Better
Reference
Worse

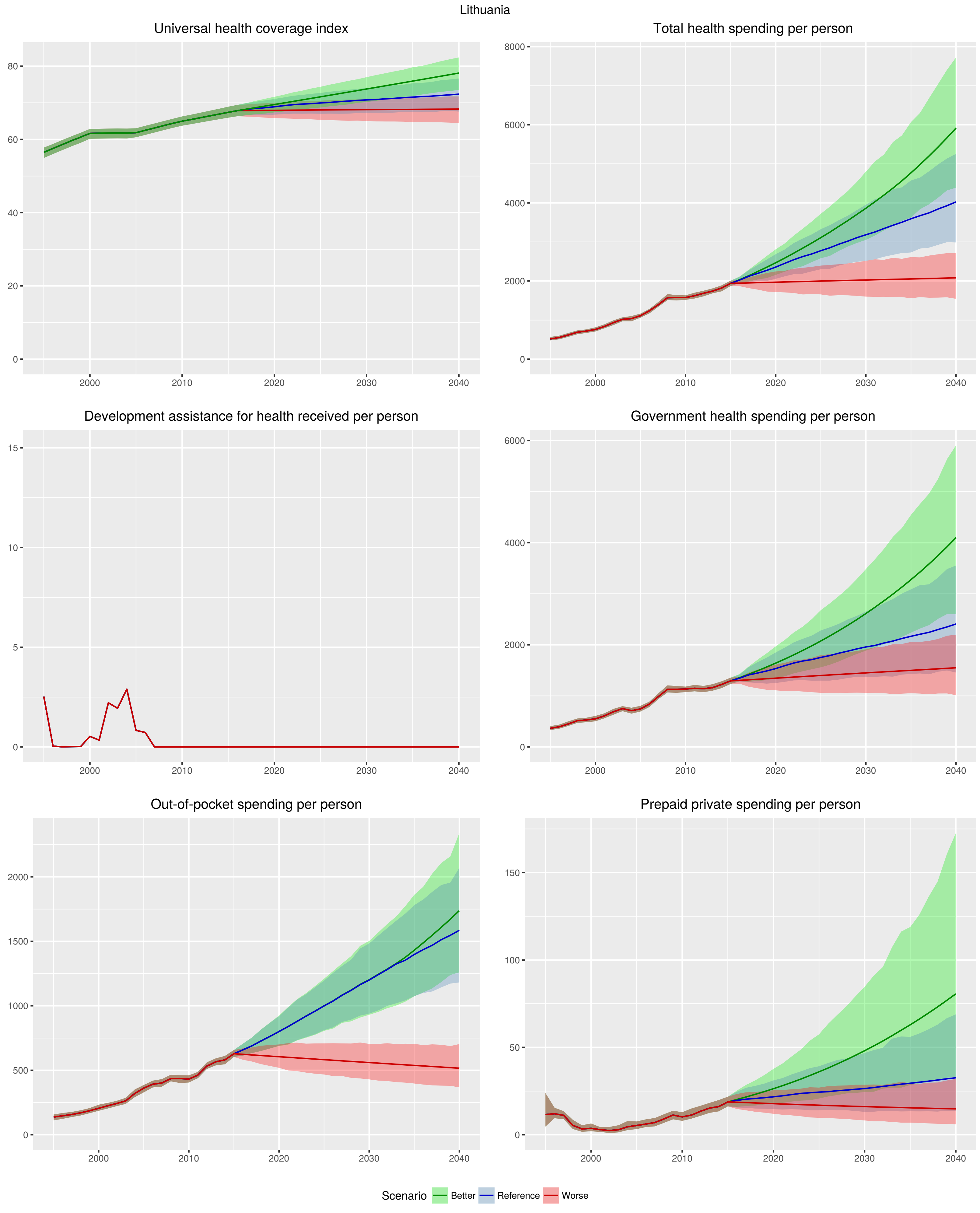

Lithuania
Universal health coverage index
Total health spending per person
Development assistance for health received per person
Government health spending per person
Out-of-pocket spending per person
Prepaid private spending per person
Scenario
Better
Reference
Worse

Luxembourg

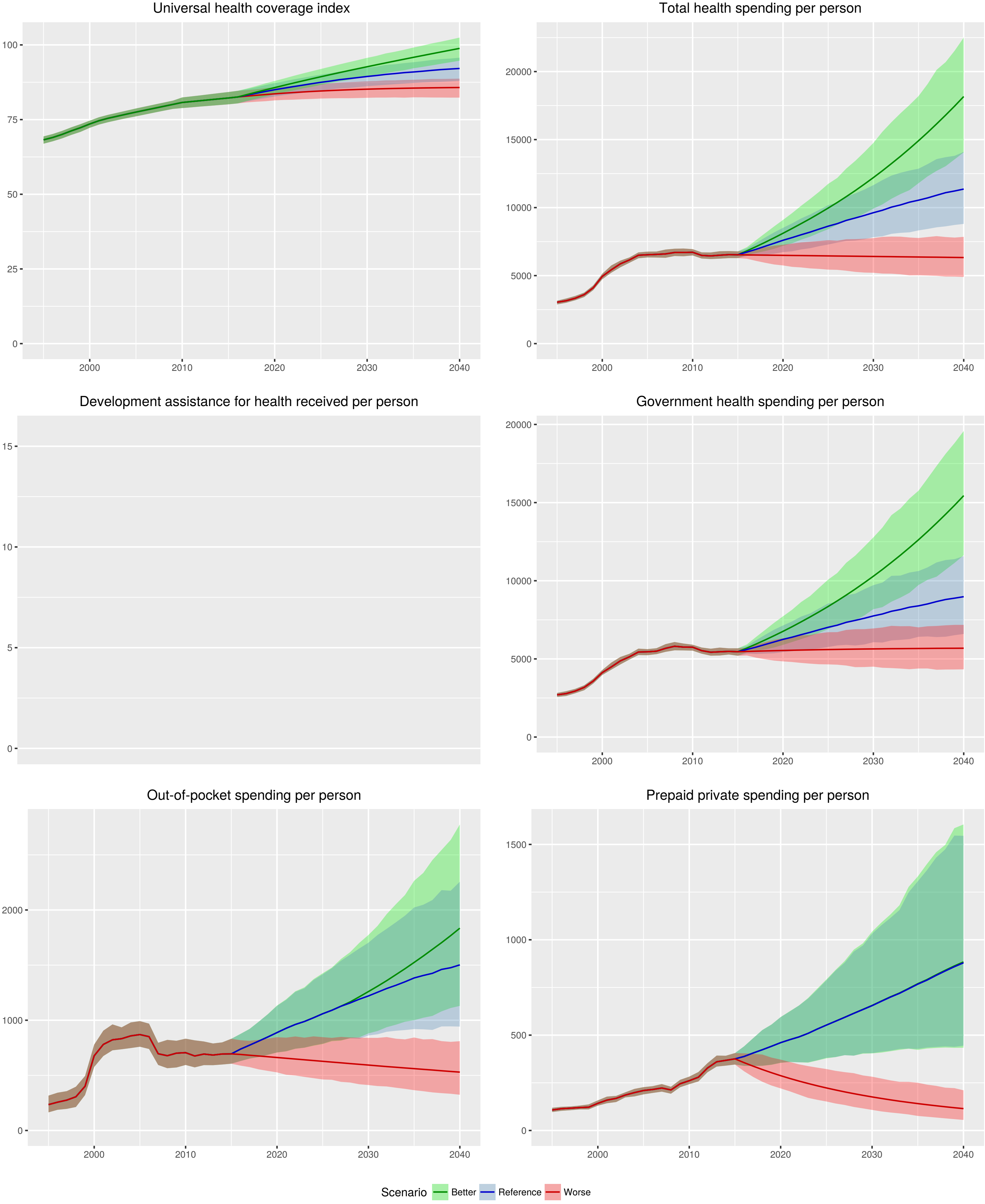
Universal health coverage index
0
25
50
75
100
2000
2010
2020
2030
2040
Total health spending per person
0
5000
10000
15000
20000
Development assistance for health received per person
0
5
10
15
Government health spending per person
Out-of-pocket spending per person
1000
2000
Prepaid private spending per person
500
1000
1500
Scenario
Better
Reference
Worse

Macedonia

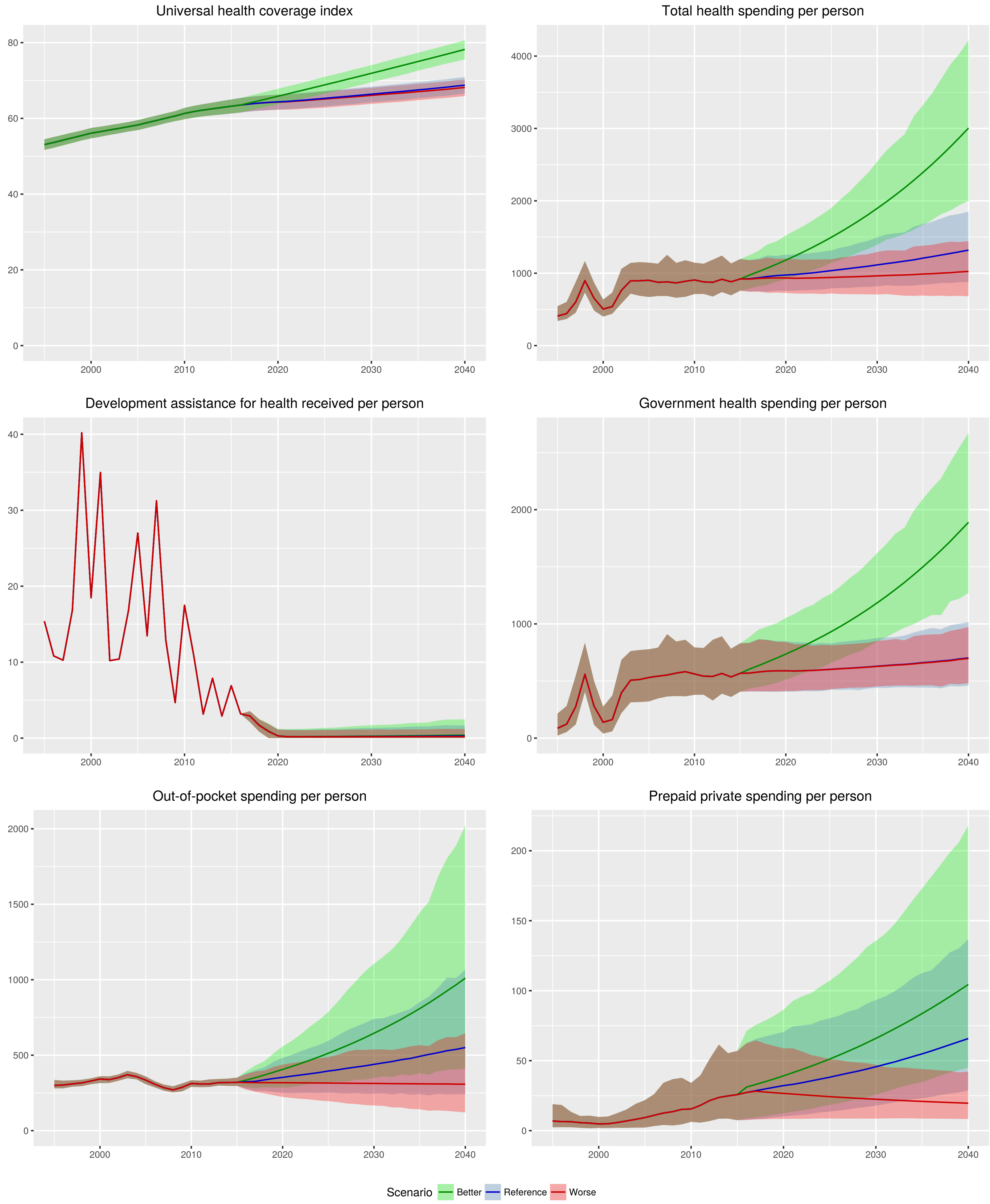

Universal health coverage index
80
60
40
20
0
2000
2010
2020
2030
2040
Total health spending per person
4000
3000
2000
1000
0
2000
2010
2020
2030
2040
Development assistance for health received per person
40
30
20
10
0
2000
2010
2020
2030
2040
Government health spending per person
2000
1000
0
2000
2010
2020
2030
2040
Out-of-pocket spending per person
2000
1500
1000
500
0
2000
2010
2020
2030
2040
Prepaid private spending per person
200
150
100
50
0
2000
2010
2020
2030
2040
Scenario
Better
Reference
Worse

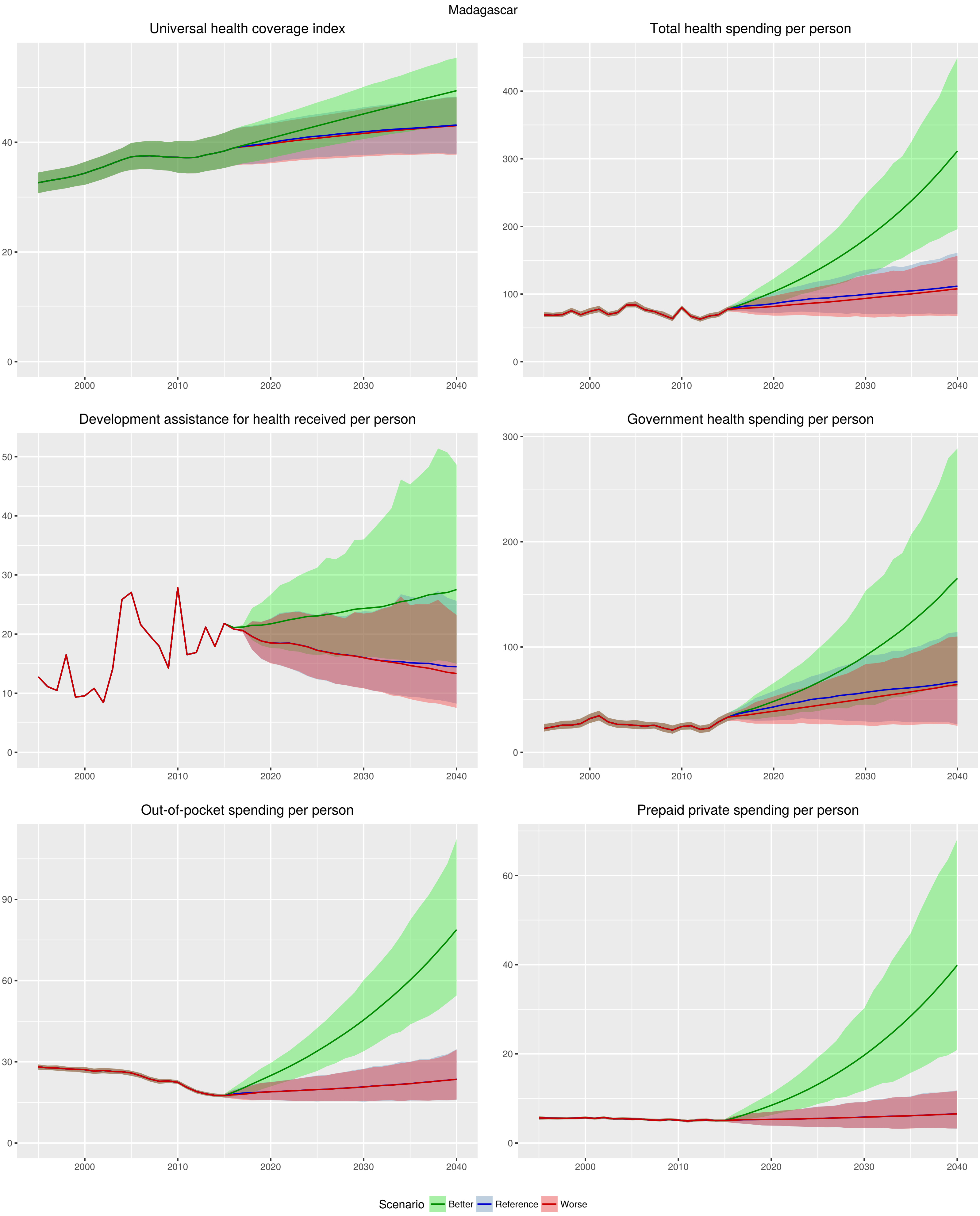

Madagascar
Universal health coverage index
Total health spending per person
Development assistance for health received per person
Government health spending per person
Out-of-pocket spending per person
Prepaid private spending per person
0
20
40
100
200
300
400
10
30
50
60
90
2000
2010
2020
2030
2040
Scenario
Better
Reference
Worse

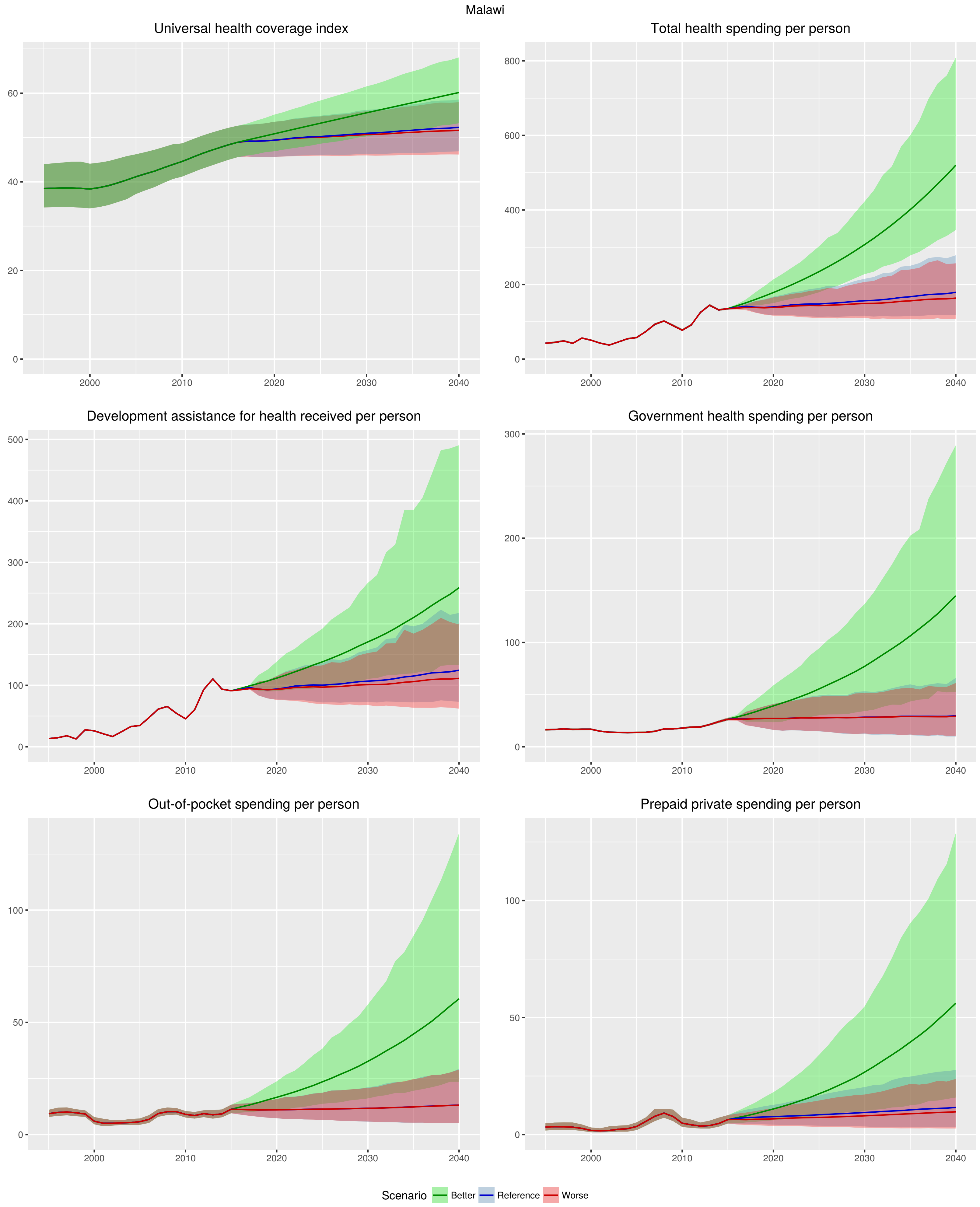

Malawi
Universal health coverage index
Total health spending per person
Development assistance for health received per person
Government health spending per person
Out-of-pocket spending per person
Prepaid private spending per person
Scenario
Better
Reference
Worse

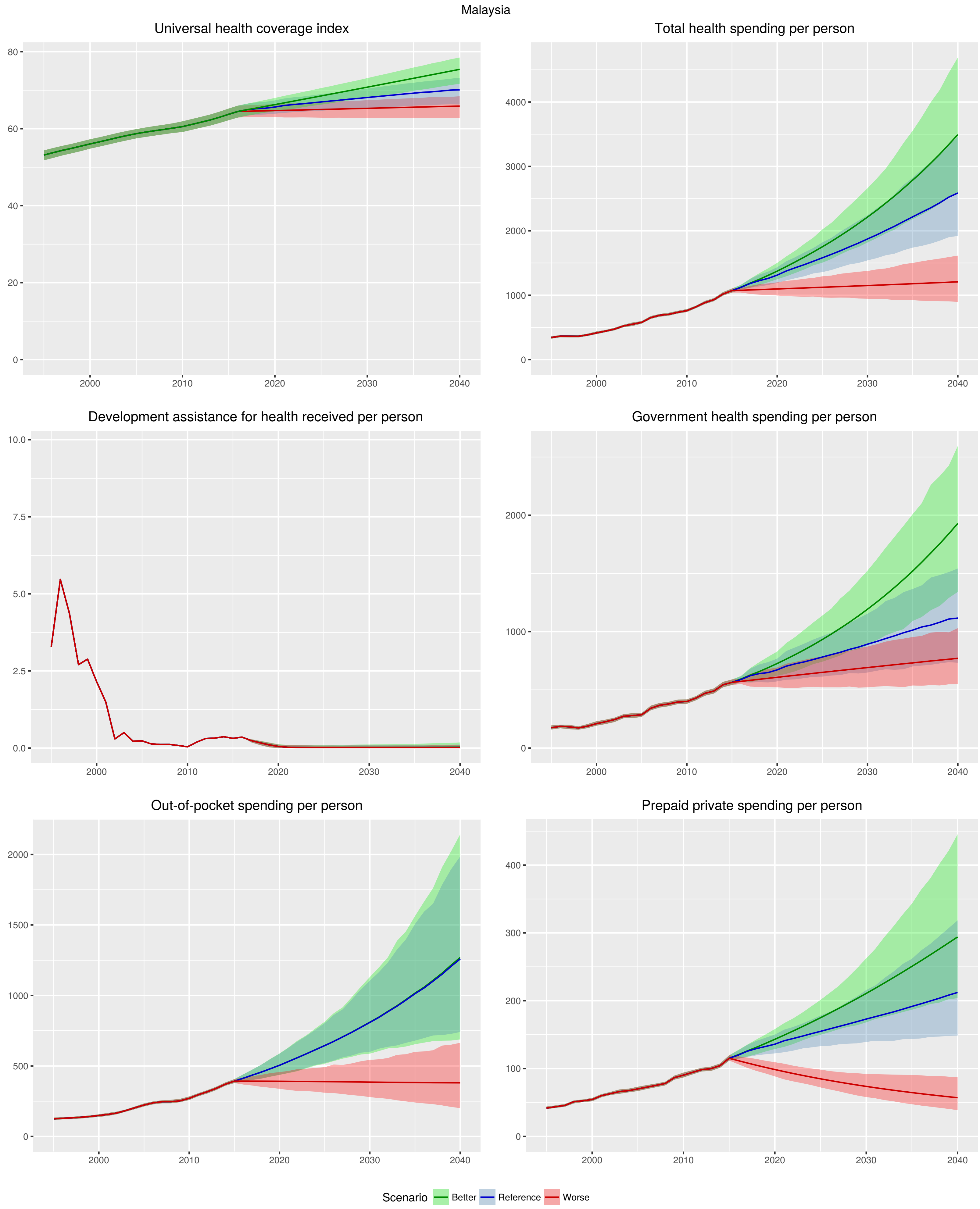
Malaysia
Universal health coverage index
Total health spending per person
Development assistance for health received per person
Government health spending per person
Out-of-pocket spending per person
Prepaid private spending per person
2000
2010
2020
2030
2040
Scenario
Better
Reference
Worse

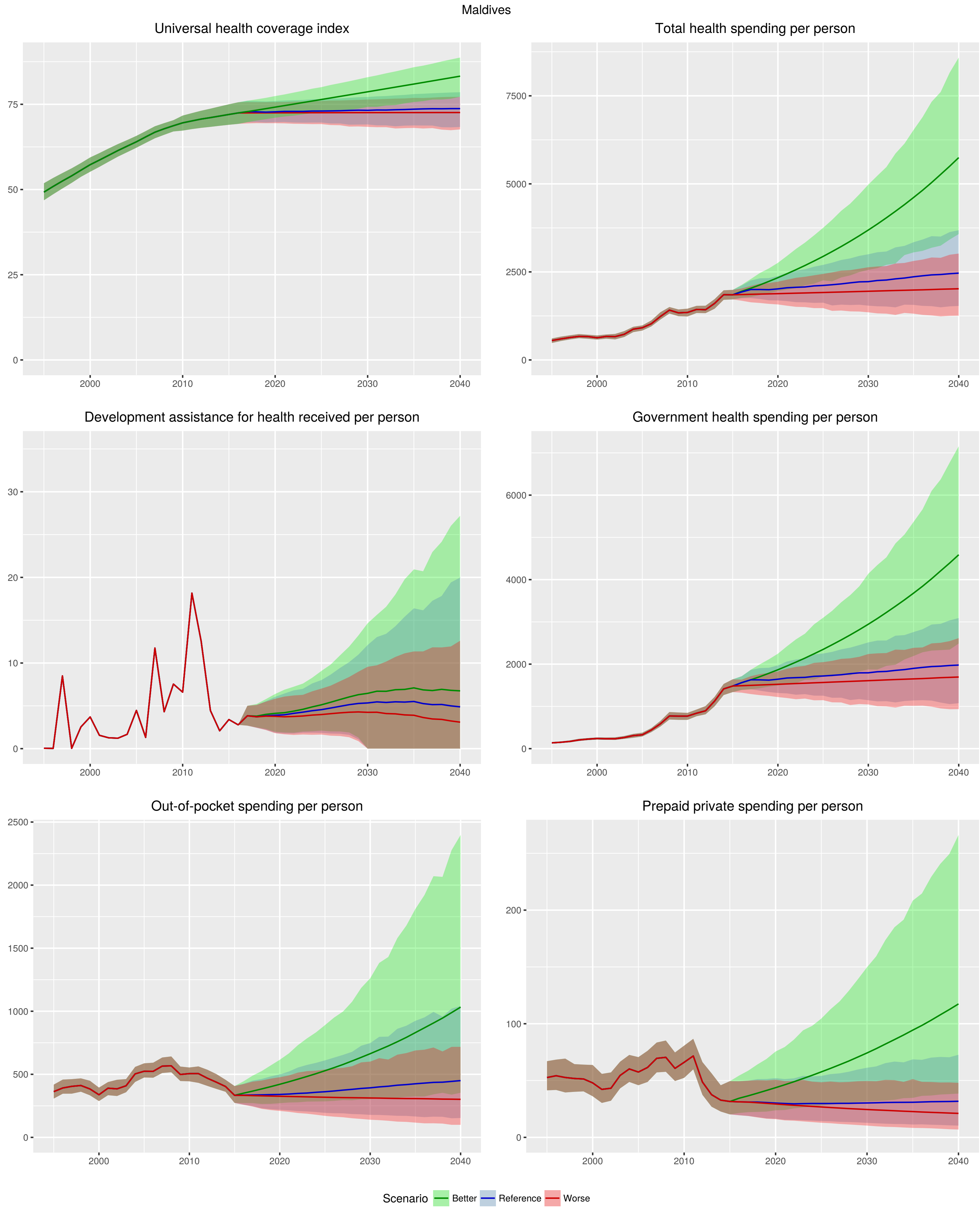

Maldives
Universal health coverage index
Total health spending per person
Development assistance for health received per person
Government health spending per person
Out-of-pocket spending per person
Prepaid private spending per person
Scenario
Better
Reference
Worse

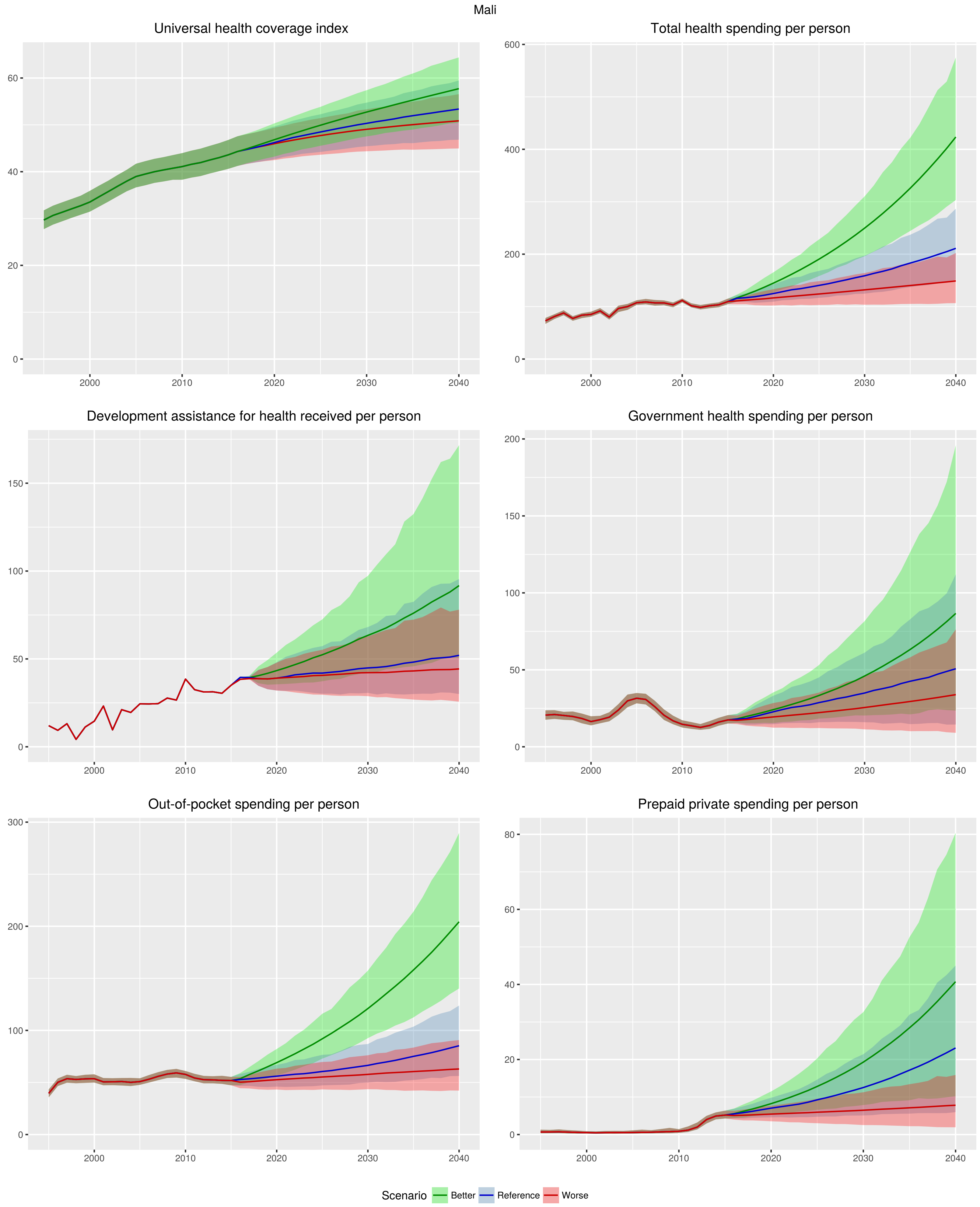
Mali
Universal health coverage index
Total health spending per person
Development assistance for health received per person
Government health spending per person
Out-of-pocket spending per person
Prepaid private spending per person
2000
2010
2020
2030
2040
Scenario
Better
Reference
Worse

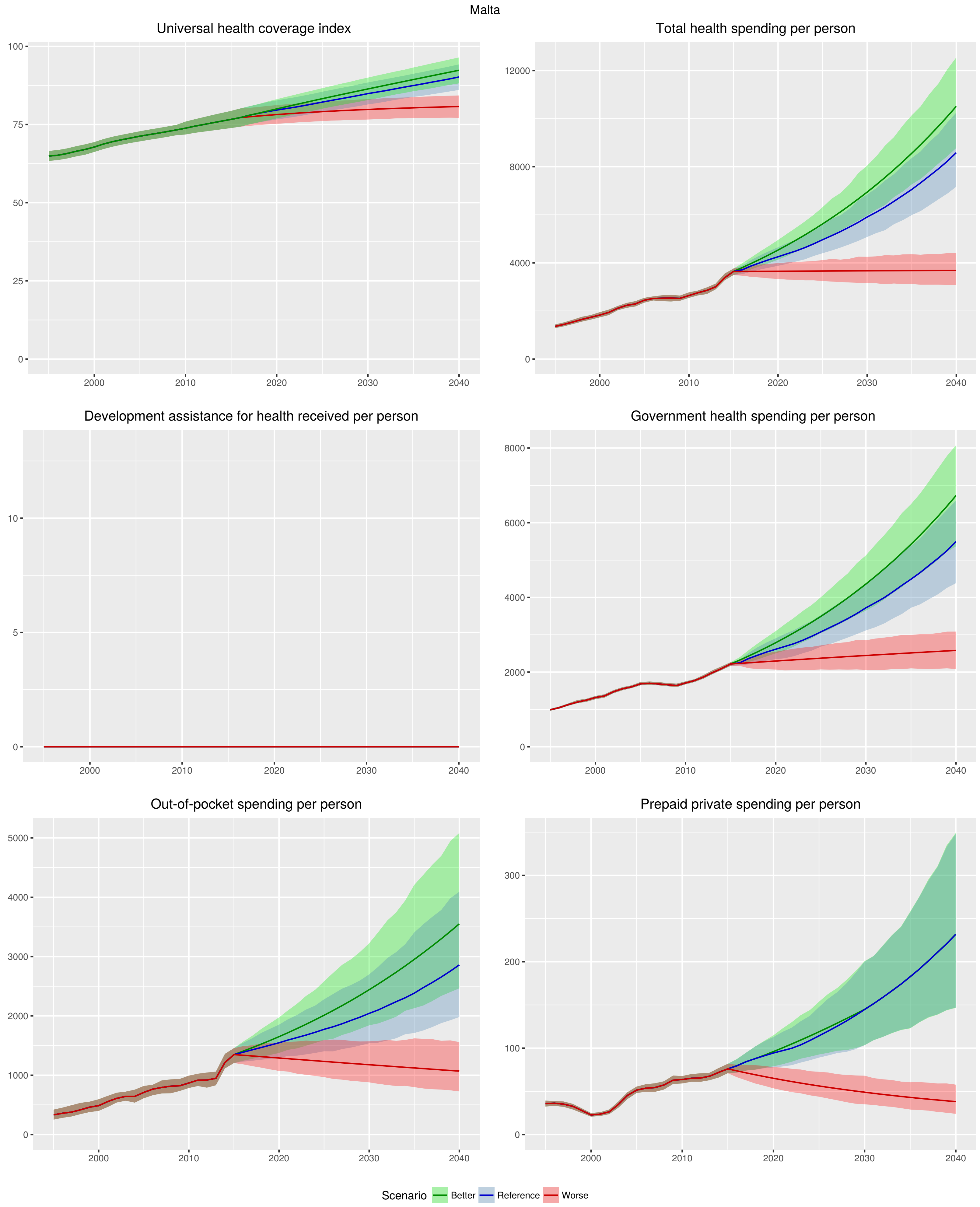
Malta
Universal health coverage index
Total health spending per person
Development assistance for health received per person
Government health spending per person
Out-of-pocket spending per person
Prepaid private spending per person
Scenario
Better
Reference
Worse

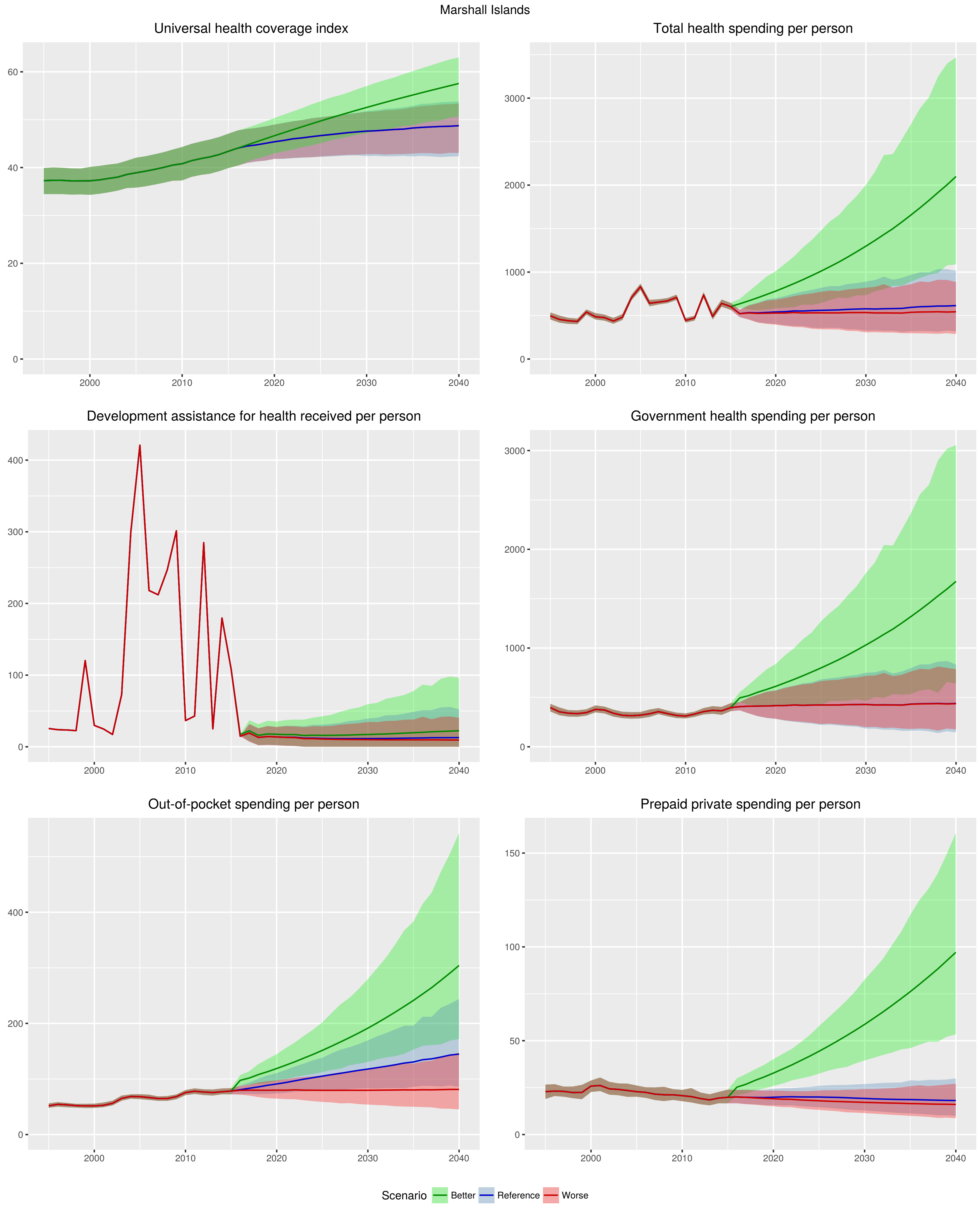

Marshall Islands
Universal health coverage index
Total health spending per person
Development assistance for health received per person
Government health spending per person
Out-of-pocket spending per person
Prepaid private spending per person
Scenario
Better
Reference
Worse

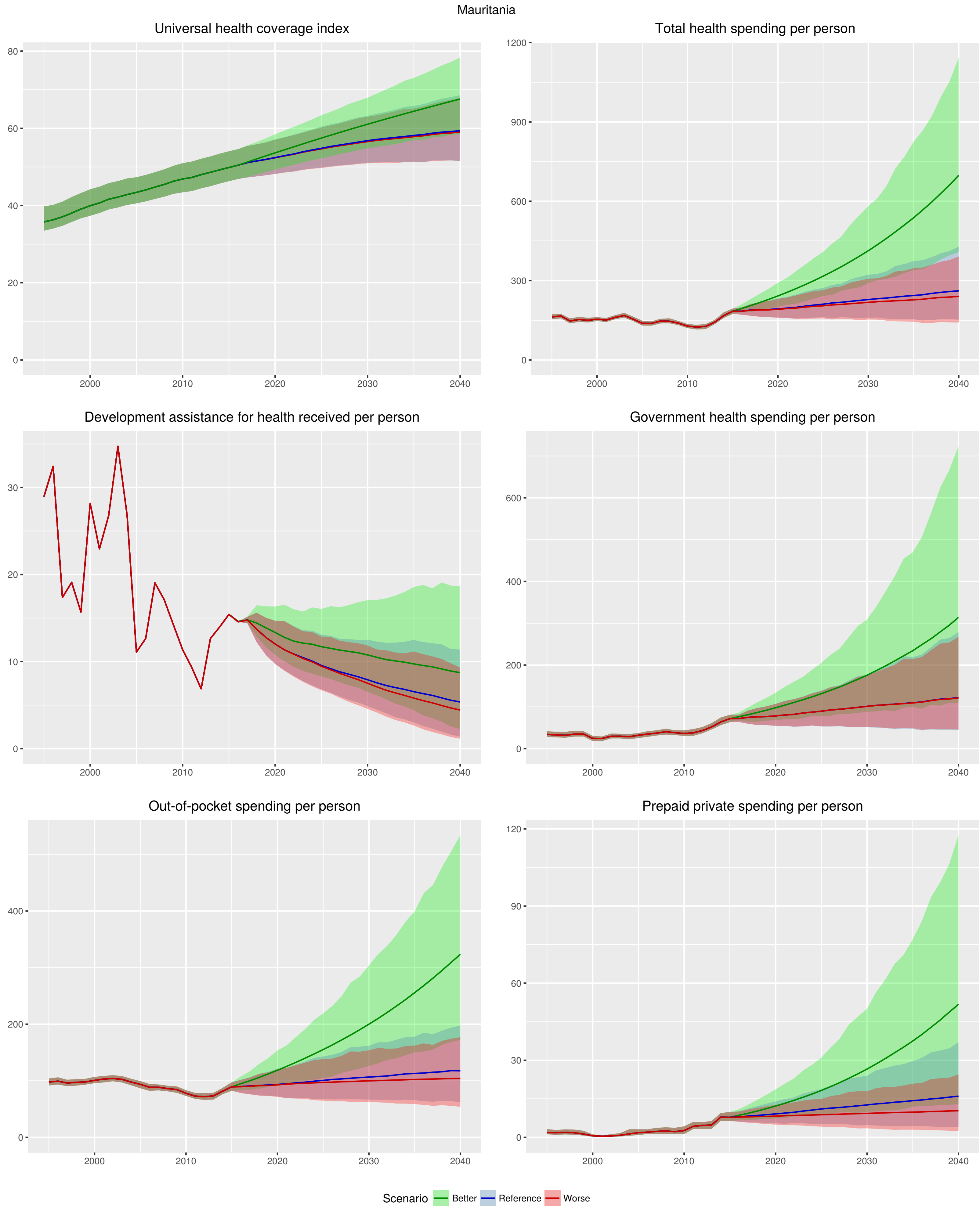
Mauritania
Universal health coverage index
0
20
40
60
80
2000
2010
2020
2030
2040
Total health spending per person
0
300
600
900
1200
2000
2010
2020
2030
2040
Development assistance for health received per person
0
10
20
30
2000
2010
2020
2030
2040
Government health spending per person
0
200
400
600
2000
2010
2020
2030
2040
Out-of-pocket spending per person
0
200
400
2000
2010
2020
2030
2040
Prepaid private spending per person
0
30
60
90
120
2000
2010
2020
2030
2040
Scenario
Better
Reference
Worse

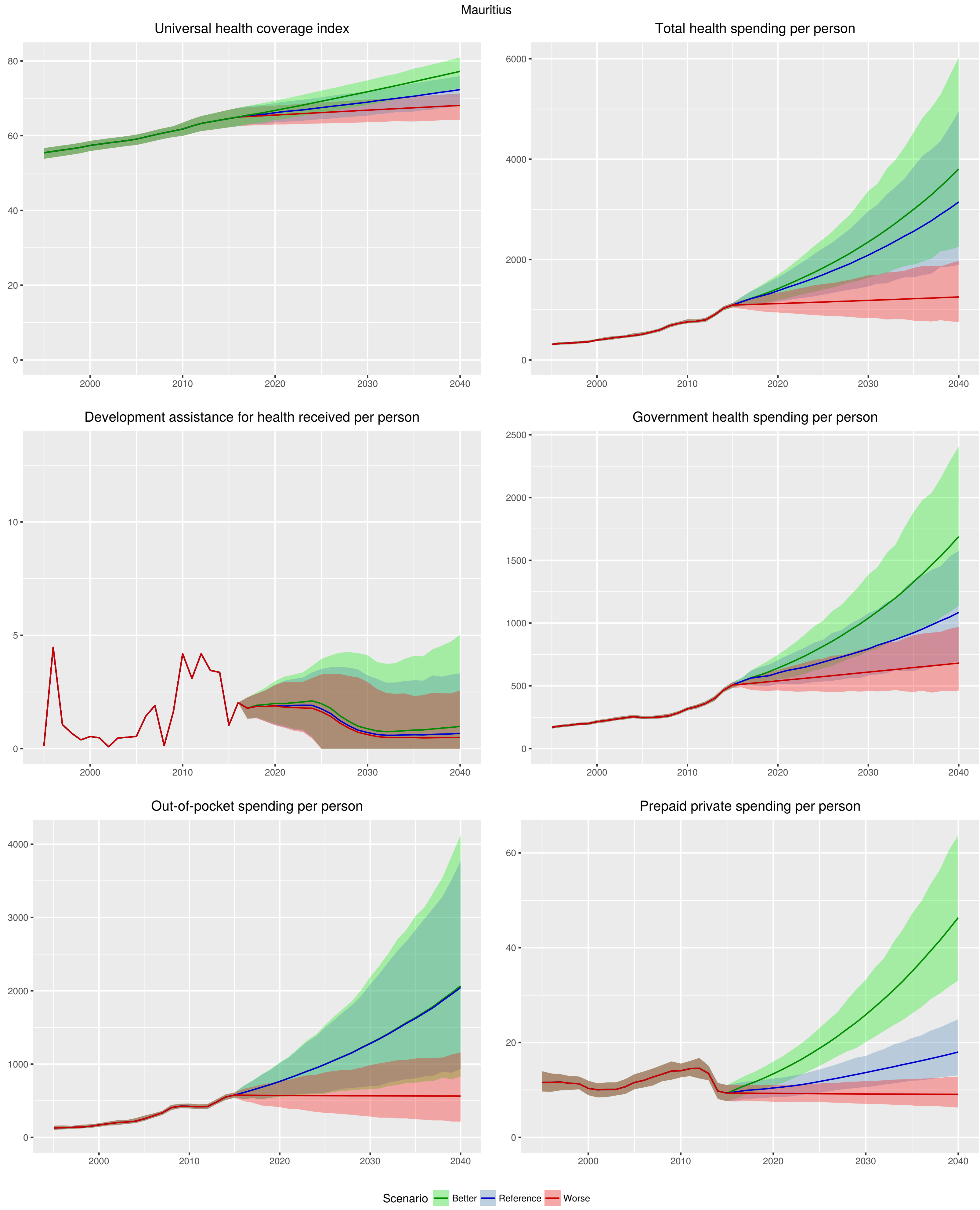
Mauritius
Universal health coverage index
Total health spending per person
Development assistance for health received per person
Government health spending per person
Out-of-pocket spending per person
Prepaid private spending per person
0
20
40
60
80
2000
4000
6000
5
10
500
1000
1500
2000
2500
3000
2010
2020
2030
2040
Scenario
Better
Reference
Worse

Mexico

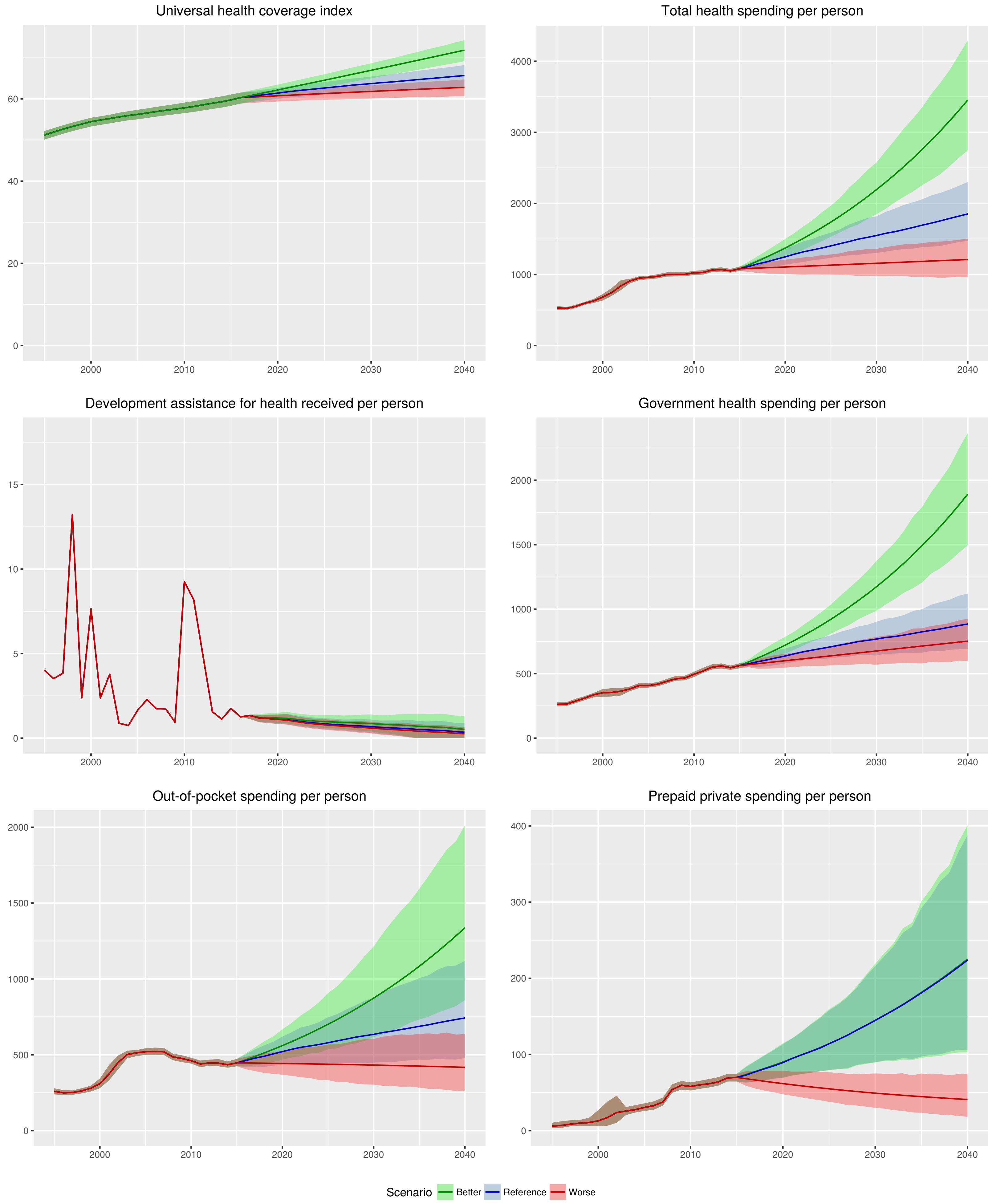
Universal health coverage index
Total health spending per person
Development assistance for health received per person
Government health spending per person
Out-of-pocket spending per person
Prepaid private spending per person
Scenario
Better
Reference
Worse

Moldova

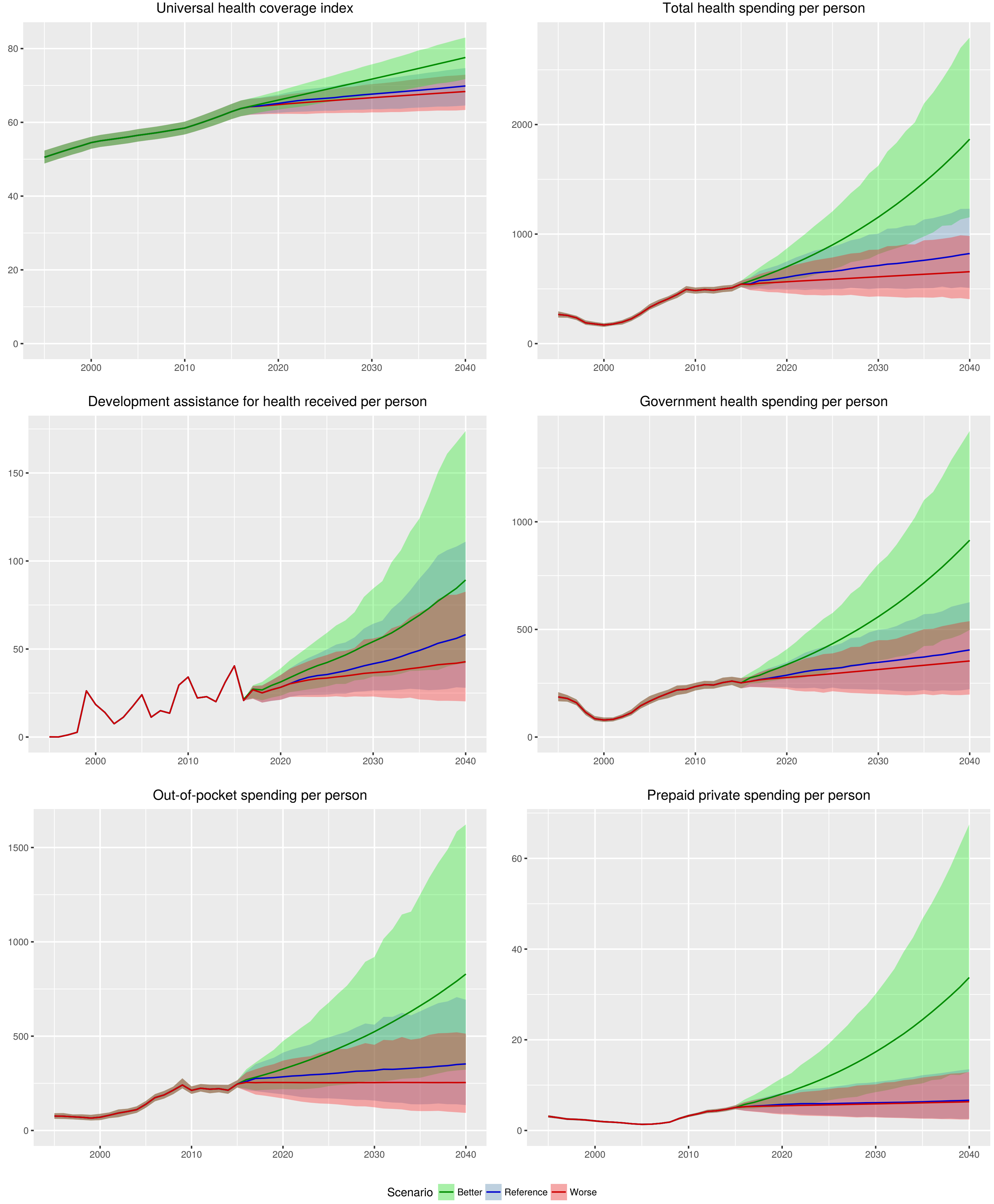

Universal health coverage index
Total health spending per person
Development assistance for health received per person
Government health spending per person
Out-of-pocket spending per person
Prepaid private spending per person
0
20
40
60
80
1000
2000
50
100
150
500
1500
2000
2010
2020
2030
2040
Scenario
Better
Reference
Worse

Mongolia

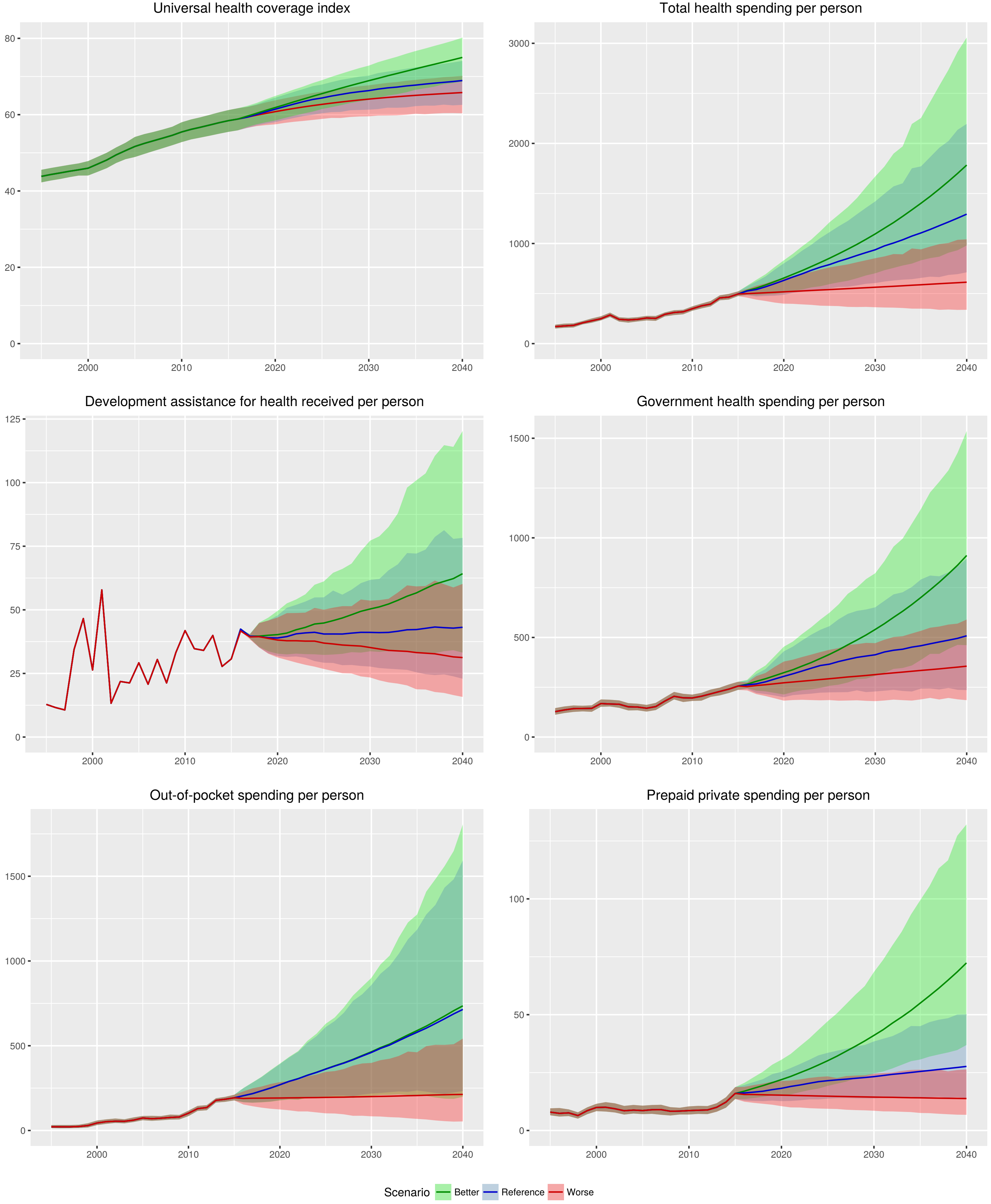

Universal health coverage index
Total health spending per person
Development assistance for health received per person
Government health spending per person
Out-of-pocket spending per person
Prepaid private spending per person
Scenario
Better
Reference
Worse

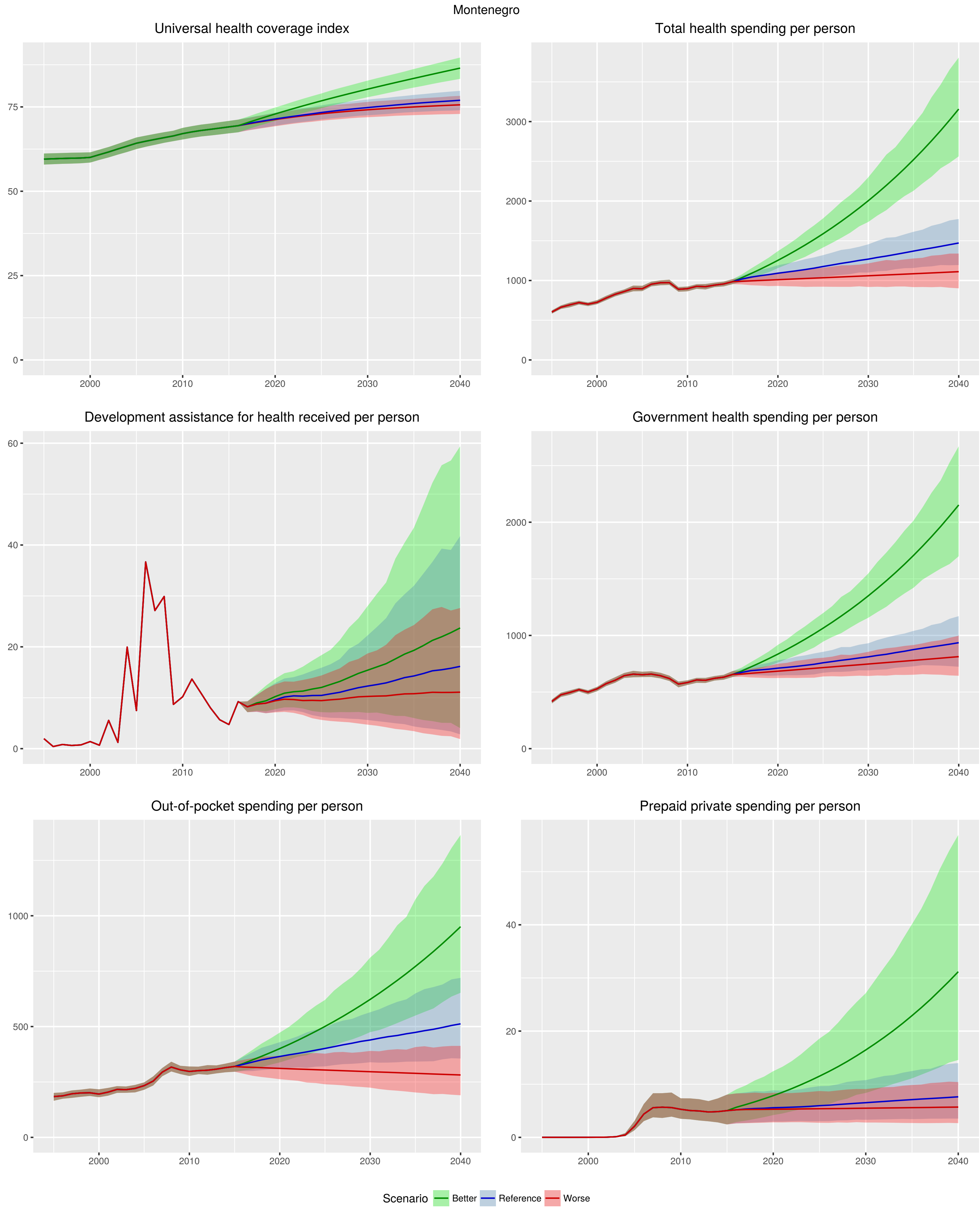

Montenegro
Universal health coverage index
Total health spending per person
Development assistance for health received per person
Government health spending per person
Out-of-pocket spending per person
Prepaid private spending per person
Scenario
Better
Reference
Worse

Morocco

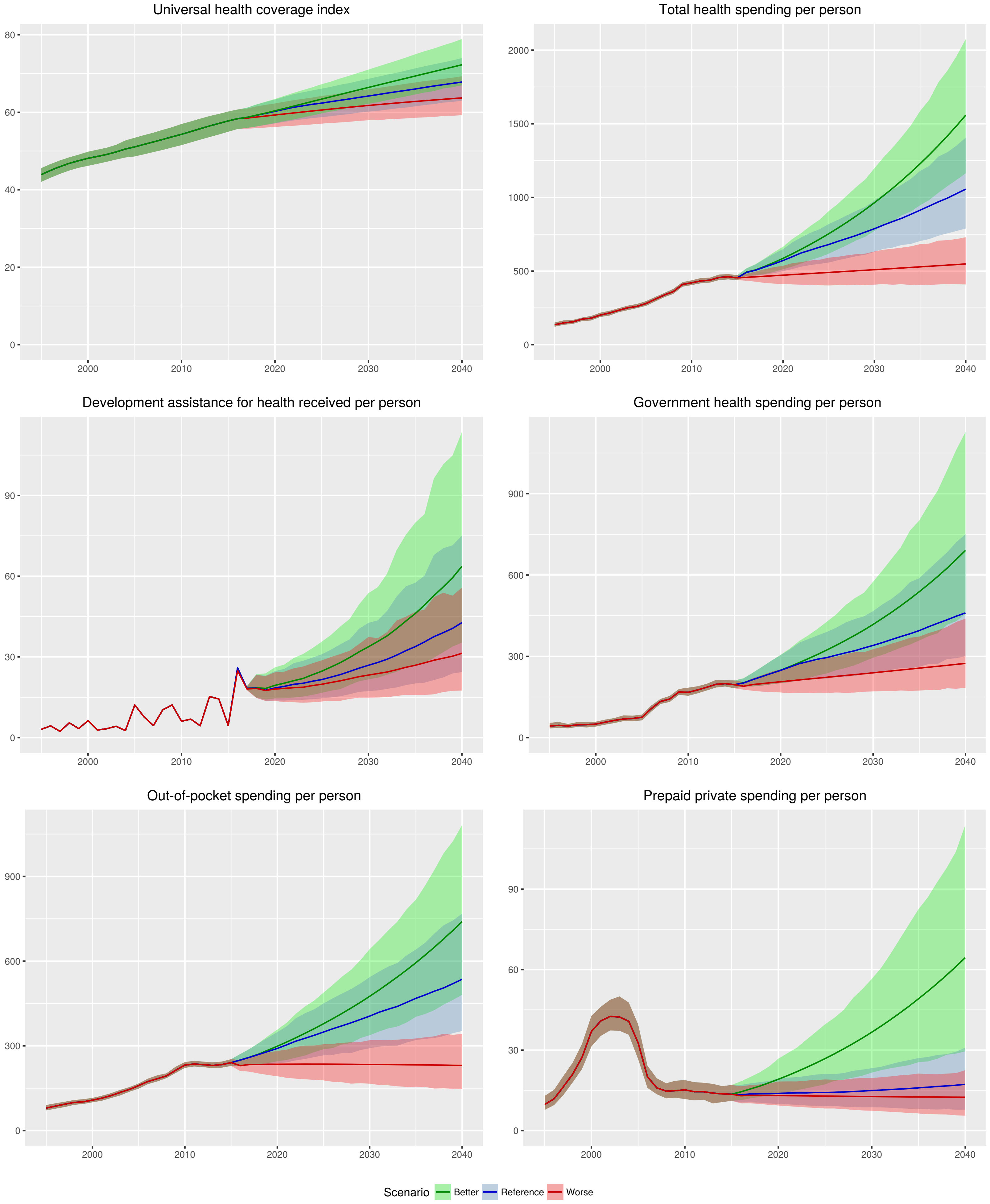
Universal health coverage index
Total health spending per person
Development assistance for health received per person
Government health spending per person
Out-of-pocket spending per person
Prepaid private spending per person
Scenario
Better
Reference
Worse

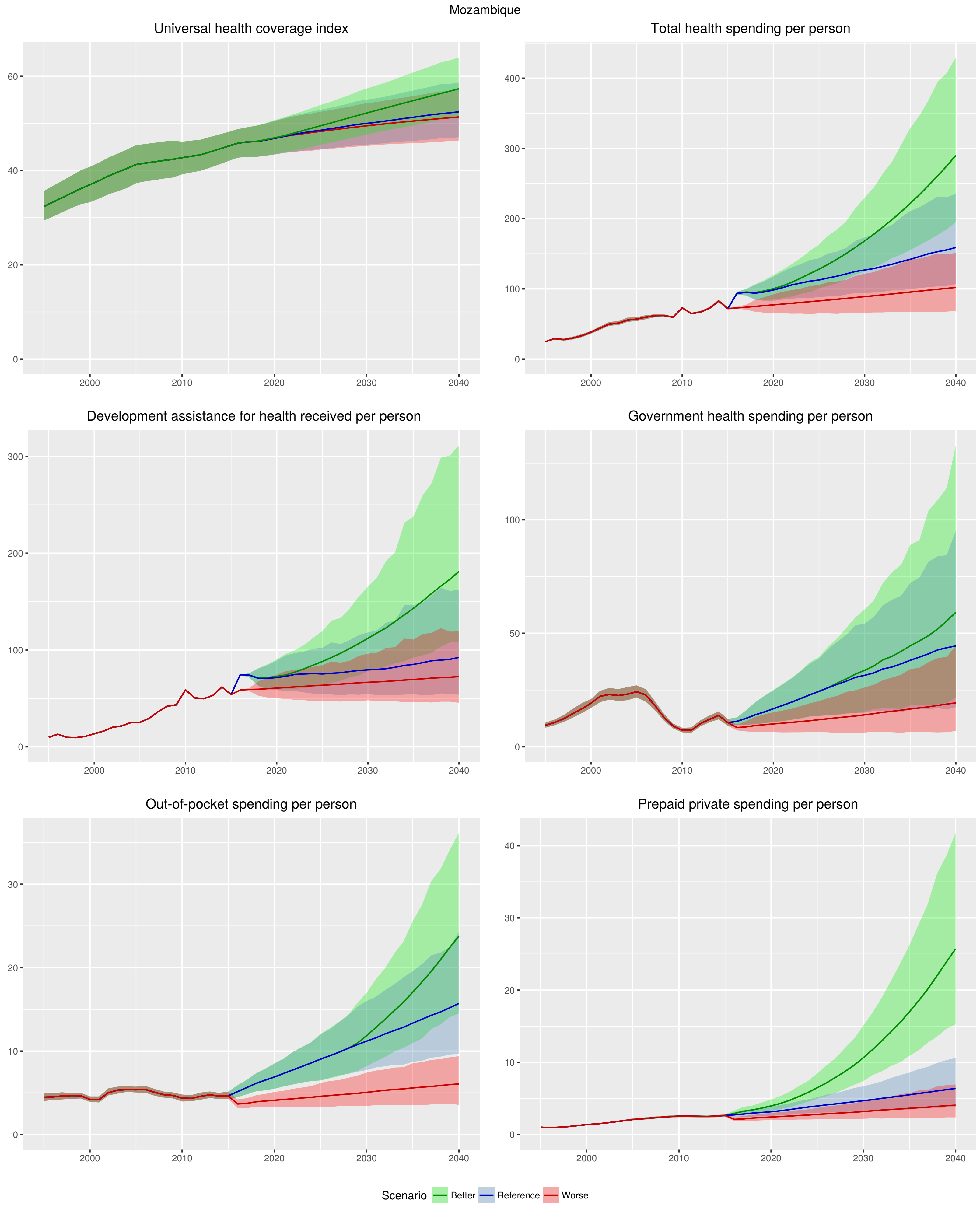
Mozambique
Universal health coverage index
0
20
40
60
2000
2010
2020
2030
2040
Total health spending per person
0
100
200
300
400
Development assistance for health received per person
0
100
200
300
Government health spending per person
0
50
100
Out-of-pocket spending per person
0
10
20
30
Prepaid private spending per person
0
10
20
30
40
Scenario
Better
Reference
Worse

Myanmar

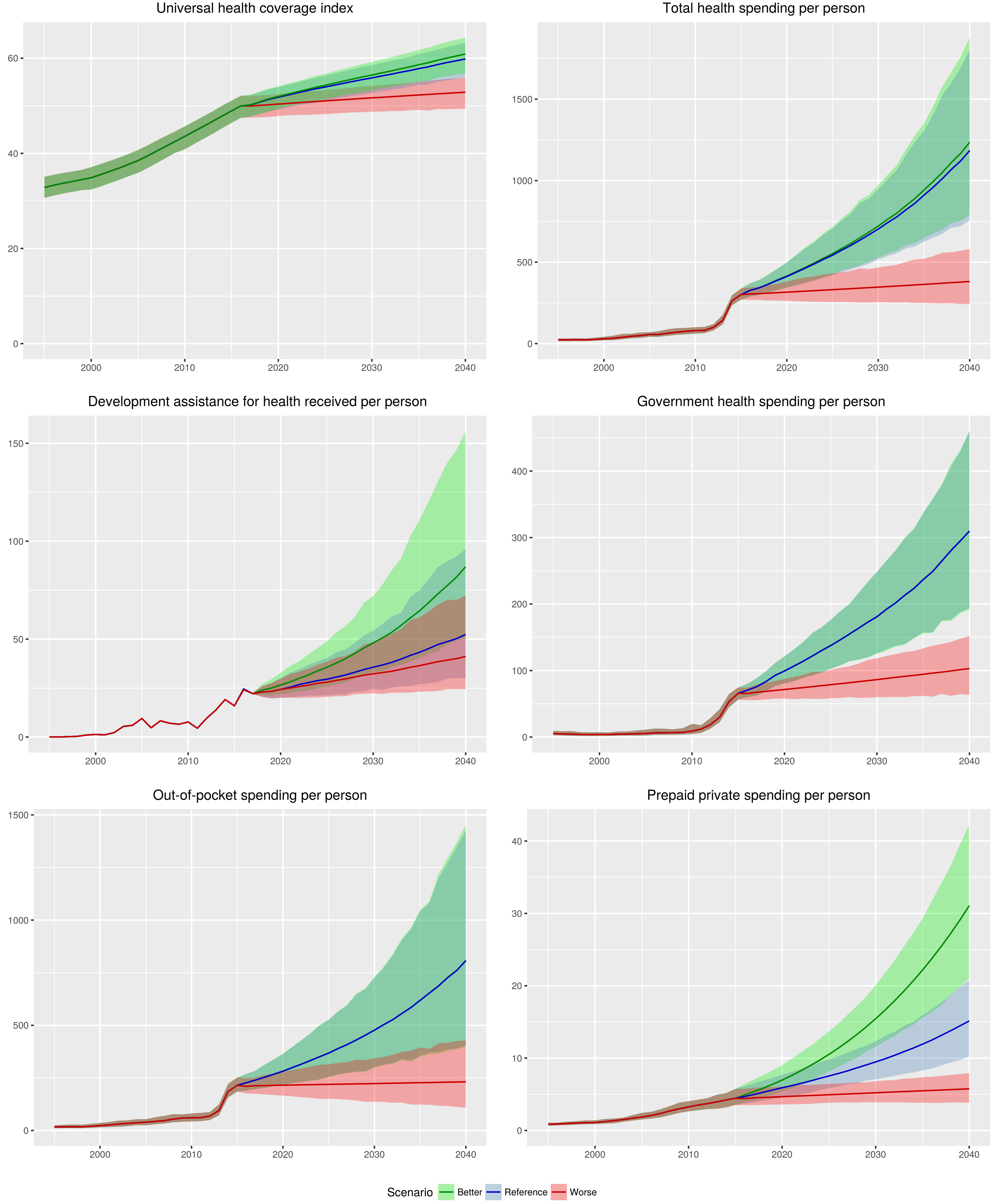

Universal health coverage index
Total health spending per person
Development assistance for health received per person
Government health spending per person
Out-of-pocket spending per person
Prepaid private spending per person
Scenario
Better
Reference
Worse

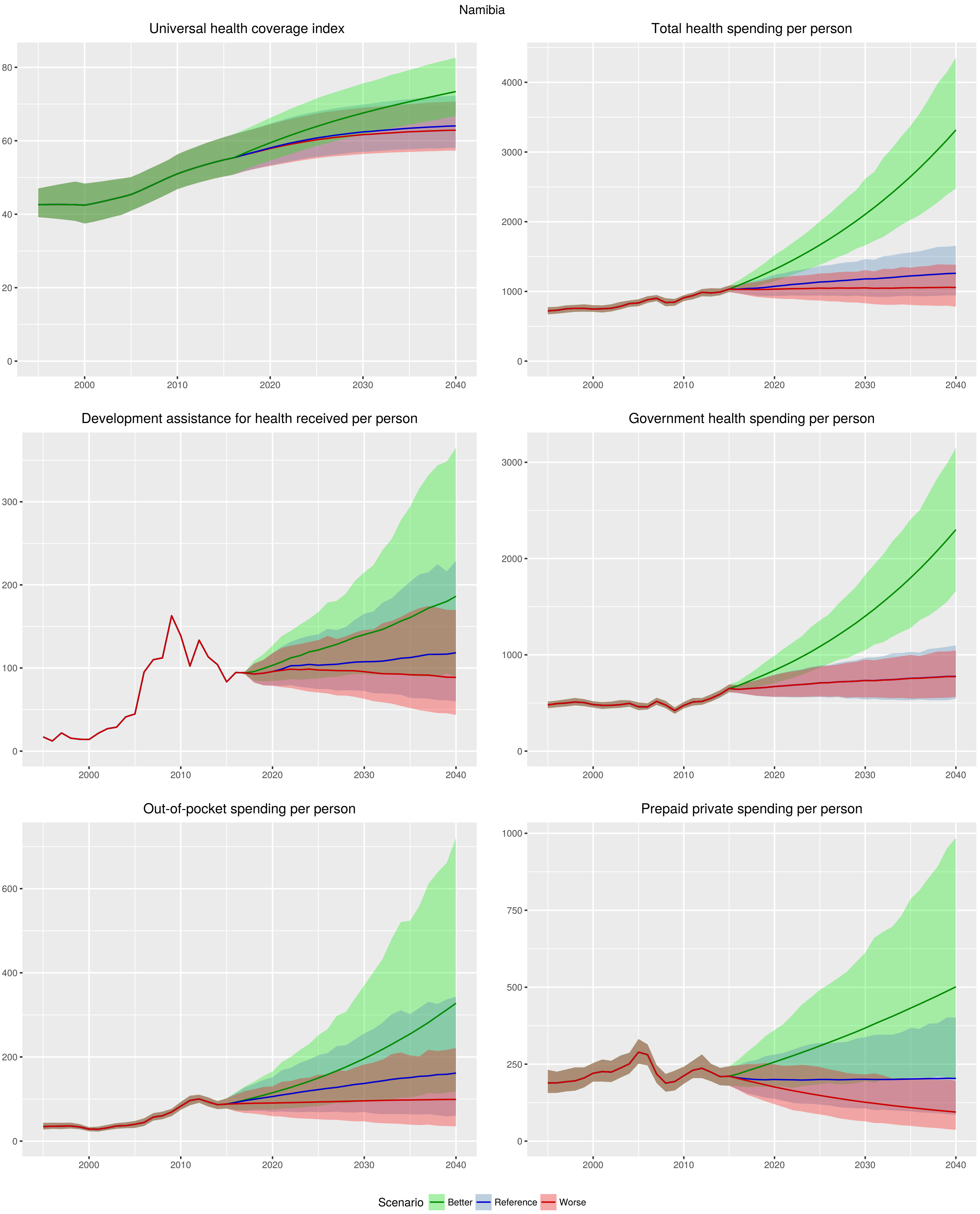

Namibia
Universal health coverage index
Total health spending per person
Development assistance for health received per person
Government health spending per person
Out-of-pocket spending per person
Prepaid private spending per person
Scenario
Better
Reference
Worse

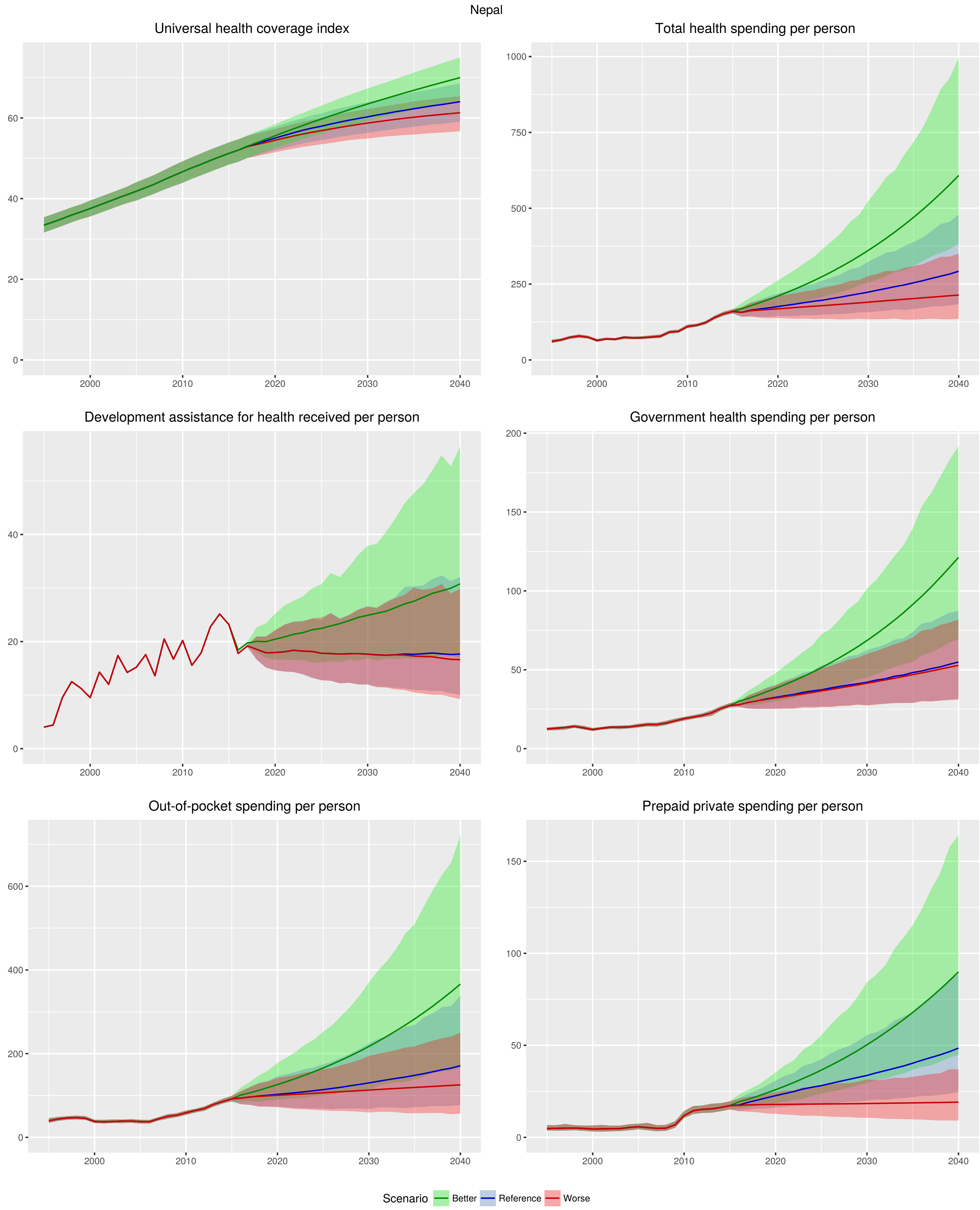
Nepal
Universal health coverage index
Total health spending per person
Development assistance for health received per person
Government health spending per person
Out-of-pocket spending per person
Prepaid private spending per person
0
20
40
60
0
250
500
750
1000
0
20
40
0
50
100
150
200
0
200
400
600
0
50
100
150
2000
2010
2020
2030
2040
Scenario
Better
Reference
Worse

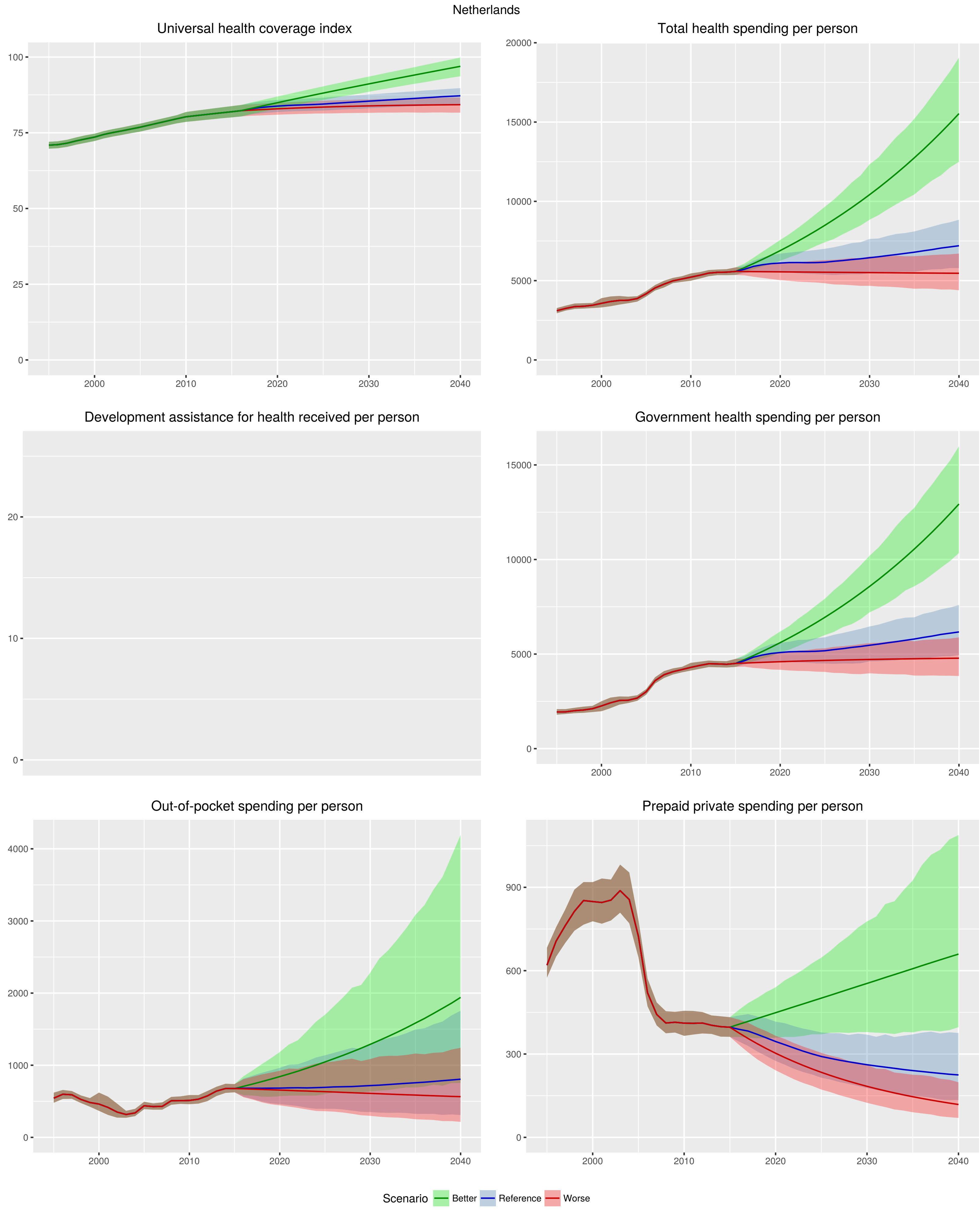
Netherlands
Universal health coverage index
100
75
50
25
0
2000
2010
2020
2030
2040
Total health spending per person
20000
15000
10000
5000
0
Development assistance for health received per person
20
10
0
Government health spending per person
15000
10000
5000
0
Out-of-pocket spending per person
4000
3000
2000
1000
0
Prepaid private spending per person
900
600
300
0
Scenario
Better
Reference
Worse

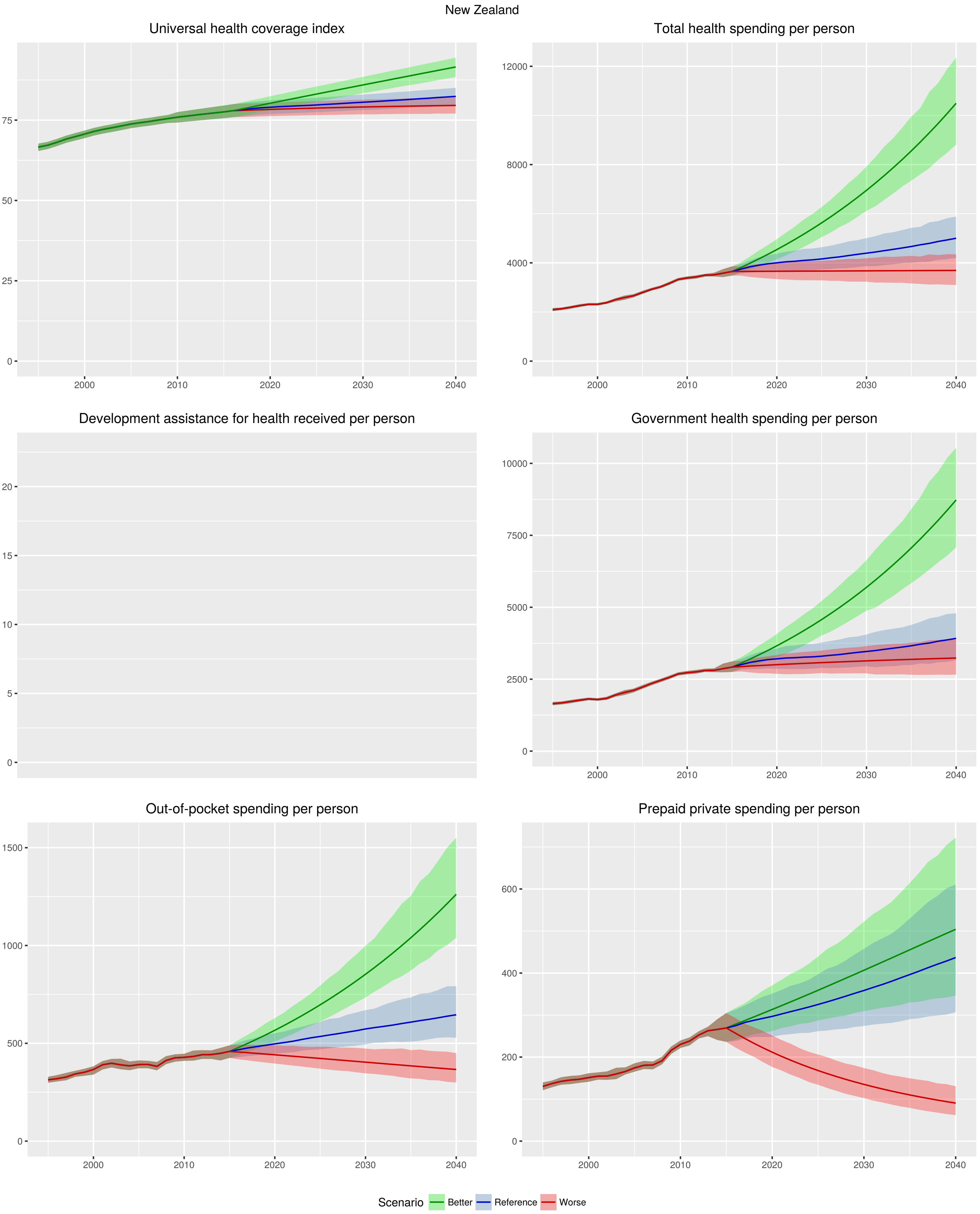

New Zealand
Universal health coverage index
Total health spending per person
Development assistance for health received per person
Government health spending per person
Out-of-pocket spending per person
Prepaid private spending per person
0
25
50
75
0
4000
8000
12000
0
5
10
15
20
0
2500
5000
7500
10000
0
500
1000
1500
0
200
400
600
2000
2010
2020
2030
2040
Scenario
Better
Reference
Worse

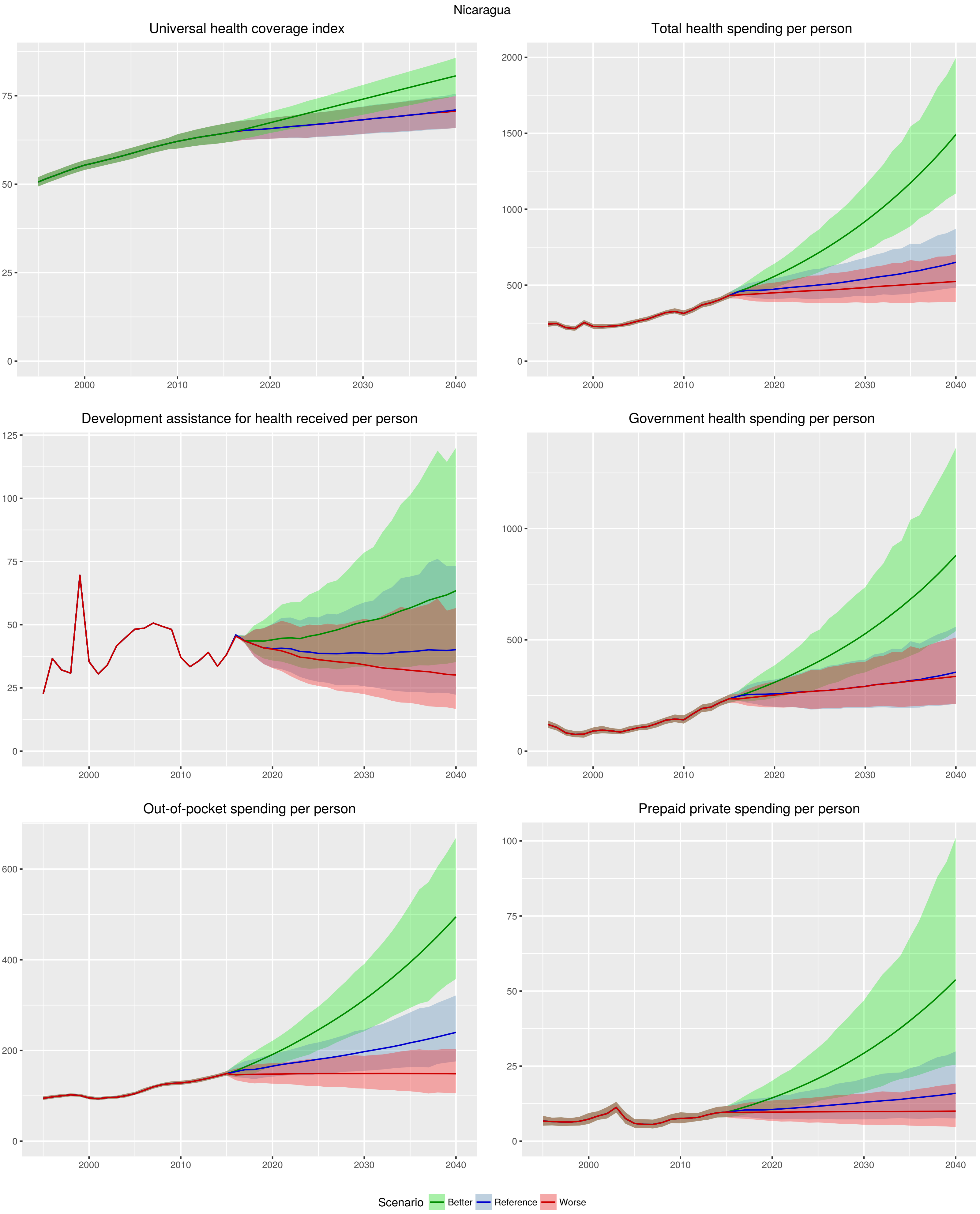

Nicaragua
Universal health coverage index
Total health spending per person
Development assistance for health received per person
Government health spending per person
Out-of-pocket spending per person
Prepaid private spending per person
Scenario
Better
Reference
Worse

Niger

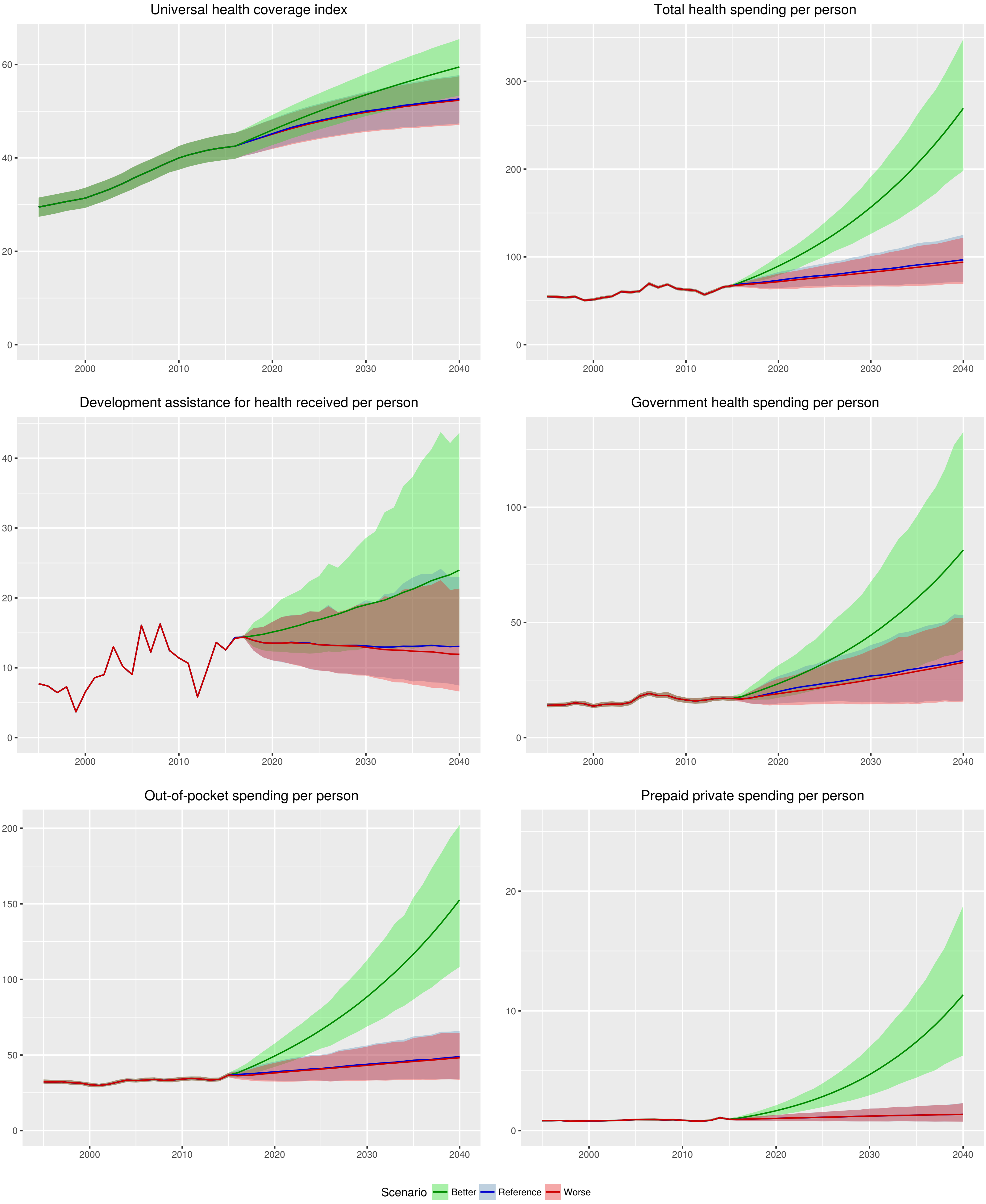
Universal health coverage index
0
20
40
60
2000
2010
2020
2030
2040
Total health spending per person
0
100
200
300
2000
2010
2020
2030
2040
Development assistance for health received per person
0
10
20
30
40
2000
2010
2020
2030
2040
Government health spending per person
0
50
100
2000
2010
2020
2030
2040
Out-of-pocket spending per person
0
50
100
150
200
2000
2010
2020
2030
2040
Prepaid private spending per person
0
10
20
2000
2010
2020
2030
2040
Scenario
Better
Reference
Worse

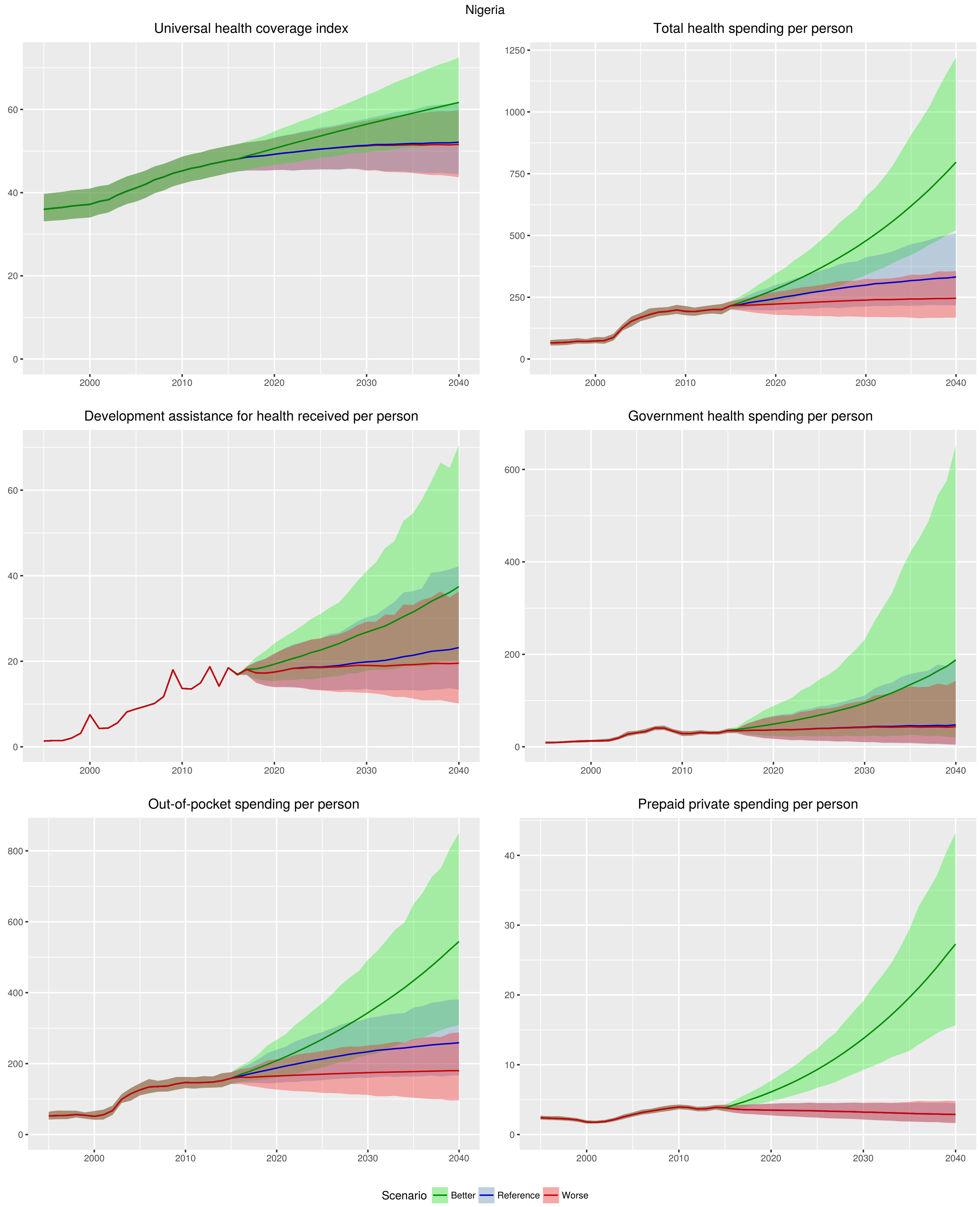
Nigeria
Universal health coverage index
Total health spending per person
Development assistance for health received per person
Government health spending per person
Out-of-pocket spending per person
Prepaid private spending per person
2000
2010
2020
2030
2040
Scenario
Better
Reference
Worse

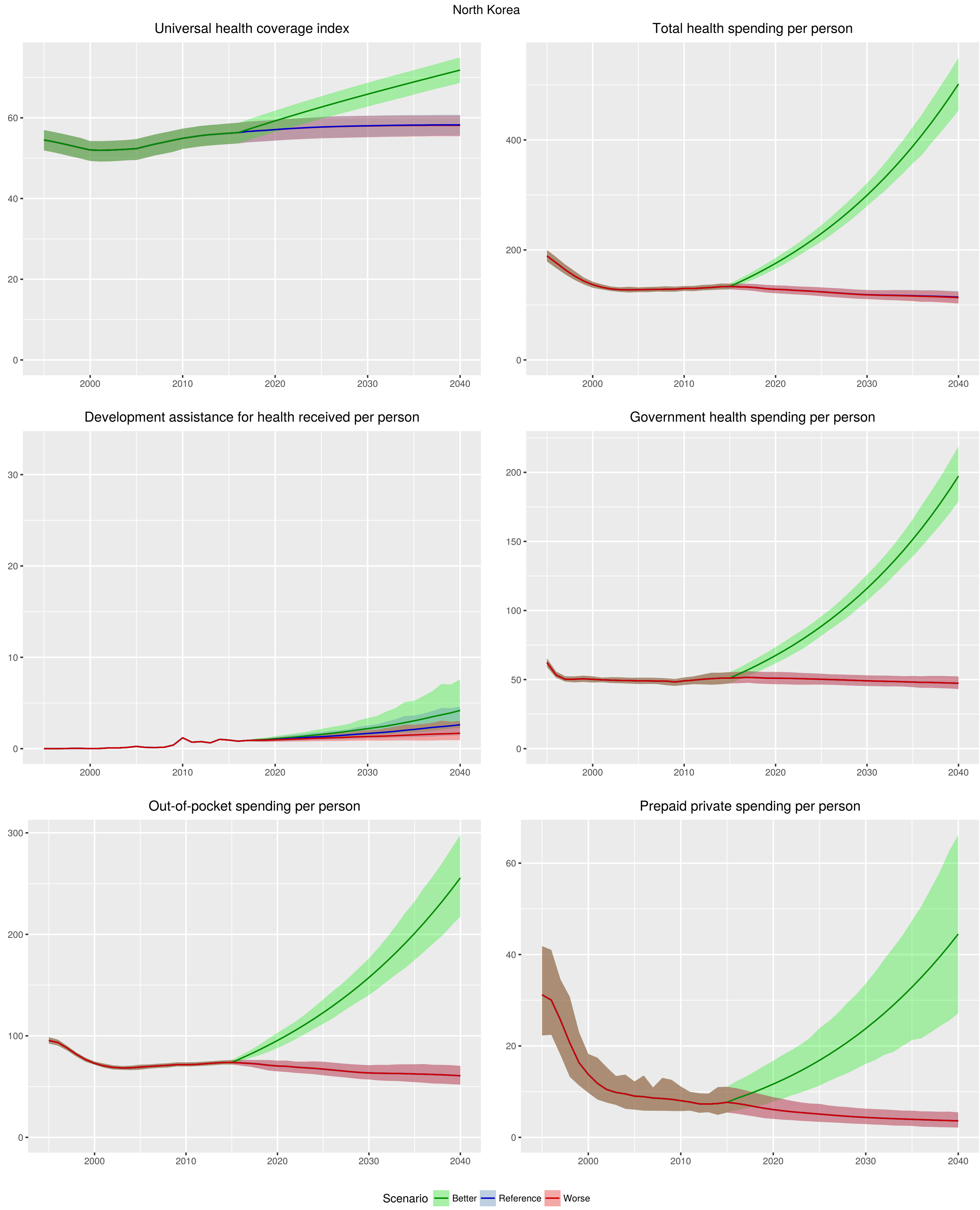

North Korea
Universal health coverage index
Total health spending per person
Development assistance for health received per person
Government health spending per person
Out-of-pocket spending per person
Prepaid private spending per person
Scenario
Better
Reference
Worse

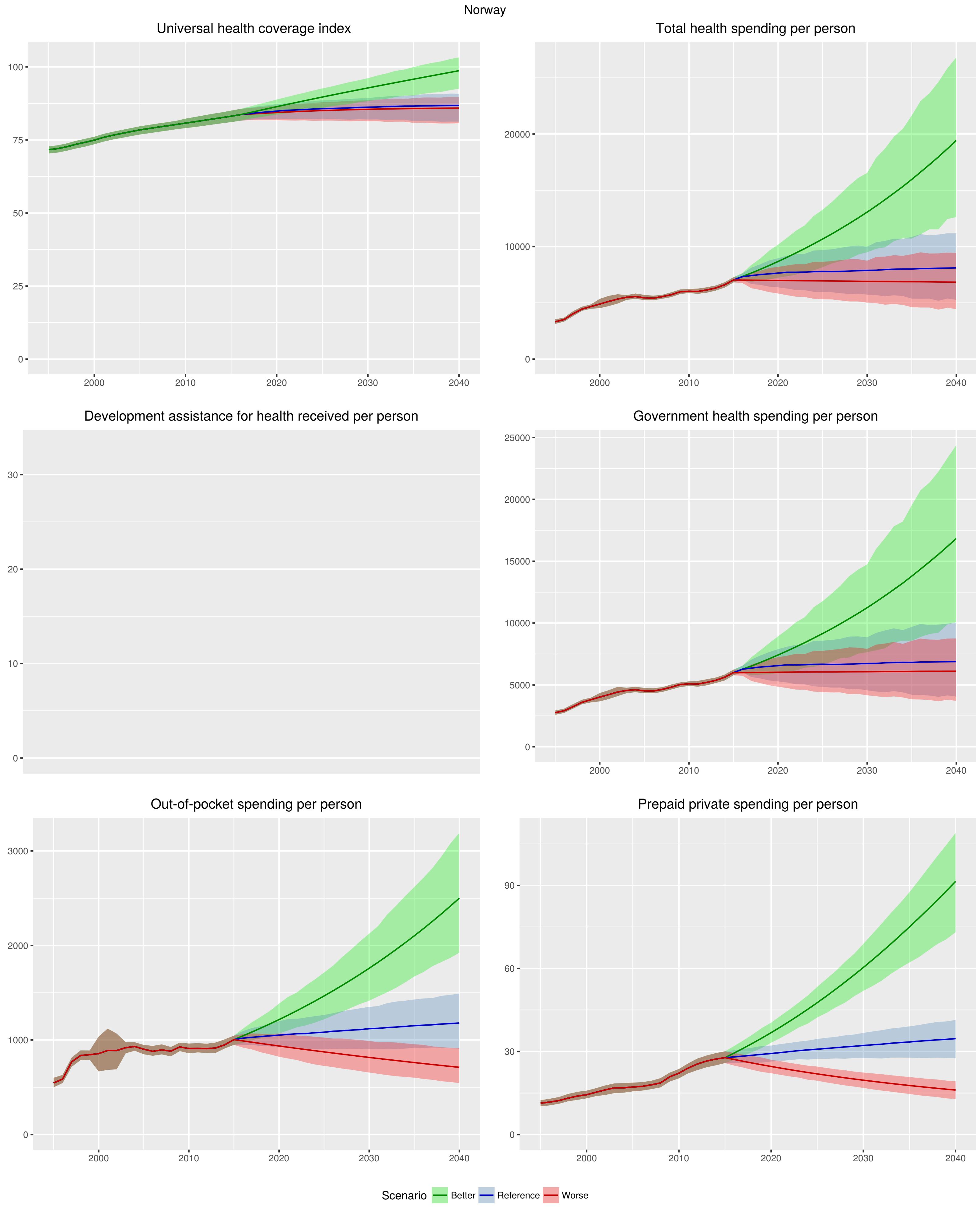

Norway
Universal health coverage index
Total health spending per person
Development assistance for health received per person
Government health spending per person
Out-of-pocket spending per person
Prepaid private spending per person
Scenario
Better
Reference
Worse

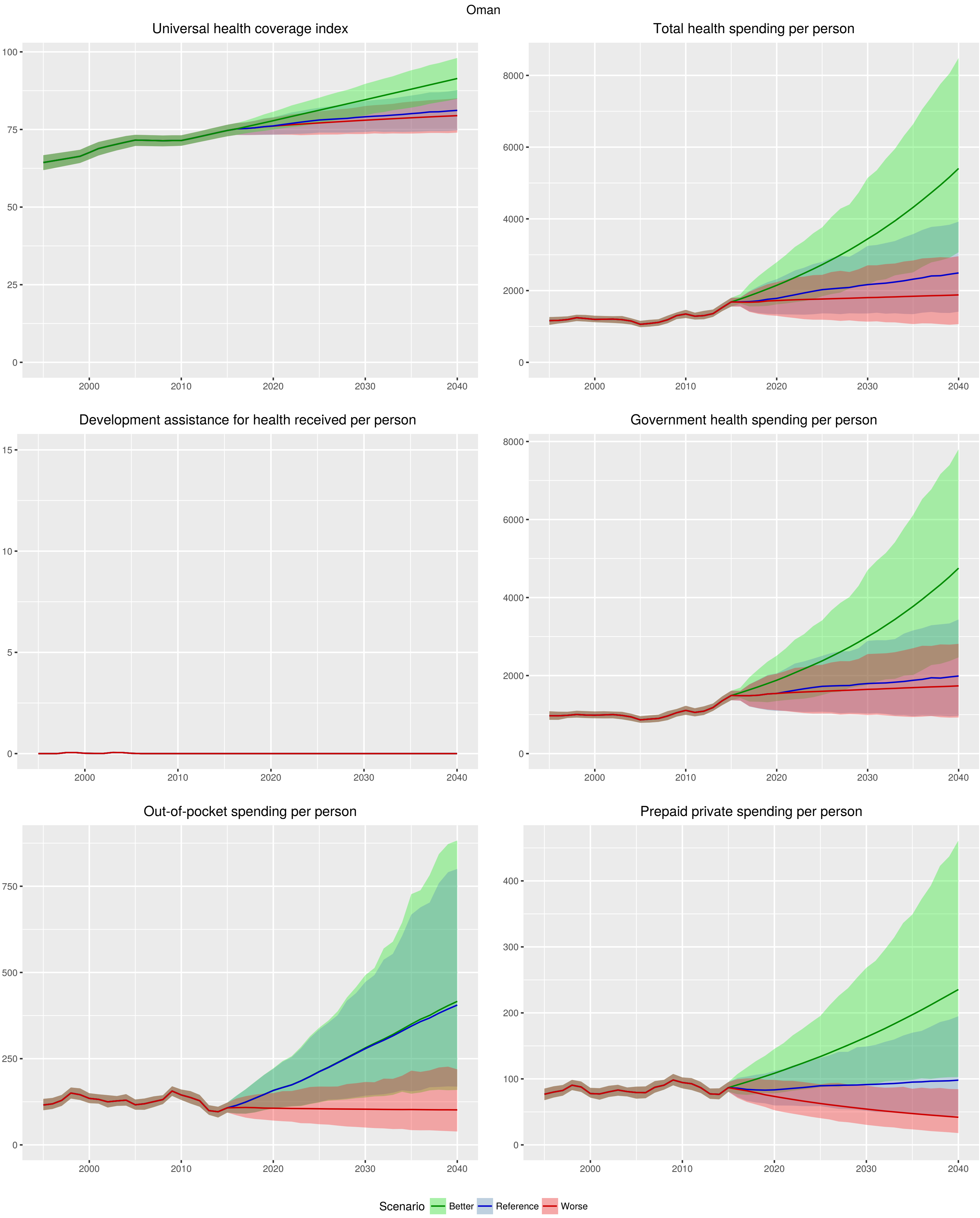
Oman
Universal health coverage index
Total health spending per person
Development assistance for health received per person
Government health spending per person
Out-of-pocket spending per person
Prepaid private spending per person
0
25
50
75
100
2000
2010
2020
2030
2040
2000
4000
6000
8000
5
10
15
250
500
750
200
300
400
Scenario
Better
Reference
Worse

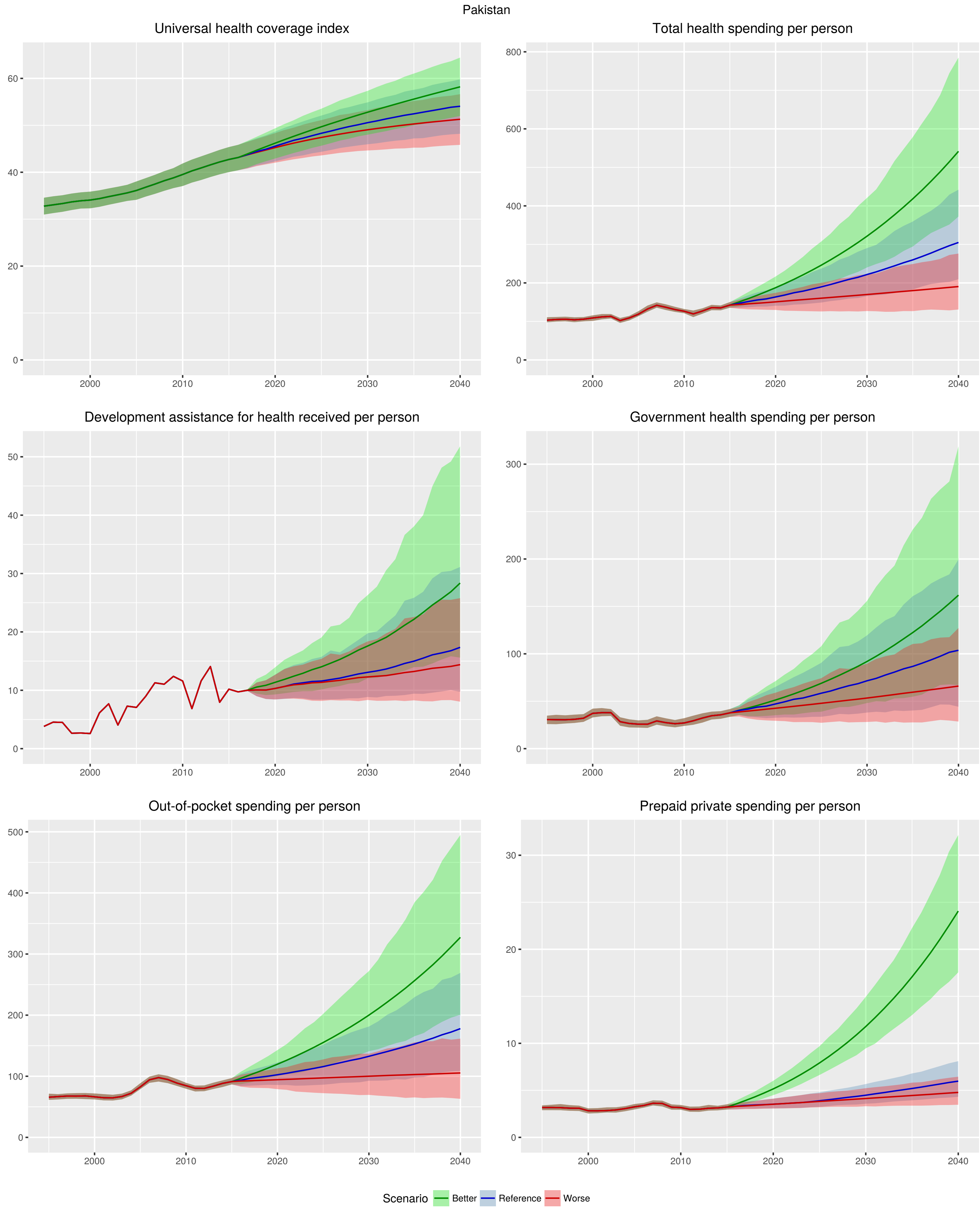

Pakistan
Universal health coverage index
Total health spending per person
Development assistance for health received per person
Government health spending per person
Out-of-pocket spending per person
Prepaid private spending per person
0
20
40
60
200
400
600
800
10
30
50
100
300
500
2000
2010
2020
2030
2040
Scenario
Better
Reference
Worse

Palestine

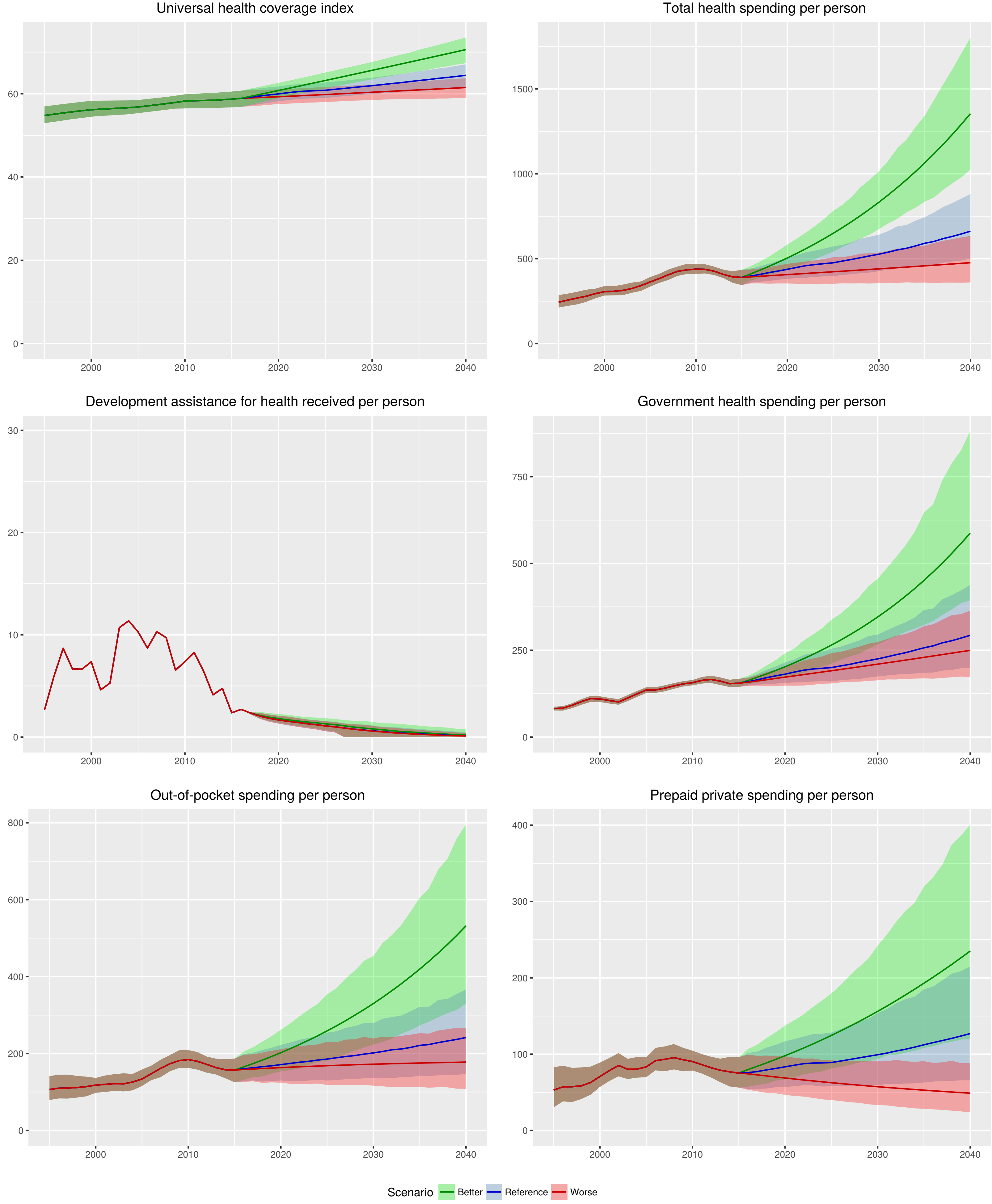

Universal health coverage index
Total health spending per person
Development assistance for health received per person
Government health spending per person
Out-of-pocket spending per person
Prepaid private spending per person
0
20
40
60
500
1000
1500
10
30
250
750
200
400
600
800
100
300
2000
2010
2020
2030
2040
Scenario
Better
Reference
Worse

Panama

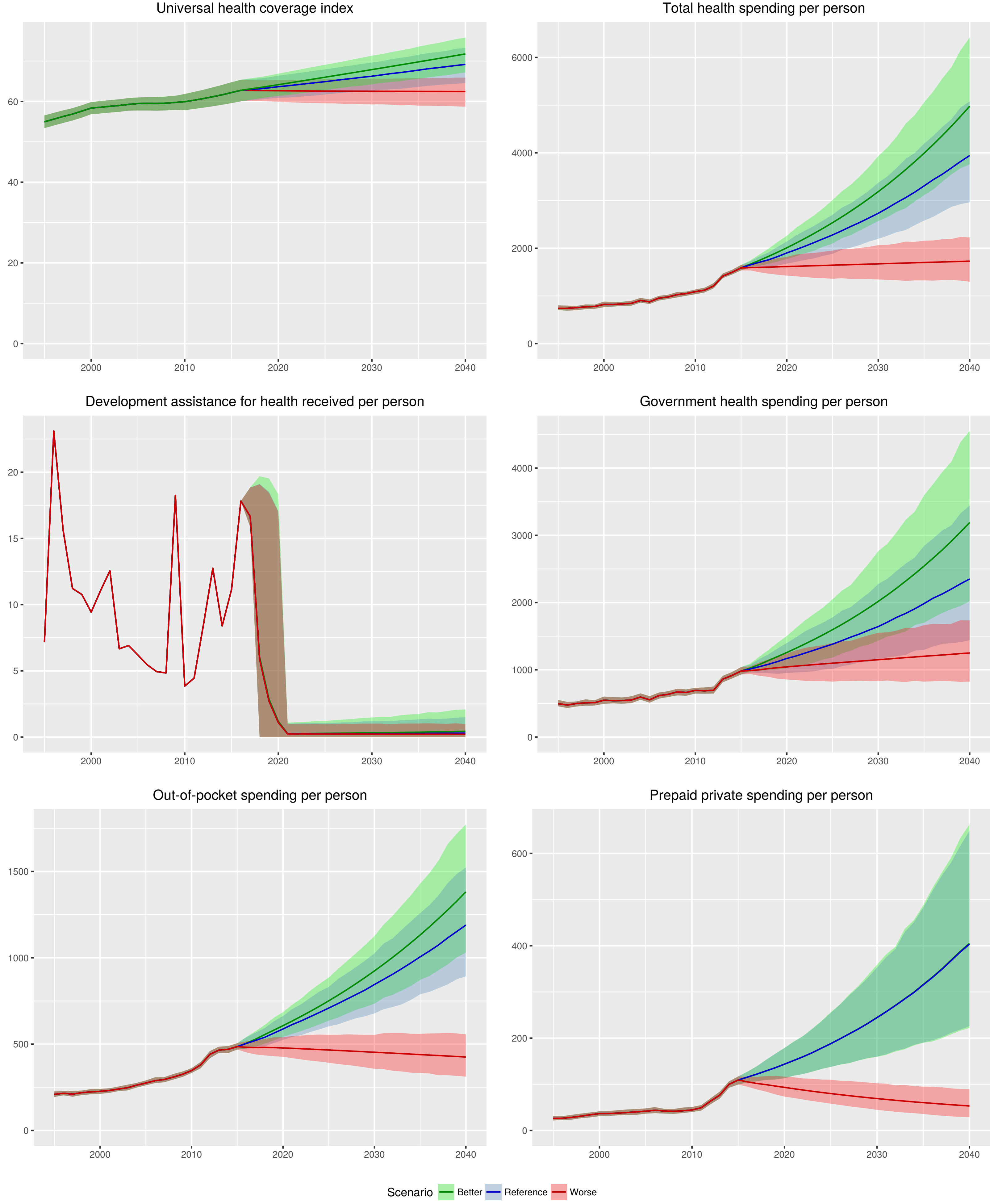
Universal health coverage index
Total health spending per person
Development assistance for health received per person
Government health spending per person
Out-of-pocket spending per person
Prepaid private spending per person
2000
2010
2020
2030
2040
Scenario
Better
Reference
Worse

Papua New Guinea

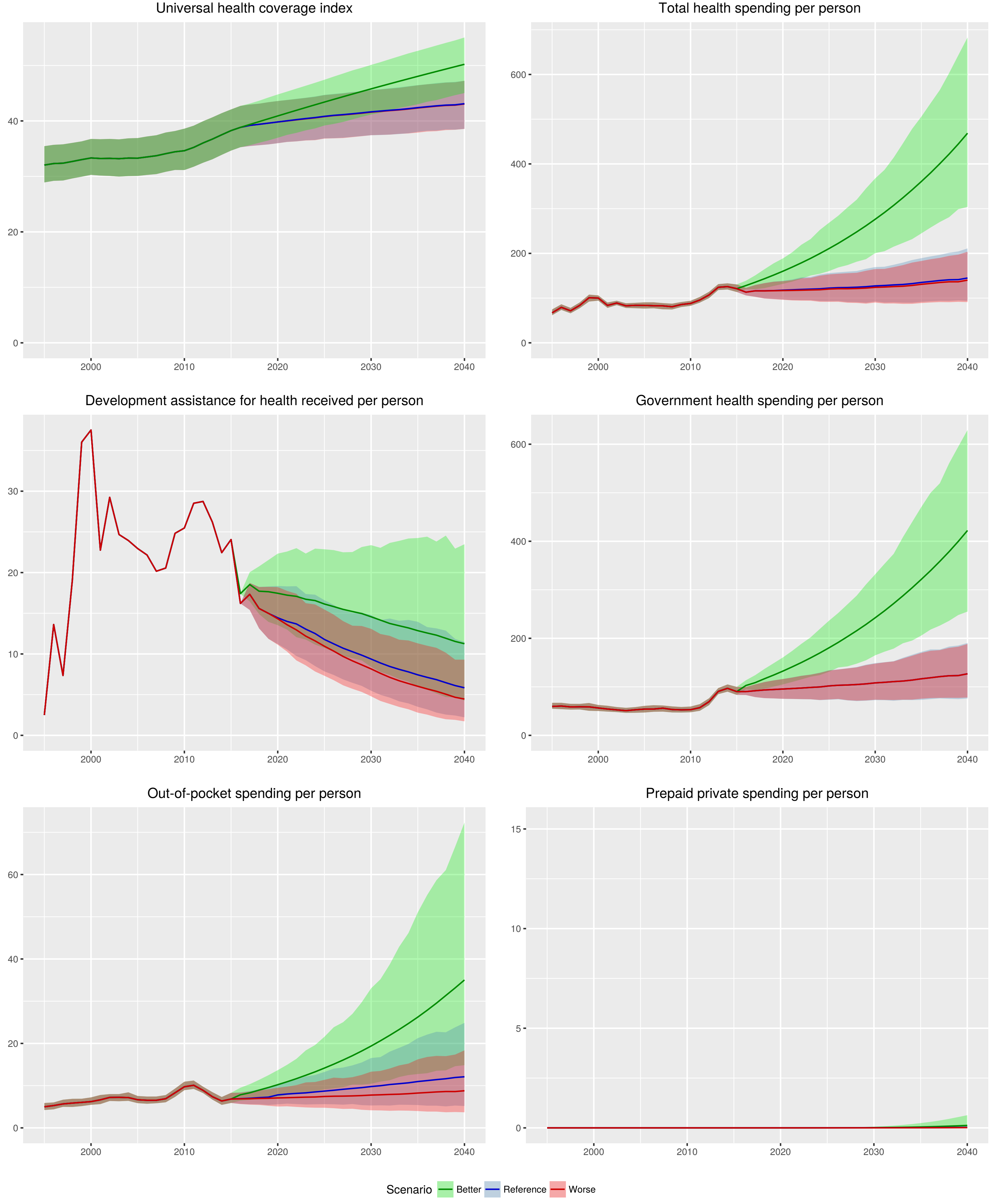

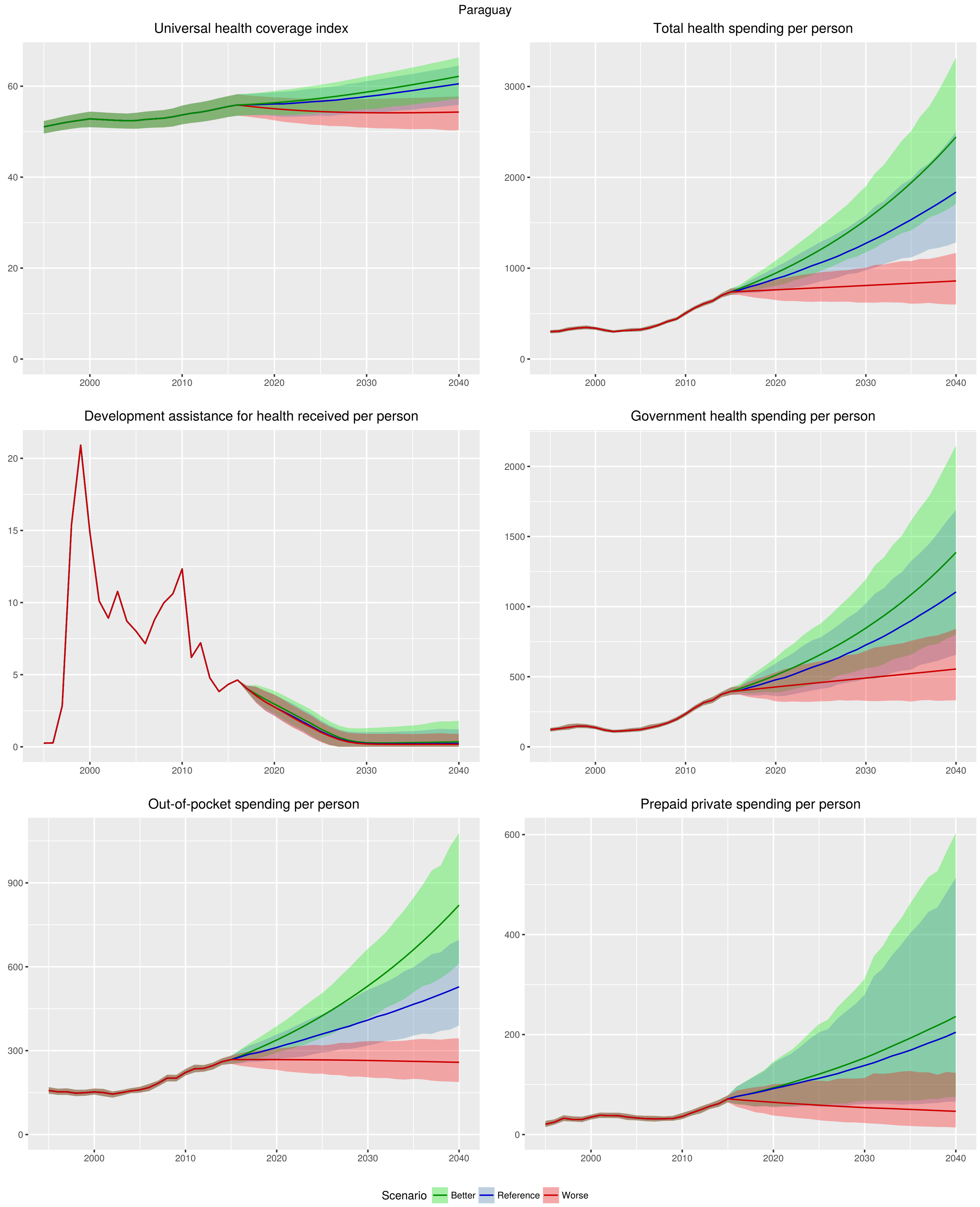

Paraguay
Universal health coverage index
Total health spending per person
Development assistance for health received per person
Government health spending per person
Out-of-pocket spending per person
Prepaid private spending per person
Scenario
Better
Reference
Worse

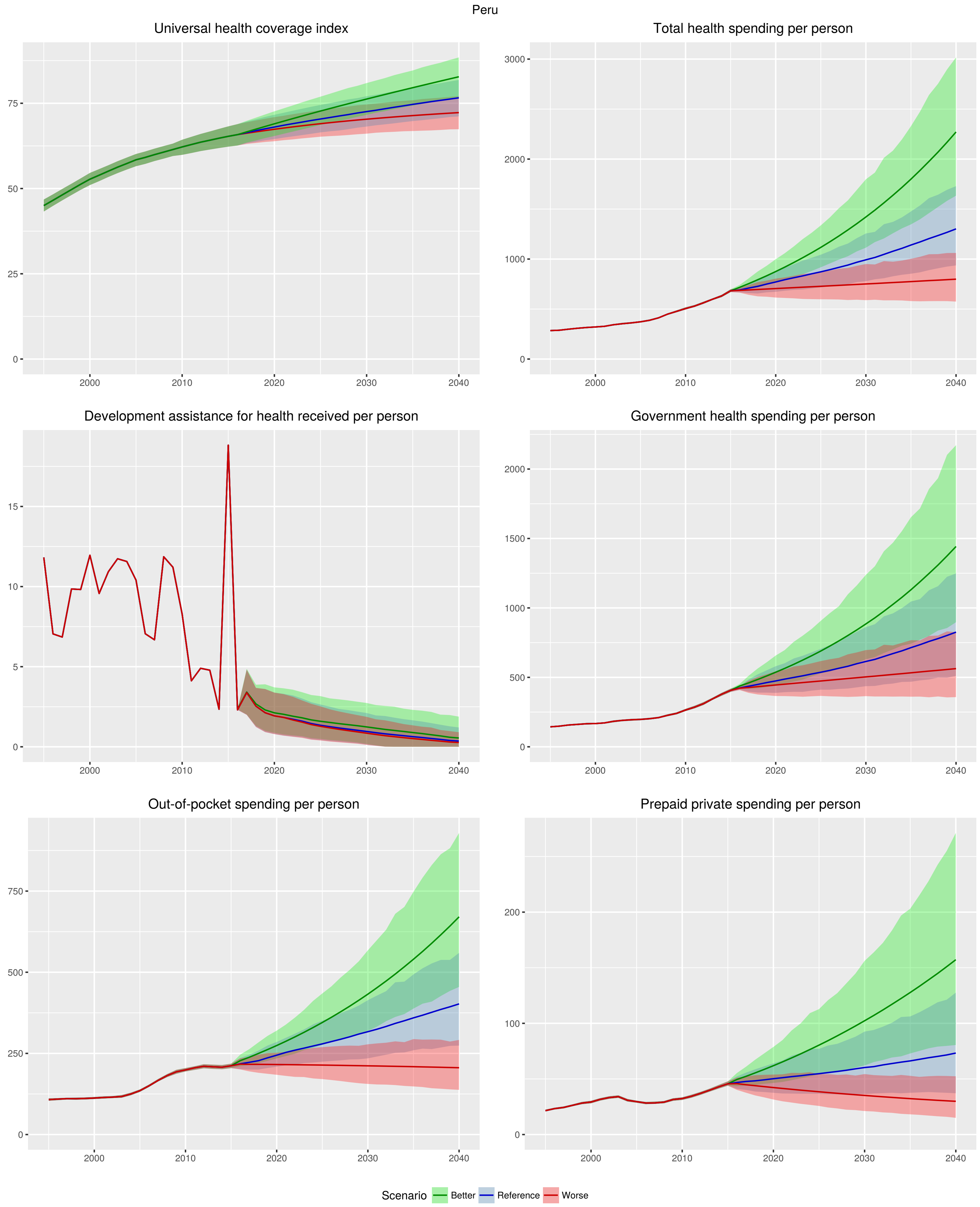

Peru
Universal health coverage index
Total health spending per person
Development assistance for health received per person
Government health spending per person
Out-of-pocket spending per person
Prepaid private spending per person
0
25
50
75
0
1000
2000
3000
0
5
10
15
0
500
1000
1500
2000
0
250
500
750
0
100
200
2000
2010
2020
2030
2040
Scenario
Better
Reference
Worse

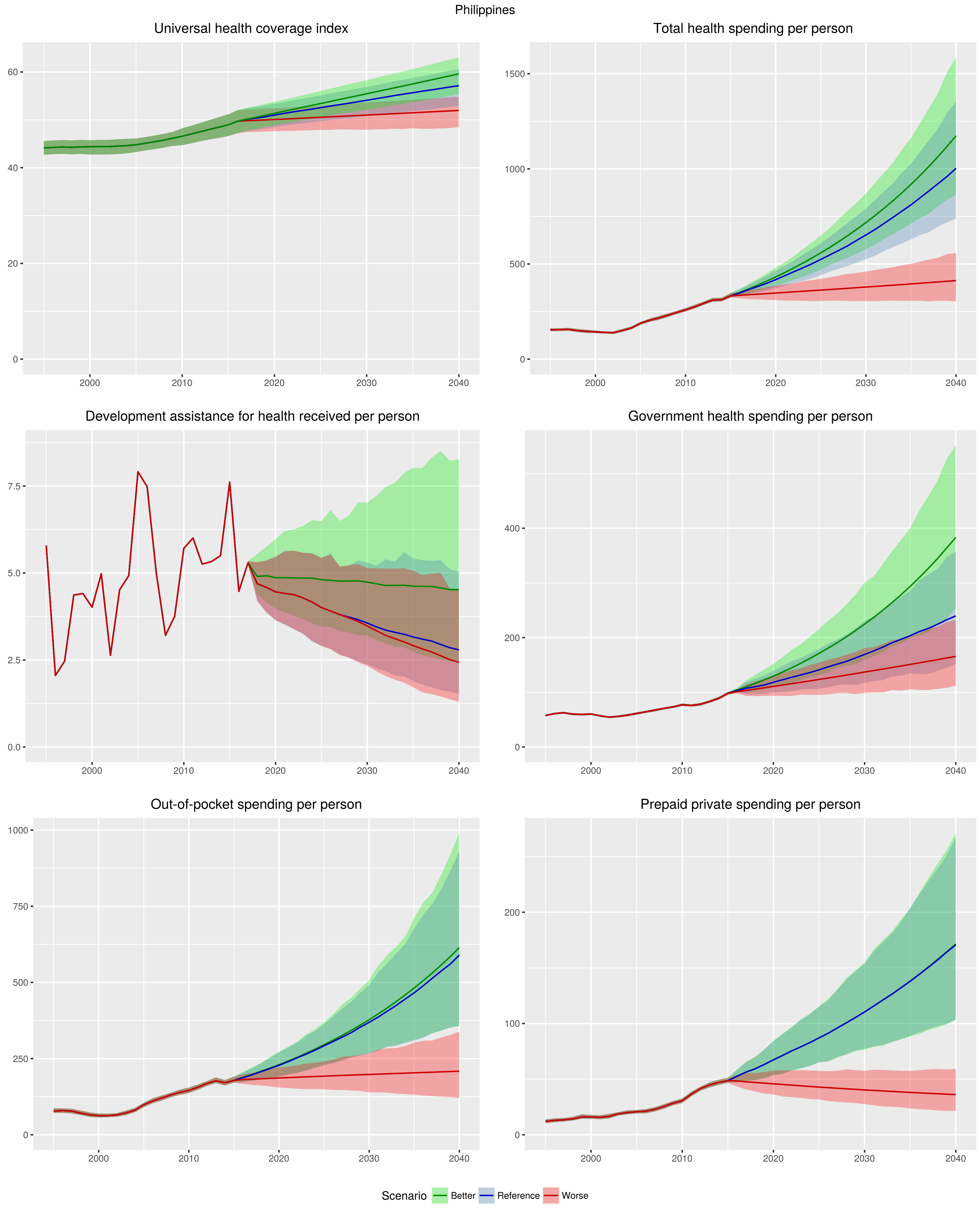

Philippines
Universal health coverage index
Total health spending per person
Development assistance for health received per person
Government health spending per person
Out-of-pocket spending per person
Prepaid private spending per person
Scenario
Better
Reference
Worse

Poland

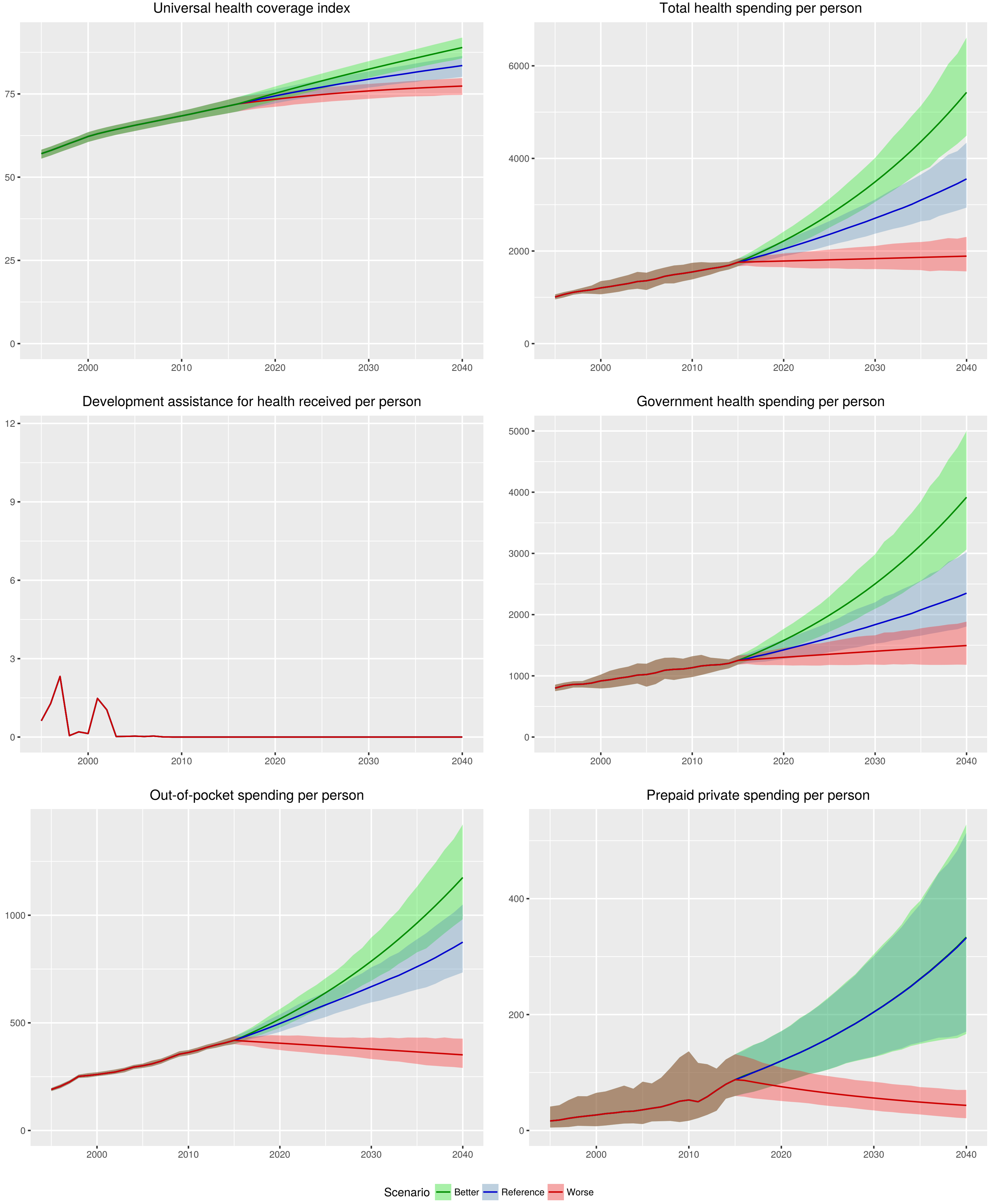

Universal health coverage index
0
25
50
75
2000
2010
2020
2030
2040
Total health spending per person
0
2000
4000
6000
Development assistance for health received per person
0
3
6
9
12
Government health spending per person
1000
2000
3000
4000
5000
Out-of-pocket spending per person
500
1000
Prepaid private spending per person
200
400
Scenario
Better
Reference
Worse

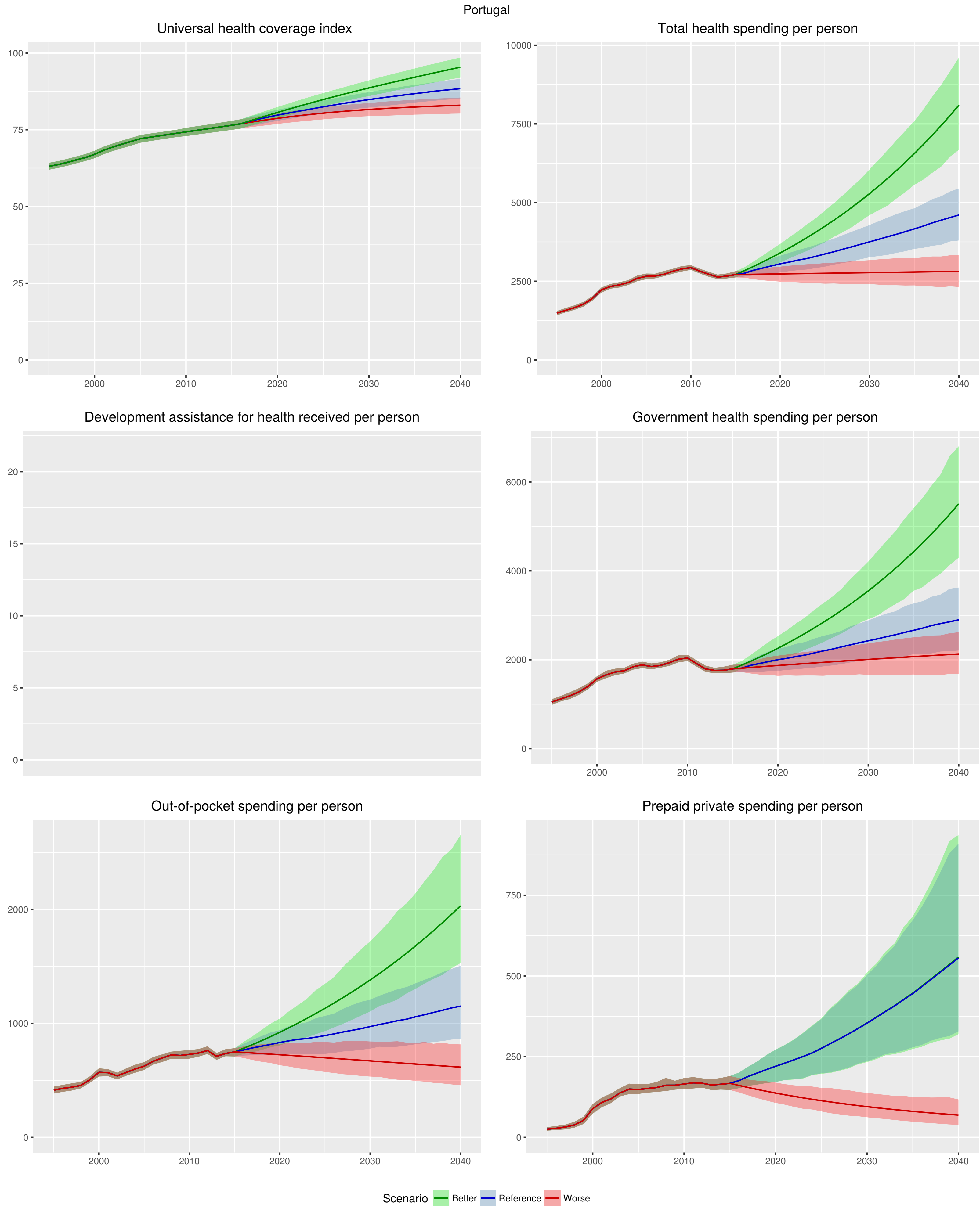

Portugal
Universal health coverage index
Total health spending per person
Development assistance for health received per person
Government health spending per person
Out-of-pocket spending per person
Prepaid private spending per person
Scenario
Better
Reference
Worse

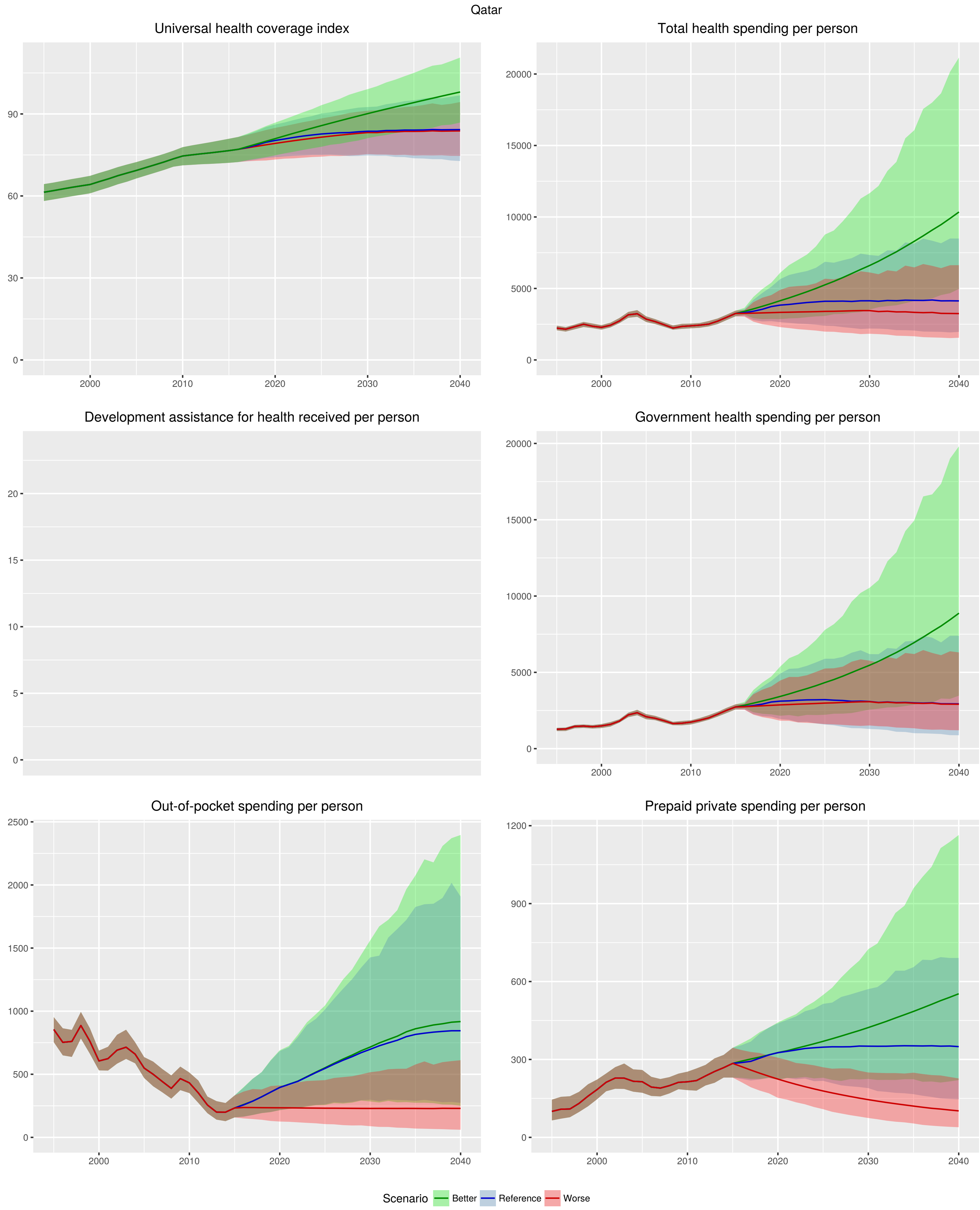

Qatar
Universal health coverage index
Total health spending per person
Development assistance for health received per person
Government health spending per person
Out-of-pocket spending per person
Prepaid private spending per person
0
30
60
90
5
10
15
20
5000
10000
15000
20000
500
1000
1500
2000
2500
300
600
900
1200
2000
2010
2020
2030
2040
Scenario
Better
Reference
Worse

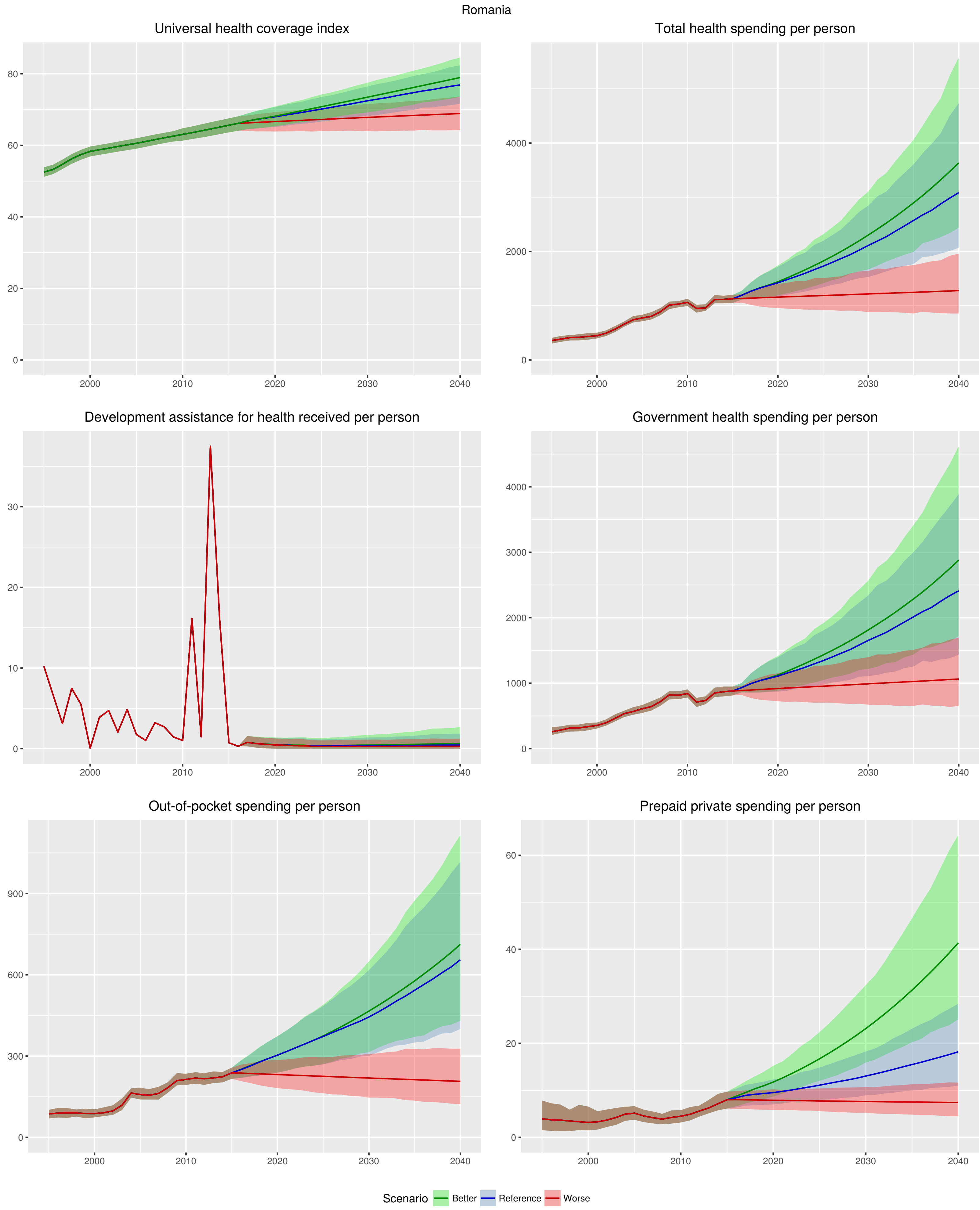
Romania
Universal health coverage index
Total health spending per person
Development assistance for health received per person
Government health spending per person
Out-of-pocket spending per person
Prepaid private spending per person
Scenario
Better
Reference
Worse

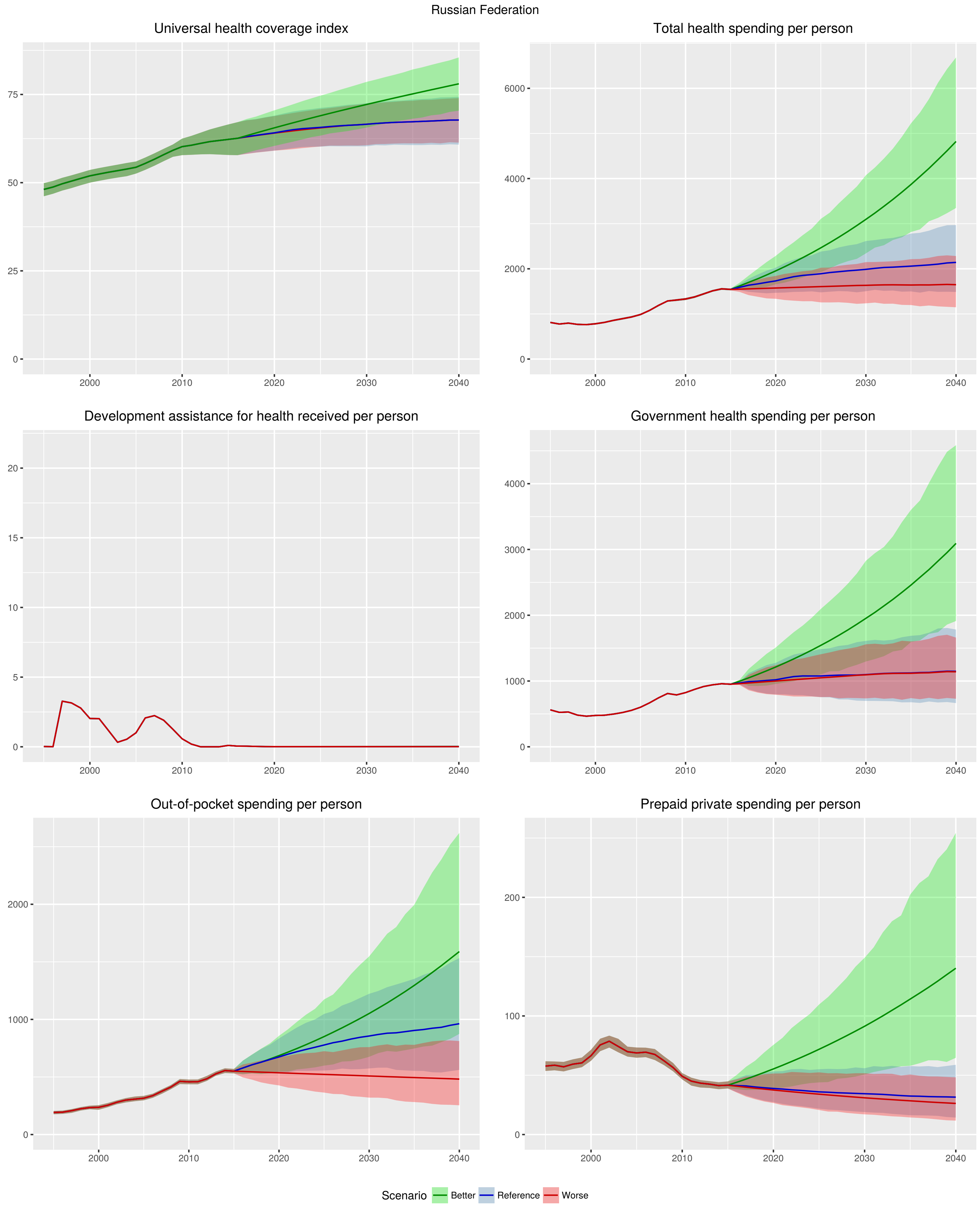
Russian Federation
Universal health coverage index
0
25
50
75
2000
2010
2020
2030
2040
Total health spending per person
0
2000
4000
6000
2000
2010
2020
2030
2040
Development assistance for health received per person
0
5
10
15
20
2000
2010
2020
2030
2040
Government health spending per person
0
1000
2000
3000
4000
2000
2010
2020
2030
2040
Out-of-pocket spending per person
0
1000
2000
2000
2010
2020
2030
2040
Prepaid private spending per person
0
100
200
2000
2010
2020
2030
2040
Scenario
Better
Reference
Worse

Rwanda

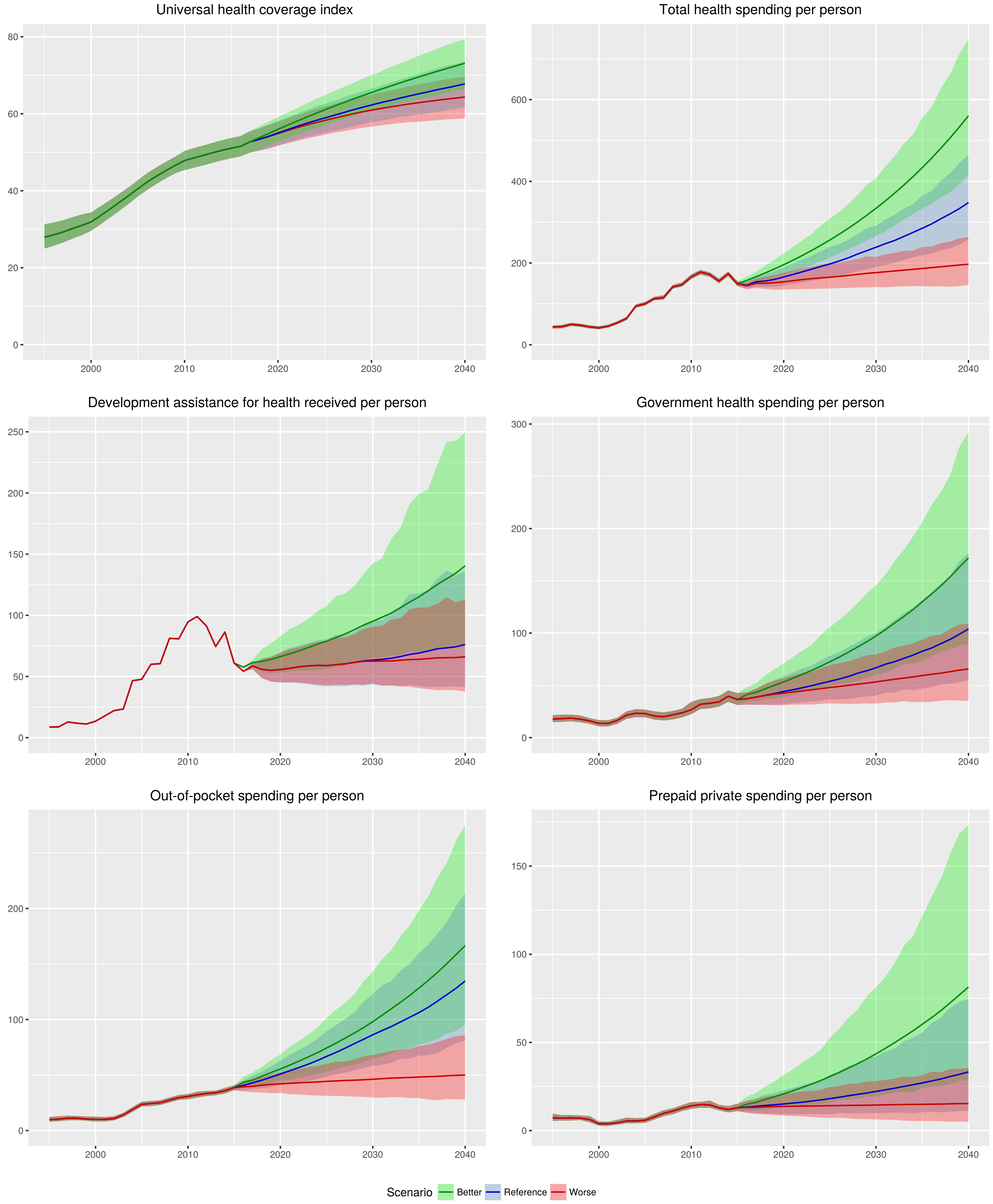
Universal health coverage index
Total health spending per person
Development assistance for health received per person
Government health spending per person
Out-of-pocket spending per person
Prepaid private spending per person
Scenario
Better
Reference
Worse

Saint Lucia

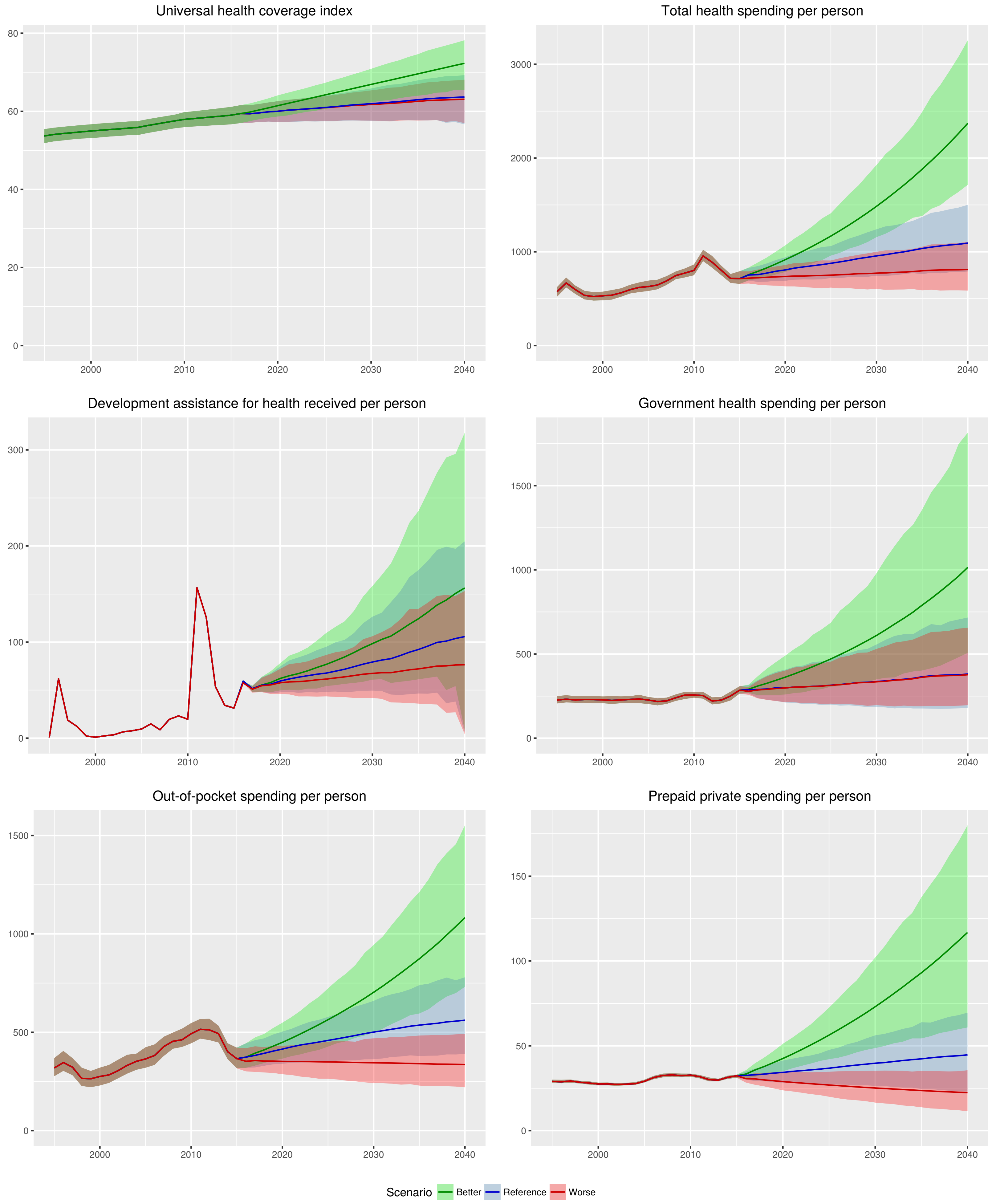

Universal health coverage index
Total health spending per person
Development assistance for health received per person
Government health spending per person
Out-of-pocket spending per person
Prepaid private spending per person
Scenario
Better
Reference
Worse

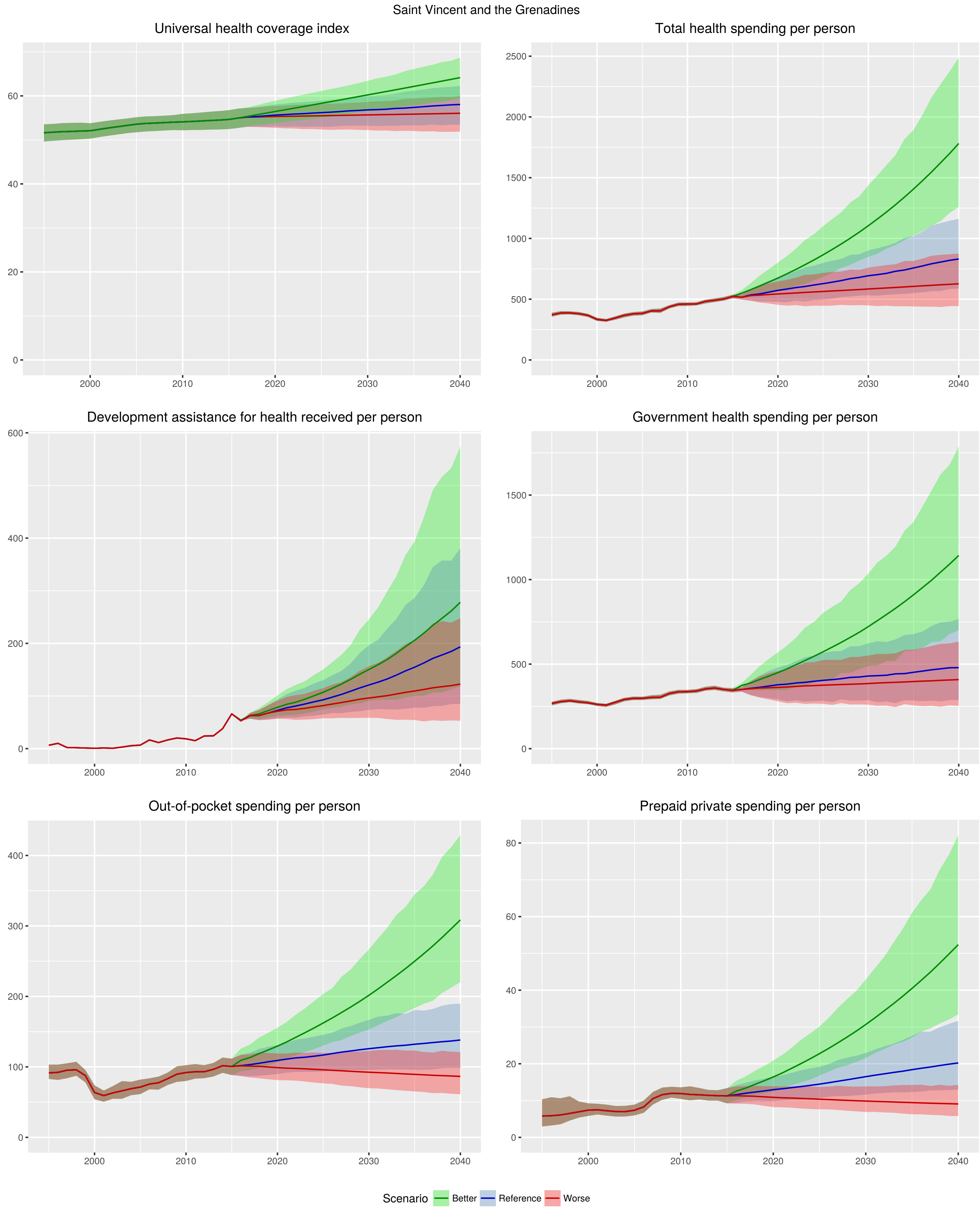

Saint Vincent and the Grenadines
Universal health coverage index
Total health spending per person
Development assistance for health received per person
Government health spending per person
Out-of-pocket spending per person
Prepaid private spending per person
Scenario
Better
Reference
Worse

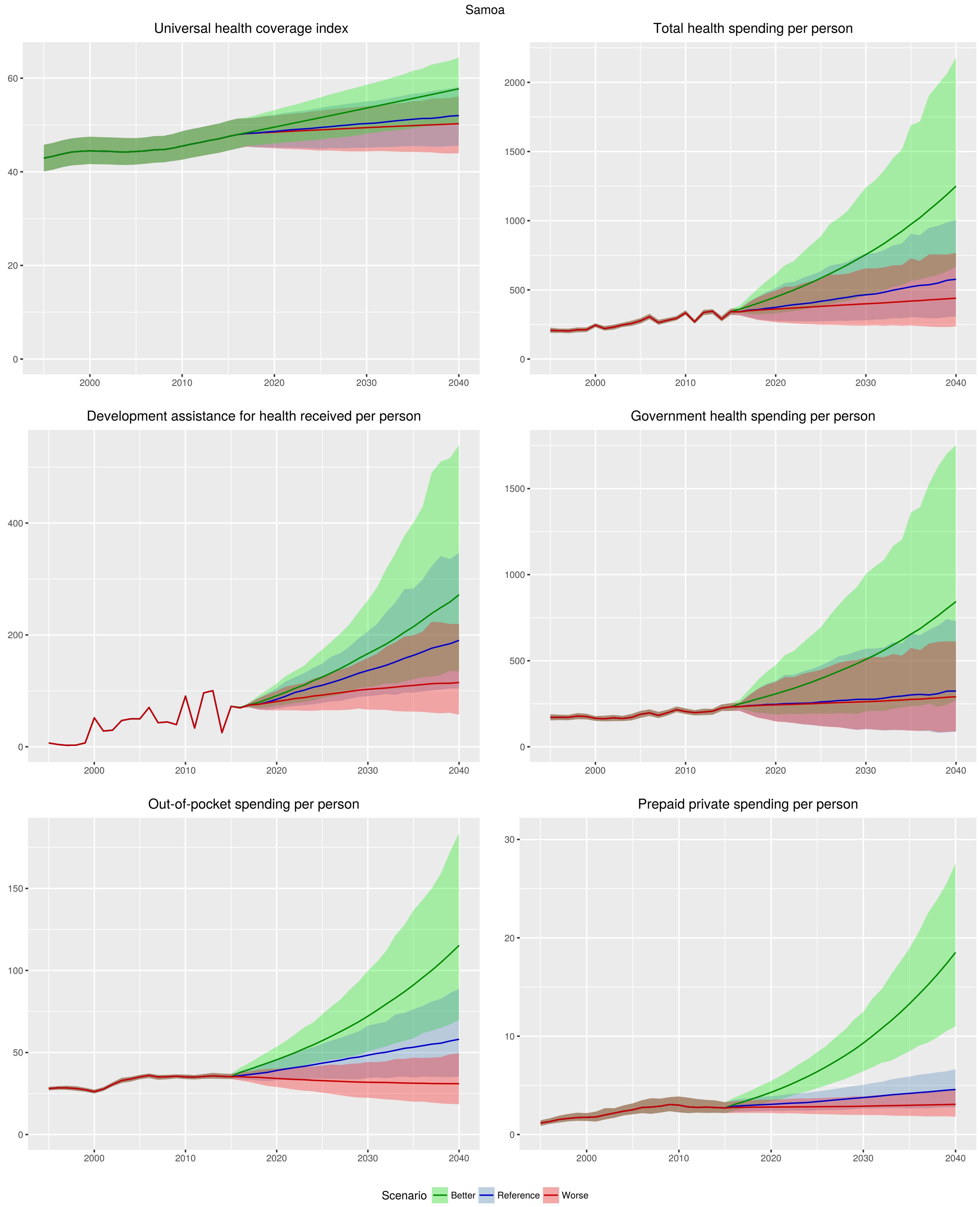
Samoa
Universal health coverage index
Total health spending per person
Development assistance for health received per person
Government health spending per person
Out-of-pocket spending per person
Prepaid private spending per person
0
20
40
60
0
500
1000
1500
2000
200
400
150
100
50
10
30
2000
2010
2020
2030
2040
Scenario
Better
Reference
Worse

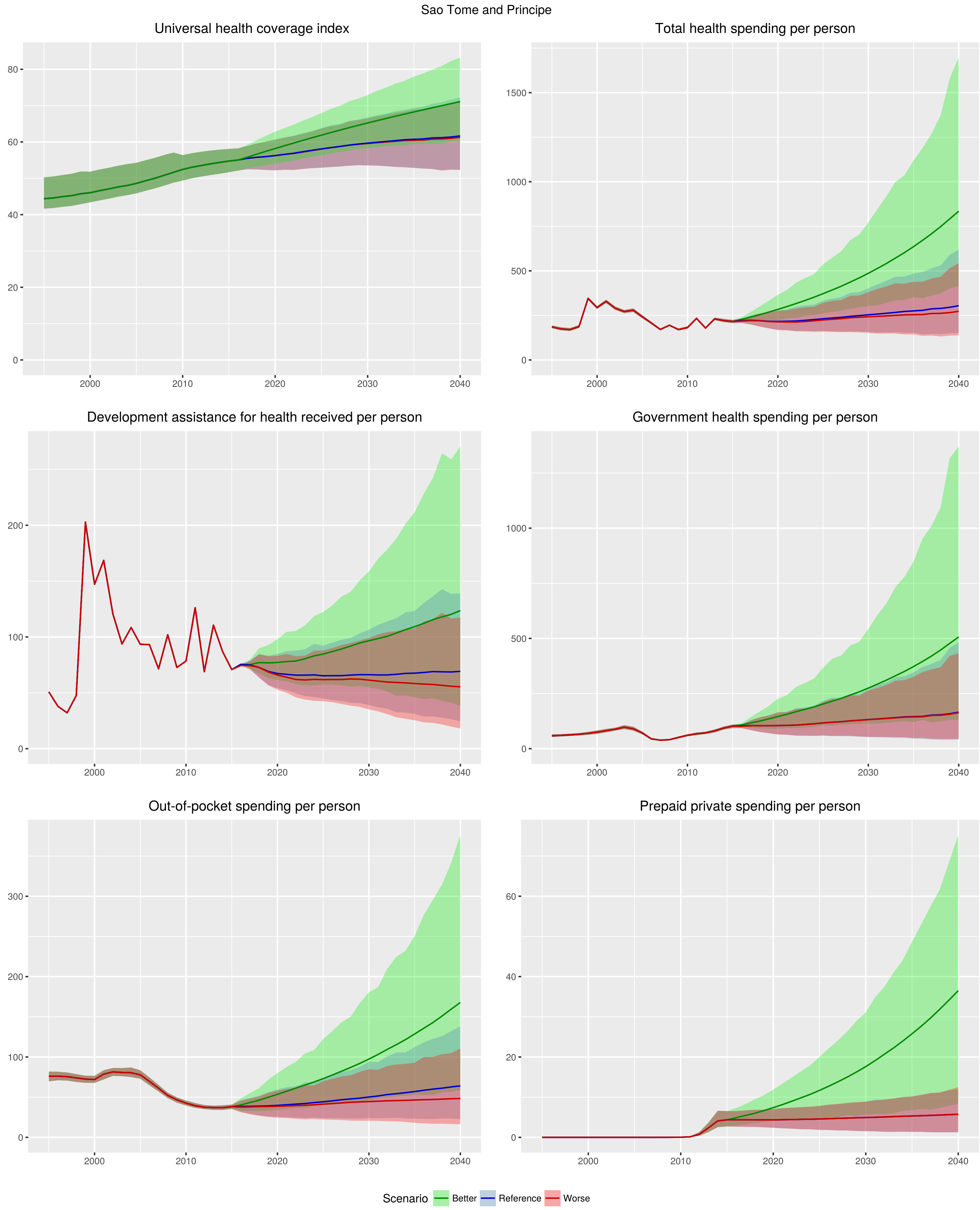
Sao Tome and Principe
Universal health coverage index
Total health spending per person
Development assistance for health received per person
Government health spending per person
Out-of-pocket spending per person
Prepaid private spending per person
Scenario
Better
Reference
Worse

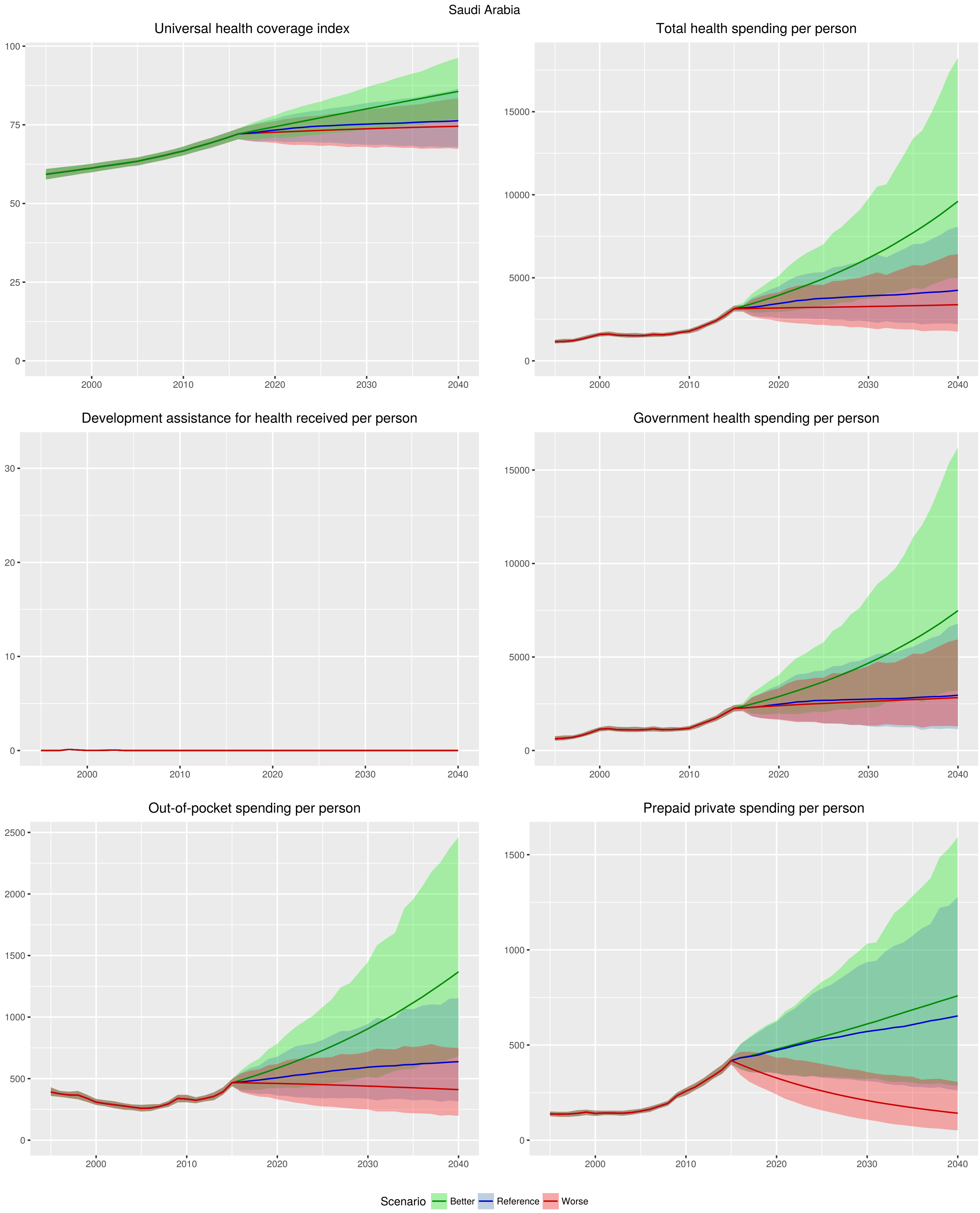
Saudi Arabia
Universal health coverage index
Total health spending per person
Development assistance for health received per person
Government health spending per person
Out-of-pocket spending per person
Prepaid private spending per person
Scenario
Better
Reference
Worse

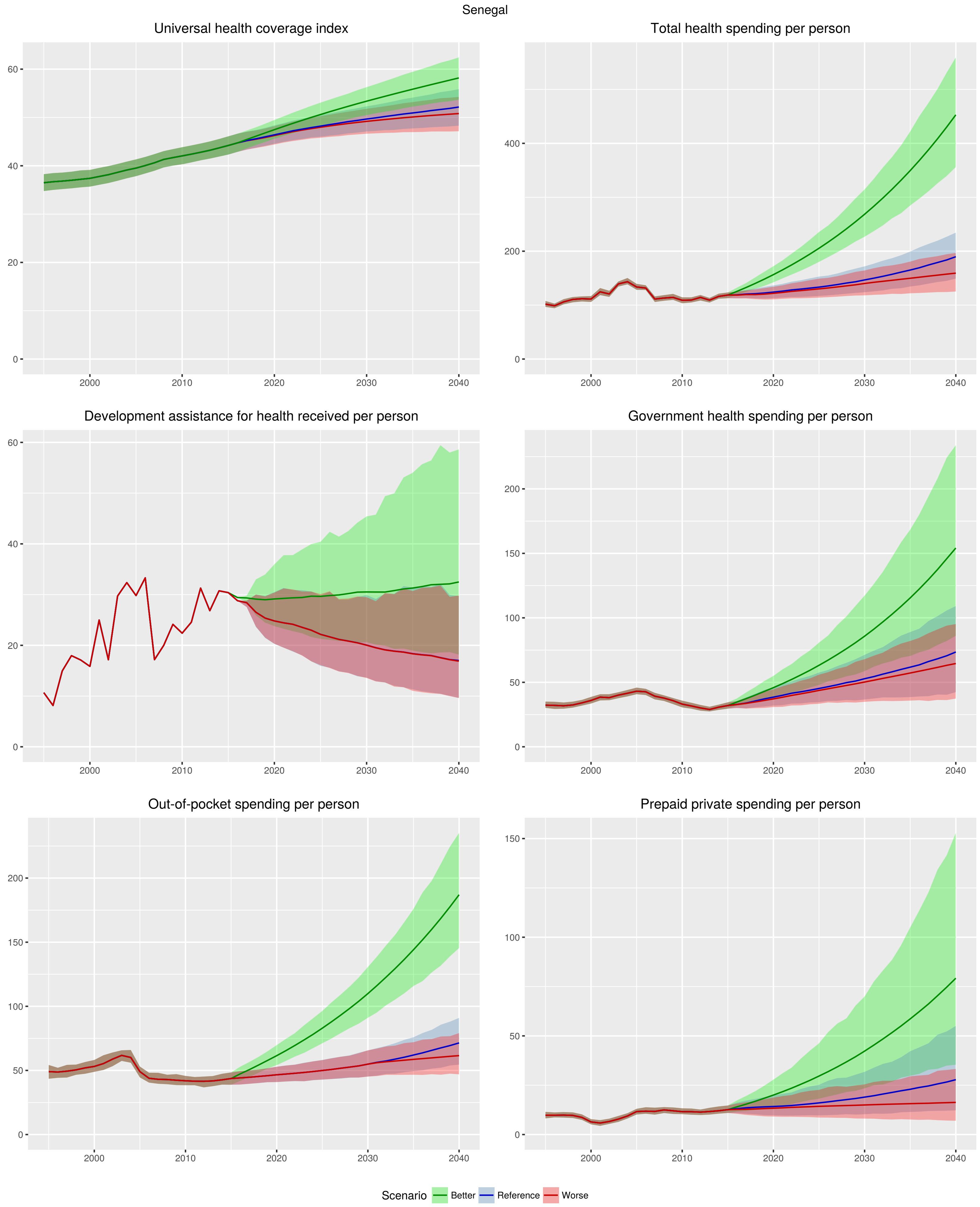
Senegal
Universal health coverage index
Total health spending per person
Development assistance for health received per person
Government health spending per person
Out-of-pocket spending per person
Prepaid private spending per person
0
20
40
60
200
400
50
100
150
2000
2010
2020
2030
2040
Scenario
Better
Reference
Worse

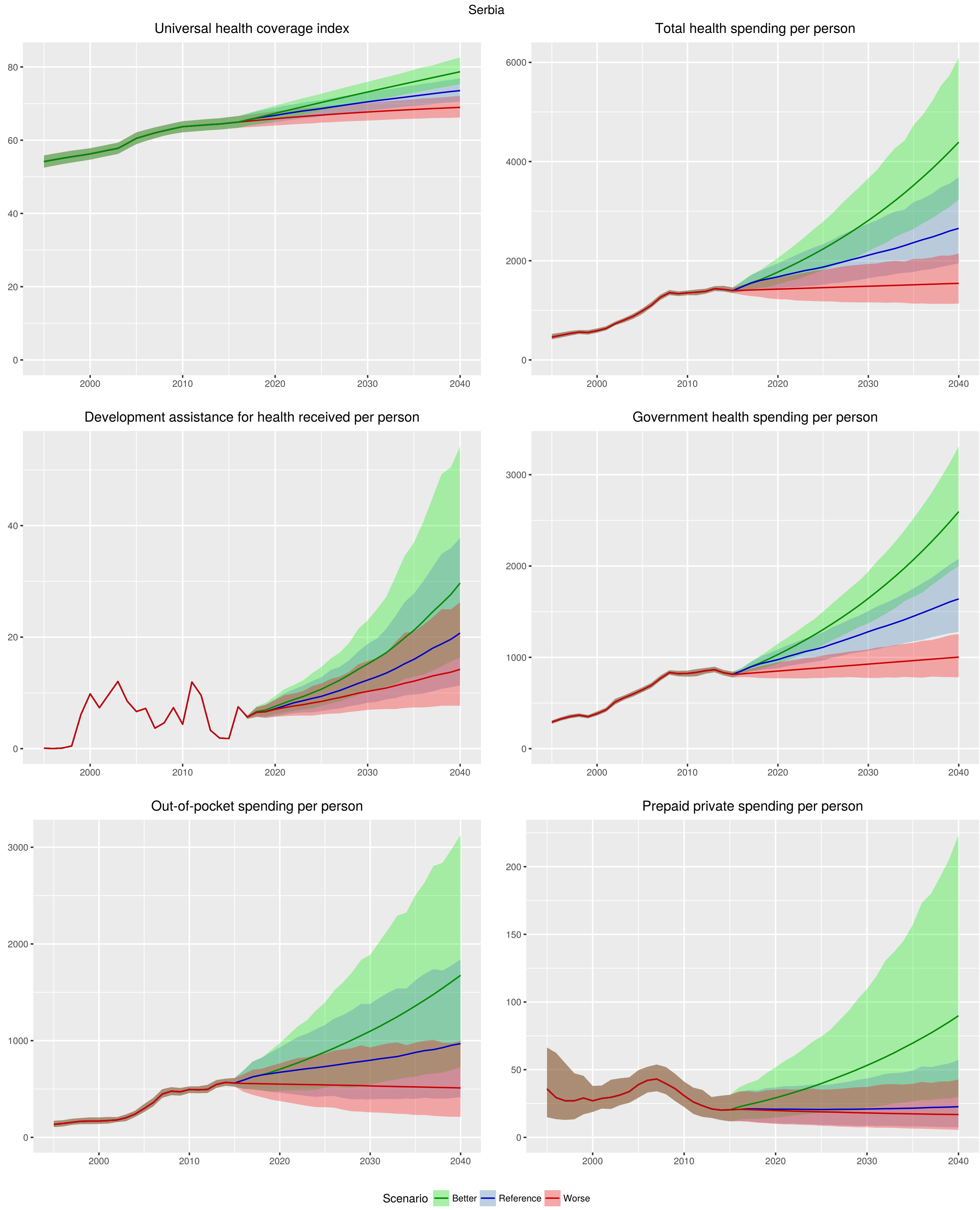

Serbia
Universal health coverage index
Total health spending per person
Development assistance for health received per person
Government health spending per person
Out-of-pocket spending per person
Prepaid private spending per person
Scenario
Better
Reference
Worse

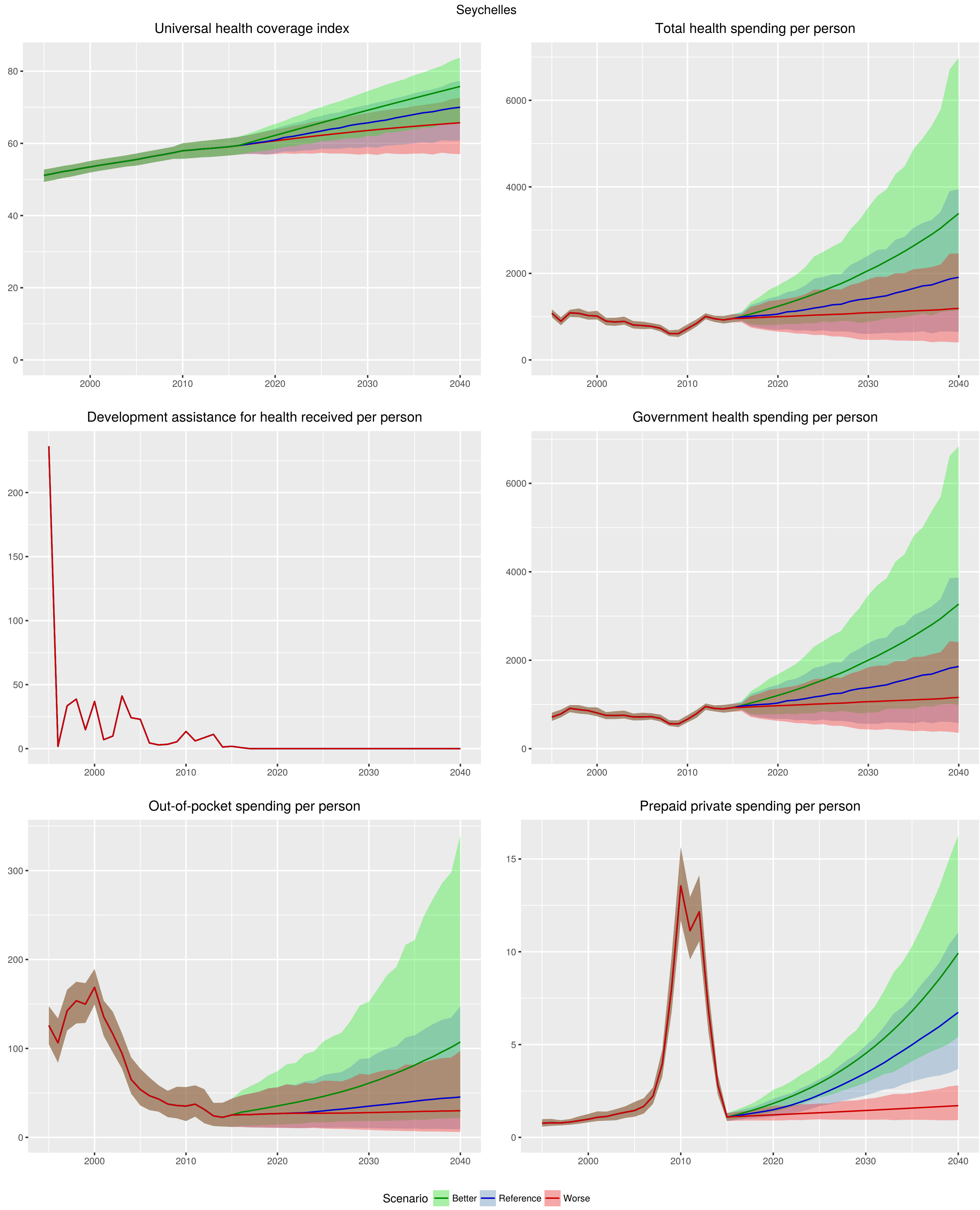
Seychelles
Universal health coverage index
Total health spending per person
Development assistance for health received per person
Government health spending per person
Out-of-pocket spending per person
Prepaid private spending per person
0
20
40
60
80
2000
2010
2020
2030
2040
2000
4000
6000
50
100
150
200
300
5
10
15
Scenario
Better
Reference
Worse

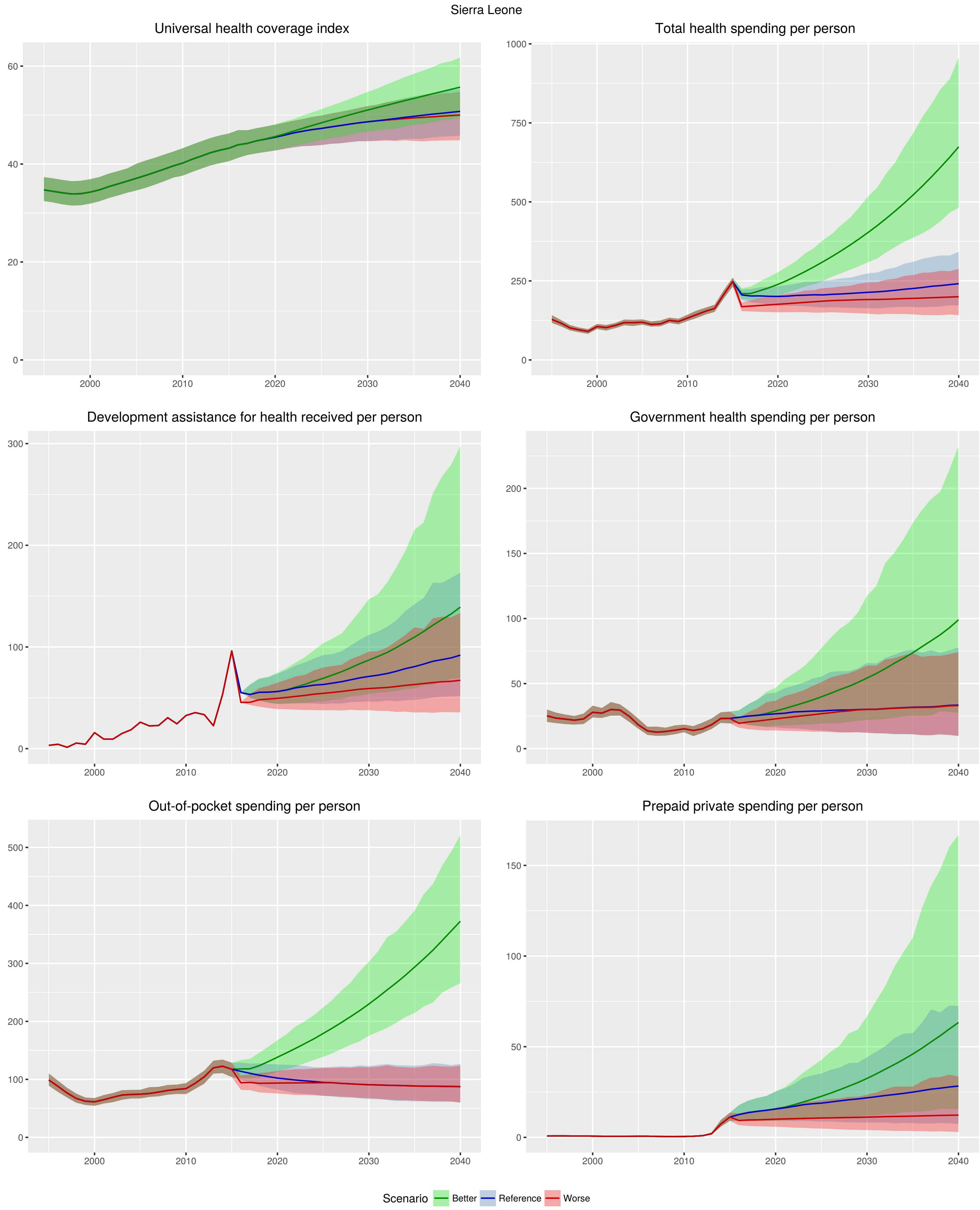

Sierra Leone
Universal health coverage index
Total health spending per person
Development assistance for health received per person
Government health spending per person
Out-of-pocket spending per person
Prepaid private spending per person
Scenario
Better
Reference
Worse

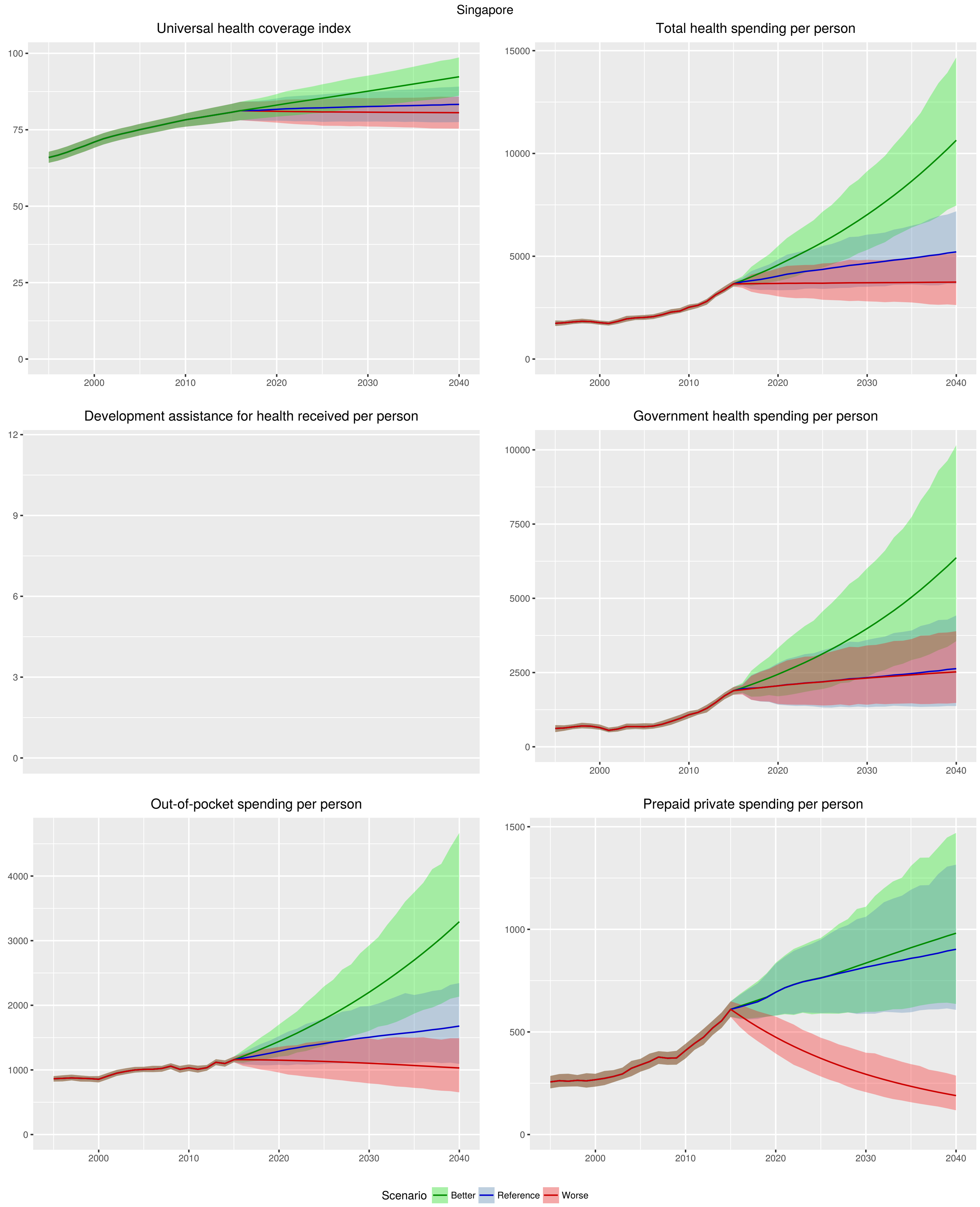
Singapore
Universal health coverage index
Total health spending per person
Development assistance for health received per person
Government health spending per person
Out-of-pocket spending per person
Prepaid private spending per person
Scenario
Better
Reference
Worse

Slovakia

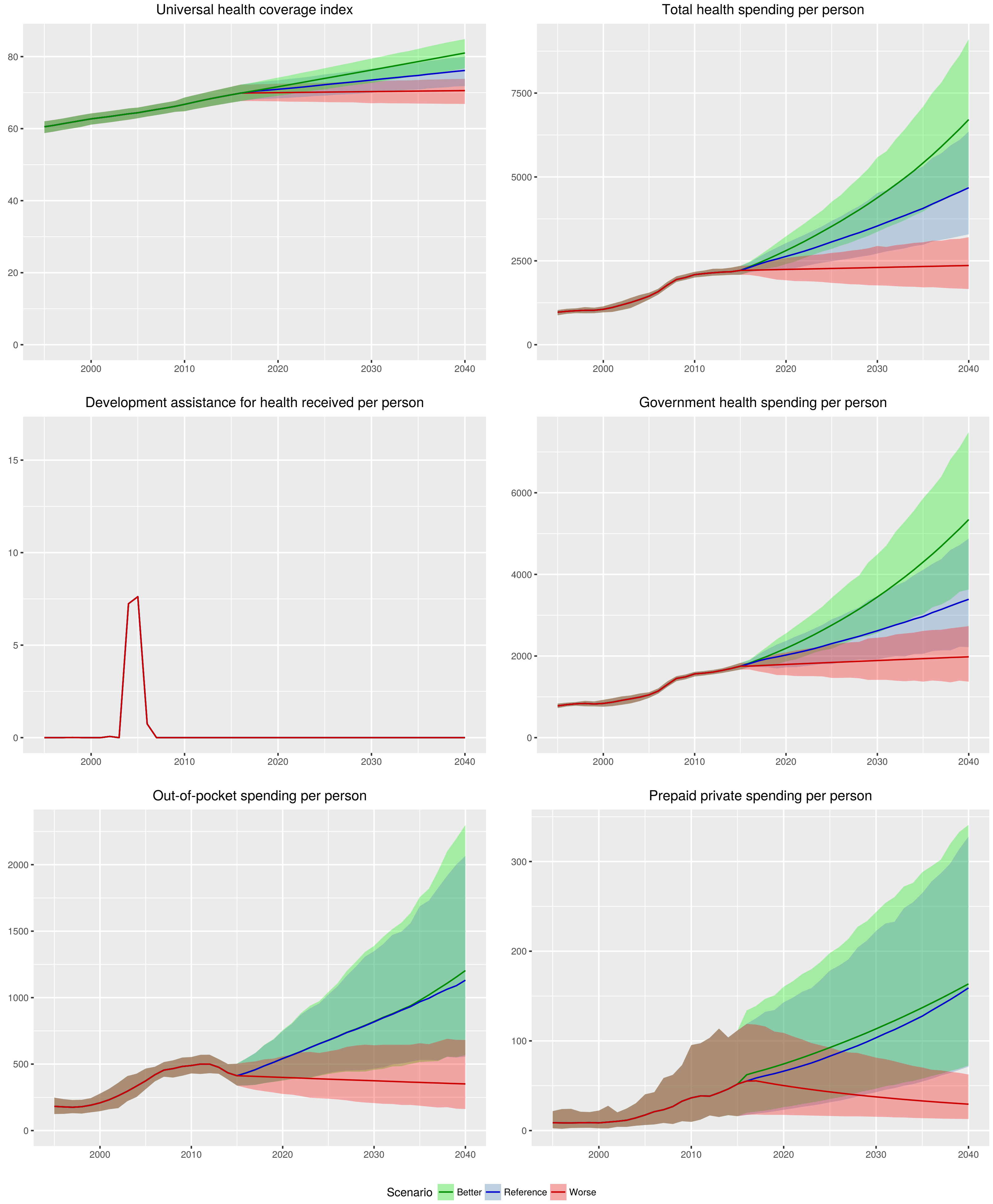

Universal health coverage index
0
20
40
60
80
2000
2010
2020
2030
2040
Total health spending per person
0
2500
5000
7500
Development assistance for health received per person
0
5
10
15
Government health spending per person
0
2000
4000
6000
Out-of-pocket spending per person
0
500
1000
1500
2000
Prepaid private spending per person
0
100
200
300
Scenario
Better
Reference
Worse

Slovenia

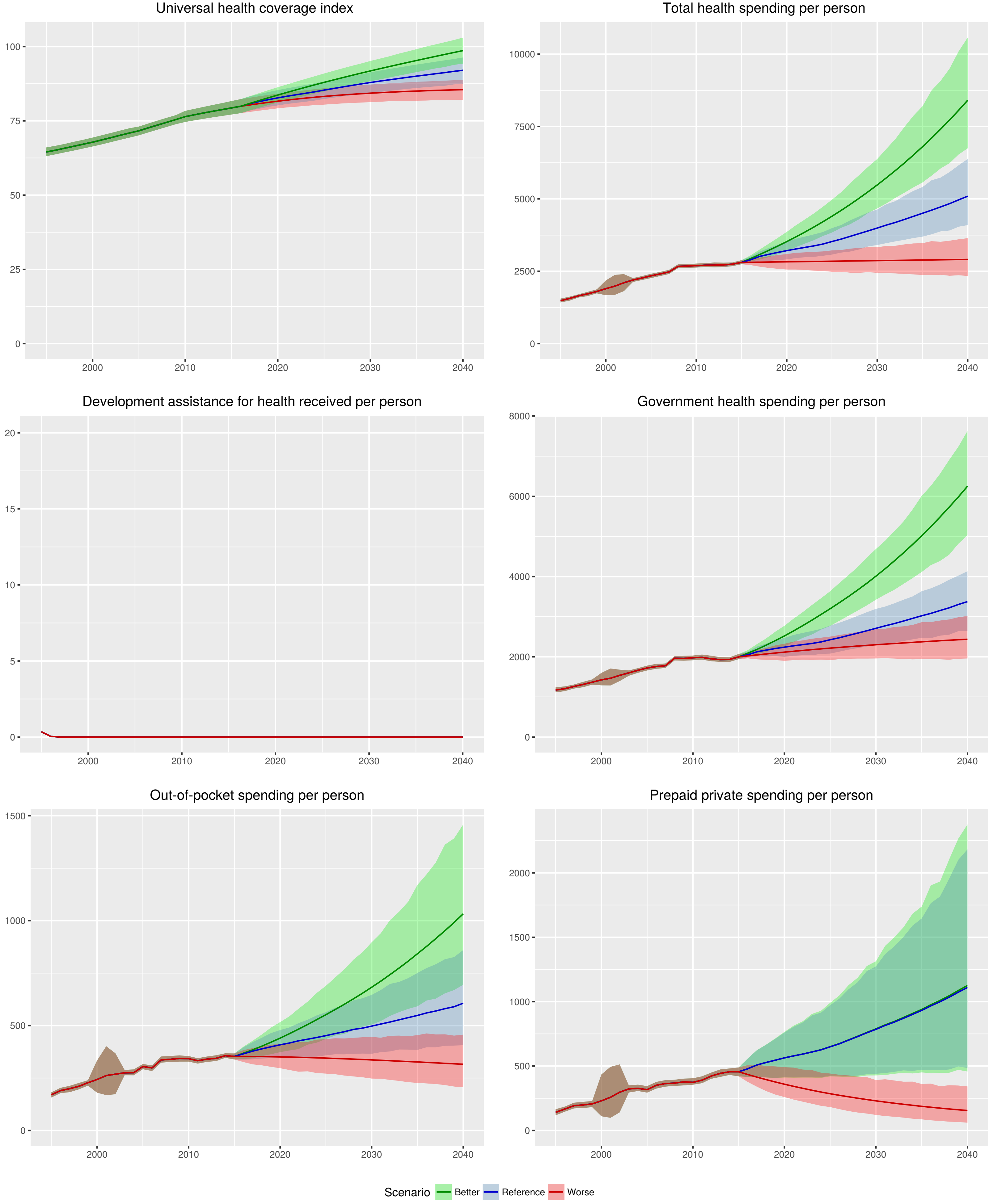
Universal health coverage index
Total health spending per person
Development assistance for health received per person
Government health spending per person
Out-of-pocket spending per person
Prepaid private spending per person
0
25
50
75
100
2500
5000
7500
10000
5
10
15
20
2000
4000
6000
8000
500
1000
1500
2000
2010
2020
2030
2040
Scenario
Better
Reference
Worse

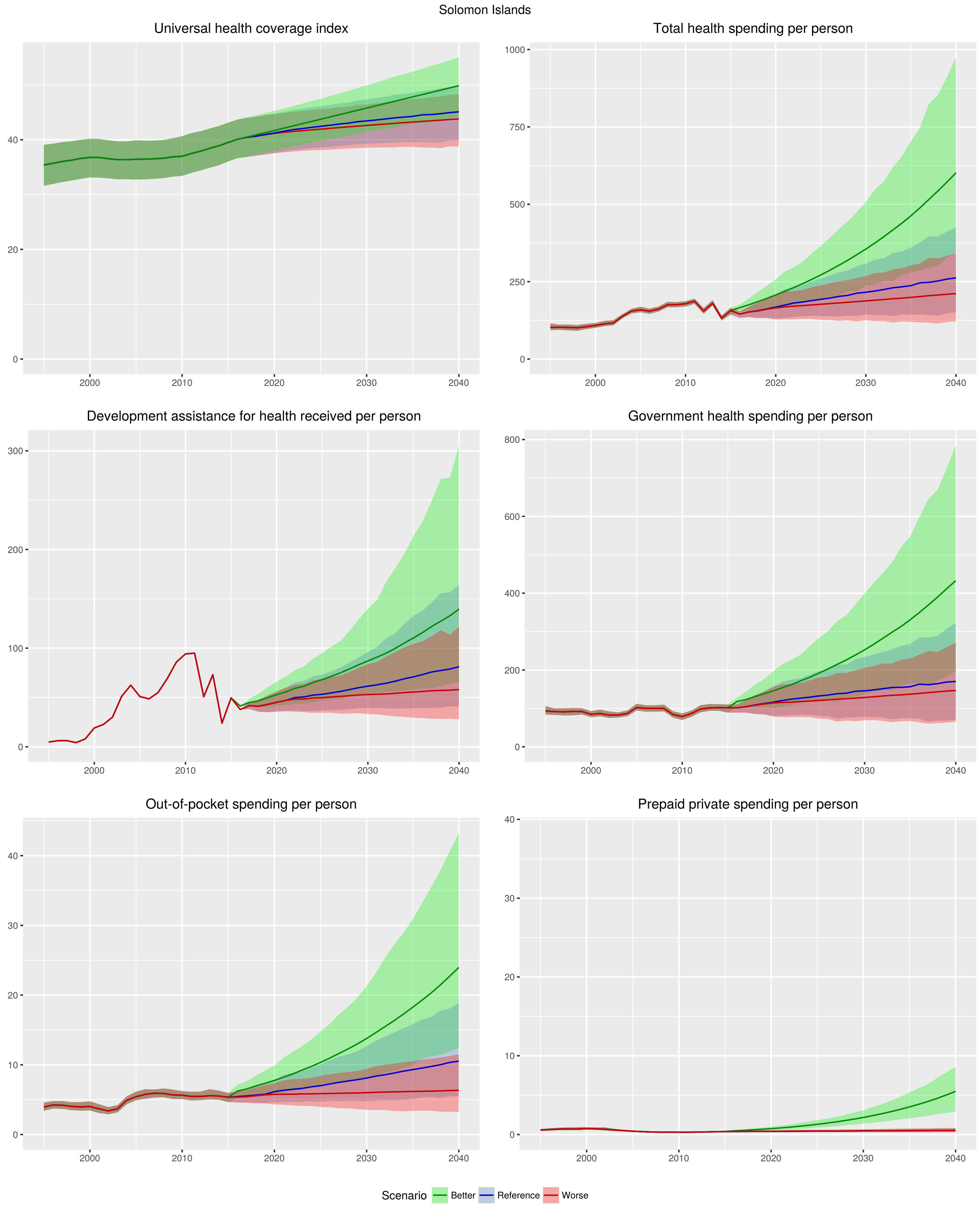
Solomon Islands
Universal health coverage index
Total health spending per person
Development assistance for health received per person
Government health spending per person
Out-of-pocket spending per person
Prepaid private spending per person
0
20
40
250
500
750
1000
100
200
300
200
400
600
800
10
30
2000
2010
2020
2030
2040
Scenario
Better
Reference
Worse

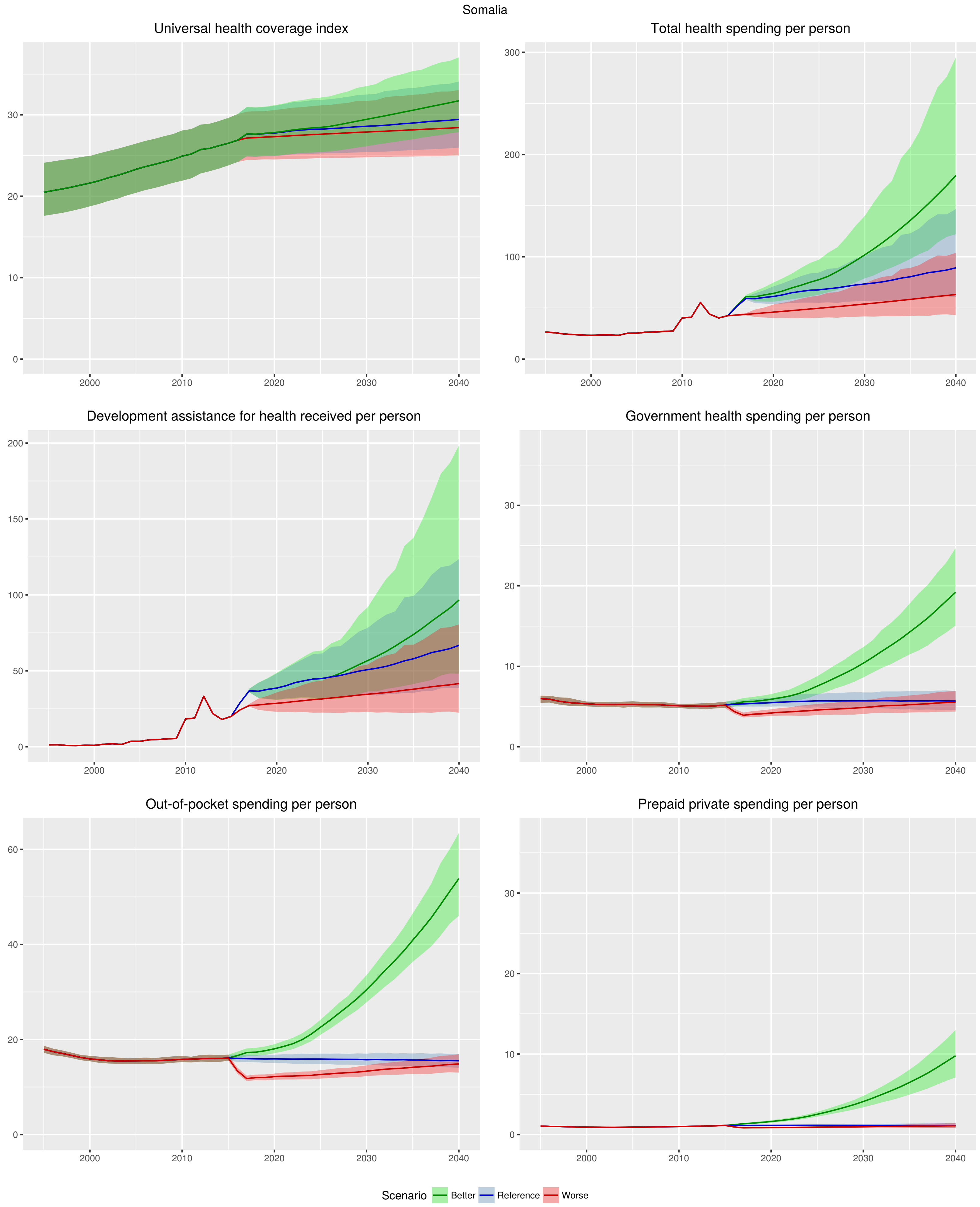

Somalia
Universal health coverage index
Total health spending per person
Development assistance for health received per person
Government health spending per person
Out-of-pocket spending per person
Prepaid private spending per person
0
10
20
30
100
200
300
50
150
40
60
2000
2010
2020
2030
2040
Scenario
Better
Reference
Worse

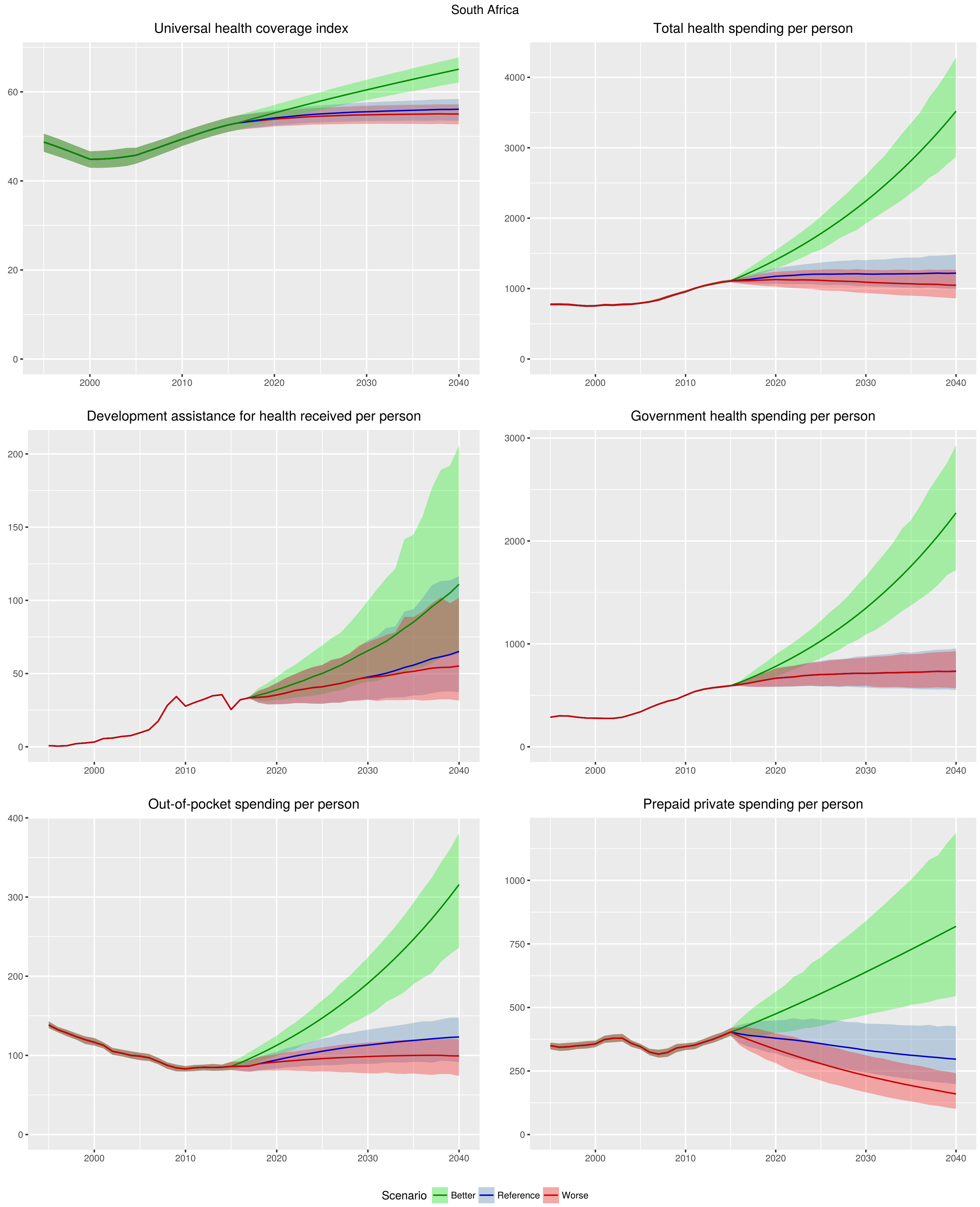
South Africa
Universal health coverage index
Total health spending per person
Development assistance for health received per person
Government health spending per person
Out-of-pocket spending per person
Prepaid private spending per person
Scenario
Better
Reference
Worse

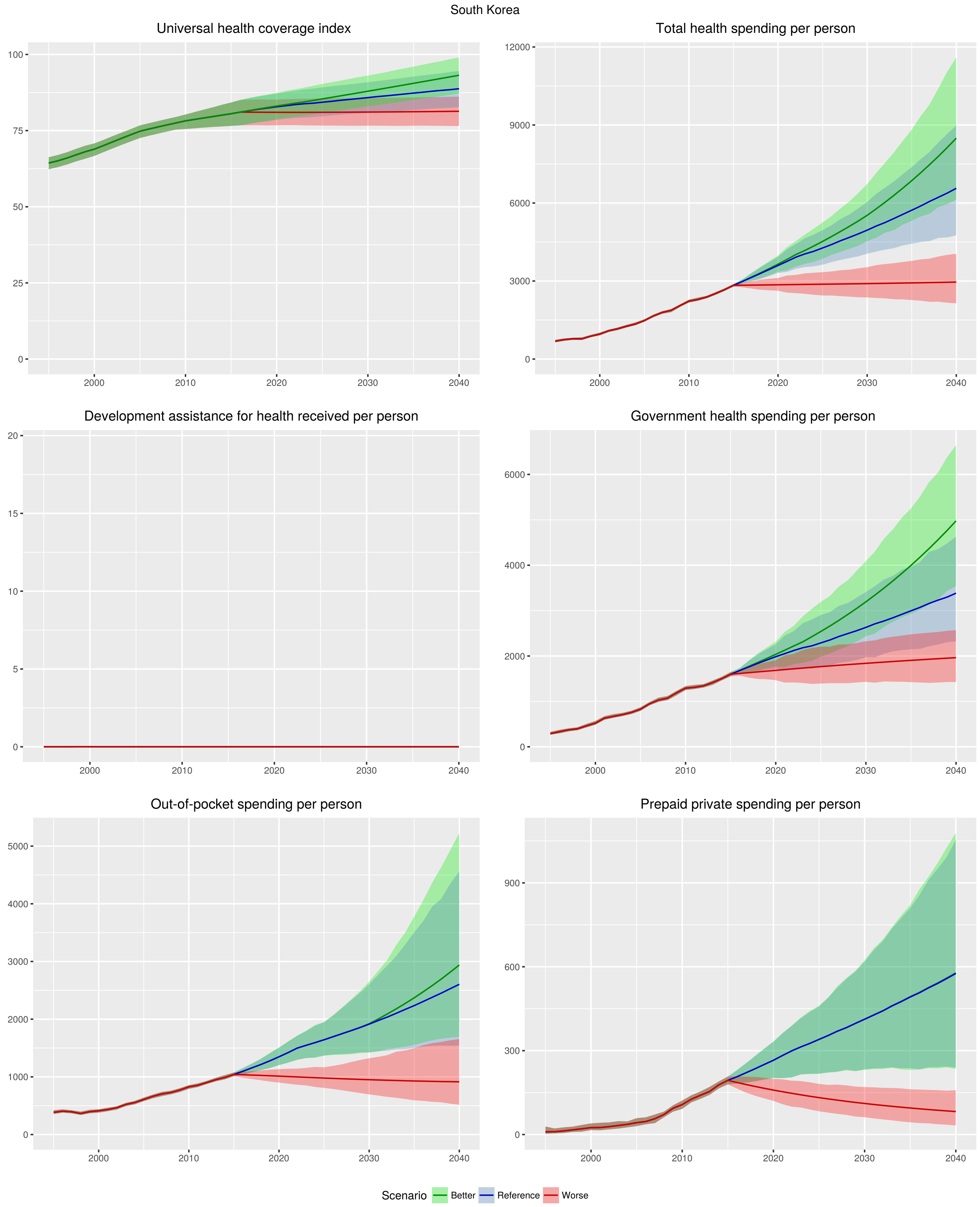
South Korea
Universal health coverage index
Total health spending per person
Development assistance for health received per person
Government health spending per person
Out-of-pocket spending per person
Prepaid private spending per person
Scenario
Better
Reference
Worse

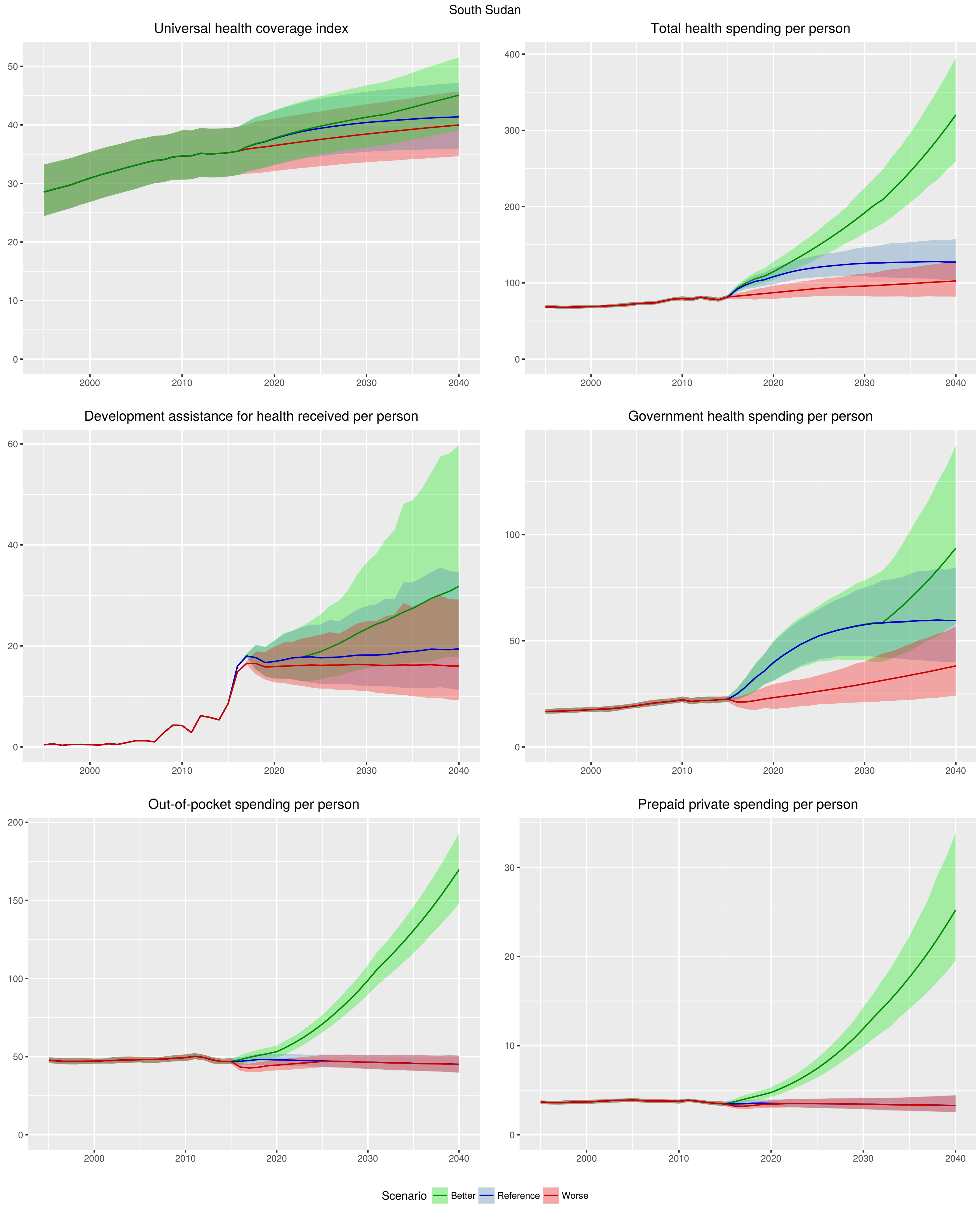
South Sudan
Universal health coverage index
Total health spending per person
Development assistance for health received per person
Government health spending per person
Out-of-pocket spending per person
Prepaid private spending per person
Scenario
Better
Reference
Worse

Spain

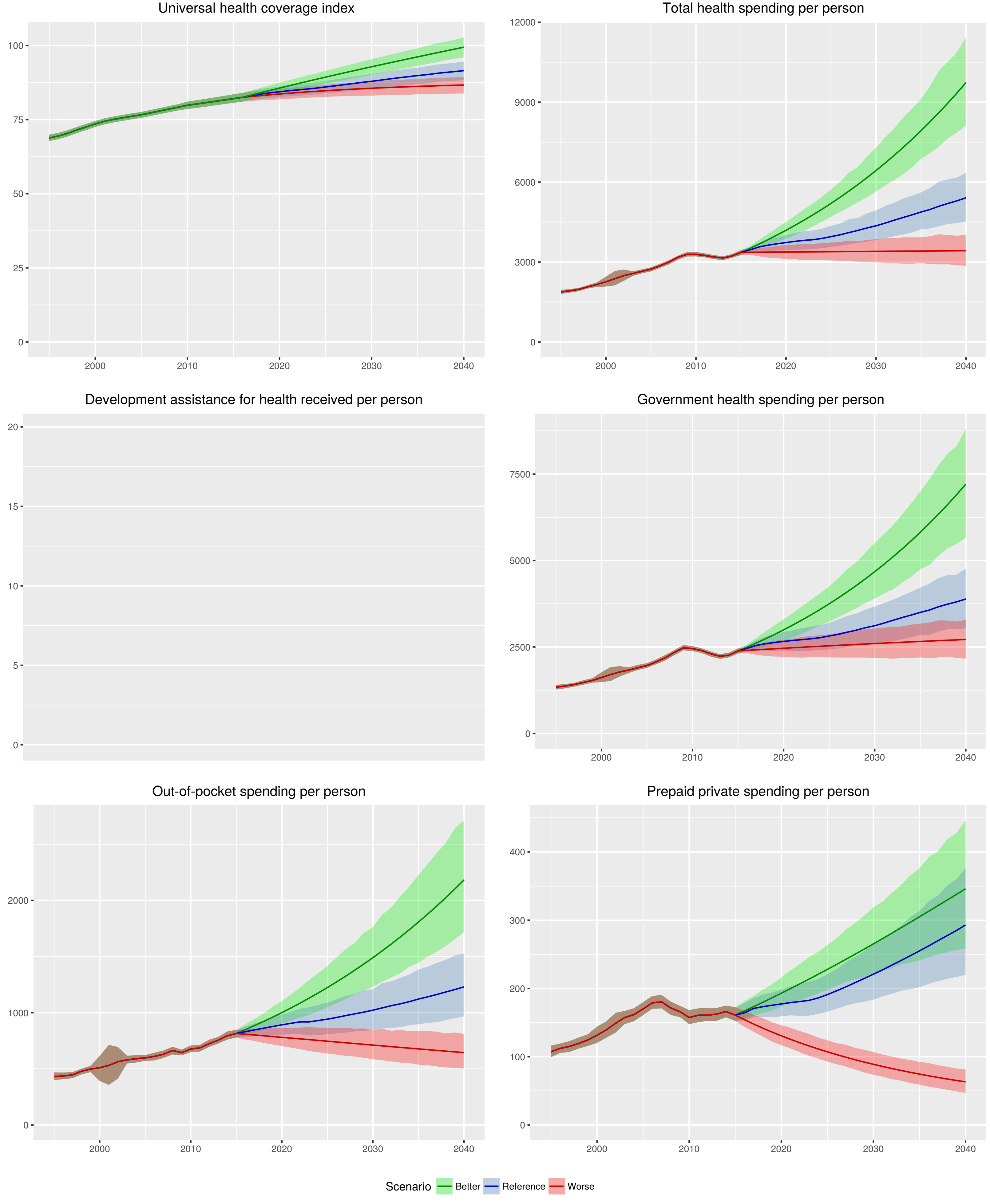

Universal health coverage index
100
75
50
25
0
2000
2010
2020
2030
2040
Total health spending per person
12000
9000
6000
3000
0
Development assistance for health received per person
20
15
10
5
Government health spending per person
7500
5000
2500
Out-of-pocket spending per person
2000
1000
Prepaid private spending per person
400
300
200
100
Scenario
Better
Reference
Worse

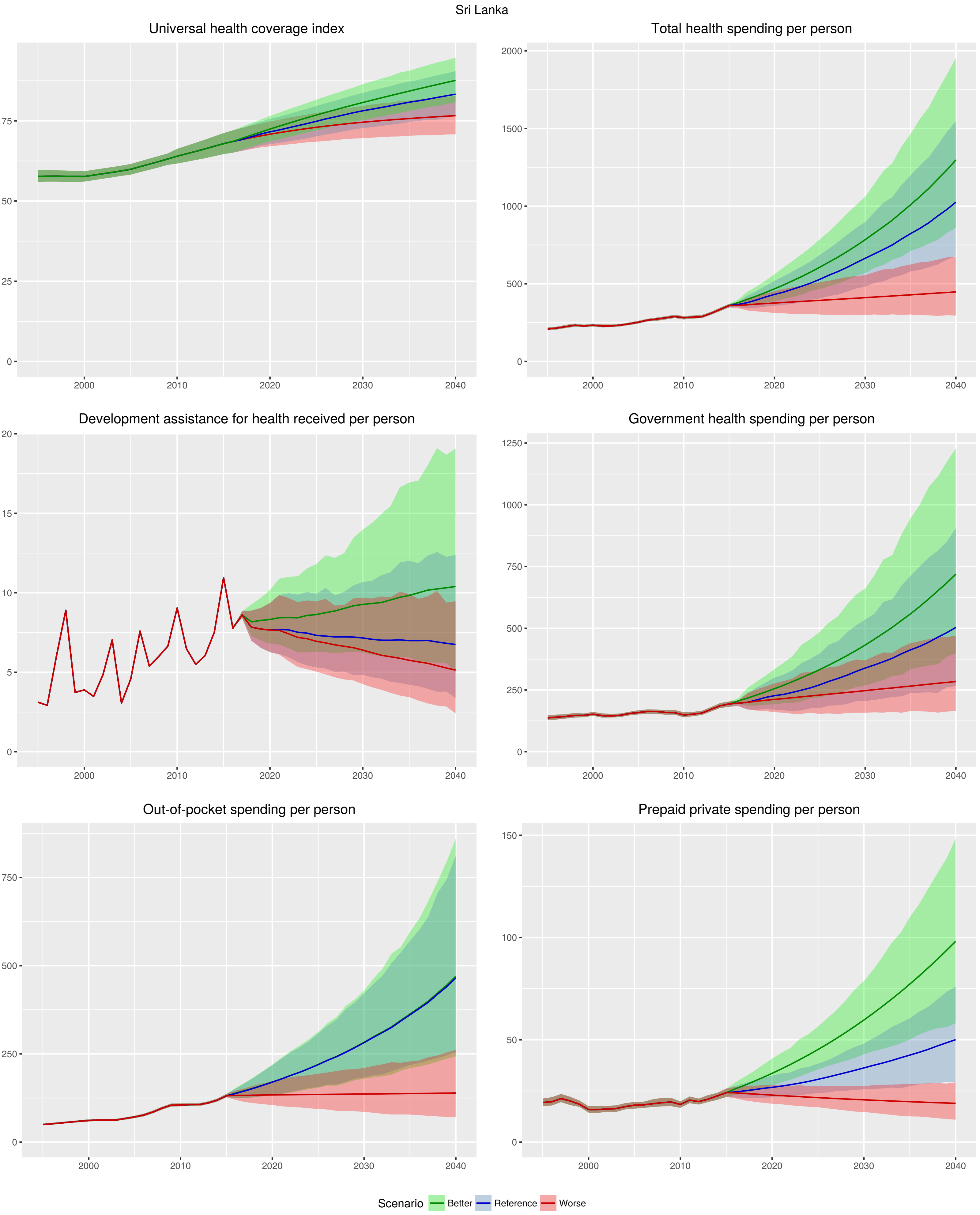

Sri Lanka
Universal health coverage index
Total health spending per person
Development assistance for health received per person
Government health spending per person
Out-of-pocket spending per person
Prepaid private spending per person
Scenario
Better
Reference
Worse

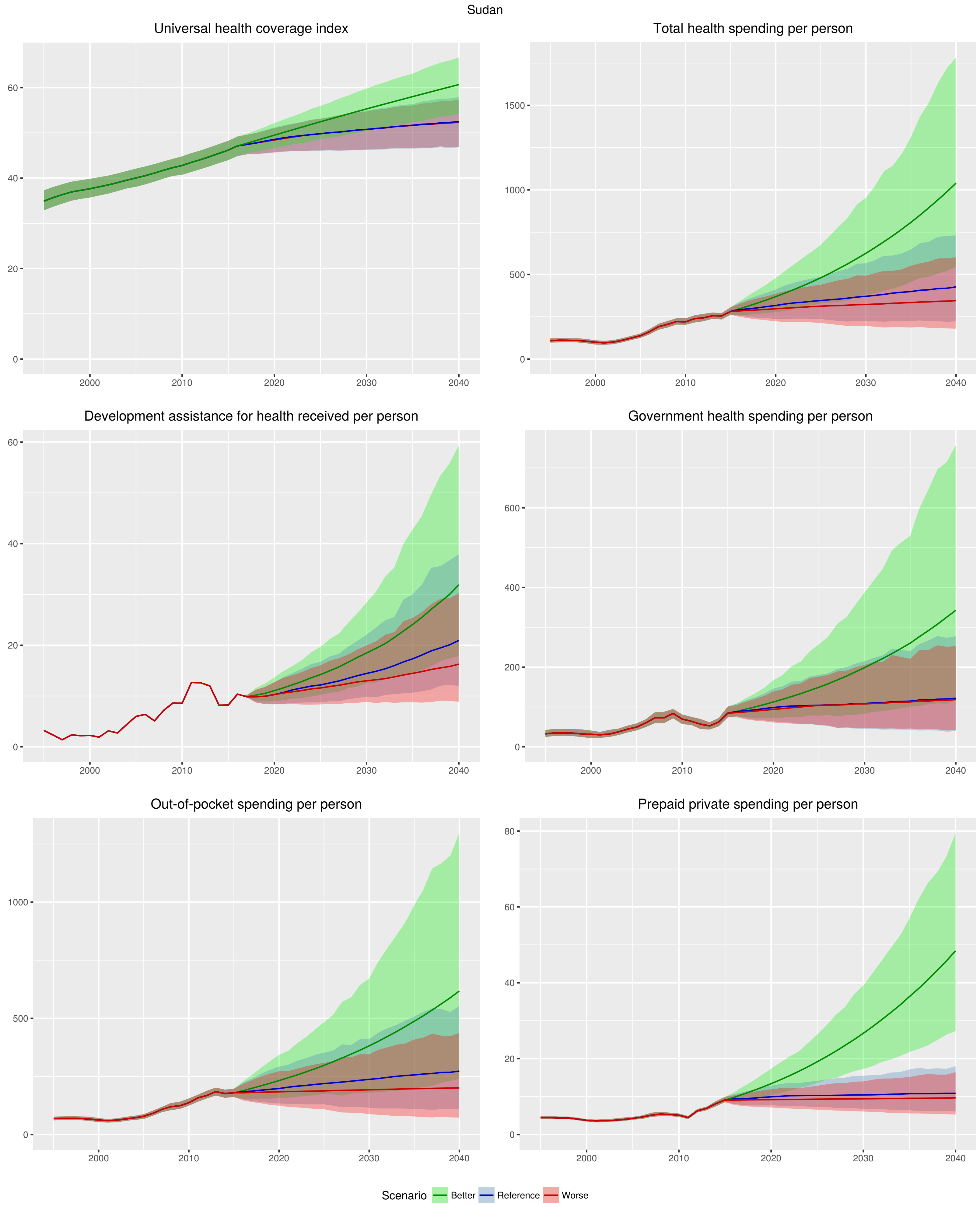

Sudan
Universal health coverage index
Total health spending per person
Development assistance for health received per person
Government health spending per person
Out-of-pocket spending per person
Prepaid private spending per person
Scenario
Better
Reference
Worse

Suriname

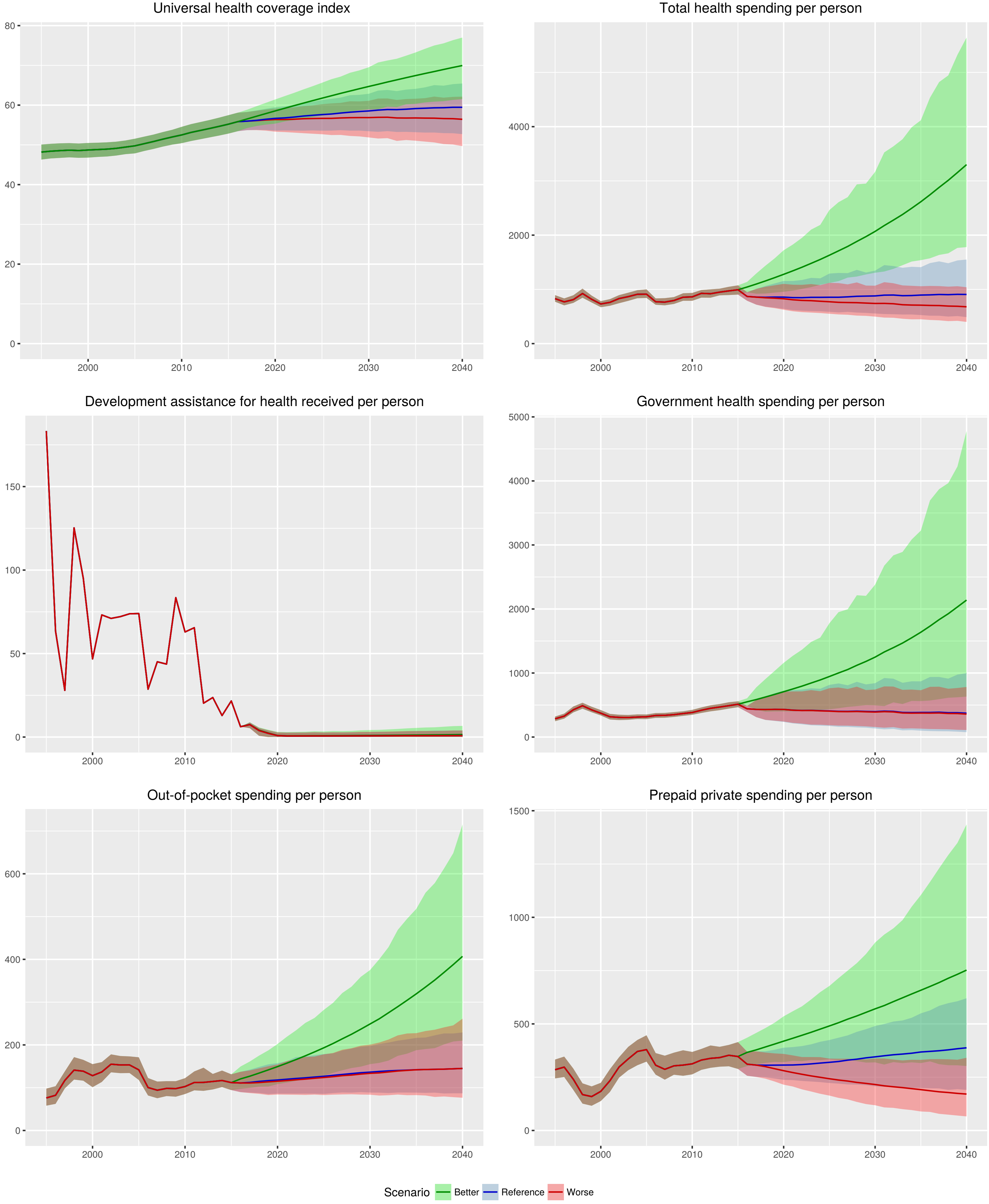

Universal health coverage index
Total health spending per person
Development assistance for health received per person
Government health spending per person
Out-of-pocket spending per person
Prepaid private spending per person
Scenario
Better
Reference
Worse

Swaziland

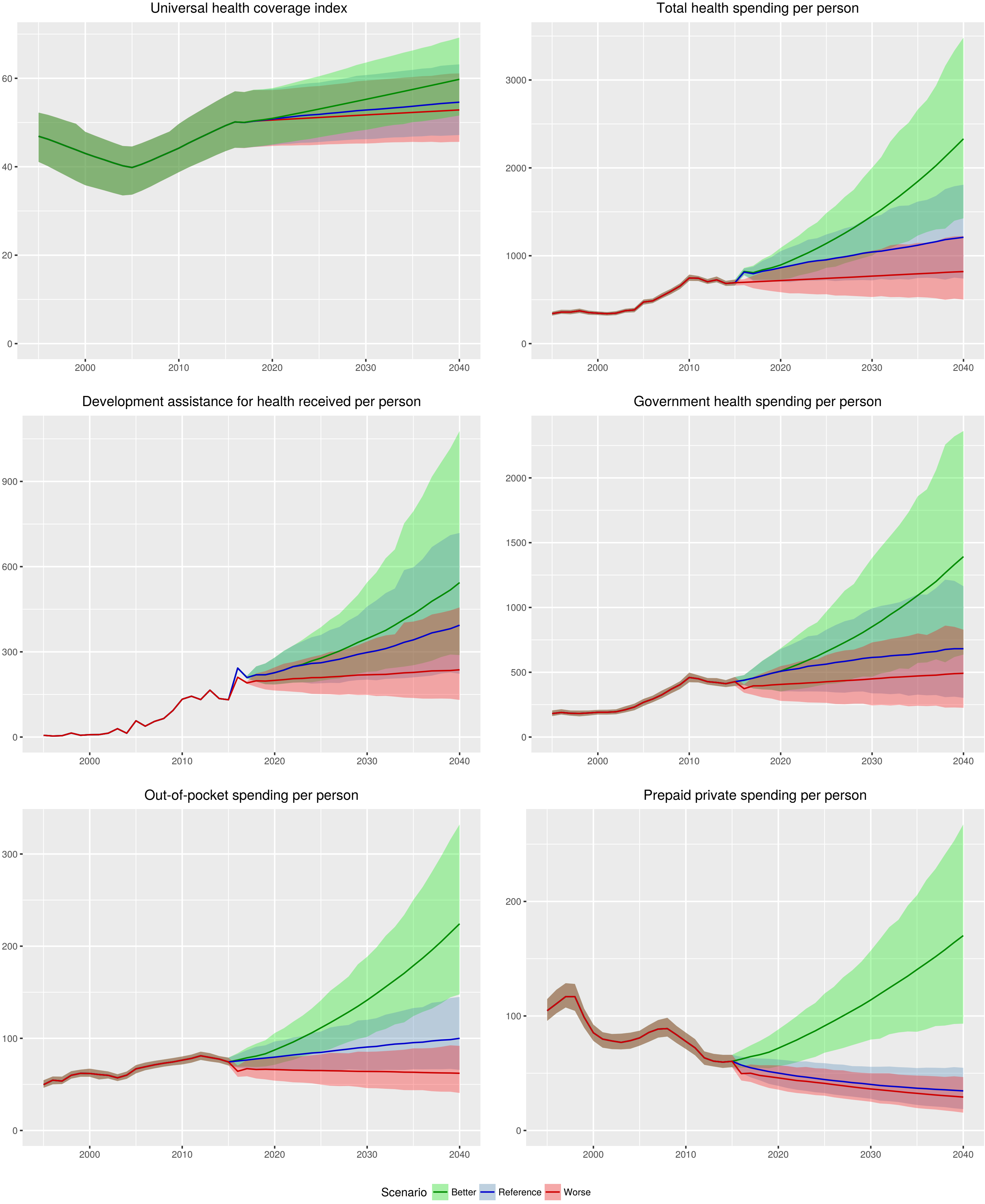

Universal health coverage index
Total health spending per person
Development assistance for health received per person
Government health spending per person
Out-of-pocket spending per person
Prepaid private spending per person
Scenario
Better
Reference
Worse

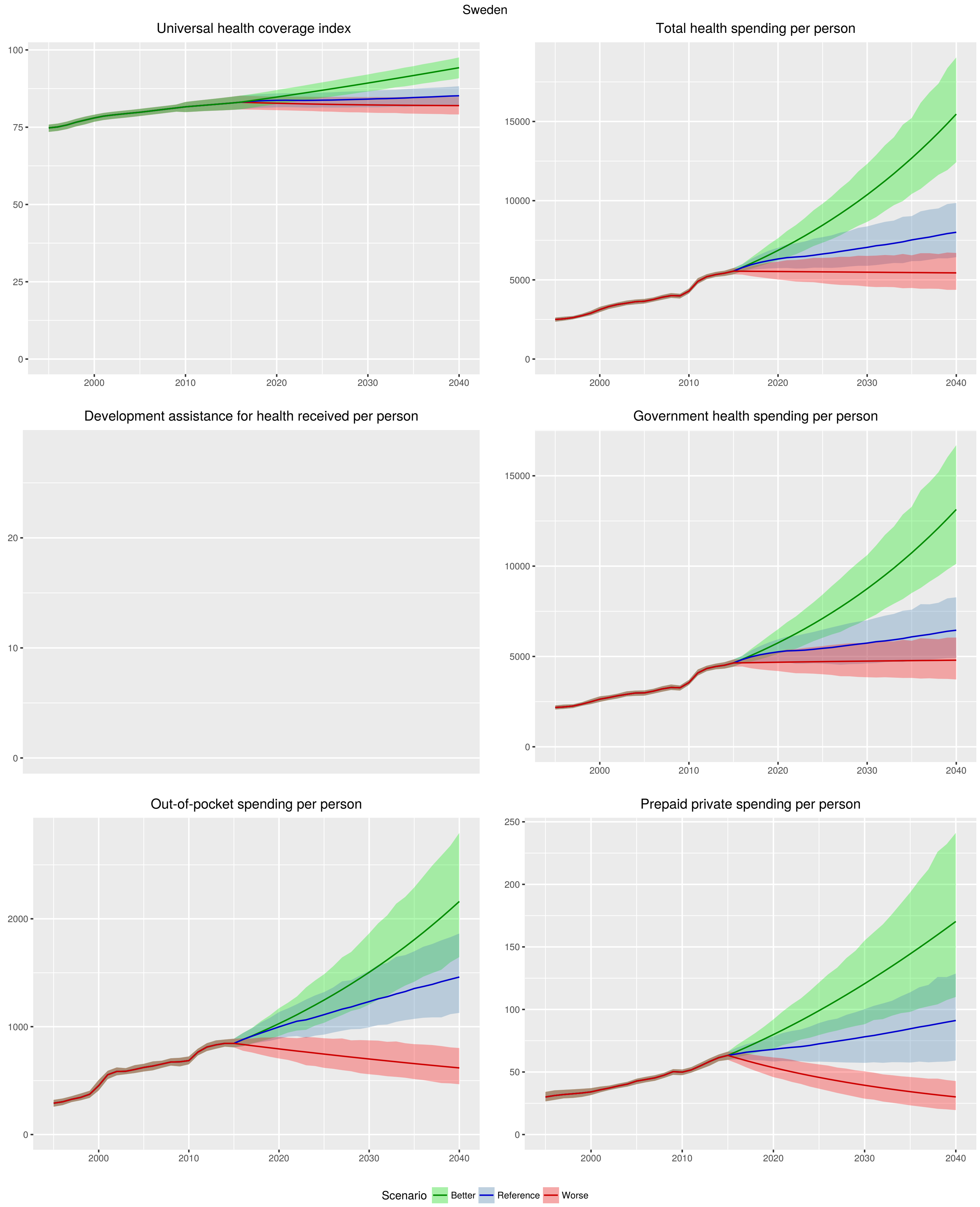
Sweden
Universal health coverage index
Total health spending per person
Development assistance for health received per person
Government health spending per person
Out-of-pocket spending per person
Prepaid private spending per person
Scenario
Better
Reference
Worse

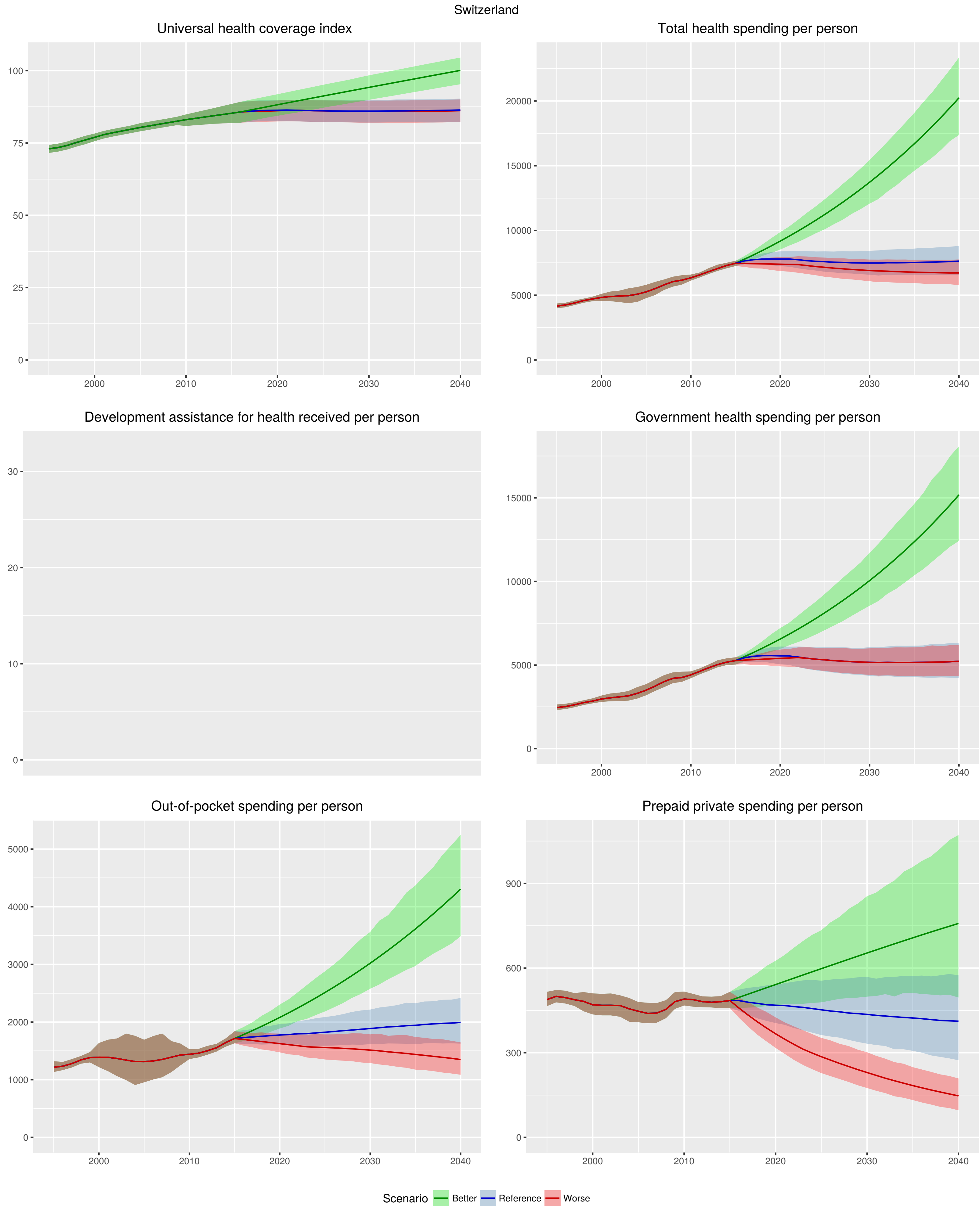
Switzerland
Universal health coverage index
Total health spending per person
Development assistance for health received per person
Government health spending per person
Out-of-pocket spending per person
Prepaid private spending per person
0
25
50
75
100
5000
10000
15000
20000
10
20
30
1000
2000
3000
4000
300
600
900
2000
2010
2020
2030
2040
Scenario
Better
Reference
Worse

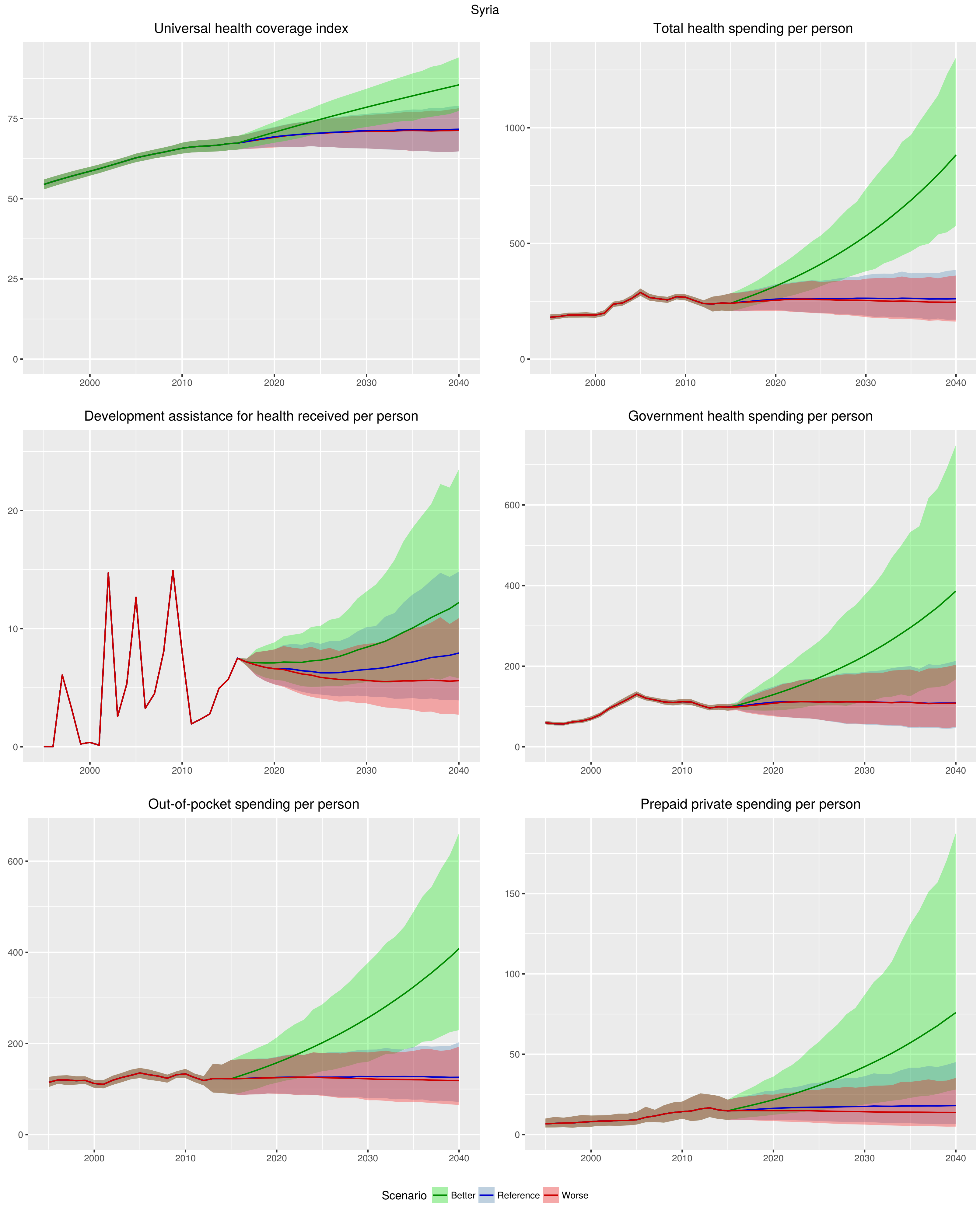
Syria
Universal health coverage index
Total health spending per person
Development assistance for health received per person
Government health spending per person
Out-of-pocket spending per person
Prepaid private spending per person
Scenario
Better
Reference
Worse

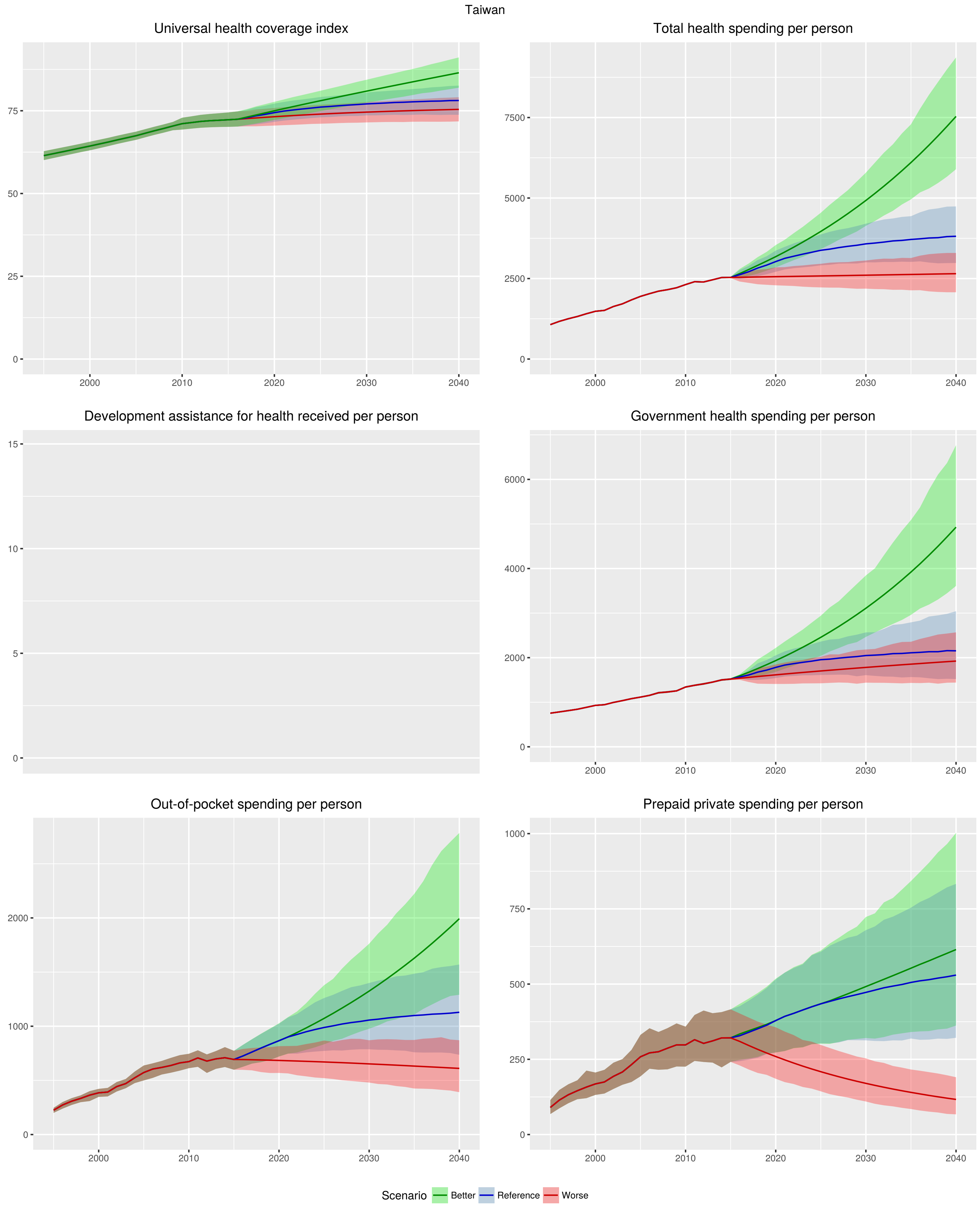

Taiwan
Universal health coverage index
Total health spending per person
Development assistance for health received per person
Government health spending per person
Out-of-pocket spending per person
Prepaid private spending per person
0
25
50
75
2500
5000
7500
5
10
15
2000
4000
6000
1000
250
500
750
2000
2010
2020
2030
2040
Scenario
Better
Reference
Worse

Tajikistan

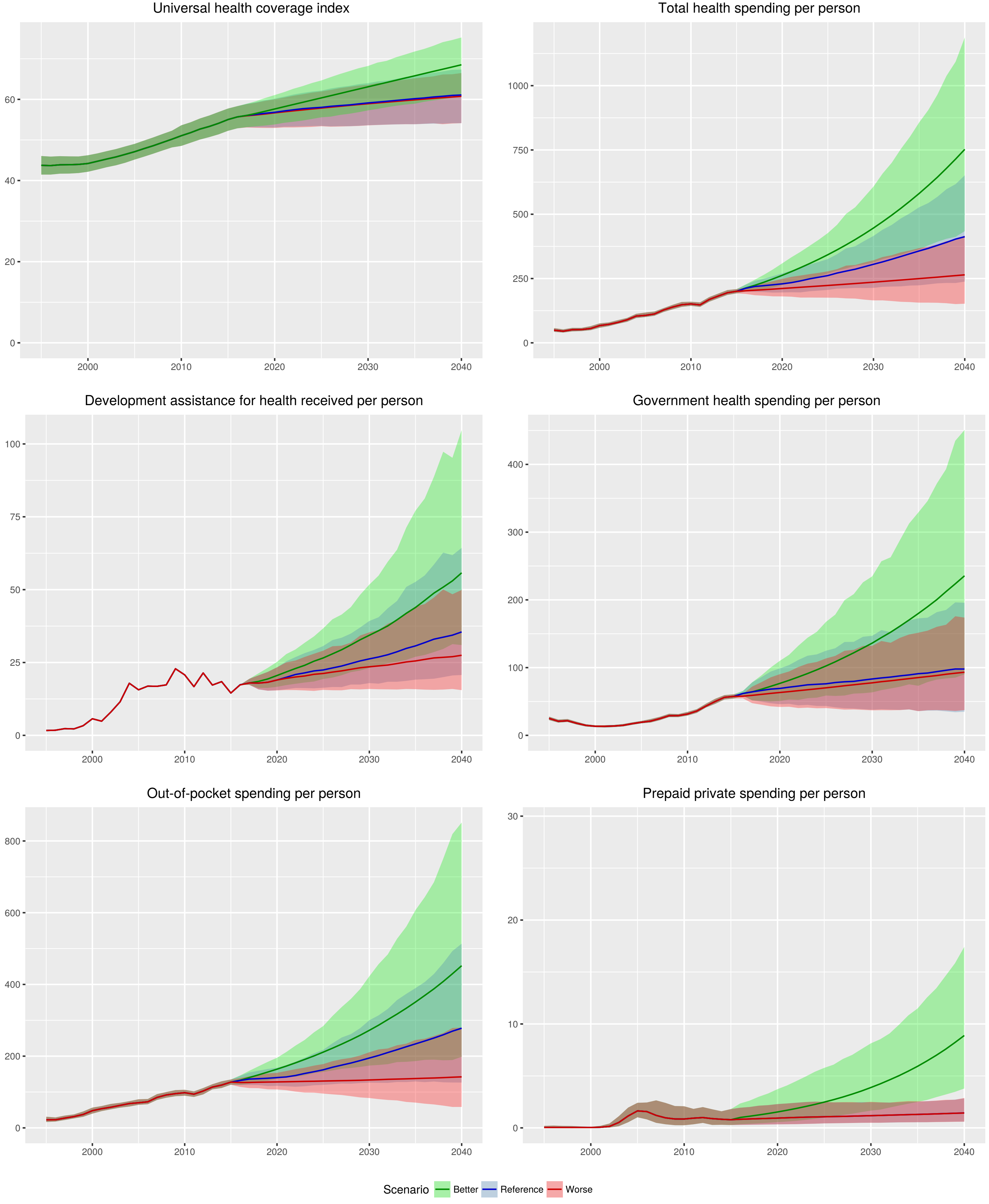

Universal health coverage index
Total health spending per person
Development assistance for health received per person
Government health spending per person
Out-of-pocket spending per person
Prepaid private spending per person
Scenario
Better
Reference
Worse

Tanzania

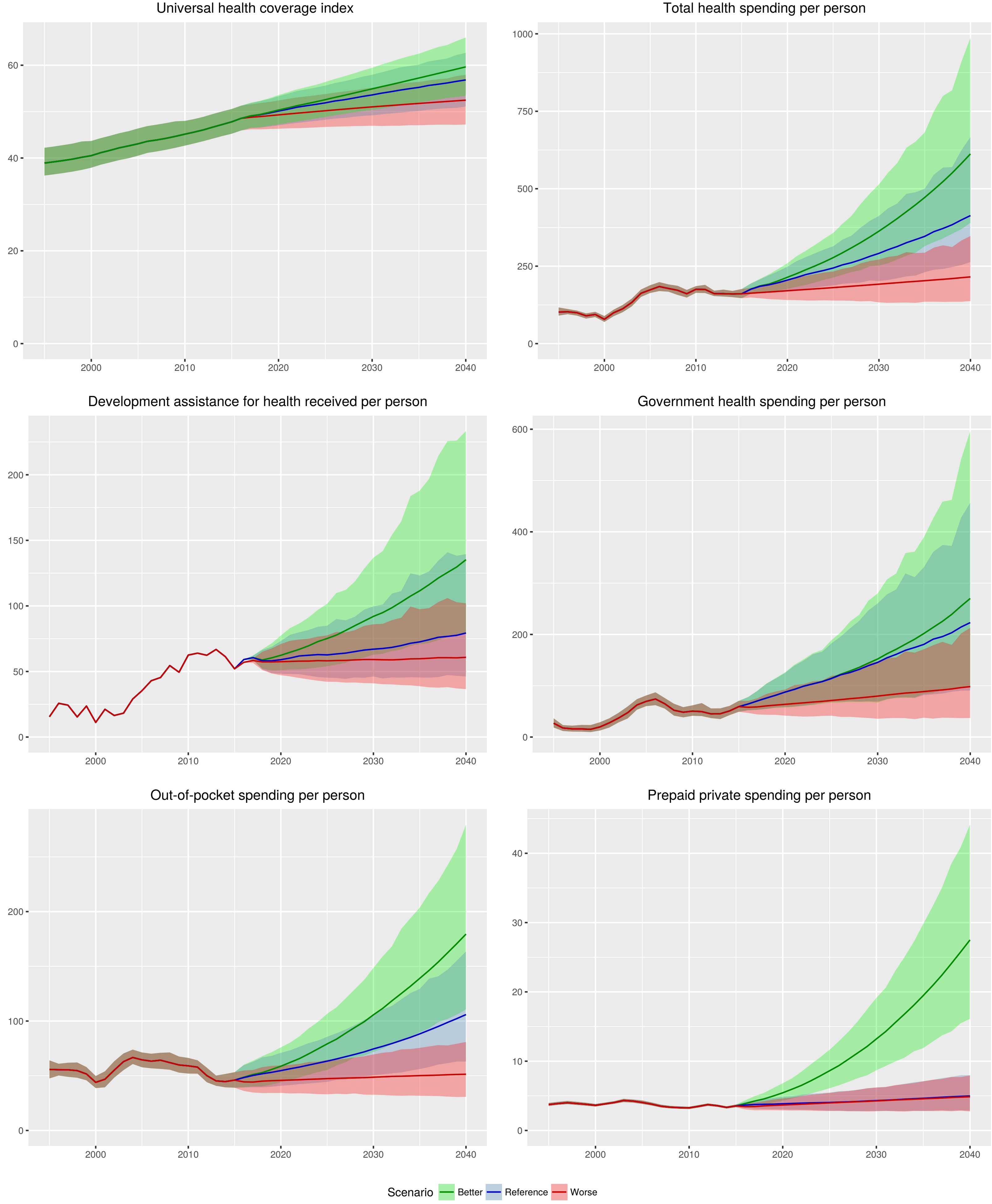

Universal health coverage index
Total health spending per person
Development assistance for health received per person
Government health spending per person
Out-of-pocket spending per person
Prepaid private spending per person
Scenario
Better
Reference
Worse

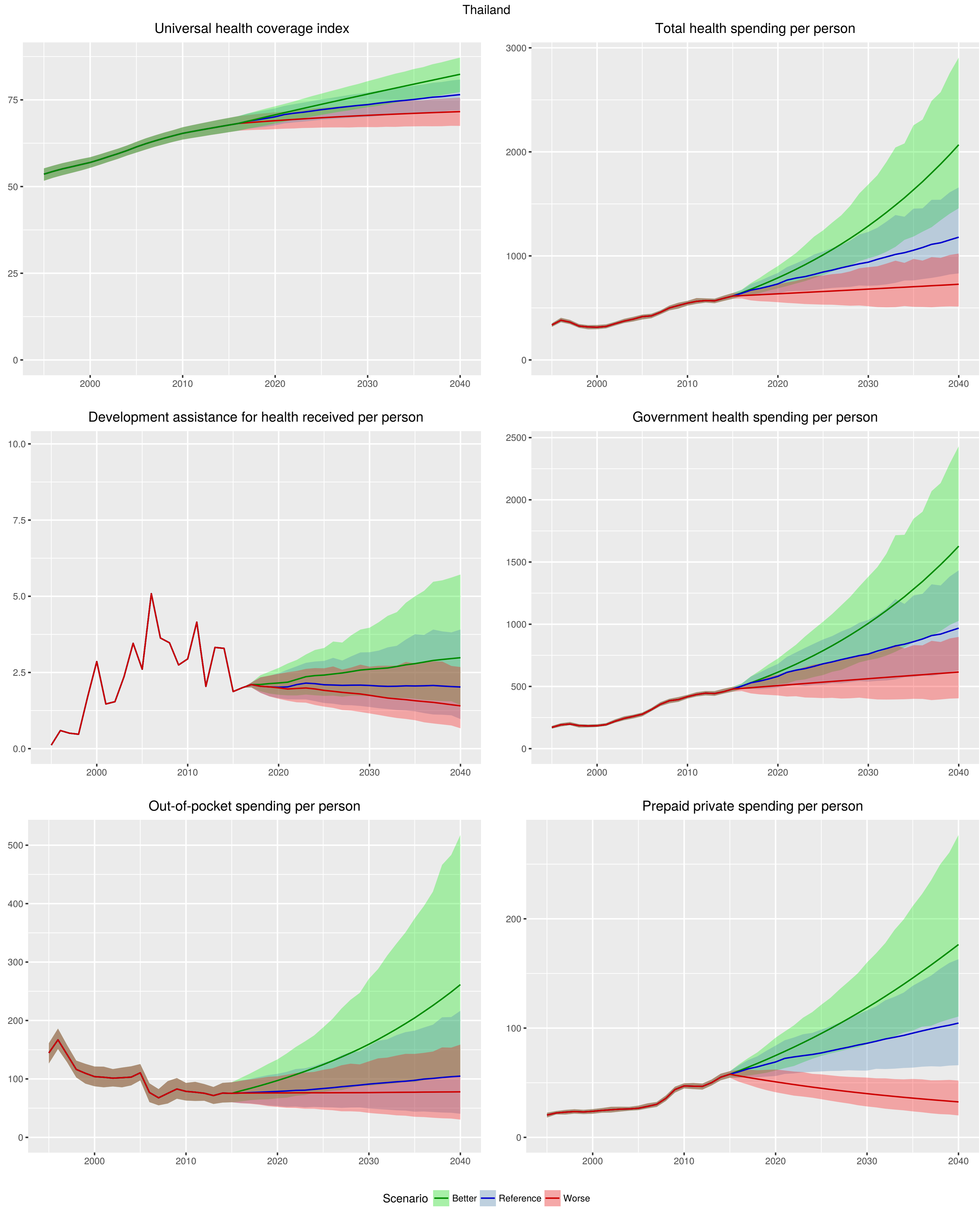

Thailand
Universal health coverage index
0
25
50
75
2000
2010
2020
2030
2040
Total health spending per person
0
1000
2000
3000
Development assistance for health received per person
0.0
2.5
5.0
7.5
10.0
Government health spending per person
0
500
1000
1500
2000
2500
Out-of-pocket spending per person
0
100
200
300
400
500
Prepaid private spending per person
0
100
200
Scenario
Better
Reference
Worse

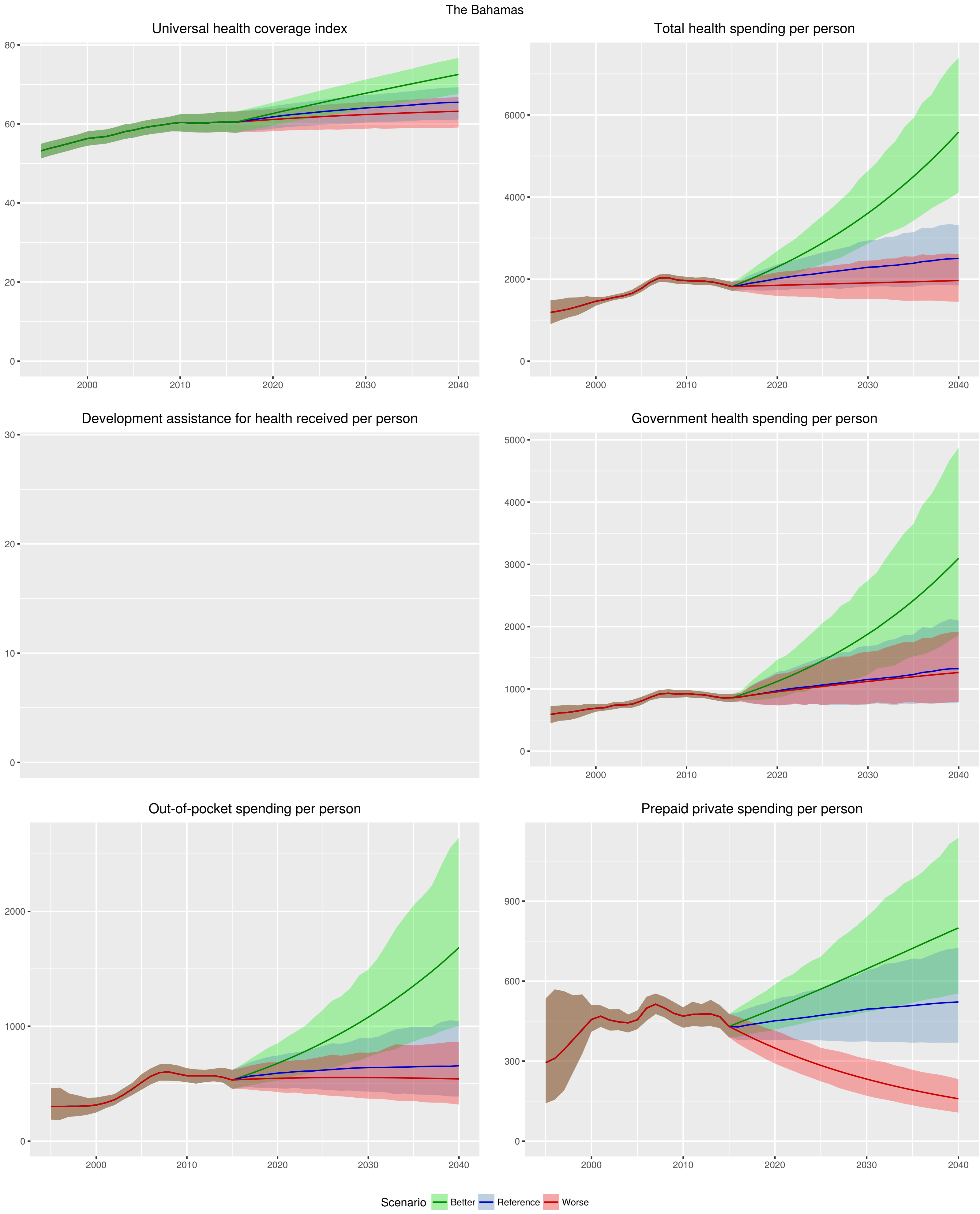
The Bahamas
Universal health coverage index
Total health spending per person
Development assistance for health received per person
Government health spending per person
Out-of-pocket spending per person
Prepaid private spending per person
0
20
40
60
80
2000
2010
2020
2030
2040
2000
4000
6000
10
30
1000
3000
5000
300
600
900
Scenario
Better
Reference
Worse

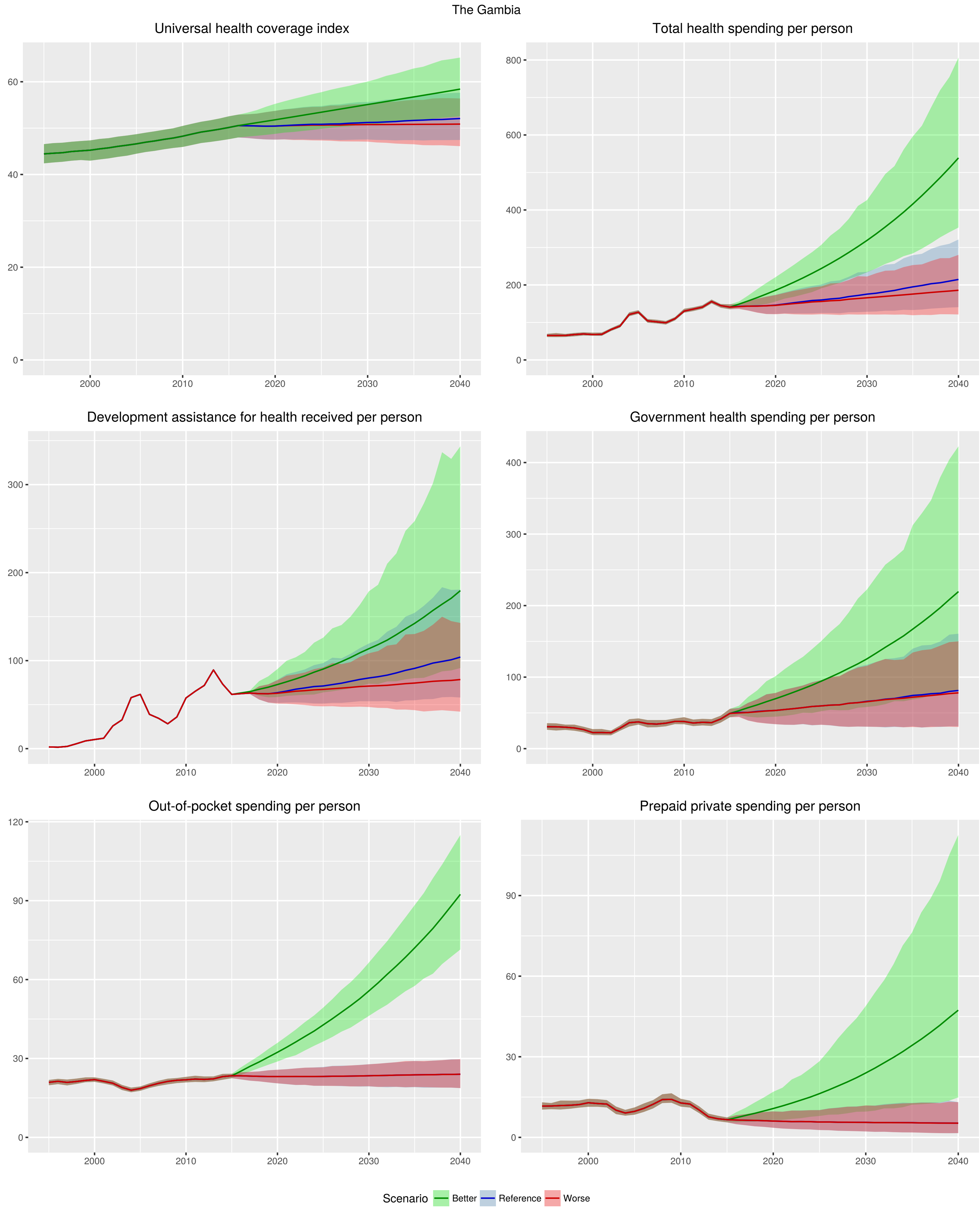

The Gambia
Universal health coverage index
Total health spending per person
Development assistance for health received per person
Government health spending per person
Out-of-pocket spending per person
Prepaid private spending per person
0
20
40
60
200
400
600
800
100
300
30
90
120
2000
2010
2020
2030
2040
Scenario
Better
Reference
Worse

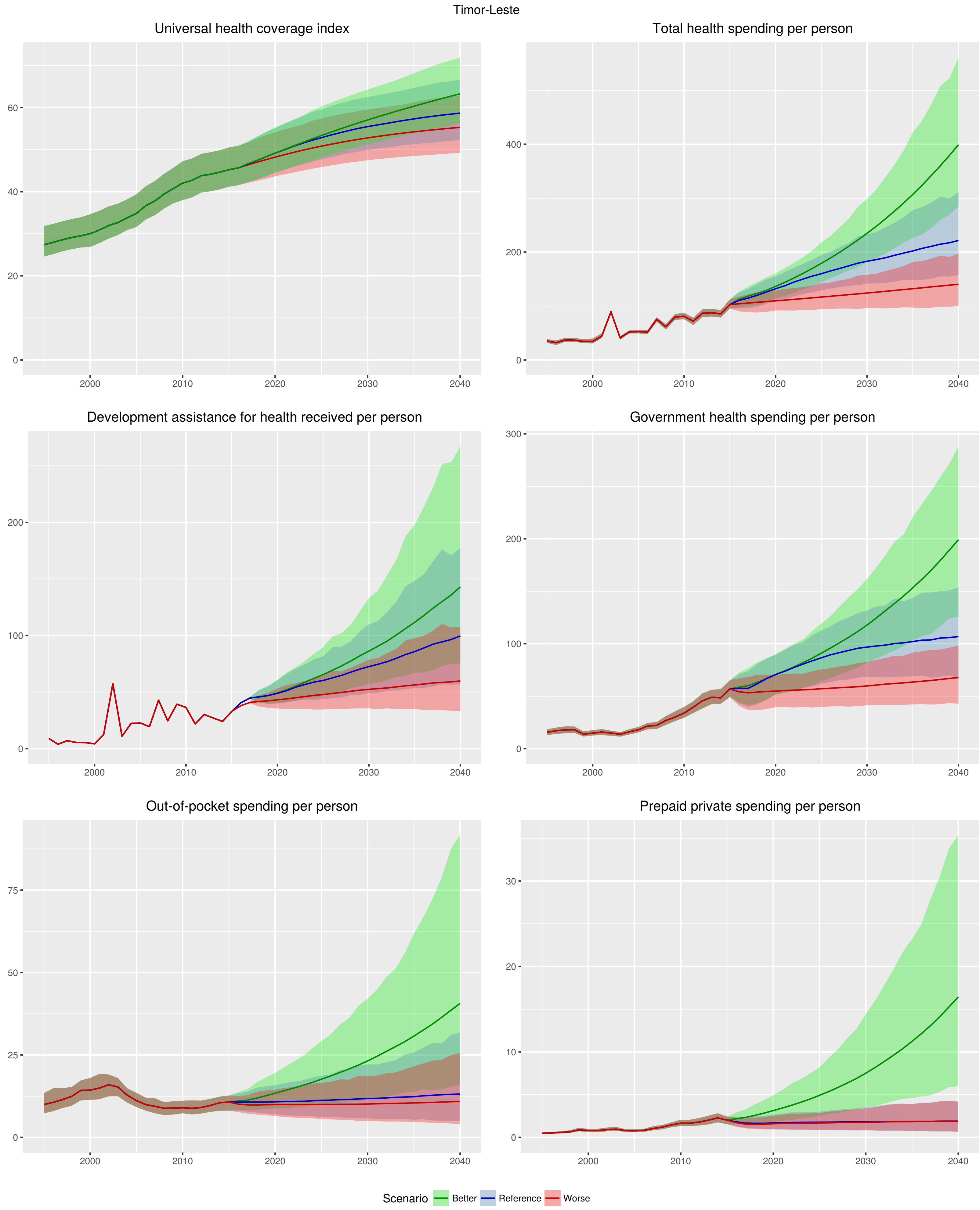
Timor-Leste
Universal health coverage index
Total health spending per person
Development assistance for health received per person
Government health spending per person
Out-of-pocket spending per person
Prepaid private spending per person
Scenario
Better
Reference
Worse

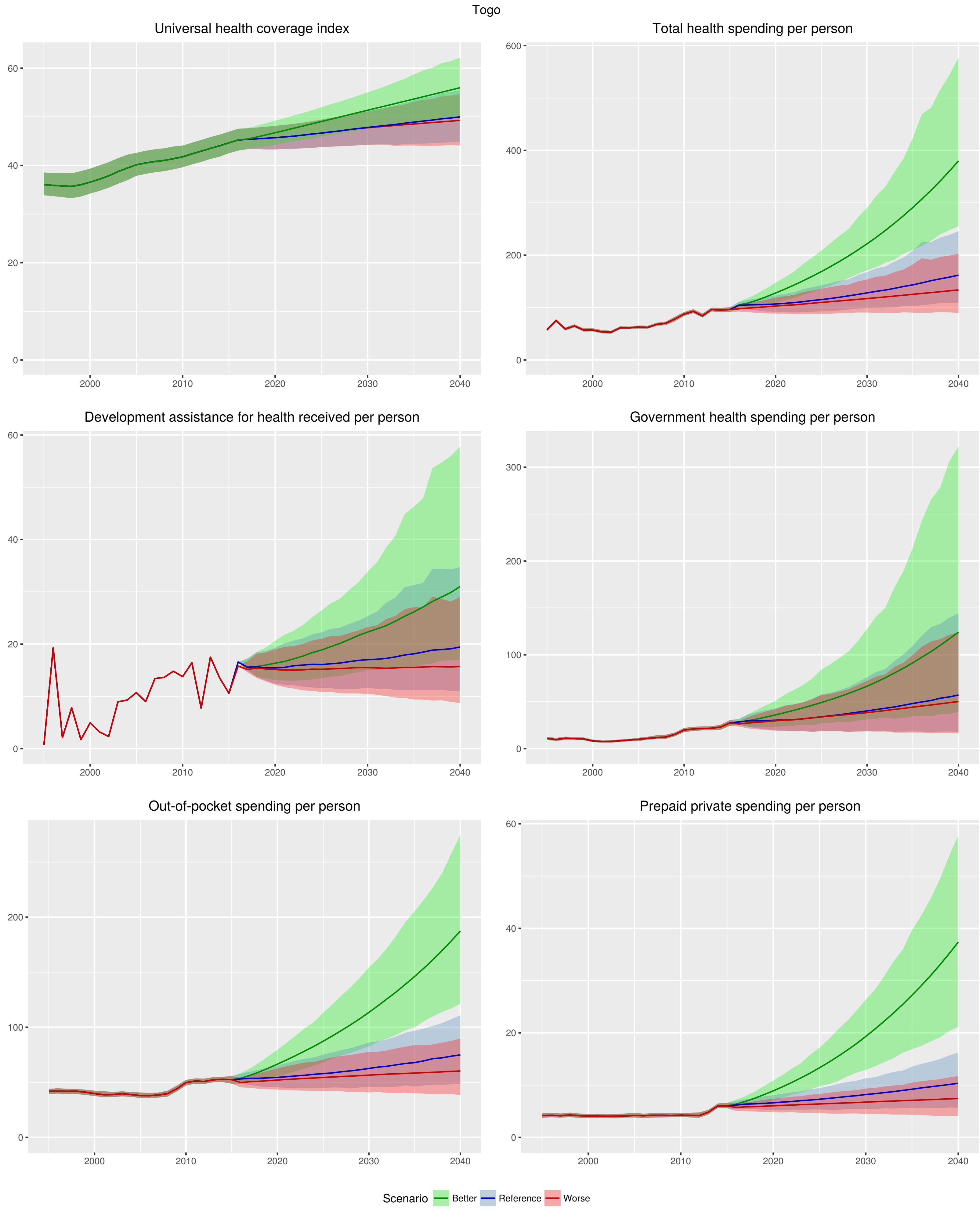

Togo
Universal health coverage index
0
20
40
60
2000
2010
2020
2030
2040
Total health spending per person
0
200
400
600
2000
2010
2020
2030
2040
Development assistance for health received per person
0
20
40
60
2000
2010
2020
2030
2040
Government health spending per person
0
100
200
300
2000
2010
2020
2030
2040
Out-of-pocket spending per person
0
100
200
2000
2010
2020
2030
2040
Prepaid private spending per person
0
20
40
60
2000
2010
2020
2030
2040
Scenario
Better
Reference
Worse

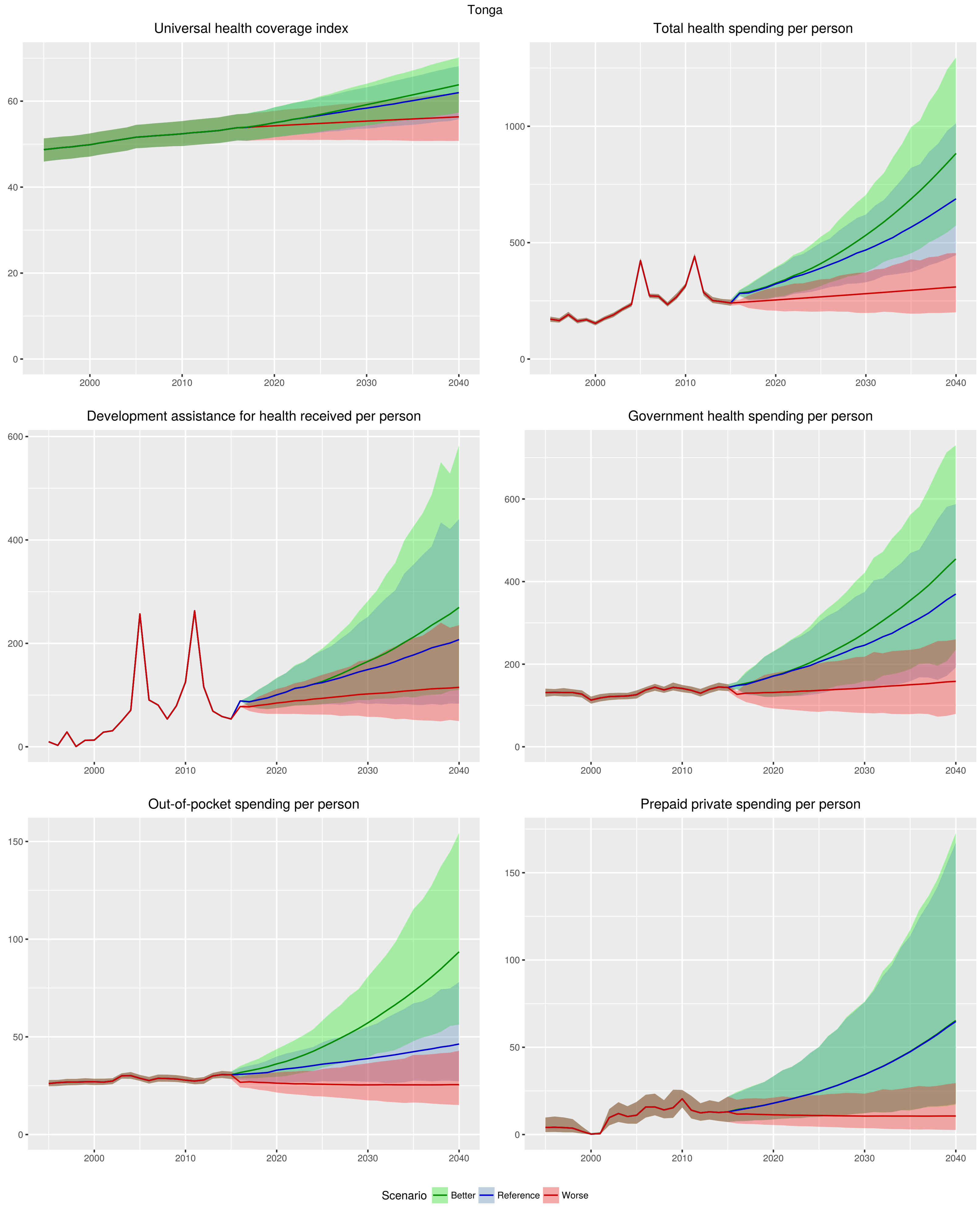

Tonga
Universal health coverage index
Total health spending per person
Development assistance for health received per person
Government health spending per person
Out-of-pocket spending per person
Prepaid private spending per person
Scenario
Better
Reference
Worse

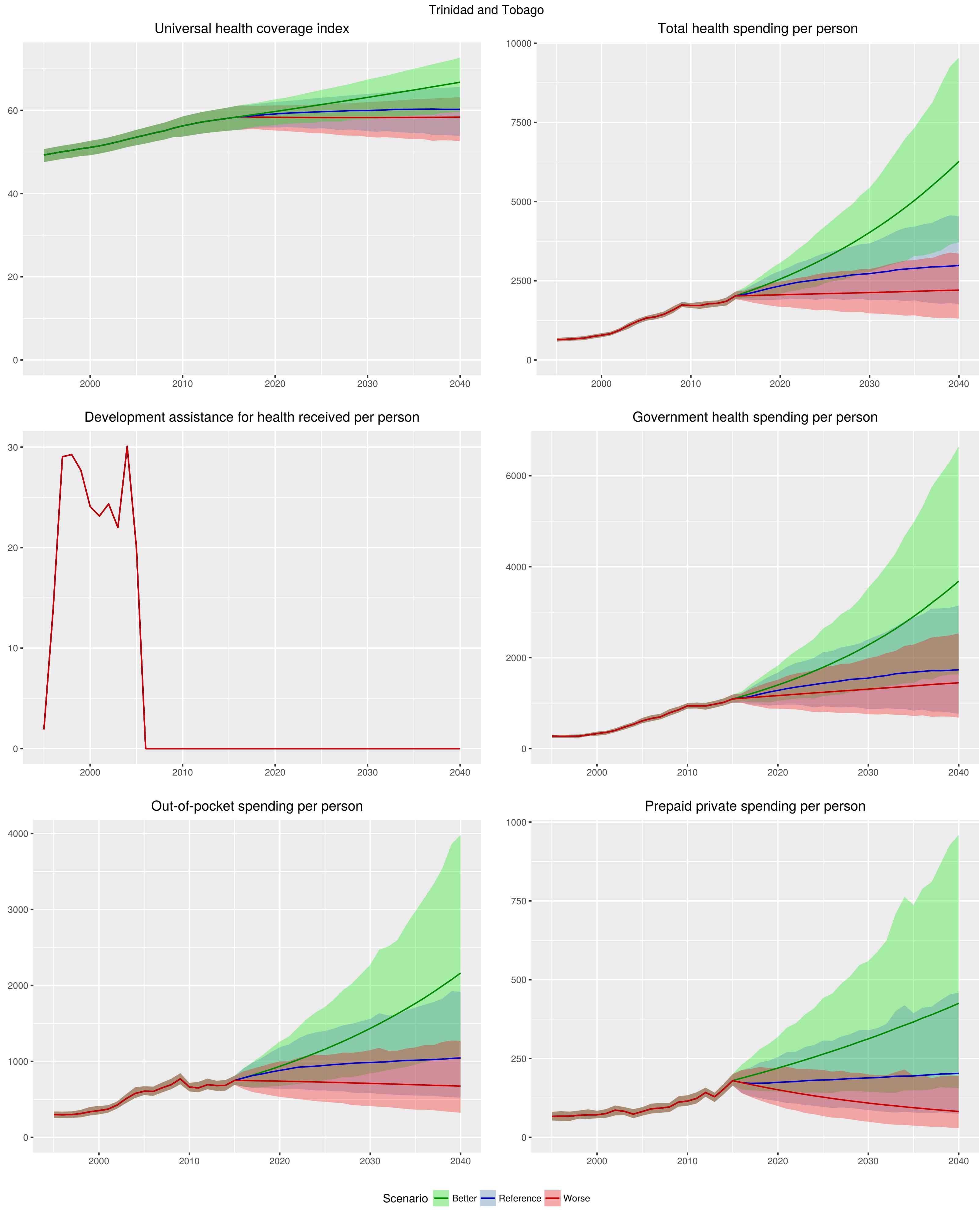
Trinidad and Tobago
Universal health coverage index
Total health spending per person
Development assistance for health received per person
Government health spending per person
Out-of-pocket spending per person
Prepaid private spending per person
2000
2010
2020
2030
2040
0
20
40
60
2500
5000
7500
10000
10
30
2000
4000
6000
1000
3000
250
500
750
1000
Scenario
Better
Reference
Worse

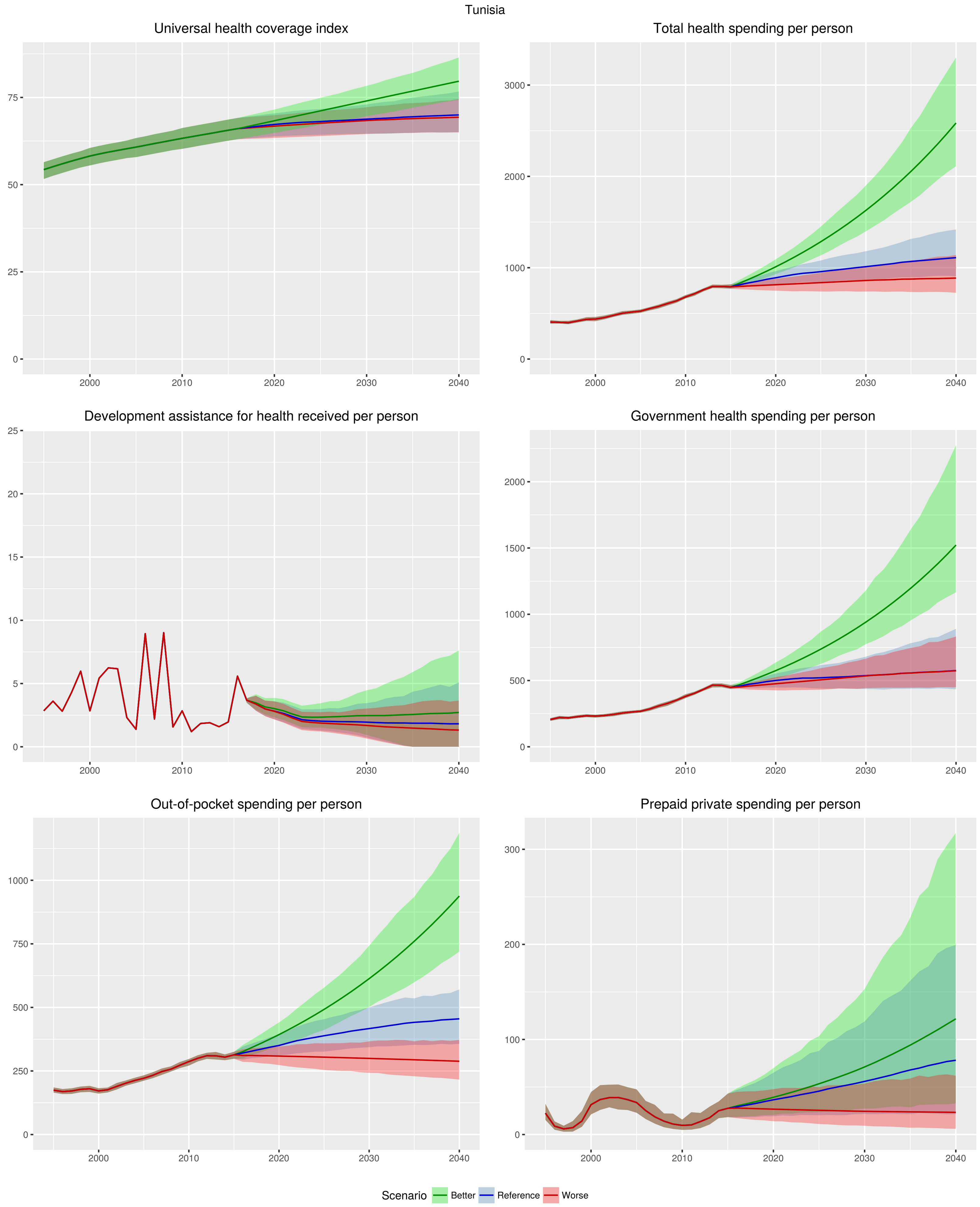
Tunisia
Universal health coverage index
Total health spending per person
Development assistance for health received per person
Government health spending per person
Out-of-pocket spending per person
Prepaid private spending per person
Scenario
Better
Reference
Worse

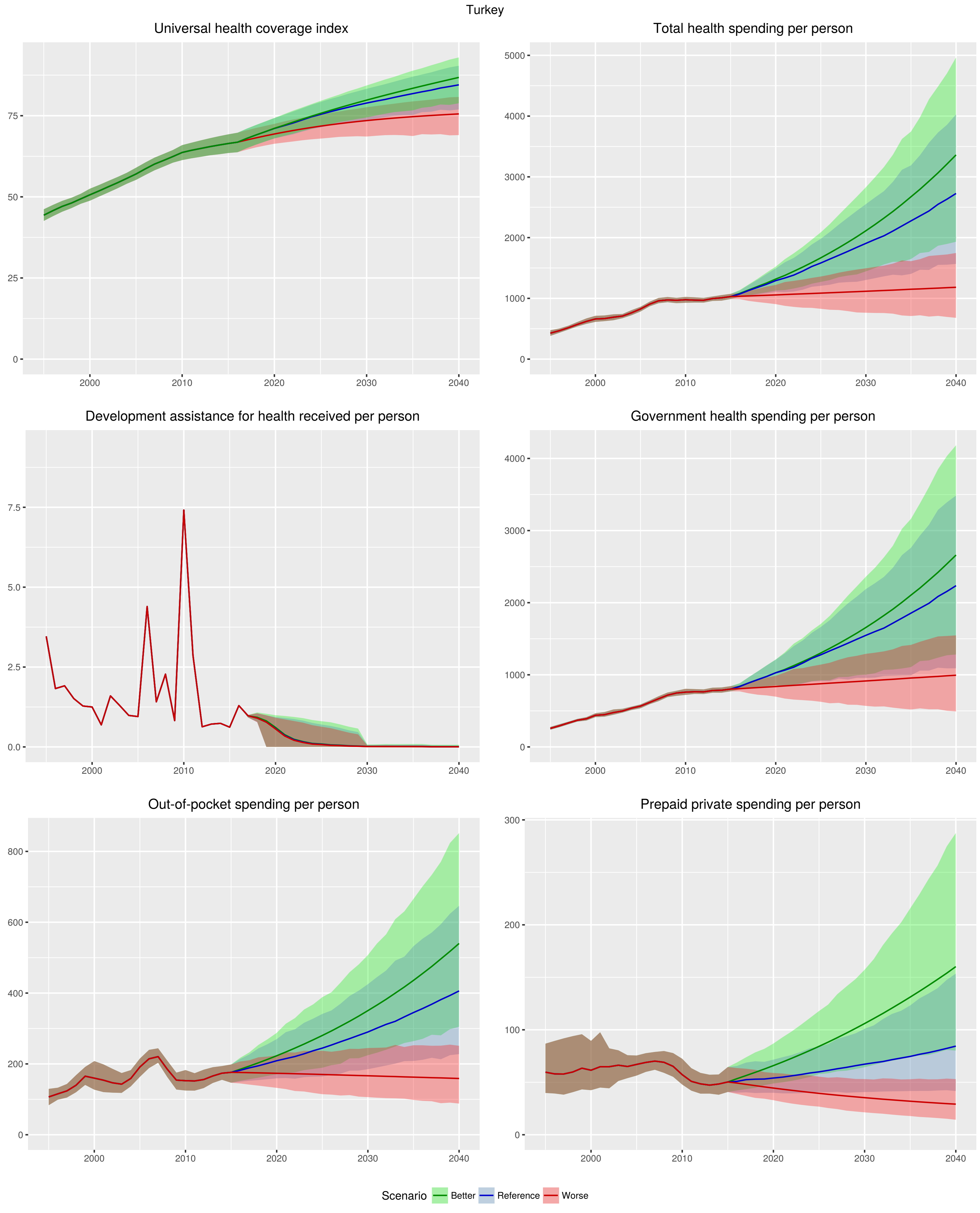
Turkey
Universal health coverage index
Total health spending per person
Development assistance for health received per person
Government health spending per person
Out-of-pocket spending per person
Prepaid private spending per person
0
25
50
75
0
1000
2000
3000
4000
5000
0.0
2.5
5.0
7.5
0
1000
2000
3000
4000
0
200
400
600
800
0
100
200
300
2000
2010
2020
2030
2040
Scenario
Better
Reference
Worse

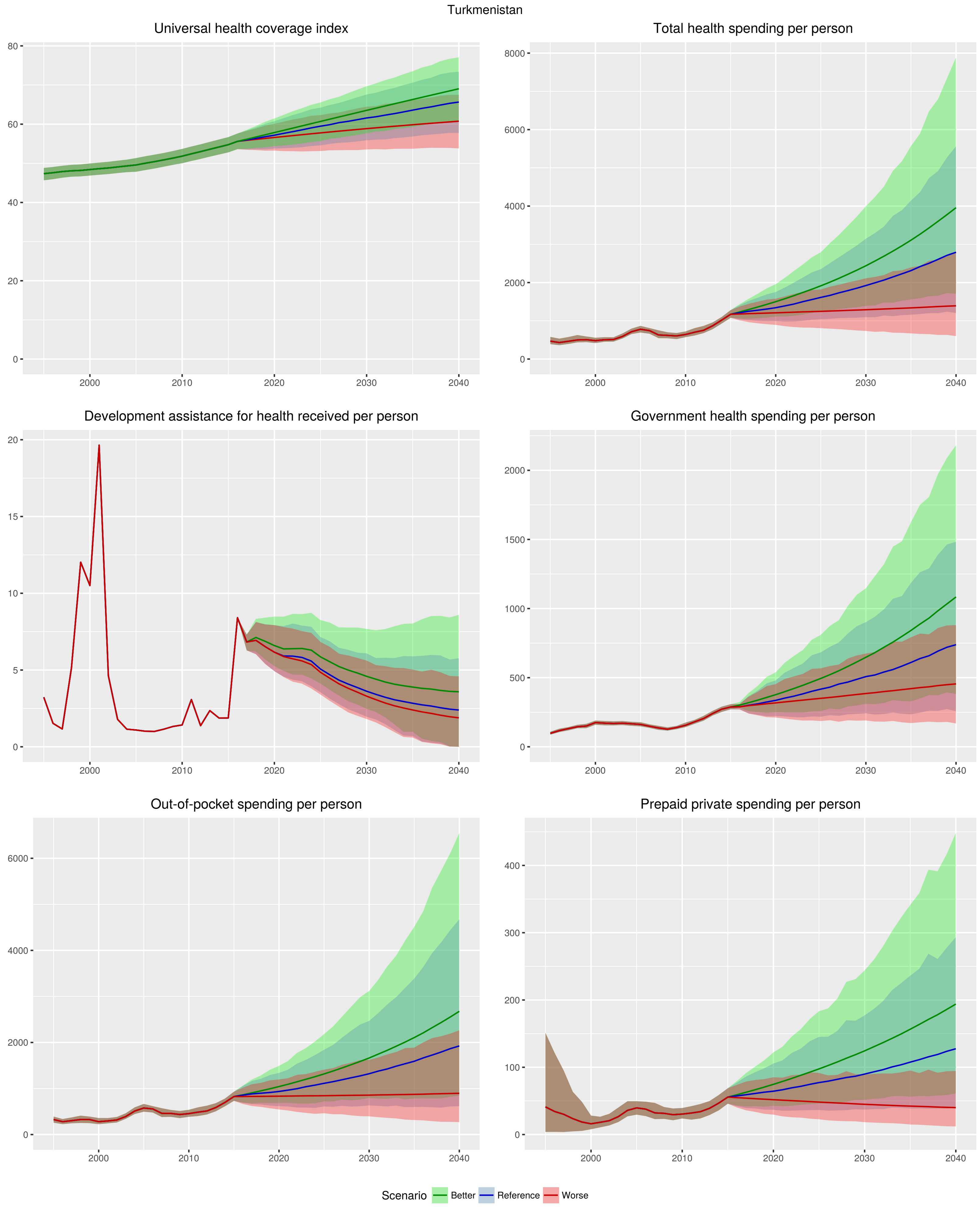

Turkmenistan
Universal health coverage index
Total health spending per person
Development assistance for health received per person
Government health spending per person
Out-of-pocket spending per person
Prepaid private spending per person
Scenario
Better
Reference
Worse

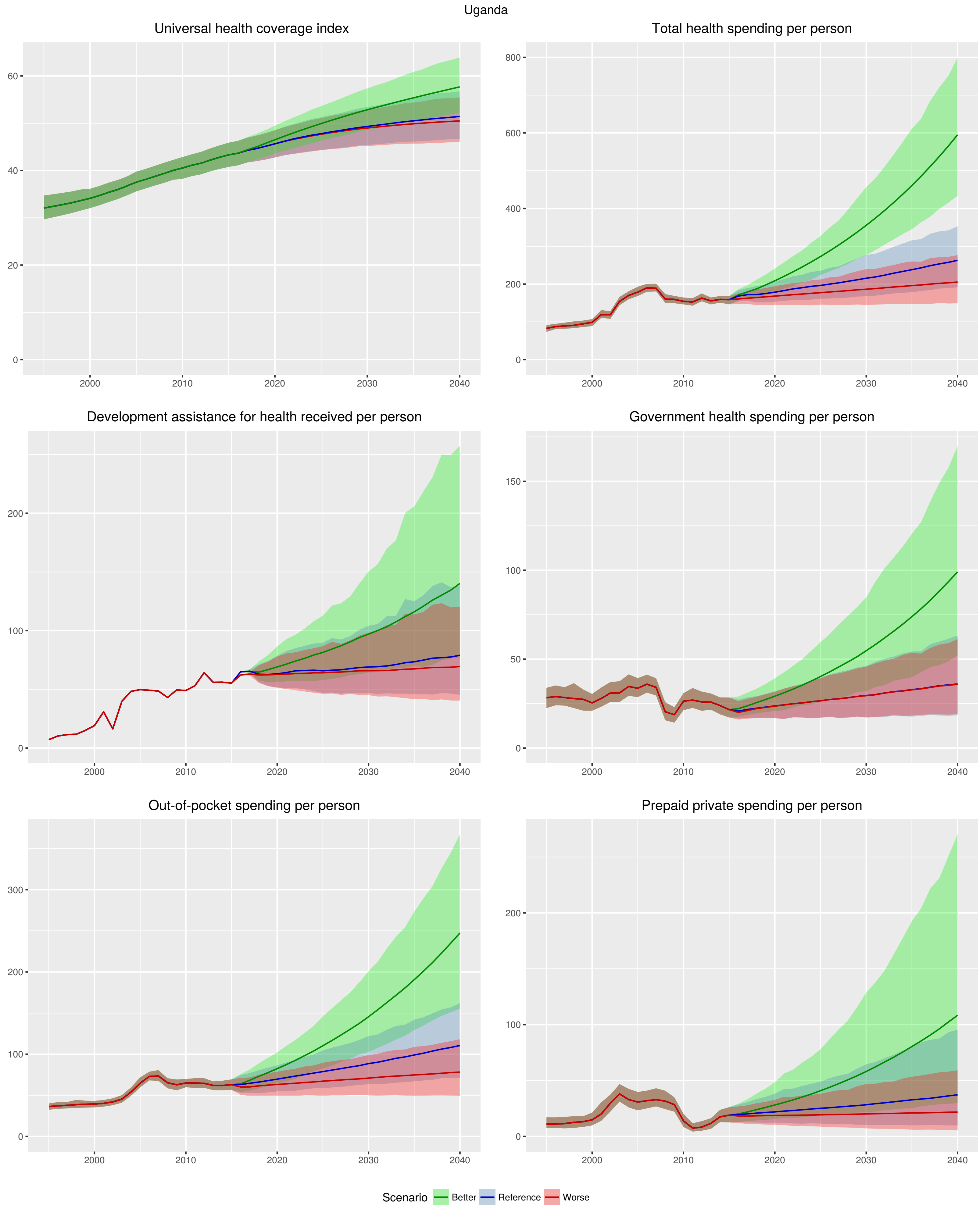
Uganda
Universal health coverage index
0
20
40
60
Total health spending per person
0
200
400
600
800
Development assistance for health received per person
0
100
200
Government health spending per person
0
50
100
150
Out-of-pocket spending per person
0
100
200
300
Prepaid private spending per person
0
100
200
2000
2010
2020
2030
2040
Scenario
Better
Reference
Worse

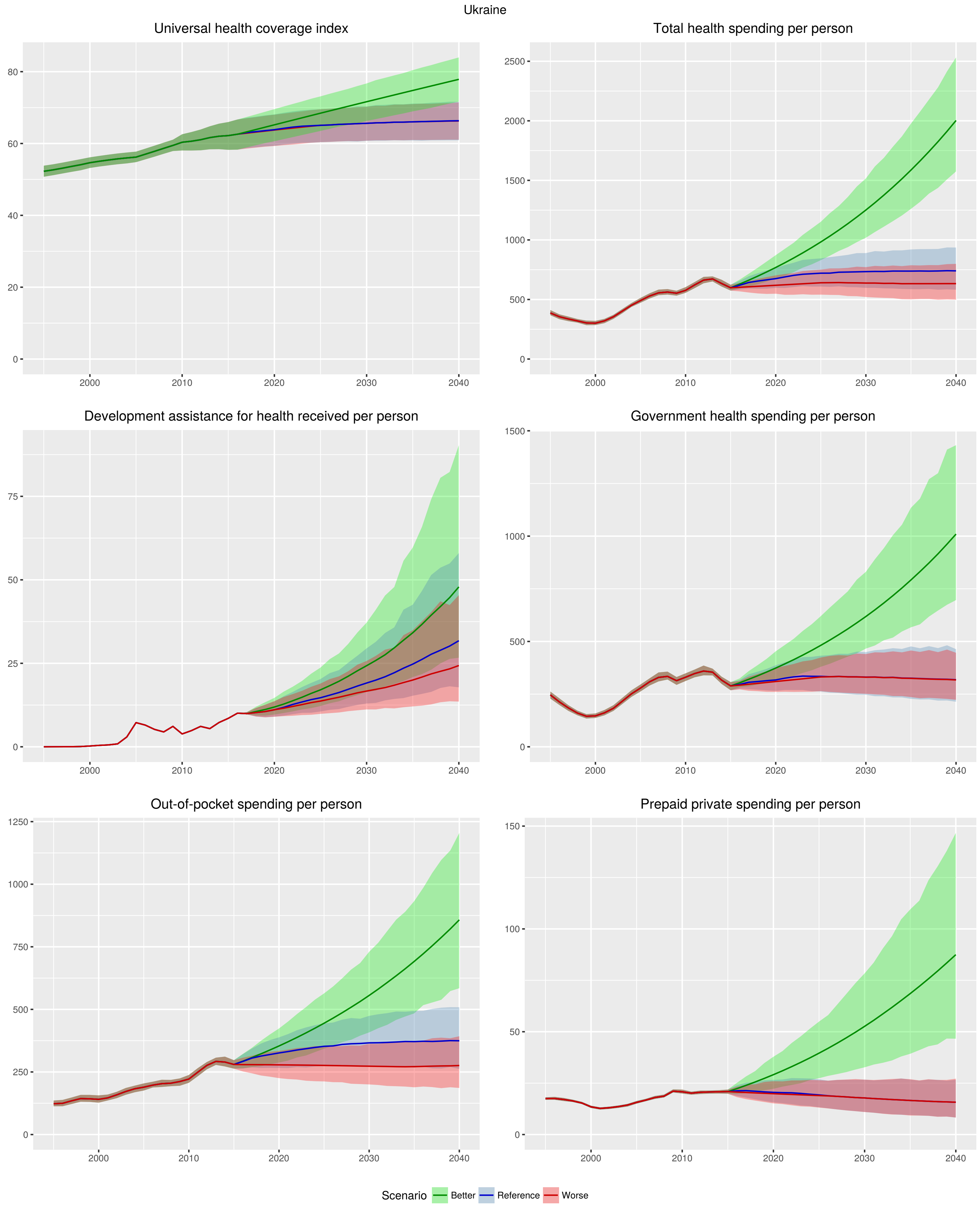
Ukraine
Universal health coverage index
Total health spending per person
Development assistance for health received per person
Government health spending per person
Out-of-pocket spending per person
Prepaid private spending per person
2000
2010
2020
2030
2040
Scenario
Better
Reference
Worse

United Arab Emirates

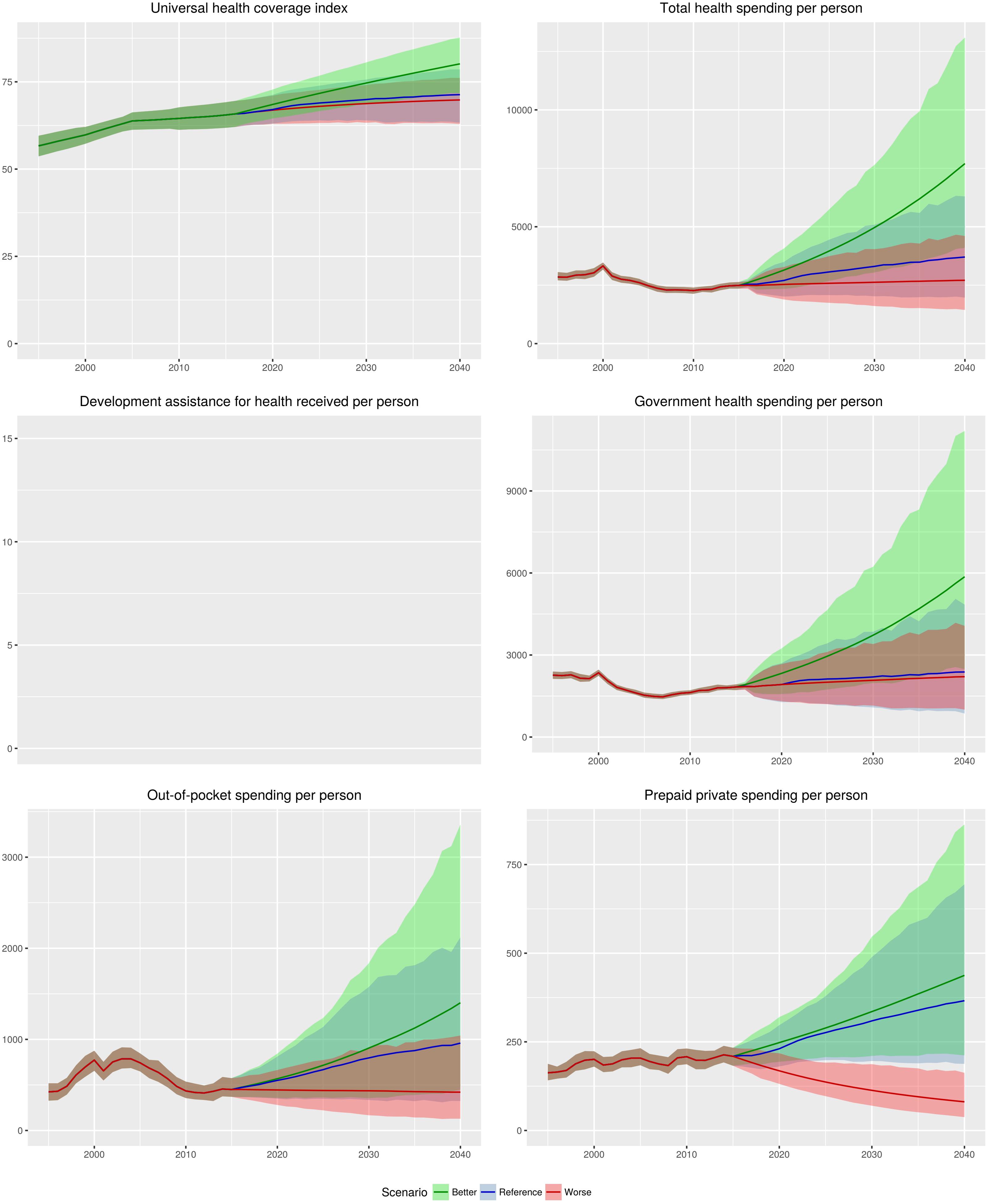

United Kingdom

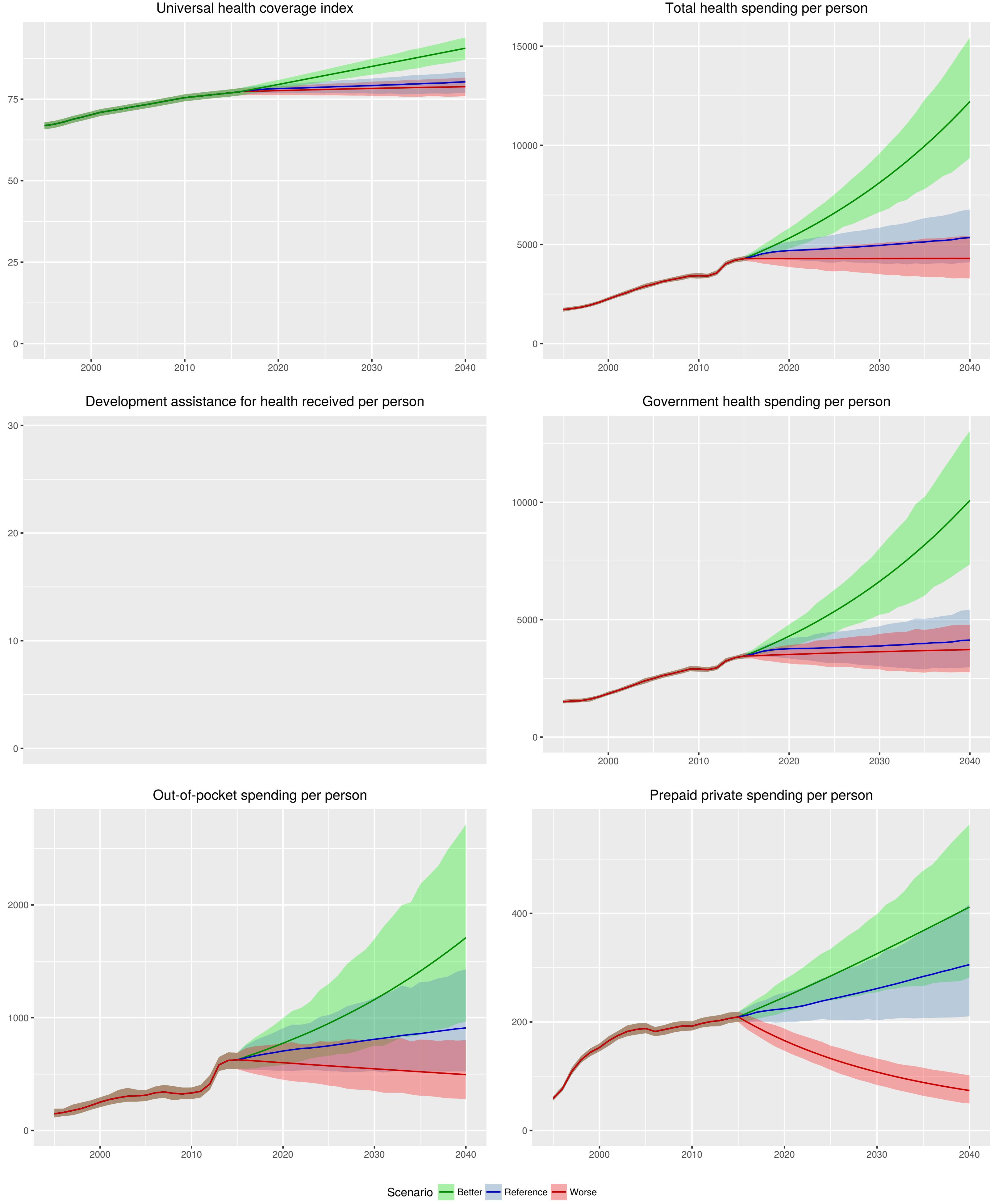

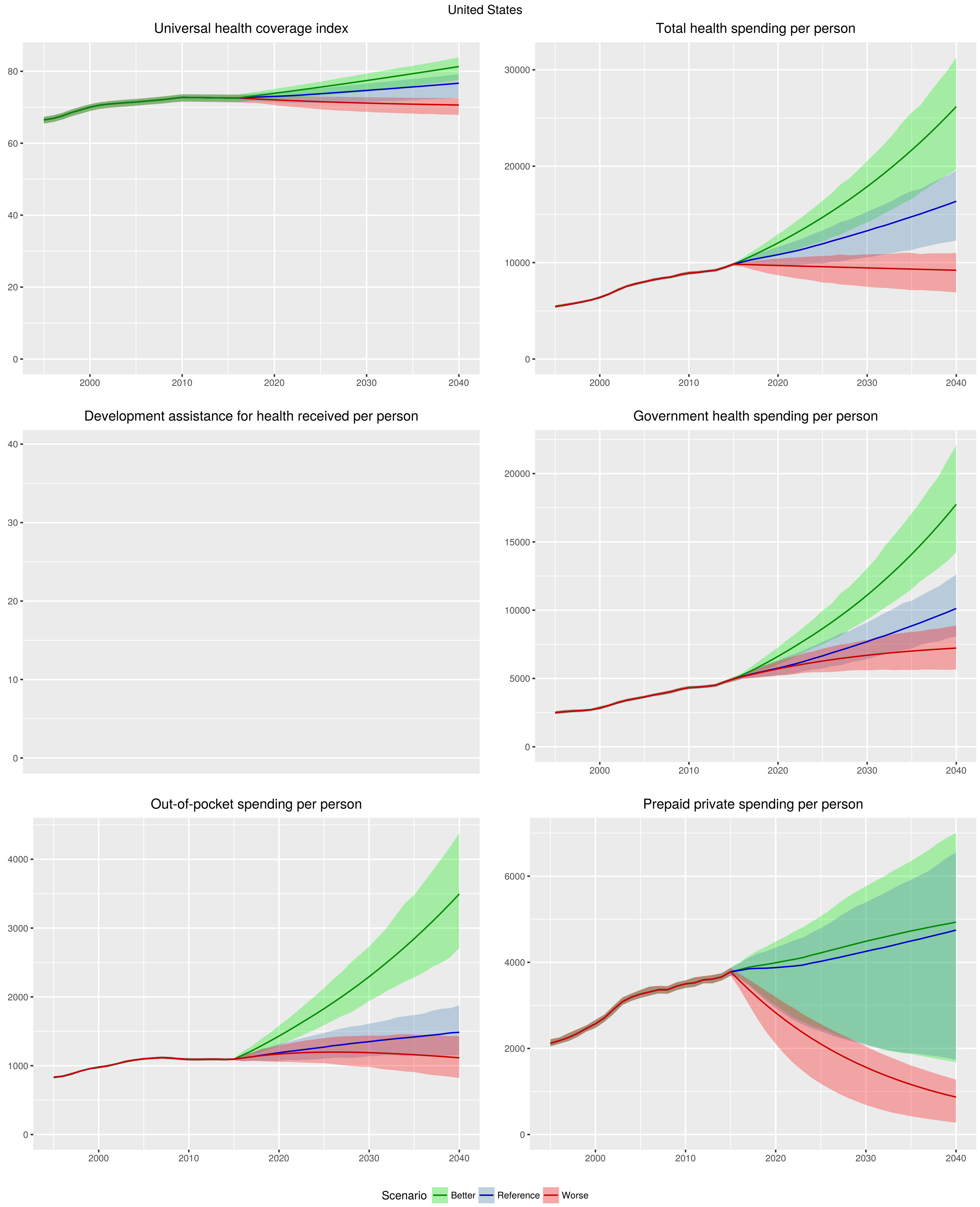
United States
Universal health coverage index
Total health spending per person
Development assistance for health received per person
Government health spending per person
Out-of-pocket spending per person
Prepaid private spending per person
Scenario
Better
Reference
Worse

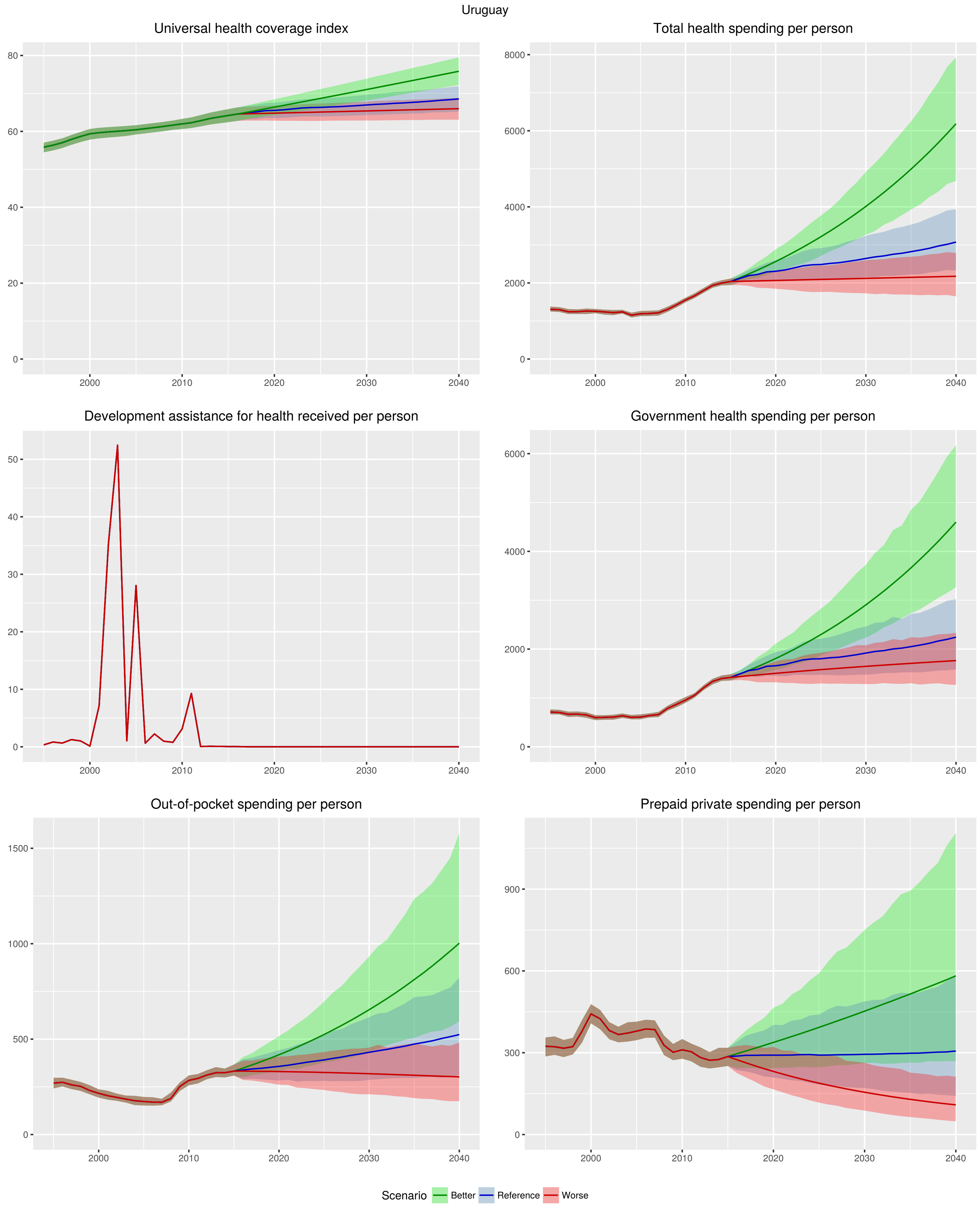
Uruguay
Universal health coverage index
Total health spending per person
Development assistance for health received per person
Government health spending per person
Out-of-pocket spending per person
Prepaid private spending per person
0
20
40
60
80
0
2000
4000
6000
8000
0
10
20
30
40
50
0
2000
4000
6000
0
500
1000
1500
0
300
600
900
2000
2010
2020
2030
2040
Scenario
Better
Reference
Worse

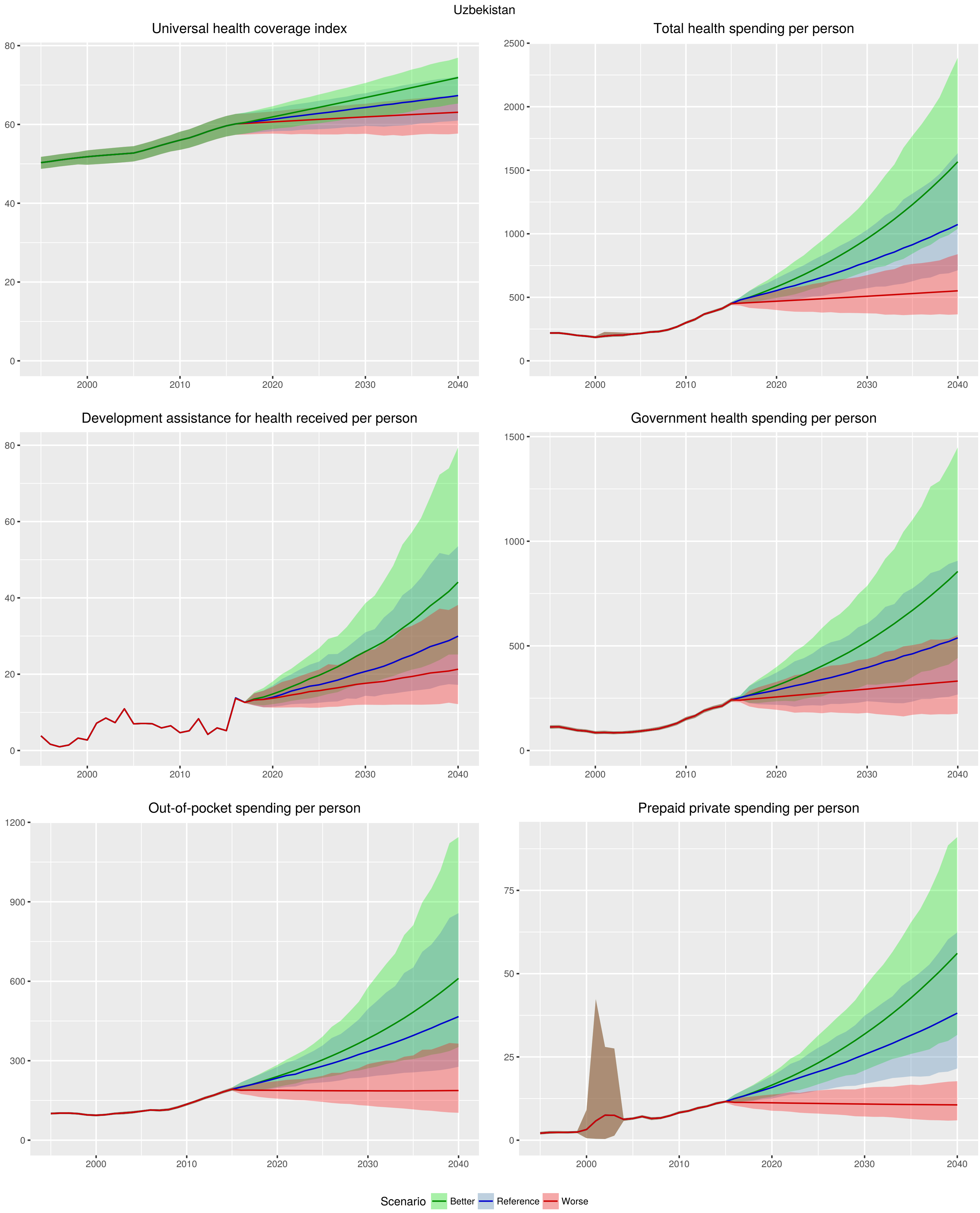

Uzbekistan
Universal health coverage index
Total health spending per person
Development assistance for health received per person
Government health spending per person
Out-of-pocket spending per person
Prepaid private spending per person
0
20
40
60
80
500
1000
1500
2000
2500
300
600
900
1200
25
50
75
2000
2010
2020
2030
2040
Scenario
Better
Reference
Worse

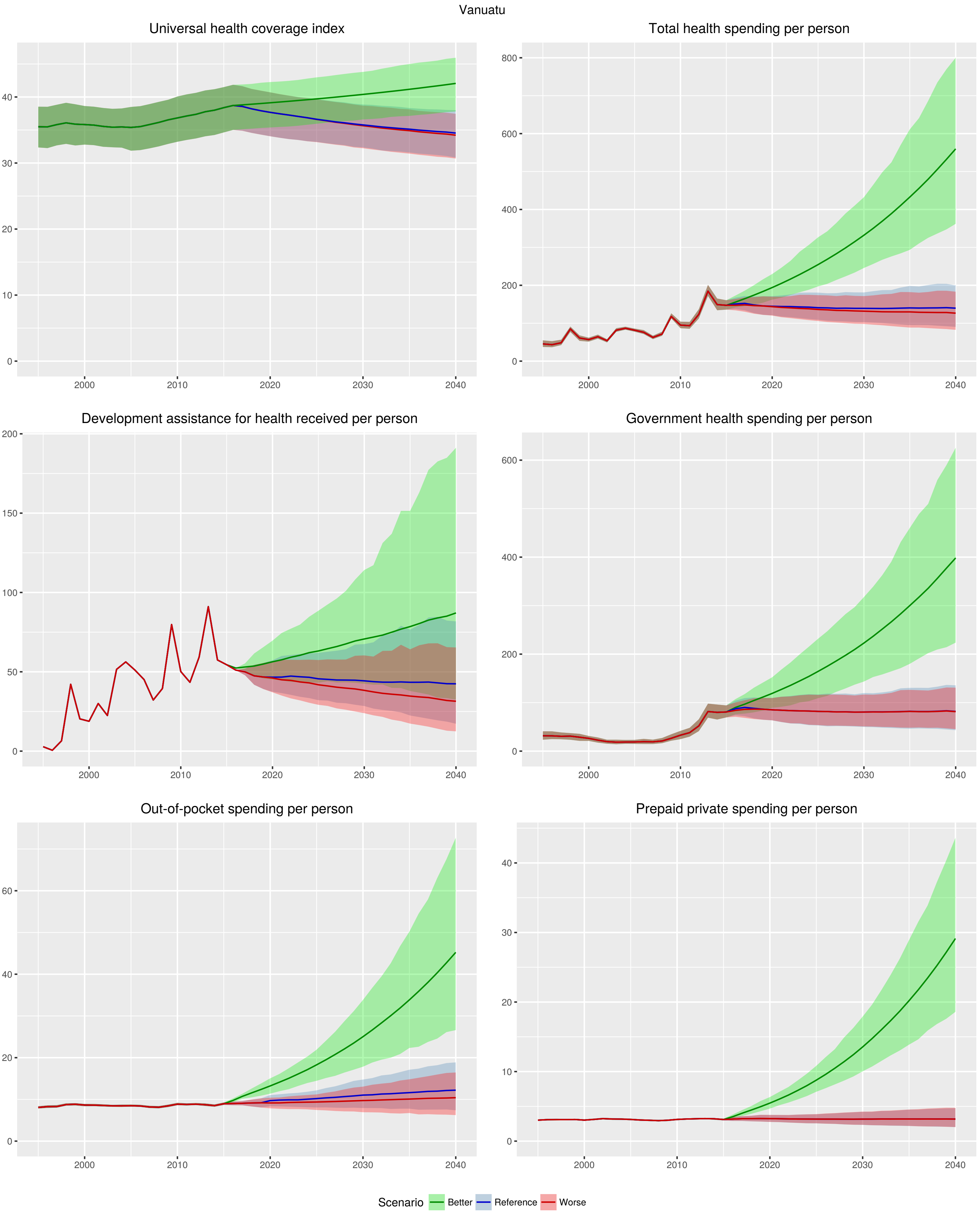
Vanuatu
Universal health coverage index
Total health spending per person
Development assistance for health received per person
Government health spending per person
Out-of-pocket spending per person
Prepaid private spending per person
Scenario
Better
Reference
Worse

Venezuela

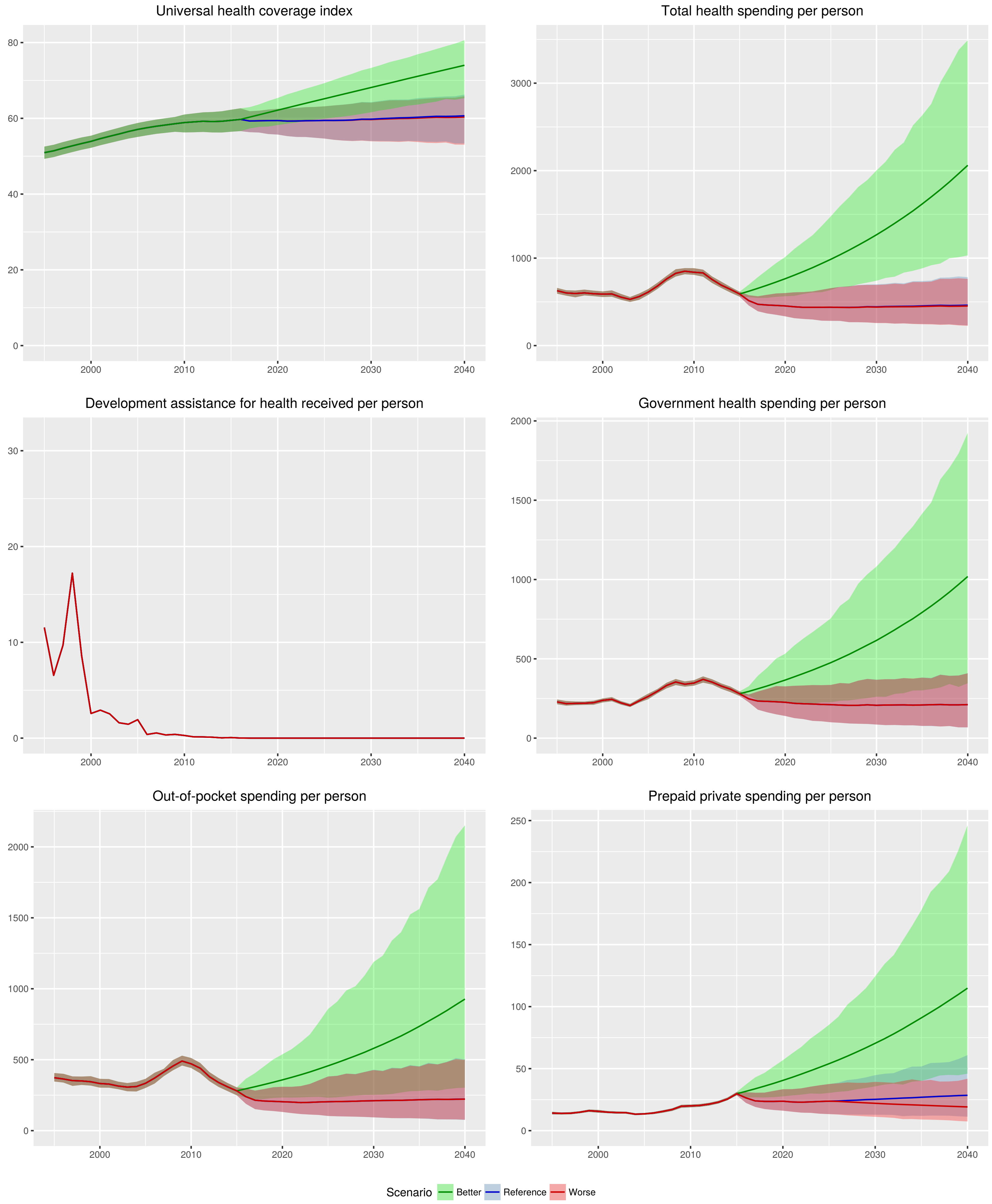
Universal health coverage index
Total health spending per person
Development assistance for health received per person
Government health spending per person
Out-of-pocket spending per person
Prepaid private spending per person
Scenario
Better
Reference
Worse

Vietnam

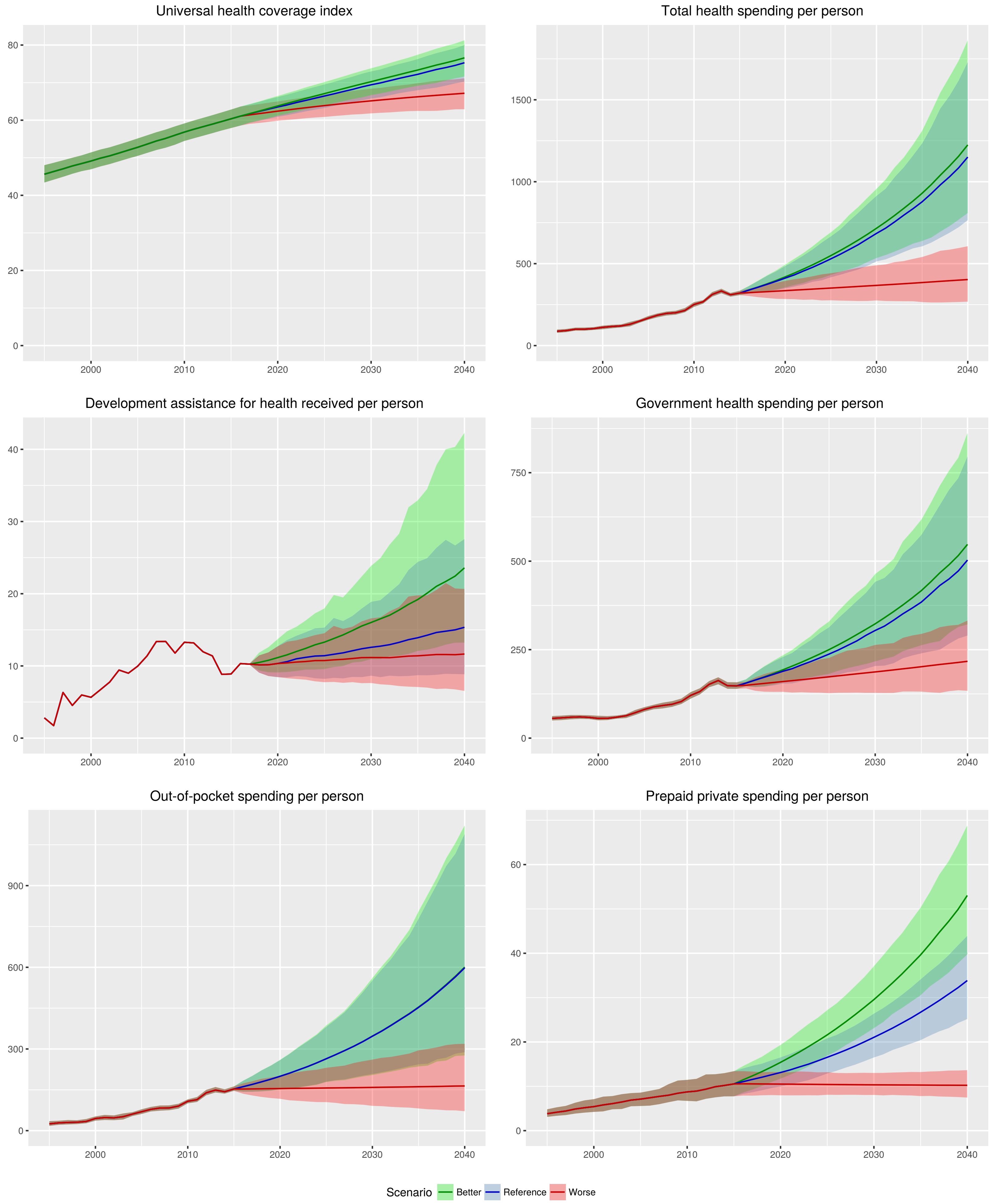
Universal health coverage index
Total health spending per person
Development assistance for health received per person
Government health spending per person
Out-of-pocket spending per person
Prepaid private spending per person
Scenario
Better
Reference
Worse

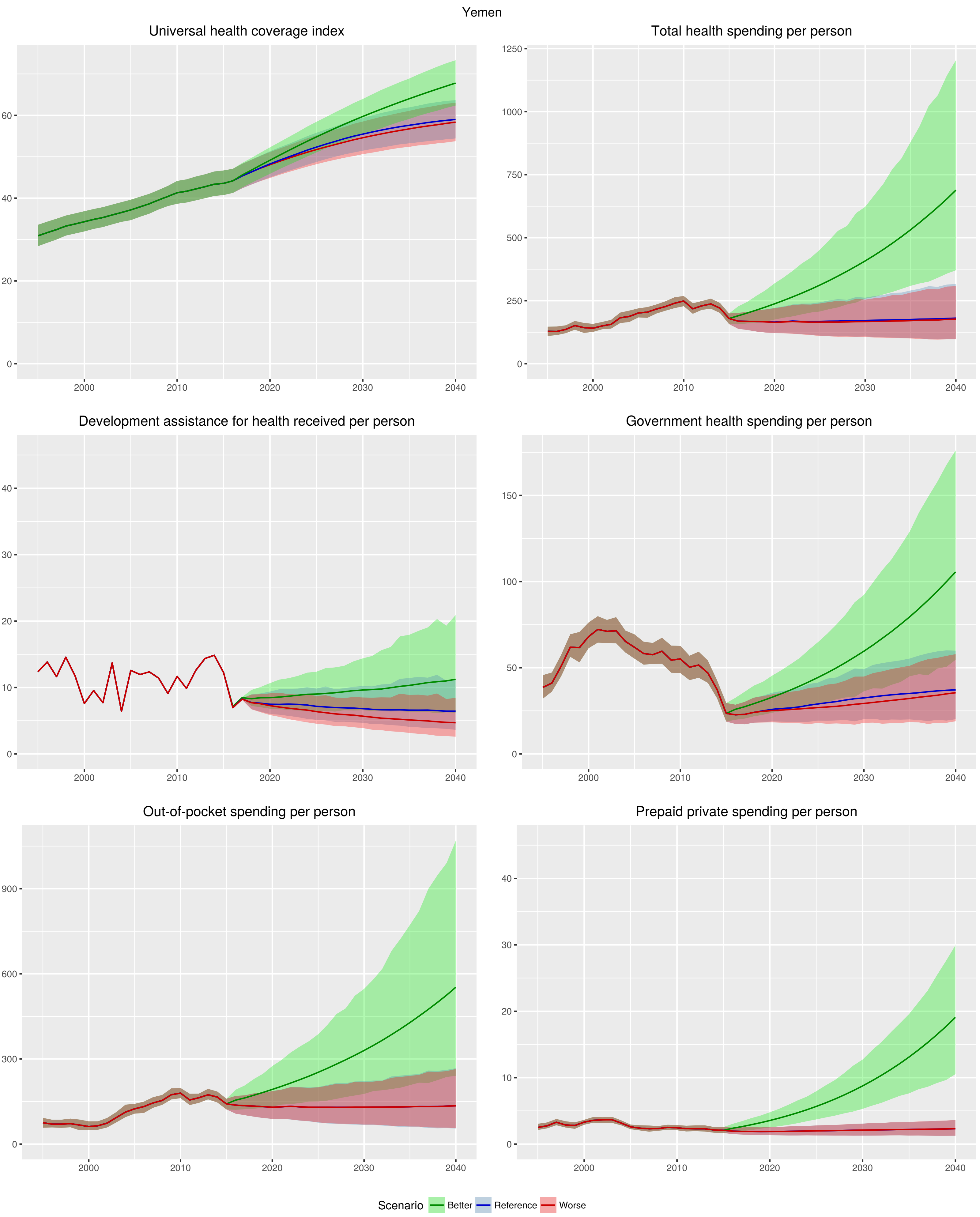
Yemen
Universal health coverage index
Total health spending per person
Development assistance for health received per person
Government health spending per person
Out-of-pocket spending per person
Prepaid private spending per person
Scenario
Better
Reference
Worse

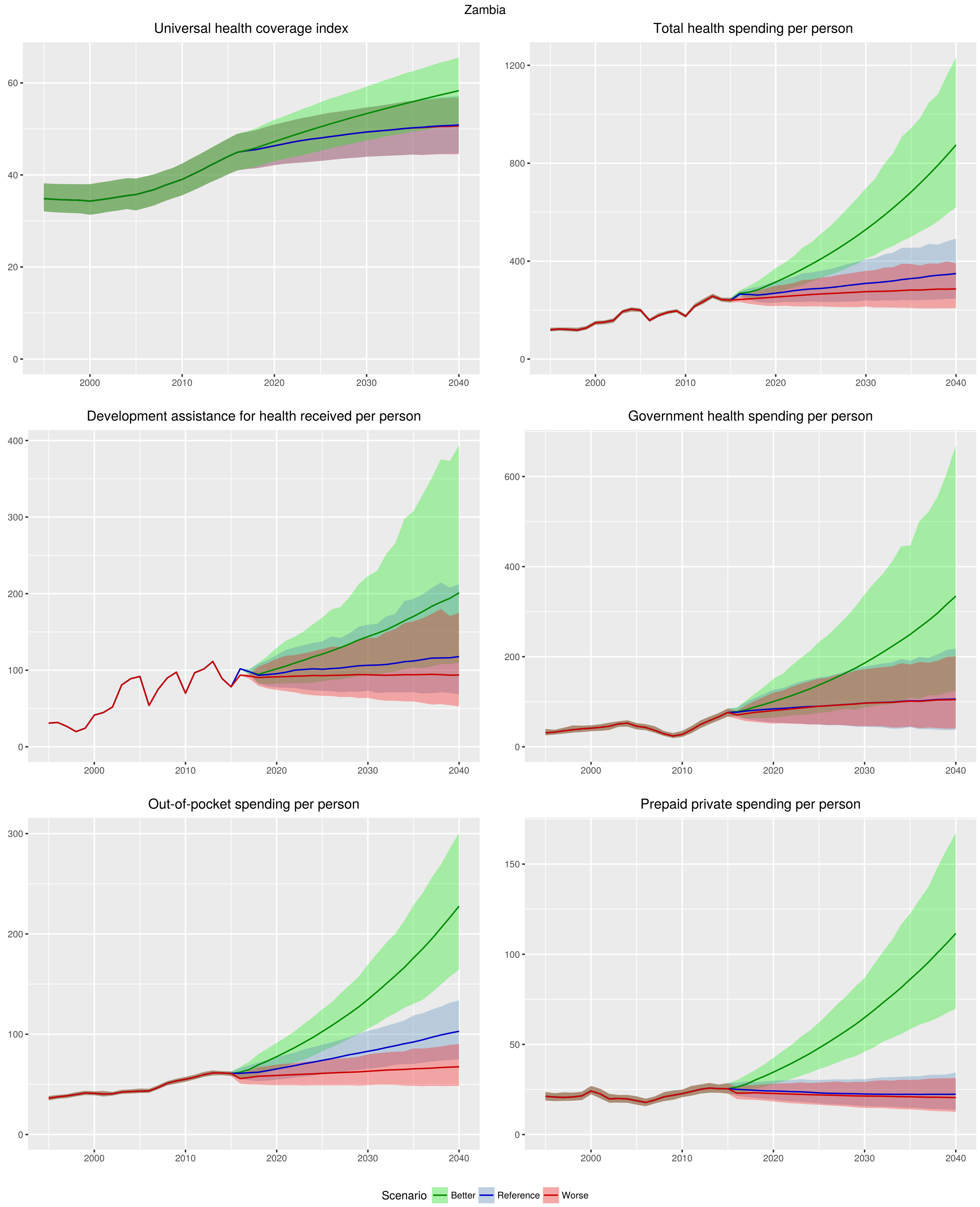
Zambia
Universal health coverage index
0
20
40
60
2000
2010
2020
2030
2040
Total health spending per person
0
400
800
1200
2000
2010
2020
2030
2040
Development assistance for health received per person
0
100
200
300
400
2000
2010
2020
2030
2040
Government health spending per person
0
200
400
600
2000
2010
2020
2030
2040
Out-of-pocket spending per person
0
100
200
300
2000
2010
2020
2030
2040
Prepaid private spending per person
0
50
100
150
2000
2010
2020
2030
2040
Scenario
Better
Reference
Worse

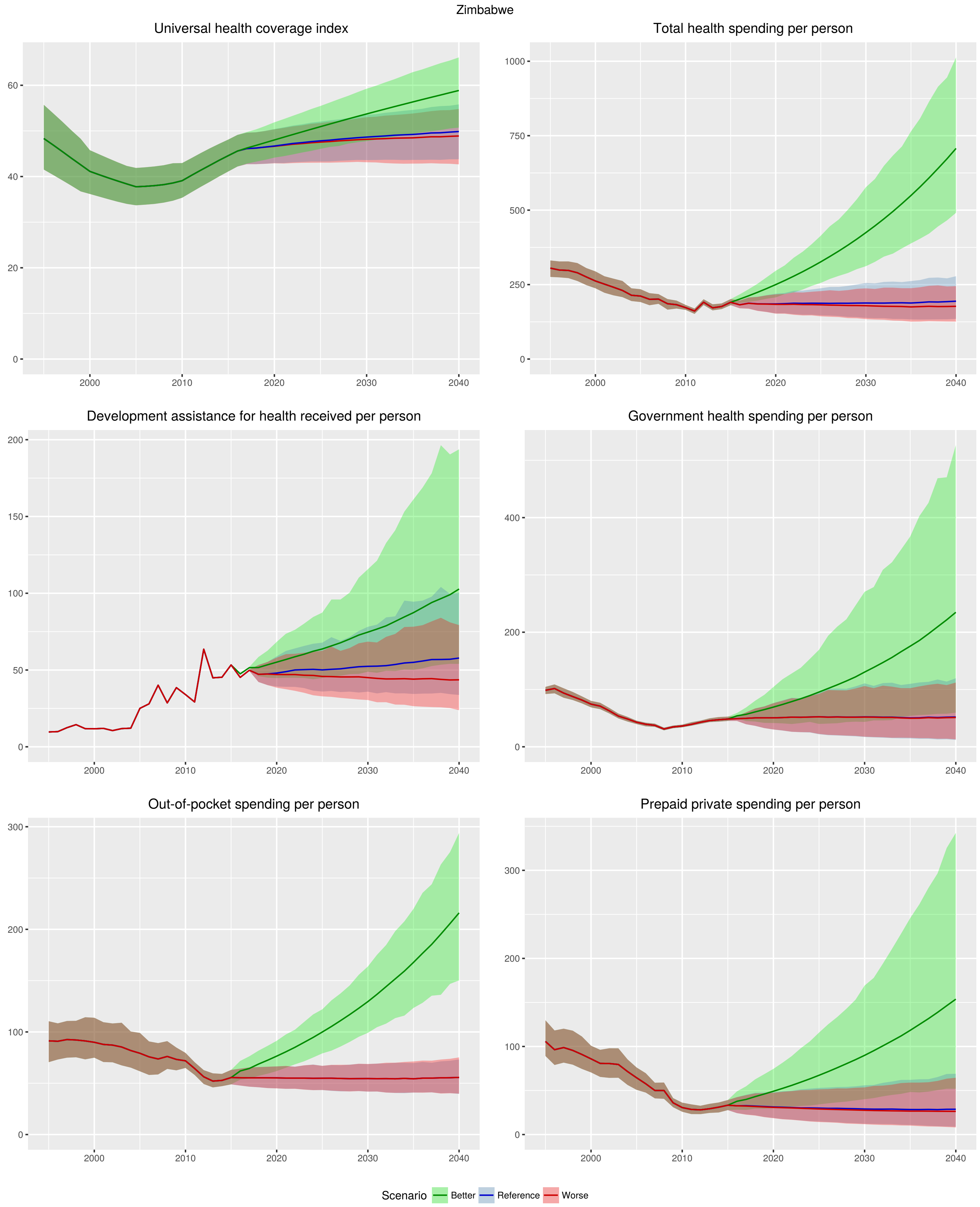
Zimbabwe
Universal health coverage index
Total health spending per person
Development assistance for health received per person
Government health spending per person
Out-of-pocket spending per person
Prepaid private spending per person
0
20
40
60
250
500
750
1000
50
100
150
200
300
2000
2010
2020
2030
2040
Scenario
Better
Reference
Worse
